# Hidden Treasures in “Ancient” Microarrays: Gene-Expression Portrays Biology and Potential Resistance Pathways of Major Lung Cancer Subtypes and Normal Tissue

**DOI:** 10.3389/fonc.2014.00251

**Published:** 2014-09-29

**Authors:** Konstantinos Kerkentzes, Vincenzo Lagani, Ioannis Tsamardinos, Mogens Vyberg, Oluf Dimitri Røe

**Affiliations:** ^1^Department of Computer Science, University of Crete, Heraklion, Greece; ^2^Institute of Computer Science, Foundation of Research and Technology – Hellas, Heraklion, Greece; ^3^Institute of Pathology, Aalborg University Hospital, Aalborg, Denmark; ^4^Department of Cancer Research and Molecular Medicine, Norwegian University of Science and Technology, Trondheim, Norway; ^5^Department of Oncology, Clinical Cancer Research Center, Aalborg University Hospital, Aalborg, Denmark; ^6^Cancer Clinic, Levanger Hospital, Nord-Trøndelag Health Trust, Levanger, Norway

**Keywords:** bioinformatics, carcinoid, lung adenocarcinoma, mesothelioma, microarray, small cell, squamous

## Abstract

**Objective:** Novel statistical methods and increasingly more accurate gene annotations can transform “old” biological data into a renewed source of knowledge with potential clinical relevance. Here, we provide an *in silico* proof-of-concept by extracting novel information from a high-quality mRNA expression dataset, originally published in 2001, using state-of-the-art bioinformatics approaches.

**Methods:** The dataset consists of histologically defined cases of lung adenocarcinoma (AD), squamous (SQ) cell carcinoma, small-cell lung cancer, carcinoid, metastasis (breast and colon AD), and normal lung specimens (203 samples in total). A battery of statistical tests was used for identifying differential gene expressions, diagnostic and prognostic genes, enriched gene ontologies, and signaling pathways.

**Results:** Our results showed that gene expressions faithfully recapitulate immunohistochemical subtype markers, as chromogranin A in carcinoids, cytokeratin 5, p63 in SQ, and TTF1 in non-squamous types. Moreover, biological information with putative clinical relevance was revealed as potentially novel diagnostic genes for each subtype with specificity 93–100% (AUC = 0.93–1.00). Cancer subtypes were characterized by (a) differential expression of treatment target genes as *TYMS*, *HER2*, and *HER3* and (b) overrepresentation of treatment-related pathways like cell cycle, DNA repair, and ERBB pathways. The vascular smooth muscle contraction, leukocyte trans-endothelial migration, and actin cytoskeleton pathways were overexpressed in normal tissue.

**Conclusion:** Reanalysis of this public dataset displayed the known biological features of lung cancer subtypes and revealed novel pathways of potentially clinical importance. The findings also support our hypothesis that even old omics data of high quality can be a source of significant biological information when appropriate bioinformatics methods are used.

## Introduction

Lung cancer is the cancer entity that takes most lives worldwide and overall 5-year survival is still only 15%, despite recent progress in targeted therapy in small subsets of patients (1). The need to identify subgroups with different biology and treatment resistance patterns has been an underlying theme in research, but translating this to the clinic has been very difficult due to the complexity and heterogeneity of the disease (2). The major histological lung cancer subgroups include adenocarcinoma (AD), squamous cell carcinoma (SQ), large cell carcinoma (LC), the carcinoids (COID), and small-cell lung cancer (SCLC) (3). There is an obvious lack of molecular knowledge on lung cancer (4, 5) and an urgent need to dissect the biological differences between/within types both for improving diagnosis, for detecting prognostic subgroups defined by novel molecular signatures and novel targets and strategies for personalized treatment.

More than a decade ago, genome-wide profiling was believed to allow detection of cancer subgroups and to be able to provide an answer to all these questions. In 2001, Bhattacharjee et al. proposed that the lung AD should be divided into four subgroups based on gene-expression profiles (6). These subgroup descriptions did not materialize into practice-changing information. However, several bioinformatics tools and resources were not yet developed. Particularly, gene annotation was not as complete and we did not have the same level of pathway insight. Could a fresh look into this material reveal new information? In this study, we reanalyzed the Bhattacharjee dataset *in silico* – still one of the largest, publicly available, high-quality microarray datasets on normal lung tissue and lung cancer histological subgroups (6). The aim was to characterize the classical histological subgroups in-depth based on differentially expressed genes, gene ontologies, and pathways, and comparing this with our own published mesothelioma dataset (7). In the AD group, we correlated gene expression to clinicopathological information and survival.

## Materials and Methods

### Data and preprocessing

**Figure 1:**
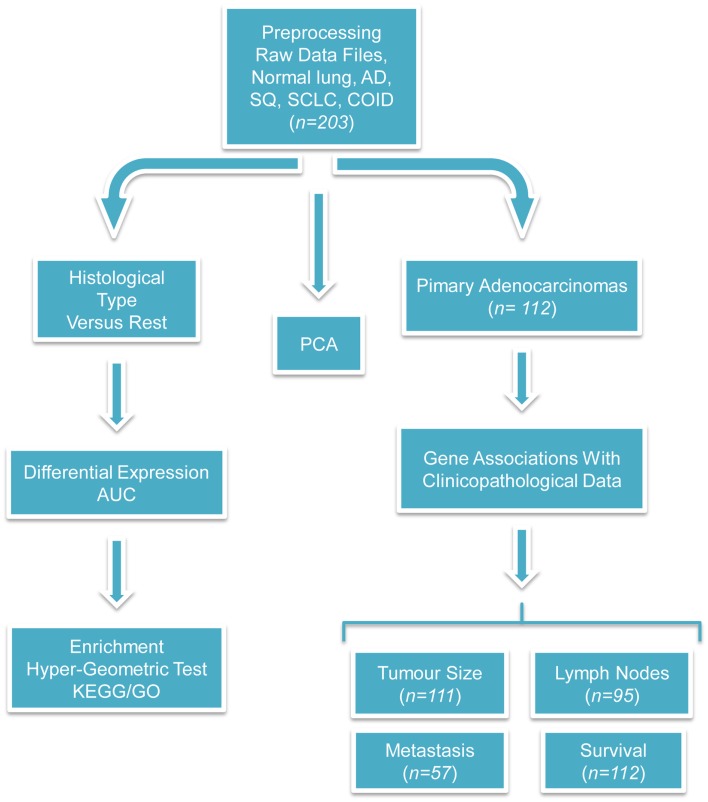
**Flow-chart of the performed analyses**. This figure illustrates the analyses performed during this study. The raw CEL files were obtained from the site of the authors of the original publication and were preprocessed with the RMA algorithm. Three different branches of analyses were performed; first, PCA was used for investigating the relationship among different types of tumors and against normal tissues. Second, differentially expressed and diagnostic genes were identified in each histological type versus the rest. On the basis of these lists of differentially expressed genes, enriched GO biological processes, and KEGG pathways were identified with the hyper-geometric test. Finally, the association between clinicopathological and transcriptional information were investigated in the primary adenocarcinoma samples. We tried to identify genes correlated with the tumor size, differentially expressed and diagnostic genes of lymph node status and metastasis, and single gene or gene signatures predictive of survival. The sample size differs because complete clinicopathological information was not always provided and in the survival analysis only the primary adenocarcinomas were taken into account.

Publicly available raw gene-expression profiling data (6) of 203 snap-frozen samples from lung tumors and normal tissue were obtained from http://www.broadinstitute.org/mpr/lung/. These data have not been submitted in any repository (e.g., ArrayExpress, Gene Expression Omnibus, DNA databank of Japan) probably because it was published as early as 2001. These included histologically defined AD (*n* = 126), SQ (*n* = 21), COID (*n* = 20), SCLC (*n* = 6) cases, metastasis from other ADs (*n* = 13, suspected to be extrapulmonary metastases), and normal lung specimens (*n* = 17). Of these, 125 AD samples were associated with clinical data and with histological slides from adjacent sections (6). The total RNA extracted from samples was used to generate cRNA target, subsequently hybridized to human U95A v2 oligonucleotide probe arrays of 12,600 transcripts/8655 unique genes (Affymetrix, Santa Clara, CA, USA) according to standard protocols (8). We calculated expression values from the raw data (CEL files) using the robust multi-array average (RMA) normalization approach as implemented in the Bioconductor’s “affy” package (9) and any probe with insufficient annotation was removed. When multiple probes were referring to the same gene, only the probe with the higher variation was retained. Principal components analysis (PCA) was performed and either the first two (2D) or three (3D) principal components were plotted in order to visualize a significant portion of the information on the data. A schematic representation of the different analyses performed on the lung cancer dataset in this study is provided (Figure 1).

### Identification of differentially expressed and diagnostic genes

For each diagnostic group (normal/AD/SQ/SCLC/COID), differentially expressed and diagnostic genes were analyzed in a “one vs. all the rest” fashion (e.g., normal versus the union of AD/SQ/SCLC/COID, SQ versus the union of normal/AD/SCLC/COID, etc.). For differential expression, linear models and empirical Bayes statistics from Bioconductor’s “limma” package (10, 11) were used and any gene with false discovery rate (FDR) adjusted *p*-value below 0.05 was considered significant. For identifying diagnostic genes, the receiver operating characteristic (ROC) – area under the curve (AUC) (12) for each gene was determined using its expression as a ranking criterion, and genes with high AUC were deemed as the most diagnostic.

### Pathway over/underrepresentation analysis

GO biological processes and KEGG pathways that were overrepresented or underrepresented for each diagnostic group were identified via a hyper-geometric statistical test as implemented in the hyperGTest R function (13). In our analyses, the hyper-geometric test was used for contrasting specific GO terms/KEGG pathways against sets of genes “of interest.” These sets were defined, in turn, as all differentially expressed genes, the upregulated genes, and the downregulated genes of each diagnostic group.

### Comparison with mesothelioma

Our previous study on microarray of mesothelioma versus parietal pleura has a gene list of differentially expressed genes as well as GO and KEGG pathways (7). We compared these findings with the lung cancer profiles to see possible similarities and differences. The mesothelioma versus normal pleura expression profile has been submitted to ArrayExpress registered with accession number E-MTAB-47.

### Correlation with phenotype characteristics and survival

We used the supplementary information available for the ADs samples (*n* = 126; www.pnas.org/content/suppl/2001/11/13/191502998.DC1/SampleData.xls), for investigating possible associations among gene expressions and different phenotype. We correlated gene expression with tumor size by performing Spearman’s rank correlation test. The resulted *p*-values were corrected through Storey’s *q*-value method (14) (as implemented in Bioconductor’s “*q*-value” package), where a cutoff threshold of 0.05 was used to control the FDR.

Differentially expressed and diagnostic genes for the metastatic vs. primary AD tumors and for each lymph node status were identified as described in Section “Identification of Differentially Expressed and Diagnostic Genes.”

A complex analysis protocol was applied in order to identify gene signatures able to predict survival in AD. All the available clinicopathological information as sex, age, and smoking were included in the analysis, and missing values were replaced with the mean of their respective variables. The experimentation protocol from Lagani et al. (15) was applied. Briefly, this protocol consists of several multivariate regression and feature selection algorithms applied with a nested N-fold cross-validation procedure. The N-fold cross validation consists in subdividing the available sample in N-folds; in turn, each fold is held out for testing purposes while the remaining data (training set) is employed for model selection and fitting. The nested-cross validation is a generalization of the standard cross validation, where an inner cross validation is applied in the training set in order to select the best model. The performance estimation provided by nested-cross validation is known to be more accurate than the ones provided by simple cross validation (16).

### Validation on external datasets

The validation on the robustness and reproducibility of our methods was tested by analysis of two additional, more recent lung cancer datasets (17, 18), as detailed in Text S1 in Supplementary Material. In brief, three ordered gene lists were produced for each dataset, one based on FDR adjusted *p*-value, one based on log-fold change, and one based on AUC. A statistical methods based on differentially weighting overlaps among lists depending by whether they occur at the extremes of in the middle of the lists [Bioconductor’s package “OrderedList,” (19)] was employed to test and measure the similarity between the lists of each ordering scheme.

### Immunohistochemistry

The immunohistochemistry images are from our institution, Institute of Pathology, Aalborg University Hospital and are representative for each diagnostic antibody. The microphotos are presented in accordance with the rules of the Review Board at our institution.

## Results

**Figure 2:**
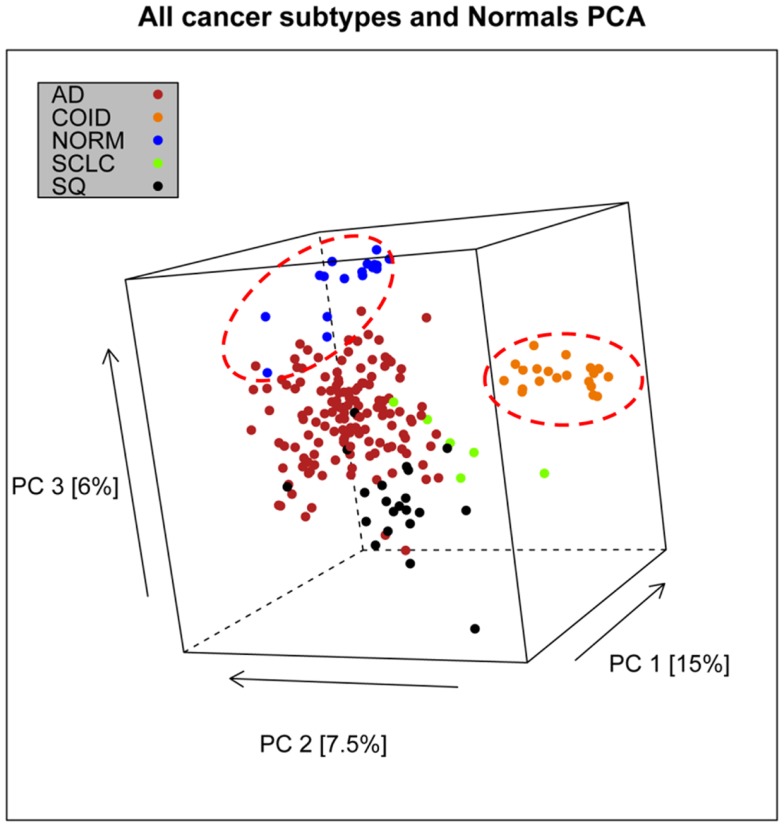
**Distribution of normal tissues and cancer subtypes in the principal component space**. This visualization shows that normal and carcinoids (COID) have distinct expression profiles, while adenocarcinomas (AD) overlap with both squamous (SQ) and small-cell carcinoma (SCLC).

**Figure 3:**
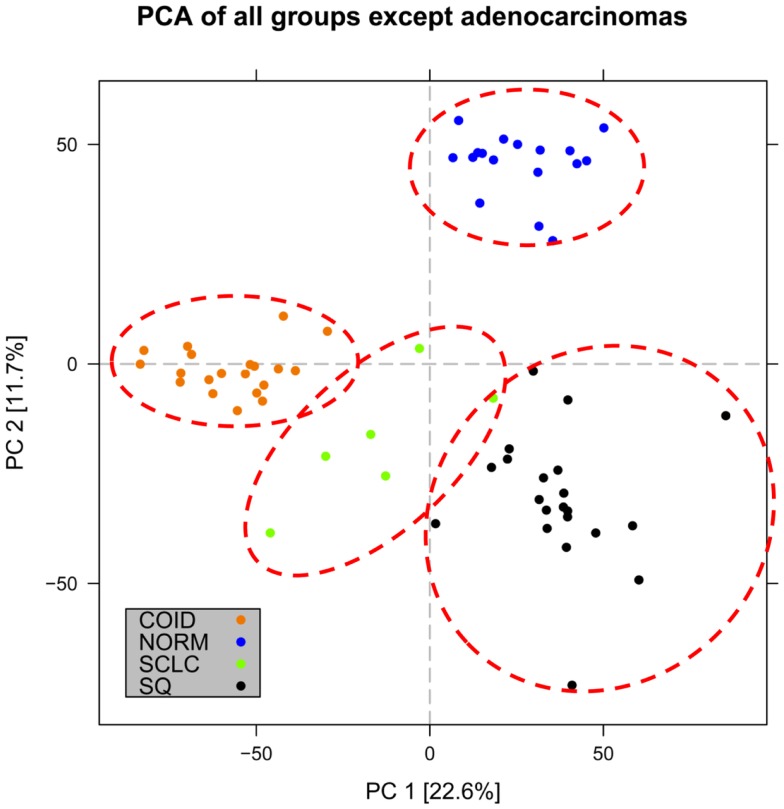
**Distribution of normal tissues and cancer subtypes except adenocarcinoma in the principal component space**. Small-cell, squamous, and carcinoid subgroups show distinctive expression profile when the adenocarcinomas are omitted, indicating that adenocarcinoma is the most heterogeneous group.

**Figure 4:**
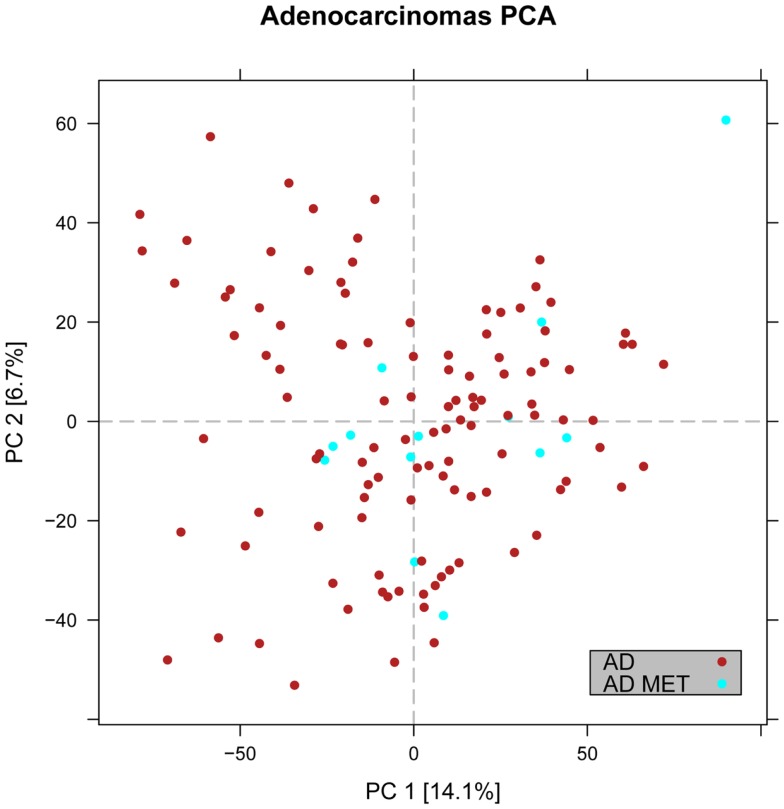
**Distribution of primary versus metastatic adenocarcinoma samples in the principal component space**. PCA plot showing the expression of the adenocarcinoma metastases to the lung from other locations (light blue) in relation to primary lung cancers including adenocarcinoma (red). The general expression profile of the adenocarcinoma metastases versus primary adenocarcinomas did not vary significantly.

In the first analysis, differential gene expression of each tissue type versus all the other types was determined. Data showed that each of the tumor subtypes and normal tissue had >1000 differentially expressed genes, except the SCLC with 842 genes (Table 1; File S1 in Supplementary Material). Likewise each tissue type had tens of genes with an AUC greater than 0.8, indicating a strong discriminatory or diagnostic value of these genes (File S2 in Supplementary Material). Moreover, there were several significantly over and underrepresented ontologies and pathways in GO and KEGG (Files S3 and S4 in Supplementary Material). There was a distinctive expression profile of the normal tissue, SCLC, SQ, and COID as visualized by principal component analysis (PCA) in 2D and 3D. In contrast, AD showed high degree of heterogeneity and overlapped the expressions of SQ and SCLC (Figures 2 and 3). In the PCA, the expression of the AD metastases to the lung from other locations did not vary significantly from the primary lung ADs (Figure 4). However, between the primary AD and metastatic groups there were 267 differentially expressed genes with FDR adjusted *p*-value <0.05. Below the main results of each tissue type is described.

**Table 1:** **Differentially regulated genes between each diagnostic group versus the rest (*p* < 0.05)**.

Genes	Normal	Lung AD	SQ	COID	SCLC
Up	1338	1527	1611	3247	493
Down	1680	1975	2064	2130	349

*Up, upregulated; Down, downregulated; AD, adenocarcinoma; SQ, squamous cell carcinoma; COID, carcinoids; SCLC, small-cell lung cancer*.

### Normal lung

The top five diagnostic genes were all overexpressed, *PECAM1* (AUC >0.99), *TGFBR2*, *CDH5*, *AGER*, and *TCF21* (File S2 in Supplementary Material). These results were similar with the results from the original paper that found all these genes overexpressed in the normal samples as well (6). The differentially expressed genes of normal lung tissue versus all types of lung tumors were overrepresented in several GO processes as actin cytoskeleton organization and regulation of developmental process and underrepresented in such as mRNA metabolic process and G1/S transition checkpoint (File S3 in Supplementary Material). Similar KEGG pathways were overrepresented, such as regulation of actin cytoskeleton, vascular smooth muscle contraction, and leukocyte trans-endothelial migration (File S4 in Supplementary Material). Interestingly, these pathways were downregulated in tumors.

### Non-small cell lung cancer

#### Squamous cell lung cancer

The top five genes with the highest predictive value were *KRT5* (AUC >0.98), *ATP11A*, *DSP*, *UBXN7*, and *RHOBTB2* from which *ATP11A* and *RHOBTB2* were underexpressed and the rest were overexpressed. The GO of the differentially expressed genes of SQ included M/G1 transition and DNA repair, while in KEGG several DNA repair mechanisms where overexpressed. Among the underrepresented was cellular response to cytokine stimulus and similarly in KEGG, cytokine–cytokine receptor interaction.

#### Adenocarcinoma

Among the top five genes with the highest diagnostic value *ERBB2* (*HER2*) (AUC >0.93), *GALE*, *PRSS8*, and *PYCR1* were overexpressed and *PGAP1* was downregulated. The GO of the differentially expressed genes of the primary lung AD showed antigen procession and presentation, identical as in KEGG, while response to bacterium and cell cycle was underrepresented in GO and KEGG, respectively (Files S3 and S4 in Supplementary Material).

### Small cell lung cancer

The top five genes were all overexpressed, namely the *ISL1* (AUC = 1.00), *HMGN2*, *CDKN2C*, *STMN1*, and *ACYP1*. The GO of the differentially expressed genes of SCLC included mitotic cell cycle and DNA repair, while regulation of several metabolic processes was underrepresented. In KEGG, cell cycle, several DNA repair mechanisms and one carbon pool by folate was overrepresented while cytokine–cytokine receptor interaction and proteasome were among the underrepresented pathways (Files S3 and S4 in Supplementary Material).

### Carcinoids

The lung COID was the only tumor entity that separated completely from the rest of the tumors and normal tissue by PCA and AUC (Figures 2 and 3). There were seven genes with a diagnostic AUC = 1.00, namely *VAMP2*, *MYT1L*, *GPRASP1*, *TSPYL2*, *CHGA*, *MAPRE3*, and *SNAP91*, all overexpressed. Differentially expressed genes of COID in GO showed overrepresentation of regulation of angiogenesis and Hippo signaling cascade, and upregulation of synaptic transmission and neurological system process. In KEGG pathways, the lysosome, the SCLC, and phagosome pathways were overrepresented, and neuroactive-ligand receptor interaction neurological disease pathways such as Parkinson’s, Huntington’s, and Alzheimers’s were significantly upregulated. Underrepresentation of GO included response to stress and immune-related ontologies while in KEGG the ribosome, the ECM-receptor interaction, phagosome, and the leukocyte trans-endothelial migration was downregulated (Files S3 and S4 in Supplementary Material).

### Primary lung adenocarcinoma versus lung metastases

In the PCA, we observed that the AD metastases of the lung from other sites had an overlapping and overall similar gene-expression profile with the primary lung AD (Figure 4). However, there were 267 differentially expressed genes, where 207 were downregulated in the primary lung AD (File S1 in Supplementary Material). The overexpressed gene with highest discriminatory power was the *SLC26A3* with AUC >0.88. In KEGG pathways, key genes of the PI3K-Akt and Jak-Stat, e.g., the oncogenes *MYC* and *MYB* and in the mTOR signaling pathways the *AKT1*, *MTOR*, *OSMR*, and *PKN1* were upregulated.

### Clinicopathological correlation and survival in adenocarcinomas

We performed a Spearman rank test using only the primary ADs of the lung where clinical data were available (*n* = 112) to examine the correlation of gene expression and tumor size in centimeters. There were four genes with a significant *p*-value, and only one of them, the *NR2C1* was positively correlated with tumor size (Table 2). Analysis of N-status revealed only in the N0 cases versus N1–N3 two differentially expressed genes (*P* < 0.05) with an AUC of >0.74, *FLNB* and *ILVBL*, both downregulated. There were no differentially expressed gene related to metastasis, but there were very few cases in the M1 group (*n* = 4), most where MX (undefined M status).

**Table 2:** **Genes significantly correlated with primary lung adenocarcinoma tumor size**.

Symbol	Correlation	Adjusted *p*-value <0.05
*NR2C1*	0.4527	0.00564
*DLGAP1*	−0.4255	0.01395
*ZNF259P1*	−0.3952	0.0369
*CD22*	−0.3946	0.0369

*Positive correlation implies higher expression in larger tumors*.

The survival analysis did not achieve any notable results as no single gene neither gene signature could predict survival in this cohort. Particularly, we measured predictive performances in terms of concordance index (CI) and integrated brier score (IBS) (20). The first metric has an interpretation similar to the AUC and provides an estimation of the probability of correctly ranking two randomly chosen subjects according to their respective risk of experiencing the event of interest. The IBS evaluates the calibration of the predicted survival curves. In all our analyses, the predictive performances of any single gene and gene signature (as measured through nested-cross validation, see “Materials and Methods”) were around 0.5 (CI) and 0.25 (IBS), which corresponds to random guessing.

## Discussion

New insight in molecular subtyping is of increasing importance in the era of personalized medicine, as advances in high-throughput technology have revealed distinct molecular differences between and within tumor types and even a high degree of intratumoral heterogeneity. Here, we provide a proof-of-concept of how a well curated set of expression profiles on normal lung tissues and lung cancer subtypes can be used to make biologically and clinically relevant assumptions on the phenotype based on the transcription profiles.

Through more than four decades, patients with lung cancer were generally treated uniformly in two large groups, the non-small cell lung cancers (NSCLC) and the SCLC. Current progress include more efficient chemotherapy compounds for so-called non-squamous carcinomas, including the multifolate inhibitor pemetrexed (21), and smaller subgroups with molecular changes such as EGFR mutations and ALK rearrangements that significantly predict the effect of biological targeted therapies (22, 23). However, the SQ, LC, SCLC, or COID and the majority of the AD still have no molecular prognostic or predictive markers in clinical use (4, 5) and most treatment modalities are stratified according to clinical staging as TNM (Tumor, Node, Metastasis), Stage I–IV and Karnofsky or WHO performance status.

In lung cancer, mRNA expression profiling showed promising results in defining some subgroups more than a decade ago, but surprisingly this information has still not led to any radical path to a personalized and effective treatment. Performing high-quality microarray analysis of human tissue is not easy, and several factors need to be optimal at sample collection, processing, and analysis, that may account for large variations of the results. Moreover, they are both time consuming and costly. However, currently there are thousands of public datasets on human material, and as both gene function knowledge and bioinformatics methods have evolved, these datasets may be a valuable source of new knowledge.

### Selection of dataset and idea of analysis

The dataset used was from 2001, but was elected due to the unusual high number of participants where all the large subgroups (except the large cell type) were represented, the completeness of the clinical data, as well as the quality of the research center. The main focus of the original publication was to present several new molecular subgroups of lung AD and pinned out one subtype with less favorable prognosis. We did not try to recapitulate their findings, but we observed that some of the differences between the classical subgroups in their analysis were similar to our findings [Figure 1 in Ref. (6)]. The main idea of our analysis was to identify the differential and diagnostic genes for each classical tissue type versus all the other groups, hereby trying to define the most specific molecular characteristics of each tissue type. The results showed a high degree of coherence between our findings and previous knowledge, but also revealed novel potential biomarker and target genes and pathways.

### Validation on immunohistochemical markers

**Figure 5:**
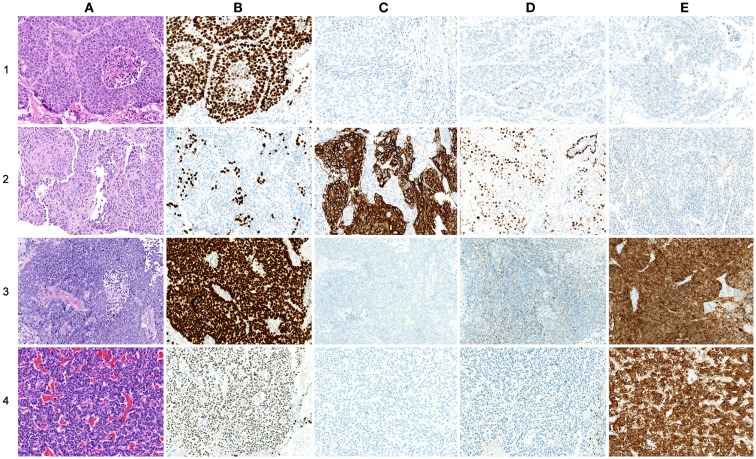
**Comparison of protein and gene-expression patterns in primary lung neoplasias**. **(A)** hematoxylin and eosin (H&E), **(B)** thyroid transcription factor-1 (TTF1), **(C)** cytokeratin 5 (CK5), **(D)** p63, and **(E)** synaptophysin (SNP). All photos ×200. (1) Adenocarcinoma, **(A)** H&E showing low differentiation with solid growth, **(B)** TTF1 showing strong nuclear staining of all tumor cells, **(C)** CK5 showing no cytoplasmic staining of tumor cells, **(D)** p63 showing nuclear staining of a few scattered tumor cells, and **(E)** SNP showing cytoplasmic staining of a few scattered tumor cells. (2) Squamous cell carcinoma, **(A)** H&E showing solid growth with slight squamous maturation to the left, **(B)** TTF1 showing no nuclear staining of tumor cells, scattered entrapped alveolar cells with normal TTF1 expression is seen, **(C)** CK5 showing strong cytoplasmic staining of most tumor cells, **(E)** p63 showing widespread nuclear staining of tumor cells, in the upper right corner an entrapped bronchiolus with normal p63 expression of basal cells is seen, and **(E)** SNP showing no cytoplasmic staining of tumor cells. (3) Small-cell carcinoma, **(A)** H&E showing sheet-like growth, **(B)** TTF1 showing very strong nuclear staining of tumor cells, **(C)** CK5 showing no staining of tumor cells, **(D)** p63 showing widespread but weak nuclear staining of tumor cells, and **(E)** SNP showing strong cytoplasmic staining of tumor cells. (4) Carcinoid, **(A)** H&E showing trabecular growth, **(B)** TTF1 showing moderate nuclear staining of most tumor cells, **(C)** CK5 showing no cytoplasmic staining of tumor cells, **(D)** p63 showing no nuclear staining of tumor cells; a few entrapped alveolar cells are positive, and **(E)** SNP showing strong cytoplasmic staining of tumor cells.

As histopathology and immunohistochemistry is the gold standard of cancer diagnosis, we validated our results by comparing the diagnostic gene results (File S2 in Supplementary Material) with the most common negative and positive immunohistochemical markers (proteins) for each subtype used for diagnosis in the clinic. These clinical diagnostic immunohistochemical markers are already validated through several studies, typically with sensitivity >80–90% (24–30). This comparison revealed a quite impressive overlap where genes encoding the diagnostic proteins were found in the top of their respective AUC list (Table 3; Figure 5). These included chromogranin A (*CHGA*) and synaptophysin (*SYN*) for COID, cytokeratin 5 (*KRT5*) and p63/p40 (*TP63*) for SQ, thyroidea trancription factor 1 (*TTF1*, synonymous with *NKX2-1*) for AD, and its homolog (*NKX2-2*) for SCLC (24–28). Interestingly, we also detected that the *NKX2-1* encoding *TTF1*, the most specific marker for cancers originating from the lung, was downregulated in SQ, in line with recent knowledge of SQ immunomarkers (28). In line with our findings, a large immunohistochemical study verified that a two-marker panel (*TTF1*/p63) is sufficient for subtyping of the majority of tumors as AD or SQ (29). Moreover, another obviously very expensive and time-consuming study on lung cancer subtypes testing >1000 cases with 108 antibodies got similar results, showing a five protein signature that could also separate cases that were undefined after *TTF1*/p63 analysis. This signature included the SQ positive markers *KRT5* (CK5), *TRIM29*, and *SLC7A5* (AUC 0.98, 0.93, and 0.83, respectively, in our analysis) and the AD positive markers *MUC1* and *CEACAM5* (AUC 0.81 and 0.78, respectively, in our analysis) (30).

**Table 3:** **Immunomarker occurrence expressed as percentage of cases in lung adenocarcinoma, squamous cell carcinoma, small-cell carcinoma, and carcinoid**.

Lung cancer	CGA (%)	*CHGA*, AUC	CK5 (%)	*KRT5*, AUC	p63/p40 (%)	*TP63*, AUC	SNP (%)	*SYP*, AUC	TTF1 (%)	NKX2–1/TTF1, AUC
AD	2	0.59D	5–30^a^^,^^b^	0.71D	5–30^a^^,^^b^	0.72D	10	0.56D	60–90	0.76U
SQ	0	ND	90–100	**0.98U**	90–100	**0.89U**	0	0.78D	0–20^a^	**0.89D**
SCLC	70–90	ND	0–5	ND	0–5	ND	70–90	ND	70–100	0.85U (*NKX2*–2)
COID	95	1,0U	0	ND	0	ND	100	0.99U	50–70	0.72D

^a^Predominantly focal reactions

*^b^p40 virtually negative in adenocarcinoma*.

*Values are based on recent large studies [Ref. (24–30)]. For each immunomarker protein, the diagnostic power of the respective gene (expressed in terms of AUC) is reported in blue*.

*CGA, chromogranin A; CHGA, CGA gene; CK5, cytokeratin 5; KRT5, CK5 gene; p63/p40, tumor protein p63/isoform of p63; TP63, p63 gene; SNP, synaptophysin; TTF1, thyroidea transcription factor 1; NKX2-1, TTF1 encoding gene; NKX2-2, homolog of NKX2; D, downregulated; U, upregulated; ND, not differentially expressed; COID, carcinoids; AD, adenocarcinoma; SCLC, small-cell lung cancer; SQ, squamous cell lung cancer*.

However, several of the top genes diagnosing each subgroup have not been described in the literature for lung cancer, as the neuroendocrine marker *ISL1* (insulin gene enhancer protein ISL-1) for the SCLC, the *ATP11A* (ATPase, class VI, type 11A) for SQ, *GALE* (UDP-galactose-4-epimerase) for the AD, and *VAMP2* (vesicle-associated membrane protein 2/synaptobrevin 2) for the COID (see Results and Supplementary Material). Currently, some of these genes are validated in tumors in an ongoing collaboration project.

### Validation on external datasets

In order to validate the robustness of our methods, two more datasets were retrieved, one published in 2005 (17) and one in 2010 (18), where the second dataset was obtained through the ExpressionBlast tool (31). Unfortunately, we were not able to identify any dataset with the exact cancer subtypes as in the Bhattacharjee et al. study, and thus a head-to-head comparison of our results was not possible. Due to this limitation, in each dataset we only contrasted the AD versus the healthy tissue samples. Moreover, since the measurements in each study were performed on the Affymetrix platform but different chip types, HGU-95Av2, HGU-133A, and HGU-133plus2, only the common genes of all three datasets were used (*n* = 8556). The genes of each study were ranked according to different criteria (FDR adjusted *p*-value for differential expression, log-fold change, and AUC). These ordered lists were compared across studies with a statistical method devised for evaluating similarity in gene ranks (19). In each pairwise comparison, the statistical tests rejected the null-hypothesis of ranks being different, thus indicating that results obtained on the Bhattacharjee et al. study is reproducible on other datasets (see Text S1 in Supplementary Material).

### Gene ontology and pathway analysis

Among the abundant gene and pathway information acquired through this quantitative analysis, several genes, and pathways recapitulating known phenotypes were detected, such as resistance to known chemotherapy regimens, targeted treatments, and tumor aggressiveness. Some of the gene ontologies/pathways with highest differential overrepresentation of genes will be discussed below. We observed that there were some similarities between these results and some of the finding of our previous study (7) on mesothelioma versus parietal pleura (normal mesothelial tissue). Thus, we also contrasted our current results against the key pathways that are differentially expressed in this treatment-refractory thoracic cancer.

#### Cell cycle

**Figure 6:**
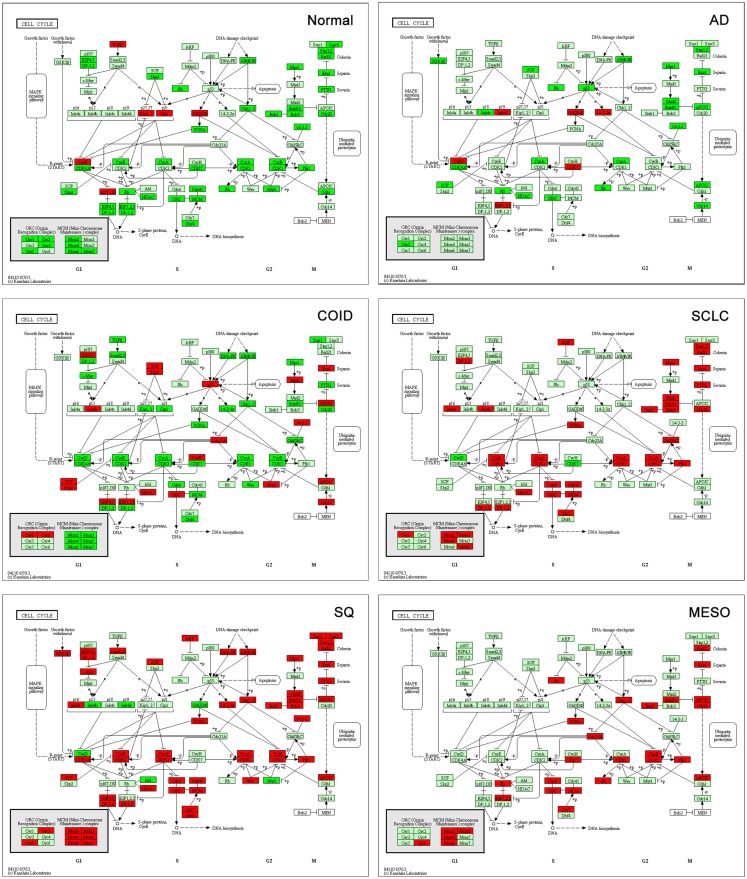
**Cell cycle pathway**. Tumor suppressor genes (CDKN1A/p21, CDKN1C/p57, RBL2/p130) and the cyclin D family were overexpressed in normal lung tissue while there was type-specific overexpression of oncogenes, cyclins, cyclin dependent kinases, and other cell cycle driving genes in tumors. Genes belonging to the mini-chromosome maintenance complex were overexpressed in squamous and small-cell lung cancer, as well as in mesothelioma. The same genes were not differentially expressed in AD and downregulated in COID. Fifty-six out of 63 genes in the Cell Cycle pathway were overexpressed in SQ, including CCNE/CDK2, CCNA/CDK2, CCNA/CDK1, CCNB/CDK1 complexes, BUB1, BUB1B, BUB3, and MYC oncogenes, damage response genes (ATR, CHEK1/2) and five genes of the 14-3-3 family. Red is overexpressed, dark green downregulated. SQ, squamous type; AD, adenocarcinoma; SCLC, small-cell lung cancer; COID, carcinoid; MESO, mesothelioma.

Analysis of cell cycle pathway in KEGG of each differential list of genes showed that in normal lung tissue only tumor suppressor genes (*CDKN1A*/p21, *CDKN1C*/p57, *RBL2*/p130) and the cyclin D family were overexpressed, reflecting a normal phenotype. Tumor suppressors are crucial factors in tumorigenesis, often deleted or downregulated in tumors. Cyclins are often upregulated in cancers (32); however, cyclin D1 has not been shown to be a negative prognostic factor in cancer (32), cyclin D2 is often methylated and thus downregulated in lung cancer (33), and cyclin D3 was suggested not to have a profound role in tumorigenesis (34). Opposite, in all the tumors, including mesothelioma, these genes were suppressed, while oncogenes, cyclins, cyclin dependent kinases, and other cell cycle driving genes were overexpressed (Figure 6). The SQ, SCLC but also mesothelioma displayed overexpressed Mini-Chromosome Maintenance complex genes, while not differentially expressed in AD and downregulated in COID. In the SQ, 56 of 63 genes in the cell cycle were overexpressed, including *CCNE/CDK2*, *CCNA*/*CDK2*, *CCNA*/*CDK1*, *CCNB*/*CDK1* complexes, *BUB1*, *BUB1B*, *BUB3*, and *MYC* oncogenes, the damage response genes as *ATR*, *CHEK1/2*, and five genes of the 14-3-3 family, where several have been linked to tumorigenesis including the hypomethylated SFN or Stratifin (35). Again, some of these findings are not surprising, but recapitulate prior knowledge. Moreover, the *TGFB1I1* was overexpressed in normal lung but less so in tumors, as seen in the Human Protein Atlas (36). Recently, a gene-expression study on aggressive versus more indolent lung COID showed that several of these cell cycle genes, including *BUB1*, were overexpressed in the aggressive forms (37).

#### Thymidylate synthase

The one carbon pool by folate pathway is the target of several antineoplastic treatments, where thymidylate synthase (TS) is a key enzyme. Pemetrexed is a multifolate antagonist that inhibits replication through inhibition of folate-dependent enzymes as TS, GARFT, and DHFR, where TS is the main target with many orders of magnitude higher affinity for pemetrexed (38). The TS encoded by TYMS, is a key protein that catalyzes the methylation of deoxyuridylate (dUMP) to deoxythymidylate (dTMP) that maintains the dTMP pool critical for DNA replication and repair and has shown a strong correlation to the effect of pemetrexed. Low TS expression increases the pemetrexed response *in vitro* (39, 40) and a recent meta-analysis on clinical data showed that both TYMS and TS expression were inversely correlated to the effect of pemetrexed in lung cancer (41). This is in line with our findings were TYMS was not overexpressed in AD where pemetrexed has a main role in treatment, but significantly overexpressed in SCLC and SQ were it has no proven effect, and is not used in the clinic (42–44). Interestingly, while in the normal tissue, no genes in this one carbon pool by folate pathway were overexpressed, several genes were upregulated in the cancers (Figure S1 in Supplementary Material). The overexpression of TYMS in our mesothelioma cohort, where pemetrexed is a key treatment, was probably due to the fact that most of the mesothelioma cases included were generally resistant to pemetrexed, and had overexpressed TS (45, 46). In the COID, the TYMS was not overexpressed, indicating that COID could be a potential tumor group for multifolate inhibitors. To our knowledge, this has not been tested yet.

#### ERBB signaling pathway

The genes of the epidermal growth factor receptor (ERBB) signaling pathway were differentially expressed in each tissue type, including the *ERBB2* (*HER2*), *ERBB3* (*HER3*), and *ERBB4*. These are type I transmembrane growth factor receptors that activate intracellular signaling pathways in response to extracellular signals and activate numerous downstream pathways involved in the regulation of differentiation, migration, proliferation, and survival (47). Interestingly, *EGFR* (*HER1*), encoding EGFR, the primary target of the tyrosine kinases gefitinib and erlotinib, was not overexpressed in any type. This may have a simple explanation, either that only a minority has activating mutations of EGFR (17 and 2% of the non-squamous cancers in Caucasian and Afro-American population, respectively), and thus did not show a statistical difference as a group, or the array did not detect mutant EGFR (48). Currently, the HER2 receptor is a very important drug target in HER2 overexpressing breast and gastric cancers. It is known that the HER2 protein is overexpressed in 20% of lung cancer cases, but treatment with trastuzumab in this group has not been beneficial except probably in 1–2% of cases with a *HER2* gene driver mutation (49). More surprising was the finding that *HER3* was overexpressed in AD. Recently afatinib, a selective, orally bioavailable ERBB family blocker that irreversibly blocks signaling from EGFR, HER2, and ERBB4 showed an increased progression-free survival of *EGFR* mutated ADs of the lung in a phase III study versus chemotherapy (50). The *ERBB4* was overexpressed in the COID, recapitulating immunohistochemistry showing that 100% of lung COID expresses *ERBB4* (51) and thus indicating a potential novel candidate target for COID (Figure S1 in Supplementary Material).

#### DNA repair

**Figure 7:**
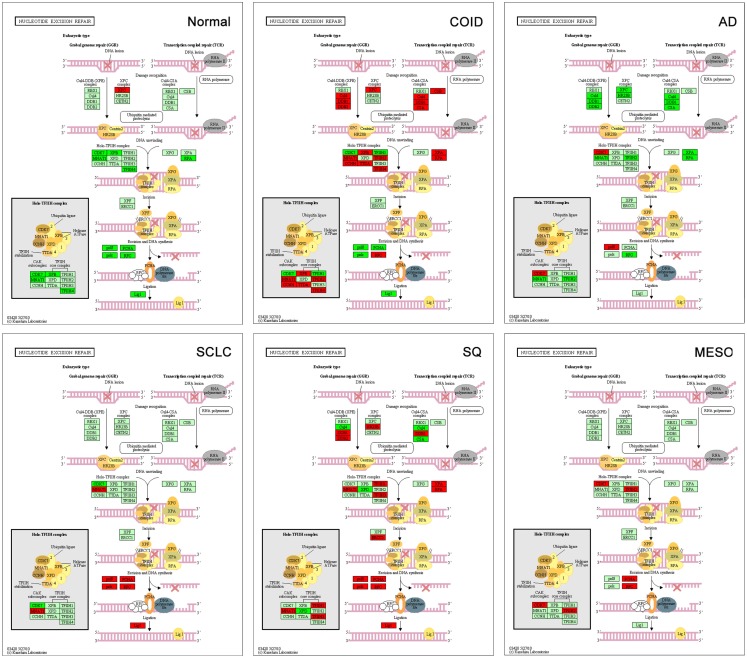
**Nucleotide excision repair (NER) pathway**. Genes belonging to all the principal DNA repair systems were predominantly overexpressed in tumors. The NER is the most analyzed pathway related to cisplatin resistance. In normal lung tissue (Normal), only one DNA repair gene was overexpressed, the XPC. In the COID, SCLC, SQ, and MESO tumors, an array of genes was overexpressed. Key genes of the whole repair process were overexpressed. In the SQ, 25 NER genes were differentially expressed, and only four were downregulated, the CUL4, XPD, ERCC2, and ERCC8. The most heterogeneous tumor group, AD, had the least NER genes overexpressed. Red indicates significantly overexpression, dark green significant downregulation, and light green no differential expression. COID, carcinoids; AD, adenocarcinoma; SCLC, small-cell lung cancer; SQ, Squamous cell lung cancer; MESO, mesothelioma.

The backbone of cytotoxic treatment of non-EGFR mutated lung cancers as well as mesothelioma includes platinum derivatives cisplatin and carboplatin. They are often designated cross-linking agents due to their capacity of inducing lethal DNA damage in cells through DNA inter- and intra-strand crosslinks. However, the response rates in the first line treatment of advanced cases are approximately 35%, meaning that the intrinsic resistance is high (52). Resistance to the cross-linking agents requires a functioning DNA repair system, especially the pathways that take care of crosslinks and adducts. In that respect, this analysis showed a marked difference between tumors and normal lung tissue. Tumors showed overrepresentation and overexpression of genes of all the DNA repair pathways, including the NER, BER, HR, MMR, and NHEJ, as well as the Fanconi anemia, all known to play a role in platinum resistance, while in normal lung only one DNA repair gene was overexpressed, the *XPC* in NER (Figure 7) (7, 53). Importantly, the tumor types with the highest intrinsic resistance to platinum, the COID and SQ also had the highest number of repair genes overexpressed (Table 4). The NER pathway is the principal and most analyzed pathway related to cisplatin resistance, and here, there was an array of significantly overexpressed NER genes in the COID, SCLC, SQ, and MESO tumors. Interestingly, the most heterogeneous tumor group, both genetically and regarding treatment response, the AD, had the least NER genes overexpressed (Figure 7). In the SQ, 25 NER genes were differentially expressed, and only four were downregulated, the *CUL4*, *XPD*, *ERCC2*, and *ERCC8*. Key genes of the whole NER repair process through damage recognition, DNA unwinding, incision, excision, DNA synthesis, and ligation were overexpressed (Figure 7). There are also several similarities with mesothelioma in this respect where overexpression of two or more of *CDK7*, *GTF2H2*, *PCNA*, *RFC4*, and the *RFC5* were shared by the lung tumors (Table 4). Importantly, a recent study showed that DNA repair proteins of the NER pathway can predict effect of adjuvant cisplatin in SQ but not in AD, showing the relevance of more knowledge related to these pathways (54).

**Table 4:** **Differential expression for each tumor type versus rest in KEGG pathways related to DNA repair**.

Genes	NER	BER	NHEJ	HR	MMR
**NOR**	***XPC***	*None*	*None*	*None*	*None*
**COID**	*CCNH*, *CUL4B*, *DDB1*, *DDB2*, *ERCC3*, *ERCC6*, *ERCC8*, ***GTF2H2***, *GTF2H4*, *MNAT1*, *RFC1*, *RFC2*, ***RFC4***, *RPA2*, *XPA*, ***XPC***	*LIG3*, *MUTYH*, *OGG1*, *XRCC1*	*DNL4*, *LIG4*, ***RAD50***, *XRCC6 (KU70)*	*RAD51C*, *RAD51D*, *XRCC2*	*MLH1*, *MLH3*, *MSH3*, *RFC1*, *RFC2*, ***RFC4***, *RPA2*
**AD**	***CDK7***, ***POLD4***	*MPG*, *NTHL1*, ***POLD4***, *PARP3*, *PARP4*	*MRE11A*, ***XRCC4***, *XRCC6 (KU70)*	*MRE11A*, ***POLD4***	***POLD4***
**SCLC**	***LIG1***, *MNAT1*, ***PCNA***, *POLD3*, ***POLD4***, *POLE*, *POLE2*, *POLE3*, *RFC3*, ***RFC4***, ***RFC5***	***FEN1***, ***LIG1***, *MUTYH*, *PARP1*, *PARP2*, *PARP3*, ***PCNA***, *POLD3*, *POLD4*, *POLE*, *POLE2TDG*, *UNG*	***FEN1***	*BLM*, ***BRCA2***, *POLD3*, ***POLD4***, ***RAD54L***, *RAD51C*	*EXO1*, ***LIG1***, *MSH2*, ***MSH6***, *POLD3*, *POLD4*, ***PCNA***, *RFC3*, ***RFC4***, ***RFC5***
**SQ**	*DDB1*, *DDB2*, *ERCC1*, *GTF2H1*, *GTF2H3*, ***LIG1***, *MNAT1*, ***PCNA***, *POLD2*, ***POLD4***, *POLE*, *POLE2*, *POLE3*, *RAD23A*, *RAD23B*, *RFC2*, *RFC3*, ***RFC4***, ***RFC5***, *RPA1*, *RPA2*, *XPA*	*APEX2*, ***FEN1***, *HMBG1*, ***LIG1***, *MBD4*, ***PCNA***, *POLD2*, ***POLD4***, *POLE*, *POLE2*, *POLE3*, *POLB*, *PARP1*, *PARP2*, *PARP3*, *TDG*, *UNG*	*LIG4*, ***FEN1***, *PRKDC*, *XRCC5 (KU80)*	*BLM*, ***BRCA2***, *POLD2*, *POLD4*, *RPA1*, *RPA2*, *RAD51C*, *RAD51D*, ***RAD54L***, ***SHFM1***	***LIG1***, *MSH2*, ***MSH6***, *POLD2*, *POLD4*, *PCNA*, *RFC2*, *RFC3*, ***RFC4***, ***RFC5***, *RPA1*, *RPA2*
**MESO**	***CDK7***, ***GTF2H2***, ***PCNA***, ***RFC4***, ***RFC5***	***FEN1***, ***PCNA***	***FEN1***, ***RAD50***, ***XRCC4***	***BRCA2***, *DSS1*, *EME1*, *RAD50*, ***SHFM1***, ***RAD54L***, *SSBP1*	***MSH6***, ***PCNA***, ***RFC4***, ***RFC5***, *SSBP1*

*For each pathway, only the overexpressed genes are shown. Genes that are common between several lung cancer subtypes are in bold. The mesothelioma results are based on mesothelioma versus parietal pleura (see Materials and Methods)*.

*BER, base excision repair; HR, homologous recombination; MMR, mismatch repair; NER, nucleotide excision repair; NHEJ, non-homologous end joining; NOR, normal lung tissue; COID, carcinoids; AD, adenocarcinoma; SCLC, small-cell lung cancer; SQ, squamous cell lung cancer; MESO, mesothelioma*.

**Figure 8:**
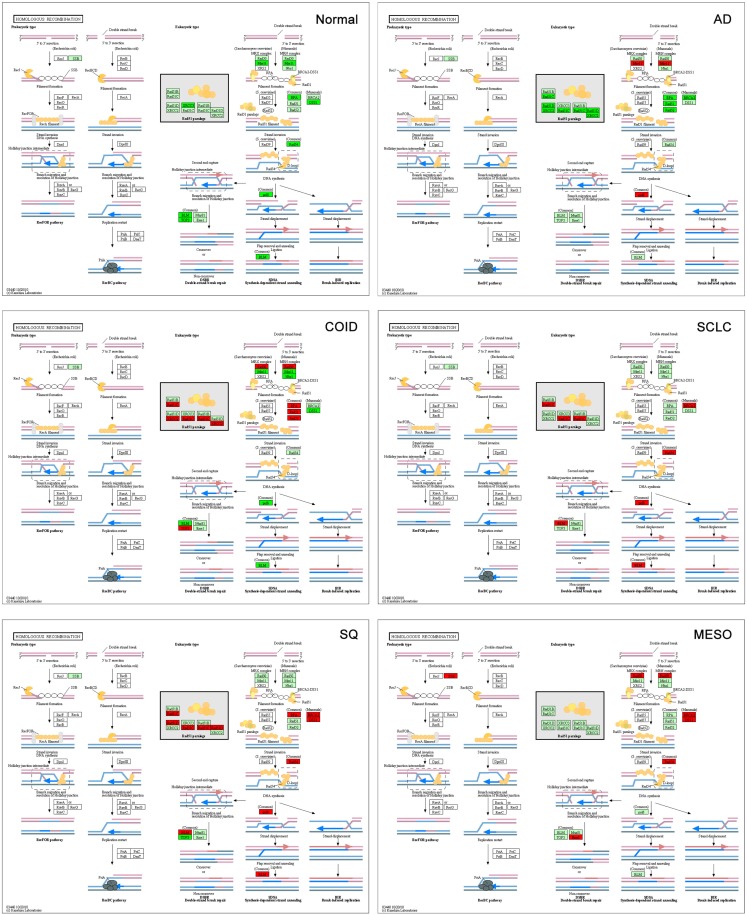
**Homologous recombination (HR) pathway**. The HR is the principal, non-error-prone, repair mechanism for DNA double-strand breaks. SQ and SCLC had 10 of 11 overexpressed genes throughout the pathway, including the very important BRCA2, RAD51 paralogs, and BLM (Bloom syndrome mutated). Red indicates significantly overexpression, dark green significant downregulation, and light green no differential expression. COID, carcinoids; AD, adenocarcinoma; SCLC, small-cell lung cancer; SQ, Squamous cell lung cancer; MESO, mesothelioma.

Other important platinum resistance repair pathways were highly overexpressed, including the HR pathway. The HR is the principal DNA double-strand break (DSB), non-error-prone repair mechanism that takes place in the late S-G2 phase of the cell cycle and involves generation of a single-stranded region of DNA, strand invasion, formation of a Holliday junction, DNA synthesis using the intact strand as a template, branch migration, and resolution. Our results showed that SQ and SCLC had overexpressed genes throughout the pathway with 10 of 11 genes overexpressed including the very important *BRCA2*, *RAD51* paralogs and BLM (Bloom syndrome mutated) (Figure 8) (55). In normal lung tissue, none of the HR genes were overexpressed.

One indispensable part of cisplatin induced DNA inter-strand crosslink repair is the Fanconia anemia pathway (56, 57). The crosslinks are recognized by *FANCM* and associated proteins, recruiting the FA core complex. The *FANCD2* and *FANCI* are monoubiquitinated by the FA core complex. The monoubiquitinated *FANCD2*/*FANCI* becomes an active form and interacts with a series of DNA repair proteins and facilitates downstream repair pathways. In the normal tissue, all *FANC* genes were downregulated, while in all tumors several were overexpressed (File S1 in Supplementary Material).

In SCLC, the standard treatment consists of etoposide combined with cisplatin, which gives a high response rate but an almost 100% recurrence rate. Etoposide is a DNA topoisomerase II poison that prevents the re-ligation of topoisomerase II-induced single-strand breaks. However, the DNA repair profile was very similar to the SQ, so that other genes also interfere both for the higher response, and the high relapse rate. A recent study on deciphering the resistance genes of etoposide, the polymerase beta (*POLB*), and the homolog of *TTF1*, *NKX2-2* were identified. Overexpression of *NKX2-2* has been shown to predict a more dismal survival (58). In our data, *NKX2-2* was overexpressed only in the SCLC with a diagnostic AUC of 0.85 (Table 3).

### Primary versus metastatic adenocarcinoma

The distribution of the gene expressions of metastatic AD from colon (*n* = 8), breast cancer (*n* = 2), and unspecified (*n* = 3) was in the same range as the primary lung AD (Figure 4), so the overall expression profiles were similar. However, differential expression of 267 genes was detected in metastatic versus primary tumors. The gene with highest discriminatory power was the *SLC26A3*, synonymous to downregulated in adenoma (DRA) that was overexpressed in the metastatic AD. This gene encodes a transmembrane glycoprotein that transports chloride ions across the cell membrane in exchange for bicarbonate ions and is specifically localized to the normal mucosa of the lower intestinal tract. Downregulation of this gene was not only inversely correlated to adenomas and ADs of the colon but also to the clinical stage of the tumors (59, 60). However, this gene/protein is not expressed in normal lung, or in lung cancer (36), so this finding probably reflected the relative overweight of colon cancer metastases in this material, and how such an analysis could identify putative markers for colon cancer metastasis to the lung.

### Normal lung tissue

There are few published accounts of the gene profile of normal lung tissue, as it usually is used as a control to describe tumor tissue. However, in this analysis, we have also tried to describe the normal tissue as related to the tumors. Actually the observed, almost non-existent overexpression, and even downregulation of DNA repair systems pathways was a novel finding (Figure S2 in Supplementary Material). That does not mean that the normal tissue do not have DNA repair, only that the levels are much lower in relation to the cancers. In this respect, the finding that some other crucial mechanisms for cell maintenance and survival were overexpressed in normal tissue while being downregulated or indifferent in the tumors, was surprising. Below, we will discuss some of the pathways that clearly were most upregulated in the normal tissue and downregulated in the cancers.

#### Vascular smooth muscle contraction

**Figure 9:**
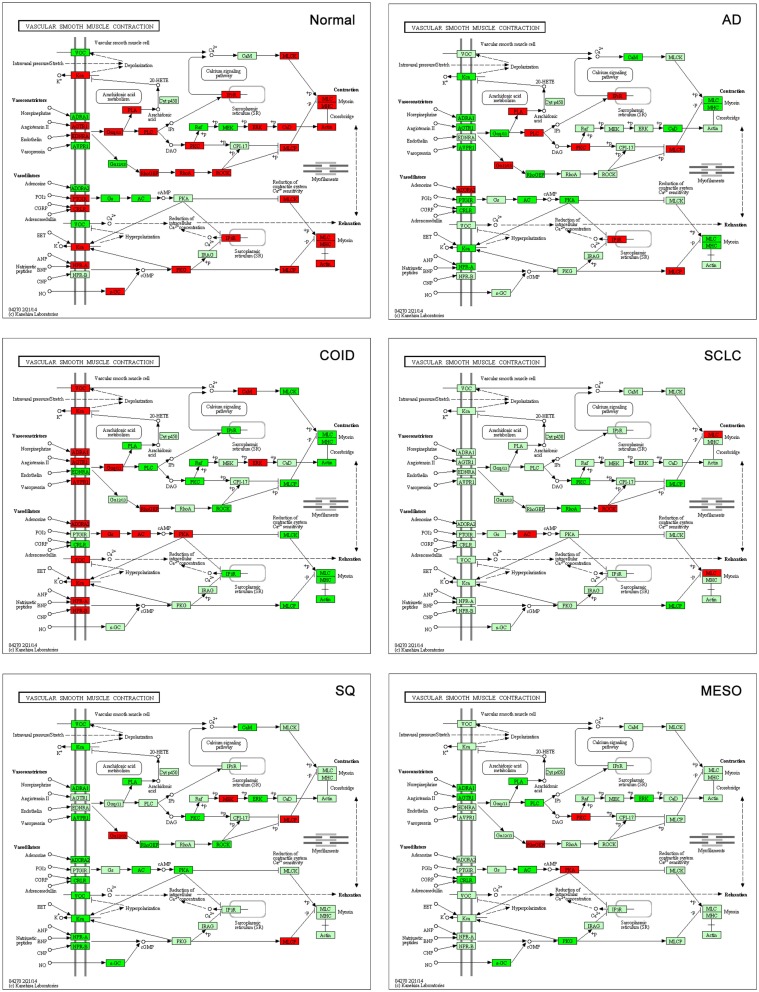
**Vascular smooth muscle contraction pathway**. Genes belonging to the vascular smooth muscle contraction pathway were significantly overrepresented and upregulated in normal tissue (15/36 genes, *p* < 0.0001). None of the genes of the smooth muscle cell membrane (according to KEGG pathways) were overexpressed in SQ, SCLC, and mesothelioma. Red indicates significantly overexpression, dark green significant downregulation, and light green no differential expression. COID, carcinoids; AD, adenocarcinoma; SCLC, small- cell lung cancer; SQ, squamous cell lung cancer; MESO, mesothelioma.

Contraction of vasculature is important in controlling the blood flow and influx of oxygen and nutrients to tissues. Vascular smooth muscle contraction pathway genes were significantly overrepresented and upregulated in normal tissue (15/36 genes, *p* < 0.0001, File S4 in Supplementary Material) opposed to SQ, SCLC, and mesothelioma, were none of the genes of the smooth muscle cell membrane were overexpressed in the cancers (Figure 9). Recently, a bioinformatics study on gene expression of lung cancer of unspecified type versus normal showed that vascular smooth muscle contraction pathway was negatively regulated in tumor, in line with our findings (61). The significantly overexpressed genes in the normal lung included potassium channel genes (*KCNMB1*), the vaso-constricting angiotensin receptor II genes (*AGTR1*), endothelin receptor type A (*EDNRA*), and vasodilating genes as the adenosine (*ADORA2B*), calcitonin (*CRLCR*, *RAMP1*, *RAMP2*, and *RAMP3*), and natriuretic peptide receptor (*NPR1*). How can this be explained? Normal vasculature needs a well-functioning vasoconstrictor and dilator system, while some of these functions may be defective in pathological tumor vessels formed by neo-angiogenesis (62). Tumors often have a higher microvessel density than normal tissues; however, these pathological blood vessels are often described as less elastic and with different physiology than normal vessels. As an example, the endothelium of the microvessels has been incriminated in promoting tumor growth by autocrine loops (63).

An intriguing finding was the overexpression of the *ILK* gene (integrin-linked kinase) in normal lung (*p* = 3.71 × 10^−7^) and its downregulation in SQ (*p* = 0.0037). Loss of this integrin-linked kinase induces failure in the formation of a unitary layer in ILK-deficient vascular smooth muscle cells and also induces abnormal contractility, through the activation of *RHOA*/*ROCK2* that was also overexpressed in normal lung (64). In the literature, we were not able to identify accounts of this gene profile in lung tumors and normal lung tissue.

#### Leukocyte trans-endothelial migration

Leukocyte trans-endothelial migration pathway genes were highly overrepresented and upregulated in normal tissue versus the rest (16/35 genes, *p* < 0.0001, File S4 in Supplementary Material), and thus downregulated in the tumors. The migration of cells through the vasculature is an important mechanism that is complex, active, and requires the presence and activity of several genes. This mechanism is very important for the flux of neutrophils in case of inflammation but also lymphocytes for cytotoxic T-lymphocytes and killer T-cells. Leukocyte trans-endothelial migration downregulation was found to correlate with recurrence after operation of stage I lung cancer in four large datasets, and thus seem to play a role in tumor aggressiveness (65). We have previously shown that this pathway was downregulated in mesothelioma versus normal parietal pleura and that it was even more downregulated after acquired pemetrexed and platinum resistance (66). Here, we show that it is a consistent trait in all the lung tumors (Figure S3 in Supplementary Material). Among the consistently downregulated genes in tumors were the *PECAM1*, *JAM2*, and *CDH5*. The *PECAM1*/*CD31* (platelet/endothelial cell adhesion molecule) is a member of the immunoglobulin superfamily that is expressed on the surface of platelets, monocytes, neutrophils, and T-cell subsets and is also a major constituent of the endothelial cell intercellular junction. It modulates multiple functions besides trans-endothelial migration, integrin-mediated cell adhesion, angiogenesis, apoptosis, cell migration, and negative regulation of immune cell signaling (67). Inhibition of the *JAM2*/*JAMB* (junctional adhesion molecule) decreases leukocyte infiltration (68). Vascular endothelial cadherin or CDH5 is a key protein controlling the endothelial barrier and its disruption by specific antibody both amplifies metastasis in normal mice and overcomes the genetic resistance in mice (69). Moreover, CDH5 is a candidate tumor suppressor and low expression strongly correlated to decreased survival in neuroblastoma (70). One is tempted to speculate that this is a trait of aggressive and treatment resistant cancers as well, where the antitumor immune system cannot reach the tumor cell due to hindering the entrance of immunological cells in tumor resulting in a barrier to immunologic tumor rejection and thus to immunological therapies. Interestingly, this coincides with results from an immunotherapy trial showing good responses in mesothelioma, but only in small tumors (71).

#### Actin cytoskeleton

Regulation of actin cytoskeleton genes was the top overrepresented and upregulated pathway in normal tissue versus the rest (29/58 genes, *p* < 0.0001, File S4 in Supplementary Material) (Figure S3 in Supplementary Material). Actin cytoskeleton is larger than any organelle, and is comprised of actin filaments and microtubules, that make cell locomotion and cell division possible, as well as keeping cell polarity and morphology. Within this pathway some genes were uniquely overexpressed in normal tissue including *IQGAP1* and *IQGAP2* (IQ motif containing GTPase activating protein). These are members of a family of scaffold proteins where most studied *IQGAP1* modulates several cellular functions, including cell–cell adhesion, transcription, cytoskeletal architecture, migration, and selected signaling pathways, reviewed in Ref. (72). Overexpression of this gene has been associated to several cancer types, in contrast to our findings, where it was downregulated or indifferent in the tumors. However, *IQGAP2* overexpression is protective against tumorigenesis, as the loss or downregulation of *IQGAP2* has been shown to play a role in development of hepatocellular, gastric, and prostate cancer (73). Another uniquely overexpressed gene in normal lung tissue was the *RHOA* (ras homolog family member A). The *RHOA* is involved in a multitude of processes as controlling epithelial cell junctions as well as regulating microtubule function and cytokinesis (74). It stimulates the formation of active non-muscle myosin filaments and long, unbranched F-actin, and is crucial for cell polarity, of which is disturbed in epithelial–mesenchymal transition (*EMT*) during metastasis. Cytokinesis dysregulation also leads to the formation of polyploid and aneuploid cells that are prone to tumor formation. Recently, it was shown that the increased degradation of *RHOA* encoded protein RhoA by altered autophagy induced pathological cytokinesis resulting in multinucleation and aneuploidy (75). In the Human Protein Atlas, the RhoA is expressed in all normal respiratory tissues while only in 34% of lung cancers, supporting our findings (36).

### Genes correlated to tumor size but not to survival

The original dataset provided survival data only for the AD patients, and all were treated surgically. Only type of operation performed and TNM stage was shown, but no information on adjuvant or systemic treatment in general. Eligible for survival analysis were 31 cases in Stage IA, 40 patients with Stage IB, four with Stage IIA, 20 with Stage IIB, 7 with Stage IIIA, 3 with Stage IIIB, 3 with Stage IV, in total 108 cases. In the original paper, an AD subgroup called C2 or neuroendocrine expression type, was claimed to have a less favorable survival compared to the rest of the AD and that one gene, kallikrein 11 (*KLK11*) could separate this group from the rest of the tumors (6). However, the C2 cases were only nine, making valid conclusions on survival difficult, and the kallikrein 11 was upregulated in the other groups as well (original publication, Figure 4). In our analysis of only the primary lung AD (Figure 1), no single gene or gene signature could predict survival. This is not surprising as the survival is an endpoint influenced by many factors, and this population had a very heterogenous stage spectrum, probably heterogenous systemic treatment that was not reported and gene-expression heterogeneity as we reported. Probably, a clinically more homogenous group would provide a better basis for discovery of prognostic and predictive signatures.

There was, however, significant correlation of four genes with tumor size, where one gene, *NR2C1* (nuclear receptor subfamily 2, group C, member 1), showed a positive correlation that is increased expression with larger tumor (Table 2). Interestingly, this not very well-characterized gene seems to play an important role in early embryonic development by regulating key genes involved in stem cell self-renewal, commitment, and differentiation (76). Finally, there were two genes significantly downregulated in N0 versus N1–3 cases. One was the *FLNB*, one of the mammalian filamins, large actin-binding protein that is important for migration of cells (77).

### Novel bioinformatics applications versus the methods used in the original paper

Several groundbreaking advancements have been introduced in the field of bioinformatics in the 13 years that separate the Bhattacharjee study and this analysis. Some of the statistical methods used in this work, for example, quantile normalization (78), were either not available or not widely known at the time of the first study, while they are nowadays widely accepted and used.

Importantly, the last 10 years have witnessed a dramatic effort by the bioinformatics community for creating and maintaining comprehensive on-line resources able to store in a structured way the massive mole of biological knowledge that is steadily generated. The KEGG pathways database (79), the Gene Expression Omnibus (80), and the REACTOME database (81, 82) are just a few examples of such resources.

Particularly, most of the conclusions drawn in the present work were obtained by integrating data-driven results (set of differentially expressed genes) with the pathway-oriented information provided by the KEGG and GO database. Such type of study would have been severely limited back in 2001, when the information stored in these repositories was much scarcer.

### Study drawbacks

The main weakness of this study is that it is based on transcriptome data obtained in the early days of microarray technology, when mRNA extraction procedure and microarray technical solution were not as mature, optimized, and reliable as today. As a first consequence, the number of gene-expression levels measured in the original study was inferior to the number of genes that can now be quantified. There was no other dataset analyzing identical subgroups, so a direct comparison with our study could not be performed. However, the results are aligned with other studies in several respects indirectly showing the high quality of the data, and comparative analysis performed on two separate datasets showed a high concordance of differentially expressed genes, ranked gene lists and AUC (see Text S1 in Supplementary Material). Another problem is the scarcity of SCLC cases, which can be attributed to the fact that they are seldom operated and fine needle cytology often gain too few cells for analysis. Ideally, large cell or other rare subgroups of NSCLC should have been analyzed, but these were not included in this dataset. Based on our findings, we would recommend to use old microarray datasets for novel data mining, given that the study design and the sample treatment are of provably adequate quality.

## Conclusion

The lung cancer gene-expression profiles provided through this analysis, recapitulated some of the known features of classical histological subtypes, indicated novel candidate diagnostic markers and outlined new information on differences between normal lung tissue and tumor. The overrepresentation and overexpression of DNA repair genes was remarkably consistent in all tumors, and recapitulated known treatment resistance patterns. Novel interesting features of upregulated pathways in normal lung may shed some light on the biology of the normal lung as well as in tumors, deserving further study. This study provides an example of how the integrative *in silico* analysis of “old data” in conjunction with novel biological knowledge and computational techniques can provide much information not deducible at the time when the data were produced.

## Author Contributions

Conceived and designed the experiments: Oluf Dimitri Røe, Ioannis Tsamardinos, Vincenzo Lagani, and Konstantinos Kerkentzes. Performed the experiments: Konstantinos Kerkentzes, Vincenzo Lagani, and Ioannis Tsamardinos. Analyzed the data: Oluf Dimitri Røe, Ioannis Tsamardinos, Vincenzo Lagani, and Konstantinos Kerkentzes. Contributed reagents/materials/analysis tools: Ioannis Tsamardinos, Mogens Vyberg, and Oluf Dimitri Røe. Wrote the paper: Konstantinos Kerkentzes, Oluf Dimitri Røe, Ioannis Tsamardinos, Vincenzo Lagani, and Mogens Vyberg.

## Conflict of Interest Statement

The authors declare that the research was conducted in the absence of any commercial or financial relationships that could be construed as a potential conflict of interest.

## Supplementary Material

The Supplementary Material for this article can be found online at http://www.frontiersin.org/Journal/10.3389/fonc.2014.00251/abstract

Figure S1 **Clinically relevant differentially expressed pathways in each tissue type versus the rest**. The pathway maps are arranged according to the scheme in the left-top corner. Red indicates overexpression in genes, dark green downregulation. In the Cell Cycle pathway, tumor suppressor genes were overexpressed in normal lung tissue while oncogenes and tumor driving cyclins were overexpressed in cancers. The gene thymidylate synthase (TYMS or TS, belonging to the pathway “one carbon pool by folate”) is relevant for tumor growth and is also a treatment target. Notably, TYMS was overexpressed only in the tumors that are generally refractory to the drug pemetrexed, as the squamous and the small-cell lung cancer. TYMS was not overexpressed in mesothelioma, but it is known that TYMS expression is highly variable in this cancer. In the ERBB pathway, the ERBB2/HER2 and ERBB3/HER3 were overexpressed in adenocarcinoma, while the ERBB4 was overexpressed in the carcinoids.

Figure S2 **Genes in overrepresented DNA repair pathways are predominantly overexpressed in cancer**. The figure reports the base excision repair (BER), homologous recombination (HR), and mismatch repair (MMR) pathways. Red indicates significantly overexpressed genes, dark green significantly downregulated, and light green not differentially expressed.

Figure S3 **Pathways predominantly overexpressed in the normal lung tissue**. The regulation of KEGG pathways actin cytoskeleton, leukocyte trans-endothelial migration, and smooth muscle vasculature contraction. Red indicates significantly overexpressed genes, dark green significantly downregulated, and light green not differentially expressed.

Text S1 **Detailed report of the validation procedure and results**.

File S1 **Differentially expressed genes lists**. A zip file containing the differentially expressed genes of each histological type versus the rest and of the metastatic versus the primary adenocarcinomas.

File S2 **AUC lists**. A zip file containing the AUC of the differentially expressed genes of each histological type versus the rest and of the metastatic versus the primary adenocarcinomas.

File S3 **GO analysis**. A zip file containing the enriched GO biological processes resulting from the hyper-geometric test for each histological type versus the rest.

File S4 **KEGG analysis**. A zip file containing the enriched KEGG pathways processes resulting from the hyper-geometric test for each histological type versus the rest.
