# Supplementary material for: Hidden Treasures in “Ancient” Microarrays: Gene-Expression Portrays Biology and Potential Resistance Pathways of Major Lung Cancer Subtypes and Normal Tissue
Source: Front Oncol. 2014 Sep 29;4:251. doi: 10.3389/fonc.2014.00251 (PMC4178426; doi:10.3389/fonc.2014.00251)
Supplement: Figure S1 — Clinically relevant differentially expressed pathways in each tissue type versus the rest. The pathway maps are arranged according to the scheme in the left-top corner. Red indicates overexpression in genes, dark green downregulation. In the Cell Cycle pathway, tumor suppressor genes were overexpressed in normal lung tissue while oncogenes and tumor driving cyclins were overexpressed in cancers. The gene thymidylate synthase (TYMS or TS, belonging to the pathway “one carbon pool by folate”) is relevant for tumor growth and is also a treatment target. Notably, TYMS was overexpressed only in the tumors that are generally refractory to the drug pemetrexed, as the squamous and the small-cell lung cancer. TYMS was not overexpressed in mesothelioma, but it is known that TYMS expression is highly variable in this cancer. In the ERBB pathway, the ERBB2/HER2 and ERBB3/HER3 were overexpressed in adenocarcinoma, while the ERBB4 was overexpressed in the carcinoids. [file Data_Sheet_1.ZIP › Text S1.DOCX]

Supplementary Text S1

# Validation on external datasets

In order to validate the robustness of our methods, we employed two more datasets to explore whether our approach can provide consistent findings in other lung cancer microarray data. This is a detailed report of our validation procedure.

# Datasets

The datasets that will be used in this validation analysis are the study we have focused on, Bhattacharjee et al. from 2001 [[1](#_ENREF_1)] referred to as Bhatt., Yap et al. published in 2005 referred to as Yap [[2](#_ENREF_2)] and Hou et al. published 2010 [[3](#_ENREF_3)] referred to it as Hou. This dataset has 156 samples in total, 91 which belong to 3 cancer subtypes (adenocarcinoma, squamous and large cell) and 65 of normal tissue. We did not find any dataset with all the subtypes as in Bhatt.

*However, since only the lung adenocarcinomas and the normal samples were common in all three datasets, were used these two tissues for the results on each dataset to be comparable.* In Table 1 we present the number of samples of each dataset that were used for this validation.

| **Samples** | **Bhatt.** | **Yap** | **Hou** |
| --- | --- | --- | --- |
| Adenocarcinomas | 139 | 49 | 45 |
| Normal tissue | 17 | 9 | 65 |
| **Total** | 156 | 58 | 110 |
| *Table 1.* |  |  |  |

The reason that the Hou dataset has so many normal samples is that they took these samples from adjacent unaffected lung tissue while the normal samples of the other datasets were taken from different patients.

Moreover, the measurement of gene expression in each study was performed on different Affymetrix platforms with variable number of genes (Bhatt.: HGU-95Av2, Yap: HGU-133A, Hou: HGU-133plus2). Therefore, only the common genes (based on the EntrezID) were kept, which were 8556.

# Methods

On all the datasets we applied the same methods that we described in our paper. We calculated the RMA expression measure of the raw data (CEL files) using Bioconductor's “affy” package. Any probe with insufficient annotation was removed and when multiple probes were referring to the same gene, only the probe with the higher variation was retained. For differential expression, linear models and empirical Bayes statistics from Bioconductor's “limma” package were used and any gene with FDR adjusted p-value below 0.05 was considered significant. For identifying diagnostic genes, the AUC for each gene was determined using its expression as a ranking criterion, and the genes with high AUC were deemed as the most diagnostic. Principal components analysis (PCA) was performed on all the genes and either the first two (2D) principal components were plotted in order to visualize a significant portion of the information on the data.

## Expression of common genes

First of all, we present an overview of the results of the expression of common genes. We report the number of differentially expressed and non-differentially expressed genes of each dataset in Table 2 and the percentage of each category over the 8556 common genes in Table 3.

|  | **Bhatt.** | **Yap** | **Hou** |
| --- | --- | --- | --- |
| **Up Regulated** | 1710 | 1572 | 3147 |
| **Non-Diff. Expressed** | 5430 | 5315 | 2740 |
| **Down Regulated** | 1416 | 1669 | 2669 |
| **Total** | 8556 | 8556 | 8556 |
| *Table 2.* |  |  |  |

|  | **Bhatt.** | **Yap** | **Hou** |
| --- | --- | --- | --- |
| **Up Regulated** | 19.99% | 18.37% | 36.78% |
| **Non-Diff. Expressed** | 63.46% | 62.12% | 32.02% |
| **Down Regulated** | 16.55% | 19.51% | 31.19% |
| *Table 3.* |  |  |  |

From Tables 2 and 3, we see that the Bhatt. and Yap datasets have similar numbers of genes in each category compared to the Hou. This can be attributed partly to the different ratio of normal and adenocarcinoma samples in the Hou dataset (A: 40.91%, N: 59.09%) in comparison to the ratios in the other datasets (Bhatt. - A: 89.1%, N: 10.9%, Yap - A: 87.5%, N: 12.5%).

|  |  | Yap Dataset | | | |
| --- | --- | --- | --- | --- | --- |
| Bhatt. Dataset |  | Up regulated | Non-Diff. Expressed | Down Regulated | Row Total |
|  | Up Regulated | 735 | 890 | 85 | 1710 |
|  | Non-Diff. Expressed | 773 | 3831 | 826 | 5430 |
|  | Down Regulated | 64 | 594 | 758 | 1416 |
|  | Column Total | 1572 | 5315 | 1669 | 8556 |
|  | *Table 4.* |  |  |  |  |

|  |  | Hou Dataset | | | |
| --- | --- | --- | --- | --- | --- |
| Bhatt. Dataset |  | Up regulated | Non-Diff. Expressed | Down Regulated | Row Total |
|  | Up Regulated | 1324 | 306 | 80 | 1710 |
|  | Non-Diff. Expressed | 1791 | 2266 | 1373 | 5430 |
|  | Down Regulated | 32 | 168 | 1216 | 1416 |
|  | Column Total | 3147 | 2740 | 2669 | 8556 |
|  | *Table 5.* |  |  |  |  |

|  |  | Hou Dataset | | | |
| --- | --- | --- | --- | --- | --- |
|  |  | Up regulated | Non-Diff. Expressed | Down Regulated | Row Total |
| Yap Dataset | Up Regulated | 1089 | 350 | 133 | 1572 |
|  | Non-Diff. Expressed | 1902 | 1962 | 1451 | 5315 |
|  | Down Regulated | 156 | 428 | 1085 | 1669 |
|  | Column Total | 3147 | 2740 | 2669 | 8556 |
|  | *Table 6.* |  |  |  |  |

In Tables 4 to 6 we present the contingency tables between each pair of datasets. We see that in all these tables the number of genes that are reported to have contradicting expression between two datasets (being upregulated in one dataset while being downregulated in the other) are quite low and are almost at the same level for each pair. Furthermore, we notice that Bhatt.'s overlap with the other two datasets is nearly constant with respect to their gene sets and this is clearer in the column percentages in Table 7 and 8. Finally, although the Hou dataset has big differences in the expression of its genes compared to the other two datasets, we see that is more in concordance with the Bhatt. than with the Yap dataset (see the Tables 8 and 9).

|  |  | | Yap Dataset | | | |  |
| --- | --- | --- | --- | --- | --- | --- | --- |
| Bhatt. Dataset |  | | Up regulated | Non-Diff. Expressed | Down Regulated | | |
|  | Up Regulated | | 46.76% | 16.75% | 5.09% | | |
|  | Non-Diff. Expressed | | 49.17% | 72.08% | 49.49% | | |
|  | Down Regulated | | 4.07% | 11.18% | 45.42% | | |
|  | Column Total | | 100% | 100% | 100% | | |
|  | *Table 7.* | Column percentages of Table 4 | | | |  |  |

|  |  | | Hou Dataset | | | |  |
| --- | --- | --- | --- | --- | --- | --- | --- |
| Bhatt. Dataset |  | | Up regulated | Non-Diff. Expressed | Down Regulated | | |
|  | Up Regulated | | 42.07% | 11.17% | 3.00% | | |
|  | Non-Diff. Expressed | | 56.91% | 82.70% | 51.44% | | |
|  | Down Regulated | | 1.02% | 6.13% | 45.56% | | |
|  | Column Total | | 100% | 100% | 100% | | |
|  | *Table 8.* | Column percentages of Table 5 | | | |  |  |

|  |  | | Hou Dataset | | | |  |
| --- | --- | --- | --- | --- | --- | --- | --- |
|  |  | | Up regulated | Non-Diff. Expressed | Down Regulated | | |
| Yap Dataset | Up Regulated | | 34.60% | 12.77% | 4.98% | | |
|  | Non-Diff. Expressed | | 60.44% | 71.61% | 54.36% | | |
|  | Down Regulated | | 4.96% | 15.62% | 40.65% | | |
|  | Column Total | | 100% | 100% | 100% | | |
|  | *Table 9.* | Column percentages of Table 6 | | | |  |  |

In Figure 1 and 2 we present, respectively, the number of overlapping up and down regulated genes in all three datasets. The numbers in each figure sum up to 8556.


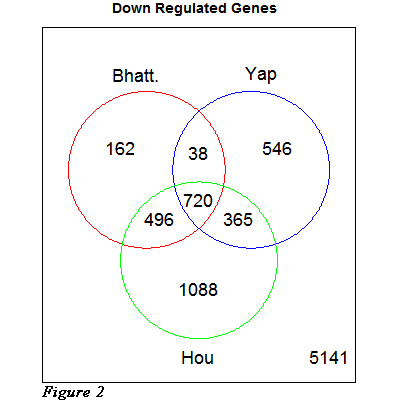


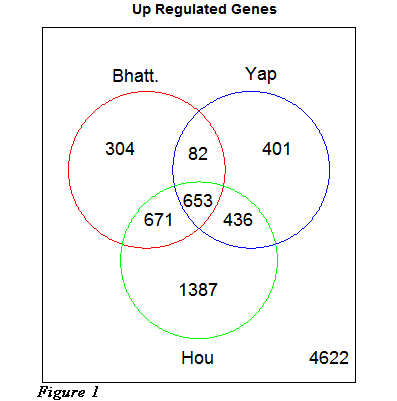


All these results indicate a substantial overlap of the expression of the genes in all datasets.

### PCA plots

Below, we present the PCA plots of the first two principal components for each dataset. We see that in all datasets the two groups are quite separable, and that the adenocarcinoma samples show a great heterogeneity.


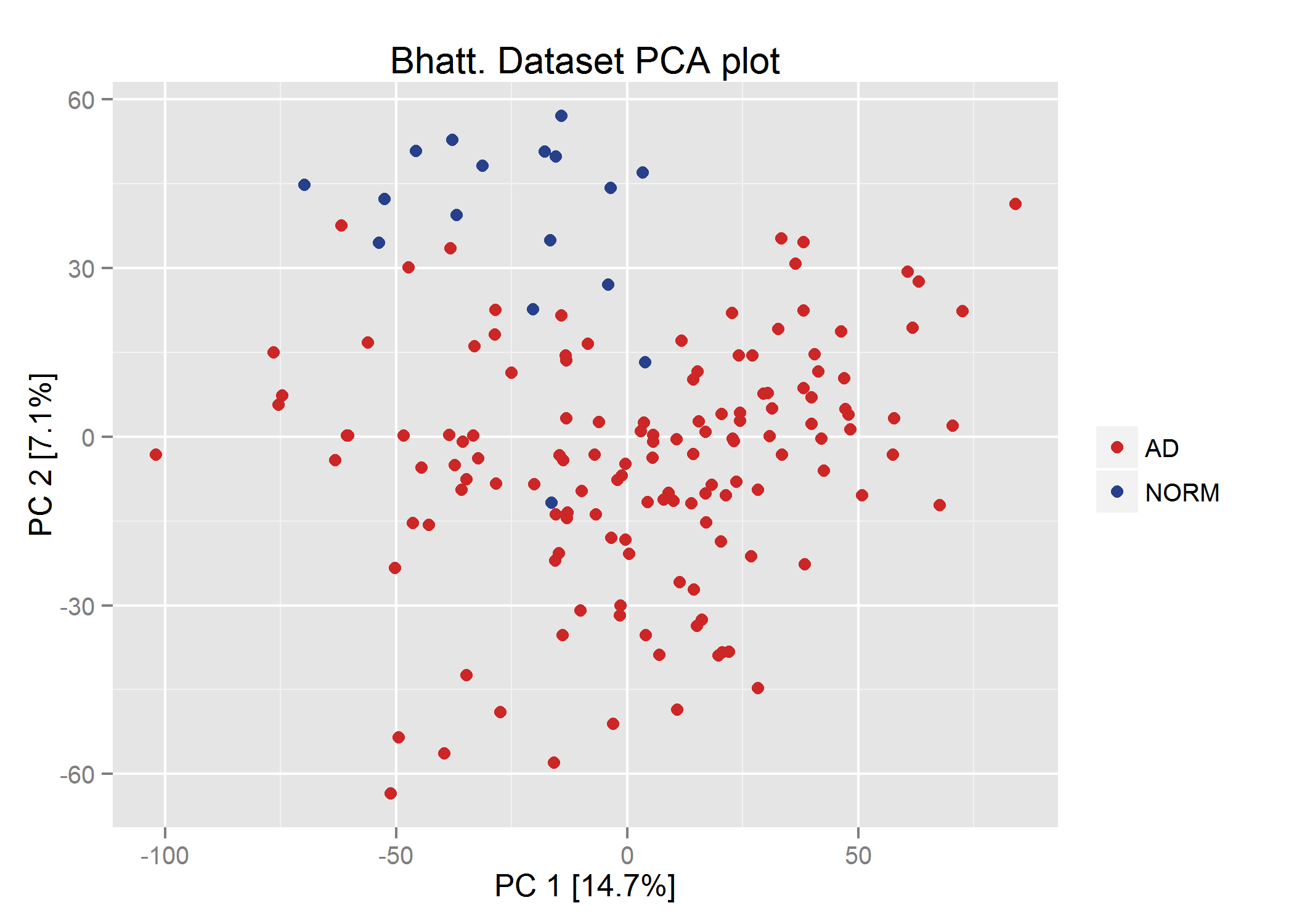


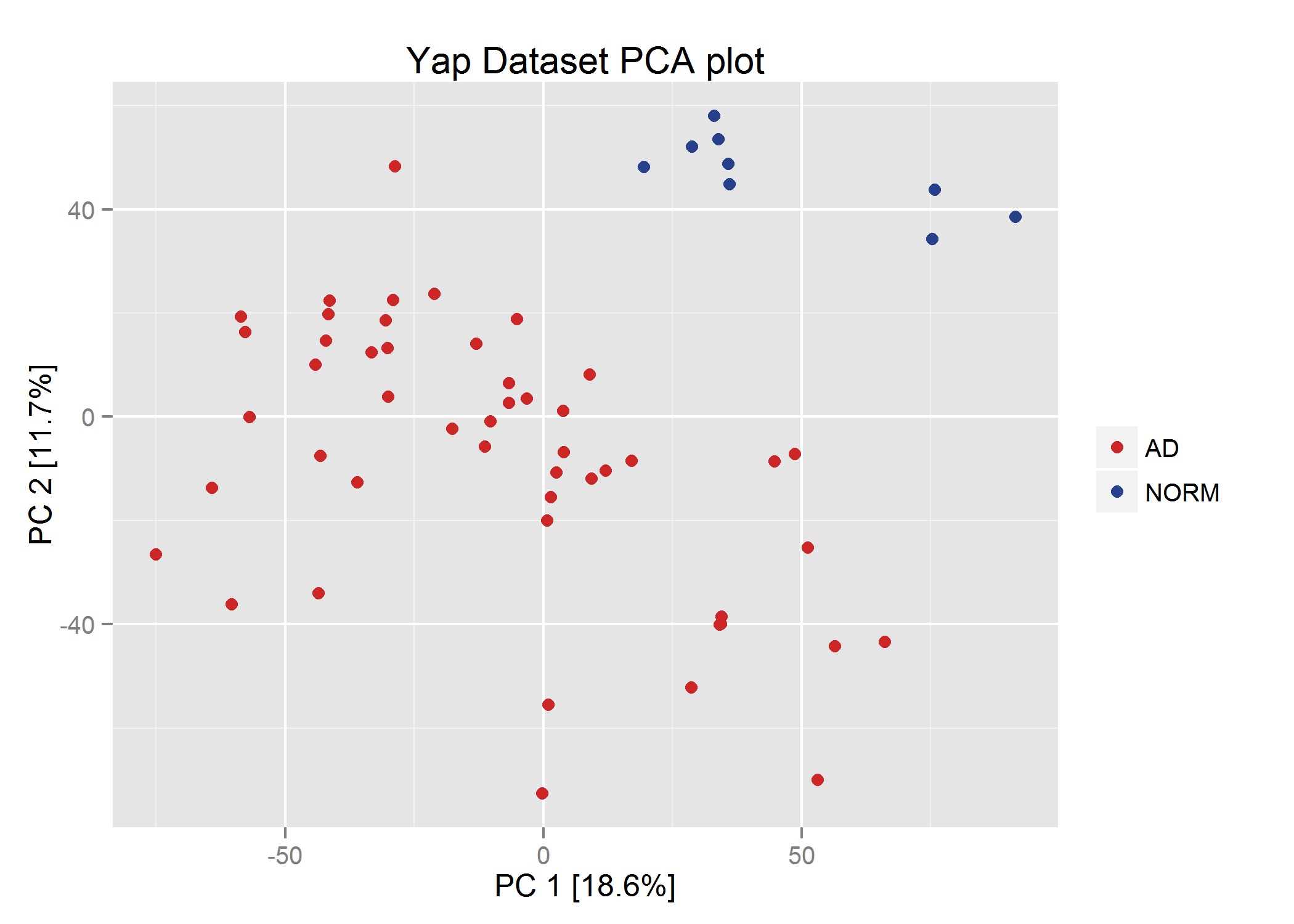

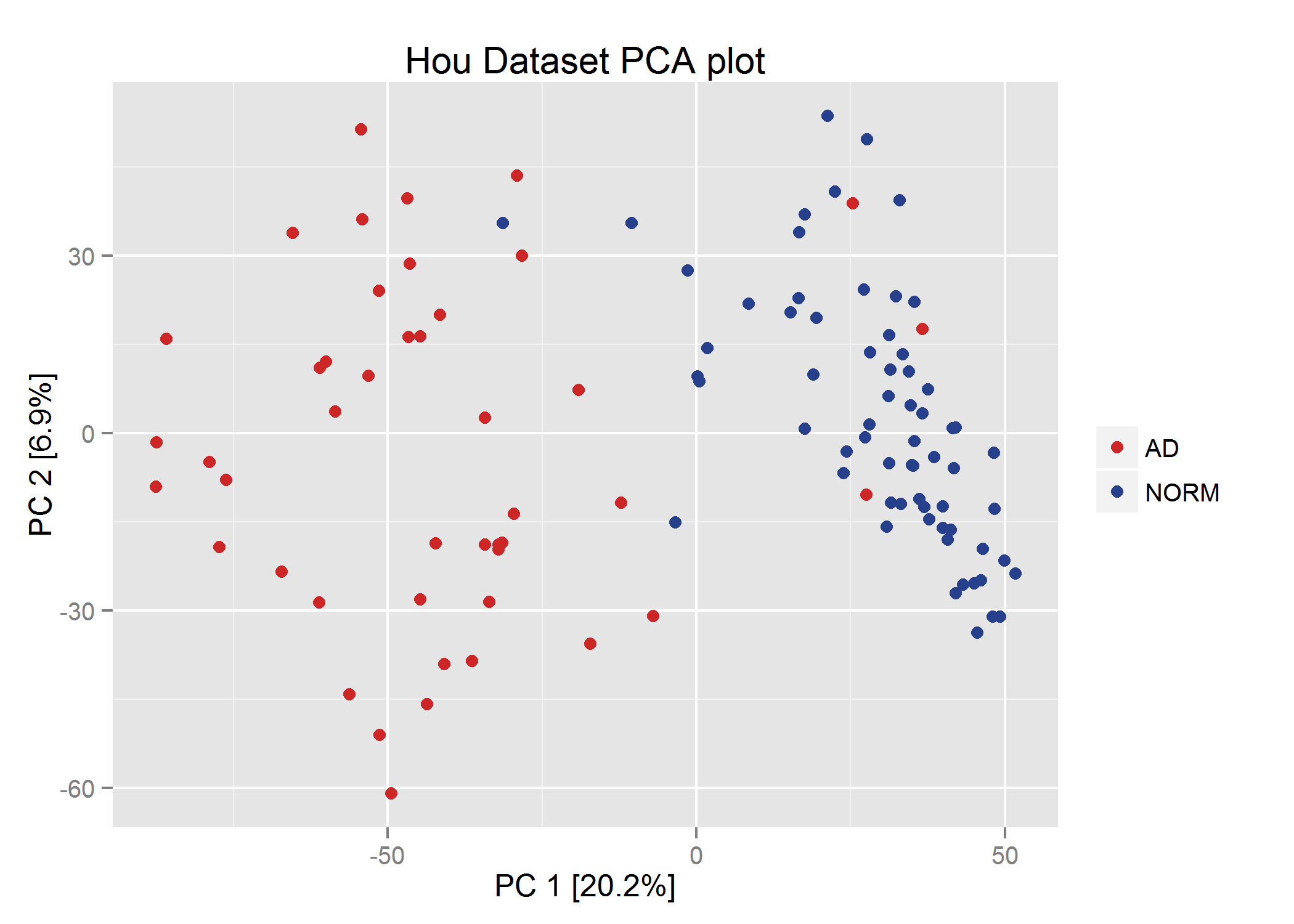


## Statistical comparison of gene ranks

### How to compare different gene rankings?

We used the function "compareLists" from Bioconductor’s "OrderedList" package version 1.34.0 (www.bioconductor.org). This test can measure the similarity between two ordered lists(they must have the exact same elements) and calculate a p-value of this similarity. The null hypothesis that is tested is that the two ordered lists that are being compared are not similar. In order to reject this hypothesis the weighted overlap score (WOS - measure of similarity) of the two lists is computed. The statistical significance of the obtained WOS is calculated with a permutation based approach, where one of the two lists is randomly permuted several times in order to generate the null distributions

The similarity score for two gene rakings $G_{A},G_{B}$ is defined as $WOS\left( G_{A},G_{B} \right)=\sum_{n=1}^{\# genes} w_{\alpha}A_{n}$, where $w_{\alpha}$ is a weight defined as $w_{\alpha}=e^{-\alpha n}$, where *n* is the position of the gene in the list and *a* is a hyper-parameter. $A_{n}=O_{n}(G_{A},G_{B})$ is the overlap at position *n*. A small example of how the $A_{n}$ is calculated is shown in Table 10.

| Rank *n* | $G_{A}$ | $G_{B}$ | $O_{n}\left( G_{A},G_{B} \right)$ |
| --- | --- | --- | --- |
| 1 | 1003 | **6440** | 0 |
| 2 | **6440** | **9754** | 1 |
| 3 | **5175** | **10659** | 1 |
| 4 | **177** | 23673 | 1 |
| 5 | 10266 | **177** | 2 |
| 6 | **9754** | **2040** | 3 |
| 7 | 6943 | **5175** | 4 |
| 8 | **2040** | 23176 | 5 |
| 9 | 2773 | 395 | 5 |
| 10 | **10659** | 825 | 6 |
| *Table 10.* |  |  |  |

As the weight decreases toward zero for large *n*, the summation might stop before reaching rank *n* = *#genes*. The minimum weight which the computation should take into account must be provided and we used the default value of ${10}^{-5}$. We should mention that *α* is set by the user or it can be left empty and it will be determined by the algorithm such that reasonable maximal ranks to be considered result. The *α* can be any number that will result in the weight getting the minimum weight value before the summation reaches the last element of the lists and in our case *α* could not be anything smaller than ≈0.001346. We let the algorithm choose *α* and in most cases it chose 0.004605 which results in comparing the first 2500 genes(or first and last 2500 genes, 5000 in total, in the case that we compare the top and bottom ranks). Based on the overlap of differentially expressed genes, we considered that the number of genes compared is reasonable, therefore we decided to keep the *α* that the algorithm selected.

More detailed information about how this method works can be found [here](http://www.bioconductor.org/packages/release/bioc/vignettes/OrderedList/inst/doc/tr_2006_01.pdf).

### How results are presented

Here is an example of the result of the algorithm on two randomly ordered lists of our genes:

**Comparison of two randomly ordered lists** (alpha set to 0.004605)

List comparison

Assessing similarity of : top ranks

Length of lists : 8556

Number of random samples : 2000

----------------------------------------------------------

Lists are more alike in direct order

Chosen regularization parameter : alpha = 0.005 ( 2500 genes)

Weighted overlap score : 2026.44

Significance of similarity : p-value = 1

Score percentage for common entries : 100

Entries contributing score percentage : 751

First of all, in the results we see if the test was performed on top ranks only or on top and bottom, how many elements each list has and how many permutations were performed in order to generate the null distribution. Then, it reports if the lists are more alike in the direct order (the given order) or if they are more alike in the reversed order, meaning that they are more alike if one of them is reversed.

The *α* that was used is reported next, rounded to the three decimal points, and in the parenthesis is the number of genes that could potentially contribute to the score before the weight becomes smaller than the minimum weight we have provided, this is the maximum rank.

After that, the weighted overlap score (similarity score) and the empirical p-value are reported. WOS does not have standard minimum and maximum values because it is heavily based on the *α* and the weight threshold.

The score percentage of common entries is a parameter that gives us the list of overlapping genes that contributed a certain percentage to the overall similarity score, the number of which is reported below as "Entries contributing score percentage". The number of genes that are taken into consideration are shown in the parenthesis next to *α* as I mentioned before. We set this to 100% in order to know exactly how many genes (even if they contribute very little to the WOS) were found in both lists up until the rank that the algorithm gets to before the weight becomes smaller than the minimum set weight.

We presented below the result of the comparison of the top ranks between two randomly ordered lists. Below, we present the results of the similarity test on top and bottom ranks between two randomly ordered lists and the results of the comparison on top and bottom ranks of two lists that match perfectly, in order to have a sense of the best and worst case.

Comparison of two randomly ordered lists (testing on top and bottom ranks, alpha set to 0.005)

List comparison

Assessing similarity of : top and bottom ranks

Length of lists : 8556

Number of random samples : 2000

----------------------------------------------------------

Lists are more alike in reversed order

Chosen regularization parameter : alpha = 0.005 ( 2302 genes)

Weighted overlap score : 4171.963

Significance of similarity : p-value = 1

Score percentage for common entries : 100

Entries contributing score percentage : 1269

Comparison of two ordered lists that match perfectly (testing for the top ranked elements of the list)

List comparison

Assessing similarity of : top ranks

Length of lists : 8556

Number of random samples : 2000

----------------------------------------------------------

Lists are more alike in direct order

Chosen regularization parameter : alpha = 0.005 ( 2500 genes)

Weighted overlap score : 47146.95

Significance of similarity : p-value = 0

Score percentage for common entries : 100

Entries contributing score percentage : 2500

Comparison of two ordered lists that match perfectly (testing for the top and bottom ranked elements of the list)

List comparison

Assessing similarity of : top and bottom ranks

Length of lists : 8556

Number of random samples : 2000

----------------------------------------------------------

Lists are more alike in direct order

Chosen regularization parameter : alpha = 0.005 ( 2500 genes)

Weighted overlap score : 94293.91

Significance of similarity : p-value = 0

Score percentage for common entries : 100

Entries contributing score percentage : 5000

# Results

## Differential expression

The expression of the genes of each dataset was tested for differential expression and the similarity of the results between the pairs of datasets was measured. First, we ordered the genes based on their false discovery rate adjusted p-values and we calculated the similarity of the lists on the top ranks. Therefore we measure what is the similarity between the most statistically significant differentially expressed genes. These are results we got:

### Ordered based on the adjusted p-values

##### Bhatt. vs Yap

List comparison

Assessing similarity of : top ranks

Length of lists : 8556

Number of random samples : 2000

----------------------------------------------------------

Lists are more alike in direct order

Chosen regularization parameter : alpha = 0.005 ( 2500 genes)

Weighted overlap score : 16146.77

Significance of similarity : p-value = 0

Score percentage for common entries : 100

Entries contributing score percentage : 1177

##### Bhatt. vs Hou

List comparison

Assessing similarity of : top ranks

Length of lists : 8556

Number of random samples : 2000

----------------------------------------------------------

Lists are more alike in direct order

Chosen regularization parameter : alpha = 0.005 ( 2500 genes)

Weighted overlap score : 19407.85

Significance of similarity : p-value = 0

Score percentage for common entries : 100

Entries contributing score percentage : 1438

##### Yap vs Hou

List comparison

Assessing similarity of : top ranks

Length of lists : 8556

Number of random samples : 2000

----------------------------------------------------------

Lists are more alike in direct order

Chosen regularization parameter : alpha = 0.005 ( 2500 genes)

Weighted overlap score : 14595.17

Significance of similarity : p-value = 0

Score percentage for common entries : 100

Entries contributing score percentage : 1219

Then, we ordered them based on the log fold change and we measured their similarity on the top and bottom ranks.

### Ordered based on the log fold change

##### Bhatt. vs Yap

List comparison

Assessing similarity of : top and bottom ranks

Length of lists : 8556

Number of random samples : 2000

----------------------------------------------------------

Lists are more alike in direct order

Chosen regularization parameter : alpha = 0.005 ( 2500 genes)

Weighted overlap score : 42938.09

Significance of similarity : p-value = 0

Score percentage for common entries : 100

Entries contributing score percentage : 2831

##### Bhatt. vs Hou

List comparison

Assessing similarity of : top and bottom ranks

Length of lists : 8556

Number of random samples : 2000

----------------------------------------------------------

Lists are more alike in direct order

Chosen regularization parameter : alpha = 0.005 ( 2500 genes)

Weighted overlap score : 44230.93

Significance of similarity : p-value = 0

Score percentage for common entries : 100

Entries contributing score percentage : 3298

##### Yap vs Hou

List comparison

Assessing similarity of : top and bottom ranks

Length of lists : 8556

Number of random samples : 2000

----------------------------------------------------------

Lists are more alike in direct order

Chosen regularization parameter : alpha = 0.005 ( 2500 genes)

Weighted overlap score : 44097.78

Significance of similarity : p-value = 0

Score percentage for common entries : 100

Entries contributing score percentage : 3057

The results of the comparison on the lists ordered based on the FDR adjusted p-value gives a first indication there is a statistically significant similarity between the differentially expressed genes. The comparison on the lists ordered on the log fold change gives us a stronger proof that the overall expression of the genes in the three datasets has a statistically significant concordance.

### AUC of gene expression

Now, we wanted to check whether the most predictive genes, the ones with higher AUC, are the same or similar between the three datasets therefore the AUC of each gene was calculated. For each dataset a list sorted in descending order based on the AUC was generated and we tested the top ranks of each pair of lists. In order to be able to perform the test all the genes were kept in the analysis regardless if they were differentially expressed or not.

### Ordered based on the AUC (decreasing order)

##### Bhatt. vs Yap

List comparison

Assessing similarity of : top ranks

Length of lists : 8556

Number of random samples : 2000

----------------------------------------------------------

Lists are more alike in direct order

Chosen regularization parameter : alpha = 0.005 ( 2500 genes)

Weighted overlap score : 16155.45

Significance of similarity : p-value = 0

Score percentage for common entries : 100

Entries contributing score percentage : 1161

##### Bhatt. vs Hou

List comparison

Assessing similarity of : top ranks

Length of lists : 8556

Number of random samples : 2000

----------------------------------------------------------

Lists are more alike in direct order

Chosen regularization parameter : alpha = 0.005 ( 2500 genes)

Weighted overlap score : 17744.37

Significance of similarity : p-value = 0

Score percentage for common entries : 100

Entries contributing score percentage : 1431

##### Yap vs Hou

List comparison

Assessing similarity of : top ranks

Length of lists : 8556

Number of random samples : 2000

----------------------------------------------------------

Lists are more alike in direct order

Chosen regularization parameter : alpha = 0.005 ( 2500 genes)

Weighted overlap score : 14408.77

Significance of similarity : p-value = 0

Score percentage for common entries : 100

Entries contributing score percentage : 1250

The results of this analysis indicated a statistically significant high concordance between the lists of the three datasets supporting that our methods are expected to have similar results on other datasets.

In the paper we identified in the Bhatt. dataset the genes with the highest diagnostic value(in terms of AUC). The following table reports the top diagnostic genes of Bhatt. dataset and their respective AUC in the new datasets. Genes that have a drop in AUC between Bhatt. and the respective new dataset of more than 0.4, 0.3, 0.2, 0.1, 0.05 are reported in red, orange, yellow, green and blue, respectively.

The results show that genes with high diagnostic value in the Bhatt. dataset have also high AUC values in the other datasets.

|  | **EntrezIDs** | **Symbols** | **AUC in Bhatt.** | **AUC in Yap** | **AUC in Hou** |
| --- | --- | --- | --- | --- | --- |
| 1 | 1003 | CDH5 | 0.997037664 | 1.000000 | 0.930598291 |
| 2 | 5175 | PECAM1 | 0.997037664 | 0.993197279 | 0.939487179 |
| 3 | 7048 | TGFBR2 | 0.995768091 | 0.789115646 | 0.597948718 |
| 4 | 177 | AGER | 0.995344901 | 1.000000 | 0.94974359 |
| 5 | 7122 | CLDN5 | 0.99492171 | 1.000000 | 0.914188034 |
| 6 | 10266 | RAMP2 | 0.993652137 | 1.000000 | 0.916581197 |
| 7 | 6943 | TCF21 | 0.993228946 | 0.990929705 | 0.936410256 |
| 8 | 2040 | STOM | 0.993228946 | 0.952380952 | 0.962393162 |
| 9 | 2273 | FHL1 | 0.992805755 | 1.000000 | 0.928547009 |
| 10 | 6867 | TACC1 | 0.992805755 | 0.800453515 | 0.953162393 |
| 11 | 10268 | RAMP3 | 0.991959374 | 0.997732426 | 0.947008547 |
| 12 | 7010 | TEK | 0.991959374 | 0.997732426 | 0.949401709 |
| 13 | 2869 | GRK5 | 0.991536183 | 0.997732426 | 0.938803419 |
| 14 | 8547 | FCN3 | 0.991536183 | 0.990929705 | 0.950769231 |
| 15 | 857 | CAV1 | 0.990689801 | 0.977324263 | 0.930940171 |
| 16 | 11170 | FAM107A | 0.990689801 | 0.979591837 | 0.951111111 |
| 17 | 9079 | LDB2 | 0.99026661 | 1.000000 | 0.945299145 |
| 18 | 2878 | GPX3 | 0.99026661 | 0.859410431 | 0.91008547 |
| 19 | 8639 | AOC3 | 0.988997038 | 0.984126984 | 0.95042735 |
| 20 | 762 | CA4 | 0.988997038 | 0.984126984 | 0.941538462 |
| 21 | 2615 | LRRC32 | 0.988573847 | 1.000000 | 0.922393162 |
| 22 | 7075 | TIE1 | 0.987304274 | 0.965986395 | 0.904615385 |
| 23 | 2268 | FGR | 0.987304274 | 0.986394558 | 0.941880342 |
| 24 | 2013 | EMP2 | 0.987304274 | 1.000000 | 0.938119658 |
| 25 | 7450 | VWF | 0.987304274 | 0.990929705 | 0.943931624 |
| 26 | 4881 | NPR1 | 0.986881083 | 0.941043084 | 0.928888889 |
| 27 | 2022 | ENG | 0.986457893 | 0.866213152 | 0.924102564 |
| 28 | 3384 | ICAM2 | 0.986457893 | 0.975056689 | 0.886153846 |
| 29 | 2167 | FABP4 | 0.986457893 | 1.000000 | 0.943247863 |
| 30 | 7049 | TGFBR3 | 0.986457893 | 0.945578231 | 0.951111111 |
| 31 | 2294 | FOXF1 | 0.986034702 | 0.995464853 | 0.92991453 |
| 32 | 5787 | PTPRB | 0.98518832 | 0.920634921 | 0.941196581 |
| 33 | 1808 | DPYSL2 | 0.984341938 | 0.907029478 | 0.940854701 |
| 34 | 9341 | VAMP3 | 0.984341938 | 0.845804989 | 0.554188034 |
| 35 | 8692 | HYAL2 | 0.983495556 | 0.902494331 | 0.847179487 |
| 36 | 4239 | MFAP4 | 0.982649175 | 0.922902494 | 0.905299145 |
| 37 | 10908 | PNPLA6 | 0.982225984 | 0.870748299 | 0.892649573 |
| 38 | 7433 | VIPR1 | 0.981379602 | 0.893424036 | 0.915897436 |
| 39 | 6909 | TBX2 | 0.981379602 | 0.922902494 | 0.878974359 |
| 40 | 2624 | GATA2 | 0.980956411 | 0.988662132 | 0.896068376 |
| 41 | 2719 | GPC3 | 0.980956411 | 0.814058957 | 0.913162393 |
| 42 | 9353 | SLIT2 | 0.980956411 | 0.920634921 | 0.922393162 |
| 43 | 8685 | MARCO | 0.980956411 | 0.977324263 | 0.925811966 |
| 44 | 9413 | FAM189A2 | 0.98053322 | 0.995464853 | 0.942564103 |
| 45 | 125 | ADH1B | 0.98053322 | 0.988662132 | 0.918290598 |
| 46 | 1346 | COX7A1 | 0.979263648 | 1.000000 | 0.934700855 |
| 47 | 4855 | NOTCH4 | 0.979263648 | 1.000000 | 0.915555556 |
| 48 | 7134 | TNNC1 | 0.979263648 | 0.995464853 | 0.945299145 |
| 49 | 9806 | SPOCK2 | 0.977994075 | 0.963718821 | 0.946324786 |
| 50 | 10979 | FERMT2 | 0.977570884 | 0.560090703 | 0.902564103 |
|  | *Table 11.* | Top 50 diagnostic genes of Bhatt. dataset and their AUC in the other two datasets | | | |

1. Bhattacharjee A, Richards WG, Staunton J, Li C, Monti S, et al. (2001) Classification of human lung carcinomas by mRNA expression profiling reveals distinct adenocarcinoma subclasses. Proc Natl Acad Sci U S A 98: 13790-13795.

2. Yap YL, Lam DC, Luc G, Zhang XW, Hernandez D, et al. (2005) Conserved transcription factor binding sites of cancer markers derived from primary lung adenocarcinoma microarrays. Nucleic Acids Res 33: 409-421.

3. Hou J, Aerts J, den Hamer B, van Ijcken W, den Bakker M, et al. (2010) Gene expression-based classification of non-small cell lung carcinomas and survival prediction. PLoS One 5: e10312.
