# Supplementary material for: Hidden Treasures in “Ancient” Microarrays: Gene-Expression Portrays Biology and Potential Resistance Pathways of Major Lung Cancer Subtypes and Normal Tissue
Source: Front Oncol. 2014 Sep 29;4:251. doi: 10.3389/fonc.2014.00251 (PMC4178426; doi:10.3389/fonc.2014.00251)
Supplement: Figure S1 — Clinically relevant differentially expressed pathways in each tissue type versus the rest. The pathway maps are arranged according to the scheme in the left-top corner. Red indicates overexpression in genes, dark green downregulation. In the Cell Cycle pathway, tumor suppressor genes were overexpressed in normal lung tissue while oncogenes and tumor driving cyclins were overexpressed in cancers. The gene thymidylate synthase (TYMS or TS, belonging to the pathway “one carbon pool by folate”) is relevant for tumor growth and is also a treatment target. Notably, TYMS was overexpressed only in the tumors that are generally refractory to the drug pemetrexed, as the squamous and the small-cell lung cancer. TYMS was not overexpressed in mesothelioma, but it is known that TYMS expression is highly variable in this cancer. In the ERBB pathway, the ERBB2/HER2 and ERBB3/HER3 were overexpressed in adenocarcinoma, while the ERBB4 was overexpressed in the carcinoids. [file Data_Sheet_1.ZIP › File S3.ZIP!/GO Analysis/Adenocarcinomas vs Rest Enrichment Analysis/Overrepresented GO terms of Differentially Expressed Genes.html]

Gene to GO BP Conditional test for over-representation

| GOBPID | Pvalue | OddsRatio | ExpCount | Count | Size | Term |
| --- | --- | --- | --- | --- | --- | --- |
| GO:0002504 | <0.0001 | 3.220 | 27 | 46 | 67 | antigen processing and presentation of peptide or polysaccharide antigen via MHC class II |
| GO:0019886 | <0.0001 | 3.149 | 27 | 45 | 66 | antigen processing and presentation of exogenous peptide antigen via MHC class II |
| GO:0002474 | 0.001 | 2.054 | 34 | 49 | 84 | antigen processing and presentation of peptide antigen via MHC class I |
| GO:0050808 | 0.001 | 1.922 | 40 | 55 | 97 | synapse organization |
| GO:0006614 | 0.001 | 2.081 | 31 | 44 | 75 | SRP-dependent cotranslational protein targeting to membrane |
| GO:0006414 | 0.001 | 2.123 | 29 | 42 | 71 | translational elongation |
| GO:0006020 | 0.002 | Inf | 3 | 7 | 7 | inositol metabolic process |
| GO:0018279 | 0.003 | 2.108 | 25 | 36 | 61 | protein N-linked glycosylation via asparagine |
| GO:0034340 | 0.003 | 2.108 | 25 | 36 | 61 | response to type I interferon |
| GO:0060337 | 0.003 | 2.108 | 25 | 36 | 61 | type I interferon-mediated signaling pathway |
| GO:0043687 | 0.003 | 1.731 | 44 | 59 | 109 | post-translational protein modification |
| GO:0051965 | 0.003 | 7.297 | 5 | 10 | 12 | positive regulation of synapse assembly |
| GO:0071697 | 0.003 | 7.297 | 5 | 10 | 12 | ectodermal placode morphogenesis |
| GO:0000184 | 0.003 | 1.861 | 34 | 47 | 84 | nuclear-transcribed mRNA catabolic process, nonsense-mediated decay |
| GO:0072599 | 0.004 | 1.896 | 32 | 44 | 78 | establishment of protein localization to endoplasmic reticulum |
| GO:0070727 | 0.004 | 1.249 | 268 | 301 | 659 | cellular macromolecule localization |
| GO:0006415 | 0.004 | 2.006 | 26 | 37 | 64 | translational termination |
| GO:0050919 | 0.004 | 11.670 | 4 | 8 | 9 | negative chemotaxis |
| GO:0045184 | 0.004 | 1.217 | 339 | 375 | 833 | establishment of protein localization |
| GO:0032984 | 0.005 | 1.609 | 53 | 68 | 130 | macromolecular complex disassembly |
| GO:0051649 | 0.005 | 1.176 | 512 | 554 | 1258 | establishment of localization in cell |
| GO:0071702 | 0.006 | 1.172 | 516 | 557 | 1267 | organic substance transport |
| GO:0060333 | 0.006 | 1.970 | 25 | 35 | 61 | interferon-gamma-mediated signaling pathway |
| GO:0006987 | 0.007 | 2.061 | 22 | 31 | 53 | activation of signaling protein activity involved in unfolded protein response |
| GO:0090317 | 0.007 | 2.121 | 20 | 29 | 49 | negative regulation of intracellular protein transport |
| GO:0040036 | 0.007 | 3.795 | 7 | 13 | 18 | regulation of fibroblast growth factor receptor signaling pathway |
| GO:0051220 | 0.007 | 3.795 | 7 | 13 | 18 | cytoplasmic sequestering of protein |
| GO:0051289 | 0.007 | 2.339 | 16 | 24 | 39 | protein homotetramerization |
| GO:0045664 | 0.007 | 1.398 | 96 | 115 | 236 | regulation of neuron differentiation |
| GO:0044085 | 0.009 | 1.173 | 441 | 477 | 1082 | cellular component biogenesis |
| GO:0035176 | 0.009 | 2.920 | 10 | 16 | 24 | social behavior |
| GO:0032069 | 0.009 | 1.931 | 24 | 33 | 58 | regulation of nuclease activity |
| GO:0006612 | 0.009 | 1.606 | 44 | 57 | 109 | protein targeting to membrane |
| GO:0001967 | 0.010 | 10.208 | 3 | 7 | 8 | suckling behavior |
| GO:0042345 | 0.010 | 2.758 | 11 | 17 | 26 | regulation of NF-kappaB import into nucleus |
| GO:0046823 | 0.010 | 2.077 | 19 | 27 | 46 | negative regulation of nucleocytoplasmic transport |
| GO:0048675 | 0.011 | 2.149 | 17 | 25 | 42 | axon extension |
| GO:0009296 | 0.011 | Inf | 2 | 5 | 5 | flagellum assembly |
| GO:0021683 | 0.011 | Inf | 2 | 5 | 5 | cerebellar granular layer morphogenesis |
| GO:0060789 | 0.011 | Inf | 2 | 5 | 5 | hair follicle placode formation |
| GO:0010629 | 0.012 | 1.217 | 248 | 275 | 609 | negative regulation of gene expression |
| GO:0007249 | 0.013 | 1.487 | 57 | 70 | 139 | I-kappaB kinase/NF-kappaB cascade |
| GO:0016079 | 0.014 | 3.167 | 8 | 13 | 19 | synaptic vesicle exocytosis |
| GO:0001919 | 0.014 | 5.834 | 4 | 8 | 10 | regulation of receptor recycling |
| GO:0071526 | 0.014 | 5.834 | 4 | 8 | 10 | semaphorin-plexin signaling pathway |
| GO:0031344 | 0.014 | 1.383 | 82 | 98 | 202 | regulation of cell projection organization |
| GO:0043624 | 0.015 | 2.012 | 18 | 26 | 45 | cellular protein complex disassembly |
| GO:0008038 | 0.015 | 2.919 | 9 | 14 | 21 | neuron recognition |
| GO:0048169 | 0.015 | 2.919 | 9 | 14 | 21 | regulation of long-term neuronal synaptic plasticity |
| GO:0042992 | 0.016 | 2.482 | 11 | 17 | 27 | negative regulation of transcription factor import into nucleus |
| GO:0050885 | 0.016 | 2.312 | 13 | 19 | 31 | neuromuscular process controlling balance |
| GO:0050770 | 0.017 | 1.666 | 31 | 41 | 77 | regulation of axonogenesis |
| GO:0048813 | 0.019 | 1.827 | 22 | 30 | 54 | dendrite morphogenesis |
| GO:0006457 | 0.021 | 1.444 | 56 | 68 | 137 | protein folding |
| GO:0002295 | 0.021 | 8.747 | 3 | 6 | 7 | T-helper cell lineage commitment |
| GO:0006349 | 0.021 | 8.747 | 3 | 6 | 7 | regulation of gene expression by genetic imprinting |
| GO:0007253 | 0.021 | 8.747 | 3 | 6 | 7 | cytoplasmic sequestering of NF-kappaB |
| GO:0019934 | 0.021 | 8.747 | 3 | 6 | 7 | cGMP-mediated signaling |
| GO:0046855 | 0.021 | 8.747 | 3 | 6 | 7 | inositol phosphate dephosphorylation |
| GO:0071425 | 0.021 | 8.747 | 3 | 6 | 7 | hematopoietic stem cell proliferation |
| GO:0072539 | 0.021 | 8.747 | 3 | 6 | 7 | T-helper 17 cell differentiation |
| GO:0006342 | 0.024 | 2.918 | 7 | 12 | 18 | chromatin silencing |
| GO:0000122 | 0.024 | 1.245 | 147 | 166 | 362 | negative regulation of transcription from RNA polymerase II promoter |
| GO:0006984 | 0.025 | 1.636 | 29 | 38 | 72 | ER-nucleus signaling pathway |
| GO:0000041 | 0.025 | 1.743 | 23 | 31 | 57 | transition metal ion transport |
| GO:0051494 | 0.025 | 1.743 | 23 | 31 | 57 | negative regulation of cytoskeleton organization |
| GO:1901880 | 0.025 | 2.134 | 13 | 19 | 32 | negative regulation of protein depolymerization |
| GO:0051253 | 0.025 | 1.190 | 236 | 259 | 580 | negative regulation of RNA metabolic process |
| GO:0051966 | 0.026 | 2.432 | 10 | 15 | 24 | regulation of synaptic transmission, glutamatergic |
| GO:0048858 | 0.026 | 1.208 | 194 | 215 | 477 | cell projection morphogenesis |
| GO:0009100 | 0.026 | 1.323 | 87 | 101 | 213 | glycoprotein metabolic process |
| GO:0035966 | 0.027 | 1.492 | 43 | 53 | 105 | response to topologically incorrect protein |
| GO:0071822 | 0.027 | 1.170 | 288 | 313 | 708 | protein complex subunit organization |
| GO:0000389 | 0.027 | Inf | 2 | 4 | 4 | mRNA 3'-splice site recognition |
| GO:0006983 | 0.027 | Inf | 2 | 4 | 4 | ER overload response |
| GO:0008298 | 0.027 | Inf | 2 | 4 | 4 | intracellular mRNA localization |
| GO:0016024 | 0.027 | Inf | 2 | 4 | 4 | CDP-diacylglycerol biosynthetic process |
| GO:0016233 | 0.027 | Inf | 2 | 4 | 4 | telomere capping |
| GO:0018027 | 0.027 | Inf | 2 | 4 | 4 | peptidyl-lysine dimethylation |
| GO:0021707 | 0.027 | Inf | 2 | 4 | 4 | cerebellar granule cell differentiation |
| GO:0021797 | 0.027 | Inf | 2 | 4 | 4 | forebrain anterior/posterior pattern specification |
| GO:0032847 | 0.027 | Inf | 2 | 4 | 4 | regulation of cellular pH reduction |
| GO:0051290 | 0.027 | Inf | 2 | 4 | 4 | protein heterotetramerization |
| GO:0060666 | 0.027 | Inf | 2 | 4 | 4 | dichotomous subdivision of terminal units involved in salivary gland branching |
| GO:0060686 | 0.027 | Inf | 2 | 4 | 4 | negative regulation of prostatic bud formation |
| GO:0070100 | 0.027 | Inf | 2 | 4 | 4 | negative regulation of chemokine-mediated signaling pathway |
| GO:0070198 | 0.027 | Inf | 2 | 4 | 4 | protein localization to chromosome, telomeric region |
| GO:0070245 | 0.027 | Inf | 2 | 4 | 4 | positive regulation of thymocyte apoptotic process |
| GO:0072578 | 0.027 | Inf | 2 | 4 | 4 | neurotransmitter-gated ion channel clustering |
| GO:2000303 | 0.027 | Inf | 2 | 4 | 4 | regulation of ceramide biosynthetic process |
| GO:0036092 | 0.028 | 5.103 | 4 | 7 | 9 | phosphatidylinositol-3-phosphate biosynthetic process |
| GO:0043368 | 0.028 | 5.103 | 4 | 7 | 9 | positive T cell selection |
| GO:0045723 | 0.028 | 5.103 | 4 | 7 | 9 | positive regulation of fatty acid biosynthetic process |
| GO:0071294 | 0.028 | 5.103 | 4 | 7 | 9 | cellular response to zinc ion |
| GO:0010976 | 0.028 | 1.779 | 21 | 28 | 51 | positive regulation of neuron projection development |
| GO:0045017 | 0.028 | 1.403 | 57 | 69 | 141 | glycerolipid biosynthetic process |
| GO:0045185 | 0.028 | 1.578 | 32 | 41 | 79 | maintenance of protein location |
| GO:0006402 | 0.029 | 1.440 | 49 | 60 | 121 | mRNA catabolic process |
| GO:0019083 | 0.029 | 1.440 | 49 | 60 | 121 | viral transcription |
| GO:0002479 | 0.030 | 1.656 | 26 | 34 | 64 | antigen processing and presentation of exogenous peptide antigen via MHC class I, TAP-dependent |
| GO:0051651 | 0.030 | 1.581 | 31 | 40 | 77 | maintenance of location in cell |
| GO:0006334 | 0.030 | 1.809 | 19 | 26 | 47 | nucleosome assembly |
| GO:0051172 | 0.032 | 1.170 | 265 | 288 | 651 | negative regulation of nitrogen compound metabolic process |
| GO:0019538 | 0.032 | 1.101 | 898 | 935 | 2216 | protein metabolic process |
| GO:2000113 | 0.032 | 1.172 | 257 | 279 | 630 | negative regulation of cellular macromolecule biosynthetic process |
| GO:0007004 | 0.033 | 3.888 | 4 | 8 | 11 | telomere maintenance via telomerase |
| GO:0016338 | 0.033 | 3.888 | 4 | 8 | 11 | calcium-independent cell-cell adhesion |
| GO:0021591 | 0.033 | 3.888 | 4 | 8 | 11 | ventricular system development |
| GO:0046174 | 0.033 | 3.888 | 4 | 8 | 11 | polyol catabolic process |
| GO:0051129 | 0.034 | 1.285 | 97 | 111 | 238 | negative regulation of cellular component organization |
| GO:0051641 | 0.035 | 2.757 | 7 | 11 | 17 | cellular localization |
| GO:0030193 | 0.035 | 1.686 | 23 | 30 | 56 | regulation of blood coagulation |
| GO:0065003 | 0.035 | 1.163 | 275 | 298 | 676 | macromolecular complex assembly |
| GO:0002478 | 0.035 | 1.618 | 26 | 34 | 65 | antigen processing and presentation of exogenous peptide antigen |
| GO:0032272 | 0.035 | 1.916 | 15 | 21 | 37 | negative regulation of protein polymerization |
| GO:0006470 | 0.035 | 1.463 | 42 | 51 | 102 | protein dephosphorylation |
| GO:0097066 | 0.036 | 3.281 | 5 | 9 | 13 | response to thyroid hormone stimulus |
| GO:0006886 | 0.037 | 1.198 | 182 | 201 | 449 | intracellular protein transport |
| GO:0034620 | 0.037 | 1.598 | 27 | 35 | 67 | cellular response to unfolded protein |
| GO:0046394 | 0.037 | 1.317 | 77 | 89 | 188 | carboxylic acid biosynthetic process |
| GO:0034341 | 0.038 | 1.495 | 36 | 45 | 89 | response to interferon-gamma |
| GO:0007215 | 0.038 | 2.021 | 13 | 18 | 31 | glutamate receptor signaling pathway |
| GO:0010771 | 0.038 | 2.917 | 6 | 10 | 15 | negative regulation of cell morphogenesis involved in differentiation |
| GO:0032288 | 0.038 | 2.917 | 6 | 10 | 15 | myelin assembly |
| GO:1901071 | 0.038 | 2.917 | 6 | 10 | 15 | glucosamine-containing compound metabolic process |
| GO:0051960 | 0.040 | 1.229 | 133 | 149 | 327 | regulation of nervous system development |
| GO:0001736 | 0.040 | 2.674 | 7 | 11 | 17 | establishment of planar polarity |
| GO:0000245 | 0.040 | 2.123 | 11 | 16 | 27 | spliceosomal complex assembly |
| GO:0001913 | 0.041 | 2.501 | 8 | 12 | 19 | T cell mediated cytotoxicity |
| GO:0048199 | 0.041 | 2.501 | 8 | 12 | 19 | vesicle targeting, to, from or within Golgi |
| GO:0060441 | 0.041 | 2.501 | 8 | 12 | 19 | epithelial tube branching involved in lung morphogenesis |
| GO:0033572 | 0.041 | 2.270 | 9 | 14 | 23 | transferrin transport |
| GO:0071230 | 0.041 | 2.270 | 9 | 14 | 23 | cellular response to amino acid stimulus |
| GO:0072512 | 0.041 | 2.270 | 9 | 14 | 23 | trivalent inorganic cation transport |
| GO:0007157 | 0.041 | 2.371 | 9 | 13 | 21 | heterophilic cell-cell adhesion |
| GO:0016567 | 0.042 | 1.232 | 126 | 141 | 309 | protein ubiquitination |
| GO:0006641 | 0.043 | 1.545 | 29 | 37 | 72 | triglyceride metabolic process |
| GO:0006002 | 0.044 | 7.287 | 2 | 5 | 6 | fructose 6-phosphate metabolic process |
| GO:0007185 | 0.044 | 7.287 | 2 | 5 | 6 | transmembrane receptor protein tyrosine phosphatase signaling pathway |
| GO:0009649 | 0.044 | 7.287 | 2 | 5 | 6 | entrainment of circadian clock |
| GO:0010873 | 0.044 | 7.287 | 2 | 5 | 6 | positive regulation of cholesterol esterification |
| GO:0016322 | 0.044 | 7.287 | 2 | 5 | 6 | neuron remodeling |
| GO:0019471 | 0.044 | 7.287 | 2 | 5 | 6 | 4-hydroxyproline metabolic process |
| GO:0021877 | 0.044 | 7.287 | 2 | 5 | 6 | forebrain neuron fate commitment |
| GO:0030490 | 0.044 | 7.287 | 2 | 5 | 6 | maturation of SSU-rRNA |
| GO:0043097 | 0.044 | 7.287 | 2 | 5 | 6 | pyrimidine nucleoside salvage |
| GO:0043587 | 0.044 | 7.287 | 2 | 5 | 6 | tongue morphogenesis |
| GO:0043589 | 0.044 | 7.287 | 2 | 5 | 6 | skin morphogenesis |
| GO:0048172 | 0.044 | 7.287 | 2 | 5 | 6 | regulation of short-term neuronal synaptic plasticity |
| GO:0051546 | 0.044 | 7.287 | 2 | 5 | 6 | keratinocyte migration |
| GO:0060235 | 0.044 | 7.287 | 2 | 5 | 6 | lens induction in camera-type eye |
| GO:0061072 | 0.044 | 7.287 | 2 | 5 | 6 | iris morphogenesis |
| GO:2000300 | 0.044 | 7.287 | 2 | 5 | 6 | regulation of synaptic vesicle exocytosis |
| GO:2000311 | 0.044 | 7.287 | 2 | 5 | 6 | regulation of alpha-amino-3-hydroxy-5-methyl-4-isoxazole propionate selective glutamate receptor activity |
| GO:2001273 | 0.044 | 7.287 | 2 | 5 | 6 | regulation of glucose import in response to insulin stimulus |
| GO:0009267 | 0.045 | 1.623 | 23 | 30 | 57 | cellular response to starvation |
| GO:0045638 | 0.046 | 1.767 | 17 | 23 | 42 | negative regulation of myeloid cell differentiation |
| GO:1901616 | 0.046 | 1.767 | 17 | 23 | 42 | organic hydroxy compound catabolic process |
| GO:0010970 | 0.048 | 1.784 | 16 | 22 | 40 | microtubule-based transport |
| GO:0009890 | 0.049 | 1.145 | 286 | 307 | 702 | negative regulation of biosynthetic process |
| GO:0002040 | 0.049 | 1.803 | 15 | 21 | 38 | sprouting angiogenesis |
| GO:0050819 | 0.049 | 1.803 | 15 | 21 | 38 | negative regulation of coagulation |
| GO:0051648 | 0.050 | 1.636 | 22 | 28 | 53 | vesicle localization |
| GO:0070647 | 0.050 | 1.201 | 150 | 166 | 369 | protein modification by small protein conjugation or removal |
