# Supplementary material for: Hidden Treasures in “Ancient” Microarrays: Gene-Expression Portrays Biology and Potential Resistance Pathways of Major Lung Cancer Subtypes and Normal Tissue
Source: Front Oncol. 2014 Sep 29;4:251. doi: 10.3389/fonc.2014.00251 (PMC4178426; doi:10.3389/fonc.2014.00251)
Supplement: Figure S1 — Clinically relevant differentially expressed pathways in each tissue type versus the rest. The pathway maps are arranged according to the scheme in the left-top corner. Red indicates overexpression in genes, dark green downregulation. In the Cell Cycle pathway, tumor suppressor genes were overexpressed in normal lung tissue while oncogenes and tumor driving cyclins were overexpressed in cancers. The gene thymidylate synthase (TYMS or TS, belonging to the pathway “one carbon pool by folate”) is relevant for tumor growth and is also a treatment target. Notably, TYMS was overexpressed only in the tumors that are generally refractory to the drug pemetrexed, as the squamous and the small-cell lung cancer. TYMS was not overexpressed in mesothelioma, but it is known that TYMS expression is highly variable in this cancer. In the ERBB pathway, the ERBB2/HER2 and ERBB3/HER3 were overexpressed in adenocarcinoma, while the ERBB4 was overexpressed in the carcinoids. [file Data_Sheet_1.ZIP › File S3.ZIP!/GO Analysis/Adenocarcinomas vs Rest Enrichment Analysis/Overrepresented GO terms of Down-regulated Genes.html]

Gene to GO BP Conditional test for over-representation

| GOBPID | Pvalue | OddsRatio | ExpCount | Count | Size | Term |
| --- | --- | --- | --- | --- | --- | --- |
| GO:0030030 | <0.0001 | 1.591 | 147 | 200 | 652 | cell projection organization |
| GO:0045934 | <0.0001 | 1.544 | 144 | 193 | 641 | negative regulation of nucleobase-containing compound metabolic process |
| GO:0007399 | <0.0001 | 1.511 | 154 | 203 | 700 | nervous system development |
| GO:0031327 | <0.0001 | 1.504 | 155 | 204 | 690 | negative regulation of cellular biosynthetic process |
| GO:0010558 | <0.0001 | 1.516 | 148 | 196 | 659 | negative regulation of macromolecule biosynthetic process |
| GO:0032990 | <0.0001 | 1.608 | 108 | 150 | 482 | cell part morphogenesis |
| GO:0071840 | <0.0001 | 1.256 | 611 | 683 | 2717 | cellular component organization or biogenesis |
| GO:0000122 | <0.0001 | 1.624 | 81 | 114 | 362 | negative regulation of transcription from RNA polymerase II promoter |
| GO:0050808 | <0.0001 | 2.346 | 22 | 39 | 97 | synapse organization |
| GO:0007017 | <0.0001 | 1.714 | 56 | 81 | 247 | microtubule-based process |
| GO:0010468 | <0.0001 | 1.255 | 436 | 496 | 1940 | regulation of gene expression |
| GO:0050767 | <0.0001 | 1.610 | 67 | 93 | 296 | regulation of neurogenesis |
| GO:0000902 | <0.0001 | 1.388 | 148 | 185 | 658 | cell morphogenesis |
| GO:0070647 | <0.0001 | 1.556 | 73 | 99 | 324 | protein modification by small protein conjugation or removal |
| GO:0048523 | <0.0001 | 1.232 | 451 | 507 | 2006 | negative regulation of cellular process |
| GO:0007215 | <0.0001 | 3.699 | 7 | 16 | 31 | glutamate receptor signaling pathway |
| GO:0042773 | <0.0001 | 3.469 | 8 | 17 | 34 | ATP synthesis coupled electron transport |
| GO:0048812 | <0.0001 | 1.494 | 85 | 112 | 378 | neuron projection morphogenesis |
| GO:0006120 | <0.0001 | 4.043 | 6 | 14 | 26 | mitochondrial electron transport, NADH to ubiquinone |
| GO:0048667 | <0.0001 | 1.491 | 84 | 111 | 375 | cell morphogenesis involved in neuron differentiation |
| GO:0048699 | 0.001 | 1.454 | 97 | 126 | 439 | generation of neurons |
| GO:0009296 | 0.001 | Inf | 1 | 5 | 5 | flagellum assembly |
| GO:0036092 | 0.001 | 12.102 | 2 | 7 | 9 | phosphatidylinositol-3-phosphate biosynthetic process |
| GO:0071294 | 0.001 | 12.102 | 2 | 7 | 9 | cellular response to zinc ion |
| GO:0051969 | 0.001 | 1.762 | 37 | 55 | 164 | regulation of transmission of nerve impulse |
| GO:0048813 | 0.001 | 2.577 | 12 | 23 | 54 | dendrite morphogenesis |
| GO:0009892 | 0.001 | 1.284 | 229 | 269 | 1016 | negative regulation of metabolic process |
| GO:0010976 | 0.001 | 2.634 | 11 | 22 | 51 | positive regulation of neuron projection development |
| GO:0045665 | 0.001 | 3.103 | 8 | 17 | 36 | negative regulation of neuron differentiation |
| GO:0031323 | 0.001 | 1.193 | 596 | 651 | 2650 | regulation of cellular metabolic process |
| GO:2000112 | 0.001 | 1.214 | 402 | 450 | 1787 | regulation of cellular macromolecule biosynthetic process |
| GO:0071696 | 0.001 | 6.917 | 3 | 8 | 12 | ectodermal placode development |
| GO:2001141 | 0.001 | 1.217 | 369 | 415 | 1641 | regulation of RNA biosynthetic process |
| GO:0040014 | 0.001 | 2.463 | 12 | 22 | 53 | regulation of multicellular organism growth |
| GO:0048869 | 0.002 | 1.203 | 418 | 465 | 1859 | cellular developmental process |
| GO:0032288 | 0.002 | 5.189 | 3 | 9 | 15 | myelin assembly |
| GO:0022607 | 0.002 | 1.259 | 225 | 262 | 1003 | cellular component assembly |
| GO:0003008 | 0.002 | 1.238 | 270 | 309 | 1199 | system process |
| GO:0006342 | 0.002 | 4.325 | 4 | 10 | 18 | chromatin silencing |
| GO:0051171 | 0.002 | 1.188 | 466 | 513 | 2072 | regulation of nitrogen compound metabolic process |
| GO:0044708 | 0.002 | 1.502 | 59 | 79 | 263 | single-organism behavior |
| GO:0016567 | 0.002 | 1.608 | 42 | 59 | 188 | protein ubiquitination |
| GO:0034622 | 0.002 | 1.431 | 78 | 100 | 345 | cellular macromolecular complex assembly |
| GO:0000389 | 0.003 | Inf | 1 | 4 | 4 | mRNA 3'-splice site recognition |
| GO:0051290 | 0.003 | Inf | 1 | 4 | 4 | protein heterotetramerization |
| GO:0070198 | 0.003 | Inf | 1 | 4 | 4 | protein localization to chromosome, telomeric region |
| GO:0007157 | 0.003 | 3.807 | 5 | 11 | 21 | heterophilic cell-cell adhesion |
| GO:0048169 | 0.003 | 3.807 | 5 | 11 | 21 | regulation of long-term neuronal synaptic plasticity |
| GO:0072384 | 0.003 | 3.807 | 5 | 11 | 21 | organelle transport along microtubule |
| GO:0048172 | 0.003 | 17.271 | 1 | 5 | 6 | regulation of short-term neuronal synaptic plasticity |
| GO:2000300 | 0.003 | 17.271 | 1 | 5 | 6 | regulation of synaptic vesicle exocytosis |
| GO:2000311 | 0.003 | 17.271 | 1 | 5 | 6 | regulation of alpha-amino-3-hydroxy-5-methyl-4-isoxazole propionate selective glutamate receptor activity |
| GO:0015991 | 0.003 | 3.462 | 5 | 12 | 24 | ATP hydrolysis coupled proton transport |
| GO:0016568 | 0.003 | 1.864 | 22 | 34 | 98 | chromatin modification |
| GO:0007156 | 0.004 | 2.135 | 14 | 24 | 63 | homophilic cell adhesion |
| GO:0010970 | 0.004 | 3.862 | 4 | 10 | 19 | microtubule-based transport |
| GO:0031109 | 0.004 | 3.000 | 6 | 13 | 28 | microtubule polymerization or depolymerization |
| GO:0007420 | 0.004 | 1.393 | 79 | 100 | 352 | brain development |
| GO:0080090 | 0.005 | 1.161 | 594 | 640 | 2640 | regulation of primary metabolic process |
| GO:0031333 | 0.006 | 2.119 | 13 | 22 | 58 | negative regulation of protein complex assembly |
| GO:0050789 | 0.006 | 1.156 | 1115 | 1161 | 4958 | regulation of biological process |
| GO:0050919 | 0.006 | 6.910 | 2 | 6 | 9 | negative chemotaxis |
| GO:0016079 | 0.006 | 3.902 | 4 | 9 | 17 | synaptic vesicle exocytosis |
| GO:0016486 | 0.006 | 3.890 | 4 | 9 | 17 | peptide hormone processing |
| GO:0031069 | 0.006 | 3.890 | 4 | 9 | 17 | hair follicle morphogenesis |
| GO:0042490 | 0.006 | 2.693 | 7 | 14 | 32 | mechanoreceptor differentiation |
| GO:0070646 | 0.006 | 2.310 | 10 | 18 | 45 | protein modification by small protein removal |
| GO:0051128 | 0.006 | 1.302 | 119 | 143 | 535 | regulation of cellular component organization |
| GO:2001251 | 0.006 | 2.812 | 7 | 13 | 29 | negative regulation of chromosome organization |
| GO:0060079 | 0.006 | 3.177 | 5 | 11 | 23 | regulation of excitatory postsynaptic membrane potential |
| GO:0045892 | 0.006 | 1.539 | 40 | 55 | 183 | negative regulation of transcription, DNA-dependent |
| GO:0033572 | 0.006 | 3.172 | 5 | 11 | 23 | transferrin transport |
| GO:0072512 | 0.006 | 3.172 | 5 | 11 | 23 | trivalent inorganic cation transport |
| GO:0048675 | 0.007 | 2.356 | 9 | 17 | 42 | axon extension |
| GO:0033043 | 0.007 | 1.366 | 80 | 100 | 356 | regulation of organelle organization |
| GO:0019228 | 0.007 | 1.827 | 20 | 30 | 87 | regulation of action potential in neuron |
| GO:0071824 | 0.007 | 1.827 | 20 | 30 | 87 | protein-DNA complex subunit organization |
| GO:0006650 | 0.007 | 1.600 | 33 | 46 | 146 | glycerophospholipid metabolic process |
| GO:0006836 | 0.007 | 1.763 | 22 | 33 | 98 | neurotransmitter transport |
| GO:0042391 | 0.007 | 1.477 | 48 | 64 | 215 | regulation of membrane potential |
| GO:0007049 | 0.007 | 1.224 | 207 | 237 | 921 | cell cycle |
| GO:0050890 | 0.008 | 1.611 | 31 | 44 | 139 | cognition |
| GO:0046855 | 0.008 | 8.634 | 2 | 5 | 7 | inositol phosphate dephosphorylation |
| GO:0048505 | 0.008 | 8.634 | 2 | 5 | 7 | regulation of timing of cell differentiation |
| GO:0042384 | 0.008 | 2.551 | 7 | 14 | 33 | cilium assembly |
| GO:0051276 | 0.009 | 1.326 | 92 | 112 | 409 | chromosome organization |
| GO:0043632 | 0.009 | 1.422 | 57 | 73 | 252 | modification-dependent macromolecule catabolic process |
| GO:0050885 | 0.009 | 2.772 | 6 | 12 | 27 | neuromuscular process controlling balance |
| GO:0006470 | 0.009 | 1.739 | 22 | 32 | 96 | protein dephosphorylation |
| GO:0000245 | 0.009 | 2.768 | 6 | 12 | 27 | spliceosomal complex assembly |
| GO:0007268 | 0.009 | 1.368 | 72 | 90 | 323 | synaptic transmission |
| GO:0019226 | 0.009 | 14.325 | 1 | 4 | 5 | transmission of nerve impulse |
| GO:0035176 | 0.010 | 2.927 | 5 | 11 | 24 | social behavior |
| GO:0022403 | 0.010 | 1.272 | 126 | 149 | 560 | cell cycle phase |
| GO:0045017 | 0.010 | 1.577 | 32 | 44 | 141 | glycerolipid biosynthetic process |
| GO:0016070 | 0.010 | 1.150 | 496 | 535 | 2204 | RNA metabolic process |
| GO:0000730 | 0.010 | 13.809 | 1 | 4 | 5 | DNA recombinase assembly |
| GO:0016188 | 0.010 | 13.809 | 1 | 4 | 5 | synaptic vesicle maturation |
| GO:0016198 | 0.010 | 13.809 | 1 | 4 | 5 | axon choice point recognition |
| GO:0021683 | 0.010 | 13.809 | 1 | 4 | 5 | cerebellar granular layer morphogenesis |
| GO:0034661 | 0.010 | 13.809 | 1 | 4 | 5 | ncRNA catabolic process |
| GO:0045836 | 0.010 | 13.809 | 1 | 4 | 5 | positive regulation of meiosis |
| GO:0048026 | 0.010 | 13.809 | 1 | 4 | 5 | positive regulation of mRNA splicing, via spliceosome |
| GO:0048554 | 0.010 | 13.809 | 1 | 4 | 5 | positive regulation of metalloenzyme activity |
| GO:0060789 | 0.010 | 13.809 | 1 | 4 | 5 | hair follicle placode formation |
| GO:0070932 | 0.010 | 13.809 | 1 | 4 | 5 | histone H3 deacetylation |
| GO:0097120 | 0.010 | 13.809 | 1 | 4 | 5 | receptor localization to synapse |
| GO:0010927 | 0.011 | 2.445 | 8 | 14 | 34 | cellular component assembly involved in morphogenesis |
| GO:0007411 | 0.011 | 1.388 | 62 | 79 | 278 | axon guidance |
| GO:0006511 | 0.011 | 1.414 | 55 | 71 | 246 | ubiquitin-dependent protein catabolic process |
| GO:0009987 | 0.011 | 1.293 | 1129 | 1151 | 5240 | cellular process |
| GO:0043242 | 0.011 | 2.423 | 8 | 14 | 34 | negative regulation of protein complex disassembly |
| GO:0000436 | 0.011 | Inf | 1 | 3 | 3 | carbon catabolite activation of transcription from RNA polymerase II promoter |
| GO:0002175 | 0.011 | Inf | 1 | 3 | 3 | protein localization to paranode region of axon |
| GO:0006122 | 0.011 | Inf | 1 | 3 | 3 | mitochondrial electron transport, ubiquinol to cytochrome c |
| GO:0007412 | 0.011 | Inf | 1 | 3 | 3 | axon target recognition |
| GO:0010586 | 0.011 | Inf | 1 | 3 | 3 | miRNA metabolic process |
| GO:0019230 | 0.011 | Inf | 1 | 3 | 3 | proprioception |
| GO:0021800 | 0.011 | Inf | 1 | 3 | 3 | cerebral cortex tangential migration |
| GO:0031848 | 0.011 | Inf | 1 | 3 | 3 | protection from non-homologous end joining at telomere |
| GO:0032206 | 0.011 | Inf | 1 | 3 | 3 | positive regulation of telomere maintenance |
| GO:0035385 | 0.011 | Inf | 1 | 3 | 3 | Roundabout signaling pathway |
| GO:0071625 | 0.011 | Inf | 1 | 3 | 3 | vocalization behavior |
| GO:0072383 | 0.011 | Inf | 1 | 3 | 3 | plus-end-directed vesicle transport along microtubule |
| GO:0097114 | 0.011 | Inf | 1 | 3 | 3 | N-methyl-D-aspartate receptor clustering |
| GO:0016339 | 0.012 | 5.182 | 2 | 6 | 10 | calcium-dependent cell-cell adhesion |
| GO:0043267 | 0.012 | 5.182 | 2 | 6 | 10 | negative regulation of potassium ion transport |
| GO:0046856 | 0.012 | 5.182 | 2 | 6 | 10 | phosphatidylinositol dephosphorylation |
| GO:0022900 | 0.012 | 1.708 | 21 | 31 | 94 | electron transport chain |
| GO:0009889 | 0.013 | 1.149 | 444 | 480 | 1972 | regulation of biosynthetic process |
| GO:0044087 | 0.013 | 1.383 | 59 | 75 | 264 | regulation of cellular component biogenesis |
| GO:0035249 | 0.013 | 4.045 | 3 | 7 | 13 | synaptic transmission, glutamatergic |
| GO:0060384 | 0.014 | 4.031 | 3 | 7 | 13 | innervation |
| GO:0048489 | 0.014 | 2.902 | 5 | 10 | 22 | synaptic vesicle transport |
| GO:0009653 | 0.014 | 1.166 | 314 | 346 | 1406 | anatomical structure morphogenesis |
| GO:0021772 | 0.015 | 3.111 | 4 | 9 | 19 | olfactory bulb development |
| GO:0006479 | 0.015 | 2.102 | 10 | 17 | 45 | protein methylation |
| GO:0007275 | 0.017 | 1.130 | 594 | 632 | 2641 | multicellular organismal development |
| GO:0055002 | 0.017 | 1.655 | 22 | 31 | 96 | striated muscle cell development |
| GO:0060218 | 0.017 | 5.755 | 2 | 5 | 8 | hematopoietic stem cell differentiation |
| GO:0070989 | 0.017 | 5.755 | 2 | 5 | 8 | oxidative demethylation |
| GO:0023061 | 0.018 | 1.389 | 52 | 66 | 232 | signal release |
| GO:0030111 | 0.018 | 1.566 | 27 | 37 | 119 | regulation of Wnt receptor signaling pathway |
| GO:0045926 | 0.019 | 1.499 | 33 | 44 | 146 | negative regulation of growth |
| GO:0000086 | 0.020 | 1.577 | 25 | 35 | 112 | G2/M transition of mitotic cell cycle |
| GO:0051494 | 0.020 | 1.872 | 13 | 20 | 57 | negative regulation of cytoskeleton organization |
| GO:0006397 | 0.021 | 1.355 | 58 | 72 | 257 | mRNA processing |
| GO:0007026 | 0.021 | 4.145 | 2 | 6 | 11 | negative regulation of microtubule depolymerization |
| GO:0008089 | 0.021 | 4.145 | 2 | 6 | 11 | anterograde axon cargo transport |
| GO:0021591 | 0.021 | 4.145 | 2 | 6 | 11 | ventricular system development |
| GO:0046174 | 0.021 | 4.145 | 2 | 6 | 11 | polyol catabolic process |
| GO:0071705 | 0.021 | 1.279 | 90 | 107 | 399 | nitrogen compound transport |
| GO:0006661 | 0.021 | 1.825 | 14 | 21 | 61 | phosphatidylinositol biosynthetic process |
| GO:0045931 | 0.021 | 2.248 | 7 | 13 | 33 | positive regulation of mitotic cell cycle |
| GO:0051647 | 0.022 | 3.455 | 3 | 7 | 14 | nucleus localization |
| GO:0055003 | 0.022 | 3.455 | 3 | 7 | 14 | cardiac myofibril assembly |
| GO:0060216 | 0.022 | 3.455 | 3 | 7 | 14 | definitive hemopoiesis |
| GO:0051641 | 0.022 | 1.153 | 314 | 343 | 1395 | cellular localization |
| GO:0007417 | 0.023 | 1.550 | 25 | 35 | 115 | central nervous system development |
| GO:0007010 | 0.023 | 1.253 | 104 | 122 | 465 | cytoskeleton organization |
| GO:0016569 | 0.023 | 1.417 | 41 | 53 | 183 | covalent chromatin modification |
| GO:0000209 | 0.024 | 1.529 | 27 | 37 | 121 | protein polyubiquitination |
| GO:0008366 | 0.024 | 1.699 | 17 | 25 | 76 | axon ensheathment |
| GO:0008654 | 0.025 | 1.493 | 30 | 40 | 133 | phospholipid biosynthetic process |
| GO:1901879 | 0.025 | 2.106 | 8 | 14 | 37 | regulation of protein depolymerization |
| GO:0045839 | 0.026 | 2.377 | 6 | 11 | 27 | negative regulation of mitosis |
| GO:0010975 | 0.026 | 1.681 | 17 | 25 | 77 | regulation of neuron projection development |
| GO:0006895 | 0.026 | 6.904 | 1 | 4 | 6 | Golgi to endosome transport |
| GO:0007097 | 0.026 | 6.904 | 1 | 4 | 6 | nuclear migration |
| GO:0007171 | 0.026 | 6.904 | 1 | 4 | 6 | activation of transmembrane receptor protein tyrosine kinase activity |
| GO:0016322 | 0.026 | 6.904 | 1 | 4 | 6 | neuron remodeling |
| GO:0032515 | 0.026 | 6.904 | 1 | 4 | 6 | negative regulation of phosphoprotein phosphatase activity |
| GO:0035970 | 0.026 | 6.904 | 1 | 4 | 6 | peptidyl-threonine dephosphorylation |
| GO:0042428 | 0.026 | 6.904 | 1 | 4 | 6 | serotonin metabolic process |
| GO:0043587 | 0.026 | 6.904 | 1 | 4 | 6 | tongue morphogenesis |
| GO:0044091 | 0.026 | 6.904 | 1 | 4 | 6 | membrane biogenesis |
| GO:0045110 | 0.026 | 6.904 | 1 | 4 | 6 | intermediate filament bundle assembly |
| GO:0045647 | 0.026 | 6.904 | 1 | 4 | 6 | negative regulation of erythrocyte differentiation |
| GO:0060235 | 0.026 | 6.904 | 1 | 4 | 6 | lens induction in camera-type eye |
| GO:0061072 | 0.026 | 6.904 | 1 | 4 | 6 | iris morphogenesis |
| GO:0070933 | 0.026 | 6.904 | 1 | 4 | 6 | histone H4 deacetylation |
| GO:0090129 | 0.026 | 6.904 | 1 | 4 | 6 | positive regulation of synapse maturation |
| GO:0046486 | 0.026 | 2.163 | 8 | 13 | 34 | glycerolipid metabolic process |
| GO:0031056 | 0.028 | 1.827 | 12 | 19 | 55 | regulation of histone modification |
| GO:0000724 | 0.028 | 2.141 | 8 | 13 | 34 | double-strand break repair via homologous recombination |
| GO:0090263 | 0.028 | 2.141 | 8 | 13 | 34 | positive regulation of canonical Wnt receptor signaling pathway |
| GO:0051966 | 0.028 | 2.469 | 5 | 10 | 24 | regulation of synaptic transmission, glutamatergic |
| GO:0019886 | 0.028 | 1.732 | 15 | 22 | 66 | antigen processing and presentation of exogenous peptide antigen via MHC class II |
| GO:0050773 | 0.028 | 1.898 | 11 | 17 | 48 | regulation of dendrite development |
| GO:0007286 | 0.028 | 1.996 | 9 | 15 | 41 | spermatid development |
| GO:0032259 | 0.030 | 1.695 | 16 | 23 | 70 | methylation |
| GO:0050657 | 0.030 | 1.613 | 19 | 27 | 85 | nucleic acid transport |
| GO:0051236 | 0.030 | 1.613 | 19 | 27 | 85 | establishment of RNA localization |
| GO:0016358 | 0.031 | 2.792 | 4 | 8 | 18 | dendrite development |
| GO:0072659 | 0.031 | 1.832 | 12 | 18 | 52 | protein localization to plasma membrane |
| GO:0007612 | 0.031 | 1.663 | 17 | 24 | 74 | learning |
| GO:0002040 | 0.032 | 2.018 | 9 | 14 | 38 | sprouting angiogenesis |
| GO:0050803 | 0.032 | 2.018 | 9 | 14 | 38 | regulation of synapse structure and activity |
| GO:0010544 | 0.032 | 4.316 | 2 | 5 | 9 | negative regulation of platelet activation |
| GO:0042572 | 0.032 | 4.316 | 2 | 5 | 9 | retinol metabolic process |
| GO:0048935 | 0.032 | 4.316 | 2 | 5 | 9 | peripheral nervous system neuron development |
| GO:0051642 | 0.032 | 4.316 | 2 | 5 | 9 | centrosome localization |
| GO:0060004 | 0.032 | 4.316 | 2 | 5 | 9 | reflex |
| GO:2000310 | 0.032 | 4.316 | 2 | 5 | 9 | regulation of N-methyl-D-aspartate selective glutamate receptor activity |
| GO:0040036 | 0.032 | 2.764 | 4 | 8 | 18 | regulation of fibroblast growth factor receptor signaling pathway |
| GO:0042771 | 0.032 | 2.764 | 4 | 8 | 18 | intrinsic apoptotic signaling pathway in response to DNA damage by p53 class mediator |
| GO:0030072 | 0.032 | 1.452 | 31 | 41 | 139 | peptide hormone secretion |
| GO:0042551 | 0.033 | 3.028 | 3 | 7 | 15 | neuron maturation |
| GO:0001578 | 0.033 | 3.022 | 3 | 7 | 15 | microtubule bundle formation |
| GO:0042491 | 0.033 | 3.022 | 3 | 7 | 15 | auditory receptor cell differentiation |
| GO:0002504 | 0.033 | 1.693 | 15 | 22 | 67 | antigen processing and presentation of peptide or polysaccharide antigen via MHC class II |
| GO:0051965 | 0.034 | 3.453 | 3 | 6 | 12 | positive regulation of synapse assembly |
| GO:0048666 | 0.034 | 1.429 | 33 | 43 | 151 | neuron development |
| GO:0014047 | 0.034 | 2.237 | 6 | 11 | 28 | glutamate secretion |
| GO:0032387 | 0.035 | 1.731 | 13 | 20 | 60 | negative regulation of intracellular transport |
| GO:0021537 | 0.036 | 1.492 | 25 | 34 | 113 | telencephalon development |
| GO:0051603 | 0.037 | 1.302 | 60 | 73 | 268 | proteolysis involved in cellular protein catabolic process |
| GO:0090090 | 0.037 | 1.780 | 12 | 18 | 53 | negative regulation of canonical Wnt receptor signaling pathway |
| GO:0006726 | 0.038 | 10.351 | 1 | 3 | 4 | eye pigment biosynthetic process |
| GO:0006983 | 0.038 | 10.351 | 1 | 3 | 4 | ER overload response |
| GO:0007021 | 0.038 | 10.351 | 1 | 3 | 4 | tubulin complex assembly |
| GO:0007549 | 0.038 | 10.351 | 1 | 3 | 4 | dosage compensation |
| GO:0008298 | 0.038 | 10.351 | 1 | 3 | 4 | intracellular mRNA localization |
| GO:0016082 | 0.038 | 10.351 | 1 | 3 | 4 | synaptic vesicle priming |
| GO:0018027 | 0.038 | 10.351 | 1 | 3 | 4 | peptidyl-lysine dimethylation |
| GO:0019896 | 0.038 | 10.351 | 1 | 3 | 4 | axon transport of mitochondrion |
| GO:0021707 | 0.038 | 10.351 | 1 | 3 | 4 | cerebellar granule cell differentiation |
| GO:0021891 | 0.038 | 10.351 | 1 | 3 | 4 | olfactory bulb interneuron development |
| GO:0032211 | 0.038 | 10.351 | 1 | 3 | 4 | negative regulation of telomere maintenance via telomerase |
| GO:0032847 | 0.038 | 10.351 | 1 | 3 | 4 | regulation of cellular pH reduction |
| GO:0034088 | 0.038 | 10.351 | 1 | 3 | 4 | maintenance of mitotic sister chromatid cohesion |
| GO:0038030 | 0.038 | 10.351 | 1 | 3 | 4 | non-canonical Wnt receptor signaling pathway via MAPK cascade |
| GO:0043324 | 0.038 | 10.351 | 1 | 3 | 4 | pigment metabolic process involved in developmental pigmentation |
| GO:0043983 | 0.038 | 10.351 | 1 | 3 | 4 | histone H4-K12 acetylation |
| GO:0045634 | 0.038 | 10.351 | 1 | 3 | 4 | regulation of melanocyte differentiation |
| GO:0046015 | 0.038 | 10.351 | 1 | 3 | 4 | regulation of transcription by glucose |
| GO:0050957 | 0.038 | 10.351 | 1 | 3 | 4 | equilibrioception |
| GO:0070100 | 0.038 | 10.351 | 1 | 3 | 4 | negative regulation of chemokine-mediated signaling pathway |
| GO:0071205 | 0.038 | 10.351 | 1 | 3 | 4 | protein localization to juxtaparanode region of axon |
| GO:0071499 | 0.038 | 10.351 | 1 | 3 | 4 | cellular response to laminar fluid shear stress |
| GO:0072393 | 0.038 | 10.351 | 1 | 3 | 4 | microtubule anchoring at microtubule organizing center |
| GO:0097105 | 0.038 | 10.351 | 1 | 3 | 4 | presynaptic membrane assembly |
| GO:0097119 | 0.038 | 10.351 | 1 | 3 | 4 | postsynaptic density protein 95 clustering |
| GO:2000121 | 0.038 | 10.351 | 1 | 3 | 4 | regulation of removal of superoxide radicals |
| GO:2001214 | 0.038 | 10.351 | 1 | 3 | 4 | positive regulation of vasculogenesis |
| GO:0048167 | 0.038 | 1.603 | 18 | 25 | 79 | regulation of synaptic plasticity |
| GO:0002791 | 0.038 | 1.458 | 28 | 37 | 125 | regulation of peptide secretion |
| GO:0018205 | 0.039 | 1.513 | 23 | 31 | 102 | peptidyl-lysine modification |
| GO:0050772 | 0.039 | 2.074 | 7 | 12 | 32 | positive regulation of axonogenesis |
| GO:0021766 | 0.040 | 1.937 | 9 | 14 | 39 | hippocampus development |
| GO:0048839 | 0.040 | 1.499 | 24 | 32 | 106 | inner ear development |
| GO:0000377 | 0.040 | 1.407 | 34 | 43 | 149 | RNA splicing, via transesterification reactions with bulged adenosine as nucleophile |
| GO:0045333 | 0.040 | 1.486 | 25 | 33 | 110 | cellular respiration |
| GO:0042471 | 0.041 | 1.625 | 16 | 23 | 72 | ear morphogenesis |
| GO:0001954 | 0.041 | 2.392 | 5 | 9 | 22 | positive regulation of cell-matrix adhesion |
| GO:0032845 | 0.041 | 2.392 | 5 | 9 | 22 | negative regulation of homeostatic process |
| GO:0045104 | 0.041 | 2.392 | 5 | 9 | 22 | intermediate filament cytoskeleton organization |
| GO:0050796 | 0.041 | 1.473 | 26 | 34 | 114 | regulation of insulin secretion |
| GO:0000087 | 0.041 | 1.322 | 51 | 62 | 225 | M phase of mitotic cell cycle |
| GO:0021987 | 0.042 | 1.782 | 11 | 17 | 50 | cerebral cortex development |
| GO:0051028 | 0.042 | 1.598 | 17 | 24 | 76 | mRNA transport |
| GO:0007517 | 0.043 | 1.294 | 58 | 70 | 258 | muscle organ development |
| GO:0051147 | 0.044 | 1.574 | 18 | 25 | 80 | regulation of muscle cell differentiation |
| GO:0045893 | 0.044 | 1.172 | 163 | 182 | 725 | positive regulation of transcription, DNA-dependent |
| GO:0001505 | 0.044 | 1.492 | 23 | 31 | 103 | regulation of neurotransmitter levels |
| GO:0044260 | 0.044 | 1.132 | 337 | 361 | 1545 | cellular macromolecule metabolic process |
| GO:0006818 | 0.044 | 1.730 | 12 | 18 | 54 | hydrogen transport |
| GO:0045935 | 0.044 | 1.159 | 194 | 214 | 861 | positive regulation of nucleobase-containing compound metabolic process |
| GO:0000718 | 0.045 | 2.512 | 4 | 8 | 19 | nucleotide-excision repair, DNA damage removal |
| GO:0007620 | 0.045 | 2.512 | 4 | 8 | 19 | copulation |
| GO:0045595 | 0.045 | 1.181 | 145 | 163 | 649 | regulation of cell differentiation |
| GO:0042692 | 0.046 | 1.395 | 33 | 42 | 147 | muscle cell differentiation |
| GO:0006334 | 0.047 | 1.785 | 11 | 16 | 47 | nucleosome assembly |
| GO:0048709 | 0.047 | 1.785 | 11 | 16 | 47 | oligodendrocyte differentiation |
| GO:0001764 | 0.047 | 1.592 | 16 | 23 | 73 | neuron migration |
| GO:0008038 | 0.048 | 2.692 | 4 | 7 | 16 | neuron recognition |
| GO:0034453 | 0.048 | 2.690 | 4 | 7 | 16 | microtubule anchoring |
| GO:0031113 | 0.048 | 2.686 | 4 | 7 | 16 | regulation of microtubule polymerization |
| GO:0046854 | 0.048 | 2.686 | 4 | 7 | 16 | phosphatidylinositol phosphorylation |
| GO:2000209 | 0.048 | 2.686 | 4 | 7 | 16 | regulation of anoikis |
| GO:0010769 | 0.049 | 1.401 | 31 | 40 | 139 | regulation of cell morphogenesis involved in differentiation |
| GO:0090199 | 0.049 | 2.160 | 6 | 10 | 26 | regulation of release of cytochrome c from mitochondria |
| GO:0000280 | 0.049 | 1.311 | 49 | 60 | 219 | nuclear division |
| GO:0060788 | 0.049 | 4.611 | 2 | 4 | 7 | ectodermal placode formation |
| GO:0007004 | 0.049 | 4.609 | 2 | 4 | 7 | telomere maintenance via telomerase |
| GO:0006476 | 0.050 | 1.975 | 7 | 12 | 33 | protein deacetylation |
| GO:0006349 | 0.050 | 4.602 | 2 | 4 | 7 | regulation of gene expression by genetic imprinting |
| GO:0014866 | 0.050 | 4.602 | 2 | 4 | 7 | skeletal myofibril assembly |
| GO:0018023 | 0.050 | 4.602 | 2 | 4 | 7 | peptidyl-lysine trimethylation |
| GO:0019934 | 0.050 | 4.602 | 2 | 4 | 7 | cGMP-mediated signaling |
| GO:0021535 | 0.050 | 4.602 | 2 | 4 | 7 | cell migration in hindbrain |
| GO:0032008 | 0.050 | 4.602 | 2 | 4 | 7 | positive regulation of TOR signaling cascade |
| GO:0033689 | 0.050 | 4.602 | 2 | 4 | 7 | negative regulation of osteoblast proliferation |
| GO:0046548 | 0.050 | 4.602 | 2 | 4 | 7 | retinal rod cell development |
| GO:0048703 | 0.050 | 4.602 | 2 | 4 | 7 | embryonic viscerocranium morphogenesis |
| GO:0051823 | 0.050 | 4.602 | 2 | 4 | 7 | regulation of synapse structural plasticity |
| GO:0060073 | 0.050 | 4.602 | 2 | 4 | 7 | micturition |
| GO:0070536 | 0.050 | 4.602 | 2 | 4 | 7 | protein K63-linked deubiquitination |
| GO:0071108 | 0.050 | 4.602 | 2 | 4 | 7 | protein K48-linked deubiquitination |
| GO:0071425 | 0.050 | 4.602 | 2 | 4 | 7 | hematopoietic stem cell proliferation |
| GO:0072239 | 0.050 | 4.602 | 2 | 4 | 7 | metanephric glomerulus vasculature development |
| GO:2000463 | 0.050 | 4.602 | 2 | 4 | 7 | positive regulation of excitatory postsynaptic membrane potential |
