# Supplementary material for: Hidden Treasures in “Ancient” Microarrays: Gene-Expression Portrays Biology and Potential Resistance Pathways of Major Lung Cancer Subtypes and Normal Tissue
Source: Front Oncol. 2014 Sep 29;4:251. doi: 10.3389/fonc.2014.00251 (PMC4178426; doi:10.3389/fonc.2014.00251)
Supplement: Figure S1 — Clinically relevant differentially expressed pathways in each tissue type versus the rest. The pathway maps are arranged according to the scheme in the left-top corner. Red indicates overexpression in genes, dark green downregulation. In the Cell Cycle pathway, tumor suppressor genes were overexpressed in normal lung tissue while oncogenes and tumor driving cyclins were overexpressed in cancers. The gene thymidylate synthase (TYMS or TS, belonging to the pathway “one carbon pool by folate”) is relevant for tumor growth and is also a treatment target. Notably, TYMS was overexpressed only in the tumors that are generally refractory to the drug pemetrexed, as the squamous and the small-cell lung cancer. TYMS was not overexpressed in mesothelioma, but it is known that TYMS expression is highly variable in this cancer. In the ERBB pathway, the ERBB2/HER2 and ERBB3/HER3 were overexpressed in adenocarcinoma, while the ERBB4 was overexpressed in the carcinoids. [file Data_Sheet_1.ZIP › File S3.ZIP!/GO Analysis/Adenocarcinomas vs Rest Enrichment Analysis/Overrepresented GO terms of Up-regulated Genes.html]

Gene to GO BP Conditional test for over-representation

| GOBPID | Pvalue | OddsRatio | ExpCount | Count | Size | Term |
| --- | --- | --- | --- | --- | --- | --- |
| GO:0006614 | <0.0001 | 5.245 | 14 | 40 | 75 | SRP-dependent cotranslational protein targeting to membrane |
| GO:0072599 | <0.0001 | 4.829 | 14 | 40 | 78 | establishment of protein localization to endoplasmic reticulum |
| GO:0006414 | <0.0001 | 4.707 | 13 | 36 | 71 | translational elongation |
| GO:0043687 | <0.0001 | 3.482 | 20 | 47 | 109 | post-translational protein modification |
| GO:0006415 | <0.0001 | 4.864 | 12 | 33 | 64 | translational termination |
| GO:0018279 | <0.0001 | 5.040 | 11 | 32 | 61 | protein N-linked glycosylation via asparagine |
| GO:0006612 | <0.0001 | 3.101 | 20 | 44 | 109 | protein targeting to membrane |
| GO:0060333 | <0.0001 | 3.862 | 11 | 28 | 61 | interferon-gamma-mediated signaling pathway |
| GO:0000184 | <0.0001 | 3.100 | 15 | 34 | 84 | nuclear-transcribed mRNA catabolic process, nonsense-mediated decay |
| GO:0009100 | <0.0001 | 2.304 | 29 | 54 | 162 | glycoprotein metabolic process |
| GO:0034341 | <0.0001 | 2.955 | 16 | 35 | 89 | response to interferon-gamma |
| GO:0034340 | <0.0001 | 3.611 | 11 | 27 | 61 | response to type I interferon |
| GO:0060337 | <0.0001 | 3.611 | 11 | 27 | 61 | type I interferon-mediated signaling pathway |
| GO:0006486 | <0.0001 | 2.385 | 27 | 50 | 146 | protein glycosylation |
| GO:0070085 | <0.0001 | 2.360 | 27 | 50 | 147 | glycosylation |
| GO:0006886 | <0.0001 | 1.643 | 91 | 130 | 498 | intracellular protein transport |
| GO:0002474 | <0.0001 | 12.559 | 3 | 11 | 15 | antigen processing and presentation of peptide antigen via MHC class I |
| GO:0050776 | <0.0001 | 2.054 | 36 | 62 | 203 | regulation of immune response |
| GO:0019083 | <0.0001 | 2.427 | 22 | 42 | 121 | viral transcription |
| GO:0019884 | <0.0001 | 2.336 | 24 | 45 | 133 | antigen processing and presentation of exogenous antigen |
| GO:0043241 | <0.0001 | 2.466 | 21 | 40 | 114 | protein complex disassembly |
| GO:0048199 | <0.0001 | 7.746 | 3 | 12 | 19 | vesicle targeting, to, from or within Golgi |
| GO:0006413 | <0.0001 | 2.402 | 21 | 39 | 113 | translational initiation |
| GO:0046394 | <0.0001 | 1.991 | 34 | 57 | 188 | carboxylic acid biosynthetic process |
| GO:0002460 | <0.0001 | 2.260 | 23 | 42 | 127 | adaptive immune response based on somatic recombination of immune receptors built from immunoglobulin superfamily domains |
| GO:0006987 | <0.0001 | 3.217 | 10 | 22 | 53 | activation of signaling protein activity involved in unfolded protein response |
| GO:0006955 | <0.0001 | 1.890 | 37 | 59 | 211 | immune response |
| GO:0045184 | <0.0001 | 1.408 | 152 | 193 | 833 | establishment of protein localization |
| GO:0002253 | <0.0001 | 1.842 | 39 | 62 | 216 | activation of immune response |
| GO:0006488 | <0.0001 | 7.522 | 3 | 10 | 16 | dolichol-linked oligosaccharide biosynthetic process |
| GO:0002449 | <0.0001 | 2.143 | 23 | 40 | 125 | lymphocyte mediated immunity |
| GO:0033365 | <0.0001 | 1.595 | 67 | 94 | 365 | protein localization to organelle |
| GO:0034976 | <0.0001 | 2.433 | 16 | 30 | 86 | response to endoplasmic reticulum stress |
| GO:0032069 | <0.0001 | 2.936 | 10 | 22 | 56 | regulation of nuclease activity |
| GO:0002252 | <0.0001 | 1.610 | 63 | 89 | 343 | immune effector process |
| GO:0016482 | <0.0001 | 1.513 | 85 | 115 | 468 | cytoplasmic transport |
| GO:0043123 | <0.0001 | 2.271 | 18 | 32 | 96 | positive regulation of I-kappaB kinase/NF-kappaB cascade |
| GO:0002684 | <0.0001 | 1.794 | 36 | 56 | 201 | positive regulation of immune system process |
| GO:0005975 | <0.0001 | 1.538 | 70 | 97 | 390 | carbohydrate metabolic process |
| GO:0051650 | <0.0001 | 3.445 | 7 | 16 | 37 | establishment of vesicle localization |
| GO:0022411 | <0.0001 | 1.720 | 41 | 61 | 223 | cellular component disassembly |
| GO:0002504 | <0.0001 | 2.529 | 12 | 24 | 67 | antigen processing and presentation of peptide or polysaccharide antigen via MHC class II |
| GO:0034620 | <0.0001 | 2.529 | 12 | 24 | 67 | cellular response to unfolded protein |
| GO:0006950 | 0.001 | 1.246 | 357 | 406 | 1974 | response to stress |
| GO:0070727 | 0.001 | 1.387 | 121 | 153 | 663 | cellular macromolecule localization |
| GO:0019058 | 0.001 | 1.788 | 33 | 51 | 181 | viral infectious cycle |
| GO:0006984 | 0.001 | 2.357 | 14 | 26 | 76 | ER-nucleus signaling pathway |
| GO:0006402 | 0.001 | 2.002 | 22 | 37 | 121 | mRNA catabolic process |
| GO:0009084 | 0.001 | 7.212 | 2 | 8 | 13 | glutamine family amino acid biosynthetic process |
| GO:0043436 | 0.001 | 1.395 | 114 | 145 | 625 | oxoacid metabolic process |
| GO:0016064 | 0.001 | 2.360 | 13 | 25 | 73 | immunoglobulin mediated immune response |
| GO:0019886 | 0.001 | 2.422 | 12 | 23 | 66 | antigen processing and presentation of exogenous peptide antigen via MHC class II |
| GO:0042345 | 0.001 | 3.869 | 5 | 12 | 26 | regulation of NF-kappaB import into nucleus |
| GO:0048208 | 0.001 | 7.884 | 2 | 7 | 11 | COPII vesicle coating |
| GO:0060666 | 0.001 | Inf | 1 | 4 | 4 | dichotomous subdivision of terminal units involved in salivary gland branching |
| GO:0009607 | 0.001 | 1.454 | 78 | 103 | 428 | response to biotic stimulus |
| GO:0048584 | 0.001 | 1.321 | 155 | 189 | 852 | positive regulation of response to stimulus |
| GO:0002764 | 0.001 | 1.720 | 34 | 51 | 186 | immune response-regulating signaling pathway |
| GO:0044764 | 0.001 | 1.400 | 98 | 126 | 540 | multi-organism cellular process |
| GO:0071702 | 0.001 | 1.265 | 231 | 270 | 1267 | organic substance transport |
| GO:0030198 | 0.001 | 1.898 | 23 | 37 | 126 | extracellular matrix organization |
| GO:0071345 | 0.001 | 1.512 | 59 | 81 | 326 | cellular response to cytokine stimulus |
| GO:0002479 | 0.001 | 2.371 | 12 | 22 | 64 | antigen processing and presentation of exogenous peptide antigen via MHC class I, TAP-dependent |
| GO:0035966 | 0.002 | 1.988 | 19 | 32 | 105 | response to topologically incorrect protein |
| GO:1901607 | 0.002 | 3.161 | 6 | 14 | 34 | alpha-amino acid biosynthetic process |
| GO:0045723 | 0.002 | 9.005 | 2 | 6 | 9 | positive regulation of fatty acid biosynthetic process |
| GO:0048205 | 0.002 | 9.005 | 2 | 6 | 9 | COPI coating of Golgi vesicle |
| GO:0042993 | 0.002 | 4.100 | 4 | 10 | 21 | positive regulation of transcription factor import into nucleus |
| GO:0002687 | 0.002 | 2.380 | 11 | 20 | 58 | positive regulation of leukocyte migration |
| GO:0006767 | 0.002 | 2.823 | 7 | 15 | 39 | water-soluble vitamin metabolic process |
| GO:0051289 | 0.002 | 2.823 | 7 | 15 | 39 | protein homotetramerization |
| GO:0016052 | 0.003 | 1.925 | 19 | 31 | 104 | carbohydrate catabolic process |
| GO:0019882 | 0.003 | 3.829 | 4 | 10 | 22 | antigen processing and presentation |
| GO:0006412 | 0.003 | 1.749 | 26 | 40 | 146 | translation |
| GO:0009225 | 0.003 | 3.757 | 4 | 10 | 22 | nucleotide-sugar metabolic process |
| GO:0002480 | 0.003 | 11.250 | 1 | 5 | 7 | antigen processing and presentation of exogenous peptide antigen via MHC class I, TAP-independent |
| GO:0002293 | 0.003 | 3.185 | 5 | 12 | 29 | alpha-beta T cell differentiation involved in immune response |
| GO:0002286 | 0.003 | 2.709 | 7 | 15 | 40 | T cell activation involved in immune response |
| GO:0002526 | 0.004 | 1.972 | 16 | 27 | 89 | acute inflammatory response |
| GO:0030449 | 0.004 | 4.505 | 3 | 8 | 16 | regulation of complement activation |
| GO:0048194 | 0.004 | 6.753 | 2 | 6 | 10 | Golgi vesicle budding |
| GO:0022614 | 0.005 | 17.990 | 1 | 4 | 5 | membrane to membrane docking |
| GO:0042161 | 0.005 | 17.990 | 1 | 4 | 5 | lipoprotein oxidation |
| GO:0072540 | 0.005 | 17.990 | 1 | 4 | 5 | T-helper 17 cell lineage commitment |
| GO:1901137 | 0.005 | 1.384 | 75 | 96 | 415 | carbohydrate derivative biosynthetic process |
| GO:0006958 | 0.005 | 3.100 | 5 | 11 | 27 | complement activation, classical pathway |
| GO:0030199 | 0.005 | 3.100 | 5 | 11 | 27 | collagen fibril organization |
| GO:0002429 | 0.006 | 1.867 | 17 | 28 | 96 | immune response-activating cell surface receptor signaling pathway |
| GO:0006041 | 0.006 | Inf | 1 | 3 | 3 | glucosamine metabolic process |
| GO:0008588 | 0.006 | Inf | 1 | 3 | 3 | release of cytoplasmic sequestered NF-kappaB |
| GO:0018401 | 0.006 | Inf | 1 | 3 | 3 | peptidyl-proline hydroxylation to 4-hydroxy-L-proline |
| GO:0071420 | 0.006 | Inf | 1 | 3 | 3 | cellular response to histamine |
| GO:0043367 | 0.006 | 2.666 | 6 | 13 | 35 | CD4-positive, alpha-beta T cell differentiation |
| GO:0001775 | 0.007 | 1.424 | 57 | 75 | 318 | cell activation |
| GO:0030157 | 0.007 | 7.499 | 1 | 5 | 8 | pancreatic juice secretion |
| GO:0045059 | 0.007 | 7.499 | 1 | 5 | 8 | positive thymic T cell selection |
| GO:0046348 | 0.007 | 7.499 | 1 | 5 | 8 | amino sugar catabolic process |
| GO:0072538 | 0.007 | 7.499 | 1 | 5 | 8 | T-helper 17 type immune response |
| GO:0045087 | 0.007 | 1.459 | 49 | 65 | 272 | innate immune response |
| GO:0043124 | 0.008 | 3.379 | 4 | 9 | 21 | negative regulation of I-kappaB kinase/NF-kappaB cascade |
| GO:0006888 | 0.008 | 2.549 | 7 | 13 | 36 | ER to Golgi vesicle-mediated transport |
| GO:0002366 | 0.010 | 1.750 | 19 | 29 | 104 | leukocyte activation involved in immune response |
| GO:0031663 | 0.010 | 2.576 | 6 | 12 | 33 | lipopolysaccharide-mediated signaling pathway |
| GO:0002688 | 0.011 | 2.132 | 10 | 17 | 53 | regulation of leukocyte chemotaxis |
| GO:0006900 | 0.011 | 3.130 | 4 | 9 | 22 | membrane budding |
| GO:0006027 | 0.011 | 2.339 | 7 | 14 | 41 | glycosaminoglycan catabolic process |
| GO:0016266 | 0.011 | 3.119 | 4 | 9 | 22 | O-glycan processing |
| GO:0032526 | 0.011 | 1.936 | 13 | 21 | 70 | response to retinoic acid |
| GO:0032943 | 0.012 | 1.594 | 27 | 38 | 146 | mononuclear cell proliferation |
| GO:0045580 | 0.012 | 1.964 | 12 | 20 | 66 | regulation of T cell differentiation |
| GO:0006002 | 0.012 | 8.994 | 1 | 4 | 6 | fructose 6-phosphate metabolic process |
| GO:0010873 | 0.012 | 8.994 | 1 | 4 | 6 | positive regulation of cholesterol esterification |
| GO:0019883 | 0.012 | 8.994 | 1 | 4 | 6 | antigen processing and presentation of endogenous antigen |
| GO:0043097 | 0.012 | 8.994 | 1 | 4 | 6 | pyrimidine nucleoside salvage |
| GO:0001916 | 0.012 | 4.500 | 2 | 6 | 12 | positive regulation of T cell mediated cytotoxicity |
| GO:0006760 | 0.012 | 4.500 | 2 | 6 | 12 | folic acid-containing compound metabolic process |
| GO:0046135 | 0.012 | 4.500 | 2 | 6 | 12 | pyrimidine nucleoside catabolic process |
| GO:0006508 | 0.013 | 1.277 | 106 | 127 | 582 | proteolysis |
| GO:0042093 | 0.013 | 5.640 | 2 | 5 | 9 | T-helper cell differentiation |
| GO:0045454 | 0.013 | 2.609 | 5 | 11 | 30 | cell redox homeostasis |
| GO:0002230 | 0.013 | 5.623 | 2 | 5 | 9 | positive regulation of defense response to virus by host |
| GO:0006662 | 0.013 | 5.623 | 2 | 5 | 9 | glycerol ether metabolic process |
| GO:2000401 | 0.013 | 3.275 | 3 | 8 | 19 | regulation of lymphocyte migration |
| GO:0051249 | 0.014 | 1.450 | 41 | 54 | 223 | regulation of lymphocyte activation |
| GO:0051186 | 0.015 | 1.582 | 25 | 36 | 139 | cofactor metabolic process |
| GO:0032760 | 0.015 | 2.896 | 4 | 9 | 23 | positive regulation of tumor necrosis factor production |
| GO:0071230 | 0.015 | 2.896 | 4 | 9 | 23 | cellular response to amino acid stimulus |
| GO:0010627 | 0.016 | 1.280 | 94 | 113 | 516 | regulation of intracellular protein kinase cascade |
| GO:0042994 | 0.016 | 3.501 | 3 | 7 | 16 | cytoplasmic sequestering of transcription factor |
| GO:0050727 | 0.018 | 1.720 | 17 | 25 | 91 | regulation of inflammatory response |
| GO:0002696 | 0.018 | 1.478 | 33 | 45 | 183 | positive regulation of leukocyte activation |
| GO:0046649 | 0.019 | 1.556 | 25 | 35 | 138 | lymphocyte activation |
| GO:0009615 | 0.019 | 1.451 | 36 | 48 | 198 | response to virus |
| GO:0003084 | 0.019 | 3.857 | 2 | 6 | 13 | positive regulation of systemic arterial blood pressure |
| GO:0015949 | 0.019 | 3.857 | 2 | 6 | 13 | nucleobase-containing small molecule interconversion |
| GO:0022415 | 0.021 | 1.384 | 47 | 60 | 260 | viral reproductive process |
| GO:0000462 | 0.021 | 13.483 | 1 | 3 | 4 | maturation of SSU-rRNA from tricistronic rRNA transcript (SSU-rRNA, 5.8S rRNA, LSU-rRNA) |
| GO:0003183 | 0.021 | 13.483 | 1 | 3 | 4 | mitral valve morphogenesis |
| GO:0006048 | 0.021 | 13.483 | 1 | 3 | 4 | UDP-N-acetylglucosamine biosynthetic process |
| GO:0019885 | 0.021 | 13.483 | 1 | 3 | 4 | antigen processing and presentation of endogenous peptide antigen via MHC class I |
| GO:0030579 | 0.021 | 13.483 | 1 | 3 | 4 | ubiquitin-dependent SMAD protein catabolic process |
| GO:0032020 | 0.021 | 13.483 | 1 | 3 | 4 | ISG15-protein conjugation |
| GO:0033239 | 0.021 | 13.483 | 1 | 3 | 4 | negative regulation of cellular amine metabolic process |
| GO:0042762 | 0.021 | 13.483 | 1 | 3 | 4 | regulation of sulfur metabolic process |
| GO:0060591 | 0.021 | 13.483 | 1 | 3 | 4 | chondroblast differentiation |
| GO:2000303 | 0.021 | 13.483 | 1 | 3 | 4 | regulation of ceramide biosynthetic process |
| GO:1901566 | 0.021 | 1.290 | 79 | 96 | 435 | organonitrogen compound biosynthetic process |
| GO:0006520 | 0.021 | 1.355 | 54 | 68 | 296 | cellular amino acid metabolic process |
| GO:0002821 | 0.022 | 2.253 | 7 | 12 | 36 | positive regulation of adaptive immune response |
| GO:0006665 | 0.022 | 1.806 | 13 | 20 | 70 | sphingolipid metabolic process |
| GO:0071526 | 0.022 | 4.498 | 2 | 5 | 10 | semaphorin-plexin signaling pathway |
| GO:0071621 | 0.022 | 4.498 | 2 | 5 | 10 | granulocyte chemotaxis |
| GO:0071219 | 0.023 | 1.745 | 14 | 22 | 79 | cellular response to molecule of bacterial origin |
| GO:0001913 | 0.024 | 6.015 | 1 | 4 | 7 | T cell mediated cytotoxicity |
| GO:0002827 | 0.024 | 5.995 | 1 | 4 | 7 | positive regulation of T-helper 1 type immune response |
| GO:0002921 | 0.024 | 5.995 | 1 | 4 | 7 | negative regulation of humoral immune response |
| GO:0006020 | 0.024 | 5.995 | 1 | 4 | 7 | inositol metabolic process |
| GO:0010759 | 0.024 | 5.995 | 1 | 4 | 7 | positive regulation of macrophage chemotaxis |
| GO:0032727 | 0.024 | 5.995 | 1 | 4 | 7 | positive regulation of interferon-alpha production |
| GO:0044283 | 0.024 | 1.339 | 55 | 69 | 303 | small molecule biosynthetic process |
| GO:0007249 | 0.025 | 2.576 | 4 | 9 | 25 | I-kappaB kinase/NF-kappaB cascade |
| GO:0050921 | 0.026 | 1.770 | 13 | 20 | 71 | positive regulation of chemotaxis |
| GO:0015914 | 0.027 | 2.536 | 5 | 9 | 25 | phospholipid transport |
| GO:0045071 | 0.027 | 2.533 | 5 | 9 | 25 | negative regulation of viral genome replication |
| GO:0072524 | 0.027 | 2.252 | 6 | 11 | 33 | pyridine-containing compound metabolic process |
| GO:0042098 | 0.027 | 1.605 | 19 | 27 | 103 | T cell proliferation |
| GO:0050852 | 0.028 | 1.723 | 14 | 21 | 76 | T cell receptor signaling pathway |
| GO:0001773 | 0.029 | 3.374 | 3 | 6 | 14 | myeloid dendritic cell activation |
| GO:0032728 | 0.029 | 3.374 | 3 | 6 | 14 | positive regulation of interferon-beta production |
| GO:0006633 | 0.029 | 1.742 | 13 | 20 | 72 | fatty acid biosynthetic process |
| GO:0050865 | 0.029 | 1.338 | 51 | 64 | 281 | regulation of cell activation |
| GO:0050795 | 0.030 | 1.560 | 21 | 29 | 113 | regulation of behavior |
| GO:0031668 | 0.031 | 1.613 | 17 | 25 | 95 | cellular response to extracellular stimulus |
| GO:0050870 | 0.032 | 1.492 | 25 | 34 | 137 | positive regulation of T cell activation |
| GO:0072678 | 0.032 | 2.864 | 3 | 7 | 18 | T cell migration |
| GO:0071706 | 0.033 | 1.850 | 10 | 16 | 55 | tumor necrosis factor superfamily cytokine production |
| GO:0042590 | 0.033 | Inf | 0 | 2 | 2 | antigen processing and presentation of exogenous peptide antigen via MHC class I |
| GO:0046365 | 0.033 | Inf | 0 | 2 | 2 | monosaccharide catabolic process |
| GO:0051704 | 0.033 | 1.247 | 88 | 104 | 494 | multi-organism process |
| GO:0030490 | 0.033 | Inf | 0 | 2 | 2 | maturation of SSU-rRNA |
| GO:0001543 | 0.033 | Inf | 0 | 2 | 2 | ovarian follicle rupture |
| GO:0002220 | 0.033 | Inf | 0 | 2 | 2 | innate immune response activating cell surface receptor signaling pathway |
| GO:0002268 | 0.033 | Inf | 0 | 2 | 2 | follicular dendritic cell differentiation |
| GO:0002323 | 0.033 | Inf | 0 | 2 | 2 | natural killer cell activation involved in immune response |
| GO:0002378 | 0.033 | Inf | 0 | 2 | 2 | immunoglobulin biosynthetic process |
| GO:0005999 | 0.033 | Inf | 0 | 2 | 2 | xylulose biosynthetic process |
| GO:0006097 | 0.033 | Inf | 0 | 2 | 2 | glyoxylate cycle |
| GO:0006450 | 0.033 | Inf | 0 | 2 | 2 | regulation of translational fidelity |
| GO:0006517 | 0.033 | Inf | 0 | 2 | 2 | protein deglycosylation |
| GO:0006649 | 0.033 | Inf | 0 | 2 | 2 | phospholipid transfer to membrane |
| GO:0006990 | 0.033 | Inf | 0 | 2 | 2 | positive regulation of transcription from RNA polymerase II promoter involved in unfolded protein response |
| GO:0009440 | 0.033 | Inf | 0 | 2 | 2 | cyanate catabolic process |
| GO:0010909 | 0.033 | Inf | 0 | 2 | 2 | positive regulation of heparan sulfate proteoglycan biosynthetic process |
| GO:0016259 | 0.033 | Inf | 0 | 2 | 2 | selenocysteine metabolic process |
| GO:0019043 | 0.033 | Inf | 0 | 2 | 2 | establishment of viral latency |
| GO:0019264 | 0.033 | Inf | 0 | 2 | 2 | glycine biosynthetic process from serine |
| GO:0019747 | 0.033 | Inf | 0 | 2 | 2 | regulation of isoprenoid metabolic process |
| GO:0035814 | 0.033 | Inf | 0 | 2 | 2 | negative regulation of renal sodium excretion |
| GO:0035993 | 0.033 | Inf | 0 | 2 | 2 | deltoid tuberosity development |
| GO:0036023 | 0.033 | Inf | 0 | 2 | 2 | embryonic skeletal limb joint morphogenesis |
| GO:0038110 | 0.033 | Inf | 0 | 2 | 2 | interleukin-2-mediated signaling pathway |
| GO:0042891 | 0.033 | Inf | 0 | 2 | 2 | antibiotic transport |
| GO:0043628 | 0.033 | Inf | 0 | 2 | 2 | ncRNA 3'-end processing |
| GO:0044209 | 0.033 | Inf | 0 | 2 | 2 | AMP salvage |
| GO:0044334 | 0.033 | Inf | 0 | 2 | 2 | canonical Wnt receptor signaling pathway involved in positive regulation of epithelial to mesenchymal transition |
| GO:0044336 | 0.033 | Inf | 0 | 2 | 2 | canonical Wnt receptor signaling pathway involved in negative regulation of apoptotic process |
| GO:0045082 | 0.033 | Inf | 0 | 2 | 2 | positive regulation of interleukin-10 biosynthetic process |
| GO:0046967 | 0.033 | Inf | 0 | 2 | 2 | cytosol to ER transport |
| GO:0048550 | 0.033 | Inf | 0 | 2 | 2 | negative regulation of pinocytosis |
| GO:0051561 | 0.033 | Inf | 0 | 2 | 2 | elevation of mitochondrial calcium ion concentration |
| GO:0055129 | 0.033 | Inf | 0 | 2 | 2 | L-proline biosynthetic process |
| GO:0060448 | 0.033 | Inf | 0 | 2 | 2 | dichotomous subdivision of terminal units involved in lung branching |
| GO:0060587 | 0.033 | Inf | 0 | 2 | 2 | regulation of lipoprotein lipid oxidation |
| GO:0060700 | 0.033 | Inf | 0 | 2 | 2 | regulation of ribonuclease activity |
| GO:0061047 | 0.033 | Inf | 0 | 2 | 2 | positive regulation of branching involved in lung morphogenesis |
| GO:0071224 | 0.033 | Inf | 0 | 2 | 2 | cellular response to peptidoglycan |
| GO:0071585 | 0.033 | Inf | 0 | 2 | 2 | detoxification of cadmium ion |
| GO:0071638 | 0.033 | Inf | 0 | 2 | 2 | negative regulation of monocyte chemotactic protein-1 production |
| GO:0090182 | 0.033 | Inf | 0 | 2 | 2 | regulation of secretion of lysosomal enzymes |
| GO:2000110 | 0.033 | Inf | 0 | 2 | 2 | negative regulation of macrophage apoptotic process |
| GO:0006029 | 0.035 | 1.878 | 9 | 15 | 51 | proteoglycan metabolic process |
| GO:0006954 | 0.035 | 1.405 | 34 | 44 | 188 | inflammatory response |
| GO:0006144 | 0.035 | 2.252 | 5 | 10 | 30 | purine nucleobase metabolic process |
| GO:0035456 | 0.035 | 3.748 | 2 | 5 | 11 | response to interferon-beta |
| GO:0051701 | 0.036 | 1.292 | 61 | 74 | 334 | interaction with host |
| GO:0032479 | 0.036 | 1.912 | 9 | 14 | 47 | regulation of type I interferon production |
| GO:0032963 | 0.036 | 1.912 | 9 | 14 | 47 | collagen metabolic process |
| GO:0001959 | 0.037 | 1.714 | 13 | 19 | 69 | regulation of cytokine-mediated signaling pathway |
| GO:0009617 | 0.037 | 1.347 | 44 | 55 | 240 | response to bacterium |
| GO:0006690 | 0.038 | 1.952 | 8 | 13 | 43 | icosanoid metabolic process |
| GO:0045621 | 0.038 | 1.803 | 10 | 16 | 56 | positive regulation of lymphocyte differentiation |
| GO:0046822 | 0.040 | 1.490 | 23 | 31 | 125 | regulation of nucleocytoplasmic transport |
| GO:0006022 | 0.040 | 1.567 | 18 | 25 | 97 | aminoglycan metabolic process |
| GO:0042308 | 0.040 | 2.002 | 7 | 12 | 39 | negative regulation of protein import into nucleus |
| GO:0043588 | 0.040 | 2.002 | 7 | 12 | 39 | skin development |
| GO:0035455 | 0.041 | 2.999 | 3 | 6 | 15 | response to interferon-alpha |
| GO:0002577 | 0.041 | 4.495 | 1 | 4 | 8 | regulation of antigen processing and presentation |
| GO:0008631 | 0.041 | 4.495 | 1 | 4 | 8 | intrinsic apoptotic signaling pathway in response to oxidative stress |
| GO:0010613 | 0.041 | 4.495 | 1 | 4 | 8 | positive regulation of cardiac muscle hypertrophy |
| GO:0032928 | 0.041 | 4.495 | 1 | 4 | 8 | regulation of superoxide anion generation |
| GO:0046033 | 0.041 | 4.495 | 1 | 4 | 8 | AMP metabolic process |
| GO:0007159 | 0.042 | 2.064 | 6 | 11 | 35 | leukocyte cell-cell adhesion |
| GO:0050729 | 0.042 | 1.742 | 11 | 17 | 61 | positive regulation of inflammatory response |
| GO:0019320 | 0.042 | 1.680 | 13 | 19 | 70 | hexose catabolic process |
| GO:0046496 | 0.043 | 2.144 | 6 | 10 | 31 | nicotinamide nucleotide metabolic process |
| GO:0032735 | 0.043 | 2.625 | 3 | 7 | 19 | positive regulation of interleukin-12 production |
| GO:0033280 | 0.043 | 2.625 | 3 | 7 | 19 | response to vitamin D |
| GO:0038061 | 0.043 | 2.625 | 3 | 7 | 19 | NIK/NF-kappaB cascade |
| GO:0045622 | 0.043 | 2.625 | 3 | 7 | 19 | regulation of T-helper cell differentiation |
| GO:0042743 | 0.044 | 2.251 | 5 | 9 | 27 | hydrogen peroxide metabolic process |
| GO:0009267 | 0.045 | 1.759 | 10 | 16 | 57 | cellular response to starvation |
| GO:0042274 | 0.045 | 6.766 | 1 | 3 | 5 | ribosomal small subunit biogenesis |
| GO:0000028 | 0.045 | 6.741 | 1 | 3 | 5 | ribosomal small subunit assembly |
| GO:0001510 | 0.045 | 6.741 | 1 | 3 | 5 | RNA methylation |
| GO:0001957 | 0.045 | 6.741 | 1 | 3 | 5 | intramembranous ossification |
| GO:0006032 | 0.045 | 6.741 | 1 | 3 | 5 | chitin catabolic process |
| GO:0006678 | 0.045 | 6.741 | 1 | 3 | 5 | glucosylceramide metabolic process |
| GO:0010535 | 0.045 | 6.741 | 1 | 3 | 5 | positive regulation of activation of JAK2 kinase activity |
| GO:0010592 | 0.045 | 6.741 | 1 | 3 | 5 | positive regulation of lamellipodium assembly |
| GO:0019321 | 0.045 | 6.741 | 1 | 3 | 5 | pentose metabolic process |
| GO:0032471 | 0.045 | 6.741 | 1 | 3 | 5 | reduction of endoplasmic reticulum calcium ion concentration |
| GO:0033015 | 0.045 | 6.741 | 1 | 3 | 5 | tetrapyrrole catabolic process |
| GO:0042167 | 0.045 | 6.741 | 1 | 3 | 5 | heme catabolic process |
| GO:0042989 | 0.045 | 6.741 | 1 | 3 | 5 | sequestering of actin monomers |
| GO:0044342 | 0.045 | 6.741 | 1 | 3 | 5 | type B pancreatic cell proliferation |
| GO:0050748 | 0.045 | 6.741 | 1 | 3 | 5 | negative regulation of lipoprotein metabolic process |
| GO:0051006 | 0.045 | 6.741 | 1 | 3 | 5 | positive regulation of lipoprotein lipase activity |
| GO:0060457 | 0.045 | 6.741 | 1 | 3 | 5 | negative regulation of digestive system process |
| GO:0070234 | 0.045 | 6.741 | 1 | 3 | 5 | positive regulation of T cell apoptotic process |
| GO:0071361 | 0.045 | 6.741 | 1 | 3 | 5 | cellular response to ethanol |
| GO:0071681 | 0.045 | 6.741 | 1 | 3 | 5 | cellular response to indole-3-methanol |
| GO:0090050 | 0.045 | 6.741 | 1 | 3 | 5 | positive regulation of cell migration involved in sprouting angiogenesis |
| GO:2000503 | 0.045 | 6.741 | 1 | 3 | 5 | positive regulation of natural killer cell chemotaxis |
| GO:2000116 | 0.046 | 1.447 | 25 | 33 | 136 | regulation of cysteine-type endopeptidase activity |
| GO:0043603 | 0.046 | 1.572 | 16 | 23 | 89 | cellular amide metabolic process |
| GO:1900180 | 0.046 | 1.524 | 19 | 26 | 103 | regulation of protein localization to nucleus |
| GO:0044236 | 0.048 | 1.703 | 11 | 17 | 62 | multicellular organismal metabolic process |
| GO:0052547 | 0.050 | 1.351 | 36 | 46 | 200 | regulation of peptidase activity |
| GO:0006959 | 0.050 | 1.614 | 14 | 20 | 76 | humoral immune response |
