# Supplementary material for: Hidden Treasures in “Ancient” Microarrays: Gene-Expression Portrays Biology and Potential Resistance Pathways of Major Lung Cancer Subtypes and Normal Tissue
Source: Front Oncol. 2014 Sep 29;4:251. doi: 10.3389/fonc.2014.00251 (PMC4178426; doi:10.3389/fonc.2014.00251)
Supplement: Figure S1 — Clinically relevant differentially expressed pathways in each tissue type versus the rest. The pathway maps are arranged according to the scheme in the left-top corner. Red indicates overexpression in genes, dark green downregulation. In the Cell Cycle pathway, tumor suppressor genes were overexpressed in normal lung tissue while oncogenes and tumor driving cyclins were overexpressed in cancers. The gene thymidylate synthase (TYMS or TS, belonging to the pathway “one carbon pool by folate”) is relevant for tumor growth and is also a treatment target. Notably, TYMS was overexpressed only in the tumors that are generally refractory to the drug pemetrexed, as the squamous and the small-cell lung cancer. TYMS was not overexpressed in mesothelioma, but it is known that TYMS expression is highly variable in this cancer. In the ERBB pathway, the ERBB2/HER2 and ERBB3/HER3 were overexpressed in adenocarcinoma, while the ERBB4 was overexpressed in the carcinoids. [file Data_Sheet_1.ZIP › File S3.ZIP!/GO Analysis/Adenocarcinomas vs Rest Enrichment Analysis/Underrepresented GO terms of Differentially Expressed Genes.html]

Gene to GO BP Conditional test for under-representation

| GOBPID | Pvalue | OddsRatio | ExpCount | Count | Size | Term |
| --- | --- | --- | --- | --- | --- | --- |
| GO:0046395 | 0.001 | 0.563 | 59 | 41 | 146 | carboxylic acid catabolic process |
| GO:0048015 | 0.001 | 0.537 | 48 | 32 | 118 | phosphatidylinositol-mediated signaling |
| GO:0006270 | 0.002 | 0.198 | 10 | 3 | 25 | DNA replication initiation |
| GO:0006298 | 0.003 | 0.097 | 7 | 1 | 16 | mismatch repair |
| GO:0042107 | 0.003 | 0.482 | 31 | 19 | 76 | cytokine metabolic process |
| GO:0009062 | 0.003 | 0.428 | 23 | 13 | 57 | fatty acid catabolic process |
| GO:0001974 | 0.004 | 0.311 | 14 | 6 | 34 | blood vessel remodeling |
| GO:0001776 | 0.004 | 0.391 | 19 | 10 | 47 | leukocyte homeostasis |
| GO:0002200 | 0.004 | 0.338 | 15 | 7 | 37 | somatic diversification of immune receptors |
| GO:0030261 | 0.005 | 0.171 | 8 | 2 | 19 | chromosome condensation |
| GO:0043278 | 0.005 | 0.171 | 8 | 2 | 19 | response to morphine |
| GO:0045745 | 0.005 | <0.0001 | 4 | 0 | 10 | positive regulation of G-protein coupled receptor protein signaling pathway |
| GO:0007050 | 0.005 | 0.716 | 113 | 92 | 277 | cell cycle arrest |
| GO:0016444 | 0.006 | 0.350 | 15 | 7 | 36 | somatic cell DNA recombination |
| GO:0019395 | 0.006 | 0.492 | 27 | 17 | 67 | fatty acid oxidation |
| GO:0010761 | 0.007 | 0.112 | 6 | 1 | 14 | fibroblast migration |
| GO:0031648 | 0.007 | 0.112 | 6 | 1 | 14 | protein destabilization |
| GO:0060716 | 0.007 | 0.112 | 6 | 1 | 14 | labyrinthine layer blood vessel development |
| GO:0051301 | 0.008 | 0.738 | 123 | 102 | 301 | cell division |
| GO:0009566 | 0.008 | 0.459 | 22 | 13 | 54 | fertilization |
| GO:0046635 | 0.009 | 0.387 | 15 | 8 | 38 | positive regulation of alpha-beta T cell activation |
| GO:0042541 | 0.009 | <0.0001 | 4 | 0 | 9 | hemoglobin biosynthetic process |
| GO:0048340 | 0.009 | <0.0001 | 4 | 0 | 9 | paraxial mesoderm morphogenesis |
| GO:0009617 | 0.010 | 0.721 | 98 | 80 | 240 | response to bacterium |
| GO:0044364 | 0.011 | 0.194 | 7 | 2 | 17 | disruption of cells of other organism |
| GO:0051281 | 0.011 | 0.121 | 5 | 1 | 13 | positive regulation of release of sequestered calcium ion into cytosol |
| GO:0006367 | 0.011 | 0.677 | 68 | 53 | 166 | transcription initiation from RNA polymerase II promoter |
| GO:0003012 | 0.013 | 0.726 | 95 | 78 | 233 | muscle system process |
| GO:0032677 | 0.013 | 0.330 | 11 | 5 | 27 | regulation of interleukin-8 production |
| GO:0001892 | 0.013 | 0.515 | 25 | 16 | 61 | embryonic placenta development |
| GO:0009628 | 0.013 | 0.819 | 242 | 216 | 594 | response to abiotic stimulus |
| GO:0086004 | 0.013 | 0.256 | 8 | 3 | 20 | regulation of cardiac muscle cell contraction |
| GO:0003015 | 0.014 | 0.635 | 48 | 36 | 118 | heart process |
| GO:0044707 | 0.014 | 0.903 | 1428 | 1380 | 3498 | single-multicellular organism process |
| GO:0021940 | 0.015 | <0.0001 | 3 | 0 | 8 | positive regulation of cerebellar granule cell precursor proliferation |
| GO:0035357 | 0.015 | <0.0001 | 3 | 0 | 8 | peroxisome proliferator activated receptor signaling pathway |
| GO:0048642 | 0.015 | <0.0001 | 3 | 0 | 8 | negative regulation of skeletal muscle tissue development |
| GO:2000725 | 0.015 | <0.0001 | 3 | 0 | 8 | regulation of cardiac muscle cell differentiation |
| GO:0032504 | 0.015 | 0.788 | 159 | 138 | 390 | multicellular organism reproduction |
| GO:0001953 | 0.016 | 0.207 | 7 | 2 | 16 | negative regulation of cell-matrix adhesion |
| GO:0051898 | 0.016 | 0.207 | 7 | 2 | 16 | negative regulation of protein kinase B signaling cascade |
| GO:0032388 | 0.016 | 0.623 | 42 | 31 | 103 | positive regulation of intracellular transport |
| GO:0006957 | 0.017 | 0.132 | 5 | 1 | 12 | complement activation, alternative pathway |
| GO:0042953 | 0.017 | 0.132 | 5 | 1 | 12 | lipoprotein transport |
| GO:0050832 | 0.017 | 0.132 | 5 | 1 | 12 | defense response to fungus |
| GO:0030324 | 0.018 | 0.654 | 50 | 38 | 122 | lung development |
| GO:0007126 | 0.019 | 0.611 | 37 | 27 | 91 | meiosis |
| GO:0065009 | 0.019 | 0.877 | 529 | 495 | 1299 | regulation of molecular function |
| GO:0010923 | 0.019 | 0.379 | 12 | 6 | 29 | negative regulation of phosphatase activity |
| GO:0016447 | 0.019 | 0.379 | 12 | 6 | 29 | somatic recombination of immunoglobulin gene segments |
| GO:0006700 | 0.020 | 0.272 | 8 | 3 | 19 | C21-steroid hormone biosynthetic process |
| GO:0007040 | 0.020 | 0.272 | 8 | 3 | 19 | lysosome organization |
| GO:0031572 | 0.020 | 0.272 | 8 | 3 | 19 | G2/M transition DNA damage checkpoint |
| GO:0046579 | 0.020 | 0.272 | 8 | 3 | 19 | positive regulation of Ras protein signal transduction |
| GO:0048872 | 0.020 | 0.623 | 39 | 29 | 96 | homeostasis of number of cells |
| GO:0006284 | 0.020 | 0.406 | 13 | 7 | 32 | base-excision repair |
| GO:0006919 | 0.020 | 0.547 | 25 | 17 | 62 | activation of cysteine-type endopeptidase activity involved in apoptotic process |
| GO:0031100 | 0.021 | 0.450 | 15 | 9 | 38 | organ regeneration |
| GO:0060840 | 0.022 | 0.468 | 17 | 10 | 41 | artery development |
| GO:0014032 | 0.022 | 0.483 | 18 | 11 | 44 | neural crest cell development |
| GO:0042108 | 0.022 | 0.483 | 18 | 11 | 44 | positive regulation of cytokine biosynthetic process |
| GO:0032496 | 0.022 | 0.710 | 68 | 55 | 167 | response to lipopolysaccharide |
| GO:0006310 | 0.022 | 0.638 | 41 | 31 | 101 | DNA recombination |
| GO:0042181 | 0.023 | 0.323 | 9 | 4 | 22 | ketone biosynthetic process |
| GO:0010517 | 0.024 | 0.580 | 29 | 20 | 70 | regulation of phospholipase activity |
| GO:0035295 | 0.024 | 0.786 | 132 | 114 | 323 | tube development |
| GO:0002446 | 0.024 | 0.223 | 6 | 2 | 15 | neutrophil mediated immunity |
| GO:0009074 | 0.024 | 0.223 | 6 | 2 | 15 | aromatic amino acid family catabolic process |
| GO:0033146 | 0.024 | 0.223 | 6 | 2 | 15 | regulation of intracellular estrogen receptor signaling pathway |
| GO:0033574 | 0.024 | 0.223 | 6 | 2 | 15 | response to testosterone stimulus |
| GO:0035303 | 0.024 | 0.574 | 27 | 19 | 67 | regulation of dephosphorylation |
| GO:0051384 | 0.024 | 0.639 | 40 | 30 | 98 | response to glucocorticoid stimulus |
| GO:2000107 | 0.025 | 0.363 | 10 | 5 | 25 | negative regulation of leukocyte apoptotic process |
| GO:0051321 | 0.025 | 0.636 | 39 | 29 | 95 | meiotic cell cycle |
| GO:0007186 | 0.025 | 0.808 | 163 | 144 | 400 | G-protein coupled receptor signaling pathway |
| GO:0002679 | 0.026 | <0.0001 | 3 | 0 | 7 | respiratory burst involved in defense response |
| GO:0007096 | 0.026 | <0.0001 | 3 | 0 | 7 | regulation of exit from mitosis |
| GO:0032909 | 0.026 | <0.0001 | 3 | 0 | 7 | regulation of transforming growth factor beta2 production |
| GO:0086069 | 0.026 | <0.0001 | 3 | 0 | 7 | bundle of His cell to Purkinje myocyte communication |
| GO:0000278 | 0.026 | 0.832 | 217 | 195 | 532 | mitotic cell cycle |
| GO:0034644 | 0.026 | 0.396 | 11 | 6 | 28 | cellular response to UV |
| GO:0001946 | 0.027 | 0.145 | 4 | 1 | 11 | lymphangiogenesis |
| GO:0010155 | 0.027 | 0.145 | 4 | 1 | 11 | regulation of proton transport |
| GO:0031998 | 0.027 | 0.145 | 4 | 1 | 11 | regulation of fatty acid beta-oxidation |
| GO:0032715 | 0.027 | 0.145 | 4 | 1 | 11 | negative regulation of interleukin-6 production |
| GO:0060068 | 0.027 | 0.145 | 4 | 1 | 11 | vagina development |
| GO:0071470 | 0.027 | 0.145 | 4 | 1 | 11 | cellular response to osmotic stress |
| GO:0007202 | 0.028 | 0.524 | 20 | 13 | 49 | activation of phospholipase C activity |
| GO:0014013 | 0.028 | 0.524 | 20 | 13 | 49 | regulation of gliogenesis |
| GO:0051174 | 0.028 | 0.868 | 367 | 340 | 901 | regulation of phosphorus metabolic process |
| GO:0006637 | 0.028 | 0.484 | 16 | 10 | 40 | acyl-CoA metabolic process |
| GO:0001782 | 0.028 | 0.290 | 7 | 3 | 18 | B cell homeostasis |
| GO:0001945 | 0.028 | 0.290 | 7 | 3 | 18 | lymph vessel development |
| GO:0048821 | 0.028 | 0.290 | 7 | 3 | 18 | erythrocyte development |
| GO:0001822 | 0.029 | 0.709 | 61 | 49 | 149 | kidney development |
| GO:1901605 | 0.029 | 0.679 | 48 | 38 | 119 | alpha-amino acid metabolic process |
| GO:0010564 | 0.031 | 0.790 | 123 | 107 | 302 | regulation of cell cycle process |
| GO:1901362 | 0.031 | 0.907 | 898 | 861 | 2205 | organic cyclic compound biosynthetic process |
| GO:0000279 | 0.032 | 0.796 | 128 | 112 | 315 | M phase |
| GO:0002026 | 0.032 | 0.342 | 9 | 4 | 21 | regulation of the force of heart contraction |
| GO:0002695 | 0.032 | 0.617 | 31 | 23 | 77 | negative regulation of leukocyte activation |
| GO:0070665 | 0.032 | 0.617 | 31 | 23 | 77 | positive regulation of leukocyte proliferation |
| GO:0009416 | 0.033 | 0.697 | 53 | 42 | 129 | response to light stimulus |
| GO:0050671 | 0.033 | 0.613 | 30 | 22 | 74 | positive regulation of lymphocyte proliferation |
| GO:0070848 | 0.033 | 0.811 | 148 | 131 | 364 | response to growth factor stimulus |
| GO:0046777 | 0.033 | 0.716 | 60 | 49 | 148 | protein autophosphorylation |
| GO:0002221 | 0.033 | 0.660 | 40 | 31 | 99 | pattern recognition receptor signaling pathway |
| GO:0007173 | 0.034 | 0.699 | 53 | 42 | 129 | epidermal growth factor receptor signaling pathway |
| GO:0001975 | 0.034 | 0.382 | 10 | 5 | 24 | response to amphetamine |
| GO:0009607 | 0.035 | 0.826 | 174 | 156 | 428 | response to biotic stimulus |
| GO:0002902 | 0.035 | 0.242 | 6 | 2 | 14 | regulation of B cell apoptotic process |
| GO:0010634 | 0.035 | 0.242 | 6 | 2 | 14 | positive regulation of epithelial cell migration |
| GO:0015721 | 0.035 | 0.242 | 6 | 2 | 14 | bile acid and bile salt transport |
| GO:0030838 | 0.036 | 0.415 | 11 | 6 | 27 | positive regulation of actin filament polymerization |
| GO:0060393 | 0.036 | 0.415 | 11 | 6 | 27 | regulation of pathway-restricted SMAD protein phosphorylation |
| GO:0001817 | 0.036 | 0.778 | 100 | 86 | 245 | regulation of cytokine production |
| GO:0046058 | 0.036 | 0.656 | 38 | 29 | 93 | cAMP metabolic process |
| GO:0045913 | 0.037 | 0.500 | 16 | 10 | 39 | positive regulation of carbohydrate metabolic process |
| GO:0009583 | 0.037 | 0.464 | 13 | 8 | 33 | detection of light stimulus |
| GO:0044282 | 0.037 | 0.752 | 77 | 65 | 190 | small molecule catabolic process |
| GO:0002684 | 0.038 | 0.828 | 172 | 154 | 422 | positive regulation of immune system process |
| GO:2001056 | 0.039 | 0.629 | 31 | 23 | 76 | positive regulation of cysteine-type endopeptidase activity |
| GO:0001654 | 0.039 | 0.765 | 85 | 72 | 208 | eye development |
| GO:0006941 | 0.040 | 0.310 | 7 | 3 | 17 | striated muscle contraction |
| GO:0042094 | 0.041 | 0.311 | 7 | 3 | 17 | interleukin-2 biosynthetic process |
| GO:0048635 | 0.041 | 0.311 | 7 | 3 | 17 | negative regulation of muscle organ development |
| GO:0051298 | 0.041 | 0.311 | 7 | 3 | 17 | centrosome duplication |
| GO:2000249 | 0.041 | 0.311 | 7 | 3 | 17 | regulation of actin cytoskeleton reorganization |
| GO:0045089 | 0.041 | 0.707 | 51 | 41 | 125 | positive regulation of innate immune response |
| GO:0043279 | 0.041 | 0.650 | 34 | 26 | 84 | response to alkaloid |
| GO:0048706 | 0.041 | 0.650 | 34 | 26 | 84 | embryonic skeletal system development |
| GO:0052652 | 0.041 | 0.669 | 39 | 30 | 95 | cyclic purine nucleotide metabolic process |
| GO:0051341 | 0.042 | 0.573 | 22 | 15 | 53 | regulation of oxidoreductase activity |
| GO:0060048 | 0.042 | 0.573 | 22 | 15 | 53 | cardiac muscle contraction |
| GO:0002031 | 0.042 | 0.161 | 4 | 1 | 10 | G-protein coupled receptor internalization |
| GO:0010288 | 0.042 | 0.161 | 4 | 1 | 10 | response to lead ion |
| GO:0010737 | 0.042 | 0.161 | 4 | 1 | 10 | protein kinase A signaling cascade |
| GO:0032769 | 0.042 | 0.161 | 4 | 1 | 10 | negative regulation of monooxygenase activity |
| GO:0033540 | 0.042 | 0.161 | 4 | 1 | 10 | fatty acid beta-oxidation using acyl-CoA oxidase |
| GO:0045824 | 0.042 | 0.161 | 4 | 1 | 10 | negative regulation of innate immune response |
| GO:0046689 | 0.042 | 0.161 | 4 | 1 | 10 | response to mercury ion |
| GO:0048484 | 0.042 | 0.161 | 4 | 1 | 10 | enteric nervous system development |
| GO:0048486 | 0.042 | 0.161 | 4 | 1 | 10 | parasympathetic nervous system development |
| GO:0071364 | 0.042 | 0.161 | 4 | 1 | 10 | cellular response to epidermal growth factor stimulus |
| GO:1901655 | 0.042 | 0.161 | 4 | 1 | 10 | cellular response to ketone |
| GO:0051325 | 0.043 | 0.808 | 127 | 112 | 312 | interphase |
| GO:0010863 | 0.043 | <0.0001 | 2 | 0 | 6 | positive regulation of phospholipase C activity |
| GO:0006942 | 0.043 | 0.564 | 20 | 14 | 50 | regulation of striated muscle contraction |
| GO:0002922 | 0.043 | <0.0001 | 2 | 0 | 6 | positive regulation of humoral immune response |
| GO:0006554 | 0.043 | <0.0001 | 2 | 0 | 6 | lysine catabolic process |
| GO:0006853 | 0.043 | <0.0001 | 2 | 0 | 6 | carnitine shuttle |
| GO:0006924 | 0.043 | <0.0001 | 2 | 0 | 6 | activation-induced cell death of T cells |
| GO:0007342 | 0.043 | <0.0001 | 2 | 0 | 6 | fusion of sperm to egg plasma membrane |
| GO:0016446 | 0.043 | <0.0001 | 2 | 0 | 6 | somatic hypermutation of immunoglobulin genes |
| GO:0018344 | 0.043 | <0.0001 | 2 | 0 | 6 | protein geranylgeranylation |
| GO:0021561 | 0.043 | <0.0001 | 2 | 0 | 6 | facial nerve development |
| GO:0032667 | 0.043 | <0.0001 | 2 | 0 | 6 | regulation of interleukin-23 production |
| GO:0042368 | 0.043 | <0.0001 | 2 | 0 | 6 | vitamin D biosynthetic process |
| GO:0045072 | 0.043 | <0.0001 | 2 | 0 | 6 | regulation of interferon-gamma biosynthetic process |
| GO:0051583 | 0.043 | <0.0001 | 2 | 0 | 6 | dopamine uptake involved in synaptic transmission |
| GO:0051883 | 0.043 | <0.0001 | 2 | 0 | 6 | killing of cells in other organism involved in symbiotic interaction |
| GO:0060136 | 0.043 | <0.0001 | 2 | 0 | 6 | embryonic process involved in female pregnancy |
| GO:0086064 | 0.043 | <0.0001 | 2 | 0 | 6 | cell communication by electrical coupling involved in cardiac conduction |
| GO:0090493 | 0.043 | <0.0001 | 2 | 0 | 6 | catecholamine uptake |
| GO:2000380 | 0.043 | <0.0001 | 2 | 0 | 6 | regulation of mesoderm development |
| GO:0051249 | 0.043 | 0.777 | 91 | 78 | 223 | regulation of lymphocyte activation |
| GO:0032720 | 0.044 | 0.363 | 8 | 4 | 20 | negative regulation of tumor necrosis factor production |
| GO:0071320 | 0.044 | 0.363 | 8 | 4 | 20 | cellular response to cAMP |
| GO:0048285 | 0.046 | 0.785 | 95 | 82 | 233 | organelle fission |
| GO:0007178 | 0.046 | 0.772 | 83 | 71 | 204 | transmembrane receptor protein serine/threonine kinase signaling pathway |
| GO:0000080 | 0.046 | 0.532 | 17 | 11 | 41 | G1 phase of mitotic cell cycle |
| GO:0002889 | 0.047 | 0.403 | 9 | 5 | 23 | regulation of immunoglobulin mediated immune response |
| GO:0060444 | 0.047 | 0.403 | 9 | 5 | 23 | branching involved in mammary gland duct morphogenesis |
| GO:0009791 | 0.047 | 0.607 | 25 | 18 | 61 | post-embryonic development |
| GO:0042446 | 0.048 | 0.461 | 12 | 7 | 29 | hormone biosynthetic process |
| GO:0007276 | 0.048 | 0.799 | 108 | 94 | 264 | gamete generation |
| GO:0006953 | 0.048 | 0.502 | 14 | 9 | 35 | acute-phase response |
| GO:0070227 | 0.048 | 0.502 | 14 | 9 | 35 | lymphocyte apoptotic process |
| GO:0008406 | 0.048 | 0.732 | 57 | 47 | 140 | gonad development |
| GO:0008206 | 0.048 | 0.484 | 13 | 8 | 32 | bile acid metabolic process |
| GO:0006626 | 0.048 | 0.462 | 12 | 7 | 29 | protein targeting to mitochondrion |
| GO:0050867 | 0.049 | 0.769 | 79 | 67 | 193 | positive regulation of cell activation |
