# Supplementary material for: Hidden Treasures in “Ancient” Microarrays: Gene-Expression Portrays Biology and Potential Resistance Pathways of Major Lung Cancer Subtypes and Normal Tissue
Source: Front Oncol. 2014 Sep 29;4:251. doi: 10.3389/fonc.2014.00251 (PMC4178426; doi:10.3389/fonc.2014.00251)
Supplement: Figure S1 — Clinically relevant differentially expressed pathways in each tissue type versus the rest. The pathway maps are arranged according to the scheme in the left-top corner. Red indicates overexpression in genes, dark green downregulation. In the Cell Cycle pathway, tumor suppressor genes were overexpressed in normal lung tissue while oncogenes and tumor driving cyclins were overexpressed in cancers. The gene thymidylate synthase (TYMS or TS, belonging to the pathway “one carbon pool by folate”) is relevant for tumor growth and is also a treatment target. Notably, TYMS was overexpressed only in the tumors that are generally refractory to the drug pemetrexed, as the squamous and the small-cell lung cancer. TYMS was not overexpressed in mesothelioma, but it is known that TYMS expression is highly variable in this cancer. In the ERBB pathway, the ERBB2/HER2 and ERBB3/HER3 were overexpressed in adenocarcinoma, while the ERBB4 was overexpressed in the carcinoids. [file Data_Sheet_1.ZIP › File S3.ZIP!/GO Analysis/Adenocarcinomas vs Rest Enrichment Analysis/Underrepresented GO terms of Down-regulated Genes.html]

Gene to GO BP Conditional test for under-representation

| GOBPID | Pvalue | OddsRatio | ExpCount | Count | Size | Term |
| --- | --- | --- | --- | --- | --- | --- |
| GO:0016064 | <0.0001 | <0.0001 | 11 | 0 | 50 | immunoglobulin mediated immune response |
| GO:0002263 | <0.0001 | 0.208 | 23 | 6 | 104 | cell activation involved in immune response |
| GO:0006959 | <0.0001 | 0.173 | 19 | 4 | 83 | humoral immune response |
| GO:0002696 | <0.0001 | 0.393 | 41 | 19 | 183 | positive regulation of leukocyte activation |
| GO:0009607 | <0.0001 | 0.499 | 64 | 37 | 284 | response to biotic stimulus |
| GO:0072599 | <0.0001 | 0.184 | 18 | 4 | 78 | establishment of protein localization to endoplasmic reticulum |
| GO:0002460 | <0.0001 | 0.255 | 23 | 7 | 100 | adaptive immune response based on somatic recombination of immune receptors built from immunoglobulin superfamily domains |
| GO:0006955 | <0.0001 | 0.422 | 42 | 21 | 181 | immune response |
| GO:0002449 | <0.0001 | 0.328 | 28 | 11 | 125 | lymphocyte mediated immunity |
| GO:0006614 | <0.0001 | 0.192 | 17 | 4 | 75 | SRP-dependent cotranslational protein targeting to membrane |
| GO:0009617 | <0.0001 | 0.407 | 38 | 18 | 167 | response to bacterium |
| GO:0002285 | <0.0001 | 0.163 | 15 | 3 | 66 | lymphocyte activation involved in immune response |
| GO:0002757 | <0.0001 | 0.229 | 18 | 5 | 79 | immune response-activating signal transduction |
| GO:0031349 | <0.0001 | 0.443 | 41 | 21 | 182 | positive regulation of defense response |
| GO:0050865 | <0.0001 | 0.545 | 63 | 39 | 281 | regulation of cell activation |
| GO:0002218 | <0.0001 | 0.323 | 23 | 9 | 104 | activation of innate immune response |
| GO:0043062 | <0.0001 | 0.425 | 34 | 17 | 153 | extracellular structure organization |
| GO:0045088 | <0.0001 | 0.467 | 39 | 21 | 174 | regulation of innate immune response |
| GO:0046634 | <0.0001 | 0.143 | 11 | 2 | 50 | regulation of alpha-beta T cell activation |
| GO:0002221 | <0.0001 | 0.341 | 22 | 9 | 99 | pattern recognition receptor signaling pathway |
| GO:0006415 | <0.0001 | 0.228 | 14 | 4 | 64 | translational termination |
| GO:0070085 | <0.0001 | 0.445 | 33 | 17 | 147 | glycosylation |
| GO:0045621 | 0.001 | 0.194 | 13 | 3 | 56 | positive regulation of lymphocyte differentiation |
| GO:0006486 | 0.001 | 0.449 | 33 | 17 | 146 | protein glycosylation |
| GO:0070663 | 0.001 | 0.397 | 26 | 12 | 115 | regulation of leukocyte proliferation |
| GO:0051249 | 0.001 | 0.401 | 26 | 12 | 113 | regulation of lymphocyte activation |
| GO:0032946 | 0.001 | 0.297 | 17 | 6 | 75 | positive regulation of mononuclear cell proliferation |
| GO:0018279 | 0.001 | 0.240 | 14 | 4 | 61 | protein N-linked glycosylation via asparagine |
| GO:0002521 | 0.001 | 0.585 | 60 | 39 | 265 | leukocyte differentiation |
| GO:0045321 | 0.001 | 0.575 | 56 | 36 | 245 | leukocyte activation |
| GO:0001819 | 0.001 | 0.420 | 27 | 13 | 118 | positive regulation of cytokine production |
| GO:0002200 | 0.001 | 0.095 | 8 | 1 | 37 | somatic diversification of immune receptors |
| GO:0006958 | 0.001 | <0.0001 | 6 | 0 | 27 | complement activation, classical pathway |
| GO:0030199 | 0.001 | <0.0001 | 6 | 0 | 27 | collagen fibril organization |
| GO:0016444 | 0.001 | 0.098 | 8 | 1 | 36 | somatic cell DNA recombination |
| GO:0050670 | 0.001 | 0.418 | 25 | 12 | 110 | regulation of lymphocyte proliferation |
| GO:0010876 | 0.001 | 0.501 | 35 | 20 | 156 | lipid localization |
| GO:0043687 | 0.002 | 0.422 | 25 | 12 | 109 | post-translational protein modification |
| GO:0006414 | 0.002 | 0.316 | 16 | 6 | 71 | translational elongation |
| GO:0060326 | 0.002 | 0.475 | 29 | 16 | 131 | cell chemotaxis |
| GO:0002526 | 0.002 | 0.384 | 20 | 9 | 89 | acute inflammatory response |
| GO:0006413 | 0.002 | 0.444 | 25 | 13 | 113 | translational initiation |
| GO:0043436 | 0.002 | 0.735 | 141 | 112 | 625 | oxoacid metabolic process |
| GO:0002685 | 0.002 | 0.352 | 17 | 7 | 75 | regulation of leukocyte migration |
| GO:0002683 | 0.003 | 0.496 | 30 | 17 | 134 | negative regulation of immune system process |
| GO:0016052 | 0.003 | 0.446 | 23 | 12 | 104 | carbohydrate catabolic process |
| GO:0002429 | 0.003 | 0.432 | 22 | 11 | 98 | immune response-activating cell surface receptor signaling pathway |
| GO:0042742 | 0.003 | 0.363 | 16 | 7 | 73 | defense response to bacterium |
| GO:0006612 | 0.004 | 0.463 | 25 | 13 | 109 | protein targeting to membrane |
| GO:0032388 | 0.004 | 0.451 | 23 | 12 | 103 | positive regulation of intracellular transport |
| GO:0034121 | 0.004 | <0.0001 | 5 | 0 | 22 | regulation of toll-like receptor signaling pathway |
| GO:0009100 | 0.004 | 0.602 | 48 | 32 | 213 | glycoprotein metabolic process |
| GO:0051607 | 0.004 | 0.509 | 29 | 17 | 131 | defense response to virus |
| GO:0001816 | 0.004 | 0.556 | 37 | 23 | 162 | cytokine production |
| GO:0050727 | 0.005 | 0.459 | 23 | 12 | 101 | regulation of inflammatory response |
| GO:0002252 | 0.005 | 0.413 | 19 | 9 | 82 | immune effector process |
| GO:0050870 | 0.005 | 0.413 | 19 | 9 | 83 | positive regulation of T cell activation |
| GO:0002253 | 0.005 | 0.192 | 8 | 2 | 37 | activation of immune response |
| GO:0034341 | 0.005 | 0.433 | 20 | 10 | 89 | response to interferon-gamma |
| GO:0010952 | 0.005 | 0.416 | 19 | 9 | 83 | positive regulation of peptidase activity |
| GO:0045580 | 0.005 | 0.291 | 11 | 4 | 51 | regulation of T cell differentiation |
| GO:2001056 | 0.006 | 0.403 | 17 | 8 | 76 | positive regulation of cysteine-type endopeptidase activity |
| GO:0002287 | 0.006 | 0.123 | 7 | 1 | 29 | alpha-beta T cell activation involved in immune response |
| GO:0002292 | 0.006 | 0.123 | 7 | 1 | 29 | T cell differentiation involved in immune response |
| GO:0016447 | 0.006 | 0.123 | 7 | 1 | 29 | somatic recombination of immunoglobulin gene segments |
| GO:0045087 | 0.006 | 0.566 | 35 | 22 | 152 | innate immune response |
| GO:0006909 | 0.007 | 0.387 | 16 | 7 | 69 | phagocytosis |
| GO:0071216 | 0.007 | 0.444 | 20 | 10 | 87 | cellular response to biotic stimulus |
| GO:0050776 | 0.007 | 0.409 | 17 | 8 | 73 | regulation of immune response |
| GO:0048872 | 0.007 | 0.555 | 32 | 20 | 143 | homeostasis of number of cells |
| GO:0002294 | 0.007 | 0.127 | 6 | 1 | 28 | CD4-positive, alpha-beta T cell differentiation involved in immune response |
| GO:0030101 | 0.007 | 0.127 | 6 | 1 | 28 | natural killer cell activation |
| GO:0046395 | 0.007 | 0.432 | 18 | 9 | 80 | carboxylic acid catabolic process |
| GO:0006919 | 0.007 | 0.367 | 14 | 6 | 62 | activation of cysteine-type endopeptidase activity involved in apoptotic process |
| GO:0048199 | 0.008 | <0.0001 | 4 | 0 | 19 | vesicle targeting, to, from or within Golgi |
| GO:0006509 | 0.009 | 0.132 | 6 | 1 | 27 | membrane protein ectodomain proteolysis |
| GO:0046638 | 0.009 | 0.132 | 6 | 1 | 27 | positive regulation of alpha-beta T cell differentiation |
| GO:0045860 | 0.010 | 0.701 | 71 | 54 | 316 | positive regulation of protein kinase activity |
| GO:0002690 | 0.010 | 0.214 | 8 | 2 | 34 | positive regulation of leukocyte chemotaxis |
| GO:0001974 | 0.010 | 0.214 | 8 | 2 | 34 | blood vessel remodeling |
| GO:0045576 | 0.010 | 0.214 | 8 | 2 | 34 | mast cell activation |
| GO:0002532 | 0.010 | <0.0001 | 4 | 0 | 18 | production of molecular mediator involved in inflammatory response |
| GO:0034142 | 0.010 | 0.428 | 16 | 8 | 72 | toll-like receptor 4 signaling pathway |
| GO:0051222 | 0.010 | 0.565 | 30 | 19 | 134 | positive regulation of protein transport |
| GO:0032963 | 0.011 | 0.319 | 11 | 4 | 47 | collagen metabolic process |
| GO:0050778 | 0.012 | 0.356 | 12 | 5 | 52 | positive regulation of immune response |
| GO:0051704 | 0.012 | 0.824 | 222 | 194 | 983 | multi-organism process |
| GO:0031663 | 0.012 | 0.221 | 7 | 2 | 33 | lipopolysaccharide-mediated signaling pathway |
| GO:0072524 | 0.012 | 0.221 | 7 | 2 | 33 | pyridine-containing compound metabolic process |
| GO:0080134 | 0.013 | 0.778 | 126 | 105 | 562 | regulation of response to stress |
| GO:0006635 | 0.013 | 0.327 | 10 | 4 | 46 | fatty acid beta-oxidation |
| GO:0031640 | 0.013 | <0.0001 | 4 | 0 | 17 | killing of cells of other organism |
| GO:0044058 | 0.013 | <0.0001 | 4 | 0 | 17 | regulation of digestive system process |
| GO:0070206 | 0.013 | <0.0001 | 4 | 0 | 17 | protein trimerization |
| GO:2000516 | 0.013 | <0.0001 | 4 | 0 | 17 | positive regulation of CD4-positive, alpha-beta T cell activation |
| GO:0044282 | 0.013 | 0.640 | 43 | 30 | 190 | small molecule catabolic process |
| GO:1901564 | 0.013 | 0.834 | 243 | 215 | 1079 | organonitrogen compound metabolic process |
| GO:0042107 | 0.014 | 0.460 | 17 | 9 | 76 | cytokine metabolic process |
| GO:0050852 | 0.014 | 0.460 | 17 | 9 | 76 | T cell receptor signaling pathway |
| GO:0002819 | 0.014 | 0.442 | 16 | 8 | 70 | regulation of adaptive immune response |
| GO:0002208 | 0.014 | 0.143 | 6 | 1 | 25 | somatic diversification of immunoglobulins involved in immune response |
| GO:0032481 | 0.014 | 0.143 | 6 | 1 | 25 | positive regulation of type I interferon production |
| GO:0045190 | 0.014 | 0.143 | 6 | 1 | 25 | isotype switching |
| GO:1901606 | 0.014 | 0.421 | 14 | 7 | 64 | alpha-amino acid catabolic process |
| GO:0002260 | 0.014 | 0.286 | 9 | 3 | 39 | lymphocyte homeostasis |
| GO:0002275 | 0.014 | 0.286 | 9 | 3 | 39 | myeloid cell activation involved in immune response |
| GO:0002444 | 0.014 | 0.286 | 9 | 3 | 39 | myeloid leukocyte mediated immunity |
| GO:0042110 | 0.014 | 0.587 | 31 | 20 | 135 | T cell activation |
| GO:0032640 | 0.015 | 0.228 | 7 | 2 | 32 | tumor necrosis factor production |
| GO:0052548 | 0.015 | 0.649 | 44 | 31 | 194 | regulation of endopeptidase activity |
| GO:0006284 | 0.015 | 0.229 | 7 | 2 | 32 | base-excision repair |
| GO:0015908 | 0.015 | 0.335 | 10 | 4 | 45 | fatty acid transport |
| GO:0002377 | 0.016 | 0.373 | 11 | 5 | 51 | immunoglobulin production |
| GO:0007229 | 0.016 | 0.403 | 13 | 6 | 57 | integrin-mediated signaling pathway |
| GO:0001953 | 0.017 | <0.0001 | 4 | 0 | 16 | negative regulation of cell-matrix adhesion |
| GO:0006488 | 0.017 | <0.0001 | 4 | 0 | 16 | dolichol-linked oligosaccharide biosynthetic process |
| GO:0030449 | 0.017 | <0.0001 | 4 | 0 | 16 | regulation of complement activation |
| GO:0070229 | 0.017 | <0.0001 | 4 | 0 | 16 | negative regulation of lymphocyte apoptotic process |
| GO:0010770 | 0.017 | 0.149 | 5 | 1 | 24 | positive regulation of cell morphogenesis involved in differentiation |
| GO:0046496 | 0.018 | 0.237 | 7 | 2 | 31 | nicotinamide nucleotide metabolic process |
| GO:0007162 | 0.019 | 0.457 | 15 | 8 | 68 | negative regulation of cell adhesion |
| GO:0071356 | 0.019 | 0.381 | 11 | 5 | 50 | cellular response to tumor necrosis factor |
| GO:0043123 | 0.019 | 0.536 | 22 | 13 | 96 | positive regulation of I-kappaB kinase/NF-kappaB cascade |
| GO:0002684 | 0.020 | 0.300 | 8 | 3 | 36 | positive regulation of immune system process |
| GO:0009615 | 0.020 | 0.460 | 15 | 8 | 67 | response to virus |
| GO:0006954 | 0.020 | 0.659 | 42 | 30 | 183 | inflammatory response |
| GO:2000106 | 0.020 | 0.303 | 8 | 3 | 37 | regulation of leukocyte apoptotic process |
| GO:0008284 | 0.021 | 0.784 | 113 | 94 | 501 | positive regulation of cell proliferation |
| GO:0006732 | 0.021 | <0.0001 | 3 | 0 | 15 | coenzyme metabolic process |
| GO:0032496 | 0.021 | 0.530 | 20 | 12 | 89 | response to lipopolysaccharide |
| GO:0043085 | 0.021 | 0.815 | 157 | 135 | 696 | positive regulation of catalytic activity |
| GO:0016482 | 0.021 | 0.780 | 106 | 88 | 470 | cytoplasmic transport |
| GO:0002889 | 0.022 | 0.156 | 5 | 1 | 23 | regulation of immunoglobulin mediated immune response |
| GO:0009065 | 0.022 | 0.156 | 5 | 1 | 23 | glutamine family amino acid catabolic process |
| GO:0043370 | 0.022 | 0.156 | 5 | 1 | 23 | regulation of CD4-positive, alpha-beta T cell differentiation |
| GO:0051336 | 0.022 | 0.795 | 123 | 104 | 548 | regulation of hydrolase activity |
| GO:0033081 | 0.022 | <0.0001 | 3 | 0 | 15 | regulation of T cell differentiation in thymus |
| GO:0033574 | 0.022 | <0.0001 | 3 | 0 | 15 | response to testosterone stimulus |
| GO:0034377 | 0.022 | <0.0001 | 3 | 0 | 15 | plasma lipoprotein particle assembly |
| GO:0035455 | 0.022 | <0.0001 | 3 | 0 | 15 | response to interferon-alpha |
| GO:0050691 | 0.022 | <0.0001 | 3 | 0 | 15 | regulation of defense response to virus by host |
| GO:0051181 | 0.022 | <0.0001 | 3 | 0 | 15 | cofactor transport |
| GO:0042100 | 0.022 | 0.352 | 10 | 4 | 43 | B cell proliferation |
| GO:0001892 | 0.022 | 0.444 | 14 | 7 | 61 | embryonic placenta development |
| GO:0034138 | 0.022 | 0.444 | 14 | 7 | 61 | toll-like receptor 3 signaling pathway |
| GO:0060333 | 0.022 | 0.444 | 14 | 7 | 61 | interferon-gamma-mediated signaling pathway |
| GO:0006778 | 0.022 | 0.245 | 7 | 2 | 30 | porphyrin-containing compound metabolic process |
| GO:0022617 | 0.022 | 0.245 | 7 | 2 | 30 | extracellular matrix disassembly |
| GO:0043154 | 0.022 | 0.420 | 12 | 6 | 55 | negative regulation of cysteine-type endopeptidase activity involved in apoptotic process |
| GO:0072376 | 0.022 | 0.420 | 12 | 6 | 55 | protein activation cascade |
| GO:0071260 | 0.022 | 0.390 | 11 | 5 | 49 | cellular response to mechanical stimulus |
| GO:0046365 | 0.024 | 0.490 | 16 | 9 | 72 | monosaccharide catabolic process |
| GO:0051272 | 0.024 | 0.683 | 46 | 34 | 204 | positive regulation of cellular component movement |
| GO:0019083 | 0.024 | 0.598 | 27 | 18 | 121 | viral transcription |
| GO:0008063 | 0.025 | 0.473 | 15 | 8 | 66 | Toll signaling pathway |
| GO:0072329 | 0.025 | 0.473 | 15 | 8 | 66 | monocarboxylic acid catabolic process |
| GO:0006888 | 0.025 | 0.312 | 8 | 3 | 36 | ER to Golgi vesicle-mediated transport |
| GO:0032611 | 0.027 | 0.254 | 7 | 2 | 29 | interleukin-1 beta production |
| GO:0001893 | 0.027 | 0.164 | 5 | 1 | 22 | maternal placenta development |
| GO:0016266 | 0.027 | 0.164 | 5 | 1 | 22 | O-glycan processing |
| GO:0002507 | 0.028 | <0.0001 | 3 | 0 | 14 | tolerance induction |
| GO:0002902 | 0.028 | <0.0001 | 3 | 0 | 14 | regulation of B cell apoptotic process |
| GO:0010761 | 0.028 | <0.0001 | 3 | 0 | 14 | fibroblast migration |
| GO:0031648 | 0.028 | <0.0001 | 3 | 0 | 14 | protein destabilization |
| GO:0060716 | 0.028 | <0.0001 | 3 | 0 | 14 | labyrinthine layer blood vessel development |
| GO:0033365 | 0.028 | 0.766 | 82 | 67 | 365 | protein localization to organelle |
| GO:0035666 | 0.029 | 0.462 | 13 | 7 | 59 | TRIF-dependent toll-like receptor signaling pathway |
| GO:0002824 | 0.029 | 0.322 | 8 | 3 | 35 | positive regulation of adaptive immune response based on somatic recombination of immune receptors built from immunoglobulin superfamily domains |
| GO:0006733 | 0.029 | 0.322 | 8 | 3 | 35 | oxidoreduction coenzyme metabolic process |
| GO:0042113 | 0.029 | 0.503 | 16 | 9 | 70 | B cell activation |
| GO:0009967 | 0.030 | 0.815 | 135 | 116 | 599 | positive regulation of signal transduction |
| GO:0042102 | 0.030 | 0.370 | 9 | 4 | 41 | positive regulation of T cell proliferation |
| GO:0044403 | 0.030 | 0.768 | 82 | 67 | 364 | symbiosis, encompassing mutualism through parasitism |
| GO:0034440 | 0.031 | 0.506 | 16 | 9 | 70 | lipid oxidation |
| GO:0002755 | 0.032 | 0.490 | 14 | 8 | 64 | MyD88-dependent toll-like receptor signaling pathway |
| GO:0007030 | 0.032 | 0.264 | 6 | 2 | 28 | Golgi organization |
| GO:0032652 | 0.032 | 0.264 | 6 | 2 | 28 | regulation of interleukin-1 production |
| GO:0043038 | 0.032 | 0.264 | 6 | 2 | 28 | amino acid activation |
| GO:0006631 | 0.033 | 0.688 | 42 | 31 | 185 | fatty acid metabolic process |
| GO:0030168 | 0.033 | 0.692 | 43 | 32 | 190 | platelet activation |
| GO:0034599 | 0.033 | 0.556 | 19 | 12 | 86 | cellular response to oxidative stress |
| GO:0030260 | 0.033 | 0.172 | 5 | 1 | 21 | entry into host cell |
| GO:0042993 | 0.033 | 0.172 | 5 | 1 | 21 | positive regulation of transcription factor import into nucleus |
| GO:0050853 | 0.033 | 0.172 | 5 | 1 | 21 | B cell receptor signaling pathway |
| GO:0050854 | 0.033 | 0.172 | 5 | 1 | 21 | regulation of antigen receptor-mediated signaling pathway |
| GO:0051828 | 0.033 | 0.172 | 5 | 1 | 21 | entry into other organism involved in symbiotic interaction |
| GO:0052126 | 0.033 | 0.172 | 5 | 1 | 21 | movement in host environment |
| GO:0044242 | 0.034 | 0.602 | 24 | 16 | 107 | cellular lipid catabolic process |
| GO:0006399 | 0.035 | 0.447 | 12 | 6 | 52 | tRNA metabolic process |
| GO:0032655 | 0.035 | 0.332 | 8 | 3 | 34 | regulation of interleukin-12 production |
| GO:0043299 | 0.035 | 0.332 | 8 | 3 | 34 | leukocyte degranulation |
| GO:1901607 | 0.035 | 0.332 | 8 | 3 | 34 | alpha-amino acid biosynthetic process |
| GO:0044283 | 0.035 | 0.756 | 68 | 55 | 303 | small molecule biosynthetic process |
| GO:0030335 | 0.036 | 0.701 | 44 | 33 | 194 | positive regulation of cell migration |
| GO:0006637 | 0.036 | 0.381 | 9 | 4 | 40 | acyl-CoA metabolic process |
| GO:0001562 | 0.036 | <0.0001 | 3 | 0 | 13 | response to protozoan |
| GO:0009084 | 0.036 | <0.0001 | 3 | 0 | 13 | glutamine family amino acid biosynthetic process |
| GO:0009595 | 0.036 | <0.0001 | 3 | 0 | 13 | detection of biotic stimulus |
| GO:0010611 | 0.036 | <0.0001 | 3 | 0 | 13 | regulation of cardiac muscle hypertrophy |
| GO:0032757 | 0.036 | <0.0001 | 3 | 0 | 13 | positive regulation of interleukin-8 production |
| GO:0042255 | 0.036 | <0.0001 | 3 | 0 | 13 | ribosome assembly |
| GO:0046641 | 0.036 | <0.0001 | 3 | 0 | 13 | positive regulation of alpha-beta T cell proliferation |
| GO:0090023 | 0.036 | <0.0001 | 3 | 0 | 13 | positive regulation of neutrophil chemotaxis |
| GO:0019048 | 0.038 | 0.760 | 68 | 55 | 302 | virus-host interaction |
| GO:0046394 | 0.039 | 0.701 | 42 | 32 | 188 | carboxylic acid biosynthetic process |
| GO:0006007 | 0.039 | 0.480 | 13 | 7 | 57 | glucose catabolic process |
| GO:0009108 | 0.039 | 0.480 | 13 | 7 | 57 | coenzyme biosynthetic process |
| GO:0006901 | 0.039 | 0.275 | 6 | 2 | 27 | vesicle coating |
| GO:0071900 | 0.040 | 0.748 | 60 | 48 | 267 | regulation of protein serine/threonine kinase activity |
| GO:0035821 | 0.040 | 0.458 | 11 | 6 | 51 | modification of morphology or physiology of other organism |
| GO:0050728 | 0.040 | 0.458 | 11 | 6 | 51 | negative regulation of inflammatory response |
| GO:0071222 | 0.041 | 0.427 | 10 | 5 | 45 | cellular response to lipopolysaccharide |
| GO:0006693 | 0.041 | 0.181 | 4 | 1 | 20 | prostaglandin metabolic process |
| GO:0032720 | 0.041 | 0.181 | 4 | 1 | 20 | negative regulation of tumor necrosis factor production |
| GO:0046131 | 0.041 | 0.181 | 4 | 1 | 20 | pyrimidine ribonucleoside metabolic process |
| GO:0046627 | 0.041 | 0.181 | 4 | 1 | 20 | negative regulation of insulin receptor signaling pathway |
| GO:0007243 | 0.042 | 0.665 | 32 | 23 | 140 | intracellular protein kinase cascade |
| GO:0002831 | 0.042 | 0.508 | 14 | 8 | 62 | regulation of response to biotic stimulus |
| GO:0006767 | 0.042 | 0.392 | 9 | 4 | 39 | water-soluble vitamin metabolic process |
| GO:0015748 | 0.042 | 0.392 | 9 | 4 | 39 | organophosphate ester transport |
| GO:0010627 | 0.043 | 0.818 | 116 | 100 | 516 | regulation of intracellular protein kinase cascade |
| GO:0050920 | 0.043 | 0.586 | 20 | 13 | 89 | regulation of chemotaxis |
| GO:0044703 | 0.044 | 0.830 | 134 | 117 | 596 | multi-organism reproductive process |
| GO:0001501 | 0.045 | 0.760 | 63 | 51 | 280 | skeletal system development |
| GO:0035295 | 0.047 | 0.778 | 73 | 60 | 323 | tube development |
| GO:0042307 | 0.047 | 0.285 | 6 | 2 | 26 | positive regulation of protein import into nucleus |
| GO:0002347 | 0.047 | <0.0001 | 3 | 0 | 12 | response to tumor cell |
| GO:0006525 | 0.047 | <0.0001 | 3 | 0 | 12 | arginine metabolic process |
| GO:0006957 | 0.047 | <0.0001 | 3 | 0 | 12 | complement activation, alternative pathway |
| GO:0009226 | 0.047 | <0.0001 | 3 | 0 | 12 | nucleotide-sugar biosynthetic process |
| GO:0032489 | 0.047 | <0.0001 | 3 | 0 | 12 | regulation of Cdc42 protein signal transduction |
| GO:0038084 | 0.047 | <0.0001 | 3 | 0 | 12 | vascular endothelial growth factor signaling pathway |
| GO:0042534 | 0.047 | <0.0001 | 3 | 0 | 12 | regulation of tumor necrosis factor biosynthetic process |
| GO:0045624 | 0.047 | <0.0001 | 3 | 0 | 12 | positive regulation of T-helper cell differentiation |
| GO:0046135 | 0.047 | <0.0001 | 3 | 0 | 12 | pyrimidine nucleoside catabolic process |
| GO:0048820 | 0.047 | <0.0001 | 3 | 0 | 12 | hair follicle maturation |
| GO:0061298 | 0.047 | <0.0001 | 3 | 0 | 12 | retina vasculature development in camera-type eye |
| GO:2000696 | 0.047 | <0.0001 | 3 | 0 | 12 | regulation of epithelial cell differentiation involved in kidney development |
| GO:0002699 | 0.047 | 0.552 | 16 | 10 | 72 | positive regulation of immune effector process |
| GO:0006418 | 0.047 | 0.286 | 6 | 2 | 26 | tRNA aminoacylation for protein translation |
| GO:0019835 | 0.047 | 0.286 | 6 | 2 | 26 | cytolysis |
| GO:0032370 | 0.047 | 0.286 | 6 | 2 | 26 | positive regulation of lipid transport |
| GO:0043406 | 0.048 | 0.673 | 31 | 23 | 140 | positive regulation of MAP kinase activity |
| GO:0006917 | 0.048 | 0.679 | 33 | 24 | 145 | induction of apoptosis |
| GO:0042108 | 0.048 | 0.440 | 10 | 5 | 44 | positive regulation of cytokine biosynthetic process |
| GO:0097306 | 0.048 | 0.440 | 10 | 5 | 44 | cellular response to alcohol |
| GO:0009056 | 0.049 | 0.884 | 300 | 277 | 1335 | catabolic process |
| GO:0048008 | 0.049 | 0.355 | 7 | 3 | 32 | platelet-derived growth factor receptor signaling pathway |
| GO:0031100 | 0.050 | 0.404 | 9 | 4 | 38 | organ regeneration |
| GO:0050871 | 0.050 | 0.404 | 9 | 4 | 38 | positive regulation of B cell activation |
