# Supplementary material for: Hidden Treasures in “Ancient” Microarrays: Gene-Expression Portrays Biology and Potential Resistance Pathways of Major Lung Cancer Subtypes and Normal Tissue
Source: Front Oncol. 2014 Sep 29;4:251. doi: 10.3389/fonc.2014.00251 (PMC4178426; doi:10.3389/fonc.2014.00251)
Supplement: Figure S1 — Clinically relevant differentially expressed pathways in each tissue type versus the rest. The pathway maps are arranged according to the scheme in the left-top corner. Red indicates overexpression in genes, dark green downregulation. In the Cell Cycle pathway, tumor suppressor genes were overexpressed in normal lung tissue while oncogenes and tumor driving cyclins were overexpressed in cancers. The gene thymidylate synthase (TYMS or TS, belonging to the pathway “one carbon pool by folate”) is relevant for tumor growth and is also a treatment target. Notably, TYMS was overexpressed only in the tumors that are generally refractory to the drug pemetrexed, as the squamous and the small-cell lung cancer. TYMS was not overexpressed in mesothelioma, but it is known that TYMS expression is highly variable in this cancer. In the ERBB pathway, the ERBB2/HER2 and ERBB3/HER3 were overexpressed in adenocarcinoma, while the ERBB4 was overexpressed in the carcinoids. [file Data_Sheet_1.ZIP › File S3.ZIP!/GO Analysis/Adenocarcinomas vs Rest Enrichment Analysis/Underrepresented GO terms of Up-regulated Genes.html]

Gene to GO BP Conditional test for under-representation

| GOBPID | Pvalue | OddsRatio | ExpCount | Count | Size | Term |
| --- | --- | --- | --- | --- | --- | --- |
| GO:0035637 | <0.0001 | 0.506 | 104 | 60 | 571 | multicellular organismal signaling |
| GO:0007399 | <0.0001 | 0.650 | 219 | 160 | 1201 | nervous system development |
| GO:0032501 | <0.0001 | 0.758 | 668 | 588 | 3664 | multicellular organismal process |
| GO:0000278 | <0.0001 | 0.432 | 57 | 28 | 307 | mitotic cell cycle |
| GO:0051252 | <0.0001 | 0.713 | 306 | 244 | 1680 | regulation of RNA metabolic process |
| GO:0050877 | <0.0001 | 0.627 | 152 | 106 | 832 | neurological system process |
| GO:0048285 | <0.0001 | 0.390 | 42 | 19 | 233 | organelle fission |
| GO:0051325 | <0.0001 | 0.466 | 57 | 30 | 312 | interphase |
| GO:0000086 | <0.0001 | 0.207 | 20 | 5 | 112 | G2/M transition of mitotic cell cycle |
| GO:0051171 | <0.0001 | 0.760 | 378 | 318 | 2072 | regulation of nitrogen compound metabolic process |
| GO:0080090 | <0.0001 | 0.779 | 481 | 418 | 2640 | regulation of primary metabolic process |
| GO:0007268 | <0.0001 | 0.543 | 71 | 43 | 387 | synaptic transmission |
| GO:0090304 | <0.0001 | 0.785 | 454 | 394 | 2488 | nucleic acid metabolic process |
| GO:0051301 | <0.0001 | 0.483 | 46 | 25 | 252 | cell division |
| GO:0000279 | <0.0001 | 0.409 | 32 | 15 | 175 | M phase |
| GO:0032774 | <0.0001 | 0.780 | 339 | 289 | 1862 | RNA biosynthetic process |
| GO:0007018 | <0.0001 | 0.222 | 15 | 4 | 84 | microtubule-based movement |
| GO:0031326 | <0.0001 | 0.746 | 234 | 191 | 1258 | regulation of cellular biosynthetic process |
| GO:0010556 | <0.0001 | 0.745 | 221 | 180 | 1189 | regulation of macromolecule biosynthetic process |
| GO:0010564 | 0.001 | 0.446 | 32 | 16 | 173 | regulation of cell cycle process |
| GO:0051276 | 0.001 | 0.581 | 59 | 38 | 323 | chromosome organization |
| GO:0007275 | 0.001 | 0.801 | 363 | 317 | 1955 | multicellular organismal development |
| GO:1901362 | 0.001 | 0.810 | 400 | 352 | 2190 | organic cyclic compound biosynthetic process |
| GO:0042773 | 0.001 | <0.0001 | 6 | 0 | 34 | ATP synthesis coupled electron transport |
| GO:0010558 | 0.001 | 0.709 | 120 | 92 | 659 | negative regulation of macromolecule biosynthetic process |
| GO:0030030 | 0.001 | 0.709 | 119 | 91 | 652 | cell projection organization |
| GO:0019438 | 0.002 | 0.817 | 385 | 340 | 2108 | aromatic compound biosynthetic process |
| GO:0030705 | 0.002 | 0.106 | 8 | 1 | 43 | cytoskeleton-dependent intracellular transport |
| GO:0015992 | 0.002 | 0.175 | 10 | 2 | 53 | proton transport |
| GO:0031327 | 0.002 | 0.724 | 126 | 98 | 690 | negative regulation of cellular biosynthetic process |
| GO:0045934 | 0.002 | 0.715 | 117 | 90 | 641 | negative regulation of nucleobase-containing compound metabolic process |
| GO:0048699 | 0.002 | 0.678 | 89 | 65 | 481 | generation of neurons |
| GO:0006357 | 0.002 | 0.691 | 95 | 71 | 515 | regulation of transcription from RNA polymerase II promoter |
| GO:0000075 | 0.002 | 0.513 | 33 | 19 | 183 | cell cycle checkpoint |
| GO:0016569 | 0.002 | 0.513 | 33 | 19 | 183 | covalent chromatin modification |
| GO:0018130 | 0.002 | 0.825 | 387 | 344 | 2123 | heterocycle biosynthetic process |
| GO:0048666 | 0.003 | 0.499 | 31 | 17 | 166 | neuron development |
| GO:0007389 | 0.003 | 0.596 | 48 | 31 | 261 | pattern specification process |
| GO:0071363 | 0.003 | 0.646 | 64 | 45 | 352 | cellular response to growth factor stimulus |
| GO:0044271 | 0.003 | 0.833 | 396 | 354 | 2170 | cellular nitrogen compound biosynthetic process |
| GO:0051327 | 0.004 | 0.371 | 17 | 7 | 91 | M phase of meiotic cell cycle |
| GO:0032990 | 0.004 | 0.698 | 88 | 66 | 482 | cell part morphogenesis |
| GO:0009790 | 0.004 | 0.735 | 116 | 91 | 634 | embryo development |
| GO:0010927 | 0.004 | 0.351 | 15 | 6 | 82 | cellular component assembly involved in morphogenesis |
| GO:0007067 | 0.004 | 0.500 | 27 | 15 | 147 | mitosis |
| GO:0034220 | 0.005 | 0.583 | 39 | 25 | 215 | ion transmembrane transport |
| GO:0048015 | 0.005 | 0.457 | 22 | 11 | 118 | phosphatidylinositol-mediated signaling |
| GO:0006120 | 0.005 | <0.0001 | 5 | 0 | 26 | mitochondrial electron transport, NADH to ubiquinone |
| GO:0070647 | 0.006 | 0.676 | 67 | 49 | 369 | protein modification by small protein conjugation or removal |
| GO:0000236 | 0.006 | 0.203 | 8 | 2 | 46 | mitotic prometaphase |
| GO:0048523 | 0.006 | 0.840 | 366 | 328 | 2006 | negative regulation of cellular process |
| GO:0022402 | 0.006 | 0.582 | 38 | 24 | 199 | cell cycle process |
| GO:0032409 | 0.006 | 0.338 | 13 | 5 | 71 | regulation of transporter activity |
| GO:0006270 | 0.006 | <0.0001 | 5 | 0 | 25 | DNA replication initiation |
| GO:0048856 | 0.007 | 0.771 | 146 | 121 | 762 | anatomical structure development |
| GO:0007283 | 0.008 | 0.581 | 35 | 22 | 190 | spermatogenesis |
| GO:0016568 | 0.008 | 0.269 | 10 | 3 | 52 | chromatin modification |
| GO:0009653 | 0.009 | 0.829 | 261 | 230 | 1434 | anatomical structure morphogenesis |
| GO:0045935 | 0.009 | 0.783 | 144 | 120 | 788 | positive regulation of nucleobase-containing compound metabolic process |
| GO:0055021 | 0.010 | <0.0001 | 4 | 0 | 23 | regulation of cardiac muscle tissue growth |
| GO:0030518 | 0.010 | 0.410 | 15 | 7 | 83 | intracellular steroid hormone receptor signaling pathway |
| GO:0006511 | 0.010 | 0.639 | 45 | 31 | 246 | ubiquitin-dependent protein catabolic process |
| GO:0006310 | 0.011 | 0.472 | 19 | 10 | 104 | DNA recombination |
| GO:0035303 | 0.011 | 0.360 | 12 | 5 | 67 | regulation of dephosphorylation |
| GO:0045931 | 0.011 | 0.140 | 6 | 1 | 33 | positive regulation of mitotic cell cycle |
| GO:0032412 | 0.011 | 0.324 | 11 | 4 | 59 | regulation of ion transmembrane transporter activity |
| GO:0007517 | 0.011 | 0.650 | 47 | 33 | 258 | muscle organ development |
| GO:0006936 | 0.011 | 0.613 | 38 | 25 | 206 | muscle contraction |
| GO:0060537 | 0.011 | 0.648 | 46 | 32 | 251 | muscle tissue development |
| GO:0048468 | 0.012 | 0.739 | 89 | 70 | 477 | cell development |
| GO:0048812 | 0.012 | 0.726 | 79 | 61 | 432 | neuron projection morphogenesis |
| GO:0008088 | 0.012 | <0.0001 | 4 | 0 | 22 | axon cargo transport |
| GO:0051783 | 0.012 | 0.365 | 12 | 5 | 66 | regulation of nuclear division |
| GO:0007611 | 0.012 | 0.532 | 24 | 14 | 131 | learning or memory |
| GO:0045893 | 0.013 | 0.786 | 132 | 110 | 725 | positive regulation of transcription, DNA-dependent |
| GO:0051297 | 0.013 | 0.229 | 7 | 2 | 41 | centrosome organization |
| GO:0000723 | 0.014 | 0.291 | 9 | 3 | 49 | telomere maintenance |
| GO:0031623 | 0.014 | 0.291 | 9 | 3 | 49 | receptor internalization |
| GO:0007051 | 0.014 | 0.337 | 10 | 4 | 57 | spindle organization |
| GO:0051100 | 0.014 | 0.337 | 10 | 4 | 57 | negative regulation of binding |
| GO:0043473 | 0.014 | <0.0001 | 4 | 0 | 21 | pigmentation |
| GO:0023061 | 0.015 | 0.667 | 47 | 34 | 260 | signal release |
| GO:0007187 | 0.015 | 0.505 | 20 | 11 | 108 | G-protein coupled receptor signaling pathway, coupled to cyclic nucleotide second messenger |
| GO:0001822 | 0.015 | 0.573 | 27 | 17 | 149 | kidney development |
| GO:1901652 | 0.016 | 0.686 | 53 | 39 | 291 | response to peptide |
| GO:0071495 | 0.016 | 0.769 | 104 | 85 | 572 | cellular response to endogenous stimulus |
| GO:0033044 | 0.016 | 0.433 | 14 | 7 | 79 | regulation of chromosome organization |
| GO:0007127 | 0.016 | 0.298 | 9 | 3 | 48 | meiosis I |
| GO:0035264 | 0.016 | 0.457 | 16 | 8 | 86 | multicellular organism growth |
| GO:0007346 | 0.017 | 0.632 | 37 | 25 | 200 | regulation of mitotic cell cycle |
| GO:0009891 | 0.017 | 0.817 | 172 | 148 | 942 | positive regulation of biosynthetic process |
| GO:0006260 | 0.018 | 0.623 | 34 | 23 | 187 | DNA replication |
| GO:0034453 | 0.018 | <0.0001 | 4 | 0 | 20 | microtubule anchoring |
| GO:0043666 | 0.018 | <0.0001 | 4 | 0 | 20 | regulation of phosphoprotein phosphatase activity |
| GO:0051865 | 0.018 | <0.0001 | 4 | 0 | 20 | protein autoubiquitination |
| GO:0086065 | 0.018 | <0.0001 | 4 | 0 | 20 | cell communication involved in cardiac conduction |
| GO:0000375 | 0.018 | 0.589 | 28 | 18 | 154 | RNA splicing, via transesterification reactions |
| GO:0006271 | 0.018 | 0.154 | 5 | 1 | 30 | DNA strand elongation involved in DNA replication |
| GO:0014855 | 0.018 | 0.154 | 5 | 1 | 30 | striated muscle cell proliferation |
| GO:0000902 | 0.019 | 0.789 | 120 | 100 | 658 | cell morphogenesis |
| GO:0045892 | 0.019 | 0.637 | 36 | 25 | 197 | negative regulation of transcription, DNA-dependent |
| GO:0051128 | 0.020 | 0.818 | 162 | 140 | 891 | regulation of cellular component organization |
| GO:0044260 | 0.020 | 0.885 | 683 | 648 | 3749 | cellular macromolecule metabolic process |
| GO:0022412 | 0.021 | 0.534 | 20 | 12 | 112 | cellular process involved in reproduction in multicellular organism |
| GO:0007189 | 0.021 | 0.248 | 7 | 2 | 38 | adenylate cyclase-activating G-protein coupled receptor signaling pathway |
| GO:0060419 | 0.021 | 0.248 | 7 | 2 | 38 | heart growth |
| GO:0010817 | 0.021 | 0.523 | 19 | 11 | 104 | regulation of hormone levels |
| GO:0010923 | 0.022 | 0.160 | 5 | 1 | 29 | negative regulation of phosphatase activity |
| GO:0048066 | 0.022 | 0.160 | 5 | 1 | 29 | developmental pigmentation |
| GO:0000083 | 0.022 | <0.0001 | 3 | 0 | 19 | regulation of transcription involved in G1/S phase of mitotic cell cycle |
| GO:0006700 | 0.022 | <0.0001 | 3 | 0 | 19 | C21-steroid hormone biosynthetic process |
| GO:0030261 | 0.022 | <0.0001 | 3 | 0 | 19 | chromosome condensation |
| GO:0043278 | 0.022 | <0.0001 | 3 | 0 | 19 | response to morphine |
| GO:0001508 | 0.022 | 0.561 | 23 | 14 | 125 | regulation of action potential |
| GO:0048511 | 0.023 | 0.584 | 25 | 16 | 138 | rhythmic process |
| GO:0006355 | 0.024 | 0.767 | 88 | 72 | 466 | regulation of transcription, DNA-dependent |
| GO:0060048 | 0.024 | 0.364 | 10 | 4 | 53 | cardiac muscle contraction |
| GO:0016579 | 0.024 | 0.255 | 7 | 2 | 37 | protein deubiquitination |
| GO:0044057 | 0.025 | 0.738 | 66 | 52 | 364 | regulation of system process |
| GO:0050767 | 0.025 | 0.713 | 54 | 41 | 296 | regulation of neurogenesis |
| GO:0030178 | 0.025 | 0.459 | 14 | 7 | 75 | negative regulation of Wnt receptor signaling pathway |
| GO:0007050 | 0.025 | 0.532 | 19 | 11 | 102 | cell cycle arrest |
| GO:0015980 | 0.025 | 0.688 | 45 | 33 | 246 | energy derivation by oxidation of organic compounds |
| GO:0045165 | 0.025 | 0.622 | 30 | 20 | 163 | cell fate commitment |
| GO:0001654 | 0.025 | 0.663 | 38 | 27 | 208 | eye development |
| GO:0001754 | 0.026 | 0.166 | 5 | 1 | 28 | eye photoreceptor cell differentiation |
| GO:0016575 | 0.026 | 0.166 | 5 | 1 | 28 | histone deacetylation |
| GO:0031109 | 0.026 | 0.166 | 5 | 1 | 28 | microtubule polymerization or depolymerization |
| GO:0043632 | 0.026 | 0.692 | 46 | 34 | 252 | modification-dependent macromolecule catabolic process |
| GO:0000084 | 0.026 | 0.572 | 22 | 14 | 123 | S phase of mitotic cell cycle |
| GO:0030219 | 0.027 | <0.0001 | 3 | 0 | 18 | megakaryocyte differentiation |
| GO:0060765 | 0.027 | <0.0001 | 3 | 0 | 18 | regulation of androgen receptor signaling pathway |
| GO:0007276 | 0.027 | 0.701 | 48 | 36 | 264 | gamete generation |
| GO:0090068 | 0.027 | 0.549 | 20 | 12 | 109 | positive regulation of cell cycle process |
| GO:0000398 | 0.027 | 0.611 | 27 | 18 | 149 | mRNA splicing, via spliceosome |
| GO:0000122 | 0.028 | 0.743 | 66 | 52 | 362 | negative regulation of transcription from RNA polymerase II promoter |
| GO:0008202 | 0.028 | 0.673 | 39 | 28 | 213 | steroid metabolic process |
| GO:0052652 | 0.028 | 0.524 | 17 | 10 | 95 | cyclic purine nucleotide metabolic process |
| GO:0045665 | 0.029 | 0.263 | 7 | 2 | 36 | negative regulation of neuron differentiation |
| GO:0006873 | 0.029 | 0.771 | 83 | 68 | 458 | cellular ion homeostasis |
| GO:0045840 | 0.030 | 0.172 | 5 | 1 | 27 | positive regulation of mitosis |
| GO:0009952 | 0.031 | 0.610 | 26 | 17 | 141 | anterior/posterior pattern specification |
| GO:0034754 | 0.031 | 0.446 | 12 | 6 | 66 | cellular hormone metabolic process |
| GO:0006302 | 0.032 | 0.473 | 13 | 7 | 73 | double-strand break repair |
| GO:0007270 | 0.032 | 0.473 | 13 | 7 | 73 | neuron-neuron synaptic transmission |
| GO:0051054 | 0.032 | 0.473 | 13 | 7 | 73 | positive regulation of DNA metabolic process |
| GO:0016486 | 0.033 | <0.0001 | 3 | 0 | 17 | peptide hormone processing |
| GO:0032465 | 0.033 | <0.0001 | 3 | 0 | 17 | regulation of cytokinesis |
| GO:2000249 | 0.033 | <0.0001 | 3 | 0 | 17 | regulation of actin cytoskeleton reorganization |
| GO:0006997 | 0.033 | 0.335 | 8 | 3 | 43 | nucleus organization |
| GO:0021510 | 0.033 | 0.335 | 8 | 3 | 43 | spinal cord development |
| GO:0060078 | 0.033 | 0.271 | 6 | 2 | 35 | regulation of postsynaptic membrane potential |
| GO:0071774 | 0.033 | 0.597 | 23 | 15 | 127 | response to fibroblast growth factor stimulus |
| GO:0007178 | 0.034 | 0.678 | 37 | 27 | 204 | transmembrane receptor protein serine/threonine kinase signaling pathway |
| GO:0046058 | 0.035 | 0.537 | 17 | 10 | 93 | cAMP metabolic process |
| GO:0051899 | 0.035 | 0.454 | 12 | 6 | 65 | membrane depolarization |
| GO:0007186 | 0.035 | 0.730 | 53 | 41 | 288 | G-protein coupled receptor signaling pathway |
| GO:0006281 | 0.036 | 0.579 | 21 | 13 | 112 | DNA repair |
| GO:0070830 | 0.036 | 0.179 | 5 | 1 | 26 | tight junction assembly |
| GO:0007224 | 0.036 | 0.388 | 9 | 4 | 50 | smoothened signaling pathway |
| GO:0051093 | 0.037 | 0.765 | 71 | 57 | 387 | negative regulation of developmental process |
| GO:0048667 | 0.037 | 0.777 | 78 | 64 | 429 | cell morphogenesis involved in neuron differentiation |
| GO:0010467 | 0.037 | 0.885 | 390 | 363 | 2122 | gene expression |
| GO:0010638 | 0.037 | 0.526 | 16 | 9 | 85 | positive regulation of organelle organization |
| GO:0031124 | 0.038 | 0.429 | 10 | 5 | 57 | mRNA 3'-end processing |
| GO:0000819 | 0.039 | 0.279 | 6 | 2 | 34 | sister chromatid segregation |
| GO:0035601 | 0.039 | 0.279 | 6 | 2 | 34 | protein deacylation |
| GO:0042177 | 0.039 | 0.279 | 6 | 2 | 34 | negative regulation of protein catabolic process |
| GO:0035270 | 0.039 | 0.585 | 20 | 13 | 112 | endocrine system development |
| GO:0006338 | 0.039 | 0.462 | 12 | 6 | 64 | chromatin remodeling |
| GO:0016202 | 0.039 | 0.510 | 14 | 8 | 78 | regulation of striated muscle tissue development |
| GO:0016573 | 0.039 | 0.510 | 14 | 8 | 78 | histone acetylation |
| GO:0060541 | 0.040 | 0.626 | 25 | 17 | 138 | respiratory system development |
| GO:0030900 | 0.040 | 0.692 | 38 | 28 | 208 | forebrain development |
| GO:0006298 | 0.040 | <0.0001 | 3 | 0 | 16 | mismatch repair |
| GO:0015986 | 0.040 | <0.0001 | 3 | 0 | 16 | ATP synthesis coupled proton transport |
| GO:0023058 | 0.040 | <0.0001 | 3 | 0 | 16 | adaptation of signaling pathway |
| GO:0031113 | 0.040 | <0.0001 | 3 | 0 | 16 | regulation of microtubule polymerization |
| GO:0048665 | 0.040 | <0.0001 | 3 | 0 | 16 | neuron fate specification |
| GO:0051898 | 0.040 | <0.0001 | 3 | 0 | 16 | negative regulation of protein kinase B signaling cascade |
| GO:0060045 | 0.040 | <0.0001 | 3 | 0 | 16 | positive regulation of cardiac muscle cell proliferation |
| GO:0070979 | 0.040 | <0.0001 | 3 | 0 | 16 | protein K11-linked ubiquitination |
| GO:0071599 | 0.040 | <0.0001 | 3 | 0 | 16 | otic vesicle development |
| GO:0072132 | 0.040 | <0.0001 | 3 | 0 | 16 | mesenchyme morphogenesis |
| GO:0009914 | 0.040 | 0.662 | 31 | 22 | 170 | hormone transport |
| GO:0000226 | 0.040 | 0.433 | 10 | 5 | 56 | microtubule cytoskeleton organization |
| GO:0071375 | 0.041 | 0.702 | 40 | 30 | 220 | cellular response to peptide hormone stimulus |
| GO:1901698 | 0.041 | 0.799 | 93 | 78 | 510 | response to nitrogen compound |
| GO:0046883 | 0.041 | 0.636 | 26 | 18 | 144 | regulation of hormone secretion |
| GO:0051781 | 0.041 | 0.397 | 9 | 4 | 49 | positive regulation of cell division |
| GO:0070507 | 0.041 | 0.397 | 9 | 4 | 49 | regulation of microtubule cytoskeleton organization |
| GO:0016358 | 0.042 | 0.551 | 17 | 10 | 91 | dendrite development |
| GO:0008543 | 0.042 | 0.591 | 20 | 13 | 111 | fibroblast growth factor receptor signaling pathway |
| GO:0016925 | 0.043 | 0.186 | 5 | 1 | 25 | protein sumoylation |
| GO:0065008 | 0.043 | 0.877 | 290 | 266 | 1571 | regulation of biological quality |
| GO:0021700 | 0.043 | 0.631 | 25 | 17 | 137 | developmental maturation |
| GO:0043279 | 0.043 | 0.535 | 15 | 9 | 84 | response to alkaloid |
| GO:0007420 | 0.043 | 0.654 | 28 | 20 | 155 | brain development |
| GO:0009411 | 0.044 | 0.517 | 14 | 8 | 77 | response to UV |
| GO:0007286 | 0.044 | 0.353 | 7 | 3 | 41 | spermatid development |
| GO:0048645 | 0.044 | 0.353 | 7 | 3 | 41 | organ formation |
| GO:0006289 | 0.044 | 0.470 | 11 | 6 | 63 | nucleotide-excision repair |
| GO:0006661 | 0.044 | 0.496 | 13 | 7 | 70 | phosphatidylinositol biosynthetic process |
| GO:0040008 | 0.045 | 0.774 | 69 | 56 | 377 | regulation of growth |
| GO:0006312 | 0.045 | 0.288 | 6 | 2 | 33 | mitotic recombination |
| GO:0033762 | 0.045 | 0.288 | 6 | 2 | 33 | response to glucagon stimulus |
| GO:1900542 | 0.046 | 0.727 | 45 | 35 | 249 | regulation of purine nucleotide metabolic process |
| GO:0060415 | 0.047 | 0.406 | 9 | 4 | 48 | muscle tissue morphogenesis |
| GO:0010863 | 0.048 | 0.447 | 10 | 5 | 55 | positive regulation of phospholipase C activity |
| GO:0006839 | 0.048 | 0.525 | 14 | 8 | 76 | mitochondrial transport |
| GO:0051495 | 0.048 | 0.525 | 14 | 8 | 76 | positive regulation of cytoskeleton organization |
| GO:0001578 | 0.049 | <0.0001 | 3 | 0 | 15 | microtubule bundle formation |
| GO:0007214 | 0.049 | <0.0001 | 3 | 0 | 15 | gamma-aminobutyric acid signaling pathway |
| GO:0034312 | 0.049 | <0.0001 | 3 | 0 | 15 | diol biosynthetic process |
| GO:0042423 | 0.049 | <0.0001 | 3 | 0 | 15 | catecholamine biosynthetic process |
| GO:0043928 | 0.049 | <0.0001 | 3 | 0 | 15 | exonucleolytic nuclear-transcribed mRNA catabolic process involved in deadenylation-dependent decay |
| GO:0046189 | 0.049 | <0.0001 | 3 | 0 | 15 | phenol-containing compound biosynthetic process |
| GO:0070849 | 0.049 | <0.0001 | 3 | 0 | 15 | response to epidermal growth factor stimulus |
| GO:0022904 | 0.049 | 0.360 | 7 | 3 | 40 | respiratory electron transport chain |
| GO:0009628 | 0.049 | 0.821 | 108 | 93 | 594 | response to abiotic stimulus |
| GO:0006396 | 0.050 | 0.779 | 68 | 56 | 375 | RNA processing |
