# Supplementary material for: Hidden Treasures in “Ancient” Microarrays: Gene-Expression Portrays Biology and Potential Resistance Pathways of Major Lung Cancer Subtypes and Normal Tissue
Source: Front Oncol. 2014 Sep 29;4:251. doi: 10.3389/fonc.2014.00251 (PMC4178426; doi:10.3389/fonc.2014.00251)
Supplement: Figure S1 — Clinically relevant differentially expressed pathways in each tissue type versus the rest. The pathway maps are arranged according to the scheme in the left-top corner. Red indicates overexpression in genes, dark green downregulation. In the Cell Cycle pathway, tumor suppressor genes were overexpressed in normal lung tissue while oncogenes and tumor driving cyclins were overexpressed in cancers. The gene thymidylate synthase (TYMS or TS, belonging to the pathway “one carbon pool by folate”) is relevant for tumor growth and is also a treatment target. Notably, TYMS was overexpressed only in the tumors that are generally refractory to the drug pemetrexed, as the squamous and the small-cell lung cancer. TYMS was not overexpressed in mesothelioma, but it is known that TYMS expression is highly variable in this cancer. In the ERBB pathway, the ERBB2/HER2 and ERBB3/HER3 were overexpressed in adenocarcinoma, while the ERBB4 was overexpressed in the carcinoids. [file Data_Sheet_1.ZIP › File S3.ZIP!/GO Analysis/Carcinoids vs Rest Enrichment Analysis/Overrepresented GO terms of Differentially Expressed Genes.html]

Gene to GO BP Conditional test for over-representation

| GOBPID | Pvalue | OddsRatio | ExpCount | Count | Size | Term |
| --- | --- | --- | --- | --- | --- | --- |
| GO:0050896 | <0.0001 | 1.193 | 2155 | 2229 | 3503 | response to stimulus |
| GO:0016525 | 0.001 | 3.366 | 28 | 38 | 45 | negative regulation of angiogenesis |
| GO:0035329 | 0.001 | 11.136 | 12 | 18 | 19 | hippo signaling cascade |
| GO:0071276 | 0.002 | Inf | 8 | 13 | 13 | cellular response to cadmium ion |
| GO:0000018 | 0.003 | 9.911 | 11 | 16 | 17 | regulation of DNA recombination |
| GO:0023052 | 0.004 | 1.138 | 1945 | 2002 | 3144 | signaling |
| GO:0050789 | 0.004 | 1.138 | 3067 | 3123 | 4958 | regulation of biological process |
| GO:0008542 | 0.004 | 3.868 | 18 | 25 | 29 | visual learning |
| GO:0016079 | 0.004 | 4.537 | 15 | 22 | 25 | synaptic vesicle exocytosis |
| GO:0007229 | 0.004 | 2.324 | 35 | 45 | 57 | integrin-mediated signaling pathway |
| GO:0007026 | 0.005 | Inf | 7 | 11 | 11 | negative regulation of microtubule depolymerization |
| GO:0033151 | 0.005 | Inf | 7 | 11 | 11 | V(D)J recombination |
| GO:0008360 | 0.005 | 1.999 | 47 | 58 | 76 | regulation of cell shape |
| GO:0048002 | 0.005 | 1.626 | 87 | 102 | 141 | antigen processing and presentation of peptide antigen |
| GO:0044699 | 0.006 | 1.162 | 3918 | 3962 | 6334 | single-organism process |
| GO:0044704 | 0.007 | 2.742 | 24 | 31 | 38 | single-organism reproductive behavior |
| GO:0019884 | 0.008 | 1.612 | 82 | 96 | 133 | antigen processing and presentation of exogenous antigen |
| GO:0006266 | 0.008 | Inf | 6 | 10 | 10 | DNA ligation |
| GO:0001775 | 0.009 | 1.241 | 371 | 398 | 599 | cell activation |
| GO:0034341 | 0.010 | 1.780 | 55 | 66 | 89 | response to interferon-gamma |
| GO:0007052 | 0.010 | 3.094 | 19 | 25 | 30 | mitotic spindle organization |
| GO:0032886 | 0.010 | 2.097 | 35 | 44 | 57 | regulation of microtubule-based process |
| GO:0000079 | 0.011 | 2.036 | 37 | 46 | 60 | regulation of cyclin-dependent protein kinase activity |
| GO:0016477 | 0.013 | 1.214 | 409 | 436 | 661 | cell migration |
| GO:0031110 | 0.013 | Inf | 6 | 9 | 9 | regulation of microtubule polymerization or depolymerization |
| GO:0031293 | 0.013 | Inf | 6 | 9 | 9 | membrane protein intracellular domain proteolysis |
| GO:0051957 | 0.013 | Inf | 6 | 9 | 9 | positive regulation of amino acid transport |
| GO:0090049 | 0.013 | Inf | 6 | 9 | 9 | regulation of cell migration involved in sprouting angiogenesis |
| GO:0000184 | 0.014 | 1.747 | 52 | 62 | 84 | nuclear-transcribed mRNA catabolic process, nonsense-mediated decay |
| GO:0007612 | 0.016 | 1.794 | 46 | 55 | 74 | learning |
| GO:0015849 | 0.016 | 1.467 | 98 | 111 | 158 | organic acid transport |
| GO:0007212 | 0.017 | 3.709 | 13 | 18 | 21 | dopamine receptor signaling pathway |
| GO:0032943 | 0.017 | 1.487 | 90 | 103 | 146 | mononuclear cell proliferation |
| GO:0032069 | 0.017 | 1.946 | 36 | 44 | 58 | regulation of nuclease activity |
| GO:0043277 | 0.018 | 4.635 | 11 | 15 | 17 | apoptotic cell clearance |
| GO:0055012 | 0.018 | 4.635 | 11 | 15 | 17 | ventricular cardiac muscle cell differentiation |
| GO:0002504 | 0.019 | 1.822 | 41 | 50 | 67 | antigen processing and presentation of peptide or polysaccharide antigen via MHC class II |
| GO:0002040 | 0.019 | 2.320 | 24 | 30 | 38 | sprouting angiogenesis |
| GO:0051674 | 0.020 | 1.190 | 432 | 458 | 699 | localization of cell |
| GO:0016445 | 0.021 | 2.577 | 19 | 25 | 31 | somatic diversification of immunoglobulins |
| GO:0043624 | 0.021 | 1.560 | 67 | 78 | 109 | cellular protein complex disassembly |
| GO:0006013 | 0.021 | Inf | 5 | 8 | 8 | mannose metabolic process |
| GO:0007158 | 0.021 | Inf | 5 | 8 | 8 | neuron cell-cell adhesion |
| GO:0008635 | 0.021 | Inf | 5 | 8 | 8 | activation of cysteine-type endopeptidase activity involved in apoptotic process by cytochrome c |
| GO:0046348 | 0.021 | Inf | 5 | 8 | 8 | amino sugar catabolic process |
| GO:0002521 | 0.022 | 1.317 | 164 | 180 | 265 | leukocyte differentiation |
| GO:0007165 | 0.022 | 1.132 | 924 | 958 | 1507 | signal transduction |
| GO:1901293 | 0.023 | 1.353 | 134 | 148 | 216 | nucleoside phosphate biosynthetic process |
| GO:0019886 | 0.023 | 1.785 | 41 | 49 | 66 | antigen processing and presentation of exogenous peptide antigen via MHC class II |
| GO:0030217 | 0.023 | 1.474 | 84 | 95 | 135 | T cell differentiation |
| GO:0002252 | 0.024 | 1.268 | 212 | 230 | 343 | immune effector process |
| GO:0014047 | 0.026 | 4.334 | 10 | 14 | 16 | glutamate secretion |
| GO:0031113 | 0.026 | 4.325 | 10 | 14 | 16 | regulation of microtubule polymerization |
| GO:0009226 | 0.026 | 6.795 | 7 | 11 | 12 | nucleotide-sugar biosynthetic process |
| GO:0014048 | 0.026 | 6.795 | 7 | 11 | 12 | regulation of glutamate secretion |
| GO:0030036 | 0.026 | 1.283 | 184 | 200 | 297 | actin cytoskeleton organization |
| GO:0015031 | 0.027 | 1.165 | 497 | 523 | 804 | protein transport |
| GO:0051251 | 0.027 | 1.400 | 103 | 115 | 166 | positive regulation of lymphocyte activation |
| GO:0007606 | 0.028 | 2.474 | 19 | 24 | 30 | sensory perception of chemical stimulus |
| GO:0006835 | 0.028 | 2.949 | 14 | 19 | 23 | dicarboxylic acid transport |
| GO:0030890 | 0.029 | 2.936 | 14 | 19 | 23 | positive regulation of B cell proliferation |
| GO:0030705 | 0.029 | 2.042 | 27 | 33 | 43 | cytoskeleton-dependent intracellular transport |
| GO:0000226 | 0.029 | 1.380 | 108 | 120 | 174 | microtubule cytoskeleton organization |
| GO:0002366 | 0.030 | 1.529 | 64 | 74 | 104 | leukocyte activation involved in immune response |
| GO:0006836 | 0.030 | 1.549 | 61 | 70 | 98 | neurotransmitter transport |
| GO:0007259 | 0.030 | 1.600 | 53 | 62 | 86 | JAK-STAT cascade |
| GO:0040011 | 0.031 | 1.147 | 588 | 615 | 951 | locomotion |
| GO:0043534 | 0.031 | 1.908 | 30 | 37 | 49 | blood vessel endothelial cell migration |
| GO:0048260 | 0.033 | 3.296 | 12 | 16 | 19 | positive regulation of receptor-mediated endocytosis |
| GO:0060333 | 0.034 | 1.741 | 38 | 45 | 61 | interferon-gamma-mediated signaling pathway |
| GO:0001867 | 0.035 | Inf | 4 | 7 | 7 | complement activation, lectin pathway |
| GO:0002313 | 0.035 | Inf | 4 | 7 | 7 | mature B cell differentiation involved in immune response |
| GO:0002480 | 0.035 | Inf | 4 | 7 | 7 | antigen processing and presentation of exogenous peptide antigen via MHC class I, TAP-independent |
| GO:0006268 | 0.035 | Inf | 4 | 7 | 7 | DNA unwinding involved in replication |
| GO:0008343 | 0.035 | Inf | 4 | 7 | 7 | adult feeding behavior |
| GO:0014051 | 0.035 | Inf | 4 | 7 | 7 | gamma-aminobutyric acid secretion |
| GO:0032495 | 0.035 | Inf | 4 | 7 | 7 | response to muramyl dipeptide |
| GO:0035235 | 0.035 | Inf | 4 | 7 | 7 | ionotropic glutamate receptor signaling pathway |
| GO:0060315 | 0.035 | Inf | 4 | 7 | 7 | negative regulation of ryanodine-sensitive calcium-release channel activity |
| GO:2000463 | 0.035 | Inf | 4 | 7 | 7 | positive regulation of excitatory postsynaptic membrane potential |
| GO:0042098 | 0.035 | 1.508 | 64 | 73 | 103 | T cell proliferation |
| GO:0042593 | 0.035 | 1.527 | 60 | 69 | 97 | glucose homeostasis |
| GO:0035556 | 0.035 | 1.123 | 796 | 825 | 1288 | intracellular signal transduction |
| GO:0032507 | 0.035 | 1.662 | 43 | 51 | 70 | maintenance of protein location in cell |
| GO:0051495 | 0.036 | 1.622 | 47 | 55 | 76 | positive regulation of cytoskeleton organization |
| GO:0019058 | 0.036 | 1.350 | 112 | 124 | 181 | viral infectious cycle |
| GO:0022029 | 0.036 | 2.370 | 18 | 23 | 29 | telencephalon cell migration |
| GO:0051235 | 0.037 | 1.431 | 80 | 90 | 129 | maintenance of location |
| GO:0000726 | 0.037 | 4.016 | 9 | 13 | 15 | non-recombinational repair |
| GO:0035455 | 0.037 | 4.016 | 9 | 13 | 15 | response to interferon-alpha |
| GO:0042119 | 0.037 | 4.016 | 9 | 13 | 15 | neutrophil activation |
| GO:0045907 | 0.037 | 4.016 | 9 | 13 | 15 | positive regulation of vasoconstriction |
| GO:0051181 | 0.037 | 4.016 | 9 | 13 | 15 | cofactor transport |
| GO:0060119 | 0.037 | 4.016 | 9 | 13 | 15 | inner ear receptor cell development |
| GO:0006955 | 0.038 | 1.153 | 486 | 509 | 785 | immune response |
| GO:0008088 | 0.039 | 2.781 | 14 | 18 | 22 | axon cargo transport |
| GO:0050690 | 0.039 | 2.781 | 14 | 18 | 22 | regulation of defense response to virus by virus |
| GO:0050850 | 0.039 | 2.781 | 14 | 18 | 22 | positive regulation of calcium-mediated signaling |
| GO:0006892 | 0.039 | 1.856 | 30 | 36 | 48 | post-Golgi vesicle-mediated transport |
| GO:0000085 | 0.039 | 6.176 | 7 | 10 | 11 | G2 phase of mitotic cell cycle |
| GO:0002833 | 0.039 | 6.176 | 7 | 10 | 11 | positive regulation of response to biotic stimulus |
| GO:0007004 | 0.039 | 6.176 | 7 | 10 | 11 | telomere maintenance via telomerase |
| GO:0032922 | 0.039 | 6.176 | 7 | 10 | 11 | circadian regulation of gene expression |
| GO:0034616 | 0.039 | 6.176 | 7 | 10 | 11 | response to laminar fluid shear stress |
| GO:0035456 | 0.039 | 6.176 | 7 | 10 | 11 | response to interferon-beta |
| GO:0043116 | 0.039 | 6.176 | 7 | 10 | 11 | negative regulation of vascular permeability |
| GO:0045191 | 0.039 | 6.176 | 7 | 10 | 11 | regulation of isotype switching |
| GO:0051494 | 0.041 | 1.733 | 35 | 42 | 57 | negative regulation of cytoskeleton organization |
| GO:0046323 | 0.042 | 2.087 | 22 | 27 | 35 | glucose import |
| GO:0002562 | 0.043 | 2.478 | 15 | 20 | 25 | somatic diversification of immune receptors via germline recombination within a single locus |
| GO:0002312 | 0.044 | 2.476 | 15 | 20 | 25 | B cell activation involved in immune response |
| GO:0002204 | 0.044 | 2.472 | 15 | 20 | 25 | somatic recombination of immunoglobulin genes involved in immune response |
| GO:0060761 | 0.044 | 2.472 | 15 | 20 | 25 | negative regulation of response to cytokine stimulus |
| GO:0002684 | 0.044 | 1.202 | 261 | 278 | 422 | positive regulation of immune system process |
| GO:0034605 | 0.046 | 3.089 | 11 | 15 | 18 | cellular response to heat |
| GO:0048512 | 0.046 | 3.089 | 11 | 15 | 18 | circadian behavior |
| GO:0071822 | 0.047 | 1.151 | 438 | 459 | 708 | protein complex subunit organization |
| GO:0043122 | 0.048 | 1.395 | 80 | 90 | 130 | regulation of I-kappaB kinase/NF-kappaB cascade |
| GO:0048534 | 0.048 | 1.197 | 260 | 277 | 421 | hematopoietic or lymphoid organ development |
| GO:0007420 | 0.048 | 1.214 | 225 | 240 | 363 | brain development |
| GO:0006987 | 0.050 | 1.723 | 33 | 39 | 53 | activation of signaling protein activity involved in unfolded protein response |
| GO:0031646 | 0.050 | 1.723 | 33 | 39 | 53 | positive regulation of neurological system process |
