# Supplementary material for: Hidden Treasures in “Ancient” Microarrays: Gene-Expression Portrays Biology and Potential Resistance Pathways of Major Lung Cancer Subtypes and Normal Tissue
Source: Front Oncol. 2014 Sep 29;4:251. doi: 10.3389/fonc.2014.00251 (PMC4178426; doi:10.3389/fonc.2014.00251)
Supplement: Figure S1 — Clinically relevant differentially expressed pathways in each tissue type versus the rest. The pathway maps are arranged according to the scheme in the left-top corner. Red indicates overexpression in genes, dark green downregulation. In the Cell Cycle pathway, tumor suppressor genes were overexpressed in normal lung tissue while oncogenes and tumor driving cyclins were overexpressed in cancers. The gene thymidylate synthase (TYMS or TS, belonging to the pathway “one carbon pool by folate”) is relevant for tumor growth and is also a treatment target. Notably, TYMS was overexpressed only in the tumors that are generally refractory to the drug pemetrexed, as the squamous and the small-cell lung cancer. TYMS was not overexpressed in mesothelioma, but it is known that TYMS expression is highly variable in this cancer. In the ERBB pathway, the ERBB2/HER2 and ERBB3/HER3 were overexpressed in adenocarcinoma, while the ERBB4 was overexpressed in the carcinoids. [file Data_Sheet_1.ZIP › File S3.ZIP!/GO Analysis/Carcinoids vs Rest Enrichment Analysis/Overrepresented GO terms of Down-regulated Genes.html]

Gene to GO BP Conditional test for over-representation

| GOBPID | Pvalue | OddsRatio | ExpCount | Count | Size | Term |
| --- | --- | --- | --- | --- | --- | --- |
| GO:0006950 | <0.0001 | 1.633 | 496 | 638 | 1957 | response to stress |
| GO:0016032 | <0.0001 | 2.131 | 112 | 180 | 441 | viral reproduction |
| GO:0050865 | <0.0001 | 2.499 | 66 | 117 | 259 | regulation of cell activation |
| GO:0044703 | <0.0001 | 1.881 | 152 | 226 | 596 | multi-organism reproductive process |
| GO:0002696 | <0.0001 | 2.909 | 47 | 90 | 183 | positive regulation of leukocyte activation |
| GO:0002253 | <0.0001 | 2.645 | 55 | 101 | 216 | activation of immune response |
| GO:0006614 | <0.0001 | 4.990 | 19 | 47 | 75 | SRP-dependent cotranslational protein targeting to membrane |
| GO:0006415 | <0.0001 | 5.667 | 16 | 42 | 64 | translational termination |
| GO:0072599 | <0.0001 | 4.757 | 20 | 48 | 78 | establishment of protein localization to endoplasmic reticulum |
| GO:0000184 | <0.0001 | 4.161 | 21 | 49 | 84 | nuclear-transcribed mRNA catabolic process, nonsense-mediated decay |
| GO:0002474 | <0.0001 | 4.161 | 21 | 49 | 84 | antigen processing and presentation of peptide antigen via MHC class I |
| GO:0045087 | <0.0001 | 2.480 | 54 | 96 | 215 | innate immune response |
| GO:0006413 | <0.0001 | 3.374 | 29 | 60 | 113 | translational initiation |
| GO:0006414 | <0.0001 | 4.556 | 18 | 43 | 71 | translational elongation |
| GO:0002684 | <0.0001 | 2.485 | 52 | 92 | 207 | positive regulation of immune system process |
| GO:0002521 | <0.0001 | 2.203 | 68 | 112 | 265 | leukocyte differentiation |
| GO:0002764 | <0.0001 | 2.519 | 47 | 85 | 186 | immune response-regulating signaling pathway |
| GO:0050900 | <0.0001 | 2.820 | 37 | 70 | 145 | leukocyte migration |
| GO:0019058 | <0.0001 | 2.534 | 46 | 83 | 181 | viral infectious cycle |
| GO:0048584 | <0.0001 | 2.538 | 45 | 81 | 180 | positive regulation of response to stimulus |
| GO:0060333 | <0.0001 | 7.275 | 10 | 27 | 38 | interferon-gamma-mediated signaling pathway |
| GO:0006612 | <0.0001 | 3.142 | 28 | 56 | 109 | protein targeting to membrane |
| GO:0006955 | <0.0001 | 2.540 | 40 | 74 | 169 | immune response |
| GO:0002479 | <0.0001 | 4.327 | 16 | 38 | 64 | antigen processing and presentation of exogenous peptide antigen via MHC class I, TAP-dependent |
| GO:0043241 | <0.0001 | 2.973 | 29 | 57 | 114 | protein complex disassembly |
| GO:0002478 | <0.0001 | 4.192 | 17 | 38 | 65 | antigen processing and presentation of exogenous peptide antigen |
| GO:0002429 | <0.0001 | 3.222 | 25 | 51 | 98 | immune response-activating cell surface receptor signaling pathway |
| GO:0070663 | <0.0001 | 2.922 | 29 | 57 | 115 | regulation of leukocyte proliferation |
| GO:0012501 | <0.0001 | 1.464 | 290 | 365 | 1137 | programmed cell death |
| GO:0019882 | <0.0001 | 3.283 | 22 | 45 | 86 | antigen processing and presentation |
| GO:0042060 | <0.0001 | 1.757 | 107 | 154 | 420 | wound healing |
| GO:0045621 | <0.0001 | 4.239 | 14 | 33 | 56 | positive regulation of lymphocyte differentiation |
| GO:0034340 | <0.0001 | 3.979 | 16 | 35 | 61 | response to type I interferon |
| GO:0060337 | <0.0001 | 3.979 | 16 | 35 | 61 | type I interferon-mediated signaling pathway |
| GO:0060326 | <0.0001 | 2.591 | 33 | 61 | 131 | cell chemotaxis |
| GO:2000145 | <0.0001 | 1.844 | 87 | 130 | 342 | regulation of cell motility |
| GO:0048534 | <0.0001 | 1.739 | 107 | 154 | 421 | hematopoietic or lymphoid organ development |
| GO:0002263 | <0.0001 | 2.854 | 27 | 51 | 104 | cell activation involved in immune response |
| GO:0043123 | <0.0001 | 2.964 | 25 | 48 | 96 | positive regulation of I-kappaB kinase/NF-kappaB cascade |
| GO:0007229 | <0.0001 | 4.062 | 15 | 33 | 57 | integrin-mediated signaling pathway |
| GO:0019083 | <0.0001 | 2.645 | 31 | 57 | 121 | viral transcription |
| GO:0030168 | <0.0001 | 2.350 | 40 | 70 | 159 | platelet activation |
| GO:0071310 | <0.0001 | 1.446 | 265 | 332 | 1038 | cellular response to organic substance |
| GO:0051716 | <0.0001 | 1.303 | 809 | 903 | 3200 | cellular response to stimulus |
| GO:0008283 | <0.0001 | 1.968 | 63 | 99 | 258 | cell proliferation |
| GO:0051249 | <0.0001 | 2.670 | 28 | 53 | 113 | regulation of lymphocyte activation |
| GO:0022411 | <0.0001 | 2.242 | 41 | 69 | 161 | cellular component disassembly |
| GO:0051704 | <0.0001 | 1.553 | 151 | 201 | 613 | multi-organism process |
| GO:0044403 | <0.0001 | 1.728 | 93 | 133 | 364 | symbiosis, encompassing mutualism through parasitism |
| GO:0006402 | <0.0001 | 2.471 | 31 | 55 | 121 | mRNA catabolic process |
| GO:0045580 | <0.0001 | 3.334 | 17 | 35 | 66 | regulation of T cell differentiation |
| GO:0016265 | <0.0001 | 1.378 | 321 | 389 | 1259 | death |
| GO:0019048 | <0.0001 | 1.832 | 73 | 108 | 285 | virus-host interaction |
| GO:0009615 | <0.0001 | 3.288 | 17 | 35 | 67 | response to virus |
| GO:0044093 | <0.0001 | 1.455 | 213 | 270 | 835 | positive regulation of molecular function |
| GO:0030335 | <0.0001 | 2.045 | 50 | 79 | 194 | positive regulation of cell migration |
| GO:0001944 | <0.0001 | 1.684 | 99 | 139 | 388 | vasculature development |
| GO:0051272 | <0.0001 | 2.002 | 52 | 82 | 204 | positive regulation of cellular component movement |
| GO:0002460 | <0.0001 | 3.150 | 18 | 36 | 70 | adaptive immune response based on somatic recombination of immune receptors built from immunoglobulin superfamily domains |
| GO:0000084 | <0.0001 | 2.398 | 31 | 55 | 123 | S phase of mitotic cell cycle |
| GO:0043933 | <0.0001 | 1.438 | 221 | 278 | 867 | macromolecular complex subunit organization |
| GO:0006954 | <0.0001 | 2.052 | 46 | 74 | 184 | inflammatory response |
| GO:0016482 | <0.0001 | 1.615 | 112 | 153 | 439 | cytoplasmic transport |
| GO:0051607 | <0.0001 | 2.766 | 22 | 41 | 85 | defense response to virus |
| GO:0072359 | <0.0001 | 1.513 | 152 | 199 | 596 | circulatory system development |
| GO:0010952 | <0.0001 | 2.749 | 21 | 40 | 83 | positive regulation of peptidase activity |
| GO:0050776 | <0.0001 | 2.080 | 41 | 67 | 166 | regulation of immune response |
| GO:0040017 | <0.0001 | 1.928 | 53 | 81 | 206 | positive regulation of locomotion |
| GO:0045184 | <0.0001 | 1.416 | 213 | 265 | 833 | establishment of protein localization |
| GO:0048514 | <0.0001 | 1.713 | 78 | 112 | 308 | blood vessel morphogenesis |
| GO:0007093 | <0.0001 | 2.377 | 28 | 49 | 110 | mitotic cell cycle checkpoint |
| GO:0033365 | <0.0001 | 1.635 | 93 | 129 | 365 | protein localization to organelle |
| GO:0002252 | <0.0001 | 2.966 | 17 | 33 | 67 | immune effector process |
| GO:0030198 | <0.0001 | 2.232 | 32 | 54 | 126 | extracellular matrix organization |
| GO:0043277 | <0.0001 | 16.160 | 3 | 11 | 13 | apoptotic cell clearance |
| GO:0002576 | <0.0001 | 2.730 | 20 | 37 | 77 | platelet degranulation |
| GO:0002285 | <0.0001 | 2.949 | 17 | 33 | 66 | lymphocyte activation involved in immune response |
| GO:0072422 | <0.0001 | 3.046 | 16 | 31 | 61 | signal transduction involved in DNA damage checkpoint |
| GO:0007599 | <0.0001 | 1.620 | 90 | 124 | 354 | hemostasis |
| GO:0002449 | <0.0001 | 2.179 | 32 | 53 | 125 | lymphocyte mediated immunity |
| GO:0000216 | <0.0001 | 2.785 | 18 | 34 | 70 | M/G1 transition of mitotic cell cycle |
| GO:0000003 | <0.0001 | 1.341 | 282 | 338 | 1106 | reproduction |
| GO:0050817 | <0.0001 | 1.623 | 88 | 122 | 348 | coagulation |
| GO:0070727 | <0.0001 | 1.437 | 169 | 214 | 663 | cellular macromolecule localization |
| GO:0072395 | <0.0001 | 2.947 | 16 | 31 | 62 | signal transduction involved in cell cycle checkpoint |
| GO:0052548 | <0.0001 | 1.871 | 50 | 75 | 194 | regulation of endopeptidase activity |
| GO:2000045 | <0.0001 | 2.549 | 21 | 38 | 82 | regulation of G1/S transition of mitotic cell cycle |
| GO:0045786 | <0.0001 | 1.667 | 76 | 107 | 299 | negative regulation of cell cycle |
| GO:0034446 | <0.0001 | 6.862 | 5 | 14 | 20 | substrate adhesion-dependent cell spreading |
| GO:0006977 | <0.0001 | 2.946 | 15 | 30 | 60 | DNA damage response, signal transduction by p53 class mediator resulting in cell cycle arrest |
| GO:0072474 | <0.0001 | 2.946 | 15 | 30 | 60 | signal transduction involved in mitotic cell cycle G1/S checkpoint |
| GO:0031571 | <0.0001 | 2.855 | 16 | 31 | 63 | mitotic cell cycle G1/S transition DNA damage checkpoint |
| GO:0044265 | <0.0001 | 1.507 | 118 | 155 | 463 | cellular macromolecule catabolic process |
| GO:0042981 | <0.0001 | 1.956 | 40 | 62 | 160 | regulation of apoptotic process |
| GO:0045071 | <0.0001 | 5.220 | 6 | 16 | 25 | negative regulation of viral genome replication |
| GO:0032946 | <0.0001 | 2.580 | 19 | 35 | 75 | positive regulation of mononuclear cell proliferation |
| GO:0048870 | <0.0001 | 1.579 | 89 | 121 | 357 | cell motility |
| GO:0035455 | <0.0001 | Inf | 2 | 7 | 7 | response to interferon-alpha |
| GO:0031145 | <0.0001 | 2.629 | 18 | 33 | 70 | anaphase-promoting complex-dependent proteasomal ubiquitin-dependent protein catabolic process |
| GO:0001775 | <0.0001 | 2.027 | 34 | 54 | 138 | cell activation |
| GO:0007155 | <0.0001 | 1.463 | 131 | 169 | 521 | cell adhesion |
| GO:2001056 | <0.0001 | 2.517 | 19 | 35 | 76 | positive regulation of cysteine-type endopeptidase activity |
| GO:0035556 | <0.0001 | 1.361 | 215 | 261 | 856 | intracellular signal transduction |
| GO:0051828 | <0.0001 | 5.868 | 5 | 14 | 21 | entry into other organism involved in symbiotic interaction |
| GO:0052126 | <0.0001 | 5.868 | 5 | 14 | 21 | movement in host environment |
| GO:0010941 | <0.0001 | 1.836 | 46 | 69 | 185 | regulation of cell death |
| GO:0031295 | <0.0001 | 2.943 | 14 | 27 | 54 | T cell costimulation |
| GO:0050670 | <0.0001 | 4.114 | 8 | 18 | 31 | regulation of lymphocyte proliferation |
| GO:0071779 | <0.0001 | 2.505 | 19 | 34 | 74 | G1/S transition checkpoint |
| GO:0010942 | <0.0001 | 1.563 | 88 | 119 | 346 | positive regulation of cell death |
| GO:0048520 | <0.0001 | 2.456 | 20 | 35 | 77 | positive regulation of behavior |
| GO:0032943 | <0.0001 | 3.486 | 10 | 21 | 39 | mononuclear cell proliferation |
| GO:0002407 | <0.0001 | 7.037 | 4 | 12 | 17 | dendritic cell chemotaxis |
| GO:0050920 | <0.0001 | 2.301 | 23 | 39 | 89 | regulation of chemotaxis |
| GO:0032069 | <0.0001 | 2.842 | 14 | 27 | 55 | regulation of nuclease activity |
| GO:0031581 | <0.0001 | 13.182 | 3 | 9 | 11 | hemidesmosome assembly |
| GO:0035456 | <0.0001 | 13.182 | 3 | 9 | 11 | response to interferon-beta |
| GO:0042330 | <0.0001 | 1.458 | 122 | 156 | 476 | taxis |
| GO:0018279 | <0.0001 | 2.668 | 16 | 29 | 61 | protein N-linked glycosylation via asparagine |
| GO:0031570 | <0.0001 | 2.129 | 27 | 44 | 105 | DNA integrity checkpoint |
| GO:0042221 | <0.0001 | 1.247 | 456 | 515 | 1803 | response to chemical stimulus |
| GO:0051325 | <0.0001 | 1.575 | 80 | 108 | 312 | interphase |
| GO:0050852 | <0.0001 | 2.484 | 18 | 32 | 70 | T cell receptor signaling pathway |
| GO:0043281 | <0.0001 | 2.397 | 19 | 34 | 76 | regulation of cysteine-type endopeptidase activity involved in apoptotic process |
| GO:0034655 | <0.0001 | 1.429 | 134 | 169 | 523 | nucleobase-containing compound catabolic process |
| GO:0008360 | <0.0001 | 2.385 | 19 | 34 | 76 | regulation of cell shape |
| GO:0050728 | <0.0001 | 2.828 | 13 | 25 | 51 | negative regulation of inflammatory response |
| GO:0046649 | <0.0001 | 2.446 | 17 | 31 | 70 | lymphocyte activation |
| GO:0060759 | <0.0001 | 2.371 | 19 | 33 | 74 | regulation of response to cytokine stimulus |
| GO:0042127 | <0.0001 | 2.350 | 19 | 33 | 76 | regulation of cell proliferation |
| GO:0031397 | <0.0001 | 2.291 | 20 | 35 | 80 | negative regulation of protein ubiquitination |
| GO:0007051 | <0.0001 | 2.648 | 15 | 27 | 57 | spindle organization |
| GO:0030593 | <0.0001 | 2.939 | 12 | 23 | 46 | neutrophil chemotaxis |
| GO:0006974 | <0.0001 | 1.464 | 105 | 136 | 413 | response to DNA damage stimulus |
| GO:0071216 | <0.0001 | 2.180 | 22 | 37 | 87 | cellular response to biotic stimulus |
| GO:0032269 | <0.0001 | 1.537 | 78 | 104 | 305 | negative regulation of cellular protein metabolic process |
| GO:0051352 | <0.0001 | 2.496 | 16 | 28 | 61 | negative regulation of ligase activity |
| GO:0051437 | <0.0001 | 2.496 | 16 | 28 | 61 | positive regulation of ubiquitin-protein ligase activity involved in mitotic cell cycle |
| GO:0046348 | <0.0001 | 20.488 | 2 | 7 | 8 | amino sugar catabolic process |
| GO:0060548 | <0.0001 | 1.415 | 123 | 155 | 482 | negative regulation of cell death |
| GO:0034620 | <0.0001 | 2.386 | 17 | 30 | 67 | cellular response to unfolded protein |
| GO:0016064 | <0.0001 | 2.298 | 19 | 32 | 73 | immunoglobulin mediated immune response |
| GO:0044699 | 0.001 | 1.296 | 588 | 631 | 2503 | single-organism process |
| GO:0006521 | 0.001 | 2.625 | 14 | 25 | 53 | regulation of cellular amino acid metabolic process |
| GO:0006987 | 0.001 | 2.625 | 14 | 25 | 53 | activation of signaling protein activity involved in unfolded protein response |
| GO:0051897 | 0.001 | 2.625 | 14 | 25 | 53 | positive regulation of protein kinase B signaling cascade |
| GO:0002685 | 0.001 | 2.559 | 14 | 26 | 56 | regulation of leukocyte migration |
| GO:0051436 | 0.001 | 2.548 | 14 | 26 | 56 | negative regulation of ubiquitin-protein ligase activity involved in mitotic cell cycle |
| GO:0030036 | 0.001 | 1.530 | 76 | 101 | 297 | actin cytoskeleton organization |
| GO:0050870 | 0.001 | 3.150 | 9 | 19 | 37 | positive regulation of T cell activation |
| GO:0051707 | 0.001 | 1.636 | 54 | 75 | 215 | response to other organism |
| GO:0010627 | 0.001 | 1.439 | 104 | 133 | 410 | regulation of intracellular protein kinase cascade |
| GO:0072331 | 0.001 | 1.950 | 28 | 43 | 108 | signal transduction by p53 class mediator |
| GO:0000079 | 0.001 | 2.406 | 15 | 27 | 60 | regulation of cyclin-dependent protein kinase activity |
| GO:1901568 | 0.001 | 2.803 | 11 | 21 | 43 | fatty acid derivative metabolic process |
| GO:0007163 | 0.001 | 2.056 | 23 | 37 | 90 | establishment or maintenance of cell polarity |
| GO:0044085 | 0.001 | 1.261 | 276 | 319 | 1082 | cellular component biogenesis |
| GO:0050850 | 0.001 | 4.235 | 6 | 13 | 22 | positive regulation of calcium-mediated signaling |
| GO:0006461 | 0.001 | 1.364 | 138 | 170 | 542 | protein complex assembly |
| GO:0042119 | 0.001 | 5.859 | 4 | 10 | 15 | neutrophil activation |
| GO:0043372 | 0.001 | 5.859 | 4 | 10 | 15 | positive regulation of CD4-positive, alpha-beta T cell differentiation |
| GO:0008284 | 0.001 | 1.420 | 103 | 131 | 407 | positive regulation of cell proliferation |
| GO:0031100 | 0.001 | 2.936 | 10 | 19 | 38 | organ regeneration |
| GO:0006271 | 0.001 | 3.353 | 8 | 16 | 30 | DNA strand elongation involved in DNA replication |
| GO:0071706 | 0.001 | 2.449 | 14 | 25 | 55 | tumor necrosis factor superfamily cytokine production |
| GO:0002634 | 0.001 | Inf | 1 | 5 | 5 | regulation of germinal center formation |
| GO:0034351 | 0.001 | Inf | 1 | 5 | 5 | negative regulation of glial cell apoptotic process |
| GO:0050855 | 0.001 | Inf | 1 | 5 | 5 | regulation of B cell receptor signaling pathway |
| GO:0050729 | 0.001 | 2.335 | 16 | 27 | 61 | positive regulation of inflammatory response |
| GO:0032651 | 0.001 | 3.732 | 6 | 14 | 25 | regulation of interleukin-1 beta production |
| GO:2000107 | 0.001 | 3.732 | 6 | 14 | 25 | negative regulation of leukocyte apoptotic process |
| GO:0006984 | 0.001 | 2.140 | 19 | 32 | 76 | ER-nucleus signaling pathway |
| GO:0030832 | 0.001 | 2.206 | 18 | 30 | 70 | regulation of actin filament length |
| GO:0015949 | 0.001 | 6.589 | 3 | 9 | 13 | nucleobase-containing small molecule interconversion |
| GO:0051271 | 0.001 | 1.904 | 27 | 42 | 107 | negative regulation of cellular component movement |
| GO:0071840 | 0.001 | 1.219 | 407 | 454 | 1635 | cellular component organization or biogenesis |
| GO:0006886 | 0.001 | 1.486 | 75 | 98 | 296 | intracellular protein transport |
| GO:0000085 | 0.001 | 7.806 | 3 | 8 | 11 | G2 phase of mitotic cell cycle |
| GO:0008154 | 0.001 | 1.999 | 23 | 36 | 89 | actin polymerization or depolymerization |
| GO:0051223 | 0.001 | 1.572 | 56 | 76 | 219 | regulation of protein transport |
| GO:0002294 | 0.001 | 3.384 | 7 | 15 | 28 | CD4-positive, alpha-beta T cell differentiation involved in immune response |
| GO:0006959 | 0.001 | 2.069 | 20 | 33 | 80 | humoral immune response |
| GO:0002313 | 0.002 | 17.553 | 2 | 6 | 7 | mature B cell differentiation involved in immune response |
| GO:0002480 | 0.002 | 17.553 | 2 | 6 | 7 | antigen processing and presentation of exogenous peptide antigen via MHC class I, TAP-independent |
| GO:0030890 | 0.002 | 3.811 | 6 | 13 | 23 | positive regulation of B cell proliferation |
| GO:0071230 | 0.002 | 3.811 | 6 | 13 | 23 | cellular response to amino acid stimulus |
| GO:0090026 | 0.002 | 10.242 | 2 | 7 | 9 | positive regulation of monocyte chemotaxis |
| GO:0051247 | 0.002 | 2.457 | 13 | 23 | 51 | positive regulation of protein metabolic process |
| GO:0051169 | 0.002 | 1.506 | 67 | 88 | 261 | nuclear transport |
| GO:0045859 | 0.002 | 1.369 | 117 | 144 | 457 | regulation of protein kinase activity |
| GO:0071559 | 0.002 | 1.662 | 41 | 58 | 161 | response to transforming growth factor beta stimulus |
| GO:0051338 | 0.002 | 1.352 | 126 | 154 | 493 | regulation of transferase activity |
| GO:0051438 | 0.002 | 2.046 | 20 | 32 | 78 | regulation of ubiquitin-protein ligase activity |
| GO:0051351 | 0.002 | 2.131 | 18 | 29 | 69 | positive regulation of ligase activity |
| GO:0006917 | 0.002 | 1.699 | 37 | 53 | 145 | induction of apoptosis |
| GO:0001960 | 0.002 | 3.907 | 5 | 12 | 21 | negative regulation of cytokine-mediated signaling pathway |
| GO:0031399 | 0.002 | 1.278 | 195 | 229 | 765 | regulation of protein modification process |
| GO:0002287 | 0.002 | 3.142 | 7 | 15 | 29 | alpha-beta T cell activation involved in immune response |
| GO:0002292 | 0.002 | 3.142 | 7 | 15 | 29 | T cell differentiation involved in immune response |
| GO:0010811 | 0.002 | 2.552 | 11 | 20 | 43 | positive regulation of cell-substrate adhesion |
| GO:0042098 | 0.002 | 3.504 | 6 | 13 | 24 | T cell proliferation |
| GO:0002690 | 0.002 | 2.947 | 8 | 16 | 32 | positive regulation of leukocyte chemotaxis |
| GO:0046456 | 0.002 | 2.933 | 8 | 16 | 32 | icosanoid biosynthetic process |
| GO:0051246 | 0.003 | 1.396 | 94 | 118 | 376 | regulation of protein metabolic process |
| GO:0044259 | 0.003 | 2.329 | 13 | 23 | 52 | multicellular organismal macromolecule metabolic process |
| GO:0071702 | 0.003 | 1.222 | 301 | 340 | 1182 | organic substance transport |
| GO:0045622 | 0.003 | 4.028 | 5 | 11 | 19 | regulation of T-helper cell differentiation |
| GO:0048199 | 0.003 | 4.028 | 5 | 11 | 19 | vesicle targeting, to, from or within Golgi |
| GO:2000401 | 0.003 | 4.028 | 5 | 11 | 19 | regulation of lymphocyte migration |
| GO:0007159 | 0.003 | 2.770 | 9 | 17 | 35 | leukocyte cell-cell adhesion |
| GO:0034097 | 0.003 | 1.515 | 57 | 76 | 229 | response to cytokine stimulus |
| GO:0050727 | 0.003 | 2.485 | 11 | 20 | 44 | regulation of inflammatory response |
| GO:0002526 | 0.003 | 1.907 | 23 | 35 | 89 | acute inflammatory response |
| GO:0007259 | 0.003 | 2.651 | 10 | 18 | 38 | JAK-STAT cascade |
| GO:0030199 | 0.003 | 3.157 | 7 | 14 | 27 | collagen fibril organization |
| GO:0070925 | 0.003 | 2.087 | 17 | 27 | 65 | organelle assembly |
| GO:0043687 | 0.003 | 1.775 | 28 | 41 | 109 | post-translational protein modification |
| GO:0030833 | 0.003 | 2.121 | 16 | 26 | 62 | regulation of actin filament polymerization |
| GO:0009225 | 0.003 | 3.516 | 6 | 12 | 22 | nucleotide-sugar metabolic process |
| GO:0046718 | 0.004 | 4.183 | 4 | 10 | 17 | viral entry into host cell |
| GO:0000278 | 0.004 | 1.441 | 71 | 91 | 280 | mitotic cell cycle |
| GO:0051649 | 0.004 | 1.207 | 321 | 360 | 1258 | establishment of localization in cell |
| GO:0031663 | 0.004 | 2.760 | 8 | 16 | 33 | lipopolysaccharide-mediated signaling pathway |
| GO:0030336 | 0.004 | 1.803 | 26 | 38 | 100 | negative regulation of cell migration |
| GO:0031398 | 0.004 | 1.803 | 26 | 38 | 100 | positive regulation of protein ubiquitination |
| GO:0042129 | 0.004 | 3.197 | 6 | 13 | 25 | regulation of T cell proliferation |
| GO:0046634 | 0.004 | 3.189 | 6 | 13 | 25 | regulation of alpha-beta T cell activation |
| GO:0016444 | 0.004 | 2.624 | 9 | 17 | 36 | somatic cell DNA recombination |
| GO:0006270 | 0.004 | 3.174 | 6 | 13 | 25 | DNA replication initiation |
| GO:2000514 | 0.004 | 3.174 | 6 | 13 | 25 | regulation of CD4-positive, alpha-beta T cell activation |
| GO:0042113 | 0.004 | 2.973 | 7 | 14 | 28 | B cell activation |
| GO:0030260 | 0.004 | Inf | 1 | 4 | 4 | entry into host cell |
| GO:0002829 | 0.004 | Inf | 1 | 4 | 4 | negative regulation of type 2 immune response |
| GO:0006048 | 0.004 | Inf | 1 | 4 | 4 | UDP-N-acetylglucosamine biosynthetic process |
| GO:0032020 | 0.004 | Inf | 1 | 4 | 4 | ISG15-protein conjugation |
| GO:0048548 | 0.004 | Inf | 1 | 4 | 4 | regulation of pinocytosis |
| GO:0070431 | 0.004 | Inf | 1 | 4 | 4 | nucleotide-binding oligomerization domain containing 2 signaling pathway |
| GO:2000425 | 0.004 | Inf | 1 | 4 | 4 | regulation of apoptotic cell clearance |
| GO:0016525 | 0.004 | 2.347 | 11 | 20 | 45 | negative regulation of angiogenesis |
| GO:0006919 | 0.004 | 2.182 | 14 | 23 | 54 | activation of cysteine-type endopeptidase activity involved in apoptotic process |
| GO:0050818 | 0.004 | 2.097 | 15 | 25 | 60 | regulation of coagulation |
| GO:0009112 | 0.004 | 2.282 | 12 | 21 | 48 | nucleobase metabolic process |
| GO:0043066 | 0.005 | 1.338 | 109 | 133 | 430 | negative regulation of apoptotic process |
| GO:0050657 | 0.005 | 1.866 | 22 | 33 | 85 | nucleic acid transport |
| GO:0051236 | 0.005 | 1.866 | 22 | 33 | 85 | establishment of RNA localization |
| GO:0030224 | 0.005 | 3.580 | 5 | 11 | 20 | monocyte differentiation |
| GO:0032732 | 0.005 | 3.580 | 5 | 11 | 20 | positive regulation of interleukin-1 production |
| GO:0046627 | 0.005 | 3.580 | 5 | 11 | 20 | negative regulation of insulin receptor signaling pathway |
| GO:0048247 | 0.005 | 3.580 | 5 | 11 | 20 | lymphocyte chemotaxis |
| GO:0002577 | 0.005 | 8.775 | 2 | 6 | 8 | regulation of antigen processing and presentation |
| GO:0045059 | 0.005 | 8.775 | 2 | 6 | 8 | positive thymic T cell selection |
| GO:0006417 | 0.005 | 1.640 | 35 | 49 | 137 | regulation of translation |
| GO:0034329 | 0.005 | 1.640 | 35 | 49 | 137 | cell junction assembly |
| GO:0001516 | 0.005 | 4.391 | 4 | 9 | 15 | prostaglandin biosynthetic process |
| GO:0006002 | 0.005 | 14.620 | 2 | 5 | 6 | fructose 6-phosphate metabolic process |
| GO:0030490 | 0.005 | 14.620 | 2 | 5 | 6 | maturation of SSU-rRNA |
| GO:0032494 | 0.005 | 14.620 | 2 | 5 | 6 | response to peptidoglycan |
| GO:0036003 | 0.005 | 14.620 | 2 | 5 | 6 | positive regulation of transcription from RNA polymerase II promoter in response to stress |
| GO:0042273 | 0.005 | 14.620 | 2 | 5 | 6 | ribosomal large subunit biogenesis |
| GO:0045630 | 0.005 | 14.620 | 2 | 5 | 6 | positive regulation of T-helper 2 cell differentiation |
| GO:0061439 | 0.005 | 14.620 | 2 | 5 | 6 | kidney vasculature morphogenesis |
| GO:0070208 | 0.005 | 14.620 | 2 | 5 | 6 | protein heterotrimerization |
| GO:0007249 | 0.005 | 2.360 | 11 | 19 | 43 | I-kappaB kinase/NF-kappaB cascade |
| GO:0019221 | 0.005 | 1.582 | 39 | 54 | 157 | cytokine-mediated signaling pathway |
| GO:0006636 | 0.005 | 2.606 | 9 | 16 | 34 | unsaturated fatty acid biosynthetic process |
| GO:0071347 | 0.005 | 2.606 | 9 | 16 | 34 | cellular response to interleukin-1 |
| GO:0032964 | 0.006 | 3.196 | 6 | 12 | 23 | collagen biosynthetic process |
| GO:0060334 | 0.006 | 3.196 | 6 | 12 | 23 | regulation of interferon-gamma-mediated signaling pathway |
| GO:0002504 | 0.006 | 1.982 | 17 | 27 | 67 | antigen processing and presentation of peptide or polysaccharide antigen via MHC class II |
| GO:0002200 | 0.006 | 2.492 | 9 | 17 | 37 | somatic diversification of immune receptors |
| GO:0034976 | 0.006 | 1.830 | 22 | 33 | 86 | response to endoplasmic reticulum stress |
| GO:0045765 | 0.006 | 1.870 | 20 | 31 | 80 | regulation of angiogenesis |
| GO:0010498 | 0.006 | 1.555 | 42 | 57 | 165 | proteasomal protein catabolic process |
| GO:0045089 | 0.006 | 1.656 | 32 | 45 | 125 | positive regulation of innate immune response |
| GO:0002221 | 0.006 | 1.755 | 25 | 37 | 99 | pattern recognition receptor signaling pathway |
| GO:0033077 | 0.006 | 2.109 | 14 | 23 | 55 | T cell differentiation in thymus |
| GO:0006468 | 0.006 | 1.230 | 217 | 248 | 851 | protein phosphorylation |
| GO:0009056 | 0.006 | 1.188 | 341 | 378 | 1335 | catabolic process |
| GO:0051493 | 0.006 | 1.680 | 29 | 42 | 116 | regulation of cytoskeleton organization |
| GO:0048519 | 0.006 | 1.238 | 204 | 234 | 825 | negative regulation of biological process |
| GO:0002544 | 0.006 | 3.660 | 5 | 10 | 18 | chronic inflammatory response |
| GO:0061437 | 0.006 | 3.660 | 5 | 10 | 18 | renal system vasculature development |
| GO:0002695 | 0.006 | 1.875 | 20 | 30 | 77 | negative regulation of leukocyte activation |
| GO:0046641 | 0.006 | 4.682 | 3 | 8 | 13 | positive regulation of alpha-beta T cell proliferation |
| GO:0090307 | 0.006 | 4.682 | 3 | 8 | 13 | spindle assembly involved in mitosis |
| GO:0030855 | 0.006 | 1.465 | 55 | 72 | 217 | epithelial cell differentiation |
| GO:0007568 | 0.007 | 1.559 | 40 | 54 | 156 | aging |
| GO:1900180 | 0.007 | 1.719 | 26 | 38 | 103 | regulation of protein localization to nucleus |
| GO:0009967 | 0.007 | 1.273 | 145 | 170 | 570 | positive regulation of signal transduction |
| GO:0006284 | 0.007 | 2.586 | 8 | 15 | 32 | base-excision repair |
| GO:0032970 | 0.007 | 1.543 | 41 | 55 | 160 | regulation of actin filament-based process |
| GO:0050792 | 0.007 | 1.735 | 25 | 36 | 97 | regulation of viral reproduction |
| GO:1900046 | 0.008 | 2.045 | 14 | 23 | 56 | regulation of hemostasis |
| GO:0042325 | 0.008 | 1.251 | 166 | 193 | 652 | regulation of phosphorylation |
| GO:0040013 | 0.008 | 1.647 | 30 | 42 | 117 | negative regulation of locomotion |
| GO:0010212 | 0.008 | 1.851 | 19 | 29 | 75 | response to ionizing radiation |
| GO:0016053 | 0.008 | 1.486 | 48 | 63 | 188 | organic acid biosynthetic process |
| GO:0010648 | 0.008 | 1.293 | 120 | 143 | 471 | negative regulation of cell communication |
| GO:0050853 | 0.008 | 3.773 | 4 | 9 | 16 | B cell receptor signaling pathway |
| GO:0006901 | 0.008 | 3.771 | 4 | 9 | 16 | vesicle coating |
| GO:0033151 | 0.009 | 5.119 | 3 | 7 | 11 | V(D)J recombination |
| GO:0071674 | 0.009 | 5.119 | 3 | 7 | 11 | mononuclear cell migration |
| GO:0048585 | 0.009 | 1.282 | 129 | 152 | 506 | negative regulation of response to stimulus |
| GO:0006488 | 0.009 | 3.763 | 4 | 9 | 16 | dolichol-linked oligosaccharide biosynthetic process |
| GO:0010718 | 0.009 | 3.763 | 4 | 9 | 16 | positive regulation of epithelial to mesenchymal transition |
| GO:0030449 | 0.009 | 3.763 | 4 | 9 | 16 | regulation of complement activation |
| GO:0050704 | 0.009 | 3.763 | 4 | 9 | 16 | regulation of interleukin-1 secretion |
| GO:0006606 | 0.009 | 1.513 | 43 | 57 | 168 | protein import into nucleus |
| GO:0019886 | 0.009 | 1.908 | 17 | 26 | 66 | antigen processing and presentation of exogenous peptide antigen via MHC class II |
| GO:0002244 | 0.009 | 2.720 | 7 | 13 | 27 | hematopoietic progenitor cell differentiation |
| GO:0044092 | 0.009 | 1.281 | 126 | 149 | 494 | negative regulation of molecular function |
| GO:0030183 | 0.009 | 1.931 | 16 | 25 | 63 | B cell differentiation |
| GO:0030100 | 0.010 | 1.726 | 23 | 34 | 92 | regulation of endocytosis |
| GO:0035966 | 0.010 | 1.667 | 27 | 38 | 105 | response to topologically incorrect protein |
| GO:0042107 | 0.010 | 1.812 | 19 | 29 | 76 | cytokine metabolic process |
| GO:0051028 | 0.010 | 1.812 | 19 | 29 | 76 | mRNA transport |
| GO:0042254 | 0.010 | 1.831 | 19 | 28 | 73 | ribosome biogenesis |
| GO:0033619 | 0.010 | 2.442 | 8 | 15 | 33 | membrane protein proteolysis |
| GO:0060425 | 0.010 | 2.442 | 8 | 15 | 33 | lung morphogenesis |
| GO:0043542 | 0.010 | 1.731 | 23 | 33 | 89 | endothelial cell migration |
| GO:0035329 | 0.010 | 3.253 | 5 | 10 | 19 | hippo signaling cascade |
| GO:0045730 | 0.010 | 3.253 | 5 | 10 | 19 | respiratory burst |
| GO:0000302 | 0.010 | 1.633 | 29 | 40 | 112 | response to reactive oxygen species |
| GO:0007179 | 0.010 | 1.852 | 18 | 27 | 70 | transforming growth factor beta receptor signaling pathway |
| GO:0043901 | 0.011 | 2.143 | 11 | 19 | 45 | negative regulation of multi-organism process |
| GO:0001824 | 0.011 | 2.199 | 11 | 18 | 42 | blastocyst development |
| GO:0035335 | 0.011 | 2.199 | 11 | 18 | 42 | peptidyl-tyrosine dephosphorylation |
| GO:0061024 | 0.011 | 1.364 | 74 | 91 | 288 | membrane organization |
| GO:0032507 | 0.011 | 1.843 | 18 | 27 | 70 | maintenance of protein location in cell |
| GO:0045185 | 0.011 | 5.885 | 2 | 6 | 9 | maintenance of protein location |
| GO:0010035 | 0.011 | 1.373 | 70 | 87 | 274 | response to inorganic substance |
| GO:0002237 | 0.011 | 1.489 | 43 | 57 | 170 | response to molecule of bacterial origin |
| GO:0000082 | 0.011 | 1.717 | 23 | 33 | 90 | G1/S transition of mitotic cell cycle |
| GO:0002643 | 0.011 | 5.849 | 2 | 6 | 9 | regulation of tolerance induction |
| GO:0009113 | 0.011 | 5.849 | 2 | 6 | 9 | purine nucleobase biosynthetic process |
| GO:0009264 | 0.011 | 5.849 | 2 | 6 | 9 | deoxyribonucleotide catabolic process |
| GO:0046386 | 0.011 | 5.849 | 2 | 6 | 9 | deoxyribose phosphate catabolic process |
| GO:0046653 | 0.011 | 5.849 | 2 | 6 | 9 | tetrahydrofolate metabolic process |
| GO:0043065 | 0.011 | 1.465 | 47 | 61 | 185 | positive regulation of apoptotic process |
| GO:0009126 | 0.012 | 2.928 | 6 | 11 | 22 | purine nucleoside monophosphate metabolic process |
| GO:0034121 | 0.012 | 2.928 | 6 | 11 | 22 | regulation of toll-like receptor signaling pathway |
| GO:0001773 | 0.012 | 3.901 | 4 | 8 | 14 | myeloid dendritic cell activation |
| GO:0007160 | 0.012 | 1.580 | 31 | 43 | 123 | cell-matrix adhesion |
| GO:0032103 | 0.012 | 1.815 | 18 | 27 | 71 | positive regulation of response to external stimulus |
| GO:0023057 | 0.013 | 1.273 | 120 | 141 | 469 | negative regulation of signaling |
| GO:0000075 | 0.013 | 2.268 | 9 | 16 | 37 | cell cycle checkpoint |
| GO:0032570 | 0.013 | 2.703 | 6 | 12 | 25 | response to progesterone stimulus |
| GO:0042742 | 0.013 | 1.993 | 13 | 21 | 52 | defense response to bacterium |
| GO:0016072 | 0.013 | 1.955 | 14 | 22 | 55 | rRNA metabolic process |
| GO:0032270 | 0.013 | 1.235 | 157 | 181 | 616 | positive regulation of cellular protein metabolic process |
| GO:0002699 | 0.014 | 1.717 | 21 | 31 | 84 | positive regulation of immune effector process |
| GO:0009100 | 0.014 | 1.412 | 54 | 69 | 213 | glycoprotein metabolic process |
| GO:2000377 | 0.014 | 2.319 | 9 | 15 | 34 | regulation of reactive oxygen species metabolic process |
| GO:0007162 | 0.014 | 1.816 | 17 | 26 | 68 | negative regulation of cell adhesion |
| GO:0002666 | 0.014 | 7.309 | 2 | 5 | 7 | positive regulation of T cell tolerance induction |
| GO:0002827 | 0.014 | 7.309 | 2 | 5 | 7 | positive regulation of T-helper 1 type immune response |
| GO:0006268 | 0.014 | 7.309 | 2 | 5 | 7 | DNA unwinding involved in replication |
| GO:0010759 | 0.014 | 7.309 | 2 | 5 | 7 | positive regulation of macrophage chemotaxis |
| GO:0032495 | 0.014 | 7.309 | 2 | 5 | 7 | response to muramyl dipeptide |
| GO:0032727 | 0.014 | 7.309 | 2 | 5 | 7 | positive regulation of interferon-alpha production |
| GO:0072102 | 0.014 | 7.309 | 2 | 5 | 7 | glomerulus morphogenesis |
| GO:1900024 | 0.014 | 7.309 | 2 | 5 | 7 | regulation of substrate adhesion-dependent cell spreading |
| GO:0055093 | 0.014 | 3.292 | 4 | 9 | 17 | response to hyperoxia |
| GO:0072012 | 0.014 | 3.292 | 4 | 9 | 17 | glomerulus vasculature development |
| GO:0010564 | 0.014 | 1.472 | 42 | 55 | 166 | regulation of cell cycle process |
| GO:0050793 | 0.015 | 1.398 | 56 | 71 | 224 | regulation of developmental process |
| GO:0033674 | 0.015 | 1.328 | 79 | 96 | 310 | positive regulation of kinase activity |
| GO:0046638 | 0.016 | 4.112 | 3 | 7 | 12 | positive regulation of alpha-beta T cell differentiation |
| GO:0045063 | 0.016 | 4.095 | 3 | 7 | 12 | T-helper 1 cell differentiation |
| GO:0046135 | 0.016 | 4.095 | 3 | 7 | 12 | pyrimidine nucleoside catabolic process |
| GO:0046605 | 0.016 | 4.095 | 3 | 7 | 12 | regulation of centrosome cycle |
| GO:0050718 | 0.016 | 4.095 | 3 | 7 | 12 | positive regulation of interleukin-1 beta secretion |
| GO:0090322 | 0.016 | 4.095 | 3 | 7 | 12 | regulation of superoxide metabolic process |
| GO:2000351 | 0.016 | 4.095 | 3 | 7 | 12 | regulation of endothelial cell apoptotic process |
| GO:0031529 | 0.016 | 2.927 | 5 | 10 | 20 | ruffle organization |
| GO:0034394 | 0.016 | 2.927 | 5 | 10 | 20 | protein localization to cell surface |
| GO:0046131 | 0.016 | 2.927 | 5 | 10 | 20 | pyrimidine ribonucleoside metabolic process |
| GO:0002758 | 0.016 | Inf | 1 | 3 | 3 | innate immune response-activating signal transduction |
| GO:0050857 | 0.017 | Inf | 1 | 3 | 3 | positive regulation of antigen receptor-mediated signaling pathway |
| GO:0002248 | 0.017 | Inf | 1 | 3 | 3 | connective tissue replacement involved in inflammatory response wound healing |
| GO:0002513 | 0.017 | Inf | 1 | 3 | 3 | tolerance induction to self antigen |
| GO:0002540 | 0.017 | Inf | 1 | 3 | 3 | leukotriene production involved in inflammatory response |
| GO:0002693 | 0.017 | Inf | 1 | 3 | 3 | positive regulation of cellular extravasation |
| GO:0006041 | 0.017 | Inf | 1 | 3 | 3 | glucosamine metabolic process |
| GO:0018401 | 0.017 | Inf | 1 | 3 | 3 | peptidyl-proline hydroxylation to 4-hydroxy-L-proline |
| GO:0019322 | 0.017 | Inf | 1 | 3 | 3 | pentose biosynthetic process |
| GO:0019730 | 0.017 | Inf | 1 | 3 | 3 | antimicrobial humoral response |
| GO:0030223 | 0.017 | Inf | 1 | 3 | 3 | neutrophil differentiation |
| GO:0032070 | 0.017 | Inf | 1 | 3 | 3 | regulation of deoxyribonuclease activity |
| GO:0046813 | 0.017 | Inf | 1 | 3 | 3 | virion attachment, binding of host cell surface receptor |
| GO:0051088 | 0.017 | Inf | 1 | 3 | 3 | PMA-inducible membrane protein ectodomain proteolysis |
| GO:0051176 | 0.017 | Inf | 1 | 3 | 3 | positive regulation of sulfur metabolic process |
| GO:0060236 | 0.017 | Inf | 1 | 3 | 3 | regulation of mitotic spindle organization |
| GO:0060440 | 0.017 | Inf | 1 | 3 | 3 | trachea formation |
| GO:0060574 | 0.017 | Inf | 1 | 3 | 3 | intestinal epithelial cell maturation |
| GO:0070427 | 0.017 | Inf | 1 | 3 | 3 | nucleotide-binding oligomerization domain containing 1 signaling pathway |
| GO:0051101 | 0.017 | 1.924 | 14 | 21 | 53 | regulation of DNA binding |
| GO:0051648 | 0.017 | 1.924 | 14 | 21 | 53 | vesicle localization |
| GO:0006693 | 0.017 | 11.731 | 1 | 4 | 5 | prostaglandin metabolic process |
| GO:0045576 | 0.017 | 11.726 | 1 | 4 | 5 | mast cell activation |
| GO:0070372 | 0.017 | 2.697 | 6 | 11 | 23 | regulation of ERK1 and ERK2 cascade |
| GO:0050663 | 0.017 | 2.695 | 6 | 11 | 23 | cytokine secretion |
| GO:0000028 | 0.017 | 11.690 | 1 | 4 | 5 | ribosomal small subunit assembly |
| GO:0001768 | 0.017 | 11.690 | 1 | 4 | 5 | establishment of T cell polarity |
| GO:0002606 | 0.017 | 11.690 | 1 | 4 | 5 | positive regulation of dendritic cell antigen processing and presentation |
| GO:0006032 | 0.017 | 11.690 | 1 | 4 | 5 | chitin catabolic process |
| GO:0006678 | 0.017 | 11.690 | 1 | 4 | 5 | glucosylceramide metabolic process |
| GO:0022614 | 0.017 | 11.690 | 1 | 4 | 5 | membrane to membrane docking |
| GO:0051014 | 0.017 | 11.690 | 1 | 4 | 5 | actin filament severing |
| GO:0060510 | 0.017 | 11.690 | 1 | 4 | 5 | Type II pneumocyte differentiation |
| GO:0072104 | 0.017 | 11.690 | 1 | 4 | 5 | glomerular capillary formation |
| GO:0046849 | 0.017 | 1.954 | 13 | 20 | 50 | bone remodeling |
| GO:0071356 | 0.017 | 1.954 | 13 | 20 | 50 | cellular response to tumor necrosis factor |
| GO:0032722 | 0.017 | 2.683 | 6 | 11 | 23 | positive regulation of chemokine production |
| GO:0032760 | 0.017 | 2.683 | 6 | 11 | 23 | positive regulation of tumor necrosis factor production |
| GO:0060325 | 0.017 | 2.683 | 6 | 11 | 23 | face morphogenesis |
| GO:0032675 | 0.018 | 1.989 | 12 | 19 | 47 | regulation of interleukin-6 production |
| GO:0006261 | 0.018 | 2.396 | 7 | 13 | 29 | DNA-dependent DNA replication |
| GO:0006418 | 0.018 | 2.509 | 7 | 12 | 26 | tRNA aminoacylation for protein translation |
| GO:0001776 | 0.018 | 2.079 | 10 | 17 | 41 | leukocyte homeostasis |
| GO:0006508 | 0.018 | 1.235 | 140 | 161 | 549 | proteolysis |
| GO:0050715 | 0.018 | 2.075 | 10 | 17 | 41 | positive regulation of cytokine secretion |
| GO:0016447 | 0.019 | 2.379 | 7 | 13 | 29 | somatic recombination of immunoglobulin gene segments |
| GO:0002040 | 0.019 | 2.131 | 10 | 16 | 38 | sprouting angiogenesis |
| GO:0006611 | 0.019 | 2.131 | 10 | 16 | 38 | protein export from nucleus |
| GO:0032945 | 0.019 | 2.131 | 10 | 16 | 38 | negative regulation of mononuclear cell proliferation |
| GO:0051495 | 0.019 | 1.712 | 19 | 28 | 76 | positive regulation of cytoskeleton organization |
| GO:0061041 | 0.019 | 1.712 | 19 | 28 | 76 | regulation of wound healing |
| GO:0006412 | 0.019 | 2.056 | 10 | 17 | 42 | translation |
| GO:0050871 | 0.019 | 3.359 | 4 | 8 | 15 | positive regulation of B cell activation |
| GO:0032655 | 0.019 | 3.353 | 4 | 8 | 15 | regulation of interleukin-12 production |
| GO:0046824 | 0.019 | 1.823 | 15 | 23 | 60 | positive regulation of nucleocytoplasmic transport |
| GO:0034599 | 0.019 | 1.654 | 22 | 31 | 86 | cellular response to oxidative stress |
| GO:0001779 | 0.020 | 3.343 | 4 | 8 | 15 | natural killer cell differentiation |
| GO:0032673 | 0.020 | 3.343 | 4 | 8 | 15 | regulation of interleukin-4 production |
| GO:0032862 | 0.020 | 3.343 | 4 | 8 | 15 | activation of Rho GTPase activity |
| GO:0043304 | 0.020 | 3.343 | 4 | 8 | 15 | regulation of mast cell degranulation |
| GO:0008285 | 0.020 | 1.262 | 107 | 126 | 421 | negative regulation of cell proliferation |
| GO:0051345 | 0.020 | 1.342 | 65 | 80 | 258 | positive regulation of hydrolase activity |
| GO:0042100 | 0.021 | 4.417 | 3 | 6 | 10 | B cell proliferation |
| GO:0051092 | 0.021 | 1.668 | 20 | 29 | 80 | positive regulation of NF-kappaB transcription factor activity |
| GO:0032481 | 0.022 | 4.398 | 3 | 6 | 10 | positive regulation of type I interferon production |
| GO:0002377 | 0.022 | 1.891 | 13 | 20 | 51 | immunoglobulin production |
| GO:0030889 | 0.022 | 4.386 | 3 | 6 | 10 | negative regulation of B cell proliferation |
| GO:0048194 | 0.022 | 4.386 | 3 | 6 | 10 | Golgi vesicle budding |
| GO:0060390 | 0.022 | 4.386 | 3 | 6 | 10 | regulation of SMAD protein import into nucleus |
| GO:0060438 | 0.022 | 4.386 | 3 | 6 | 10 | trachea development |
| GO:0071621 | 0.022 | 4.386 | 3 | 6 | 10 | granulocyte chemotaxis |
| GO:2000406 | 0.022 | 4.386 | 3 | 6 | 10 | positive regulation of T cell migration |
| GO:0001782 | 0.022 | 2.926 | 5 | 9 | 18 | B cell homeostasis |
| GO:0006221 | 0.022 | 2.926 | 5 | 9 | 18 | pyrimidine nucleotide biosynthetic process |
| GO:0009168 | 0.022 | 2.926 | 5 | 9 | 18 | purine ribonucleoside monophosphate biosynthetic process |
| GO:0032653 | 0.022 | 2.926 | 5 | 9 | 18 | regulation of interleukin-10 production |
| GO:0043536 | 0.022 | 2.926 | 5 | 9 | 18 | positive regulation of blood vessel endothelial cell migration |
| GO:0070527 | 0.022 | 2.926 | 5 | 9 | 18 | platelet aggregation |
| GO:0071222 | 0.022 | 1.963 | 11 | 18 | 45 | cellular response to lipopolysaccharide |
| GO:0070374 | 0.023 | 1.759 | 16 | 24 | 64 | positive regulation of ERK1 and ERK2 cascade |
| GO:0006796 | 0.023 | 1.161 | 286 | 313 | 1131 | phosphate-containing compound metabolic process |
| GO:0070555 | 0.023 | 2.673 | 5 | 10 | 21 | response to interleukin-1 |
| GO:0060491 | 0.023 | 1.953 | 11 | 18 | 45 | regulation of cell projection assembly |
| GO:0072527 | 0.023 | 1.953 | 11 | 18 | 45 | pyrimidine-containing compound metabolic process |
| GO:0019538 | 0.024 | 1.188 | 201 | 224 | 827 | protein metabolic process |
| GO:0090316 | 0.024 | 1.685 | 19 | 27 | 74 | positive regulation of intracellular protein transport |
| GO:0042993 | 0.024 | 2.660 | 5 | 10 | 21 | positive regulation of transcription factor import into nucleus |
| GO:0048384 | 0.024 | 2.660 | 5 | 10 | 21 | retinoic acid receptor signaling pathway |
| GO:0050830 | 0.024 | 2.660 | 5 | 10 | 21 | defense response to Gram-positive bacterium |
| GO:0070301 | 0.024 | 1.992 | 11 | 17 | 42 | cellular response to hydrogen peroxide |
| GO:2000026 | 0.024 | 1.184 | 205 | 229 | 805 | regulation of multicellular organismal development |
| GO:0050777 | 0.024 | 2.042 | 10 | 16 | 39 | negative regulation of immune response |
| GO:0043588 | 0.024 | 2.038 | 10 | 16 | 39 | skin development |
| GO:0050918 | 0.024 | 2.038 | 10 | 16 | 39 | positive chemotaxis |
| GO:0002009 | 0.025 | 1.332 | 64 | 78 | 251 | morphogenesis of an epithelium |
| GO:0000722 | 0.025 | 2.476 | 6 | 11 | 24 | telomere maintenance via recombination |
| GO:0050766 | 0.025 | 2.476 | 6 | 11 | 24 | positive regulation of phagocytosis |
| GO:0002821 | 0.025 | 2.092 | 9 | 15 | 36 | positive regulation of adaptive immune response |
| GO:0006958 | 0.025 | 2.342 | 7 | 12 | 27 | complement activation, classical pathway |
| GO:0019752 | 0.025 | 1.217 | 142 | 162 | 556 | carboxylic acid metabolic process |
| GO:0033628 | 0.025 | 2.239 | 8 | 13 | 30 | regulation of cell adhesion mediated by integrin |
| GO:0051301 | 0.026 | 1.298 | 77 | 92 | 301 | cell division |
| GO:0043154 | 0.026 | 1.810 | 14 | 21 | 55 | negative regulation of cysteine-type endopeptidase activity involved in apoptotic process |
| GO:0002520 | 0.027 | 5.050 | 2 | 5 | 8 | immune system development |
| GO:0006911 | 0.027 | 3.412 | 3 | 7 | 13 | phagocytosis, engulfment |
| GO:0033158 | 0.027 | 3.412 | 3 | 7 | 13 | regulation of protein import into nucleus, translocation |
| GO:0009892 | 0.027 | 1.160 | 259 | 285 | 1016 | negative regulation of metabolic process |
| GO:0034622 | 0.027 | 1.273 | 88 | 104 | 345 | cellular macromolecular complex assembly |
| GO:0042035 | 0.027 | 1.716 | 17 | 24 | 65 | regulation of cytokine biosynthetic process |
| GO:0060485 | 0.028 | 1.487 | 31 | 41 | 122 | mesenchyme development |
| GO:0032200 | 0.028 | 1.856 | 13 | 19 | 49 | telomere organization |
| GO:0071260 | 0.028 | 1.856 | 13 | 19 | 49 | cellular response to mechanical stimulus |
| GO:0009913 | 0.028 | 1.650 | 19 | 27 | 75 | epidermal cell differentiation |
| GO:0071346 | 0.028 | 2.972 | 4 | 8 | 16 | cellular response to interferon-gamma |
| GO:0090068 | 0.028 | 1.453 | 35 | 45 | 136 | positive regulation of cell cycle process |
| GO:0044700 | 0.029 | 1.113 | 675 | 710 | 2676 | single organism signaling |
| GO:0007154 | 0.029 | 1.114 | 667 | 701 | 2646 | cell communication |
| GO:0006921 | 0.029 | 1.729 | 16 | 23 | 62 | cellular component disassembly involved in execution phase of apoptosis |
| GO:0033036 | 0.029 | 1.219 | 130 | 149 | 523 | macromolecule localization |
| GO:0050688 | 0.029 | 1.883 | 12 | 18 | 46 | regulation of defense response to virus |
| GO:0006903 | 0.029 | 4.892 | 2 | 5 | 8 | vesicle targeting |
| GO:0032880 | 0.029 | 1.393 | 44 | 55 | 172 | regulation of protein localization |
| GO:0042554 | 0.030 | 4.880 | 2 | 5 | 8 | superoxide anion generation |
| GO:0008631 | 0.030 | 4.872 | 2 | 5 | 8 | intrinsic apoptotic signaling pathway in response to oxidative stress |
| GO:0008635 | 0.030 | 4.872 | 2 | 5 | 8 | activation of cysteine-type endopeptidase activity involved in apoptotic process by cytochrome c |
| GO:0009130 | 0.030 | 4.872 | 2 | 5 | 8 | pyrimidine nucleoside monophosphate biosynthetic process |
| GO:0009219 | 0.030 | 4.872 | 2 | 5 | 8 | pyrimidine deoxyribonucleotide metabolic process |
| GO:0031579 | 0.030 | 4.872 | 2 | 5 | 8 | membrane raft organization |
| GO:0035457 | 0.030 | 4.872 | 2 | 5 | 8 | cellular response to interferon-alpha |
| GO:0043312 | 0.030 | 4.872 | 2 | 5 | 8 | neutrophil degranulation |
| GO:0046033 | 0.030 | 4.872 | 2 | 5 | 8 | AMP metabolic process |
| GO:0090162 | 0.030 | 4.872 | 2 | 5 | 8 | establishment of epithelial cell polarity |
| GO:0016192 | 0.030 | 1.207 | 144 | 163 | 566 | vesicle-mediated transport |
| GO:0002275 | 0.030 | 2.933 | 4 | 8 | 16 | myeloid cell activation involved in immune response |
| GO:0002444 | 0.030 | 2.933 | 4 | 8 | 16 | myeloid leukocyte mediated immunity |
| GO:0002828 | 0.030 | 2.931 | 4 | 8 | 16 | regulation of type 2 immune response |
| GO:0045785 | 0.030 | 1.769 | 14 | 21 | 56 | positive regulation of cell adhesion |
| GO:0006998 | 0.030 | 2.925 | 4 | 8 | 16 | nuclear envelope organization |
| GO:0051983 | 0.030 | 2.925 | 4 | 8 | 16 | regulation of chromosome segregation |
| GO:0061082 | 0.030 | 2.925 | 4 | 8 | 16 | myeloid leukocyte cytokine production |
| GO:0060249 | 0.031 | 1.425 | 38 | 48 | 147 | anatomical structure homeostasis |
| GO:1901565 | 0.032 | 1.215 | 130 | 148 | 508 | organonitrogen compound catabolic process |
| GO:0010741 | 0.032 | 1.509 | 27 | 36 | 106 | negative regulation of intracellular protein kinase cascade |
| GO:0030099 | 0.032 | 1.359 | 49 | 61 | 193 | myeloid cell differentiation |
| GO:0001913 | 0.033 | 2.633 | 5 | 9 | 19 | T cell mediated cytotoxicity |
| GO:0010574 | 0.033 | 2.633 | 5 | 9 | 19 | regulation of vascular endothelial growth factor production |
| GO:0032735 | 0.033 | 2.633 | 5 | 9 | 19 | positive regulation of interleukin-12 production |
| GO:0033280 | 0.033 | 2.633 | 5 | 9 | 19 | response to vitamin D |
| GO:0048260 | 0.033 | 2.633 | 5 | 9 | 19 | positive regulation of receptor-mediated endocytosis |
| GO:0018212 | 0.033 | 1.391 | 42 | 53 | 165 | peptidyl-tyrosine modification |
| GO:0050673 | 0.033 | 1.548 | 24 | 32 | 93 | epithelial cell proliferation |
| GO:0009888 | 0.033 | 1.173 | 198 | 220 | 780 | tissue development |
| GO:0048844 | 0.033 | 2.049 | 9 | 14 | 34 | artery morphogenesis |
| GO:0000165 | 0.033 | 1.238 | 104 | 120 | 406 | MAPK cascade |
| GO:0002703 | 0.033 | 1.616 | 19 | 27 | 76 | regulation of leukocyte mediated immunity |
| GO:0045930 | 0.034 | 2.114 | 8 | 13 | 31 | negative regulation of mitotic cell cycle |
| GO:0001738 | 0.034 | 2.438 | 6 | 10 | 22 | morphogenesis of a polarized epithelium |
| GO:0010543 | 0.034 | 2.438 | 6 | 10 | 22 | regulation of platelet activation |
| GO:0033003 | 0.034 | 2.438 | 6 | 10 | 22 | regulation of mast cell activation |
| GO:0009161 | 0.034 | 2.303 | 6 | 11 | 25 | ribonucleoside monophosphate metabolic process |
| GO:0000018 | 0.034 | 2.195 | 7 | 12 | 28 | regulation of DNA recombination |
| GO:0034644 | 0.034 | 2.195 | 7 | 12 | 28 | cellular response to UV |
| GO:0043038 | 0.034 | 2.195 | 7 | 12 | 28 | amino acid activation |
| GO:0051208 | 0.034 | 2.195 | 7 | 12 | 28 | sequestering of calcium ion |
| GO:2000241 | 0.035 | 1.389 | 41 | 52 | 162 | regulation of reproductive process |
| GO:0032319 | 0.035 | 1.685 | 16 | 23 | 63 | regulation of Rho GTPase activity |
| GO:0001817 | 0.036 | 1.442 | 33 | 42 | 129 | regulation of cytokine production |
| GO:0030323 | 0.036 | 1.450 | 32 | 41 | 124 | respiratory tube development |
| GO:0002822 | 0.036 | 1.701 | 15 | 22 | 60 | regulation of adaptive immune response based on somatic recombination of immune receptors built from immunoglobulin superfamily domains |
| GO:0006753 | 0.037 | 1.193 | 148 | 167 | 581 | nucleoside phosphate metabolic process |
| GO:0006658 | 0.038 | 3.508 | 3 | 6 | 11 | phosphatidylserine metabolic process |
| GO:0034616 | 0.038 | 3.508 | 3 | 6 | 11 | response to laminar fluid shear stress |
| GO:0048208 | 0.038 | 3.508 | 3 | 6 | 11 | COPII vesicle coating |
| GO:0002698 | 0.038 | 1.844 | 11 | 17 | 44 | negative regulation of immune effector process |
| GO:0031023 | 0.038 | 1.844 | 11 | 17 | 44 | microtubule organizing center organization |
| GO:0035239 | 0.038 | 1.323 | 55 | 67 | 216 | tube morphogenesis |
| GO:0044106 | 0.038 | 1.420 | 34 | 44 | 135 | cellular amine metabolic process |
| GO:0032886 | 0.038 | 1.709 | 15 | 21 | 57 | regulation of microtubule-based process |
| GO:0042102 | 0.039 | 1.880 | 10 | 16 | 41 | positive regulation of T cell proliferation |
| GO:0008645 | 0.039 | 1.583 | 20 | 27 | 77 | hexose transport |
| GO:0060402 | 0.039 | 1.636 | 17 | 24 | 67 | calcium ion transport into cytosol |
| GO:0002250 | 0.039 | 2.983 | 4 | 7 | 14 | adaptive immune response |
| GO:0050679 | 0.040 | 1.514 | 24 | 32 | 94 | positive regulation of epithelial cell proliferation |
| GO:0006260 | 0.040 | 1.463 | 28 | 37 | 112 | DNA replication |
| GO:0009057 | 0.040 | 1.412 | 35 | 44 | 138 | macromolecule catabolic process |
| GO:0001780 | 0.040 | 5.844 | 2 | 4 | 6 | neutrophil homeostasis |
| GO:0002328 | 0.040 | 5.844 | 2 | 4 | 6 | pro-B cell differentiation |
| GO:0006222 | 0.040 | 5.844 | 2 | 4 | 6 | UMP biosynthetic process |
| GO:0006537 | 0.040 | 5.844 | 2 | 4 | 6 | glutamate biosynthetic process |
| GO:0006563 | 0.040 | 5.844 | 2 | 4 | 6 | L-serine metabolic process |
| GO:0007182 | 0.040 | 5.844 | 2 | 4 | 6 | common-partner SMAD protein phosphorylation |
| GO:0009173 | 0.040 | 5.844 | 2 | 4 | 6 | pyrimidine ribonucleoside monophosphate metabolic process |
| GO:0009215 | 0.040 | 5.844 | 2 | 4 | 6 | purine deoxyribonucleoside triphosphate metabolic process |
| GO:0019883 | 0.040 | 5.844 | 2 | 4 | 6 | antigen processing and presentation of endogenous antigen |
| GO:0031077 | 0.040 | 5.844 | 2 | 4 | 6 | post-embryonic camera-type eye development |
| GO:0031659 | 0.040 | 5.844 | 2 | 4 | 6 | positive regulation of cyclin-dependent protein kinase activity involved in G1/S |
| GO:0032692 | 0.040 | 5.844 | 2 | 4 | 6 | negative regulation of interleukin-1 production |
| GO:0032930 | 0.040 | 5.844 | 2 | 4 | 6 | positive regulation of superoxide anion generation |
| GO:0035089 | 0.040 | 5.844 | 2 | 4 | 6 | establishment of apical/basal cell polarity |
| GO:0043089 | 0.040 | 5.844 | 2 | 4 | 6 | positive regulation of Cdc42 GTPase activity |
| GO:0043097 | 0.040 | 5.844 | 2 | 4 | 6 | pyrimidine nucleoside salvage |
| GO:0045084 | 0.040 | 5.844 | 2 | 4 | 6 | positive regulation of interleukin-12 biosynthetic process |
| GO:0045292 | 0.040 | 5.844 | 2 | 4 | 6 | mRNA cis splicing, via spliceosome |
| GO:0045618 | 0.040 | 5.844 | 2 | 4 | 6 | positive regulation of keratinocyte differentiation |
| GO:0046655 | 0.040 | 5.844 | 2 | 4 | 6 | folic acid metabolic process |
| GO:0050862 | 0.040 | 5.844 | 2 | 4 | 6 | positive regulation of T cell receptor signaling pathway |
| GO:0051103 | 0.040 | 5.844 | 2 | 4 | 6 | DNA ligation involved in DNA repair |
| GO:0051546 | 0.040 | 5.844 | 2 | 4 | 6 | keratinocyte migration |
| GO:0060426 | 0.040 | 5.844 | 2 | 4 | 6 | lung vasculature development |
| GO:0061154 | 0.040 | 5.844 | 2 | 4 | 6 | endothelial tube morphogenesis |
| GO:0071732 | 0.040 | 5.844 | 2 | 4 | 6 | cellular response to nitric oxide |
| GO:0071888 | 0.040 | 5.844 | 2 | 4 | 6 | macrophage apoptotic process |
| GO:2000501 | 0.040 | 5.844 | 2 | 4 | 6 | regulation of natural killer cell chemotaxis |
| GO:0031347 | 0.040 | 1.399 | 36 | 46 | 145 | regulation of defense response |
| GO:0006486 | 0.041 | 1.394 | 37 | 47 | 146 | protein glycosylation |
| GO:0055123 | 0.041 | 1.546 | 21 | 29 | 84 | digestive system development |
| GO:0030217 | 0.041 | 2.101 | 7 | 12 | 29 | T cell differentiation |
| GO:0043200 | 0.041 | 1.961 | 9 | 14 | 35 | response to amino acid stimulus |
| GO:0006766 | 0.041 | 1.644 | 16 | 23 | 64 | vitamin metabolic process |
| GO:0050789 | 0.042 | 1.120 | 591 | 619 | 2463 | regulation of biological process |
| GO:0051656 | 0.042 | 1.588 | 19 | 26 | 74 | establishment of organelle localization |
| GO:0002274 | 0.042 | 2.090 | 7 | 12 | 29 | myeloid leukocyte activation |
| GO:0006303 | 0.042 | 2.924 | 4 | 7 | 14 | double-strand break repair via nonhomologous end joining |
| GO:0006929 | 0.042 | 2.924 | 4 | 7 | 14 | substrate-dependent cell migration |
| GO:0009218 | 0.042 | 2.924 | 4 | 7 | 14 | pyrimidine ribonucleotide metabolic process |
| GO:0010761 | 0.042 | 2.924 | 4 | 7 | 14 | fibroblast migration |
| GO:0032728 | 0.042 | 2.924 | 4 | 7 | 14 | positive regulation of interferon-beta production |
| GO:0046622 | 0.042 | 2.924 | 4 | 7 | 14 | positive regulation of organ growth |
| GO:0032680 | 0.044 | 2.073 | 7 | 12 | 29 | regulation of tumor necrosis factor production |
| GO:0006968 | 0.045 | 1.757 | 12 | 18 | 48 | cellular defense response |
| GO:0007260 | 0.045 | 1.757 | 12 | 18 | 48 | tyrosine phosphorylation of STAT protein |
| GO:0048145 | 0.045 | 1.757 | 12 | 18 | 48 | regulation of fibroblast proliferation |
| GO:0050714 | 0.045 | 2.155 | 7 | 11 | 26 | positive regulation of protein secretion |
| GO:0010466 | 0.045 | 1.447 | 29 | 37 | 112 | negative regulation of peptidase activity |
| GO:0032637 | 0.045 | 2.066 | 7 | 12 | 29 | interleukin-8 production |
| GO:0006541 | 0.045 | 2.599 | 4 | 8 | 17 | glutamine metabolic process |
| GO:0042094 | 0.045 | 2.599 | 4 | 8 | 17 | interleukin-2 biosynthetic process |
| GO:0042558 | 0.045 | 2.599 | 4 | 8 | 17 | pteridine-containing compound metabolic process |
| GO:0050732 | 0.045 | 2.599 | 4 | 8 | 17 | negative regulation of peptidyl-tyrosine phosphorylation |
| GO:0051298 | 0.045 | 2.599 | 4 | 8 | 17 | centrosome duplication |
| GO:0055012 | 0.045 | 2.599 | 4 | 8 | 17 | ventricular cardiac muscle cell differentiation |
| GO:0060479 | 0.045 | 2.599 | 4 | 8 | 17 | lung cell differentiation |
| GO:0070741 | 0.045 | 2.599 | 4 | 8 | 17 | response to interleukin-6 |
| GO:0000087 | 0.045 | 1.301 | 57 | 69 | 225 | M phase of mitotic cell cycle |
| GO:0007264 | 0.045 | 1.244 | 84 | 98 | 330 | small GTPase mediated signal transduction |
| GO:0072330 | 0.045 | 1.430 | 30 | 39 | 119 | monocarboxylic acid biosynthetic process |
| GO:0043299 | 0.045 | 2.150 | 7 | 11 | 26 | leukocyte degranulation |
| GO:0032623 | 0.046 | 2.399 | 5 | 9 | 20 | interleukin-2 production |
| GO:0070085 | 0.046 | 1.380 | 38 | 47 | 147 | glycosylation |
| GO:0042345 | 0.046 | 2.146 | 7 | 11 | 26 | regulation of NF-kappaB import into nucleus |
| GO:0031668 | 0.046 | 1.490 | 24 | 32 | 95 | cellular response to extracellular stimulus |
| GO:0001937 | 0.046 | 2.393 | 5 | 9 | 20 | negative regulation of endothelial cell proliferation |
| GO:0006906 | 0.046 | 2.393 | 5 | 9 | 20 | vesicle fusion |
| GO:0043094 | 0.046 | 2.393 | 5 | 9 | 20 | cellular metabolic compound salvage |
| GO:0045124 | 0.046 | 2.393 | 5 | 9 | 20 | regulation of bone resorption |
| GO:0010827 | 0.046 | 1.663 | 15 | 21 | 58 | regulation of glucose transport |
| GO:0002279 | 0.046 | 2.250 | 6 | 10 | 23 | mast cell activation involved in immune response |
| GO:0002448 | 0.046 | 2.250 | 6 | 10 | 23 | mast cell mediated immunity |
| GO:0002762 | 0.046 | 2.250 | 6 | 10 | 23 | negative regulation of myeloid leukocyte differentiation |
| GO:0090218 | 0.046 | 2.250 | 6 | 10 | 23 | positive regulation of lipid kinase activity |
| GO:0017015 | 0.046 | 1.598 | 17 | 24 | 68 | regulation of transforming growth factor beta receptor signaling pathway |
| GO:0072006 | 0.046 | 1.598 | 17 | 24 | 68 | nephron development |
| GO:0051336 | 0.047 | 1.316 | 51 | 62 | 204 | regulation of hydrolase activity |
| GO:0050868 | 0.047 | 1.778 | 11 | 17 | 45 | negative regulation of T cell activation |
| GO:0046822 | 0.047 | 1.613 | 17 | 23 | 65 | regulation of nucleocytoplasmic transport |
| GO:0008643 | 0.048 | 1.518 | 22 | 29 | 85 | carbohydrate transport |
| GO:0051258 | 0.048 | 1.447 | 28 | 36 | 109 | protein polymerization |
| GO:0042180 | 0.048 | 1.352 | 42 | 52 | 165 | cellular ketone metabolic process |
| GO:0072376 | 0.049 | 1.674 | 14 | 20 | 55 | protein activation cascade |
| GO:0000280 | 0.050 | 1.296 | 56 | 67 | 219 | nuclear division |
