# Supplementary material for: Hidden Treasures in “Ancient” Microarrays: Gene-Expression Portrays Biology and Potential Resistance Pathways of Major Lung Cancer Subtypes and Normal Tissue
Source: Front Oncol. 2014 Sep 29;4:251. doi: 10.3389/fonc.2014.00251 (PMC4178426; doi:10.3389/fonc.2014.00251)
Supplement: Figure S1 — Clinically relevant differentially expressed pathways in each tissue type versus the rest. The pathway maps are arranged according to the scheme in the left-top corner. Red indicates overexpression in genes, dark green downregulation. In the Cell Cycle pathway, tumor suppressor genes were overexpressed in normal lung tissue while oncogenes and tumor driving cyclins were overexpressed in cancers. The gene thymidylate synthase (TYMS or TS, belonging to the pathway “one carbon pool by folate”) is relevant for tumor growth and is also a treatment target. Notably, TYMS was overexpressed only in the tumors that are generally refractory to the drug pemetrexed, as the squamous and the small-cell lung cancer. TYMS was not overexpressed in mesothelioma, but it is known that TYMS expression is highly variable in this cancer. In the ERBB pathway, the ERBB2/HER2 and ERBB3/HER3 were overexpressed in adenocarcinoma, while the ERBB4 was overexpressed in the carcinoids. [file Data_Sheet_1.ZIP › File S3.ZIP!/GO Analysis/Carcinoids vs Rest Enrichment Analysis/Overrepresented GO terms of Up-regulated Genes.html]

Gene to GO BP Conditional test for over-representation

| GOBPID | Pvalue | OddsRatio | ExpCount | Count | Size | Term |
| --- | --- | --- | --- | --- | --- | --- |
| GO:0035637 | <0.0001 | 1.900 | 207 | 290 | 571 | multicellular organismal signaling |
| GO:0007268 | <0.0001 | 1.919 | 152 | 215 | 421 | synaptic transmission |
| GO:0050877 | <0.0001 | 1.880 | 143 | 201 | 402 | neurological system process |
| GO:0007612 | <0.0001 | 3.274 | 27 | 48 | 74 | learning |
| GO:0044704 | <0.0001 | 4.946 | 14 | 28 | 38 | single-organism reproductive behavior |
| GO:0008542 | <0.0001 | 5.543 | 11 | 22 | 29 | visual learning |
| GO:0007626 | <0.0001 | 2.182 | 39 | 59 | 107 | locomotory behavior |
| GO:0042773 | <0.0001 | 4.233 | 12 | 24 | 34 | ATP synthesis coupled electron transport |
| GO:0003001 | <0.0001 | 1.651 | 94 | 125 | 260 | generation of a signal involved in cell-cell signaling |
| GO:0051969 | <0.0001 | 2.043 | 42 | 62 | 116 | regulation of transmission of nerve impulse |
| GO:0050890 | <0.0001 | 1.906 | 51 | 72 | 139 | cognition |
| GO:0007215 | <0.0001 | 5.295 | 9 | 18 | 24 | glutamate receptor signaling pathway |
| GO:0045333 | <0.0001 | 2.049 | 40 | 59 | 110 | cellular respiration |
| GO:0034220 | <0.0001 | 1.666 | 78 | 104 | 215 | ion transmembrane transport |
| GO:0007158 | <0.0001 | Inf | 3 | 8 | 8 | neuron cell-cell adhesion |
| GO:0007600 | <0.0001 | 1.554 | 93 | 120 | 258 | sensory perception |
| GO:0007601 | 0.001 | 1.798 | 49 | 68 | 135 | visual perception |
| GO:0006120 | 0.001 | 3.962 | 9 | 18 | 26 | mitochondrial electron transport, NADH to ubiquinone |
| GO:0048489 | 0.001 | 3.536 | 11 | 20 | 30 | synaptic vesicle transport |
| GO:0030534 | 0.001 | 2.247 | 25 | 38 | 68 | adult behavior |
| GO:0007610 | 0.001 | 1.397 | 146 | 177 | 403 | behavior |
| GO:0007417 | 0.001 | 1.754 | 50 | 68 | 138 | central nervous system development |
| GO:0007269 | 0.001 | 2.531 | 18 | 30 | 51 | neurotransmitter secretion |
| GO:0048699 | 0.001 | 1.271 | 282 | 322 | 777 | generation of neurons |
| GO:0060079 | 0.001 | 4.030 | 8 | 16 | 23 | regulation of excitatory postsynaptic membrane potential |
| GO:0035249 | 0.001 | 2.894 | 13 | 23 | 37 | synaptic transmission, glutamatergic |
| GO:0042384 | 0.001 | 3.082 | 12 | 21 | 33 | cilium assembly |
| GO:0007156 | 0.001 | 2.205 | 23 | 35 | 63 | homophilic cell adhesion |
| GO:0009187 | 0.002 | 1.768 | 42 | 58 | 116 | cyclic nucleotide metabolic process |
| GO:0007168 | 0.002 | 14.056 | 3 | 8 | 9 | receptor guanylyl cyclase signaling pathway |
| GO:0014047 | 0.002 | 5.286 | 6 | 12 | 16 | glutamate secretion |
| GO:0015992 | 0.002 | 2.299 | 19 | 30 | 53 | proton transport |
| GO:0048172 | 0.002 | Inf | 2 | 6 | 6 | regulation of short-term neuronal synaptic plasticity |
| GO:0051481 | 0.002 | Inf | 2 | 6 | 6 | reduction of cytosolic calcium ion concentration |
| GO:2000300 | 0.002 | Inf | 2 | 6 | 6 | regulation of synaptic vesicle exocytosis |
| GO:2000311 | 0.002 | Inf | 2 | 6 | 6 | regulation of alpha-amino-3-hydroxy-5-methyl-4-isoxazole propionate selective glutamate receptor activity |
| GO:0022900 | 0.002 | 1.843 | 34 | 48 | 94 | electron transport chain |
| GO:0017156 | 0.002 | 3.776 | 8 | 15 | 22 | calcium ion-dependent exocytosis |
| GO:0007187 | 0.002 | 1.767 | 39 | 54 | 108 | G-protein coupled receptor signaling pathway, coupled to cyclic nucleotide second messenger |
| GO:0015991 | 0.002 | 3.519 | 9 | 16 | 24 | ATP hydrolysis coupled proton transport |
| GO:0035176 | 0.002 | 3.519 | 9 | 16 | 24 | social behavior |
| GO:0030258 | 0.003 | 1.767 | 39 | 53 | 106 | lipid modification |
| GO:0048148 | 0.003 | 7.908 | 4 | 9 | 11 | behavioral response to cocaine |
| GO:0007399 | 0.003 | 1.253 | 249 | 283 | 700 | nervous system development |
| GO:0050796 | 0.003 | 1.706 | 41 | 56 | 114 | regulation of insulin secretion |
| GO:0071276 | 0.003 | 5.859 | 5 | 10 | 13 | cellular response to cadmium ion |
| GO:0007214 | 0.004 | 4.834 | 5 | 11 | 15 | gamma-aminobutyric acid signaling pathway |
| GO:0033555 | 0.004 | 2.290 | 17 | 26 | 46 | multicellular organismal response to stress |
| GO:0048169 | 0.005 | 3.517 | 8 | 14 | 21 | regulation of long-term neuronal synaptic plasticity |
| GO:0050808 | 0.005 | 2.012 | 22 | 33 | 62 | synapse organization |
| GO:0007218 | 0.005 | 2.129 | 19 | 29 | 53 | neuropeptide signaling pathway |
| GO:0048167 | 0.005 | 2.020 | 22 | 32 | 60 | regulation of synaptic plasticity |
| GO:0016568 | 0.005 | 1.381 | 102 | 123 | 281 | chromatin modification |
| GO:0043267 | 0.006 | 7.027 | 4 | 8 | 10 | negative regulation of potassium ion transport |
| GO:0045745 | 0.006 | 7.027 | 4 | 8 | 10 | positive regulation of G-protein coupled receptor protein signaling pathway |
| GO:0046856 | 0.006 | 7.027 | 4 | 8 | 10 | phosphatidylinositol dephosphorylation |
| GO:0060134 | 0.006 | 7.027 | 4 | 8 | 10 | prepulse inhibition |
| GO:0071867 | 0.006 | 7.027 | 4 | 8 | 10 | response to monoamine stimulus |
| GO:0007256 | 0.006 | Inf | 2 | 5 | 5 | activation of JNKK activity |
| GO:0014848 | 0.006 | Inf | 2 | 5 | 5 | urinary tract smooth muscle contraction |
| GO:0016081 | 0.006 | Inf | 2 | 5 | 5 | synaptic vesicle docking involved in exocytosis |
| GO:0016188 | 0.006 | Inf | 2 | 5 | 5 | synaptic vesicle maturation |
| GO:0019227 | 0.006 | Inf | 2 | 5 | 5 | neuronal action potential propagation |
| GO:0032463 | 0.006 | Inf | 2 | 5 | 5 | negative regulation of protein homooligomerization |
| GO:0070932 | 0.006 | Inf | 2 | 5 | 5 | histone H3 deacetylation |
| GO:0097120 | 0.006 | Inf | 2 | 5 | 5 | receptor localization to synapse |
| GO:0044057 | 0.007 | 1.317 | 132 | 155 | 364 | regulation of system process |
| GO:0021695 | 0.007 | 2.639 | 11 | 18 | 30 | cerebellar cortex development |
| GO:0021697 | 0.007 | 5.271 | 4 | 9 | 12 | cerebellar cortex formation |
| GO:0002791 | 0.008 | 1.579 | 45 | 59 | 125 | regulation of peptide secretion |
| GO:0006836 | 0.008 | 3.111 | 8 | 14 | 22 | neurotransmitter transport |
| GO:0021766 | 0.008 | 2.278 | 14 | 22 | 39 | hippocampus development |
| GO:0016579 | 0.009 | 2.310 | 13 | 21 | 37 | protein deubiquitination |
| GO:0009260 | 0.010 | 1.451 | 63 | 78 | 173 | ribonucleotide biosynthetic process |
| GO:0050905 | 0.011 | 1.883 | 22 | 31 | 60 | neuromuscular process |
| GO:0008343 | 0.011 | 10.535 | 3 | 6 | 7 | adult feeding behavior |
| GO:0014051 | 0.011 | 10.535 | 3 | 6 | 7 | gamma-aminobutyric acid secretion |
| GO:0035235 | 0.011 | 10.535 | 3 | 6 | 7 | ionotropic glutamate receptor signaling pathway |
| GO:0043064 | 0.011 | 10.535 | 3 | 6 | 7 | flagellum organization |
| GO:2000463 | 0.011 | 10.535 | 3 | 6 | 7 | positive regulation of excitatory postsynaptic membrane potential |
| GO:0048675 | 0.011 | 2.131 | 15 | 23 | 42 | axon extension |
| GO:0010975 | 0.012 | 1.453 | 60 | 74 | 164 | regulation of neuron projection development |
| GO:0048666 | 0.013 | 1.234 | 191 | 215 | 527 | neuron development |
| GO:0021692 | 0.014 | 6.146 | 3 | 7 | 9 | cerebellar Purkinje cell layer morphogenesis |
| GO:0051957 | 0.014 | 6.146 | 3 | 7 | 9 | positive regulation of amino acid transport |
| GO:0071294 | 0.014 | 6.146 | 3 | 7 | 9 | cellular response to zinc ion |
| GO:2000310 | 0.014 | 6.146 | 3 | 7 | 9 | regulation of N-methyl-D-aspartate selective glutamate receptor activity |
| GO:0007623 | 0.014 | 1.820 | 22 | 31 | 61 | circadian rhythm |
| GO:0007628 | 0.014 | 2.735 | 8 | 14 | 23 | adult walking behavior |
| GO:0006171 | 0.015 | 1.676 | 29 | 39 | 80 | cAMP biosynthetic process |
| GO:0072384 | 0.015 | 2.856 | 8 | 13 | 21 | organelle transport along microtubule |
| GO:0072329 | 0.015 | 1.761 | 24 | 33 | 66 | monocarboxylic acid catabolic process |
| GO:0002833 | 0.016 | 4.684 | 4 | 8 | 11 | positive regulation of response to biotic stimulus |
| GO:0006000 | 0.016 | 4.684 | 4 | 8 | 11 | fructose metabolic process |
| GO:0006198 | 0.016 | 4.684 | 4 | 8 | 11 | cAMP catabolic process |
| GO:0007004 | 0.016 | 4.684 | 4 | 8 | 11 | telomere maintenance via telomerase |
| GO:0007026 | 0.016 | 4.684 | 4 | 8 | 11 | negative regulation of microtubule depolymerization |
| GO:0008089 | 0.016 | 4.684 | 4 | 8 | 11 | anterograde axon cargo transport |
| GO:0032095 | 0.016 | 4.684 | 4 | 8 | 11 | regulation of response to food |
| GO:0042593 | 0.016 | 1.590 | 35 | 46 | 97 | glucose homeostasis |
| GO:0007622 | 0.016 | 3.013 | 7 | 12 | 19 | rhythmic behavior |
| GO:0030317 | 0.016 | 3.013 | 7 | 12 | 19 | sperm motility |
| GO:0060291 | 0.016 | 3.013 | 7 | 12 | 19 | long-term synaptic potentiation |
| GO:0021549 | 0.016 | 3.233 | 6 | 11 | 17 | cerebellum development |
| GO:0016054 | 0.016 | 1.456 | 53 | 66 | 146 | organic acid catabolic process |
| GO:0016486 | 0.016 | 3.221 | 6 | 11 | 17 | peptide hormone processing |
| GO:0031110 | 0.016 | 3.520 | 5 | 10 | 15 | regulation of microtubule polymerization or depolymerization |
| GO:0007274 | 0.016 | 3.952 | 5 | 9 | 13 | neuromuscular synaptic transmission |
| GO:0008206 | 0.017 | 2.261 | 12 | 18 | 32 | bile acid metabolic process |
| GO:0006754 | 0.017 | 2.037 | 15 | 22 | 41 | ATP biosynthetic process |
| GO:0030072 | 0.017 | 1.463 | 51 | 63 | 139 | peptide hormone secretion |
| GO:0016082 | 0.017 | Inf | 1 | 4 | 4 | synaptic vesicle priming |
| GO:0045475 | 0.017 | Inf | 1 | 4 | 4 | locomotor rhythm |
| GO:0046951 | 0.017 | Inf | 1 | 4 | 4 | ketone body biosynthetic process |
| GO:0060023 | 0.017 | Inf | 1 | 4 | 4 | soft palate development |
| GO:0070198 | 0.017 | Inf | 1 | 4 | 4 | protein localization to chromosome, telomeric region |
| GO:0070857 | 0.017 | Inf | 1 | 4 | 4 | regulation of bile acid biosynthetic process |
| GO:0046883 | 0.017 | 1.452 | 52 | 65 | 144 | regulation of hormone secretion |
| GO:0051971 | 0.018 | 1.913 | 17 | 25 | 48 | positive regulation of transmission of nerve impulse |
| GO:0032501 | 0.021 | 1.102 | 1331 | 1375 | 3664 | multicellular organismal process |
| GO:0030520 | 0.021 | 2.397 | 9 | 15 | 26 | intracellular estrogen receptor signaling pathway |
| GO:0042461 | 0.021 | 2.397 | 9 | 15 | 26 | photoreceptor cell development |
| GO:0045980 | 0.021 | 2.397 | 9 | 15 | 26 | negative regulation of nucleotide metabolic process |
| GO:0015833 | 0.023 | 1.420 | 54 | 66 | 148 | peptide transport |
| GO:0046942 | 0.023 | 1.404 | 57 | 70 | 158 | carboxylic acid transport |
| GO:0030900 | 0.023 | 1.355 | 71 | 85 | 196 | forebrain development |
| GO:0030815 | 0.023 | 2.461 | 9 | 14 | 24 | negative regulation of cAMP metabolic process |
| GO:0030902 | 0.024 | 1.570 | 32 | 41 | 87 | hindbrain development |
| GO:0033762 | 0.025 | 2.110 | 12 | 18 | 33 | response to glucagon stimulus |
| GO:0071870 | 0.026 | 8.785 | 2 | 5 | 6 | cellular response to catecholamine stimulus |
| GO:0002855 | 0.026 | 8.776 | 2 | 5 | 6 | regulation of natural killer cell mediated immune response to tumor cell |
| GO:0002860 | 0.026 | 8.776 | 2 | 5 | 6 | positive regulation of natural killer cell mediated cytotoxicity directed against tumor cell target |
| GO:0006703 | 0.026 | 8.776 | 2 | 5 | 6 | estrogen biosynthetic process |
| GO:0006895 | 0.026 | 8.776 | 2 | 5 | 6 | Golgi to endosome transport |
| GO:0014898 | 0.026 | 8.776 | 2 | 5 | 6 | cardiac muscle hypertrophy in response to stress |
| GO:0016098 | 0.026 | 8.776 | 2 | 5 | 6 | monoterpenoid metabolic process |
| GO:0016559 | 0.026 | 8.776 | 2 | 5 | 6 | peroxisome fission |
| GO:0021819 | 0.026 | 8.776 | 2 | 5 | 6 | layer formation in cerebral cortex |
| GO:0032230 | 0.026 | 8.776 | 2 | 5 | 6 | positive regulation of synaptic transmission, GABAergic |
| GO:0042428 | 0.026 | 8.776 | 2 | 5 | 6 | serotonin metabolic process |
| GO:0043951 | 0.026 | 8.776 | 2 | 5 | 6 | negative regulation of cAMP-mediated signaling |
| GO:0051583 | 0.026 | 8.776 | 2 | 5 | 6 | dopamine uptake involved in synaptic transmission |
| GO:0071157 | 0.026 | 8.776 | 2 | 5 | 6 | negative regulation of cell cycle arrest |
| GO:0090129 | 0.026 | 8.776 | 2 | 5 | 6 | positive regulation of synapse maturation |
| GO:0090493 | 0.026 | 8.776 | 2 | 5 | 6 | catecholamine uptake |
| GO:0001662 | 0.026 | 2.636 | 7 | 12 | 20 | behavioral fear response |
| GO:0031279 | 0.027 | 1.760 | 20 | 27 | 54 | regulation of cyclase activity |
| GO:0048813 | 0.027 | 1.760 | 20 | 27 | 54 | dendrite morphogenesis |
| GO:0051932 | 0.028 | 2.765 | 7 | 11 | 18 | synaptic transmission, GABAergic |
| GO:0007283 | 0.029 | 1.399 | 53 | 64 | 145 | spermatogenesis |
| GO:0022029 | 0.029 | 2.163 | 11 | 16 | 29 | telencephalon cell migration |
| GO:0007606 | 0.029 | 2.934 | 6 | 10 | 16 | sensory perception of chemical stimulus |
| GO:0048515 | 0.030 | 1.839 | 16 | 23 | 45 | spermatid differentiation |
| GO:0009083 | 0.030 | 2.928 | 6 | 10 | 16 | branched-chain amino acid catabolic process |
| GO:0015697 | 0.030 | 2.928 | 6 | 10 | 16 | quaternary ammonium group transport |
| GO:0046854 | 0.030 | 2.928 | 6 | 10 | 16 | phosphatidylinositol phosphorylation |
| GO:0051412 | 0.030 | 2.928 | 6 | 10 | 16 | response to corticosterone stimulus |
| GO:0048858 | 0.030 | 1.205 | 173 | 193 | 477 | cell projection morphogenesis |
| GO:0002834 | 0.031 | 5.266 | 3 | 6 | 8 | regulation of response to tumor cell |
| GO:0002839 | 0.031 | 5.266 | 3 | 6 | 8 | positive regulation of immune response to tumor cell |
| GO:0007191 | 0.031 | 5.266 | 3 | 6 | 8 | adenylate cyclase-activating dopamine receptor signaling pathway |
| GO:0021702 | 0.031 | 5.266 | 3 | 6 | 8 | cerebellar Purkinje cell differentiation |
| GO:0031115 | 0.031 | 5.266 | 3 | 6 | 8 | negative regulation of microtubule polymerization |
| GO:0032099 | 0.031 | 5.266 | 3 | 6 | 8 | negative regulation of appetite |
| GO:0034063 | 0.031 | 5.266 | 3 | 6 | 8 | stress granule assembly |
| GO:0045956 | 0.031 | 5.266 | 3 | 6 | 8 | positive regulation of calcium ion-dependent exocytosis |
| GO:0070989 | 0.031 | 5.266 | 3 | 6 | 8 | oxidative demethylation |
| GO:0086013 | 0.031 | 5.266 | 3 | 6 | 8 | membrane repolarization involved in regulation of cardiac muscle cell action potential |
| GO:0007608 | 0.031 | 3.161 | 5 | 9 | 14 | sensory perception of smell |
| GO:0061387 | 0.032 | 1.965 | 13 | 19 | 36 | regulation of extent of cell growth |
| GO:0048488 | 0.032 | 3.518 | 4 | 8 | 12 | synaptic vesicle endocytosis |
| GO:0046785 | 0.032 | 4.104 | 4 | 7 | 10 | microtubule polymerization |
| GO:0002717 | 0.032 | 3.512 | 4 | 8 | 12 | positive regulation of natural killer cell mediated immunity |
| GO:0014048 | 0.032 | 3.512 | 4 | 8 | 12 | regulation of glutamate secretion |
| GO:0021895 | 0.032 | 3.512 | 4 | 8 | 12 | cerebral cortex neuron differentiation |
| GO:0048668 | 0.032 | 3.512 | 4 | 8 | 12 | collateral sprouting |
| GO:0031112 | 0.032 | 4.097 | 4 | 7 | 10 | positive regulation of microtubule polymerization or depolymerization |
| GO:0042737 | 0.032 | 4.097 | 4 | 7 | 10 | drug catabolic process |
| GO:0060306 | 0.032 | 4.097 | 4 | 7 | 10 | regulation of membrane repolarization |
| GO:0009201 | 0.033 | 1.759 | 18 | 25 | 50 | ribonucleoside triphosphate biosynthetic process |
| GO:0021954 | 0.033 | 1.842 | 16 | 22 | 43 | central nervous system neuron development |
| GO:0035264 | 0.033 | 1.531 | 31 | 40 | 86 | multicellular organism growth |
| GO:0007193 | 0.035 | 1.978 | 12 | 18 | 34 | adenylate cyclase-inhibiting G-protein coupled receptor signaling pathway |
| GO:0051339 | 0.035 | 1.696 | 20 | 27 | 55 | regulation of lyase activity |
| GO:0021587 | 0.035 | 2.236 | 9 | 14 | 25 | cerebellum morphogenesis |
| GO:0042417 | 0.035 | 2.236 | 9 | 14 | 25 | dopamine metabolic process |
| GO:0030808 | 0.036 | 1.526 | 31 | 39 | 84 | regulation of nucleotide biosynthetic process |
| GO:0009145 | 0.036 | 1.759 | 17 | 24 | 48 | purine nucleoside triphosphate biosynthetic process |
| GO:0032868 | 0.038 | 1.315 | 70 | 82 | 192 | response to insulin stimulus |
| GO:0030803 | 0.038 | 2.284 | 8 | 13 | 23 | negative regulation of cyclic nucleotide biosynthetic process |
| GO:1900372 | 0.038 | 2.284 | 8 | 13 | 23 | negative regulation of purine nucleotide biosynthetic process |
| GO:0050772 | 0.038 | 1.992 | 12 | 17 | 32 | positive regulation of axonogenesis |
| GO:0006164 | 0.040 | 1.363 | 53 | 64 | 147 | purine nucleotide biosynthetic process |
| GO:0006661 | 0.040 | 1.570 | 25 | 33 | 70 | phosphatidylinositol biosynthetic process |
| GO:0044712 | 0.041 | 1.311 | 69 | 81 | 190 | single-organism catabolic process |
| GO:0016574 | 0.042 | 2.342 | 8 | 12 | 21 | histone ubiquitination |
| GO:0048278 | 0.042 | 2.342 | 8 | 12 | 21 | vesicle docking |
| GO:0007420 | 0.042 | 1.418 | 41 | 50 | 113 | brain development |
| GO:0009914 | 0.043 | 1.327 | 62 | 73 | 170 | hormone transport |
| GO:0010970 | 0.045 | 2.422 | 7 | 11 | 19 | microtubule-based transport |
| GO:0046928 | 0.045 | 2.420 | 7 | 11 | 19 | regulation of neurotransmitter secretion |
| GO:0007194 | 0.045 | 2.415 | 7 | 11 | 19 | negative regulation of adenylate cyclase activity |
| GO:0032098 | 0.048 | Inf | 1 | 3 | 3 | regulation of appetite |
| GO:0000012 | 0.048 | Inf | 1 | 3 | 3 | single strand break repair |
| GO:0002091 | 0.048 | Inf | 1 | 3 | 3 | negative regulation of receptor internalization |
| GO:0006122 | 0.048 | Inf | 1 | 3 | 3 | mitochondrial electron transport, ubiquinol to cytochrome c |
| GO:0006744 | 0.048 | Inf | 1 | 3 | 3 | ubiquinone biosynthetic process |
| GO:0007412 | 0.048 | Inf | 1 | 3 | 3 | axon target recognition |
| GO:0009822 | 0.048 | Inf | 1 | 3 | 3 | alkaloid catabolic process |
| GO:0010452 | 0.048 | Inf | 1 | 3 | 3 | histone H3-K36 methylation |
| GO:0010586 | 0.048 | Inf | 1 | 3 | 3 | miRNA metabolic process |
| GO:0010832 | 0.048 | Inf | 1 | 3 | 3 | negative regulation of myotube differentiation |
| GO:0016561 | 0.048 | Inf | 1 | 3 | 3 | protein import into peroxisome matrix, translocation |
| GO:0021853 | 0.048 | Inf | 1 | 3 | 3 | cerebral cortex GABAergic interneuron migration |
| GO:0031848 | 0.048 | Inf | 1 | 3 | 3 | protection from non-homologous end joining at telomere |
| GO:0032206 | 0.048 | Inf | 1 | 3 | 3 | positive regulation of telomere maintenance |
| GO:0033076 | 0.048 | Inf | 1 | 3 | 3 | isoquinoline alkaloid metabolic process |
| GO:0033210 | 0.048 | Inf | 1 | 3 | 3 | leptin-mediated signaling pathway |
| GO:0034047 | 0.048 | Inf | 1 | 3 | 3 | regulation of protein phosphatase type 2A activity |
| GO:0034983 | 0.048 | Inf | 1 | 3 | 3 | peptidyl-lysine deacetylation |
| GO:0035330 | 0.048 | Inf | 1 | 3 | 3 | regulation of hippo signaling cascade |
| GO:0042271 | 0.048 | Inf | 1 | 3 | 3 | susceptibility to natural killer cell mediated cytotoxicity |
| GO:0043049 | 0.048 | Inf | 1 | 3 | 3 | otic placode formation |
| GO:0045143 | 0.048 | Inf | 1 | 3 | 3 | homologous chromosome segregation |
| GO:0046271 | 0.048 | Inf | 1 | 3 | 3 | phenylpropanoid catabolic process |
| GO:0046952 | 0.048 | Inf | 1 | 3 | 3 | ketone body catabolic process |
| GO:0048840 | 0.048 | Inf | 1 | 3 | 3 | otolith development |
| GO:0051645 | 0.048 | Inf | 1 | 3 | 3 | Golgi localization |
| GO:0060083 | 0.048 | Inf | 1 | 3 | 3 | smooth muscle contraction involved in micturition |
| GO:0060160 | 0.048 | Inf | 1 | 3 | 3 | negative regulation of dopamine receptor signaling pathway |
| GO:0060297 | 0.048 | Inf | 1 | 3 | 3 | regulation of sarcomere organization |
| GO:0070669 | 0.048 | Inf | 1 | 3 | 3 | response to interleukin-2 |
| GO:0071218 | 0.048 | Inf | 1 | 3 | 3 | cellular response to misfolded protein |
| GO:0071872 | 0.048 | Inf | 1 | 3 | 3 | cellular response to epinephrine stimulus |
| GO:0072321 | 0.048 | Inf | 1 | 3 | 3 | chaperone-mediated protein transport |
| GO:0072383 | 0.048 | Inf | 1 | 3 | 3 | plus-end-directed vesicle transport along microtubule |
| GO:0097011 | 0.048 | Inf | 1 | 3 | 3 | cellular response to granulocyte macrophage colony-stimulating factor stimulus |
| GO:0097091 | 0.048 | Inf | 1 | 3 | 3 | synaptic vesicle clustering |
| GO:0097114 | 0.048 | Inf | 1 | 3 | 3 | N-methyl-D-aspartate receptor clustering |
| GO:2000171 | 0.048 | Inf | 1 | 3 | 3 | negative regulation of dendrite development |
| GO:0050807 | 0.048 | 1.861 | 13 | 18 | 35 | regulation of synapse organization |
| GO:0097061 | 0.049 | 2.509 | 6 | 10 | 17 | dendritic spine organization |
