# Supplementary material for: Hidden Treasures in “Ancient” Microarrays: Gene-Expression Portrays Biology and Potential Resistance Pathways of Major Lung Cancer Subtypes and Normal Tissue
Source: Front Oncol. 2014 Sep 29;4:251. doi: 10.3389/fonc.2014.00251 (PMC4178426; doi:10.3389/fonc.2014.00251)
Supplement: Figure S1 — Clinically relevant differentially expressed pathways in each tissue type versus the rest. The pathway maps are arranged according to the scheme in the left-top corner. Red indicates overexpression in genes, dark green downregulation. In the Cell Cycle pathway, tumor suppressor genes were overexpressed in normal lung tissue while oncogenes and tumor driving cyclins were overexpressed in cancers. The gene thymidylate synthase (TYMS or TS, belonging to the pathway “one carbon pool by folate”) is relevant for tumor growth and is also a treatment target. Notably, TYMS was overexpressed only in the tumors that are generally refractory to the drug pemetrexed, as the squamous and the small-cell lung cancer. TYMS was not overexpressed in mesothelioma, but it is known that TYMS expression is highly variable in this cancer. In the ERBB pathway, the ERBB2/HER2 and ERBB3/HER3 were overexpressed in adenocarcinoma, while the ERBB4 was overexpressed in the carcinoids. [file Data_Sheet_1.ZIP › File S3.ZIP!/GO Analysis/Carcinoids vs Rest Enrichment Analysis/Underrepresented GO terms of Differentially Expressed Genes.html]

Gene to GO BP Conditional test for under-representation

| GOBPID | Pvalue | OddsRatio | ExpCount | Count | Size | Term |
| --- | --- | --- | --- | --- | --- | --- |
| GO:1901796 | <0.0001 | 0.192 | 13 | 5 | 21 | regulation of signal transduction by p53 class mediator |
| GO:0007389 | 0.001 | 0.661 | 161 | 136 | 261 | pattern specification process |
| GO:0060986 | 0.001 | 0.175 | 11 | 4 | 18 | endocrine hormone secretion |
| GO:0007586 | 0.001 | 0.484 | 48 | 34 | 77 | digestion |
| GO:0035113 | 0.002 | 0.510 | 48 | 35 | 77 | embryonic appendage morphogenesis |
| GO:0042733 | 0.003 | 0.323 | 18 | 10 | 29 | embryonic digit morphogenesis |
| GO:0007076 | 0.003 | 0.077 | 6 | 1 | 9 | mitotic chromosome condensation |
| GO:0006073 | 0.003 | 0.455 | 33 | 23 | 54 | cellular glucan metabolic process |
| GO:0009952 | 0.003 | 0.620 | 87 | 71 | 141 | anterior/posterior pattern specification |
| GO:0045862 | 0.003 | 0.444 | 31 | 21 | 50 | positive regulation of proteolysis |
| GO:0006397 | 0.003 | 0.697 | 154 | 133 | 249 | mRNA processing |
| GO:0048562 | 0.003 | 0.635 | 96 | 79 | 155 | embryonic organ morphogenesis |
| GO:0045648 | 0.004 | 0.236 | 11 | 5 | 18 | positive regulation of erythrocyte differentiation |
| GO:0008406 | 0.004 | 0.629 | 87 | 71 | 140 | gonad development |
| GO:0008380 | 0.006 | 0.491 | 35 | 25 | 56 | RNA splicing |
| GO:0043928 | 0.006 | 0.224 | 9 | 4 | 15 | exonucleolytic nuclear-transcribed mRNA catabolic process involved in deadenylation-dependent decay |
| GO:0007548 | 0.006 | 0.680 | 115 | 98 | 186 | sex differentiation |
| GO:0007350 | 0.006 | 0.088 | 5 | 1 | 8 | blastoderm segmentation |
| GO:0008595 | 0.006 | 0.088 | 5 | 1 | 8 | anterior/posterior axis specification, embryo |
| GO:0030277 | 0.006 | 0.088 | 5 | 1 | 8 | maintenance of gastrointestinal epithelium |
| GO:0032276 | 0.006 | 0.088 | 5 | 1 | 8 | regulation of gonadotropin secretion |
| GO:0050884 | 0.006 | 0.088 | 5 | 1 | 8 | neuromuscular process controlling posture |
| GO:0048736 | 0.007 | 0.587 | 59 | 47 | 96 | appendage development |
| GO:0021983 | 0.007 | 0.419 | 23 | 15 | 37 | pituitary gland development |
| GO:0006366 | 0.007 | 0.842 | 620 | 584 | 1001 | transcription from RNA polymerase II promoter |
| GO:0040018 | 0.008 | 0.307 | 13 | 7 | 21 | positive regulation of multicellular organism growth |
| GO:0051353 | 0.008 | 0.361 | 17 | 10 | 27 | positive regulation of oxidoreductase activity |
| GO:0071277 | 0.008 | 0.361 | 17 | 10 | 27 | cellular response to calcium ion |
| GO:0048729 | 0.008 | 0.759 | 214 | 192 | 346 | tissue morphogenesis |
| GO:0006622 | 0.008 | <0.0001 | 3 | 0 | 5 | protein targeting to lysosome |
| GO:0010766 | 0.008 | <0.0001 | 3 | 0 | 5 | negative regulation of sodium ion transport |
| GO:0045578 | 0.008 | <0.0001 | 3 | 0 | 5 | negative regulation of B cell differentiation |
| GO:0070562 | 0.008 | <0.0001 | 3 | 0 | 5 | regulation of vitamin D receptor signaling pathway |
| GO:0007143 | 0.009 | 0.154 | 6 | 2 | 10 | female meiosis |
| GO:0060272 | 0.009 | 0.154 | 6 | 2 | 10 | embryonic skeletal joint morphogenesis |
| GO:0070328 | 0.009 | 0.154 | 6 | 2 | 10 | triglyceride homeostasis |
| GO:0072001 | 0.010 | 0.696 | 114 | 98 | 184 | renal system development |
| GO:0003203 | 0.011 | 0.205 | 7 | 3 | 12 | endocardial cushion morphogenesis |
| GO:0010507 | 0.011 | 0.205 | 7 | 3 | 12 | negative regulation of autophagy |
| GO:0030049 | 0.011 | 0.439 | 22 | 15 | 36 | muscle filament sliding |
| GO:0005978 | 0.011 | 0.430 | 21 | 14 | 34 | glycogen biosynthetic process |
| GO:0046888 | 0.011 | 0.430 | 21 | 14 | 34 | negative regulation of hormone secretion |
| GO:0002088 | 0.012 | 0.500 | 30 | 22 | 49 | lens development in camera-type eye |
| GO:0061136 | 0.012 | 0.500 | 30 | 22 | 49 | regulation of proteasomal protein catabolic process |
| GO:0009950 | 0.012 | 0.246 | 9 | 4 | 14 | dorsal/ventral axis specification |
| GO:0070252 | 0.012 | 0.496 | 29 | 21 | 47 | actin-mediated cell contraction |
| GO:0003156 | 0.013 | 0.398 | 17 | 11 | 28 | regulation of organ formation |
| GO:0033059 | 0.013 | 0.280 | 10 | 5 | 16 | cellular pigmentation |
| GO:0060395 | 0.013 | 0.280 | 10 | 5 | 16 | SMAD protein signal transduction |
| GO:0046546 | 0.013 | 0.613 | 57 | 46 | 92 | development of primary male sexual characteristics |
| GO:0009954 | 0.013 | 0.307 | 11 | 6 | 18 | proximal/distal pattern formation |
| GO:0030219 | 0.013 | 0.307 | 11 | 6 | 18 | megakaryocyte differentiation |
| GO:0016070 | 0.013 | 0.891 | 1363 | 1320 | 2204 | RNA metabolic process |
| GO:0010862 | 0.014 | 0.351 | 14 | 8 | 22 | positive regulation of pathway-restricted SMAD protein phosphorylation |
| GO:0010467 | 0.014 | 0.895 | 1545 | 1500 | 2497 | gene expression |
| GO:0007501 | 0.014 | 0.103 | 4 | 1 | 7 | mesodermal cell fate specification |
| GO:0021978 | 0.014 | 0.103 | 4 | 1 | 7 | telencephalon regionalization |
| GO:0032438 | 0.014 | 0.103 | 4 | 1 | 7 | melanosome organization |
| GO:0032464 | 0.014 | 0.103 | 4 | 1 | 7 | positive regulation of protein homooligomerization |
| GO:0001707 | 0.015 | 0.475 | 24 | 17 | 39 | mesoderm formation |
| GO:0033692 | 0.015 | 0.475 | 24 | 17 | 39 | cellular polysaccharide biosynthetic process |
| GO:0048546 | 0.015 | 0.475 | 24 | 17 | 39 | digestive tract morphogenesis |
| GO:0044060 | 0.019 | 0.175 | 6 | 2 | 9 | regulation of endocrine process |
| GO:0060541 | 0.019 | 0.688 | 85 | 73 | 138 | respiratory system development |
| GO:0043518 | 0.019 | 0.176 | 6 | 2 | 9 | negative regulation of DNA damage response, signal transduction by p53 class mediator |
| GO:0045080 | 0.019 | 0.176 | 6 | 2 | 9 | positive regulation of chemokine biosynthetic process |
| GO:0009889 | 0.020 | 0.895 | 1220 | 1181 | 1972 | regulation of biosynthetic process |
| GO:0008589 | 0.021 | 0.423 | 17 | 11 | 27 | regulation of smoothened signaling pathway |
| GO:0006449 | 0.021 | <0.0001 | 2 | 0 | 4 | regulation of translational termination |
| GO:0016973 | 0.021 | <0.0001 | 2 | 0 | 4 | poly(A)+ mRNA export from nucleus |
| GO:0034116 | 0.021 | <0.0001 | 2 | 0 | 4 | positive regulation of heterotypic cell-cell adhesion |
| GO:0034720 | 0.021 | <0.0001 | 2 | 0 | 4 | histone H3-K4 demethylation |
| GO:0035930 | 0.021 | <0.0001 | 2 | 0 | 4 | corticosteroid hormone secretion |
| GO:0036120 | 0.021 | <0.0001 | 2 | 0 | 4 | cellular response to platelet-derived growth factor stimulus |
| GO:0042758 | 0.021 | <0.0001 | 2 | 0 | 4 | long-chain fatty acid catabolic process |
| GO:0046882 | 0.021 | <0.0001 | 2 | 0 | 4 | negative regulation of follicle-stimulating hormone secretion |
| GO:0070487 | 0.021 | <0.0001 | 2 | 0 | 4 | monocyte aggregation |
| GO:0071168 | 0.021 | <0.0001 | 2 | 0 | 4 | protein localization to chromatin |
| GO:0071481 | 0.021 | <0.0001 | 2 | 0 | 4 | cellular response to X-ray |
| GO:0072520 | 0.021 | <0.0001 | 2 | 0 | 4 | seminiferous tubule development |
| GO:0090281 | 0.021 | <0.0001 | 2 | 0 | 4 | negative regulation of calcium ion import |
| GO:0003085 | 0.021 | 0.231 | 7 | 3 | 11 | negative regulation of systemic arterial blood pressure |
| GO:0030214 | 0.021 | 0.231 | 7 | 3 | 11 | hyaluronan catabolic process |
| GO:0043517 | 0.021 | 0.231 | 7 | 3 | 11 | positive regulation of DNA damage response, signal transduction by p53 class mediator |
| GO:0071773 | 0.021 | 0.231 | 7 | 3 | 11 | cellular response to BMP stimulus |
| GO:0016925 | 0.022 | 0.410 | 15 | 10 | 25 | protein sumoylation |
| GO:0044243 | 0.022 | 0.410 | 15 | 10 | 25 | multicellular organismal catabolic process |
| GO:0050433 | 0.022 | 0.410 | 15 | 10 | 25 | regulation of catecholamine secretion |
| GO:0035136 | 0.023 | 0.395 | 14 | 9 | 23 | forelimb morphogenesis |
| GO:0044238 | 0.023 | 0.906 | 3118 | 3076 | 5040 | primary metabolic process |
| GO:0030509 | 0.023 | 0.614 | 47 | 38 | 76 | BMP signaling pathway |
| GO:0001991 | 0.023 | 0.273 | 8 | 4 | 13 | regulation of systemic arterial blood pressure by circulatory renin-angiotensin |
| GO:0023019 | 0.023 | 0.273 | 8 | 4 | 13 | signal transduction involved in regulation of gene expression |
| GO:0060317 | 0.023 | 0.273 | 8 | 4 | 13 | cardiac epithelial to mesenchymal transition |
| GO:0071371 | 0.023 | 0.273 | 8 | 4 | 13 | cellular response to gonadotropin stimulus |
| GO:0007281 | 0.023 | 0.628 | 51 | 42 | 83 | germ cell development |
| GO:0046579 | 0.024 | 0.359 | 12 | 7 | 19 | positive regulation of Ras protein signal transduction |
| GO:0061418 | 0.024 | 0.359 | 12 | 7 | 19 | regulation of transcription from RNA polymerase II promoter in response to hypoxia |
| GO:0002076 | 0.024 | 0.308 | 9 | 5 | 15 | osteoblast development |
| GO:0006378 | 0.024 | 0.308 | 9 | 5 | 15 | mRNA polyadenylation |
| GO:0007263 | 0.024 | 0.308 | 9 | 5 | 15 | nitric oxide mediated signal transduction |
| GO:0034312 | 0.024 | 0.308 | 9 | 5 | 15 | diol biosynthetic process |
| GO:0034508 | 0.024 | 0.308 | 9 | 5 | 15 | centromere complex assembly |
| GO:0042423 | 0.024 | 0.308 | 9 | 5 | 15 | catecholamine biosynthetic process |
| GO:0046189 | 0.024 | 0.308 | 9 | 5 | 15 | phenol-containing compound biosynthetic process |
| GO:0003009 | 0.024 | 0.335 | 11 | 6 | 17 | skeletal muscle contraction |
| GO:0003170 | 0.024 | 0.335 | 11 | 6 | 17 | heart valve development |
| GO:0048568 | 0.024 | 0.636 | 53 | 44 | 86 | embryonic organ development |
| GO:0006940 | 0.025 | 0.492 | 22 | 16 | 36 | regulation of smooth muscle contraction |
| GO:0033993 | 0.025 | 0.820 | 277 | 257 | 448 | response to lipid |
| GO:0000375 | 0.025 | 0.716 | 95 | 83 | 154 | RNA splicing, via transesterification reactions |
| GO:0031328 | 0.027 | 0.869 | 573 | 546 | 927 | positive regulation of cellular biosynthetic process |
| GO:0030282 | 0.027 | 0.595 | 39 | 31 | 63 | bone mineralization |
| GO:0007369 | 0.027 | 0.654 | 58 | 48 | 93 | gastrulation |
| GO:0042633 | 0.027 | 0.570 | 33 | 26 | 54 | hair cycle |
| GO:0010557 | 0.030 | 0.868 | 531 | 505 | 858 | positive regulation of macromolecule biosynthetic process |
| GO:0019216 | 0.030 | 0.730 | 99 | 87 | 160 | regulation of lipid metabolic process |
| GO:0043583 | 0.032 | 0.699 | 75 | 65 | 122 | ear development |
| GO:0036294 | 0.032 | 0.614 | 41 | 33 | 66 | cellular response to decreased oxygen levels |
| GO:0048609 | 0.033 | 0.816 | 235 | 217 | 379 | multicellular organismal reproductive process |
| GO:0000052 | 0.033 | 0.123 | 4 | 1 | 6 | citrulline metabolic process |
| GO:0002739 | 0.033 | 0.123 | 4 | 1 | 6 | regulation of cytokine secretion involved in immune response |
| GO:0002922 | 0.033 | 0.123 | 4 | 1 | 6 | positive regulation of humoral immune response |
| GO:0010700 | 0.033 | 0.123 | 4 | 1 | 6 | negative regulation of norepinephrine secretion |
| GO:0014850 | 0.033 | 0.123 | 4 | 1 | 6 | response to muscle activity |
| GO:0021520 | 0.033 | 0.123 | 4 | 1 | 6 | spinal cord motor neuron cell fate specification |
| GO:0021554 | 0.033 | 0.123 | 4 | 1 | 6 | optic nerve development |
| GO:0042368 | 0.033 | 0.123 | 4 | 1 | 6 | vitamin D biosynthetic process |
| GO:0042756 | 0.033 | 0.123 | 4 | 1 | 6 | drinking behavior |
| GO:0048755 | 0.033 | 0.123 | 4 | 1 | 6 | branching morphogenesis of a nerve |
| GO:0048843 | 0.033 | 0.123 | 4 | 1 | 6 | negative regulation of axon extension involved in axon guidance |
| GO:0048880 | 0.033 | 0.123 | 4 | 1 | 6 | sensory system development |
| GO:0050872 | 0.033 | 0.123 | 4 | 1 | 6 | white fat cell differentiation |
| GO:0051532 | 0.033 | 0.123 | 4 | 1 | 6 | regulation of NFAT protein import into nucleus |
| GO:0055091 | 0.033 | 0.123 | 4 | 1 | 6 | phospholipid homeostasis |
| GO:0060136 | 0.033 | 0.123 | 4 | 1 | 6 | embryonic process involved in female pregnancy |
| GO:0060579 | 0.033 | 0.123 | 4 | 1 | 6 | ventral spinal cord interneuron fate commitment |
| GO:0060973 | 0.033 | 0.123 | 4 | 1 | 6 | cell migration involved in heart development |
| GO:0071242 | 0.033 | 0.123 | 4 | 1 | 6 | cellular response to ammonium ion |
| GO:0072666 | 0.033 | 0.123 | 4 | 1 | 6 | establishment of protein localization to vacuole |
| GO:0090280 | 0.033 | 0.123 | 4 | 1 | 6 | positive regulation of calcium ion import |
| GO:2000380 | 0.033 | 0.123 | 4 | 1 | 6 | regulation of mesoderm development |
| GO:0048592 | 0.033 | 0.677 | 62 | 53 | 101 | eye morphogenesis |
| GO:0019835 | 0.034 | 0.451 | 16 | 11 | 26 | cytolysis |
| GO:0031076 | 0.034 | 0.451 | 16 | 11 | 26 | embryonic camera-type eye development |
| GO:0043269 | 0.034 | 0.767 | 132 | 119 | 214 | regulation of ion transport |
| GO:0042446 | 0.034 | 0.565 | 30 | 23 | 48 | hormone biosynthetic process |
| GO:0001656 | 0.035 | 0.614 | 40 | 32 | 64 | metanephros development |
| GO:0000272 | 0.035 | 0.439 | 15 | 10 | 24 | polysaccharide catabolic process |
| GO:0045666 | 0.036 | 0.563 | 28 | 22 | 46 | positive regulation of neuron differentiation |
| GO:0044260 | 0.036 | 0.894 | 968 | 938 | 1545 | cellular macromolecule metabolic process |
| GO:0035108 | 0.037 | 0.630 | 43 | 35 | 69 | limb morphogenesis |
| GO:0005980 | 0.037 | 0.426 | 14 | 9 | 22 | glycogen catabolic process |
| GO:0021515 | 0.037 | 0.426 | 14 | 9 | 22 | cell differentiation in spinal cord |
| GO:0003044 | 0.038 | 0.518 | 22 | 16 | 35 | regulation of systemic arterial blood pressure mediated by a chemical signal |
| GO:0006369 | 0.038 | 0.518 | 22 | 16 | 35 | termination of RNA polymerase II transcription |
| GO:0000910 | 0.038 | 0.632 | 43 | 35 | 69 | cytokinesis |
| GO:0001942 | 0.039 | 0.592 | 33 | 26 | 53 | hair follicle development |
| GO:0022404 | 0.039 | 0.592 | 33 | 26 | 53 | molting cycle process |
| GO:0061458 | 0.039 | 0.765 | 122 | 110 | 198 | reproductive system development |
| GO:0030318 | 0.039 | 0.410 | 12 | 8 | 20 | melanocyte differentiation |
| GO:0060571 | 0.039 | 0.410 | 12 | 8 | 20 | morphogenesis of an epithelial fold |
| GO:0001542 | 0.039 | 0.205 | 5 | 2 | 8 | ovulation from ovarian follicle |
| GO:0002438 | 0.039 | 0.205 | 5 | 2 | 8 | acute inflammatory response to antigenic stimulus |
| GO:0006379 | 0.039 | 0.205 | 5 | 2 | 8 | mRNA cleavage |
| GO:0006527 | 0.039 | 0.205 | 5 | 2 | 8 | arginine catabolic process |
| GO:0018298 | 0.039 | 0.205 | 5 | 2 | 8 | protein-chromophore linkage |
| GO:0034372 | 0.039 | 0.205 | 5 | 2 | 8 | very-low-density lipoprotein particle remodeling |
| GO:0042473 | 0.039 | 0.205 | 5 | 2 | 8 | outer ear morphogenesis |
| GO:0045651 | 0.039 | 0.205 | 5 | 2 | 8 | positive regulation of macrophage differentiation |
| GO:0045948 | 0.039 | 0.205 | 5 | 2 | 8 | positive regulation of translational initiation |
| GO:0060218 | 0.039 | 0.205 | 5 | 2 | 8 | hematopoietic stem cell differentiation |
| GO:0060513 | 0.039 | 0.205 | 5 | 2 | 8 | prostatic bud formation |
| GO:0045776 | 0.040 | 0.391 | 11 | 7 | 18 | negative regulation of blood pressure |
| GO:0003071 | 0.041 | 0.391 | 11 | 7 | 18 | renal system process involved in regulation of systemic arterial blood pressure |
| GO:0045932 | 0.041 | 0.391 | 11 | 7 | 18 | negative regulation of muscle contraction |
| GO:0045940 | 0.041 | 0.391 | 11 | 7 | 18 | positive regulation of steroid metabolic process |
| GO:0071312 | 0.041 | 0.391 | 11 | 7 | 18 | cellular response to alkaloid |
| GO:0090257 | 0.042 | 0.699 | 66 | 57 | 107 | regulation of muscle system process |
| GO:0033687 | 0.042 | 0.369 | 10 | 6 | 16 | osteoblast proliferation |
| GO:0050891 | 0.042 | 0.369 | 10 | 6 | 16 | multicellular organismal water homeostasis |
| GO:0055008 | 0.042 | 0.559 | 26 | 20 | 42 | cardiac muscle tissue morphogenesis |
| GO:0007141 | 0.042 | 0.264 | 6 | 3 | 10 | male meiosis I |
| GO:0007603 | 0.042 | 0.264 | 6 | 3 | 10 | phototransduction, visible light |
| GO:0032435 | 0.042 | 0.264 | 6 | 3 | 10 | negative regulation of proteasomal ubiquitin-dependent protein catabolic process |
| GO:0060065 | 0.042 | 0.264 | 6 | 3 | 10 | uterus development |
| GO:0071305 | 0.042 | 0.264 | 6 | 3 | 10 | cellular response to vitamin D |
| GO:0086005 | 0.042 | 0.264 | 6 | 3 | 10 | regulation of ventricular cardiac muscle cell action potential |
| GO:0030278 | 0.043 | 0.714 | 74 | 64 | 119 | regulation of ossification |
| GO:0032689 | 0.043 | 0.342 | 9 | 5 | 14 | negative regulation of interferon-gamma production |
| GO:0035809 | 0.043 | 0.342 | 9 | 5 | 14 | regulation of urine volume |
| GO:0060216 | 0.043 | 0.342 | 9 | 5 | 14 | definitive hemopoiesis |
| GO:1901576 | 0.043 | 0.920 | 1864 | 1828 | 3014 | organic substance biosynthetic process |
| GO:0007413 | 0.043 | 0.308 | 7 | 4 | 12 | axonal fasciculation |
| GO:0015874 | 0.043 | 0.308 | 7 | 4 | 12 | norepinephrine transport |
| GO:0034375 | 0.043 | 0.308 | 7 | 4 | 12 | high-density lipoprotein particle remodeling |
| GO:0045540 | 0.043 | 0.308 | 7 | 4 | 12 | regulation of cholesterol biosynthetic process |
| GO:0048016 | 0.043 | 0.308 | 7 | 4 | 12 | inositol phosphate-mediated signaling |
| GO:0051569 | 0.043 | 0.308 | 7 | 4 | 12 | regulation of histone H3-K4 methylation |
| GO:0035239 | 0.043 | 0.780 | 134 | 121 | 216 | tube morphogenesis |
| GO:0042311 | 0.045 | 0.590 | 30 | 24 | 49 | vasodilation |
| GO:0016049 | 0.045 | 0.800 | 163 | 149 | 263 | cell growth |
| GO:2001141 | 0.045 | 0.907 | 1015 | 985 | 1641 | regulation of RNA biosynthetic process |
| GO:0010558 | 0.046 | 0.867 | 408 | 387 | 659 | negative regulation of macromolecule biosynthetic process |
| GO:0031128 | 0.046 | 0.500 | 18 | 13 | 29 | developmental induction |
| GO:0007498 | 0.047 | 0.633 | 39 | 32 | 63 | mesoderm development |
| GO:0048762 | 0.048 | 0.703 | 64 | 55 | 103 | mesenchymal cell differentiation |
| GO:0001658 | 0.048 | 0.589 | 29 | 23 | 47 | branching involved in ureteric bud morphogenesis |
| GO:0045682 | 0.049 | 0.492 | 17 | 12 | 27 | regulation of epidermis development |
| GO:0060042 | 0.049 | 0.492 | 17 | 12 | 27 | retina morphogenesis in camera-type eye |
| GO:0090130 | 0.049 | 0.492 | 17 | 12 | 27 | tissue migration |
| GO:0045892 | 0.050 | 0.860 | 346 | 327 | 559 | negative regulation of transcription, DNA-dependent |
| GO:0060349 | 0.050 | 0.614 | 33 | 27 | 54 | bone morphogenesis |
