# Supplementary material for: Hidden Treasures in “Ancient” Microarrays: Gene-Expression Portrays Biology and Potential Resistance Pathways of Major Lung Cancer Subtypes and Normal Tissue
Source: Front Oncol. 2014 Sep 29;4:251. doi: 10.3389/fonc.2014.00251 (PMC4178426; doi:10.3389/fonc.2014.00251)
Supplement: Figure S1 — Clinically relevant differentially expressed pathways in each tissue type versus the rest. The pathway maps are arranged according to the scheme in the left-top corner. Red indicates overexpression in genes, dark green downregulation. In the Cell Cycle pathway, tumor suppressor genes were overexpressed in normal lung tissue while oncogenes and tumor driving cyclins were overexpressed in cancers. The gene thymidylate synthase (TYMS or TS, belonging to the pathway “one carbon pool by folate”) is relevant for tumor growth and is also a treatment target. Notably, TYMS was overexpressed only in the tumors that are generally refractory to the drug pemetrexed, as the squamous and the small-cell lung cancer. TYMS was not overexpressed in mesothelioma, but it is known that TYMS expression is highly variable in this cancer. In the ERBB pathway, the ERBB2/HER2 and ERBB3/HER3 were overexpressed in adenocarcinoma, while the ERBB4 was overexpressed in the carcinoids. [file Data_Sheet_1.ZIP › File S3.ZIP!/GO Analysis/Carcinoids vs Rest Enrichment Analysis/Underrepresented GO terms of Down-regulated Genes.html]

Gene to GO BP Conditional test for under-representation

| GOBPID | Pvalue | OddsRatio | ExpCount | Count | Size | Term |
| --- | --- | --- | --- | --- | --- | --- |
| GO:0035637 | <0.0001 | 0.413 | 146 | 74 | 571 | multicellular organismal signaling |
| GO:0050877 | <0.0001 | 0.407 | 91 | 45 | 346 | neurological system process |
| GO:0007268 | <0.0001 | 0.401 | 74 | 36 | 286 | synaptic transmission |
| GO:0034220 | <0.0001 | 0.411 | 51 | 25 | 199 | ion transmembrane transport |
| GO:0007600 | <0.0001 | 0.530 | 74 | 45 | 288 | sensory perception |
| GO:0031644 | <0.0001 | 0.470 | 45 | 25 | 178 | regulation of neurological system process |
| GO:0007187 | <0.0001 | 0.361 | 28 | 12 | 108 | G-protein coupled receptor signaling pathway, coupled to cyclic nucleotide second messenger |
| GO:0007601 | <0.0001 | 0.400 | 31 | 15 | 123 | visual perception |
| GO:0007283 | <0.0001 | 0.497 | 49 | 28 | 190 | spermatogenesis |
| GO:0046883 | <0.0001 | 0.438 | 37 | 19 | 144 | regulation of hormone secretion |
| GO:0007612 | <0.0001 | 0.302 | 19 | 7 | 74 | learning |
| GO:0006120 | <0.0001 | <0.0001 | 7 | 0 | 26 | mitochondrial electron transport, NADH to ubiquinone |
| GO:0042773 | 0.001 | 0.088 | 9 | 1 | 34 | ATP synthesis coupled electron transport |
| GO:0046928 | 0.001 | <0.0001 | 6 | 0 | 25 | regulation of neurotransmitter secretion |
| GO:0007399 | 0.001 | 0.780 | 286 | 243 | 1113 | nervous system development |
| GO:0002791 | 0.001 | 0.455 | 32 | 17 | 125 | regulation of peptide secretion |
| GO:0050890 | 0.001 | 0.485 | 35 | 20 | 139 | cognition |
| GO:0008213 | 0.001 | 0.207 | 11 | 3 | 45 | protein alkylation |
| GO:0007631 | 0.002 | 0.310 | 16 | 6 | 62 | feeding behavior |
| GO:0007276 | 0.002 | 0.623 | 67 | 47 | 264 | gamete generation |
| GO:0050796 | 0.002 | 0.472 | 29 | 16 | 114 | regulation of insulin secretion |
| GO:0007270 | 0.002 | 0.170 | 9 | 2 | 36 | neuron-neuron synaptic transmission |
| GO:0045776 | 0.002 | 0.104 | 7 | 1 | 29 | negative regulation of blood pressure |
| GO:0015844 | 0.002 | 0.264 | 12 | 4 | 48 | monoamine transport |
| GO:0023061 | 0.003 | 0.579 | 47 | 31 | 184 | signal release |
| GO:0007602 | 0.003 | 0.108 | 7 | 1 | 28 | phototransduction |
| GO:0009914 | 0.003 | 0.569 | 43 | 28 | 170 | hormone transport |
| GO:0007286 | 0.003 | 0.229 | 10 | 3 | 41 | spermatid development |
| GO:0009952 | 0.003 | 0.534 | 36 | 22 | 141 | anterior/posterior pattern specification |
| GO:0009187 | 0.003 | 0.497 | 30 | 17 | 116 | cyclic nucleotide metabolic process |
| GO:0007416 | 0.003 | 0.111 | 7 | 1 | 27 | synapse assembly |
| GO:0007389 | 0.004 | 0.649 | 67 | 48 | 261 | pattern specification process |
| GO:0015872 | 0.004 | <0.0001 | 5 | 0 | 19 | dopamine transport |
| GO:0030072 | 0.004 | 0.544 | 35 | 22 | 139 | peptide hormone secretion |
| GO:0015672 | 0.005 | 0.555 | 36 | 23 | 142 | monovalent inorganic cation transport |
| GO:0043414 | 0.005 | 0.369 | 16 | 7 | 62 | macromolecule methylation |
| GO:0048588 | 0.005 | 0.369 | 16 | 7 | 62 | developmental cell growth |
| GO:0018958 | 0.005 | <0.0001 | 5 | 0 | 18 | phenol-containing compound metabolic process |
| GO:0035113 | 0.006 | 0.433 | 20 | 10 | 77 | embryonic appendage morphogenesis |
| GO:0016054 | 0.006 | 0.569 | 37 | 24 | 146 | organic acid catabolic process |
| GO:0007189 | 0.006 | 0.249 | 10 | 3 | 38 | adenylate cyclase-activating G-protein coupled receptor signaling pathway |
| GO:0044704 | 0.006 | 0.249 | 10 | 3 | 38 | single-organism reproductive behavior |
| GO:0042417 | 0.006 | 0.121 | 6 | 1 | 25 | dopamine metabolic process |
| GO:0016486 | 0.007 | <0.0001 | 4 | 0 | 17 | peptide hormone processing |
| GO:0021954 | 0.007 | 0.298 | 11 | 4 | 43 | central nervous system neuron development |
| GO:0015992 | 0.007 | 0.256 | 9 | 3 | 37 | proton transport |
| GO:0035249 | 0.007 | 0.256 | 9 | 3 | 37 | synaptic transmission, glutamatergic |
| GO:0001975 | 0.008 | 0.126 | 6 | 1 | 24 | response to amphetamine |
| GO:0009755 | 0.008 | 0.126 | 6 | 1 | 24 | hormone-mediated signaling pathway |
| GO:0015991 | 0.008 | 0.126 | 6 | 1 | 24 | ATP hydrolysis coupled proton transport |
| GO:0055078 | 0.008 | 0.126 | 6 | 1 | 24 | sodium ion homeostasis |
| GO:0002027 | 0.008 | 0.338 | 12 | 5 | 48 | regulation of heart rate |
| GO:0045333 | 0.009 | 0.262 | 9 | 3 | 36 | cellular respiration |
| GO:0001964 | 0.009 | <0.0001 | 4 | 0 | 16 | startle response |
| GO:0015985 | 0.009 | <0.0001 | 4 | 0 | 16 | energy coupled proton transport, down electrochemical gradient |
| GO:0033059 | 0.009 | <0.0001 | 4 | 0 | 16 | cellular pigmentation |
| GO:0048665 | 0.009 | <0.0001 | 4 | 0 | 16 | neuron fate specification |
| GO:0017156 | 0.009 | 0.208 | 8 | 2 | 30 | calcium ion-dependent exocytosis |
| GO:0060079 | 0.009 | 0.208 | 8 | 2 | 30 | regulation of excitatory postsynaptic membrane potential |
| GO:0008344 | 0.009 | 0.371 | 14 | 6 | 53 | adult locomotory behavior |
| GO:0007626 | 0.009 | 0.396 | 15 | 7 | 58 | locomotory behavior |
| GO:0003015 | 0.009 | 0.556 | 30 | 19 | 118 | heart process |
| GO:0007200 | 0.010 | 0.346 | 12 | 5 | 47 | phospholipase C-activating G-protein coupled receptor signaling pathway |
| GO:0007156 | 0.010 | 0.422 | 16 | 8 | 63 | homophilic cell adhesion |
| GO:0009582 | 0.010 | 0.422 | 16 | 8 | 63 | detection of abiotic stimulus |
| GO:0032504 | 0.010 | 0.743 | 100 | 80 | 390 | multicellular organism reproduction |
| GO:0006754 | 0.011 | 0.314 | 10 | 4 | 41 | ATP biosynthetic process |
| GO:0050801 | 0.011 | 0.350 | 12 | 5 | 46 | ion homeostasis |
| GO:0008202 | 0.012 | 0.668 | 54 | 40 | 213 | steroid metabolic process |
| GO:0009584 | 0.012 | <0.0001 | 4 | 0 | 15 | detection of visible light |
| GO:0034312 | 0.012 | <0.0001 | 4 | 0 | 15 | diol biosynthetic process |
| GO:0042423 | 0.012 | <0.0001 | 4 | 0 | 15 | catecholamine biosynthetic process |
| GO:0046189 | 0.012 | <0.0001 | 4 | 0 | 15 | phenol-containing compound biosynthetic process |
| GO:0007586 | 0.012 | 0.483 | 20 | 11 | 77 | digestion |
| GO:0007269 | 0.013 | 0.385 | 13 | 6 | 51 | neurotransmitter secretion |
| GO:0021515 | 0.013 | 0.138 | 6 | 1 | 22 | cell differentiation in spinal cord |
| GO:0015833 | 0.013 | 0.617 | 38 | 26 | 148 | peptide transport |
| GO:0007193 | 0.014 | 0.281 | 9 | 3 | 34 | adenylate cyclase-inhibiting G-protein coupled receptor signaling pathway |
| GO:0072329 | 0.014 | 0.458 | 17 | 9 | 66 | monocarboxylic acid catabolic process |
| GO:0021533 | 0.016 | <0.0001 | 4 | 0 | 14 | cell differentiation in hindbrain |
| GO:0045822 | 0.016 | <0.0001 | 4 | 0 | 14 | negative regulation of heart contraction |
| GO:0021761 | 0.016 | 0.423 | 14 | 7 | 55 | limbic system development |
| GO:0032228 | 0.017 | 0.145 | 5 | 1 | 21 | regulation of synaptic transmission, GABAergic |
| GO:0040018 | 0.017 | 0.145 | 5 | 1 | 21 | positive regulation of multicellular organism growth |
| GO:0050879 | 0.017 | 0.145 | 5 | 1 | 21 | multicellular organismal movement |
| GO:0051953 | 0.017 | 0.145 | 5 | 1 | 21 | negative regulation of amine transport |
| GO:0006171 | 0.017 | 0.512 | 20 | 12 | 80 | cAMP biosynthetic process |
| GO:0034440 | 0.017 | 0.484 | 18 | 10 | 70 | lipid oxidation |
| GO:0060042 | 0.018 | 0.233 | 7 | 2 | 27 | retina morphogenesis in camera-type eye |
| GO:0006355 | 0.018 | 0.871 | 414 | 381 | 1622 | regulation of transcription, DNA-dependent |
| GO:0050767 | 0.019 | 0.734 | 76 | 60 | 296 | regulation of neurogenesis |
| GO:0030802 | 0.019 | 0.531 | 21 | 13 | 84 | regulation of cyclic nucleotide biosynthetic process |
| GO:1900371 | 0.019 | 0.531 | 21 | 13 | 84 | regulation of purine nucleotide biosynthetic process |
| GO:0044712 | 0.019 | 0.676 | 49 | 36 | 190 | single-organism catabolic process |
| GO:0050803 | 0.020 | 0.342 | 10 | 4 | 38 | regulation of synapse structure and activity |
| GO:0006338 | 0.020 | 0.475 | 16 | 9 | 64 | chromatin remodeling |
| GO:0007031 | 0.022 | 0.153 | 5 | 1 | 20 | peroxisome organization |
| GO:0009081 | 0.022 | 0.153 | 5 | 1 | 20 | branched-chain amino acid metabolic process |
| GO:0051568 | 0.022 | 0.153 | 5 | 1 | 20 | histone H3-K4 methylation |
| GO:0006590 | 0.022 | <0.0001 | 3 | 0 | 13 | thyroid hormone generation |
| GO:0007274 | 0.022 | <0.0001 | 3 | 0 | 13 | neuromuscular synaptic transmission |
| GO:0014059 | 0.022 | <0.0001 | 3 | 0 | 13 | regulation of dopamine secretion |
| GO:0035815 | 0.022 | <0.0001 | 3 | 0 | 13 | positive regulation of renal sodium excretion |
| GO:0042776 | 0.022 | <0.0001 | 3 | 0 | 13 | mitochondrial ATP synthesis coupled proton transport |
| GO:0071371 | 0.022 | <0.0001 | 3 | 0 | 13 | cellular response to gonadotropin stimulus |
| GO:0030534 | 0.022 | 0.241 | 7 | 2 | 26 | adult behavior |
| GO:0021675 | 0.022 | 0.415 | 12 | 6 | 48 | nerve development |
| GO:0030520 | 0.022 | 0.242 | 7 | 2 | 26 | intracellular estrogen receptor signaling pathway |
| GO:0042461 | 0.022 | 0.242 | 7 | 2 | 26 | photoreceptor cell development |
| GO:0001508 | 0.023 | 0.619 | 32 | 22 | 125 | regulation of action potential |
| GO:0006941 | 0.023 | 0.464 | 15 | 8 | 58 | striated muscle contraction |
| GO:0007218 | 0.023 | 0.442 | 14 | 7 | 53 | neuropeptide signaling pathway |
| GO:0022037 | 0.023 | 0.465 | 15 | 8 | 58 | metencephalon development |
| GO:0021983 | 0.024 | 0.352 | 9 | 4 | 37 | pituitary gland development |
| GO:0016569 | 0.025 | 0.684 | 47 | 35 | 183 | covalent chromatin modification |
| GO:0016568 | 0.025 | 0.447 | 13 | 7 | 52 | chromatin modification |
| GO:0060173 | 0.026 | 0.580 | 25 | 16 | 96 | limb development |
| GO:0070252 | 0.027 | 0.425 | 12 | 6 | 47 | actin-mediated cell contraction |
| GO:0043271 | 0.027 | 0.452 | 13 | 7 | 52 | negative regulation of ion transport |
| GO:0042445 | 0.027 | 0.592 | 26 | 17 | 100 | hormone metabolic process |
| GO:0042220 | 0.027 | 0.161 | 5 | 1 | 19 | response to cocaine |
| GO:0021517 | 0.028 | 0.162 | 5 | 1 | 19 | ventral spinal cord development |
| GO:0030317 | 0.028 | 0.162 | 5 | 1 | 19 | sperm motility |
| GO:0044062 | 0.028 | 0.162 | 5 | 1 | 19 | regulation of excretion |
| GO:0051963 | 0.028 | 0.162 | 5 | 1 | 19 | regulation of synapse assembly |
| GO:0048813 | 0.028 | 0.252 | 6 | 2 | 25 | dendrite morphogenesis |
| GO:0034762 | 0.028 | 0.564 | 22 | 14 | 86 | regulation of transmembrane transport |
| GO:0021587 | 0.028 | 0.253 | 6 | 2 | 25 | cerebellum morphogenesis |
| GO:0070509 | 0.028 | 0.253 | 6 | 2 | 25 | calcium ion import |
| GO:0050433 | 0.029 | <0.0001 | 3 | 0 | 12 | regulation of catecholamine secretion |
| GO:0030049 | 0.029 | 0.363 | 9 | 4 | 36 | muscle filament sliding |
| GO:0045665 | 0.029 | 0.363 | 9 | 4 | 36 | negative regulation of neuron differentiation |
| GO:0007413 | 0.029 | <0.0001 | 3 | 0 | 12 | axonal fasciculation |
| GO:0010669 | 0.029 | <0.0001 | 3 | 0 | 12 | epithelial structure maintenance |
| GO:0014821 | 0.029 | <0.0001 | 3 | 0 | 12 | phasic smooth muscle contraction |
| GO:0015874 | 0.029 | <0.0001 | 3 | 0 | 12 | norepinephrine transport |
| GO:0021697 | 0.029 | <0.0001 | 3 | 0 | 12 | cerebellar cortex formation |
| GO:0032462 | 0.029 | <0.0001 | 3 | 0 | 12 | regulation of protein homooligomerization |
| GO:0045494 | 0.029 | <0.0001 | 3 | 0 | 12 | photoreceptor cell maintenance |
| GO:0045954 | 0.029 | <0.0001 | 3 | 0 | 12 | positive regulation of natural killer cell mediated cytotoxicity |
| GO:0046839 | 0.029 | <0.0001 | 3 | 0 | 12 | phospholipid dephosphorylation |
| GO:0071600 | 0.029 | <0.0001 | 3 | 0 | 12 | otic vesicle morphogenesis |
| GO:0010975 | 0.030 | 0.621 | 29 | 20 | 113 | regulation of neuron projection development |
| GO:0050808 | 0.031 | 0.459 | 13 | 7 | 51 | synapse organization |
| GO:0051252 | 0.031 | 0.886 | 429 | 399 | 1680 | regulation of RNA metabolic process |
| GO:0006635 | 0.032 | 0.436 | 12 | 6 | 46 | fatty acid beta-oxidation |
| GO:0010976 | 0.032 | 0.462 | 13 | 7 | 51 | positive regulation of neuron projection development |
| GO:0045761 | 0.032 | 0.462 | 13 | 7 | 51 | regulation of adenylate cyclase activity |
| GO:0050804 | 0.032 | 0.610 | 26 | 18 | 103 | regulation of synaptic transmission |
| GO:0035137 | 0.032 | 0.323 | 8 | 3 | 30 | hindlimb morphogenesis |
| GO:0070988 | 0.032 | 0.323 | 8 | 3 | 30 | demethylation |
| GO:0051899 | 0.034 | 0.374 | 9 | 4 | 35 | membrane depolarization |
| GO:0021536 | 0.035 | 0.171 | 5 | 1 | 18 | diencephalon development |
| GO:0032104 | 0.035 | 0.264 | 6 | 2 | 24 | regulation of response to extracellular stimulus |
| GO:0035176 | 0.035 | 0.264 | 6 | 2 | 24 | social behavior |
| GO:0042596 | 0.035 | 0.264 | 6 | 2 | 24 | fear response |
| GO:0071616 | 0.035 | 0.264 | 6 | 2 | 24 | acyl-CoA biosynthetic process |
| GO:0016571 | 0.035 | 0.171 | 5 | 1 | 18 | histone methylation |
| GO:0043266 | 0.035 | 0.171 | 5 | 1 | 18 | regulation of potassium ion transport |
| GO:0009954 | 0.035 | 0.171 | 5 | 1 | 18 | proximal/distal pattern formation |
| GO:0031060 | 0.035 | 0.171 | 5 | 1 | 18 | regulation of histone methylation |
| GO:0048854 | 0.035 | 0.171 | 5 | 1 | 18 | brain morphogenesis |
| GO:0060986 | 0.035 | 0.171 | 5 | 1 | 18 | endocrine hormone secretion |
| GO:0022904 | 0.036 | 0.413 | 10 | 5 | 40 | respiratory electron transport chain |
| GO:0007018 | 0.036 | 0.581 | 21 | 14 | 84 | microtubule-based movement |
| GO:0070646 | 0.037 | 0.447 | 11 | 6 | 45 | protein modification by small protein removal |
| GO:0006351 | 0.039 | 0.717 | 49 | 38 | 187 | transcription, DNA-dependent |
| GO:0006582 | 0.039 | <0.0001 | 3 | 0 | 11 | melanin metabolic process |
| GO:0007320 | 0.039 | <0.0001 | 3 | 0 | 11 | insemination |
| GO:0009068 | 0.039 | <0.0001 | 3 | 0 | 11 | aspartate family amino acid catabolic process |
| GO:0022010 | 0.039 | <0.0001 | 3 | 0 | 11 | central nervous system myelination |
| GO:0032095 | 0.039 | <0.0001 | 3 | 0 | 11 | regulation of response to food |
| GO:0032098 | 0.039 | <0.0001 | 3 | 0 | 11 | regulation of appetite |
| GO:0043268 | 0.039 | <0.0001 | 3 | 0 | 11 | positive regulation of potassium ion transport |
| GO:0043949 | 0.039 | <0.0001 | 3 | 0 | 11 | regulation of cAMP-mediated signaling |
| GO:0044550 | 0.039 | <0.0001 | 3 | 0 | 11 | secondary metabolite biosynthetic process |
| GO:0048148 | 0.039 | <0.0001 | 3 | 0 | 11 | behavioral response to cocaine |
| GO:0051196 | 0.039 | <0.0001 | 3 | 0 | 11 | regulation of coenzyme metabolic process |
| GO:0051646 | 0.039 | <0.0001 | 3 | 0 | 11 | mitochondrion localization |
| GO:1900449 | 0.039 | <0.0001 | 3 | 0 | 11 | regulation of glutamate receptor signaling pathway |
| GO:0048511 | 0.040 | 0.673 | 35 | 26 | 138 | rhythmic process |
| GO:0008542 | 0.040 | 0.336 | 7 | 3 | 29 | visual learning |
| GO:0042733 | 0.040 | 0.336 | 7 | 3 | 29 | embryonic digit morphogenesis |
| GO:0048066 | 0.040 | 0.336 | 7 | 3 | 29 | developmental pigmentation |
| GO:0048814 | 0.040 | 0.336 | 7 | 3 | 29 | regulation of dendrite morphogenesis |
| GO:0007420 | 0.040 | 0.787 | 88 | 74 | 345 | brain development |
| GO:0015980 | 0.042 | 0.676 | 35 | 26 | 136 | energy derivation by oxidation of organic compounds |
| GO:0009712 | 0.042 | 0.388 | 9 | 4 | 34 | catechol-containing compound metabolic process |
| GO:0048168 | 0.042 | 0.388 | 9 | 4 | 34 | regulation of neuronal synaptic plasticity |
| GO:0032412 | 0.043 | 0.523 | 15 | 9 | 59 | regulation of ion transmembrane transporter activity |
| GO:0030182 | 0.043 | 0.838 | 155 | 137 | 603 | neuron differentiation |
| GO:0030814 | 0.043 | 0.581 | 20 | 13 | 78 | regulation of cAMP metabolic process |
| GO:0006873 | 0.043 | 0.816 | 117 | 101 | 458 | cellular ion homeostasis |
| GO:0021953 | 0.043 | 0.457 | 11 | 6 | 44 | central nervous system neuron differentiation |
| GO:0007423 | 0.044 | 0.780 | 78 | 65 | 306 | sensory organ development |
| GO:0003009 | 0.045 | 0.182 | 4 | 1 | 17 | skeletal muscle contraction |
| GO:0044060 | 0.045 | 0.182 | 4 | 1 | 17 | regulation of endocrine process |
| GO:0048488 | 0.045 | 0.182 | 4 | 1 | 17 | synaptic vesicle endocytosis |
| GO:0097061 | 0.045 | 0.182 | 4 | 1 | 17 | dendritic spine organization |
| GO:0010927 | 0.046 | 0.598 | 21 | 14 | 82 | cellular component assembly involved in morphogenesis |
| GO:0031326 | 0.047 | 0.902 | 497 | 469 | 1948 | regulation of cellular biosynthetic process |
| GO:0060560 | 0.048 | 0.619 | 23 | 16 | 91 | developmental growth involved in morphogenesis |
| GO:0001754 | 0.048 | 0.349 | 7 | 3 | 28 | eye photoreceptor cell differentiation |
| GO:0030516 | 0.048 | 0.349 | 7 | 3 | 28 | regulation of axon extension |
| GO:0042312 | 0.048 | 0.349 | 7 | 3 | 28 | regulation of vasodilation |
| GO:0046661 | 0.049 | 0.637 | 26 | 18 | 100 | male sex differentiation |
