# Supplementary material for: Hidden Treasures in “Ancient” Microarrays: Gene-Expression Portrays Biology and Potential Resistance Pathways of Major Lung Cancer Subtypes and Normal Tissue
Source: Front Oncol. 2014 Sep 29;4:251. doi: 10.3389/fonc.2014.00251 (PMC4178426; doi:10.3389/fonc.2014.00251)
Supplement: Figure S1 — Clinically relevant differentially expressed pathways in each tissue type versus the rest. The pathway maps are arranged according to the scheme in the left-top corner. Red indicates overexpression in genes, dark green downregulation. In the Cell Cycle pathway, tumor suppressor genes were overexpressed in normal lung tissue while oncogenes and tumor driving cyclins were overexpressed in cancers. The gene thymidylate synthase (TYMS or TS, belonging to the pathway “one carbon pool by folate”) is relevant for tumor growth and is also a treatment target. Notably, TYMS was overexpressed only in the tumors that are generally refractory to the drug pemetrexed, as the squamous and the small-cell lung cancer. TYMS was not overexpressed in mesothelioma, but it is known that TYMS expression is highly variable in this cancer. In the ERBB pathway, the ERBB2/HER2 and ERBB3/HER3 were overexpressed in adenocarcinoma, while the ERBB4 was overexpressed in the carcinoids. [file Data_Sheet_1.ZIP › File S3.ZIP!/GO Analysis/Carcinoids vs Rest Enrichment Analysis/Underrepresented GO terms of Up-regulated Genes.html]

Gene to GO BP Conditional test for under-representation

| GOBPID | Pvalue | OddsRatio | ExpCount | Count | Size | Term |
| --- | --- | --- | --- | --- | --- | --- |
| GO:0006614 | <0.0001 | 0.072 | 27 | 3 | 75 | SRP-dependent cotranslational protein targeting to membrane |
| GO:0051704 | <0.0001 | 0.620 | 360 | 270 | 989 | multi-organism process |
| GO:0072599 | <0.0001 | 0.093 | 28 | 4 | 78 | establishment of protein localization to endoplasmic reticulum |
| GO:0006415 | <0.0001 | 0.056 | 23 | 2 | 64 | translational termination |
| GO:0016032 | <0.0001 | 0.555 | 195 | 133 | 538 | viral reproduction |
| GO:0044703 | <0.0001 | 0.540 | 170 | 113 | 465 | multi-organism reproductive process |
| GO:0006413 | <0.0001 | 0.244 | 41 | 14 | 113 | translational initiation |
| GO:0050776 | <0.0001 | 0.390 | 71 | 36 | 192 | regulation of immune response |
| GO:0002696 | <0.0001 | 0.378 | 66 | 33 | 183 | positive regulation of leukocyte activation |
| GO:0009607 | <0.0001 | 0.510 | 125 | 79 | 341 | response to biotic stimulus |
| GO:0006414 | <0.0001 | 0.160 | 26 | 6 | 71 | translational elongation |
| GO:0019538 | <0.0001 | 0.636 | 263 | 200 | 707 | protein metabolic process |
| GO:2000145 | <0.0001 | 0.522 | 124 | 80 | 342 | regulation of cell motility |
| GO:0012501 | <0.0001 | 0.707 | 413 | 338 | 1137 | programmed cell death |
| GO:0002474 | <0.0001 | 0.234 | 31 | 10 | 84 | antigen processing and presentation of peptide antigen via MHC class I |
| GO:0034340 | <0.0001 | 0.189 | 22 | 6 | 61 | response to type I interferon |
| GO:0060337 | <0.0001 | 0.189 | 22 | 6 | 61 | type I interferon-mediated signaling pathway |
| GO:0016265 | <0.0001 | 0.743 | 457 | 387 | 1259 | death |
| GO:0080134 | <0.0001 | 0.648 | 204 | 155 | 562 | regulation of response to stress |
| GO:0006612 | <0.0001 | 0.342 | 40 | 18 | 109 | protein targeting to membrane |
| GO:0002479 | <0.0001 | 0.213 | 23 | 7 | 64 | antigen processing and presentation of exogenous peptide antigen via MHC class I, TAP-dependent |
| GO:0034341 | <0.0001 | 0.297 | 32 | 13 | 89 | response to interferon-gamma |
| GO:0019048 | <0.0001 | 0.548 | 104 | 69 | 285 | virus-host interaction |
| GO:0051272 | <0.0001 | 0.488 | 74 | 45 | 204 | positive regulation of cellular component movement |
| GO:0002684 | <0.0001 | 0.487 | 72 | 44 | 196 | positive regulation of immune system process |
| GO:0060333 | <0.0001 | 0.096 | 14 | 2 | 38 | interferon-gamma-mediated signaling pathway |
| GO:0000216 | <0.0001 | 0.256 | 25 | 9 | 70 | M/G1 transition of mitotic cell cycle |
| GO:0048729 | <0.0001 | 0.594 | 126 | 89 | 346 | tissue morphogenesis |
| GO:0060326 | <0.0001 | 0.408 | 48 | 25 | 131 | cell chemotaxis |
| GO:0032651 | <0.0001 | <0.0001 | 9 | 0 | 25 | regulation of interleukin-1 beta production |
| GO:0072331 | <0.0001 | 0.370 | 39 | 19 | 108 | signal transduction by p53 class mediator |
| GO:0072358 | <0.0001 | 0.680 | 217 | 170 | 596 | cardiovascular system development |
| GO:0006955 | <0.0001 | 0.492 | 68 | 42 | 181 | immune response |
| GO:0000184 | <0.0001 | 0.318 | 31 | 13 | 84 | nuclear-transcribed mRNA catabolic process, nonsense-mediated decay |
| GO:0051247 | <0.0001 | 0.698 | 242 | 194 | 667 | positive regulation of protein metabolic process |
| GO:0019083 | <0.0001 | 0.407 | 44 | 23 | 121 | viral transcription |
| GO:0001568 | <0.0001 | 0.620 | 137 | 100 | 376 | blood vessel development |
| GO:0000003 | <0.0001 | 0.755 | 402 | 342 | 1106 | reproduction |
| GO:0002429 | <0.0001 | 0.364 | 36 | 17 | 98 | immune response-activating cell surface receptor signaling pathway |
| GO:0070201 | <0.0001 | 0.549 | 87 | 58 | 240 | regulation of establishment of protein localization |
| GO:0042221 | <0.0001 | 0.805 | 755 | 679 | 2077 | response to chemical stimulus |
| GO:0033554 | <0.0001 | 0.728 | 298 | 246 | 818 | cellular response to stress |
| GO:0051169 | <0.0001 | 0.571 | 95 | 65 | 261 | nuclear transport |
| GO:0070663 | <0.0001 | 0.410 | 42 | 22 | 115 | regulation of leukocyte proliferation |
| GO:0019058 | <0.0001 | 0.506 | 66 | 41 | 181 | viral infectious cycle |
| GO:0051352 | <0.0001 | 0.262 | 22 | 8 | 61 | negative regulation of ligase activity |
| GO:0044403 | <0.0001 | 0.619 | 125 | 91 | 343 | symbiosis, encompassing mutualism through parasitism |
| GO:0043123 | <0.0001 | 0.373 | 35 | 17 | 96 | positive regulation of I-kappaB kinase/NF-kappaB cascade |
| GO:0050900 | <0.0001 | 0.444 | 48 | 27 | 131 | leukocyte migration |
| GO:0033365 | <0.0001 | 0.631 | 133 | 98 | 365 | protein localization to organelle |
| GO:0043241 | <0.0001 | 0.415 | 41 | 22 | 114 | protein complex disassembly |
| GO:0051674 | <0.0001 | 0.717 | 254 | 207 | 699 | localization of cell |
| GO:0006402 | <0.0001 | 0.429 | 44 | 24 | 121 | mRNA catabolic process |
| GO:0030168 | <0.0001 | 0.521 | 69 | 44 | 190 | platelet activation |
| GO:0031397 | <0.0001 | 0.337 | 29 | 13 | 80 | negative regulation of protein ubiquitination |
| GO:0051607 | <0.0001 | 0.437 | 45 | 25 | 124 | defense response to virus |
| GO:0048584 | <0.0001 | 0.493 | 59 | 36 | 158 | positive regulation of response to stimulus |
| GO:0051325 | <0.0001 | 0.614 | 113 | 82 | 312 | interphase |
| GO:0000084 | <0.0001 | 0.442 | 45 | 25 | 123 | S phase of mitotic cell cycle |
| GO:0071706 | <0.0001 | 0.254 | 20 | 7 | 55 | tumor necrosis factor superfamily cytokine production |
| GO:0060548 | <0.0001 | 0.681 | 175 | 137 | 482 | negative regulation of cell death |
| GO:0044093 | <0.0001 | 0.748 | 303 | 255 | 835 | positive regulation of molecular function |
| GO:0031145 | <0.0001 | 0.324 | 25 | 11 | 70 | anaphase-promoting complex-dependent proteasomal ubiquitin-dependent protein catabolic process |
| GO:0032732 | <0.0001 | <0.0001 | 7 | 0 | 20 | positive regulation of interleukin-1 production |
| GO:0043933 | <0.0001 | 0.541 | 70 | 46 | 191 | macromolecular complex subunit organization |
| GO:0042060 | <0.0001 | 0.688 | 176 | 139 | 485 | wound healing |
| GO:0043066 | <0.0001 | 0.679 | 164 | 128 | 451 | negative regulation of apoptotic process |
| GO:0002576 | <0.0001 | 0.353 | 28 | 13 | 77 | platelet degranulation |
| GO:0006954 | <0.0001 | 0.570 | 80 | 55 | 219 | inflammatory response |
| GO:0071310 | <0.0001 | 0.733 | 251 | 208 | 686 | cellular response to organic substance |
| GO:0033280 | <0.0001 | <0.0001 | 7 | 0 | 19 | response to vitamin D |
| GO:0002460 | <0.0001 | 0.472 | 46 | 27 | 126 | adaptive immune response based on somatic recombination of immune receptors built from immunoglobulin superfamily domains |
| GO:0006959 | <0.0001 | 0.383 | 30 | 15 | 83 | humoral immune response |
| GO:0045621 | <0.0001 | 0.290 | 20 | 8 | 56 | positive regulation of lymphocyte differentiation |
| GO:0051436 | <0.0001 | 0.290 | 20 | 8 | 56 | negative regulation of ubiquitin-protein ligase activity involved in mitotic cell cycle |
| GO:0032269 | <0.0001 | 0.634 | 111 | 82 | 305 | negative regulation of cellular protein metabolic process |
| GO:0032879 | <0.0001 | 0.777 | 367 | 317 | 1010 | regulation of localization |
| GO:0050866 | <0.0001 | 0.397 | 31 | 16 | 86 | negative regulation of cell activation |
| GO:0002218 | <0.0001 | 0.439 | 38 | 21 | 104 | activation of innate immune response |
| GO:0050921 | <0.0001 | 0.354 | 26 | 12 | 71 | positive regulation of chemotaxis |
| GO:0009059 | <0.0001 | 0.838 | 886 | 818 | 2439 | macromolecule biosynthetic process |
| GO:0000278 | <0.0001 | 0.643 | 112 | 84 | 307 | mitotic cell cycle |
| GO:0007093 | <0.0001 | 0.459 | 40 | 23 | 110 | mitotic cell cycle checkpoint |
| GO:0016482 | <0.0001 | 0.675 | 140 | 109 | 383 | cytoplasmic transport |
| GO:0051222 | <0.0001 | 0.500 | 49 | 30 | 134 | positive regulation of protein transport |
| GO:0022411 | <0.0001 | 0.594 | 81 | 57 | 223 | cellular component disassembly |
| GO:0045088 | <0.0001 | 0.551 | 63 | 42 | 174 | regulation of innate immune response |
| GO:0045087 | <0.0001 | 0.510 | 51 | 32 | 138 | innate immune response |
| GO:0044236 | <0.0001 | 0.335 | 23 | 10 | 62 | multicellular organismal metabolic process |
| GO:0016071 | <0.0001 | 0.334 | 22 | 10 | 61 | mRNA metabolic process |
| GO:0002520 | <0.0001 | 0.699 | 161 | 128 | 443 | immune system development |
| GO:0002683 | <0.0001 | 0.399 | 29 | 15 | 80 | negative regulation of immune system process |
| GO:0050870 | <0.0001 | 0.209 | 14 | 4 | 37 | positive regulation of T cell activation |
| GO:0035295 | <0.0001 | 0.615 | 89 | 64 | 243 | tube development |
| GO:0043277 | <0.0001 | <0.0001 | 6 | 0 | 17 | apoptotic cell clearance |
| GO:0050670 | <0.0001 | 0.392 | 28 | 14 | 76 | regulation of lymphocyte proliferation |
| GO:0030324 | <0.0001 | 0.493 | 44 | 27 | 122 | lung development |
| GO:0071216 | <0.0001 | 0.422 | 32 | 17 | 87 | cellular response to biotic stimulus |
| GO:0051437 | 0.001 | 0.341 | 22 | 10 | 61 | positive regulation of ubiquitin-protein ligase activity involved in mitotic cell cycle |
| GO:0072422 | 0.001 | 0.341 | 22 | 10 | 61 | signal transduction involved in DNA damage checkpoint |
| GO:0002757 | 0.001 | 0.403 | 29 | 15 | 79 | immune response-activating signal transduction |
| GO:0019882 | 0.001 | 0.543 | 56 | 37 | 155 | antigen processing and presentation |
| GO:0042330 | 0.001 | 0.716 | 173 | 140 | 476 | taxis |
| GO:0007599 | 0.001 | 0.698 | 149 | 118 | 409 | hemostasis |
| GO:0010862 | 0.001 | 0.083 | 8 | 1 | 22 | positive regulation of pathway-restricted SMAD protein phosphorylation |
| GO:0009615 | 0.001 | 0.375 | 25 | 12 | 67 | response to virus |
| GO:0009617 | 0.001 | 0.559 | 60 | 40 | 163 | response to bacterium |
| GO:0006977 | 0.001 | 0.348 | 22 | 10 | 60 | DNA damage response, signal transduction by p53 class mediator resulting in cell cycle arrest |
| GO:0072474 | 0.001 | 0.348 | 22 | 10 | 60 | signal transduction involved in mitotic cell cycle G1/S checkpoint |
| GO:0008283 | 0.001 | 0.647 | 102 | 77 | 274 | cell proliferation |
| GO:2000045 | 0.001 | 0.422 | 30 | 16 | 82 | regulation of G1/S transition of mitotic cell cycle |
| GO:0009792 | 0.001 | 0.697 | 145 | 115 | 399 | embryo development ending in birth or egg hatching |
| GO:0050704 | 0.001 | <0.0001 | 6 | 0 | 16 | regulation of interleukin-1 secretion |
| GO:0002687 | 0.001 | 0.275 | 16 | 6 | 44 | positive regulation of leukocyte migration |
| GO:0006468 | 0.001 | 0.781 | 311 | 269 | 857 | protein phosphorylation |
| GO:0051438 | 0.001 | 0.414 | 28 | 15 | 78 | regulation of ubiquitin-protein ligase activity |
| GO:0050817 | 0.001 | 0.703 | 148 | 118 | 407 | coagulation |
| GO:0002221 | 0.001 | 0.468 | 36 | 21 | 99 | pattern recognition receptor signaling pathway |
| GO:0031571 | 0.001 | 0.368 | 23 | 11 | 63 | mitotic cell cycle G1/S transition DNA damage checkpoint |
| GO:0045786 | 0.001 | 0.677 | 120 | 93 | 330 | negative regulation of cell cycle |
| GO:0019835 | 0.001 | 0.145 | 9 | 2 | 26 | cytolysis |
| GO:0044265 | 0.001 | 0.723 | 168 | 137 | 463 | cellular macromolecule catabolic process |
| GO:0045580 | 0.001 | 0.387 | 24 | 12 | 66 | regulation of T cell differentiation |
| GO:0034097 | 0.001 | 0.642 | 91 | 68 | 248 | response to cytokine stimulus |
| GO:0002449 | 0.001 | 0.525 | 45 | 29 | 125 | lymphocyte mediated immunity |
| GO:0031349 | 0.001 | 0.519 | 44 | 28 | 121 | positive regulation of defense response |
| GO:0072395 | 0.001 | 0.376 | 23 | 11 | 62 | signal transduction involved in cell cycle checkpoint |
| GO:0010950 | 0.001 | 0.435 | 29 | 16 | 80 | positive regulation of endopeptidase activity |
| GO:0007498 | 0.001 | 0.404 | 25 | 13 | 69 | mesoderm development |
| GO:0051351 | 0.001 | 0.404 | 25 | 13 | 69 | positive regulation of ligase activity |
| GO:0002478 | 0.001 | 0.538 | 48 | 31 | 131 | antigen processing and presentation of exogenous peptide antigen |
| GO:0002263 | 0.001 | 0.494 | 38 | 23 | 104 | cell activation involved in immune response |
| GO:0007568 | 0.001 | 0.488 | 36 | 22 | 100 | aging |
| GO:0042035 | 0.001 | 0.394 | 24 | 12 | 65 | regulation of cytokine biosynthetic process |
| GO:0051271 | 0.001 | 0.503 | 39 | 24 | 107 | negative regulation of cellular component movement |
| GO:0032945 | 0.001 | 0.264 | 14 | 5 | 38 | negative regulation of mononuclear cell proliferation |
| GO:0030198 | 0.002 | 0.541 | 46 | 30 | 126 | extracellular matrix organization |
| GO:0010942 | 0.002 | 0.702 | 126 | 100 | 346 | positive regulation of cell death |
| GO:0019221 | 0.002 | 0.602 | 63 | 45 | 173 | cytokine-mediated signaling pathway |
| GO:0030155 | 0.002 | 0.545 | 46 | 30 | 125 | regulation of cell adhesion |
| GO:0008285 | 0.002 | 0.728 | 153 | 125 | 421 | negative regulation of cell proliferation |
| GO:0030097 | 0.002 | 0.717 | 139 | 112 | 381 | hemopoiesis |
| GO:0001817 | 0.002 | 0.442 | 27 | 15 | 73 | regulation of cytokine production |
| GO:0002694 | 0.002 | 0.442 | 27 | 15 | 73 | regulation of leukocyte activation |
| GO:0050869 | 0.002 | 0.097 | 7 | 1 | 19 | negative regulation of B cell activation |
| GO:0070727 | 0.002 | 0.780 | 241 | 207 | 663 | cellular macromolecule localization |
| GO:0055123 | 0.002 | 0.475 | 31 | 18 | 84 | digestive system development |
| GO:0010941 | 0.002 | 0.623 | 69 | 50 | 185 | regulation of cell death |
| GO:0044260 | 0.002 | 0.870 | 1208 | 1149 | 3286 | cellular macromolecule metabolic process |
| GO:0042130 | 0.002 | 0.209 | 10 | 3 | 28 | negative regulation of T cell proliferation |
| GO:0050679 | 0.002 | 0.500 | 34 | 21 | 94 | positive regulation of epithelial cell proliferation |
| GO:0000082 | 0.003 | 0.494 | 33 | 20 | 90 | G1/S transition of mitotic cell cycle |
| GO:0002688 | 0.003 | 0.307 | 15 | 6 | 40 | regulation of leukocyte chemotaxis |
| GO:0006364 | 0.003 | 0.387 | 20 | 10 | 55 | rRNA processing |
| GO:0040013 | 0.003 | 0.547 | 43 | 28 | 117 | negative regulation of locomotion |
| GO:0015949 | 0.003 | <0.0001 | 5 | 0 | 13 | nucleobase-containing small molecule interconversion |
| GO:0042107 | 0.003 | 0.464 | 28 | 16 | 76 | cytokine metabolic process |
| GO:0032722 | 0.003 | 0.166 | 8 | 2 | 23 | positive regulation of chemokine production |
| GO:0032760 | 0.003 | 0.166 | 8 | 2 | 23 | positive regulation of tumor necrosis factor production |
| GO:0050708 | 0.003 | 0.508 | 34 | 21 | 93 | regulation of protein secretion |
| GO:0050795 | 0.003 | 0.546 | 41 | 27 | 113 | regulation of behavior |
| GO:0040017 | 0.003 | 0.580 | 49 | 34 | 135 | positive regulation of locomotion |
| GO:0001501 | 0.003 | 0.692 | 102 | 80 | 280 | skeletal system development |
| GO:0001763 | 0.003 | 0.604 | 56 | 40 | 155 | morphogenesis of a branching structure |
| GO:0032649 | 0.003 | 0.102 | 7 | 1 | 18 | regulation of interferon-gamma production |
| GO:0001655 | 0.003 | 0.658 | 79 | 60 | 218 | urogenital system development |
| GO:0045682 | 0.003 | 0.218 | 10 | 3 | 27 | regulation of epidermis development |
| GO:0046638 | 0.003 | 0.218 | 10 | 3 | 27 | positive regulation of alpha-beta T cell differentiation |
| GO:0071277 | 0.003 | 0.218 | 10 | 3 | 27 | cellular response to calcium ion |
| GO:0048519 | 0.003 | 0.860 | 711 | 661 | 1929 | negative regulation of biological process |
| GO:0090304 | 0.003 | 0.871 | 904 | 850 | 2488 | nucleic acid metabolic process |
| GO:0031295 | 0.003 | 0.396 | 20 | 10 | 54 | T cell costimulation |
| GO:0042633 | 0.003 | 0.396 | 20 | 10 | 54 | hair cycle |
| GO:0043588 | 0.004 | 0.317 | 14 | 6 | 39 | skin development |
| GO:0009967 | 0.004 | 0.772 | 199 | 170 | 547 | positive regulation of signal transduction |
| GO:0007159 | 0.004 | 0.291 | 13 | 5 | 35 | leukocyte cell-cell adhesion |
| GO:0007243 | 0.004 | 0.592 | 51 | 36 | 140 | intracellular protein kinase cascade |
| GO:0001892 | 0.004 | 0.427 | 22 | 12 | 61 | embryonic placenta development |
| GO:0008284 | 0.004 | 0.742 | 149 | 123 | 407 | positive regulation of cell proliferation |
| GO:0051240 | 0.004 | 0.741 | 146 | 121 | 403 | positive regulation of multicellular organismal process |
| GO:0042981 | 0.004 | 0.617 | 59 | 43 | 160 | regulation of apoptotic process |
| GO:0046634 | 0.004 | 0.383 | 18 | 9 | 50 | regulation of alpha-beta T cell activation |
| GO:0071356 | 0.004 | 0.383 | 18 | 9 | 50 | cellular response to tumor necrosis factor |
| GO:0090092 | 0.004 | 0.560 | 42 | 28 | 115 | regulation of transmembrane receptor protein serine/threonine kinase signaling pathway |
| GO:0060429 | 0.004 | 0.637 | 66 | 49 | 181 | epithelium development |
| GO:0042742 | 0.004 | 0.416 | 21 | 11 | 57 | defense response to bacterium |
| GO:0031570 | 0.004 | 0.544 | 38 | 25 | 105 | DNA integrity checkpoint |
| GO:0014031 | 0.004 | 0.524 | 35 | 22 | 95 | mesenchymal cell development |
| GO:0000236 | 0.004 | 0.367 | 17 | 8 | 46 | mitotic prometaphase |
| GO:0050729 | 0.004 | 0.348 | 15 | 7 | 42 | positive regulation of inflammatory response |
| GO:0045616 | 0.004 | <0.0001 | 4 | 0 | 12 | regulation of keratinocyte differentiation |
| GO:0050718 | 0.004 | <0.0001 | 4 | 0 | 12 | positive regulation of interleukin-1 beta secretion |
| GO:2000404 | 0.004 | <0.0001 | 4 | 0 | 12 | regulation of T cell migration |
| GO:0034329 | 0.004 | 0.596 | 50 | 35 | 137 | cell junction assembly |
| GO:0001942 | 0.005 | 0.405 | 19 | 10 | 53 | hair follicle development |
| GO:0022404 | 0.005 | 0.405 | 19 | 10 | 53 | molting cycle process |
| GO:0051345 | 0.005 | 0.728 | 124 | 101 | 341 | positive regulation of hydrolase activity |
| GO:0045321 | 0.005 | 0.636 | 64 | 47 | 172 | leukocyte activation |
| GO:0071779 | 0.005 | 0.481 | 27 | 16 | 74 | G1/S transition checkpoint |
| GO:0006418 | 0.005 | 0.228 | 9 | 3 | 26 | tRNA aminoacylation for protein translation |
| GO:0071347 | 0.005 | 0.301 | 12 | 5 | 34 | cellular response to interleukin-1 |
| GO:0006739 | 0.005 | 0.109 | 6 | 1 | 17 | NADP metabolic process |
| GO:0051298 | 0.005 | 0.109 | 6 | 1 | 17 | centrosome duplication |
| GO:0055093 | 0.005 | 0.109 | 6 | 1 | 17 | response to hyperoxia |
| GO:2000516 | 0.005 | 0.109 | 6 | 1 | 17 | positive regulation of CD4-positive, alpha-beta T cell activation |
| GO:0071260 | 0.005 | 0.392 | 18 | 9 | 49 | cellular response to mechanical stimulus |
| GO:0001525 | 0.005 | 0.654 | 69 | 52 | 189 | angiogenesis |
| GO:0007569 | 0.005 | 0.426 | 20 | 11 | 56 | cell aging |
| GO:0030282 | 0.005 | 0.453 | 23 | 13 | 63 | bone mineralization |
| GO:0031398 | 0.006 | 0.550 | 36 | 24 | 100 | positive regulation of protein ubiquitination |
| GO:0051301 | 0.006 | 0.686 | 85 | 66 | 232 | cell division |
| GO:0044092 | 0.006 | 0.774 | 179 | 153 | 494 | negative regulation of molecular function |
| GO:0032268 | 0.006 | 0.796 | 227 | 198 | 619 | regulation of cellular protein metabolic process |
| GO:0050715 | 0.006 | 0.359 | 15 | 7 | 41 | positive regulation of cytokine secretion |
| GO:0008063 | 0.006 | 0.469 | 24 | 14 | 66 | Toll signaling pathway |
| GO:0033157 | 0.006 | 0.603 | 48 | 34 | 132 | regulation of intracellular protein transport |
| GO:0043516 | 0.006 | 0.184 | 8 | 2 | 21 | regulation of DNA damage response, signal transduction by p53 class mediator |
| GO:0044409 | 0.006 | 0.184 | 8 | 2 | 21 | entry into host |
| GO:0050853 | 0.006 | 0.184 | 8 | 2 | 21 | B cell receptor signaling pathway |
| GO:0051806 | 0.006 | 0.184 | 8 | 2 | 21 | entry into cell of other organism involved in symbiotic interaction |
| GO:0052192 | 0.006 | 0.184 | 8 | 2 | 21 | movement in environment of other organism involved in symbiotic interaction |
| GO:0045444 | 0.006 | 0.528 | 31 | 20 | 86 | fat cell differentiation |
| GO:0006968 | 0.006 | 0.403 | 17 | 9 | 48 | cellular defense response |
| GO:0007179 | 0.006 | 0.614 | 50 | 36 | 138 | transforming growth factor beta receptor signaling pathway |
| GO:0030593 | 0.007 | 0.311 | 12 | 5 | 33 | neutrophil chemotaxis |
| GO:0006919 | 0.007 | 0.462 | 23 | 13 | 62 | activation of cysteine-type endopeptidase activity involved in apoptotic process |
| GO:0071478 | 0.007 | 0.462 | 23 | 13 | 62 | cellular response to radiation |
| GO:0045071 | 0.007 | 0.238 | 9 | 3 | 25 | negative regulation of viral genome replication |
| GO:0002287 | 0.007 | 0.279 | 11 | 4 | 29 | alpha-beta T cell activation involved in immune response |
| GO:0002292 | 0.007 | 0.279 | 11 | 4 | 29 | T cell differentiation involved in immune response |
| GO:0006925 | 0.007 | <0.0001 | 4 | 0 | 11 | inflammatory cell apoptotic process |
| GO:0030214 | 0.007 | <0.0001 | 4 | 0 | 11 | hyaluronan catabolic process |
| GO:0031581 | 0.007 | <0.0001 | 4 | 0 | 11 | hemidesmosome assembly |
| GO:0071363 | 0.007 | 0.746 | 128 | 106 | 352 | cellular response to growth factor stimulus |
| GO:0034142 | 0.007 | 0.498 | 26 | 16 | 72 | toll-like receptor 4 signaling pathway |
| GO:0010718 | 0.007 | 0.117 | 6 | 1 | 16 | positive regulation of epithelial to mesenchymal transition |
| GO:0050856 | 0.007 | 0.117 | 6 | 1 | 16 | regulation of T cell receptor signaling pathway |
| GO:0050891 | 0.007 | 0.117 | 6 | 1 | 16 | multicellular organismal water homeostasis |
| GO:0048015 | 0.007 | 0.593 | 43 | 30 | 118 | phosphatidylinositol-mediated signaling |
| GO:0031401 | 0.008 | 0.776 | 167 | 143 | 459 | positive regulation of protein modification process |
| GO:0032946 | 0.008 | 0.511 | 27 | 17 | 75 | positive regulation of mononuclear cell proliferation |
| GO:0050658 | 0.008 | 0.536 | 31 | 20 | 85 | RNA transport |
| GO:0030335 | 0.008 | 0.621 | 50 | 36 | 136 | positive regulation of cell migration |
| GO:0010498 | 0.008 | 0.652 | 60 | 45 | 165 | proteasomal protein catabolic process |
| GO:0051250 | 0.008 | 0.412 | 17 | 9 | 47 | negative regulation of lymphocyte activation |
| GO:0018279 | 0.008 | 0.472 | 22 | 13 | 61 | protein N-linked glycosylation via asparagine |
| GO:0034138 | 0.008 | 0.472 | 22 | 13 | 61 | toll-like receptor 3 signaling pathway |
| GO:0009611 | 0.009 | 0.470 | 22 | 13 | 59 | response to wounding |
| GO:0042127 | 0.009 | 0.522 | 28 | 18 | 76 | regulation of cell proliferation |
| GO:0048546 | 0.009 | 0.194 | 7 | 2 | 20 | digestive tract morphogenesis |
| GO:0034446 | 0.009 | 0.194 | 7 | 2 | 20 | substrate adhesion-dependent cell spreading |
| GO:0002828 | 0.009 | 0.194 | 7 | 2 | 20 | regulation of type 2 immune response |
| GO:0044085 | 0.009 | 0.847 | 393 | 358 | 1082 | cellular component biogenesis |
| GO:0050793 | 0.009 | 0.736 | 111 | 91 | 302 | regulation of developmental process |
| GO:0007015 | 0.009 | 0.654 | 58 | 44 | 161 | actin filament organization |
| GO:0071559 | 0.009 | 0.654 | 58 | 44 | 161 | response to transforming growth factor beta stimulus |
| GO:0050777 | 0.009 | 0.399 | 16 | 8 | 43 | negative regulation of immune response |
| GO:1901568 | 0.009 | 0.399 | 16 | 8 | 43 | fatty acid derivative metabolic process |
| GO:0002294 | 0.009 | 0.291 | 10 | 4 | 28 | CD4-positive, alpha-beta T cell differentiation involved in immune response |
| GO:0034644 | 0.009 | 0.291 | 10 | 4 | 28 | cellular response to UV |
| GO:0043038 | 0.009 | 0.291 | 10 | 4 | 28 | amino acid activation |
| GO:0032755 | 0.009 | 0.250 | 9 | 3 | 24 | positive regulation of interleukin-6 production |
| GO:0007229 | 0.010 | 0.465 | 21 | 12 | 57 | integrin-mediated signaling pathway |
| GO:0060759 | 0.010 | 0.520 | 27 | 17 | 74 | regulation of response to cytokine stimulus |
| GO:0006917 | 0.010 | 0.640 | 53 | 39 | 145 | induction of apoptosis |
| GO:0036211 | 0.010 | 0.871 | 595 | 554 | 1637 | protein modification process |
| GO:0008380 | 0.010 | 0.695 | 76 | 60 | 210 | RNA splicing |
| GO:0015931 | 0.010 | 0.580 | 36 | 25 | 100 | nucleobase-containing compound transport |
| GO:0000087 | 0.010 | 0.705 | 82 | 65 | 225 | M phase of mitotic cell cycle |
| GO:0050852 | 0.010 | 0.481 | 22 | 13 | 60 | T cell receptor signaling pathway |
| GO:0042327 | 0.011 | 0.784 | 164 | 141 | 452 | positive regulation of phosphorylation |
| GO:0010605 | 0.011 | 0.842 | 343 | 311 | 945 | negative regulation of macromolecule metabolic process |
| GO:1900180 | 0.011 | 0.588 | 37 | 26 | 103 | regulation of protein localization to nucleus |
| GO:0006403 | 0.011 | 0.564 | 33 | 22 | 90 | RNA localization |
| GO:0051897 | 0.011 | 0.457 | 19 | 11 | 53 | positive regulation of protein kinase B signaling cascade |
| GO:0003179 | 0.011 | 0.125 | 5 | 1 | 15 | heart valve morphogenesis |
| GO:0035455 | 0.011 | 0.125 | 5 | 1 | 15 | response to interferon-alpha |
| GO:0060272 | 0.011 | <0.0001 | 4 | 0 | 10 | embryonic skeletal joint morphogenesis |
| GO:0051092 | 0.011 | 0.543 | 29 | 19 | 80 | positive regulation of NF-kappaB transcription factor activity |
| GO:0010627 | 0.011 | 0.798 | 187 | 163 | 516 | regulation of intracellular protein kinase cascade |
| GO:0006369 | 0.011 | 0.361 | 13 | 6 | 35 | termination of RNA polymerase II transcription |
| GO:0042770 | 0.011 | 0.408 | 15 | 8 | 42 | signal transduction in response to DNA damage |
| GO:0016064 | 0.012 | 0.529 | 27 | 17 | 73 | immunoglobulin mediated immune response |
| GO:0007565 | 0.012 | 0.634 | 48 | 35 | 131 | female pregnancy |
| GO:0043620 | 0.012 | 0.336 | 11 | 5 | 31 | regulation of DNA-dependent transcription in response to stress |
| GO:0006260 | 0.012 | 0.687 | 68 | 53 | 187 | DNA replication |
| GO:0001701 | 0.012 | 0.686 | 67 | 52 | 183 | in utero embryonic development |
| GO:0002285 | 0.013 | 0.513 | 24 | 15 | 66 | lymphocyte activation involved in immune response |
| GO:0046579 | 0.013 | 0.206 | 7 | 2 | 19 | positive regulation of Ras protein signal transduction |
| GO:0048199 | 0.013 | 0.206 | 7 | 2 | 19 | vesicle targeting, to, from or within Golgi |
| GO:0050663 | 0.013 | 0.261 | 8 | 3 | 23 | cytokine secretion |
| GO:0006901 | 0.013 | 0.304 | 10 | 4 | 27 | vesicle coating |
| GO:0006958 | 0.013 | 0.304 | 10 | 4 | 27 | complement activation, classical pathway |
| GO:0015914 | 0.013 | 0.304 | 10 | 4 | 27 | phospholipid transport |
| GO:0032675 | 0.013 | 0.261 | 8 | 3 | 23 | regulation of interleukin-6 production |
| GO:0006606 | 0.013 | 0.675 | 61 | 47 | 168 | protein import into nucleus |
| GO:0030855 | 0.013 | 0.711 | 79 | 63 | 217 | epithelial cell differentiation |
| GO:0035666 | 0.013 | 0.493 | 21 | 13 | 59 | TRIF-dependent toll-like receptor signaling pathway |
| GO:0061180 | 0.013 | 0.493 | 21 | 13 | 59 | mammary gland epithelium development |
| GO:0045727 | 0.013 | 0.394 | 14 | 7 | 38 | positive regulation of translation |
| GO:0009887 | 0.014 | 0.807 | 194 | 170 | 533 | organ morphogenesis |
| GO:0008064 | 0.014 | 0.526 | 25 | 16 | 69 | regulation of actin polymerization or depolymerization |
| GO:0071222 | 0.014 | 0.435 | 16 | 9 | 45 | cellular response to lipopolysaccharide |
| GO:0071704 | 0.014 | 0.863 | 598 | 564 | 1588 | organic substance metabolic process |
| GO:0000377 | 0.014 | 0.660 | 54 | 41 | 149 | RNA splicing, via transesterification reactions with bulged adenosine as nucleophile |
| GO:0001822 | 0.014 | 0.660 | 54 | 41 | 149 | kidney development |
| GO:0060443 | 0.014 | 0.436 | 16 | 9 | 45 | mammary gland morphogenesis |
| GO:0044249 | 0.014 | 0.896 | 1008 | 963 | 2762 | cellular biosynthetic process |
| GO:0001818 | 0.014 | 0.589 | 35 | 24 | 95 | negative regulation of cytokine production |
| GO:0090068 | 0.015 | 0.650 | 49 | 37 | 136 | positive regulation of cell cycle process |
| GO:0000819 | 0.015 | 0.374 | 12 | 6 | 34 | sister chromatid segregation |
| GO:0006636 | 0.015 | 0.374 | 12 | 6 | 34 | unsaturated fatty acid biosynthetic process |
| GO:0043299 | 0.015 | 0.374 | 12 | 6 | 34 | leukocyte degranulation |
| GO:0050764 | 0.015 | 0.374 | 12 | 6 | 34 | regulation of phagocytosis |
| GO:1901607 | 0.015 | 0.374 | 12 | 6 | 34 | alpha-amino acid biosynthetic process |
| GO:0000280 | 0.015 | 0.717 | 80 | 64 | 219 | nuclear division |
| GO:0030336 | 0.015 | 0.580 | 32 | 22 | 88 | negative regulation of cell migration |
| GO:0002253 | 0.015 | 0.400 | 14 | 7 | 37 | activation of immune response |
| GO:0046824 | 0.015 | 0.458 | 17 | 10 | 48 | positive regulation of nucleocytoplasmic transport |
| GO:0046483 | 0.016 | 0.901 | 1134 | 1089 | 3122 | heterocycle metabolic process |
| GO:0032677 | 0.016 | 0.134 | 5 | 1 | 14 | regulation of interleukin-8 production |
| GO:0032728 | 0.016 | 0.135 | 5 | 1 | 14 | positive regulation of interferon-beta production |
| GO:0042274 | 0.016 | 0.135 | 5 | 1 | 14 | ribosomal small subunit biogenesis |
| GO:0043330 | 0.016 | 0.135 | 5 | 1 | 14 | response to exogenous dsRNA |
| GO:0045577 | 0.016 | 0.135 | 5 | 1 | 14 | regulation of B cell differentiation |
| GO:0060216 | 0.016 | 0.135 | 5 | 1 | 14 | definitive hemopoiesis |
| GO:0060716 | 0.016 | 0.135 | 5 | 1 | 14 | labyrinthine layer blood vessel development |
| GO:0097028 | 0.016 | 0.135 | 5 | 1 | 14 | dendritic cell differentiation |
| GO:2000403 | 0.016 | 0.135 | 5 | 1 | 14 | positive regulation of lymphocyte migration |
| GO:0006271 | 0.016 | 0.349 | 11 | 5 | 30 | DNA strand elongation involved in DNA replication |
| GO:0000075 | 0.016 | 0.404 | 14 | 7 | 37 | cell cycle checkpoint |
| GO:0007162 | 0.017 | 0.537 | 25 | 16 | 68 | negative regulation of cell adhesion |
| GO:0034641 | 0.017 | 0.901 | 1146 | 1101 | 3142 | cellular nitrogen compound metabolic process |
| GO:0001935 | 0.017 | <0.0001 | 3 | 0 | 9 | endothelial cell proliferation |
| GO:0002467 | 0.017 | <0.0001 | 3 | 0 | 9 | germinal center formation |
| GO:0002643 | 0.017 | <0.0001 | 3 | 0 | 9 | regulation of tolerance induction |
| GO:0007076 | 0.017 | <0.0001 | 3 | 0 | 9 | mitotic chromosome condensation |
| GO:0042535 | 0.017 | <0.0001 | 3 | 0 | 9 | positive regulation of tumor necrosis factor biosynthetic process |
| GO:0046653 | 0.017 | <0.0001 | 3 | 0 | 9 | tetrahydrofolate metabolic process |
| GO:0048535 | 0.017 | <0.0001 | 3 | 0 | 9 | lymph node development |
| GO:0050857 | 0.017 | <0.0001 | 3 | 0 | 9 | positive regulation of antigen receptor-mediated signaling pathway |
| GO:0090026 | 0.017 | <0.0001 | 3 | 0 | 9 | positive regulation of monocyte chemotaxis |
| GO:0090505 | 0.017 | <0.0001 | 3 | 0 | 9 | epiboly involved in wound healing |
| GO:0006396 | 0.017 | 0.620 | 39 | 28 | 106 | RNA processing |
| GO:0060021 | 0.017 | 0.480 | 19 | 11 | 51 | palate development |
| GO:0022409 | 0.017 | 0.318 | 9 | 4 | 26 | positive regulation of cell-cell adhesion |
| GO:0032088 | 0.017 | 0.407 | 13 | 7 | 37 | negative regulation of NF-kappaB transcription factor activity |
| GO:0051650 | 0.017 | 0.407 | 13 | 7 | 37 | establishment of vesicle localization |
| GO:0002532 | 0.018 | 0.219 | 7 | 2 | 18 | production of molecular mediator involved in inflammatory response |
| GO:0030219 | 0.018 | 0.219 | 7 | 2 | 18 | megakaryocyte differentiation |
| GO:0045648 | 0.018 | 0.219 | 7 | 2 | 18 | positive regulation of erythrocyte differentiation |
| GO:0061437 | 0.018 | 0.219 | 7 | 2 | 18 | renal system vasculature development |
| GO:0030278 | 0.018 | 0.641 | 43 | 32 | 119 | regulation of ossification |
| GO:0031103 | 0.018 | 0.276 | 8 | 3 | 22 | axon regeneration |
| GO:0034121 | 0.018 | 0.276 | 8 | 3 | 22 | regulation of toll-like receptor signaling pathway |
| GO:0045058 | 0.018 | 0.276 | 8 | 3 | 22 | T cell selection |
| GO:0002697 | 0.018 | 0.679 | 56 | 43 | 153 | regulation of immune effector process |
| GO:0042102 | 0.019 | 0.499 | 20 | 12 | 54 | positive regulation of T cell proliferation |
| GO:0060349 | 0.019 | 0.499 | 20 | 12 | 54 | bone morphogenesis |
| GO:0002755 | 0.019 | 0.534 | 23 | 15 | 64 | MyD88-dependent toll-like receptor signaling pathway |
| GO:0043281 | 0.019 | 0.512 | 21 | 13 | 57 | regulation of cysteine-type endopeptidase activity involved in apoptotic process |
| GO:0007431 | 0.019 | 0.388 | 12 | 6 | 33 | salivary gland development |
| GO:0046677 | 0.019 | 0.388 | 12 | 6 | 33 | response to antibiotic |
| GO:0032479 | 0.020 | 0.472 | 17 | 10 | 47 | regulation of type I interferon production |
| GO:0048568 | 0.020 | 0.699 | 62 | 49 | 170 | embryonic organ development |
| GO:0001657 | 0.021 | 0.581 | 29 | 20 | 80 | ureteric bud development |
| GO:0030098 | 0.021 | 0.662 | 47 | 36 | 130 | lymphocyte differentiation |
| GO:0034655 | 0.021 | 0.819 | 190 | 168 | 523 | nucleobase-containing compound catabolic process |
| GO:0051168 | 0.021 | 0.490 | 18 | 11 | 50 | nuclear export |
| GO:0022612 | 0.021 | 0.490 | 18 | 11 | 50 | gland morphogenesis |
| GO:0032680 | 0.021 | 0.363 | 11 | 5 | 29 | regulation of tumor necrosis factor production |
| GO:0032526 | 0.022 | 0.559 | 25 | 17 | 70 | response to retinoic acid |
| GO:1901360 | 0.022 | 0.907 | 1191 | 1148 | 3278 | organic cyclic compound metabolic process |
| GO:0008104 | 0.022 | 0.865 | 372 | 343 | 1025 | protein localization |
| GO:0034134 | 0.022 | 0.531 | 22 | 14 | 60 | toll-like receptor 2 signaling pathway |
| GO:0052548 | 0.022 | 0.646 | 42 | 31 | 114 | regulation of endopeptidase activity |
| GO:0006406 | 0.023 | 0.462 | 16 | 9 | 43 | mRNA export from nucleus |
| GO:0014068 | 0.023 | 0.462 | 16 | 9 | 43 | positive regulation of phosphatidylinositol 3-kinase cascade |
| GO:0006940 | 0.023 | 0.422 | 13 | 7 | 36 | regulation of smooth muscle contraction |
| GO:0034109 | 0.023 | 0.422 | 13 | 7 | 36 | homotypic cell-cell adhesion |
| GO:0006725 | 0.023 | 0.908 | 1139 | 1097 | 3135 | cellular aromatic compound metabolic process |
| GO:0014910 | 0.023 | 0.333 | 9 | 4 | 25 | regulation of smooth muscle cell migration |
| GO:0032570 | 0.023 | 0.333 | 9 | 4 | 25 | response to progesterone stimulus |
| GO:0032729 | 0.023 | 0.333 | 9 | 4 | 25 | positive regulation of interferon-gamma production |
| GO:0006521 | 0.024 | 0.511 | 19 | 12 | 53 | regulation of cellular amino acid metabolic process |
| GO:0002320 | 0.024 | 0.146 | 5 | 1 | 13 | lymphoid progenitor cell differentiation |
| GO:0032757 | 0.024 | 0.146 | 5 | 1 | 13 | positive regulation of interleukin-8 production |
| GO:0032878 | 0.024 | 0.146 | 5 | 1 | 13 | regulation of establishment or maintenance of cell polarity |
| GO:0042255 | 0.024 | 0.146 | 5 | 1 | 13 | ribosome assembly |
| GO:0042659 | 0.024 | 0.146 | 5 | 1 | 13 | regulation of cell fate specification |
| GO:0060317 | 0.024 | 0.146 | 5 | 1 | 13 | cardiac epithelial to mesenchymal transition |
| GO:0071295 | 0.024 | 0.146 | 5 | 1 | 13 | cellular response to vitamin |
| GO:0090023 | 0.024 | 0.146 | 5 | 1 | 13 | positive regulation of neutrophil chemotaxis |
| GO:1901623 | 0.024 | 0.146 | 5 | 1 | 13 | regulation of lymphocyte chemotaxis |
| GO:0006397 | 0.024 | 0.757 | 93 | 78 | 257 | mRNA processing |
| GO:0045859 | 0.024 | 0.813 | 166 | 146 | 457 | regulation of protein kinase activity |
| GO:0036294 | 0.025 | 0.558 | 24 | 16 | 66 | cellular response to decreased oxygen levels |
| GO:0045123 | 0.025 | 0.291 | 8 | 3 | 21 | cellular extravasation |
| GO:0060445 | 0.025 | 0.291 | 8 | 3 | 21 | branching involved in salivary gland morphogenesis |
| GO:2000377 | 0.025 | 0.485 | 17 | 10 | 46 | regulation of reactive oxygen species metabolic process |
| GO:0002407 | 0.025 | 0.233 | 6 | 2 | 17 | dendritic cell chemotaxis |
| GO:0044060 | 0.025 | 0.233 | 6 | 2 | 17 | regulation of endocrine process |
| GO:0046718 | 0.025 | 0.233 | 6 | 2 | 17 | viral entry into host cell |
| GO:0072012 | 0.025 | 0.233 | 6 | 2 | 17 | glomerulus vasculature development |
| GO:0046456 | 0.025 | 0.403 | 12 | 6 | 32 | icosanoid biosynthetic process |
| GO:0002275 | 0.026 | 0.451 | 14 | 8 | 39 | myeloid cell activation involved in immune response |
| GO:0033692 | 0.026 | 0.451 | 14 | 8 | 39 | cellular polysaccharide biosynthetic process |
| GO:0070371 | 0.026 | 0.636 | 37 | 27 | 101 | ERK1 and ERK2 cascade |
| GO:0000910 | 0.026 | 0.570 | 25 | 17 | 69 | cytokinesis |
| GO:0045766 | 0.026 | 0.570 | 25 | 17 | 69 | positive regulation of angiogenesis |
| GO:0048762 | 0.026 | <0.0001 | 3 | 0 | 8 | mesenchymal cell differentiation |
| GO:0002577 | 0.027 | <0.0001 | 3 | 0 | 8 | regulation of antigen processing and presentation |
| GO:0006047 | 0.027 | <0.0001 | 3 | 0 | 8 | UDP-N-acetylglucosamine metabolic process |
| GO:0007350 | 0.027 | <0.0001 | 3 | 0 | 8 | blastoderm segmentation |
| GO:0008595 | 0.027 | <0.0001 | 3 | 0 | 8 | anterior/posterior axis specification, embryo |
| GO:0010820 | 0.027 | <0.0001 | 3 | 0 | 8 | positive regulation of T cell chemotaxis |
| GO:0015669 | 0.027 | <0.0001 | 3 | 0 | 8 | gas transport |
| GO:0030157 | 0.027 | <0.0001 | 3 | 0 | 8 | pancreatic juice secretion |
| GO:0032928 | 0.027 | <0.0001 | 3 | 0 | 8 | regulation of superoxide anion generation |
| GO:0035357 | 0.027 | <0.0001 | 3 | 0 | 8 | peroxisome proliferator activated receptor signaling pathway |
| GO:0042362 | 0.027 | <0.0001 | 3 | 0 | 8 | fat-soluble vitamin biosynthetic process |
| GO:0043129 | 0.027 | <0.0001 | 3 | 0 | 8 | surfactant homeostasis |
| GO:0045059 | 0.027 | <0.0001 | 3 | 0 | 8 | positive thymic T cell selection |
| GO:0060391 | 0.027 | <0.0001 | 3 | 0 | 8 | positive regulation of SMAD protein import into nucleus |
| GO:0060513 | 0.027 | <0.0001 | 3 | 0 | 8 | prostatic bud formation |
| GO:0090177 | 0.027 | <0.0001 | 3 | 0 | 8 | establishment of planar polarity involved in neural tube closure |
| GO:2000352 | 0.027 | <0.0001 | 3 | 0 | 8 | negative regulation of endothelial cell apoptotic process |
| GO:0090257 | 0.027 | 0.648 | 39 | 29 | 107 | regulation of muscle system process |
| GO:0042990 | 0.027 | 0.505 | 18 | 11 | 49 | regulation of transcription factor import into nucleus |
| GO:0071887 | 0.027 | 0.505 | 18 | 11 | 49 | leukocyte apoptotic process |
| GO:0032963 | 0.028 | 0.379 | 10 | 5 | 28 | collagen metabolic process |
| GO:0060562 | 0.028 | 0.605 | 30 | 21 | 81 | epithelial tube morphogenesis |
| GO:0051208 | 0.028 | 0.380 | 10 | 5 | 28 | sequestering of calcium ion |
| GO:0001936 | 0.029 | 0.557 | 23 | 15 | 62 | regulation of endothelial cell proliferation |
| GO:0006399 | 0.029 | 0.524 | 19 | 12 | 52 | tRNA metabolic process |
| GO:0033135 | 0.029 | 0.524 | 19 | 12 | 52 | regulation of peptidyl-serine phosphorylation |
| GO:0050878 | 0.029 | 0.558 | 22 | 15 | 61 | regulation of body fluid levels |
| GO:0003206 | 0.030 | 0.602 | 28 | 20 | 78 | cardiac chamber morphogenesis |
| GO:0007155 | 0.030 | 0.823 | 170 | 151 | 464 | cell adhesion |
| GO:0051128 | 0.031 | 0.867 | 324 | 298 | 891 | regulation of cellular component organization |
| GO:0010562 | 0.031 | 0.831 | 186 | 166 | 512 | positive regulation of phosphorus metabolic process |
| GO:0009913 | 0.031 | 0.581 | 25 | 17 | 68 | epidermal cell differentiation |
| GO:0006888 | 0.031 | 0.349 | 9 | 4 | 24 | ER to Golgi vesicle-mediated transport |
| GO:0043901 | 0.031 | 0.499 | 16 | 10 | 45 | negative regulation of multi-organism process |
| GO:0060491 | 0.031 | 0.499 | 16 | 10 | 45 | regulation of cell projection assembly |
| GO:0000722 | 0.031 | 0.350 | 9 | 4 | 24 | telomere maintenance via recombination |
| GO:0001894 | 0.032 | 0.619 | 31 | 22 | 84 | tissue homeostasis |
| GO:0001649 | 0.032 | 0.657 | 39 | 29 | 106 | osteoblast differentiation |
| GO:0050863 | 0.032 | 0.304 | 7 | 3 | 20 | regulation of T cell activation |
| GO:0043687 | 0.032 | 0.662 | 40 | 30 | 109 | post-translational protein modification |
| GO:0045995 | 0.032 | 0.465 | 14 | 8 | 38 | regulation of embryonic development |
| GO:0018193 | 0.033 | 0.825 | 169 | 150 | 465 | peptidyl-amino acid modification |
| GO:0031100 | 0.033 | 0.466 | 14 | 8 | 38 | organ regeneration |
| GO:0061035 | 0.033 | 0.466 | 14 | 8 | 38 | regulation of cartilage development |
| GO:0006281 | 0.033 | 0.768 | 90 | 76 | 248 | DNA repair |
| GO:0032069 | 0.033 | 0.555 | 21 | 14 | 58 | regulation of nuclease activity |
| GO:0006412 | 0.033 | 0.649 | 36 | 27 | 98 | translation |
| GO:0006801 | 0.033 | 0.419 | 11 | 6 | 31 | superoxide metabolic process |
| GO:0042116 | 0.033 | 0.419 | 11 | 6 | 31 | macrophage activation |
| GO:0042110 | 0.034 | 0.645 | 35 | 26 | 95 | T cell activation |
| GO:0010564 | 0.034 | 0.723 | 61 | 49 | 166 | regulation of cell cycle process |
| GO:0016053 | 0.034 | 0.738 | 68 | 56 | 188 | organic acid biosynthetic process |
| GO:0071840 | 0.034 | 0.896 | 602 | 571 | 1635 | cellular component organization or biogenesis |
| GO:0030041 | 0.034 | 0.603 | 27 | 19 | 74 | actin filament polymerization |
| GO:0043280 | 0.034 | 0.158 | 4 | 1 | 12 | positive regulation of cysteine-type endopeptidase activity involved in apoptotic process |
| GO:0000387 | 0.034 | 0.309 | 7 | 3 | 20 | spliceosomal snRNP assembly |
| GO:0006693 | 0.034 | 0.309 | 7 | 3 | 20 | prostaglandin metabolic process |
| GO:0030514 | 0.034 | 0.309 | 7 | 3 | 20 | negative regulation of BMP signaling pathway |
| GO:0034394 | 0.034 | 0.309 | 7 | 3 | 20 | protein localization to cell surface |
| GO:0046633 | 0.034 | 0.309 | 7 | 3 | 20 | alpha-beta T cell proliferation |
| GO:0060571 | 0.034 | 0.309 | 7 | 3 | 20 | morphogenesis of an epithelial fold |
| GO:0002686 | 0.035 | 0.159 | 4 | 1 | 12 | negative regulation of leukocyte migration |
| GO:0003203 | 0.035 | 0.159 | 4 | 1 | 12 | endocardial cushion morphogenesis |
| GO:0006957 | 0.035 | 0.159 | 4 | 1 | 12 | complement activation, alternative pathway |
| GO:0010507 | 0.035 | 0.159 | 4 | 1 | 12 | negative regulation of autophagy |
| GO:0030903 | 0.035 | 0.159 | 4 | 1 | 12 | notochord development |
| GO:0042033 | 0.035 | 0.159 | 4 | 1 | 12 | chemokine biosynthetic process |
| GO:0045624 | 0.035 | 0.159 | 4 | 1 | 12 | positive regulation of T-helper cell differentiation |
| GO:0046605 | 0.035 | 0.159 | 4 | 1 | 12 | regulation of centrosome cycle |
| GO:0046827 | 0.035 | 0.159 | 4 | 1 | 12 | positive regulation of protein export from nucleus |
| GO:0048016 | 0.035 | 0.159 | 4 | 1 | 12 | inositol phosphate-mediated signaling |
| GO:0090114 | 0.035 | 0.159 | 4 | 1 | 12 | COPII-coated vesicle budding |
| GO:0051338 | 0.035 | 0.833 | 179 | 160 | 493 | regulation of transferase activity |
| GO:0007266 | 0.035 | 0.612 | 28 | 20 | 77 | Rho protein signal transduction |
| GO:0045834 | 0.035 | 0.612 | 28 | 20 | 77 | positive regulation of lipid metabolic process |
| GO:0072676 | 0.035 | 0.249 | 6 | 2 | 16 | lymphocyte migration |
| GO:0030449 | 0.036 | 0.250 | 6 | 2 | 16 | regulation of complement activation |
| GO:0048246 | 0.036 | 0.250 | 6 | 2 | 16 | macrophage chemotaxis |
| GO:0048333 | 0.036 | 0.250 | 6 | 2 | 16 | mesodermal cell differentiation |
| GO:0050829 | 0.036 | 0.250 | 6 | 2 | 16 | defense response to Gram-negative bacterium |
| GO:0061082 | 0.036 | 0.250 | 6 | 2 | 16 | myeloid leukocyte cytokine production |
| GO:0050728 | 0.036 | 0.537 | 19 | 12 | 51 | negative regulation of inflammatory response |
| GO:0050727 | 0.036 | 0.508 | 16 | 10 | 44 | regulation of inflammatory response |
| GO:0001667 | 0.036 | 0.621 | 29 | 21 | 80 | ameboidal cell migration |
| GO:0032496 | 0.037 | 0.640 | 32 | 24 | 89 | response to lipopolysaccharide |
| GO:0097194 | 0.037 | 0.722 | 57 | 46 | 157 | execution phase of apoptosis |
| GO:0009968 | 0.037 | 0.827 | 161 | 143 | 443 | negative regulation of signal transduction |
| GO:0030199 | 0.037 | 0.397 | 10 | 5 | 27 | collagen fibril organization |
| GO:0051353 | 0.037 | 0.397 | 10 | 5 | 27 | positive regulation of oxidoreductase activity |
| GO:0005978 | 0.038 | 0.453 | 12 | 7 | 34 | glycogen biosynthetic process |
| GO:0008630 | 0.038 | 0.453 | 12 | 7 | 34 | intrinsic apoptotic signaling pathway in response to DNA damage |
| GO:0030888 | 0.038 | 0.453 | 12 | 7 | 34 | regulation of B cell proliferation |
| GO:0048844 | 0.038 | 0.453 | 12 | 7 | 34 | artery morphogenesis |
| GO:0030968 | 0.038 | 0.593 | 24 | 17 | 67 | endoplasmic reticulum unfolded protein response |
| GO:0006073 | 0.038 | 0.554 | 20 | 13 | 54 | cellular glucan metabolic process |
| GO:0045595 | 0.038 | 0.863 | 267 | 245 | 736 | regulation of cell differentiation |
| GO:0002443 | 0.039 | 0.478 | 14 | 8 | 37 | leukocyte mediated immunity |
| GO:0007160 | 0.040 | 0.693 | 45 | 35 | 123 | cell-matrix adhesion |
| GO:0034130 | 0.040 | 0.568 | 21 | 14 | 57 | toll-like receptor 1 signaling pathway |
| GO:2000117 | 0.040 | 0.568 | 21 | 14 | 57 | negative regulation of cysteine-type endopeptidase activity |
| GO:0044106 | 0.041 | 0.708 | 49 | 39 | 135 | cellular amine metabolic process |
| GO:0061448 | 0.041 | 0.717 | 52 | 42 | 144 | connective tissue development |
| GO:0034660 | 0.041 | 0.629 | 29 | 21 | 79 | ncRNA metabolic process |
| GO:0007492 | 0.041 | 0.482 | 13 | 8 | 37 | endoderm development |
| GO:0032623 | 0.041 | 0.482 | 13 | 8 | 37 | interleukin-2 production |
| GO:0001707 | 0.042 | 0.367 | 8 | 4 | 23 | mesoderm formation |
| GO:0042113 | 0.042 | 0.677 | 39 | 30 | 107 | B cell activation |
| GO:0032609 | 0.042 | <0.0001 | 3 | 0 | 7 | interferon-gamma production |
| GO:0051224 | 0.042 | 0.623 | 28 | 20 | 76 | negative regulation of protein transport |
| GO:0002448 | 0.042 | 0.368 | 8 | 4 | 23 | mast cell mediated immunity |
| GO:0002762 | 0.042 | 0.368 | 8 | 4 | 23 | negative regulation of myeloid leukocyte differentiation |
| GO:0006730 | 0.042 | 0.368 | 8 | 4 | 23 | one-carbon metabolic process |
| GO:0010389 | 0.042 | 0.368 | 8 | 4 | 23 | regulation of G2/M transition of mitotic cell cycle |
| GO:0010470 | 0.042 | 0.368 | 8 | 4 | 23 | regulation of gastrulation |
| GO:0043370 | 0.042 | 0.368 | 8 | 4 | 23 | regulation of CD4-positive, alpha-beta T cell differentiation |
| GO:0060334 | 0.042 | 0.368 | 8 | 4 | 23 | regulation of interferon-gamma-mediated signaling pathway |
| GO:0071230 | 0.042 | 0.368 | 8 | 4 | 23 | cellular response to amino acid stimulus |
| GO:0046632 | 0.042 | 0.435 | 11 | 6 | 30 | alpha-beta T cell differentiation |
| GO:0009612 | 0.042 | 0.591 | 23 | 16 | 63 | response to mechanical stimulus |
| GO:0048608 | 0.042 | 0.757 | 72 | 60 | 198 | reproductive structure development |
| GO:2000241 | 0.042 | 0.733 | 59 | 48 | 162 | regulation of reproductive process |
| GO:0007049 | 0.042 | 0.785 | 94 | 81 | 256 | cell cycle |
| GO:0002666 | 0.042 | <0.0001 | 3 | 0 | 7 | positive regulation of T cell tolerance induction |
| GO:0003097 | 0.042 | <0.0001 | 3 | 0 | 7 | renal water transport |
| GO:0006098 | 0.042 | <0.0001 | 3 | 0 | 7 | pentose-phosphate shunt |
| GO:0007199 | 0.042 | <0.0001 | 3 | 0 | 7 | G-protein coupled receptor signaling pathway coupled to cGMP nucleotide second messenger |
| GO:0010225 | 0.042 | <0.0001 | 3 | 0 | 7 | response to UV-C |
| GO:0016045 | 0.042 | <0.0001 | 3 | 0 | 7 | detection of bacterium |
| GO:0016114 | 0.042 | <0.0001 | 3 | 0 | 7 | terpenoid biosynthetic process |
| GO:0021978 | 0.042 | <0.0001 | 3 | 0 | 7 | telencephalon regionalization |
| GO:0030220 | 0.042 | <0.0001 | 3 | 0 | 7 | platelet formation |
| GO:0032727 | 0.042 | <0.0001 | 3 | 0 | 7 | positive regulation of interferon-alpha production |
| GO:0035313 | 0.042 | <0.0001 | 3 | 0 | 7 | wound healing, spreading of epidermal cells |
| GO:0039528 | 0.042 | <0.0001 | 3 | 0 | 7 | cytoplasmic pattern recognition receptor signaling pathway in response to virus |
| GO:0045606 | 0.042 | <0.0001 | 3 | 0 | 7 | positive regulation of epidermal cell differentiation |
| GO:0045989 | 0.042 | <0.0001 | 3 | 0 | 7 | positive regulation of striated muscle contraction |
| GO:0046931 | 0.042 | <0.0001 | 3 | 0 | 7 | pore complex assembly |
| GO:0051187 | 0.042 | <0.0001 | 3 | 0 | 7 | cofactor catabolic process |
| GO:0051531 | 0.042 | <0.0001 | 3 | 0 | 7 | NFAT protein import into nucleus |
| GO:0051770 | 0.042 | <0.0001 | 3 | 0 | 7 | positive regulation of nitric-oxide synthase biosynthetic process |
| GO:0060558 | 0.042 | <0.0001 | 3 | 0 | 7 | regulation of calcidiol 1-monooxygenase activity |
| GO:0061099 | 0.042 | <0.0001 | 3 | 0 | 7 | negative regulation of protein tyrosine kinase activity |
| GO:0070561 | 0.042 | <0.0001 | 3 | 0 | 7 | vitamin D receptor signaling pathway |
| GO:0072102 | 0.042 | <0.0001 | 3 | 0 | 7 | glomerulus morphogenesis |
| GO:0086069 | 0.042 | <0.0001 | 3 | 0 | 7 | bundle of His cell to Purkinje myocyte communication |
| GO:0090330 | 0.042 | <0.0001 | 3 | 0 | 7 | regulation of platelet aggregation |
| GO:1900024 | 0.042 | <0.0001 | 3 | 0 | 7 | regulation of substrate adhesion-dependent cell spreading |
| GO:0009056 | 0.042 | 0.895 | 485 | 457 | 1335 | catabolic process |
| GO:0043065 | 0.043 | 0.750 | 68 | 56 | 185 | positive regulation of apoptotic process |
| GO:0009891 | 0.043 | 0.880 | 342 | 318 | 942 | positive regulation of biosynthetic process |
| GO:0033628 | 0.043 | 0.437 | 11 | 6 | 30 | regulation of cell adhesion mediated by integrin |
| GO:0051145 | 0.043 | 0.437 | 11 | 6 | 30 | smooth muscle cell differentiation |
| GO:0060324 | 0.043 | 0.437 | 11 | 6 | 30 | face development |
| GO:0034101 | 0.043 | 0.632 | 29 | 21 | 79 | erythrocyte homeostasis |
| GO:0001890 | 0.044 | 0.504 | 15 | 9 | 40 | placenta development |
| GO:0045862 | 0.044 | 0.552 | 18 | 12 | 50 | positive regulation of proteolysis |
| GO:0006886 | 0.045 | 0.799 | 106 | 92 | 290 | intracellular protein transport |
| GO:0010608 | 0.045 | 0.780 | 87 | 74 | 239 | posttranscriptional regulation of gene expression |
| GO:0003158 | 0.045 | 0.507 | 15 | 9 | 40 | endothelium development |
| GO:0034614 | 0.045 | 0.606 | 24 | 17 | 66 | cellular response to reactive oxygen species |
| GO:0065003 | 0.046 | 0.864 | 246 | 225 | 676 | macromolecular complex assembly |
| GO:0002675 | 0.046 | 0.328 | 7 | 3 | 19 | positive regulation of acute inflammatory response |
| GO:0030574 | 0.046 | 0.328 | 7 | 3 | 19 | collagen catabolic process |
| GO:0030851 | 0.046 | 0.328 | 7 | 3 | 19 | granulocyte differentiation |
| GO:0032735 | 0.046 | 0.328 | 7 | 3 | 19 | positive regulation of interleukin-12 production |
| GO:0043616 | 0.046 | 0.328 | 7 | 3 | 19 | keratinocyte proliferation |
| GO:0048557 | 0.046 | 0.328 | 7 | 3 | 19 | embryonic digestive tract morphogenesis |
| GO:0060441 | 0.046 | 0.328 | 7 | 3 | 19 | epithelial tube branching involved in lung morphogenesis |
| GO:0061418 | 0.046 | 0.328 | 7 | 3 | 19 | regulation of transcription from RNA polymerase II promoter in response to hypoxia |
| GO:1901342 | 0.046 | 0.716 | 49 | 39 | 134 | regulation of vasculature development |
| GO:0032956 | 0.047 | 0.719 | 50 | 40 | 137 | regulation of actin cytoskeleton organization |
| GO:0043406 | 0.047 | 0.722 | 51 | 41 | 140 | positive regulation of MAP kinase activity |
| GO:0005976 | 0.047 | 0.616 | 25 | 18 | 69 | polysaccharide metabolic process |
| GO:0051336 | 0.047 | 0.767 | 75 | 63 | 204 | regulation of hydrolase activity |
| GO:0031663 | 0.048 | 0.471 | 12 | 7 | 33 | lipopolysaccharide-mediated signaling pathway |
| GO:0030029 | 0.048 | 0.816 | 124 | 109 | 341 | actin filament-based process |
| GO:0010811 | 0.048 | 0.529 | 16 | 10 | 43 | positive regulation of cell-substrate adhesion |
| GO:0006611 | 0.049 | 0.415 | 9 | 5 | 26 | protein export from nucleus |
| GO:0009057 | 0.049 | 0.723 | 51 | 41 | 138 | macromolecule catabolic process |
| GO:0001569 | 0.049 | 0.416 | 9 | 5 | 26 | patterning of blood vessels |
| GO:1901564 | 0.049 | 0.892 | 404 | 379 | 1112 | organonitrogen compound metabolic process |
| GO:0048771 | 0.049 | 0.684 | 37 | 29 | 103 | tissue remodeling |
| GO:0006639 | 0.049 | 0.635 | 27 | 20 | 75 | acylglycerol metabolic process |
| GO:0051090 | 0.050 | 0.785 | 86 | 74 | 238 | regulation of sequence-specific DNA binding transcription factor activity |
| GO:0048754 | 0.050 | 0.648 | 30 | 22 | 81 | branching morphogenesis of an epithelial tube |
| GO:0010575 | 0.050 | 0.269 | 5 | 2 | 15 | positive regulation vascular endothelial growth factor production |
| GO:0032673 | 0.050 | 0.269 | 5 | 2 | 15 | regulation of interleukin-4 production |
| GO:0033574 | 0.050 | 0.269 | 5 | 2 | 15 | response to testosterone stimulus |
| GO:0042346 | 0.050 | 0.269 | 5 | 2 | 15 | positive regulation of NF-kappaB import into nucleus |
| GO:0043304 | 0.050 | 0.269 | 5 | 2 | 15 | regulation of mast cell degranulation |
| GO:0043928 | 0.050 | 0.269 | 5 | 2 | 15 | exonucleolytic nuclear-transcribed mRNA catabolic process involved in deadenylation-dependent decay |
| GO:0046885 | 0.050 | 0.269 | 5 | 2 | 15 | regulation of hormone biosynthetic process |
| GO:0050691 | 0.050 | 0.269 | 5 | 2 | 15 | regulation of defense response to virus by host |
| GO:0051668 | 0.050 | 0.269 | 5 | 2 | 15 | localization within membrane |
| GO:2001021 | 0.050 | 0.269 | 5 | 2 | 15 | negative regulation of response to DNA damage stimulus |
