# Supplementary material for: Hidden Treasures in “Ancient” Microarrays: Gene-Expression Portrays Biology and Potential Resistance Pathways of Major Lung Cancer Subtypes and Normal Tissue
Source: Front Oncol. 2014 Sep 29;4:251. doi: 10.3389/fonc.2014.00251 (PMC4178426; doi:10.3389/fonc.2014.00251)
Supplement: Figure S1 — Clinically relevant differentially expressed pathways in each tissue type versus the rest. The pathway maps are arranged according to the scheme in the left-top corner. Red indicates overexpression in genes, dark green downregulation. In the Cell Cycle pathway, tumor suppressor genes were overexpressed in normal lung tissue while oncogenes and tumor driving cyclins were overexpressed in cancers. The gene thymidylate synthase (TYMS or TS, belonging to the pathway “one carbon pool by folate”) is relevant for tumor growth and is also a treatment target. Notably, TYMS was overexpressed only in the tumors that are generally refractory to the drug pemetrexed, as the squamous and the small-cell lung cancer. TYMS was not overexpressed in mesothelioma, but it is known that TYMS expression is highly variable in this cancer. In the ERBB pathway, the ERBB2/HER2 and ERBB3/HER3 were overexpressed in adenocarcinoma, while the ERBB4 was overexpressed in the carcinoids. [file Data_Sheet_1.ZIP › File S3.ZIP!/GO Analysis/Normals vs Rest Enrichment Analysis/Overrepresented GO terms of Differentially Expressed Genes.html]

Gene to GO BP test for over-representation

| GOBPID | Pvalue | OddsRatio | ExpCount | Count | Size | Term |
| --- | --- | --- | --- | --- | --- | --- |
| GO:0030036 | <0.0001 | 1.927 | 106 | 152 | 297 | actin cytoskeleton organization |
| GO:0030029 | <0.0001 | 1.808 | 122 | 169 | 341 | actin filament-based process |
| GO:0016043 | <0.0001 | 1.259 | 957 | 1051 | 2670 | cellular component organization |
| GO:0071840 | <0.0001 | 1.257 | 974 | 1068 | 2717 | cellular component organization or biogenesis |
| GO:0044763 | <0.0001 | 1.285 | 2075 | 2161 | 5791 | single-organism cellular process |
| GO:0007010 | <0.0001 | 1.512 | 195 | 245 | 544 | cytoskeleton organization |
| GO:0048518 | <0.0001 | 1.255 | 870 | 959 | 2429 | positive regulation of biological process |
| GO:0071241 | <0.0001 | 2.836 | 28 | 47 | 77 | cellular response to inorganic substance |
| GO:0071248 | <0.0001 | 2.908 | 26 | 45 | 73 | cellular response to metal ion |
| GO:0044767 | <0.0001 | 1.248 | 851 | 936 | 2374 | single-organism developmental process |
| GO:0050793 | <0.0001 | 1.339 | 372 | 434 | 1038 | regulation of developmental process |
| GO:0016477 | <0.0001 | 1.407 | 237 | 286 | 661 | cell migration |
| GO:0006928 | <0.0001 | 1.349 | 318 | 374 | 888 | cellular component movement |
| GO:0048522 | <0.0001 | 1.235 | 796 | 874 | 2221 | positive regulation of cellular process |
| GO:0022607 | <0.0001 | 1.325 | 362 | 420 | 1009 | cellular component assembly |
| GO:0007015 | <0.0001 | 1.933 | 58 | 83 | 161 | actin filament organization |
| GO:0048856 | <0.0001 | 1.221 | 949 | 1030 | 2648 | anatomical structure development |
| GO:0048731 | <0.0001 | 1.226 | 845 | 923 | 2358 | system development |
| GO:0070887 | <0.0001 | 1.282 | 471 | 535 | 1315 | cellular response to chemical stimulus |
| GO:0006259 | <0.0001 | 1.447 | 187 | 230 | 522 | DNA metabolic process |
| GO:0006261 | <0.0001 | 2.566 | 27 | 44 | 75 | DNA-dependent DNA replication |
| GO:0044085 | <0.0001 | 1.298 | 388 | 445 | 1082 | cellular component biogenesis |
| GO:0065003 | <0.0001 | 1.376 | 242 | 289 | 676 | macromolecular complex assembly |
| GO:0044699 | <0.0001 | 1.263 | 2270 | 2334 | 6334 | single-organism process |
| GO:0007051 | <0.0001 | 2.872 | 20 | 35 | 57 | spindle organization |
| GO:0002376 | <0.0001 | 1.260 | 484 | 545 | 1352 | immune system process |
| GO:0050776 | <0.0001 | 1.478 | 145 | 181 | 405 | regulation of immune response |
| GO:0006461 | <0.0001 | 1.404 | 194 | 235 | 542 | protein complex assembly |
| GO:0006541 | <0.0001 | 8.393 | 6 | 14 | 17 | glutamine metabolic process |
| GO:0070271 | <0.0001 | 1.395 | 195 | 235 | 544 | protein complex biogenesis |
| GO:0048870 | <0.0001 | 1.344 | 250 | 295 | 699 | cell motility |
| GO:0051674 | <0.0001 | 1.344 | 250 | 295 | 699 | localization of cell |
| GO:0048513 | <0.0001 | 1.226 | 613 | 677 | 1712 | organ development |
| GO:0051128 | <0.0001 | 1.299 | 319 | 368 | 891 | regulation of cellular component organization |
| GO:0006996 | <0.0001 | 1.244 | 493 | 551 | 1376 | organelle organization |
| GO:0001568 | <0.0001 | 1.451 | 141 | 174 | 393 | blood vessel development |
| GO:0007049 | <0.0001 | 1.285 | 330 | 378 | 921 | cell cycle |
| GO:0002682 | <0.0001 | 1.336 | 232 | 273 | 648 | regulation of immune system process |
| GO:0043933 | <0.0001 | 1.290 | 311 | 357 | 867 | macromolecular complex subunit organization |
| GO:0040011 | <0.0001 | 1.271 | 341 | 388 | 951 | locomotion |
| GO:0008283 | <0.0001 | 1.247 | 417 | 468 | 1163 | cell proliferation |
| GO:0022403 | <0.0001 | 1.353 | 201 | 238 | 560 | cell cycle phase |
| GO:0006357 | <0.0001 | 1.279 | 319 | 365 | 891 | regulation of transcription from RNA polymerase II promoter |
| GO:0009888 | <0.0001 | 1.276 | 323 | 369 | 902 | tissue development |
| GO:0009893 | <0.0001 | 1.221 | 524 | 579 | 1461 | positive regulation of metabolic process |
| GO:0032970 | <0.0001 | 1.723 | 57 | 78 | 160 | regulation of actin filament-based process |
| GO:0048514 | <0.0001 | 1.457 | 123 | 152 | 342 | blood vessel morphogenesis |
| GO:0006955 | 0.001 | 1.292 | 281 | 324 | 785 | immune response |
| GO:0034622 | 0.001 | 1.451 | 124 | 153 | 345 | cellular macromolecular complex assembly |
| GO:0048869 | 0.001 | 1.199 | 666 | 726 | 1859 | cellular developmental process |
| GO:0031100 | 0.001 | 3.087 | 14 | 24 | 38 | organ regeneration |
| GO:0001944 | 0.001 | 1.407 | 147 | 179 | 411 | vasculature development |
| GO:0007052 | 0.001 | 3.600 | 11 | 20 | 30 | mitotic spindle organization |
| GO:0051493 | 0.001 | 1.634 | 69 | 91 | 192 | regulation of cytoskeleton organization |
| GO:0032956 | 0.001 | 1.784 | 49 | 68 | 137 | regulation of actin cytoskeleton organization |
| GO:0010604 | 0.001 | 1.223 | 485 | 538 | 1354 | positive regulation of macromolecule metabolic process |
| GO:0000278 | 0.001 | 1.351 | 191 | 226 | 532 | mitotic cell cycle |
| GO:0030168 | 0.001 | 1.632 | 68 | 90 | 190 | platelet activation |
| GO:0030154 | 0.001 | 1.198 | 639 | 697 | 1783 | cell differentiation |
| GO:0050663 | 0.001 | 2.319 | 23 | 36 | 64 | cytokine secretion |
| GO:0007275 | 0.001 | 1.173 | 946 | 1011 | 2641 | multicellular organismal development |
| GO:0006334 | 0.001 | 2.655 | 17 | 28 | 47 | nucleosome assembly |
| GO:0002480 | 0.001 | Inf | 3 | 7 | 7 | antigen processing and presentation of exogenous peptide antigen via MHC class I, TAP-independent |
| GO:0022402 | 0.001 | 1.297 | 251 | 290 | 700 | cell cycle process |
| GO:0071822 | 0.001 | 1.295 | 254 | 293 | 708 | protein complex subunit organization |
| GO:0009611 | 0.001 | 1.268 | 306 | 349 | 855 | response to wounding |
| GO:0032502 | 0.001 | 1.167 | 1041 | 1106 | 2904 | developmental process |
| GO:0002886 | 0.001 | 5.034 | 7 | 14 | 19 | regulation of myeloid leukocyte mediated immunity |
| GO:0043300 | 0.001 | 5.034 | 7 | 14 | 19 | regulation of leukocyte degranulation |
| GO:0002684 | 0.001 | 1.383 | 151 | 182 | 422 | positive regulation of immune system process |
| GO:0008360 | 0.001 | 2.114 | 27 | 41 | 76 | regulation of cell shape |
| GO:0061061 | 0.001 | 1.423 | 126 | 154 | 351 | muscle structure development |
| GO:0043623 | 0.001 | 1.561 | 77 | 99 | 214 | cellular protein complex assembly |
| GO:0051726 | 0.001 | 1.335 | 191 | 225 | 533 | regulation of cell cycle |
| GO:0031589 | 0.001 | 1.632 | 62 | 82 | 173 | cell-substrate adhesion |
| GO:0006270 | 0.001 | 3.822 | 9 | 17 | 25 | DNA replication initiation |
| GO:0031325 | 0.001 | 1.208 | 498 | 549 | 1391 | positive regulation of cellular metabolic process |
| GO:0050867 | 0.001 | 1.583 | 69 | 90 | 193 | positive regulation of cell activation |
| GO:0050878 | 0.001 | 1.340 | 173 | 205 | 484 | regulation of body fluid levels |
| GO:0051094 | 0.001 | 1.322 | 190 | 223 | 531 | positive regulation of developmental process |
| GO:0045597 | 0.001 | 1.378 | 140 | 168 | 390 | positive regulation of cell differentiation |
| GO:0045859 | 0.002 | 1.345 | 164 | 194 | 457 | regulation of protein kinase activity |
| GO:0050707 | 0.002 | 2.350 | 19 | 30 | 53 | regulation of cytokine secretion |
| GO:0022603 | 0.002 | 1.347 | 160 | 190 | 447 | regulation of anatomical structure morphogenesis |
| GO:0050891 | 0.002 | 5.391 | 6 | 12 | 16 | multicellular organismal water homeostasis |
| GO:0019222 | 0.002 | 1.154 | 1059 | 1120 | 2955 | regulation of metabolic process |
| GO:0071294 | 0.002 | 14.363 | 3 | 8 | 9 | cellular response to zinc ion |
| GO:1901654 | 0.002 | 2.189 | 22 | 34 | 62 | response to ketone |
| GO:0000819 | 0.002 | 2.907 | 12 | 21 | 34 | sister chromatid segregation |
| GO:0071103 | 0.002 | 1.806 | 38 | 53 | 106 | DNA conformation change |
| GO:0009127 | 0.002 | 4.673 | 6 | 13 | 18 | purine nucleoside monophosphate biosynthetic process |
| GO:0009168 | 0.002 | 4.673 | 6 | 13 | 18 | purine ribonucleoside monophosphate biosynthetic process |
| GO:0065008 | 0.002 | 1.175 | 661 | 714 | 1845 | regulation of biological quality |
| GO:0000070 | 0.002 | 2.999 | 11 | 20 | 32 | mitotic sister chromatid segregation |
| GO:0006952 | 0.002 | 1.252 | 287 | 325 | 801 | defense response |
| GO:0001775 | 0.002 | 1.291 | 215 | 248 | 599 | cell activation |
| GO:0065007 | 0.002 | 1.157 | 1883 | 1941 | 5255 | biological regulation |
| GO:0006281 | 0.002 | 1.469 | 89 | 111 | 248 | DNA repair |
| GO:0033622 | 0.002 | Inf | 2 | 6 | 6 | integrin activation |
| GO:0033623 | 0.002 | Inf | 2 | 6 | 6 | regulation of integrin activation |
| GO:0006366 | 0.002 | 1.219 | 371 | 413 | 1036 | transcription from RNA polymerase II promoter |
| GO:0051017 | 0.002 | 2.103 | 23 | 35 | 65 | actin filament bundle assembly |
| GO:0007599 | 0.002 | 1.347 | 147 | 174 | 409 | hemostasis |
| GO:2000026 | 0.002 | 1.243 | 292 | 329 | 814 | regulation of multicellular organismal development |
| GO:0006067 | 0.002 | 8.080 | 4 | 9 | 11 | ethanol metabolic process |
| GO:0060326 | 0.002 | 1.674 | 47 | 63 | 131 | cell chemotaxis |
| GO:0065009 | 0.003 | 1.192 | 465 | 510 | 1299 | regulation of molecular function |
| GO:0000087 | 0.003 | 1.476 | 81 | 101 | 225 | M phase of mitotic cell cycle |
| GO:0006323 | 0.003 | 1.953 | 27 | 39 | 75 | DNA packaging |
| GO:0008154 | 0.003 | 1.845 | 32 | 45 | 89 | actin polymerization or depolymerization |
| GO:0048468 | 0.003 | 1.208 | 384 | 425 | 1072 | cell development |
| GO:0006950 | 0.003 | 1.158 | 745 | 797 | 2079 | response to stress |
| GO:0043549 | 0.003 | 1.307 | 172 | 201 | 481 | regulation of kinase activity |
| GO:0042221 | 0.003 | 1.157 | 744 | 796 | 2077 | response to chemical stimulus |
| GO:0050900 | 0.003 | 1.470 | 79 | 99 | 221 | leukocyte migration |
| GO:0034728 | 0.003 | 2.042 | 23 | 34 | 64 | nucleosome organization |
| GO:0080090 | 0.003 | 1.145 | 946 | 1001 | 2640 | regulation of primary metabolic process |
| GO:0002446 | 0.003 | 4.940 | 5 | 11 | 15 | neutrophil mediated immunity |
| GO:0043304 | 0.003 | 4.940 | 5 | 11 | 15 | regulation of mast cell degranulation |
| GO:0072001 | 0.004 | 1.520 | 66 | 84 | 184 | renal system development |
| GO:0009124 | 0.004 | 2.943 | 10 | 18 | 29 | nucleoside monophosphate biosynthetic process |
| GO:0032355 | 0.004 | 2.050 | 22 | 33 | 62 | response to estradiol stimulus |
| GO:0009156 | 0.004 | 3.056 | 10 | 17 | 27 | ribonucleoside monophosphate biosynthetic process |
| GO:0007596 | 0.004 | 1.328 | 145 | 171 | 405 | blood coagulation |
| GO:0032413 | 0.004 | 4.312 | 6 | 12 | 17 | negative regulation of ion transmembrane transporter activity |
| GO:0036296 | 0.004 | 4.312 | 6 | 12 | 17 | response to increased oxygen levels |
| GO:0055093 | 0.004 | 4.312 | 6 | 12 | 17 | response to hyperoxia |
| GO:0012501 | 0.004 | 1.195 | 407 | 448 | 1137 | programmed cell death |
| GO:0006915 | 0.004 | 1.196 | 405 | 445 | 1129 | apoptotic process |
| GO:0030851 | 0.004 | 3.893 | 7 | 13 | 19 | granulocyte differentiation |
| GO:0032410 | 0.004 | 3.594 | 8 | 14 | 21 | negative regulation of transporter activity |
| GO:0000079 | 0.004 | 2.058 | 22 | 32 | 60 | regulation of cyclin-dependent protein kinase activity |
| GO:0009653 | 0.004 | 1.175 | 514 | 558 | 1434 | anatomical structure morphogenesis |
| GO:0035357 | 0.004 | 12.563 | 3 | 7 | 8 | peroxisome proliferator activated receptor signaling pathway |
| GO:0009617 | 0.004 | 1.433 | 86 | 106 | 240 | response to bacterium |
| GO:0030041 | 0.004 | 1.902 | 27 | 38 | 74 | actin filament polymerization |
| GO:0050778 | 0.004 | 1.404 | 96 | 117 | 268 | positive regulation of immune response |
| GO:0001655 | 0.005 | 1.451 | 78 | 97 | 218 | urogenital system development |
| GO:0009887 | 0.005 | 1.265 | 205 | 234 | 571 | organ morphogenesis |
| GO:0050817 | 0.005 | 1.317 | 146 | 171 | 407 | coagulation |
| GO:0007059 | 0.005 | 1.849 | 28 | 40 | 79 | chromosome segregation |
| GO:0060537 | 0.005 | 1.413 | 90 | 110 | 251 | muscle tissue development |
| GO:0032675 | 0.005 | 2.228 | 17 | 26 | 47 | regulation of interleukin-6 production |
| GO:0006468 | 0.005 | 1.215 | 307 | 342 | 857 | protein phosphorylation |
| GO:0010035 | 0.005 | 1.391 | 98 | 119 | 274 | response to inorganic substance |
| GO:0051259 | 0.005 | 1.437 | 81 | 100 | 226 | protein oligomerization |
| GO:0048871 | 0.005 | 1.654 | 41 | 55 | 115 | multicellular organismal homeostasis |
| GO:0001894 | 0.005 | 1.803 | 30 | 42 | 84 | tissue homeostasis |
| GO:0006333 | 0.005 | 1.908 | 25 | 36 | 70 | chromatin assembly or disassembly |
| GO:0010629 | 0.005 | 1.252 | 218 | 248 | 609 | negative regulation of gene expression |
| GO:0031497 | 0.005 | 2.088 | 19 | 29 | 54 | chromatin assembly |
| GO:0042060 | 0.005 | 1.280 | 177 | 204 | 494 | wound healing |
| GO:0061138 | 0.006 | 1.555 | 53 | 68 | 147 | morphogenesis of a branching epithelium |
| GO:0043302 | 0.006 | 7.180 | 4 | 8 | 10 | positive regulation of leukocyte degranulation |
| GO:0060347 | 0.006 | 7.180 | 4 | 8 | 10 | heart trabecula formation |
| GO:0014070 | 0.006 | 1.293 | 159 | 185 | 445 | response to organic cyclic compound |
| GO:0071310 | 0.006 | 1.192 | 372 | 409 | 1038 | cellular response to organic substance |
| GO:0030952 | 0.006 | Inf | 2 | 5 | 5 | establishment or maintenance of cytoskeleton polarity |
| GO:0031643 | 0.006 | Inf | 2 | 5 | 5 | positive regulation of myelination |
| GO:0035791 | 0.006 | Inf | 2 | 5 | 5 | platelet-derived growth factor receptor-beta signaling pathway |
| GO:0006260 | 0.006 | 1.474 | 67 | 84 | 187 | DNA replication |
| GO:0009306 | 0.006 | 1.610 | 44 | 58 | 123 | protein secretion |
| GO:0002520 | 0.006 | 1.291 | 159 | 184 | 443 | immune system development |
| GO:0050715 | 0.006 | 2.299 | 15 | 23 | 41 | positive regulation of cytokine secretion |
| GO:0045785 | 0.006 | 1.697 | 35 | 48 | 99 | positive regulation of cell adhesion |
| GO:0045646 | 0.006 | 2.778 | 10 | 17 | 28 | regulation of erythrocyte differentiation |
| GO:0051270 | 0.006 | 1.314 | 137 | 160 | 381 | regulation of cellular component movement |
| GO:0022008 | 0.006 | 1.211 | 295 | 328 | 823 | neurogenesis |
| GO:0051338 | 0.006 | 1.273 | 177 | 203 | 493 | regulation of transferase activity |
| GO:0001525 | 0.006 | 1.370 | 101 | 121 | 281 | angiogenesis |
| GO:0050789 | 0.007 | 1.131 | 1777 | 1828 | 4958 | regulation of biological process |
| GO:0031323 | 0.007 | 1.132 | 950 | 1000 | 2650 | regulation of cellular metabolic process |
| GO:0001569 | 0.007 | 2.876 | 9 | 16 | 26 | patterning of blood vessels |
| GO:0003091 | 0.007 | 5.386 | 4 | 9 | 12 | renal water homeostasis |
| GO:0060314 | 0.007 | 5.386 | 4 | 9 | 12 | regulation of ryanodine-sensitive calcium-release channel activity |
| GO:0051174 | 0.007 | 1.201 | 323 | 357 | 901 | regulation of phosphorus metabolic process |
| GO:0051100 | 0.007 | 2.000 | 20 | 30 | 57 | negative regulation of binding |
| GO:0002444 | 0.007 | 2.328 | 14 | 22 | 39 | myeloid leukocyte mediated immunity |
| GO:0072593 | 0.007 | 1.695 | 35 | 47 | 97 | reactive oxygen species metabolic process |
| GO:0032635 | 0.007 | 2.127 | 17 | 26 | 48 | interleukin-6 production |
| GO:0002696 | 0.007 | 1.467 | 66 | 82 | 183 | positive regulation of leukocyte activation |
| GO:0010628 | 0.007 | 1.211 | 285 | 317 | 795 | positive regulation of gene expression |
| GO:0006929 | 0.007 | 4.489 | 5 | 10 | 14 | substrate-dependent cell migration |
| GO:0060216 | 0.007 | 4.489 | 5 | 10 | 14 | definitive hemopoiesis |
| GO:0030104 | 0.007 | 3.144 | 8 | 14 | 22 | water homeostasis |
| GO:0006935 | 0.007 | 1.272 | 171 | 196 | 476 | chemotaxis |
| GO:0042330 | 0.007 | 1.272 | 171 | 196 | 476 | taxis |
| GO:0002253 | 0.007 | 1.420 | 77 | 95 | 216 | activation of immune response |
| GO:0009719 | 0.007 | 1.201 | 310 | 343 | 865 | response to endogenous stimulus |
| GO:0033006 | 0.008 | 3.951 | 6 | 11 | 16 | regulation of mast cell activation involved in immune response |
| GO:0035909 | 0.008 | 3.951 | 6 | 11 | 16 | aorta morphogenesis |
| GO:0051983 | 0.008 | 3.951 | 6 | 11 | 16 | regulation of chromosome segregation |
| GO:0061384 | 0.008 | 3.951 | 6 | 11 | 16 | heart trabecula morphogenesis |
| GO:0035904 | 0.008 | 3.592 | 6 | 12 | 18 | aorta development |
| GO:0000236 | 0.008 | 2.142 | 16 | 25 | 46 | mitotic prometaphase |
| GO:0000122 | 0.008 | 1.313 | 130 | 152 | 362 | negative regulation of transcription from RNA polymerase II promoter |
| GO:0008064 | 0.008 | 1.854 | 25 | 35 | 69 | regulation of actin polymerization or depolymerization |
| GO:0044707 | 0.008 | 1.122 | 1275 | 1327 | 3559 | single-multicellular organism process |
| GO:0031327 | 0.008 | 1.223 | 247 | 277 | 690 | negative regulation of cellular biosynthetic process |
| GO:0033993 | 0.008 | 1.278 | 161 | 185 | 448 | response to lipid |
| GO:0009123 | 0.008 | 2.397 | 13 | 20 | 35 | nucleoside monophosphate metabolic process |
| GO:0000280 | 0.008 | 1.412 | 78 | 96 | 219 | nuclear division |
| GO:0007067 | 0.008 | 1.412 | 78 | 96 | 219 | mitosis |
| GO:0048519 | 0.008 | 1.135 | 783 | 829 | 2184 | negative regulation of biological process |
| GO:0050708 | 0.008 | 1.690 | 33 | 45 | 93 | regulation of protein secretion |
| GO:0045595 | 0.008 | 1.213 | 264 | 294 | 736 | regulation of cell differentiation |
| GO:0050714 | 0.008 | 1.855 | 24 | 34 | 67 | positive regulation of protein secretion |
| GO:0032501 | 0.009 | 1.120 | 1313 | 1364 | 3664 | multicellular organismal process |
| GO:0006954 | 0.009 | 1.300 | 135 | 157 | 376 | inflammatory response |
| GO:0006310 | 0.009 | 1.536 | 49 | 63 | 137 | DNA recombination |
| GO:0034329 | 0.009 | 1.536 | 49 | 63 | 137 | cell junction assembly |
| GO:0060255 | 0.009 | 1.128 | 904 | 952 | 2524 | regulation of macromolecule metabolic process |
| GO:0010038 | 0.009 | 1.436 | 69 | 85 | 192 | response to metal ion |
| GO:0030030 | 0.009 | 1.224 | 234 | 262 | 652 | cell projection organization |
| GO:0048699 | 0.009 | 1.205 | 278 | 309 | 777 | generation of neurons |
| GO:0001932 | 0.009 | 1.230 | 219 | 246 | 610 | regulation of protein phosphorylation |
| GO:0019220 | 0.009 | 1.191 | 320 | 352 | 892 | regulation of phosphate metabolic process |
| GO:0030595 | 0.009 | 1.613 | 39 | 51 | 108 | leukocyte chemotaxis |
| GO:0030334 | 0.009 | 1.320 | 116 | 137 | 325 | regulation of cell migration |
| GO:0010033 | 0.010 | 1.153 | 527 | 567 | 1472 | response to organic substance |
| GO:0001763 | 0.010 | 1.487 | 56 | 70 | 155 | morphogenesis of a branching structure |
| GO:0000279 | 0.010 | 1.324 | 113 | 133 | 315 | M phase |
| GO:0002576 | 0.010 | 1.755 | 28 | 38 | 77 | platelet degranulation |
| GO:0071346 | 0.010 | 1.755 | 28 | 38 | 77 | cellular response to interferon-gamma |
| GO:0030832 | 0.010 | 1.801 | 25 | 35 | 70 | regulation of actin filament length |
| GO:0031399 | 0.010 | 1.203 | 274 | 304 | 765 | regulation of protein modification process |
| GO:0006268 | 0.010 | 10.765 | 3 | 6 | 7 | DNA unwinding involved in replication |
| GO:0045780 | 0.010 | 10.765 | 3 | 6 | 7 | positive regulation of bone resorption |
| GO:0046852 | 0.010 | 10.765 | 3 | 6 | 7 | positive regulation of bone remodeling |
| GO:0072239 | 0.010 | 10.765 | 3 | 6 | 7 | metanephric glomerulus vasculature development |
| GO:0097305 | 0.011 | 1.422 | 69 | 85 | 193 | response to alcohol |
| GO:0001933 | 0.011 | 1.504 | 51 | 65 | 143 | negative regulation of protein phosphorylation |
| GO:0009891 | 0.011 | 1.182 | 338 | 370 | 942 | positive regulation of biosynthetic process |
| GO:0030198 | 0.011 | 1.483 | 55 | 69 | 153 | extracellular matrix organization |
| GO:0043062 | 0.011 | 1.483 | 55 | 69 | 153 | extracellular structure organization |
| GO:0045087 | 0.011 | 1.283 | 140 | 162 | 391 | innate immune response |
| GO:0032091 | 0.011 | 2.614 | 10 | 16 | 27 | negative regulation of protein binding |
| GO:0060396 | 0.011 | 2.614 | 10 | 16 | 27 | growth hormone receptor signaling pathway |
| GO:0009890 | 0.011 | 1.209 | 252 | 280 | 702 | negative regulation of biosynthetic process |
| GO:0032231 | 0.011 | 2.221 | 14 | 21 | 38 | regulation of actin filament bundle assembly |
| GO:0002764 | 0.012 | 1.424 | 67 | 82 | 186 | immune response-regulating signaling pathway |
| GO:0034097 | 0.012 | 1.276 | 144 | 166 | 402 | response to cytokine stimulus |
| GO:0032943 | 0.012 | 1.489 | 52 | 66 | 146 | mononuclear cell proliferation |
| GO:0050896 | 0.012 | 1.115 | 1537 | 1585 | 4288 | response to stimulus |
| GO:0051301 | 0.012 | 1.321 | 108 | 127 | 301 | cell division |
| GO:0014706 | 0.012 | 1.363 | 87 | 104 | 242 | striated muscle tissue development |
| GO:0007178 | 0.012 | 1.398 | 73 | 89 | 204 | transmembrane receptor protein serine/threonine kinase signaling pathway |
| GO:0051272 | 0.012 | 1.398 | 73 | 89 | 204 | positive regulation of cellular component movement |
| GO:0048661 | 0.012 | 2.247 | 13 | 20 | 36 | positive regulation of smooth muscle cell proliferation |
| GO:0048534 | 0.012 | 1.265 | 151 | 173 | 421 | hematopoietic or lymphoid organ development |
| GO:0045893 | 0.013 | 1.201 | 260 | 288 | 725 | positive regulation of transcription, DNA-dependent |
| GO:0051254 | 0.013 | 1.195 | 274 | 303 | 765 | positive regulation of RNA metabolic process |
| GO:0050865 | 0.013 | 1.329 | 101 | 119 | 281 | regulation of cell activation |
| GO:0007517 | 0.013 | 1.344 | 92 | 110 | 258 | muscle organ development |
| GO:0018198 | 0.013 | 6.280 | 3 | 7 | 9 | peptidyl-cysteine modification |
| GO:0021884 | 0.013 | 6.280 | 3 | 7 | 9 | forebrain neuron development |
| GO:0030213 | 0.013 | 6.280 | 3 | 7 | 9 | hyaluronan biosynthetic process |
| GO:0033008 | 0.013 | 6.280 | 3 | 7 | 9 | positive regulation of mast cell activation involved in immune response |
| GO:0042693 | 0.013 | 6.280 | 3 | 7 | 9 | muscle cell fate commitment |
| GO:0043306 | 0.013 | 6.280 | 3 | 7 | 9 | positive regulation of mast cell degranulation |
| GO:0045628 | 0.013 | 6.280 | 3 | 7 | 9 | regulation of T-helper 2 cell differentiation |
| GO:0007399 | 0.013 | 1.157 | 430 | 465 | 1201 | nervous system development |
| GO:0006690 | 0.013 | 2.068 | 15 | 23 | 43 | icosanoid metabolic process |
| GO:0010811 | 0.013 | 2.068 | 15 | 23 | 43 | positive regulation of cell-substrate adhesion |
| GO:1901568 | 0.013 | 2.068 | 15 | 23 | 43 | fatty acid derivative metabolic process |
| GO:0006636 | 0.013 | 2.277 | 12 | 19 | 34 | unsaturated fatty acid biosynthetic process |
| GO:1900077 | 0.013 | 2.919 | 8 | 13 | 21 | negative regulation of cellular response to insulin stimulus |
| GO:0009987 | 0.013 | 1.226 | 2597 | 2622 | 7246 | cellular process |
| GO:0030155 | 0.014 | 1.377 | 77 | 93 | 215 | regulation of cell adhesion |
| GO:0046849 | 0.014 | 1.949 | 18 | 26 | 50 | bone remodeling |
| GO:0072358 | 0.014 | 1.217 | 214 | 239 | 596 | cardiovascular system development |
| GO:0072359 | 0.014 | 1.217 | 214 | 239 | 596 | circulatory system development |
| GO:0031328 | 0.014 | 1.175 | 332 | 363 | 927 | positive regulation of cellular biosynthetic process |
| GO:0060249 | 0.014 | 1.470 | 53 | 66 | 147 | anatomical structure homeostasis |
| GO:0034330 | 0.014 | 1.461 | 54 | 68 | 152 | cell junction organization |
| GO:0051924 | 0.014 | 1.577 | 38 | 49 | 105 | regulation of calcium ion transport |
| GO:0061041 | 0.014 | 1.708 | 27 | 37 | 76 | regulation of wound healing |
| GO:0016338 | 0.014 | 4.786 | 4 | 8 | 11 | calcium-independent cell-cell adhesion |
| GO:0021591 | 0.014 | 4.786 | 4 | 8 | 11 | ventricular system development |
| GO:0035306 | 0.014 | 4.786 | 4 | 8 | 11 | positive regulation of dephosphorylation |
| GO:0046040 | 0.014 | 4.786 | 4 | 8 | 11 | IMP metabolic process |
| GO:0006284 | 0.014 | 2.311 | 11 | 18 | 32 | base-excision repair |
| GO:0007566 | 0.014 | 2.311 | 11 | 18 | 32 | embryo implantation |
| GO:0014812 | 0.014 | 2.311 | 11 | 18 | 32 | muscle cell migration |
| GO:0046456 | 0.014 | 2.311 | 11 | 18 | 32 | icosanoid biosynthetic process |
| GO:0060416 | 0.014 | 2.311 | 11 | 18 | 32 | response to growth hormone stimulus |
| GO:1901570 | 0.014 | 2.311 | 11 | 18 | 32 | fatty acid derivative biosynthetic process |
| GO:0070206 | 0.014 | 3.292 | 6 | 11 | 17 | protein trimerization |
| GO:0022604 | 0.015 | 1.370 | 78 | 94 | 218 | regulation of cell morphogenesis |
| GO:0010557 | 0.015 | 1.180 | 307 | 337 | 858 | positive regulation of macromolecule biosynthetic process |
| GO:0043067 | 0.015 | 1.175 | 323 | 353 | 901 | regulation of programmed cell death |
| GO:0072224 | 0.015 | 4.039 | 5 | 9 | 13 | metanephric glomerulus development |
| GO:0090307 | 0.015 | 4.039 | 5 | 9 | 13 | spindle assembly involved in mitosis |
| GO:0042119 | 0.015 | 3.590 | 5 | 10 | 15 | neutrophil activation |
| GO:2000145 | 0.015 | 1.286 | 123 | 142 | 342 | regulation of cell motility |
| GO:2000147 | 0.015 | 1.387 | 71 | 86 | 198 | positive regulation of cell motility |
| GO:0001701 | 0.015 | 1.343 | 87 | 104 | 244 | in utero embryonic development |
| GO:0010563 | 0.015 | 1.392 | 69 | 84 | 193 | negative regulation of phosphorus metabolic process |
| GO:0045936 | 0.015 | 1.392 | 69 | 84 | 193 | negative regulation of phosphate metabolic process |
| GO:0051239 | 0.016 | 1.146 | 471 | 506 | 1315 | regulation of multicellular organismal process |
| GO:0022610 | 0.016 | 1.198 | 244 | 270 | 680 | biological adhesion |
| GO:0006458 | 0.016 | 2.097 | 14 | 21 | 39 | 'de novo' protein folding |
| GO:0015748 | 0.016 | 2.097 | 14 | 21 | 39 | organophosphate ester transport |
| GO:0021766 | 0.016 | 2.097 | 14 | 21 | 39 | hippocampus development |
| GO:0043149 | 0.016 | 2.097 | 14 | 21 | 39 | stress fiber assembly |
| GO:0045055 | 0.016 | 2.097 | 14 | 21 | 39 | regulated secretory pathway |
| GO:0048015 | 0.016 | 1.521 | 42 | 54 | 118 | phosphatidylinositol-mediated signaling |
| GO:0048017 | 0.016 | 1.521 | 42 | 54 | 118 | inositol lipid-mediated signaling |
| GO:0040017 | 0.016 | 1.374 | 74 | 89 | 206 | positive regulation of locomotion |
| GO:0007160 | 0.016 | 1.507 | 44 | 56 | 123 | cell-matrix adhesion |
| GO:0070661 | 0.016 | 1.443 | 55 | 68 | 153 | leukocyte proliferation |
| GO:0030097 | 0.016 | 1.260 | 142 | 162 | 395 | hemopoiesis |
| GO:0046777 | 0.016 | 1.452 | 53 | 66 | 148 | protein autophosphorylation |
| GO:0017014 | 0.016 | Inf | 1 | 4 | 4 | protein nitrosylation |
| GO:0018119 | 0.016 | Inf | 1 | 4 | 4 | peptidyl-cysteine S-nitrosylation |
| GO:0031584 | 0.016 | Inf | 1 | 4 | 4 | activation of phospholipase D activity |
| GO:0033625 | 0.016 | Inf | 1 | 4 | 4 | positive regulation of integrin activation |
| GO:0043415 | 0.016 | Inf | 1 | 4 | 4 | positive regulation of skeletal muscle tissue regeneration |
| GO:0043416 | 0.016 | Inf | 1 | 4 | 4 | regulation of skeletal muscle tissue regeneration |
| GO:0051414 | 0.016 | Inf | 1 | 4 | 4 | response to cortisol stimulus |
| GO:0071071 | 0.016 | Inf | 1 | 4 | 4 | regulation of phospholipid biosynthetic process |
| GO:2000113 | 0.016 | 1.204 | 226 | 251 | 630 | negative regulation of cellular macromolecule biosynthetic process |
| GO:0030593 | 0.017 | 1.962 | 16 | 24 | 46 | neutrophil chemotaxis |
| GO:0032321 | 0.017 | 1.962 | 16 | 24 | 46 | positive regulation of Rho GTPase activity |
| GO:0042981 | 0.017 | 1.171 | 321 | 350 | 895 | regulation of apoptotic process |
| GO:0051173 | 0.017 | 1.172 | 315 | 344 | 879 | positive regulation of nitrogen compound metabolic process |
| GO:0051253 | 0.017 | 1.211 | 208 | 232 | 580 | negative regulation of RNA metabolic process |
| GO:0007098 | 0.017 | 2.395 | 10 | 16 | 28 | centrosome cycle |
| GO:0030837 | 0.017 | 2.395 | 10 | 16 | 28 | negative regulation of actin filament polymerization |
| GO:0071378 | 0.017 | 2.395 | 10 | 16 | 28 | cellular response to growth hormone stimulus |
| GO:0008219 | 0.017 | 1.146 | 450 | 484 | 1257 | cell death |
| GO:0048545 | 0.017 | 1.348 | 81 | 97 | 227 | response to steroid hormone stimulus |
| GO:0042325 | 0.017 | 1.198 | 234 | 259 | 652 | regulation of phosphorylation |
| GO:0045944 | 0.018 | 1.222 | 187 | 210 | 522 | positive regulation of transcription from RNA polymerase II promoter |
| GO:0034341 | 0.018 | 1.609 | 32 | 42 | 89 | response to interferon-gamma |
| GO:0051251 | 0.018 | 1.416 | 59 | 73 | 166 | positive regulation of lymphocyte activation |
| GO:0007568 | 0.018 | 1.431 | 56 | 69 | 156 | aging |
| GO:0016337 | 0.018 | 1.322 | 92 | 109 | 258 | cell-cell adhesion |
| GO:0030335 | 0.018 | 1.379 | 70 | 84 | 194 | positive regulation of cell migration |
| GO:0007155 | 0.018 | 1.193 | 243 | 269 | 679 | cell adhesion |
| GO:0032835 | 0.018 | 1.969 | 16 | 23 | 44 | glomerulus development |
| GO:0022409 | 0.019 | 2.449 | 9 | 15 | 26 | positive regulation of cell-cell adhesion |
| GO:0032233 | 0.019 | 2.449 | 9 | 15 | 26 | positive regulation of actin filament bundle assembly |
| GO:0002697 | 0.019 | 1.405 | 61 | 74 | 169 | regulation of immune effector process |
| GO:0065004 | 0.019 | 1.700 | 25 | 34 | 70 | protein-DNA complex assembly |
| GO:0043627 | 0.019 | 1.527 | 39 | 50 | 109 | response to estrogen stimulus |
| GO:0071363 | 0.019 | 1.268 | 126 | 145 | 352 | cellular response to growth factor stimulus |
| GO:0031175 | 0.019 | 1.218 | 187 | 210 | 523 | neuron projection development |
| GO:0032879 | 0.019 | 1.158 | 362 | 392 | 1010 | regulation of localization |
| GO:0051090 | 0.019 | 1.332 | 85 | 101 | 238 | regulation of sequence-specific DNA binding transcription factor activity |
| GO:0016265 | 0.019 | 1.143 | 451 | 484 | 1259 | death |
| GO:0051084 | 0.019 | 2.134 | 13 | 19 | 35 | 'de novo' posttranslational protein folding |
| GO:0046651 | 0.019 | 1.443 | 52 | 64 | 144 | lymphocyte proliferation |
| GO:0048285 | 0.020 | 1.335 | 83 | 99 | 233 | organelle fission |
| GO:0032319 | 0.020 | 1.743 | 23 | 31 | 63 | regulation of Rho GTPase activity |
| GO:0002685 | 0.020 | 1.661 | 27 | 36 | 75 | regulation of leukocyte migration |
| GO:0060348 | 0.020 | 1.661 | 27 | 36 | 75 | bone development |
| GO:0031349 | 0.020 | 1.384 | 65 | 79 | 182 | positive regulation of defense response |
| GO:0032755 | 0.020 | 2.514 | 9 | 14 | 24 | positive regulation of interleukin-6 production |
| GO:0032496 | 0.020 | 1.401 | 60 | 73 | 167 | response to lipopolysaccharide |
| GO:0001570 | 0.021 | 1.873 | 18 | 25 | 49 | vasculogenesis |
| GO:0048144 | 0.021 | 1.873 | 18 | 25 | 49 | fibroblast proliferation |
| GO:0019219 | 0.021 | 1.117 | 717 | 755 | 2000 | regulation of nucleobase-containing compound metabolic process |
| GO:0043068 | 0.021 | 1.271 | 119 | 137 | 332 | positive regulation of programmed cell death |
| GO:0050790 | 0.021 | 1.152 | 377 | 407 | 1052 | regulation of catalytic activity |
| GO:0010810 | 0.021 | 1.600 | 30 | 40 | 85 | regulation of cell-substrate adhesion |
| GO:0032103 | 0.021 | 1.457 | 47 | 59 | 132 | positive regulation of response to external stimulus |
| GO:0045892 | 0.022 | 1.205 | 200 | 223 | 559 | negative regulation of transcription, DNA-dependent |
| GO:0043393 | 0.022 | 1.657 | 26 | 35 | 73 | regulation of protein binding |
| GO:0051246 | 0.022 | 1.150 | 379 | 409 | 1058 | regulation of protein metabolic process |
| GO:0010564 | 0.022 | 1.279 | 111 | 128 | 309 | regulation of cell cycle process |
| GO:0009126 | 0.022 | 2.594 | 8 | 13 | 22 | purine nucleoside monophosphate metabolic process |
| GO:0009167 | 0.022 | 2.594 | 8 | 13 | 22 | purine ribonucleoside monophosphate metabolic process |
| GO:0010543 | 0.022 | 2.594 | 8 | 13 | 22 | regulation of platelet activation |
| GO:0060397 | 0.022 | 2.594 | 8 | 13 | 22 | JAK-STAT cascade involved in growth hormone signaling pathway |
| GO:1901700 | 0.022 | 1.195 | 220 | 244 | 615 | response to oxygen-containing compound |
| GO:0002252 | 0.022 | 1.263 | 123 | 141 | 343 | immune effector process |
| GO:0045787 | 0.022 | 1.554 | 34 | 44 | 95 | positive regulation of cell cycle |
| GO:0043065 | 0.022 | 1.268 | 118 | 136 | 330 | positive regulation of apoptotic process |
| GO:0040012 | 0.022 | 1.254 | 130 | 149 | 364 | regulation of locomotion |
| GO:0010605 | 0.022 | 1.157 | 339 | 367 | 945 | negative regulation of macromolecule metabolic process |
| GO:0060349 | 0.022 | 1.798 | 19 | 27 | 54 | bone morphogenesis |
| GO:0009790 | 0.023 | 1.190 | 227 | 251 | 634 | embryo development |
| GO:0003206 | 0.023 | 1.624 | 28 | 37 | 78 | cardiac chamber morphogenesis |
| GO:0051928 | 0.023 | 1.876 | 17 | 24 | 47 | positive regulation of calcium ion transport |
| GO:0002009 | 0.023 | 1.296 | 98 | 114 | 273 | morphogenesis of an epithelium |
| GO:0051325 | 0.023 | 1.275 | 112 | 129 | 312 | interphase |
| GO:0036294 | 0.023 | 1.693 | 24 | 32 | 66 | cellular response to decreased oxygen levels |
| GO:0071456 | 0.023 | 1.693 | 24 | 32 | 66 | cellular response to hypoxia |
| GO:0016310 | 0.023 | 1.155 | 344 | 372 | 959 | phosphorylation |
| GO:0009161 | 0.023 | 2.181 | 11 | 17 | 31 | ribonucleoside monophosphate metabolic process |
| GO:0030182 | 0.023 | 1.178 | 255 | 280 | 712 | neuron differentiation |
| GO:0032720 | 0.024 | 2.693 | 7 | 12 | 20 | negative regulation of tumor necrosis factor production |
| GO:0034394 | 0.024 | 2.693 | 7 | 12 | 20 | protein localization to cell surface |
| GO:0046627 | 0.024 | 2.693 | 7 | 12 | 20 | negative regulation of insulin receptor signaling pathway |
| GO:0070482 | 0.024 | 1.354 | 70 | 84 | 196 | response to oxygen levels |
| GO:0031099 | 0.024 | 1.570 | 32 | 41 | 88 | regeneration |
| GO:0048523 | 0.024 | 1.113 | 719 | 756 | 2006 | negative regulation of cellular process |
| GO:0048584 | 0.024 | 1.162 | 305 | 332 | 852 | positive regulation of response to stimulus |
| GO:0051171 | 0.024 | 1.112 | 742 | 780 | 2072 | regulation of nitrogen compound metabolic process |
| GO:0010558 | 0.024 | 1.184 | 236 | 260 | 659 | negative regulation of macromolecule biosynthetic process |
| GO:0033002 | 0.024 | 1.548 | 33 | 43 | 93 | muscle cell proliferation |
| GO:0006897 | 0.024 | 1.292 | 97 | 113 | 271 | endocytosis |
| GO:0014066 | 0.025 | 1.798 | 19 | 26 | 52 | regulation of phosphatidylinositol 3-kinase cascade |
| GO:0001780 | 0.025 | 8.967 | 2 | 5 | 6 | neutrophil homeostasis |
| GO:0002566 | 0.025 | 8.967 | 2 | 5 | 6 | somatic diversification of immune receptors via somatic mutation |
| GO:0006068 | 0.025 | 8.967 | 2 | 5 | 6 | ethanol catabolic process |
| GO:0006189 | 0.025 | 8.967 | 2 | 5 | 6 | 'de novo' IMP biosynthetic process |
| GO:0016446 | 0.025 | 8.967 | 2 | 5 | 6 | somatic hypermutation of immunoglobulin genes |
| GO:0021819 | 0.025 | 8.967 | 2 | 5 | 6 | layer formation in cerebral cortex |
| GO:0042482 | 0.025 | 8.967 | 2 | 5 | 6 | positive regulation of odontogenesis |
| GO:0043587 | 0.025 | 8.967 | 2 | 5 | 6 | tongue morphogenesis |
| GO:0045630 | 0.025 | 8.967 | 2 | 5 | 6 | positive regulation of T-helper 2 cell differentiation |
| GO:0045647 | 0.025 | 8.967 | 2 | 5 | 6 | negative regulation of erythrocyte differentiation |
| GO:0045842 | 0.025 | 8.967 | 2 | 5 | 6 | positive regulation of mitotic metaphase/anaphase transition |
| GO:0070208 | 0.025 | 8.967 | 2 | 5 | 6 | protein heterotrimerization |
| GO:0090195 | 0.025 | 8.967 | 2 | 5 | 6 | chemokine secretion |
| GO:0090196 | 0.025 | 8.967 | 2 | 5 | 6 | regulation of chemokine secretion |
| GO:0002237 | 0.025 | 1.371 | 63 | 76 | 176 | response to molecule of bacterial origin |
| GO:0006887 | 0.025 | 1.346 | 71 | 85 | 199 | exocytosis |
| GO:0016525 | 0.025 | 1.879 | 16 | 23 | 45 | negative regulation of angiogenesis |
| GO:0006691 | 0.025 | 2.821 | 6 | 11 | 18 | leukotriene metabolic process |
| GO:0032633 | 0.025 | 2.821 | 6 | 11 | 18 | interleukin-4 production |
| GO:0032855 | 0.025 | 2.821 | 6 | 11 | 18 | positive regulation of Rac GTPase activity |
| GO:0061437 | 0.025 | 2.821 | 6 | 11 | 18 | renal system vasculature development |
| GO:0061440 | 0.025 | 2.821 | 6 | 11 | 18 | kidney vasculature development |
| GO:0010717 | 0.026 | 2.211 | 10 | 16 | 29 | regulation of epithelial to mesenchymal transition |
| GO:0014909 | 0.026 | 2.211 | 10 | 16 | 29 | smooth muscle cell migration |
| GO:0071495 | 0.026 | 1.194 | 205 | 227 | 572 | cellular response to endogenous stimulus |
| GO:0006974 | 0.026 | 1.229 | 148 | 167 | 413 | response to DNA damage stimulus |
| GO:0006909 | 0.026 | 1.649 | 25 | 33 | 69 | phagocytosis |
| GO:0042592 | 0.027 | 1.155 | 320 | 347 | 894 | homeostatic process |
| GO:0048646 | 0.027 | 1.139 | 406 | 435 | 1132 | anatomical structure formation involved in morphogenesis |
| GO:0019370 | 0.027 | 2.991 | 6 | 10 | 16 | leukotriene biosynthetic process |
| GO:0034308 | 0.027 | 2.991 | 6 | 10 | 16 | primary alcohol metabolic process |
| GO:0061082 | 0.027 | 2.991 | 6 | 10 | 16 | myeloid leukocyte cytokine production |
| GO:0051093 | 0.027 | 1.235 | 139 | 157 | 387 | negative regulation of developmental process |
| GO:0050794 | 0.027 | 1.098 | 1682 | 1723 | 4695 | regulation of cellular process |
| GO:0051091 | 0.027 | 1.423 | 49 | 60 | 136 | positive regulation of sequence-specific DNA binding transcription factor activity |
| GO:0002573 | 0.027 | 1.490 | 38 | 48 | 106 | myeloid leukocyte differentiation |
| GO:0014068 | 0.028 | 1.883 | 15 | 22 | 43 | positive regulation of phosphatidylinositol 3-kinase cascade |
| GO:0030833 | 0.028 | 1.686 | 22 | 30 | 62 | regulation of actin filament polymerization |
| GO:0044236 | 0.028 | 1.686 | 22 | 30 | 62 | multicellular organismal metabolic process |
| GO:0010977 | 0.028 | 2.245 | 10 | 15 | 27 | negative regulation of neuron projection development |
| GO:0014015 | 0.028 | 2.245 | 10 | 15 | 27 | positive regulation of gliogenesis |
| GO:0032508 | 0.028 | 2.245 | 10 | 15 | 27 | DNA duplex unwinding |
| GO:0061371 | 0.028 | 2.245 | 10 | 15 | 27 | determination of heart left/right asymmetry |
| GO:0071277 | 0.028 | 2.245 | 10 | 15 | 27 | cellular response to calcium ion |
| GO:0010224 | 0.028 | 3.230 | 5 | 9 | 14 | response to UV-B |
| GO:0030866 | 0.028 | 3.230 | 5 | 9 | 14 | cortical actin cytoskeleton organization |
| GO:0055003 | 0.028 | 3.230 | 5 | 9 | 14 | cardiac myofibril assembly |
| GO:0071285 | 0.028 | 3.230 | 5 | 9 | 14 | cellular response to lithium ion |
| GO:0071398 | 0.028 | 3.230 | 5 | 9 | 14 | cellular response to fatty acid |
| GO:0033043 | 0.028 | 1.243 | 128 | 145 | 356 | regulation of organelle organization |
| GO:0001833 | 0.029 | 5.381 | 3 | 6 | 8 | inner cell mass cell proliferation |
| GO:0006069 | 0.029 | 5.381 | 3 | 6 | 8 | ethanol oxidation |
| GO:0006336 | 0.029 | 5.381 | 3 | 6 | 8 | DNA replication-independent nucleosome assembly |
| GO:0031055 | 0.029 | 5.381 | 3 | 6 | 8 | chromatin remodeling at centromere |
| GO:0034080 | 0.029 | 5.381 | 3 | 6 | 8 | CENP-A containing nucleosome assembly at centromere |
| GO:0034724 | 0.029 | 5.381 | 3 | 6 | 8 | DNA replication-independent nucleosome organization |
| GO:0042559 | 0.029 | 5.381 | 3 | 6 | 8 | pteridine-containing compound biosynthetic process |
| GO:0043088 | 0.029 | 5.381 | 3 | 6 | 8 | regulation of Cdc42 GTPase activity |
| GO:0043129 | 0.029 | 5.381 | 3 | 6 | 8 | surfactant homeostasis |
| GO:2000352 | 0.029 | 5.381 | 3 | 6 | 8 | negative regulation of endothelial cell apoptotic process |
| GO:0042127 | 0.029 | 1.153 | 318 | 344 | 887 | regulation of cell proliferation |
| GO:0070848 | 0.029 | 1.240 | 130 | 148 | 364 | response to growth factor stimulus |
| GO:0002757 | 0.029 | 1.357 | 63 | 76 | 177 | immune response-activating signal transduction |
| GO:0009148 | 0.029 | 3.589 | 4 | 8 | 12 | pyrimidine nucleoside triphosphate biosynthetic process |
| GO:0033137 | 0.029 | 3.589 | 4 | 8 | 12 | negative regulation of peptidyl-serine phosphorylation |
| GO:0046112 | 0.029 | 3.589 | 4 | 8 | 12 | nucleobase biosynthetic process |
| GO:0046605 | 0.029 | 3.589 | 4 | 8 | 12 | regulation of centrosome cycle |
| GO:1901020 | 0.029 | 3.589 | 4 | 8 | 12 | negative regulation of calcium ion transmembrane transporter activity |
| GO:0051716 | 0.030 | 1.095 | 1210 | 1250 | 3376 | cellular response to stimulus |
| GO:0002283 | 0.030 | 4.186 | 4 | 7 | 10 | neutrophil activation involved in immune response |
| GO:0006188 | 0.030 | 4.186 | 4 | 7 | 10 | IMP biosynthetic process |
| GO:0008608 | 0.030 | 4.186 | 4 | 7 | 10 | attachment of spindle microtubules to kinetochore |
| GO:0035307 | 0.030 | 4.186 | 4 | 7 | 10 | positive regulation of protein dephosphorylation |
| GO:0043586 | 0.030 | 4.186 | 4 | 7 | 10 | tongue development |
| GO:0060766 | 0.030 | 4.186 | 4 | 7 | 10 | negative regulation of androgen receptor signaling pathway |
| GO:0072583 | 0.030 | 4.186 | 4 | 7 | 10 | clathrin-mediated endocytosis |
| GO:0097067 | 0.030 | 4.186 | 4 | 7 | 10 | cellular response to thyroid hormone stimulus |
| GO:0010942 | 0.030 | 1.244 | 124 | 141 | 346 | positive regulation of cell death |
| GO:0051258 | 0.030 | 1.471 | 39 | 49 | 109 | protein polymerization |
| GO:0071453 | 0.030 | 1.609 | 26 | 34 | 72 | cellular response to oxygen levels |
| GO:0043299 | 0.030 | 2.021 | 12 | 18 | 34 | leukocyte degranulation |
| GO:0048844 | 0.030 | 2.021 | 12 | 18 | 34 | artery morphogenesis |
| GO:0050764 | 0.030 | 2.021 | 12 | 18 | 34 | regulation of phagocytosis |
| GO:0051492 | 0.030 | 2.021 | 12 | 18 | 34 | regulation of stress fiber assembly |
| GO:0048145 | 0.030 | 1.797 | 17 | 24 | 48 | regulation of fibroblast proliferation |
| GO:0045935 | 0.030 | 1.153 | 309 | 334 | 861 | positive regulation of nucleobase-containing compound metabolic process |
| GO:0007017 | 0.031 | 1.291 | 89 | 103 | 247 | microtubule-based process |
| GO:0051297 | 0.031 | 1.887 | 15 | 21 | 41 | centrosome organization |
| GO:0050727 | 0.031 | 1.383 | 54 | 66 | 152 | regulation of inflammatory response |
| GO:0034134 | 0.031 | 1.682 | 22 | 29 | 60 | toll-like receptor 2 signaling pathway |
| GO:0002548 | 0.031 | 2.285 | 9 | 14 | 25 | monocyte chemotaxis |
| GO:0014910 | 0.031 | 2.285 | 9 | 14 | 25 | regulation of smooth muscle cell migration |
| GO:0030834 | 0.031 | 2.285 | 9 | 14 | 25 | regulation of actin filament depolymerization |
| GO:0032570 | 0.031 | 2.285 | 9 | 14 | 25 | response to progesterone stimulus |
| GO:0044249 | 0.031 | 1.096 | 1057 | 1096 | 2950 | cellular biosynthetic process |
| GO:0071824 | 0.032 | 1.531 | 31 | 40 | 87 | protein-DNA complex subunit organization |
| GO:0010941 | 0.032 | 1.146 | 331 | 357 | 924 | regulation of cell death |
| GO:0009892 | 0.032 | 1.140 | 364 | 391 | 1016 | negative regulation of metabolic process |
| GO:0033559 | 0.032 | 1.731 | 19 | 26 | 53 | unsaturated fatty acid metabolic process |
| GO:0045639 | 0.032 | 1.731 | 19 | 26 | 53 | positive regulation of myeloid cell differentiation |
| GO:0045637 | 0.033 | 1.479 | 37 | 46 | 102 | regulation of myeloid cell differentiation |
| GO:0009612 | 0.033 | 1.452 | 40 | 50 | 112 | response to mechanical stimulus |
| GO:0071345 | 0.033 | 1.246 | 117 | 133 | 326 | cellular response to cytokine stimulus |
| GO:0032526 | 0.033 | 1.604 | 25 | 33 | 70 | response to retinoic acid |
| GO:0033554 | 0.033 | 1.151 | 304 | 329 | 849 | cellular response to stress |
| GO:0002250 | 0.033 | 1.378 | 54 | 65 | 150 | adaptive immune response |
| GO:0031032 | 0.034 | 2.036 | 11 | 17 | 32 | actomyosin structure organization |
| GO:0048008 | 0.034 | 2.036 | 11 | 17 | 32 | platelet-derived growth factor receptor signaling pathway |
| GO:0051329 | 0.034 | 1.251 | 110 | 126 | 308 | interphase of mitotic cell cycle |
| GO:0032722 | 0.034 | 2.334 | 8 | 13 | 23 | positive regulation of chemokine production |
| GO:1901019 | 0.034 | 2.334 | 8 | 13 | 23 | regulation of calcium ion transmembrane transporter activity |
| GO:0043588 | 0.034 | 1.891 | 14 | 20 | 39 | skin development |
| GO:0022407 | 0.034 | 1.678 | 21 | 28 | 58 | regulation of cell-cell adhesion |
| GO:0031333 | 0.034 | 1.678 | 21 | 28 | 58 | negative regulation of protein complex assembly |
| GO:0051092 | 0.035 | 1.548 | 29 | 37 | 80 | positive regulation of NF-kappaB transcription factor activity |
| GO:0010468 | 0.035 | 1.105 | 695 | 729 | 1940 | regulation of gene expression |
| GO:0008643 | 0.035 | 1.525 | 30 | 39 | 85 | carbohydrate transport |
| GO:0051172 | 0.036 | 1.168 | 233 | 255 | 651 | negative regulation of nitrogen compound metabolic process |
| GO:0009605 | 0.036 | 1.139 | 347 | 373 | 969 | response to external stimulus |
| GO:0002763 | 0.037 | 2.052 | 11 | 16 | 30 | positive regulation of myeloid leukocyte differentiation |
| GO:0006144 | 0.037 | 2.052 | 11 | 16 | 30 | purine nucleobase metabolic process |
| GO:0006271 | 0.037 | 2.052 | 11 | 16 | 30 | DNA strand elongation involved in DNA replication |
| GO:0048146 | 0.037 | 2.052 | 11 | 16 | 30 | positive regulation of fibroblast proliferation |
| GO:0055013 | 0.037 | 2.052 | 11 | 16 | 30 | cardiac muscle cell development |
| GO:0071900 | 0.037 | 1.265 | 96 | 110 | 267 | regulation of protein serine/threonine kinase activity |
| GO:0031023 | 0.037 | 1.797 | 16 | 22 | 44 | microtubule organizing center organization |
| GO:0044092 | 0.037 | 1.191 | 177 | 196 | 494 | negative regulation of molecular function |
| GO:0045123 | 0.037 | 2.393 | 8 | 12 | 21 | cellular extravasation |
| GO:0050853 | 0.037 | 2.393 | 8 | 12 | 21 | B cell receptor signaling pathway |
| GO:0042742 | 0.038 | 1.568 | 26 | 34 | 73 | defense response to bacterium |
| GO:0007264 | 0.038 | 1.235 | 118 | 134 | 330 | small GTPase mediated signal transduction |
| GO:0007569 | 0.038 | 1.674 | 20 | 27 | 56 | cell aging |
| GO:0045621 | 0.038 | 1.674 | 20 | 27 | 56 | positive regulation of lymphocyte differentiation |
| GO:0032272 | 0.038 | 1.896 | 13 | 19 | 37 | negative regulation of protein polymerization |
| GO:2000106 | 0.038 | 1.896 | 13 | 19 | 37 | regulation of leukocyte apoptotic process |
| GO:0051320 | 0.038 | 1.401 | 46 | 56 | 128 | S phase |
| GO:0006959 | 0.039 | 1.519 | 30 | 38 | 83 | humoral immune response |
| GO:0009792 | 0.039 | 1.211 | 143 | 160 | 399 | embryo development ending in birth or egg hatching |
| GO:0048771 | 0.039 | 1.452 | 37 | 46 | 103 | tissue remodeling |
| GO:0050729 | 0.039 | 1.629 | 22 | 29 | 61 | positive regulation of inflammatory response |
| GO:0060333 | 0.039 | 1.629 | 22 | 29 | 61 | interferon-gamma-mediated signaling pathway |
| GO:0031324 | 0.039 | 1.137 | 340 | 365 | 949 | negative regulation of cellular metabolic process |
| GO:0051252 | 0.040 | 1.107 | 602 | 633 | 1680 | regulation of RNA metabolic process |
| GO:0032844 | 0.041 | 1.286 | 79 | 92 | 221 | regulation of homeostatic process |
| GO:0000083 | 0.041 | 2.468 | 7 | 11 | 19 | regulation of transcription involved in G1/S phase of mitotic cell cycle |
| GO:0030042 | 0.041 | 2.072 | 10 | 15 | 28 | actin filament depolymerization |
| GO:0032392 | 0.041 | 2.072 | 10 | 15 | 28 | DNA geometric change |
| GO:0045638 | 0.041 | 1.797 | 15 | 21 | 42 | negative regulation of myeloid cell differentiation |
| GO:0001822 | 0.042 | 1.356 | 53 | 64 | 149 | kidney development |
| GO:0010959 | 0.042 | 1.356 | 53 | 64 | 149 | regulation of metal ion transport |
| GO:0007159 | 0.042 | 1.902 | 13 | 18 | 35 | leukocyte cell-cell adhesion |
| GO:0060350 | 0.042 | 1.902 | 13 | 18 | 35 | endochondral bone morphogenesis |
| GO:0009725 | 0.042 | 1.176 | 194 | 213 | 541 | response to hormone stimulus |
| GO:0043009 | 0.042 | 1.208 | 140 | 157 | 392 | chordate embryonic development |
| GO:0002768 | 0.043 | 1.434 | 38 | 47 | 106 | immune response-regulating cell surface receptor signaling pathway |
| GO:0006469 | 0.043 | 1.434 | 38 | 47 | 106 | negative regulation of protein kinase activity |
| GO:0051260 | 0.043 | 1.368 | 50 | 60 | 139 | protein homooligomerization |
| GO:0030099 | 0.043 | 1.304 | 69 | 81 | 193 | myeloid cell differentiation |
| GO:0048666 | 0.044 | 1.166 | 213 | 233 | 595 | neuron development |
| GO:0009064 | 0.044 | 1.722 | 17 | 23 | 47 | glutamine family amino acid metabolic process |
| GO:0060429 | 0.044 | 1.196 | 153 | 170 | 427 | epithelium development |
| GO:0048659 | 0.045 | 1.586 | 23 | 30 | 64 | smooth muscle cell proliferation |
| GO:0048754 | 0.045 | 1.389 | 44 | 54 | 124 | branching morphogenesis of an epithelial tube |
| GO:0002262 | 0.045 | 2.564 | 6 | 10 | 17 | myeloid cell homeostasis |
| GO:0006297 | 0.045 | 2.564 | 6 | 10 | 17 | nucleotide-excision repair, DNA gap filling |
| GO:0021602 | 0.045 | 2.564 | 6 | 10 | 17 | cranial nerve morphogenesis |
| GO:0042558 | 0.045 | 2.564 | 6 | 10 | 17 | pteridine-containing compound metabolic process |
| GO:0045600 | 0.045 | 2.564 | 6 | 10 | 17 | positive regulation of fat cell differentiation |
| GO:0055012 | 0.045 | 2.564 | 6 | 10 | 17 | ventricular cardiac muscle cell differentiation |
| GO:0072012 | 0.045 | 2.564 | 6 | 10 | 17 | glomerulus vasculature development |
| GO:0090329 | 0.045 | 2.564 | 6 | 10 | 17 | regulation of DNA-dependent DNA replication |
| GO:2000516 | 0.045 | 2.564 | 6 | 10 | 17 | positive regulation of CD4-positive, alpha-beta T cell activation |
| GO:0031347 | 0.045 | 1.222 | 119 | 134 | 332 | regulation of defense response |
| GO:0006418 | 0.046 | 2.094 | 9 | 14 | 26 | tRNA aminoacylation for protein translation |
| GO:0032101 | 0.046 | 1.230 | 111 | 126 | 311 | regulation of response to external stimulus |
| GO:0007585 | 0.046 | 1.796 | 14 | 20 | 40 | respiratory gaseous exchange |
| GO:0000212 | 0.046 | Inf | 1 | 3 | 3 | meiotic spindle organization |
| GO:0001835 | 0.046 | Inf | 1 | 3 | 3 | blastocyst hatching |
| GO:0002513 | 0.046 | Inf | 1 | 3 | 3 | tolerance induction to self antigen |
| GO:0002693 | 0.046 | Inf | 1 | 3 | 3 | positive regulation of cellular extravasation |
| GO:0003104 | 0.046 | Inf | 1 | 3 | 3 | positive regulation of glomerular filtration |
| GO:0003164 | 0.046 | Inf | 1 | 3 | 3 | His-Purkinje system development |
| GO:0003166 | 0.046 | Inf | 1 | 3 | 3 | bundle of His development |
| GO:0006564 | 0.046 | Inf | 1 | 3 | 3 | L-serine biosynthetic process |
| GO:0007509 | 0.046 | Inf | 1 | 3 | 3 | mesoderm migration involved in gastrulation |
| GO:0009304 | 0.046 | Inf | 1 | 3 | 3 | tRNA transcription |
| GO:0010915 | 0.046 | Inf | 1 | 3 | 3 | regulation of very-low-density lipoprotein particle clearance |
| GO:0010916 | 0.046 | Inf | 1 | 3 | 3 | negative regulation of very-low-density lipoprotein particle clearance |
| GO:0014038 | 0.046 | Inf | 1 | 3 | 3 | regulation of Schwann cell differentiation |
| GO:0014707 | 0.046 | Inf | 1 | 3 | 3 | branchiomeric skeletal muscle development |
| GO:0014722 | 0.046 | Inf | 1 | 3 | 3 | regulation of skeletal muscle contraction by calcium ion signaling |
| GO:0015793 | 0.046 | Inf | 1 | 3 | 3 | glycerol transport |
| GO:0015867 | 0.046 | Inf | 1 | 3 | 3 | ATP transport |
| GO:0018242 | 0.046 | Inf | 1 | 3 | 3 | protein O-linked glycosylation via serine |
| GO:0018243 | 0.046 | Inf | 1 | 3 | 3 | protein O-linked glycosylation via threonine |
| GO:0030951 | 0.046 | Inf | 1 | 3 | 3 | establishment or maintenance of microtubule cytoskeleton polarity |
| GO:0032632 | 0.046 | Inf | 1 | 3 | 3 | interleukin-3 production |
| GO:0032713 | 0.046 | Inf | 1 | 3 | 3 | negative regulation of interleukin-4 production |
| GO:0033058 | 0.046 | Inf | 1 | 3 | 3 | directional locomotion |
| GO:0033136 | 0.046 | Inf | 1 | 3 | 3 | serine phosphorylation of STAT3 protein |
| GO:0033561 | 0.046 | Inf | 1 | 3 | 3 | regulation of water loss via skin |
| GO:0034135 | 0.046 | Inf | 1 | 3 | 3 | regulation of toll-like receptor 2 signaling pathway |
| GO:0034205 | 0.046 | Inf | 1 | 3 | 3 | beta-amyloid formation |
| GO:0035188 | 0.046 | Inf | 1 | 3 | 3 | hatching |
| GO:0035459 | 0.046 | Inf | 1 | 3 | 3 | cargo loading into vesicle |
| GO:0035793 | 0.046 | Inf | 1 | 3 | 3 | positive regulation of metanephric mesenchymal cell migration by platelet-derived growth factor receptor-beta signaling pathway |
| GO:0036015 | 0.046 | Inf | 1 | 3 | 3 | response to interleukin-3 |
| GO:0036016 | 0.046 | Inf | 1 | 3 | 3 | cellular response to interleukin-3 |
| GO:0042256 | 0.046 | Inf | 1 | 3 | 3 | mature ribosome assembly |
| GO:0045602 | 0.046 | Inf | 1 | 3 | 3 | negative regulation of endothelial cell differentiation |
| GO:0045626 | 0.046 | Inf | 1 | 3 | 3 | negative regulation of T-helper 1 cell differentiation |
| GO:0046813 | 0.046 | Inf | 1 | 3 | 3 | virion attachment, binding of host cell surface receptor |
| GO:0051503 | 0.046 | Inf | 1 | 3 | 3 | adenine nucleotide transport |
| GO:0060099 | 0.046 | Inf | 1 | 3 | 3 | regulation of phagocytosis, engulfment |
| GO:0060100 | 0.046 | Inf | 1 | 3 | 3 | positive regulation of phagocytosis, engulfment |
| GO:0060398 | 0.046 | Inf | 1 | 3 | 3 | regulation of growth hormone receptor signaling pathway |
| GO:0060912 | 0.046 | Inf | 1 | 3 | 3 | cardiac cell fate specification |
| GO:0061197 | 0.046 | Inf | 1 | 3 | 3 | fungiform papilla morphogenesis |
| GO:0061198 | 0.046 | Inf | 1 | 3 | 3 | fungiform papilla formation |
| GO:0070316 | 0.046 | Inf | 1 | 3 | 3 | regulation of G0 to G1 transition |
| GO:0070318 | 0.046 | Inf | 1 | 3 | 3 | positive regulation of G0 to G1 transition |
| GO:0071072 | 0.046 | Inf | 1 | 3 | 3 | negative regulation of phospholipid biosynthetic process |
| GO:0071684 | 0.046 | Inf | 1 | 3 | 3 | organism emergence from protective structure |
| GO:0071877 | 0.046 | Inf | 1 | 3 | 3 | regulation of adrenergic receptor signaling pathway |
| GO:0072275 | 0.046 | Inf | 1 | 3 | 3 | metanephric glomerulus morphogenesis |
| GO:0072276 | 0.046 | Inf | 1 | 3 | 3 | metanephric glomerulus vasculature morphogenesis |
| GO:0072277 | 0.046 | Inf | 1 | 3 | 3 | metanephric glomerular capillary formation |
| GO:0097242 | 0.046 | Inf | 1 | 3 | 3 | beta-amyloid clearance |
| GO:1900238 | 0.046 | Inf | 1 | 3 | 3 | regulation of metanephric mesenchymal cell migration by platelet-derived growth factor receptor-beta signaling pathway |
| GO:1900739 | 0.046 | Inf | 1 | 3 | 3 | regulation of protein insertion into mitochondrial membrane involved in apoptotic signaling pathway |
| GO:1900740 | 0.046 | Inf | 1 | 3 | 3 | positive regulation of protein insertion into mitochondrial membrane involved in apoptotic signaling pathway |
| GO:1901679 | 0.046 | Inf | 1 | 3 | 3 | nucleotide transmembrane transport |
| GO:2000591 | 0.046 | Inf | 1 | 3 | 3 | positive regulation of metanephric mesenchymal cell migration |
| GO:0048738 | 0.046 | 1.416 | 39 | 48 | 109 | cardiac muscle tissue development |
| GO:0021549 | 0.046 | 1.664 | 19 | 25 | 52 | cerebellum development |
| GO:0002694 | 0.046 | 1.255 | 92 | 105 | 256 | regulation of leukocyte activation |
| GO:0071560 | 0.046 | 1.332 | 57 | 68 | 160 | cellular response to transforming growth factor beta stimulus |
| GO:0050870 | 0.047 | 1.362 | 49 | 59 | 137 | positive regulation of T cell activation |
| GO:0046677 | 0.047 | 1.908 | 12 | 17 | 33 | response to antibiotic |
| GO:0070585 | 0.047 | 1.908 | 12 | 17 | 33 | protein localization to mitochondrion |
| GO:0072655 | 0.047 | 1.908 | 12 | 17 | 33 | establishment of protein localization to mitochondrion |
| GO:0002699 | 0.047 | 1.486 | 30 | 38 | 84 | positive regulation of immune effector process |
| GO:0051130 | 0.047 | 1.207 | 133 | 149 | 372 | positive regulation of cellular component organization |
| GO:0044087 | 0.047 | 1.249 | 95 | 108 | 264 | regulation of cellular component biogenesis |
| GO:0000902 | 0.048 | 1.154 | 236 | 256 | 658 | cell morphogenesis |
| GO:0009607 | 0.048 | 1.191 | 153 | 170 | 428 | response to biotic stimulus |
| GO:0045321 | 0.048 | 1.191 | 153 | 170 | 428 | leukocyte activation |
| GO:0001501 | 0.048 | 1.239 | 100 | 114 | 280 | skeletal system development |
| GO:0009058 | 0.048 | 1.085 | 1095 | 1130 | 3056 | biosynthetic process |
| GO:0031326 | 0.049 | 1.096 | 698 | 729 | 1948 | regulation of cellular biosynthetic process |
| GO:0003179 | 0.049 | 2.691 | 5 | 9 | 15 | heart valve morphogenesis |
| GO:0030865 | 0.049 | 2.691 | 5 | 9 | 15 | cortical cytoskeleton organization |
| GO:0032673 | 0.049 | 2.691 | 5 | 9 | 15 | regulation of interleukin-4 production |
| GO:0033574 | 0.049 | 2.691 | 5 | 9 | 15 | response to testosterone stimulus |
| GO:0034508 | 0.049 | 2.691 | 5 | 9 | 15 | centromere complex assembly |
| GO:0043372 | 0.049 | 2.691 | 5 | 9 | 15 | positive regulation of CD4-positive, alpha-beta T cell differentiation |
| GO:0051181 | 0.049 | 2.691 | 5 | 9 | 15 | cofactor transport |
| GO:0090279 | 0.049 | 2.691 | 5 | 9 | 15 | regulation of calcium ion import |
| GO:0043244 | 0.049 | 1.718 | 16 | 22 | 45 | regulation of protein complex disassembly |
| GO:0048660 | 0.049 | 1.580 | 22 | 29 | 62 | regulation of smooth muscle cell proliferation |
| GO:0006816 | 0.050 | 1.307 | 63 | 74 | 176 | calcium ion transport |
| GO:0042326 | 0.050 | 1.326 | 57 | 67 | 158 | negative regulation of phosphorylation |
