# Supplementary material for: Hidden Treasures in “Ancient” Microarrays: Gene-Expression Portrays Biology and Potential Resistance Pathways of Major Lung Cancer Subtypes and Normal Tissue
Source: Front Oncol. 2014 Sep 29;4:251. doi: 10.3389/fonc.2014.00251 (PMC4178426; doi:10.3389/fonc.2014.00251)
Supplement: Figure S1 — Clinically relevant differentially expressed pathways in each tissue type versus the rest. The pathway maps are arranged according to the scheme in the left-top corner. Red indicates overexpression in genes, dark green downregulation. In the Cell Cycle pathway, tumor suppressor genes were overexpressed in normal lung tissue while oncogenes and tumor driving cyclins were overexpressed in cancers. The gene thymidylate synthase (TYMS or TS, belonging to the pathway “one carbon pool by folate”) is relevant for tumor growth and is also a treatment target. Notably, TYMS was overexpressed only in the tumors that are generally refractory to the drug pemetrexed, as the squamous and the small-cell lung cancer. TYMS was not overexpressed in mesothelioma, but it is known that TYMS expression is highly variable in this cancer. In the ERBB pathway, the ERBB2/HER2 and ERBB3/HER3 were overexpressed in adenocarcinoma, while the ERBB4 was overexpressed in the carcinoids. [file Data_Sheet_1.ZIP › File S3.ZIP!/GO Analysis/Normals vs Rest Enrichment Analysis/Overrepresented GO terms of Down-regulated Genes.html]

Gene to GO BP test for over-representation

| GOBPID | Pvalue | OddsRatio | ExpCount | Count | Size | Term |
| --- | --- | --- | --- | --- | --- | --- |
| GO:0006259 | <0.0001 | 2.233 | 104 | 179 | 522 | DNA metabolic process |
| GO:0022403 | <0.0001 | 2.128 | 112 | 186 | 560 | cell cycle phase |
| GO:0006281 | <0.0001 | 2.776 | 50 | 99 | 248 | DNA repair |
| GO:0000278 | <0.0001 | 2.067 | 106 | 174 | 532 | mitotic cell cycle |
| GO:0022402 | <0.0001 | 1.872 | 140 | 213 | 700 | cell cycle process |
| GO:0044260 | <0.0001 | 1.438 | 748 | 862 | 3749 | cellular macromolecule metabolic process |
| GO:0046483 | <0.0001 | 1.442 | 623 | 735 | 3122 | heterocycle metabolic process |
| GO:0044238 | <0.0001 | 1.471 | 1006 | 1113 | 5040 | primary metabolic process |
| GO:0006139 | <0.0001 | 1.428 | 604 | 712 | 3026 | nucleobase-containing compound metabolic process |
| GO:0006725 | <0.0001 | 1.425 | 626 | 734 | 3135 | cellular aromatic compound metabolic process |
| GO:0006261 | <0.0001 | 4.429 | 15 | 39 | 75 | DNA-dependent DNA replication |
| GO:0071704 | <0.0001 | 1.454 | 1034 | 1135 | 5180 | organic substance metabolic process |
| GO:0090304 | <0.0001 | 1.429 | 497 | 597 | 2488 | nucleic acid metabolic process |
| GO:0044237 | <0.0001 | 1.435 | 993 | 1095 | 4977 | cellular metabolic process |
| GO:0034641 | <0.0001 | 1.405 | 654 | 759 | 3277 | cellular nitrogen compound metabolic process |
| GO:0006807 | <0.0001 | 1.391 | 692 | 795 | 3468 | nitrogen compound metabolic process |
| GO:0006974 | <0.0001 | 1.941 | 82 | 131 | 413 | response to DNA damage stimulus |
| GO:0008152 | <0.0001 | 1.441 | 1086 | 1179 | 5441 | metabolic process |
| GO:0043170 | <0.0001 | 1.382 | 832 | 932 | 4168 | macromolecule metabolic process |
| GO:0000279 | <0.0001 | 2.077 | 63 | 105 | 315 | M phase |
| GO:1901360 | <0.0001 | 1.377 | 654 | 753 | 3278 | organic cyclic compound metabolic process |
| GO:0006310 | <0.0001 | 2.838 | 27 | 56 | 137 | DNA recombination |
| GO:0051325 | <0.0001 | 2.045 | 62 | 103 | 312 | interphase |
| GO:0051329 | <0.0001 | 2.022 | 61 | 101 | 308 | interphase of mitotic cell cycle |
| GO:0007049 | <0.0001 | 1.557 | 184 | 247 | 921 | cell cycle |
| GO:0000087 | <0.0001 | 2.232 | 45 | 79 | 225 | M phase of mitotic cell cycle |
| GO:0071103 | <0.0001 | 2.899 | 21 | 44 | 106 | DNA conformation change |
| GO:0000280 | <0.0001 | 2.143 | 44 | 75 | 219 | nuclear division |
| GO:0007067 | <0.0001 | 2.143 | 44 | 75 | 219 | mitosis |
| GO:0051276 | <0.0001 | 1.755 | 87 | 130 | 438 | chromosome organization |
| GO:0044249 | <0.0001 | 1.329 | 589 | 674 | 2950 | cellular biosynthetic process |
| GO:0048285 | <0.0001 | 2.071 | 47 | 78 | 233 | organelle fission |
| GO:0010564 | <0.0001 | 1.890 | 62 | 97 | 309 | regulation of cell cycle process |
| GO:1901576 | <0.0001 | 1.319 | 602 | 685 | 3014 | organic substance biosynthetic process |
| GO:0009058 | <0.0001 | 1.315 | 610 | 693 | 3056 | biosynthetic process |
| GO:0034728 | <0.0001 | 3.587 | 13 | 30 | 64 | nucleosome organization |
| GO:0051320 | <0.0001 | 2.535 | 26 | 49 | 128 | S phase |
| GO:0006323 | <0.0001 | 3.197 | 15 | 33 | 75 | DNA packaging |
| GO:0006334 | <0.0001 | 4.233 | 9 | 24 | 47 | nucleosome assembly |
| GO:0000084 | <0.0001 | 2.525 | 25 | 47 | 123 | S phase of mitotic cell cycle |
| GO:0006270 | <0.0001 | 7.191 | 5 | 16 | 25 | DNA replication initiation |
| GO:0007059 | <0.0001 | 3.074 | 16 | 34 | 79 | chromosome segregation |
| GO:0009127 | <0.0001 | 10.504 | 4 | 13 | 18 | purine nucleoside monophosphate biosynthetic process |
| GO:0009168 | <0.0001 | 10.504 | 4 | 13 | 18 | purine ribonucleoside monophosphate biosynthetic process |
| GO:0007051 | <0.0001 | 3.654 | 11 | 27 | 57 | spindle organization |
| GO:0006333 | <0.0001 | 3.231 | 14 | 31 | 70 | chromatin assembly or disassembly |
| GO:0034645 | <0.0001 | 1.311 | 469 | 542 | 2348 | cellular macromolecule biosynthetic process |
| GO:0000236 | <0.0001 | 4.054 | 9 | 23 | 46 | mitotic prometaphase |
| GO:0009124 | <0.0001 | 5.732 | 6 | 17 | 29 | nucleoside monophosphate biosynthetic process |
| GO:0006260 | <0.0001 | 2.081 | 37 | 63 | 187 | DNA replication |
| GO:0006284 | <0.0001 | 5.203 | 6 | 18 | 32 | base-excision repair |
| GO:0009123 | <0.0001 | 4.808 | 7 | 19 | 35 | nucleoside monophosphate metabolic process |
| GO:0009156 | <0.0001 | 5.882 | 5 | 16 | 27 | ribonucleoside monophosphate biosynthetic process |
| GO:0071824 | <0.0001 | 2.738 | 17 | 35 | 87 | protein-DNA complex subunit organization |
| GO:0031497 | <0.0001 | 3.496 | 11 | 25 | 54 | chromatin assembly |
| GO:0000075 | <0.0001 | 2.045 | 37 | 61 | 183 | cell cycle checkpoint |
| GO:0071156 | <0.0001 | 1.986 | 40 | 65 | 199 | regulation of cell cycle arrest |
| GO:0000819 | <0.0001 | 4.552 | 7 | 18 | 34 | sister chromatid segregation |
| GO:0006283 | <0.0001 | 3.866 | 9 | 21 | 43 | transcription-coupled nucleotide-excision repair |
| GO:0009059 | <0.0001 | 1.276 | 487 | 554 | 2439 | macromolecule biosynthetic process |
| GO:0051726 | <0.0001 | 1.533 | 106 | 144 | 533 | regulation of cell cycle |
| GO:0000070 | <0.0001 | 4.583 | 6 | 17 | 32 | mitotic sister chromatid segregation |
| GO:0065004 | <0.0001 | 2.871 | 14 | 29 | 70 | protein-DNA complex assembly |
| GO:0006271 | <0.0001 | 4.619 | 6 | 16 | 30 | DNA strand elongation involved in DNA replication |
| GO:0006520 | <0.0001 | 1.708 | 59 | 87 | 296 | cellular amino acid metabolic process |
| GO:0006399 | <0.0001 | 3.212 | 10 | 23 | 52 | tRNA metabolic process |
| GO:0009126 | <0.0001 | 5.832 | 4 | 13 | 22 | purine nucleoside monophosphate metabolic process |
| GO:0009167 | <0.0001 | 5.832 | 4 | 13 | 22 | purine ribonucleoside monophosphate metabolic process |
| GO:0034660 | <0.0001 | 2.130 | 26 | 45 | 131 | ncRNA metabolic process |
| GO:0009161 | <0.0001 | 4.311 | 6 | 16 | 31 | ribonucleoside monophosphate metabolic process |
| GO:0000082 | <0.0001 | 1.981 | 32 | 52 | 159 | G1/S transition of mitotic cell cycle |
| GO:0010467 | <0.0001 | 1.252 | 498 | 561 | 2497 | gene expression |
| GO:0007050 | <0.0001 | 1.693 | 55 | 81 | 277 | cell cycle arrest |
| GO:0022616 | <0.0001 | 4.041 | 6 | 16 | 32 | DNA strand elongation |
| GO:0007052 | <0.0001 | 4.039 | 6 | 15 | 30 | mitotic spindle organization |
| GO:0046040 | <0.0001 | 10.742 | 2 | 8 | 11 | IMP metabolic process |
| GO:0016070 | <0.0001 | 1.236 | 440 | 495 | 2204 | RNA metabolic process |
| GO:0018130 | <0.0001 | 1.238 | 424 | 478 | 2123 | heterocycle biosynthetic process |
| GO:0044271 | <0.0001 | 1.232 | 433 | 487 | 2170 | cellular nitrogen compound biosynthetic process |
| GO:0006839 | <0.0001 | 2.363 | 15 | 28 | 76 | mitochondrial transport |
| GO:0006297 | <0.0001 | 5.758 | 3 | 10 | 17 | nucleotide-excision repair, DNA gap filling |
| GO:0006541 | <0.0001 | 5.758 | 3 | 10 | 17 | glutamine metabolic process |
| GO:0033554 | <0.0001 | 1.337 | 169 | 207 | 849 | cellular response to stress |
| GO:0007017 | <0.0001 | 1.647 | 49 | 71 | 247 | microtubule-based process |
| GO:0051321 | 0.001 | 2.159 | 19 | 33 | 95 | meiotic cell cycle |
| GO:0009262 | 0.001 | 4.928 | 4 | 11 | 20 | deoxyribonucleotide metabolic process |
| GO:0019438 | 0.001 | 1.228 | 424 | 476 | 2123 | aromatic compound biosynthetic process |
| GO:0046112 | 0.001 | 8.055 | 2 | 8 | 12 | nucleobase biosynthetic process |
| GO:0006418 | 0.001 | 4.035 | 5 | 13 | 26 | tRNA aminoacylation for protein translation |
| GO:0006289 | 0.001 | 2.490 | 13 | 24 | 63 | nucleotide-excision repair |
| GO:0006312 | 0.001 | 3.364 | 7 | 15 | 33 | mitotic recombination |
| GO:0034654 | 0.001 | 1.221 | 416 | 466 | 2083 | nucleobase-containing compound biosynthetic process |
| GO:0006338 | 0.001 | 2.428 | 13 | 24 | 64 | chromatin remodeling |
| GO:0006188 | 0.001 | 9.393 | 2 | 7 | 10 | IMP biosynthetic process |
| GO:0008608 | 0.001 | 9.393 | 2 | 7 | 10 | attachment of spindle microtubules to kinetochore |
| GO:0006144 | 0.001 | 3.531 | 6 | 14 | 30 | purine nucleobase metabolic process |
| GO:0032508 | 0.001 | 3.746 | 5 | 13 | 27 | DNA duplex unwinding |
| GO:0042254 | 0.001 | 2.223 | 16 | 28 | 79 | ribosome biogenesis |
| GO:0000722 | 0.001 | 4.033 | 5 | 12 | 24 | telomere maintenance via recombination |
| GO:0032201 | 0.001 | 4.033 | 5 | 12 | 24 | telomere maintenance via semi-conservative replication |
| GO:0007126 | 0.001 | 2.093 | 18 | 31 | 91 | meiosis |
| GO:0051327 | 0.001 | 2.093 | 18 | 31 | 91 | M phase of meiotic cell cycle |
| GO:1901293 | 0.001 | 1.640 | 43 | 62 | 216 | nucleoside phosphate biosynthetic process |
| GO:0042559 | 0.001 | 12.071 | 2 | 6 | 8 | pteridine-containing compound biosynthetic process |
| GO:0034622 | 0.001 | 1.487 | 69 | 92 | 345 | cellular macromolecular complex assembly |
| GO:0007091 | 0.001 | 3.323 | 6 | 14 | 31 | mitotic metaphase/anaphase transition |
| GO:0032392 | 0.001 | 3.496 | 6 | 13 | 28 | DNA geometric change |
| GO:0043038 | 0.001 | 3.496 | 6 | 13 | 28 | amino acid activation |
| GO:0043039 | 0.001 | 3.496 | 6 | 13 | 28 | tRNA aminoacylation |
| GO:0051983 | 0.001 | 5.179 | 3 | 9 | 16 | regulation of chromosome segregation |
| GO:0000216 | 0.001 | 2.247 | 14 | 25 | 70 | M/G1 transition of mitotic cell cycle |
| GO:0000083 | 0.002 | 4.477 | 4 | 10 | 19 | regulation of transcription involved in G1/S phase of mitotic cell cycle |
| GO:1901362 | 0.002 | 1.203 | 440 | 488 | 2205 | organic cyclic compound biosynthetic process |
| GO:0002566 | 0.002 | 20.109 | 1 | 5 | 6 | somatic diversification of immune receptors via somatic mutation |
| GO:0006189 | 0.002 | 20.109 | 1 | 5 | 6 | 'de novo' IMP biosynthetic process |
| GO:0016446 | 0.002 | 20.109 | 1 | 5 | 6 | somatic hypermutation of immunoglobulin genes |
| GO:0045786 | 0.002 | 1.485 | 66 | 88 | 330 | negative regulation of cell cycle |
| GO:0009165 | 0.002 | 1.612 | 43 | 61 | 215 | nucleotide biosynthetic process |
| GO:0022613 | 0.002 | 1.808 | 27 | 41 | 133 | ribonucleoprotein complex biogenesis |
| GO:0000226 | 0.002 | 1.685 | 35 | 51 | 174 | microtubule cytoskeleton organization |
| GO:0006325 | 0.002 | 1.468 | 65 | 86 | 325 | chromatin organization |
| GO:0009394 | 0.003 | 4.531 | 3 | 9 | 17 | 2'-deoxyribonucleotide metabolic process |
| GO:0019692 | 0.003 | 4.531 | 3 | 9 | 17 | deoxyribose phosphate metabolic process |
| GO:0008652 | 0.003 | 2.214 | 13 | 23 | 65 | cellular amino acid biosynthetic process |
| GO:0006396 | 0.003 | 1.425 | 75 | 97 | 375 | RNA processing |
| GO:0070585 | 0.003 | 2.972 | 7 | 14 | 33 | protein localization to mitochondrion |
| GO:0072655 | 0.003 | 2.972 | 7 | 14 | 33 | establishment of protein localization to mitochondrion |
| GO:0006996 | 0.003 | 1.226 | 275 | 313 | 1376 | organelle organization |
| GO:0042398 | 0.003 | 3.083 | 6 | 13 | 30 | cellular modified amino acid biosynthetic process |
| GO:0009113 | 0.003 | 8.046 | 2 | 6 | 9 | purine nucleobase biosynthetic process |
| GO:0033145 | 0.003 | 8.046 | 2 | 6 | 9 | positive regulation of intracellular steroid hormone receptor signaling pathway |
| GO:0043687 | 0.003 | 1.836 | 22 | 34 | 109 | post-translational protein modification |
| GO:0009112 | 0.004 | 2.422 | 10 | 18 | 48 | nucleobase metabolic process |
| GO:0046605 | 0.004 | 5.634 | 2 | 7 | 12 | regulation of centrosome cycle |
| GO:0033146 | 0.004 | 4.601 | 3 | 8 | 15 | regulation of intracellular estrogen receptor signaling pathway |
| GO:0034508 | 0.004 | 4.601 | 3 | 8 | 15 | centromere complex assembly |
| GO:0051301 | 0.004 | 1.449 | 60 | 79 | 301 | cell division |
| GO:0065003 | 0.004 | 1.294 | 135 | 162 | 676 | macromolecular complex assembly |
| GO:0006412 | 0.004 | 1.471 | 55 | 73 | 275 | translation |
| GO:0006353 | 0.005 | 2.175 | 12 | 21 | 60 | DNA-dependent transcription, termination |
| GO:0006268 | 0.005 | 10.053 | 1 | 5 | 7 | DNA unwinding involved in replication |
| GO:0009263 | 0.005 | 10.053 | 1 | 5 | 7 | deoxyribonucleotide biosynthetic process |
| GO:0051488 | 0.005 | 10.053 | 1 | 5 | 7 | activation of anaphase-promoting complex activity |
| GO:0090344 | 0.005 | 10.053 | 1 | 5 | 7 | negative regulation of cell aging |
| GO:1901605 | 0.005 | 1.756 | 24 | 36 | 119 | alpha-amino acid metabolic process |
| GO:0000723 | 0.005 | 2.344 | 10 | 18 | 49 | telomere maintenance |
| GO:0032200 | 0.005 | 2.344 | 10 | 18 | 49 | telomere organization |
| GO:0007098 | 0.005 | 3.023 | 6 | 12 | 28 | centrosome cycle |
| GO:0009108 | 0.005 | 2.182 | 11 | 20 | 57 | coenzyme biosynthetic process |
| GO:0000377 | 0.006 | 1.644 | 30 | 43 | 149 | RNA splicing, via transesterification reactions with bulged adenosine as nucleophile |
| GO:0000398 | 0.006 | 1.644 | 30 | 43 | 149 | mRNA splicing, via spliceosome |
| GO:0090305 | 0.006 | 2.365 | 9 | 17 | 46 | nucleic acid phosphodiester bond hydrolysis |
| GO:0043588 | 0.006 | 2.521 | 8 | 15 | 39 | skin development |
| GO:0010833 | 0.006 | 2.758 | 6 | 13 | 32 | telomere maintenance via telomere lengthening |
| GO:0016266 | 0.006 | 3.356 | 4 | 10 | 22 | O-glycan processing |
| GO:0000375 | 0.006 | 1.621 | 31 | 44 | 154 | RNA splicing, via transesterification reactions |
| GO:0006167 | 0.007 | 16.077 | 1 | 4 | 5 | AMP biosynthetic process |
| GO:0042074 | 0.007 | 16.077 | 1 | 4 | 5 | cell migration involved in gastrulation |
| GO:0046826 | 0.007 | 16.077 | 1 | 4 | 5 | negative regulation of protein export from nucleus |
| GO:0022037 | 0.007 | 2.125 | 12 | 20 | 58 | metencephalon development |
| GO:0006626 | 0.007 | 2.844 | 6 | 12 | 29 | protein targeting to mitochondrion |
| GO:0006298 | 0.007 | 4.025 | 3 | 8 | 16 | mismatch repair |
| GO:0006488 | 0.007 | 4.025 | 3 | 8 | 16 | dolichol-linked oligosaccharide biosynthetic process |
| GO:0009064 | 0.007 | 2.286 | 9 | 17 | 47 | glutamine family amino acid metabolic process |
| GO:0031145 | 0.007 | 1.976 | 14 | 23 | 70 | anaphase-promoting complex-dependent proteasomal ubiquitin-dependent protein catabolic process |
| GO:0045333 | 0.008 | 1.734 | 22 | 33 | 110 | cellular respiration |
| GO:0051188 | 0.008 | 1.906 | 16 | 25 | 78 | cofactor biosynthetic process |
| GO:0030520 | 0.008 | 2.954 | 5 | 11 | 26 | intracellular estrogen receptor signaling pathway |
| GO:0006564 | 0.008 | Inf | 1 | 3 | 3 | L-serine biosynthetic process |
| GO:0007509 | 0.008 | Inf | 1 | 3 | 3 | mesoderm migration involved in gastrulation |
| GO:0009304 | 0.008 | Inf | 1 | 3 | 3 | tRNA transcription |
| GO:0018242 | 0.008 | Inf | 1 | 3 | 3 | protein O-linked glycosylation via serine |
| GO:0018243 | 0.008 | Inf | 1 | 3 | 3 | protein O-linked glycosylation via threonine |
| GO:0033136 | 0.008 | Inf | 1 | 3 | 3 | serine phosphorylation of STAT3 protein |
| GO:1901679 | 0.008 | Inf | 1 | 3 | 3 | nucleotide transmembrane transport |
| GO:0006200 | 0.008 | 2.070 | 12 | 20 | 59 | ATP catabolic process |
| GO:0006575 | 0.009 | 1.711 | 22 | 33 | 111 | cellular modified amino acid metabolic process |
| GO:0050434 | 0.009 | 2.212 | 10 | 17 | 48 | positive regulation of viral transcription |
| GO:0006366 | 0.009 | 1.215 | 207 | 236 | 1036 | transcription from RNA polymerase II promoter |
| GO:0043902 | 0.009 | 1.870 | 16 | 25 | 79 | positive regulation of multi-organism process |
| GO:0006493 | 0.009 | 2.686 | 6 | 12 | 30 | protein O-linked glycosylation |
| GO:0006351 | 0.009 | 1.170 | 359 | 395 | 1800 | transcription, DNA-dependent |
| GO:0021549 | 0.009 | 2.136 | 10 | 18 | 52 | cerebellum development |
| GO:0000080 | 0.010 | 2.326 | 8 | 15 | 41 | G1 phase of mitotic cell cycle |
| GO:0051297 | 0.010 | 2.326 | 8 | 15 | 41 | centrosome organization |
| GO:0000086 | 0.010 | 1.689 | 22 | 33 | 112 | G2/M transition of mitotic cell cycle |
| GO:0000079 | 0.010 | 2.018 | 12 | 20 | 60 | regulation of cyclin-dependent protein kinase activity |
| GO:0006336 | 0.010 | 6.701 | 2 | 5 | 8 | DNA replication-independent nucleosome assembly |
| GO:0009070 | 0.010 | 6.701 | 2 | 5 | 8 | serine family amino acid biosynthetic process |
| GO:0009219 | 0.010 | 6.701 | 2 | 5 | 8 | pyrimidine deoxyribonucleotide metabolic process |
| GO:0009886 | 0.010 | 6.701 | 2 | 5 | 8 | post-embryonic morphogenesis |
| GO:0031055 | 0.010 | 6.701 | 2 | 5 | 8 | chromatin remodeling at centromere |
| GO:0033148 | 0.010 | 6.701 | 2 | 5 | 8 | positive regulation of intracellular estrogen receptor signaling pathway |
| GO:0034080 | 0.010 | 6.701 | 2 | 5 | 8 | CENP-A containing nucleosome assembly at centromere |
| GO:0034724 | 0.010 | 6.701 | 2 | 5 | 8 | DNA replication-independent nucleosome organization |
| GO:0046033 | 0.010 | 6.701 | 2 | 5 | 8 | AMP metabolic process |
| GO:0008380 | 0.010 | 1.475 | 42 | 56 | 210 | RNA splicing |
| GO:0000724 | 0.011 | 2.495 | 7 | 13 | 34 | double-strand break repair via homologous recombination |
| GO:0000725 | 0.011 | 2.495 | 7 | 13 | 34 | recombinational repair |
| GO:0010977 | 0.011 | 2.769 | 5 | 11 | 27 | negative regulation of neuron projection development |
| GO:0030071 | 0.011 | 2.769 | 5 | 11 | 27 | regulation of mitotic metaphase/anaphase transition |
| GO:0042558 | 0.011 | 3.577 | 3 | 8 | 17 | pteridine-containing compound metabolic process |
| GO:0000085 | 0.011 | 4.826 | 2 | 6 | 11 | G2 phase of mitotic cell cycle |
| GO:0050000 | 0.011 | 4.826 | 2 | 6 | 11 | chromosome localization |
| GO:0051303 | 0.011 | 4.826 | 2 | 6 | 11 | establishment of chromosome localization |
| GO:0051319 | 0.011 | 4.826 | 2 | 6 | 11 | G2 phase |
| GO:0031100 | 0.012 | 2.351 | 8 | 14 | 38 | organ regeneration |
| GO:0051252 | 0.012 | 1.168 | 335 | 369 | 1680 | regulation of RNA metabolic process |
| GO:0051318 | 0.013 | 2.239 | 8 | 15 | 42 | G1 phase |
| GO:0007005 | 0.013 | 1.572 | 29 | 40 | 143 | mitochondrion organization |
| GO:0006302 | 0.013 | 1.857 | 15 | 23 | 73 | double-strand break repair |
| GO:0006369 | 0.014 | 2.381 | 7 | 13 | 35 | termination of RNA polymerase II transcription |
| GO:0051084 | 0.014 | 2.381 | 7 | 13 | 35 | 'de novo' posttranslational protein folding |
| GO:0044724 | 0.014 | 1.684 | 20 | 30 | 102 | single-organism carbohydrate catabolic process |
| GO:2000112 | 0.014 | 1.159 | 357 | 390 | 1787 | regulation of cellular macromolecule biosynthetic process |
| GO:0008033 | 0.014 | 2.606 | 6 | 11 | 28 | tRNA processing |
| GO:0006458 | 0.015 | 2.257 | 8 | 14 | 39 | 'de novo' protein folding |
| GO:0021766 | 0.015 | 2.257 | 8 | 14 | 39 | hippocampus development |
| GO:0007088 | 0.015 | 1.883 | 13 | 21 | 66 | regulation of mitosis |
| GO:0051783 | 0.015 | 1.883 | 13 | 21 | 66 | regulation of nuclear division |
| GO:0032774 | 0.015 | 1.154 | 372 | 405 | 1862 | RNA biosynthetic process |
| GO:0019219 | 0.016 | 1.149 | 399 | 433 | 2000 | regulation of nucleobase-containing compound metabolic process |
| GO:0003222 | 0.017 | 8.037 | 1 | 4 | 6 | ventricular trabecula myocardium morphogenesis |
| GO:0006563 | 0.017 | 8.037 | 1 | 4 | 6 | L-serine metabolic process |
| GO:0007080 | 0.017 | 8.037 | 1 | 4 | 6 | mitotic metaphase plate congression |
| GO:0019673 | 0.017 | 8.037 | 1 | 4 | 6 | GDP-mannose metabolic process |
| GO:0021819 | 0.017 | 8.037 | 1 | 4 | 6 | layer formation in cerebral cortex |
| GO:0045842 | 0.017 | 8.037 | 1 | 4 | 6 | positive regulation of mitotic metaphase/anaphase transition |
| GO:0007346 | 0.017 | 1.406 | 47 | 60 | 233 | regulation of mitotic cell cycle |
| GO:0009163 | 0.018 | 1.740 | 17 | 25 | 83 | nucleoside biosynthetic process |
| GO:1901659 | 0.018 | 1.740 | 17 | 25 | 83 | glycosyl compound biosynthetic process |
| GO:0033143 | 0.018 | 2.277 | 7 | 13 | 36 | regulation of intracellular steroid hormone receptor signaling pathway |
| GO:0000726 | 0.018 | 3.520 | 3 | 7 | 15 | non-recombinational repair |
| GO:0033574 | 0.018 | 3.520 | 3 | 7 | 15 | response to testosterone stimulus |
| GO:0006486 | 0.018 | 1.526 | 29 | 40 | 146 | protein glycosylation |
| GO:0043413 | 0.018 | 1.526 | 29 | 40 | 146 | macromolecule glycosylation |
| GO:0042455 | 0.018 | 1.785 | 15 | 23 | 75 | ribonucleoside biosynthetic process |
| GO:0048524 | 0.018 | 1.785 | 15 | 23 | 75 | positive regulation of viral reproduction |
| GO:0010948 | 0.018 | 1.842 | 13 | 21 | 67 | negative regulation of cell cycle process |
| GO:0016052 | 0.018 | 1.638 | 21 | 30 | 104 | carbohydrate catabolic process |
| GO:0009148 | 0.019 | 4.021 | 2 | 6 | 12 | pyrimidine nucleoside triphosphate biosynthetic process |
| GO:0009226 | 0.019 | 4.021 | 2 | 6 | 12 | nucleotide-sugar biosynthetic process |
| GO:0034502 | 0.019 | 4.021 | 2 | 6 | 12 | protein localization to chromosome |
| GO:0009452 | 0.019 | 2.461 | 6 | 11 | 29 | 7-methylguanosine RNA capping |
| GO:0036260 | 0.019 | 2.461 | 6 | 11 | 29 | RNA capping |
| GO:0009264 | 0.019 | 5.025 | 2 | 5 | 9 | deoxyribonucleotide catabolic process |
| GO:0021783 | 0.019 | 5.025 | 2 | 5 | 9 | preganglionic parasympathetic nervous system development |
| GO:0035964 | 0.019 | 5.025 | 2 | 5 | 9 | COPI-coated vesicle budding |
| GO:0042693 | 0.019 | 5.025 | 2 | 5 | 9 | muscle cell fate commitment |
| GO:0043173 | 0.019 | 5.025 | 2 | 5 | 9 | nucleotide salvage |
| GO:0046386 | 0.019 | 5.025 | 2 | 5 | 9 | deoxyribose phosphate catabolic process |
| GO:0046653 | 0.019 | 5.025 | 2 | 5 | 9 | tetrahydrofolate metabolic process |
| GO:0048200 | 0.019 | 5.025 | 2 | 5 | 9 | Golgi transport vesicle coating |
| GO:0048205 | 0.019 | 5.025 | 2 | 5 | 9 | COPI coating of Golgi vesicle |
| GO:0051310 | 0.019 | 5.025 | 2 | 5 | 9 | metaphase plate congression |
| GO:0031023 | 0.020 | 2.084 | 9 | 15 | 44 | microtubule organizing center organization |
| GO:0009225 | 0.020 | 2.786 | 4 | 9 | 22 | nucleotide-sugar metabolic process |
| GO:0070085 | 0.020 | 1.512 | 29 | 40 | 147 | glycosylation |
| GO:0009987 | 0.020 | 1.263 | 1446 | 1466 | 7246 | cellular process |
| GO:0006354 | 0.020 | 1.710 | 17 | 25 | 84 | DNA-dependent transcription, elongation |
| GO:0051340 | 0.021 | 1.730 | 16 | 24 | 80 | regulation of ligase activity |
| GO:0051171 | 0.021 | 1.140 | 414 | 446 | 2072 | regulation of nitrogen compound metabolic process |
| GO:0006308 | 0.021 | 1.910 | 11 | 18 | 56 | DNA catabolic process |
| GO:0060255 | 0.021 | 1.131 | 504 | 538 | 2524 | regulation of macromolecule metabolic process |
| GO:0010556 | 0.021 | 1.145 | 369 | 400 | 1848 | regulation of macromolecule biosynthetic process |
| GO:0046034 | 0.022 | 1.638 | 19 | 28 | 97 | ATP metabolic process |
| GO:0006220 | 0.023 | 2.516 | 5 | 10 | 26 | pyrimidine nucleotide metabolic process |
| GO:0030574 | 0.023 | 2.926 | 4 | 8 | 19 | collagen catabolic process |
| GO:0043616 | 0.023 | 2.926 | 4 | 8 | 19 | keratinocyte proliferation |
| GO:2001141 | 0.023 | 1.149 | 328 | 357 | 1641 | regulation of RNA biosynthetic process |
| GO:0031323 | 0.023 | 1.127 | 529 | 563 | 2650 | regulation of cellular metabolic process |
| GO:0044085 | 0.023 | 1.176 | 216 | 241 | 1082 | cellular component biogenesis |
| GO:0006753 | 0.024 | 1.234 | 116 | 135 | 581 | nucleoside phosphate metabolic process |
| GO:0010468 | 0.024 | 1.138 | 387 | 418 | 1940 | regulation of gene expression |
| GO:0070507 | 0.025 | 1.954 | 10 | 16 | 49 | regulation of microtubule cytoskeleton organization |
| GO:0046782 | 0.025 | 1.824 | 12 | 19 | 61 | regulation of viral transcription |
| GO:0019318 | 0.025 | 1.415 | 39 | 50 | 193 | hexose metabolic process |
| GO:0006362 | 0.026 | 3.128 | 3 | 7 | 16 | transcription elongation from RNA polymerase I promoter |
| GO:0030878 | 0.026 | 3.128 | 3 | 7 | 16 | thyroid gland development |
| GO:1901607 | 0.027 | 2.196 | 7 | 12 | 34 | alpha-amino acid biosynthetic process |
| GO:0071174 | 0.027 | 2.587 | 5 | 9 | 23 | mitotic cell cycle spindle checkpoint |
| GO:0000466 | 0.027 | 12.050 | 1 | 3 | 4 | maturation of 5.8S rRNA from tricistronic rRNA transcript (SSU-rRNA, 5.8S rRNA, LSU-rRNA) |
| GO:0006166 | 0.027 | 12.050 | 1 | 3 | 4 | purine ribonucleoside salvage |
| GO:0009125 | 0.027 | 12.050 | 1 | 3 | 4 | nucleoside monophosphate catabolic process |
| GO:0009202 | 0.027 | 12.050 | 1 | 3 | 4 | deoxyribonucleoside triphosphate biosynthetic process |
| GO:0009223 | 0.027 | 12.050 | 1 | 3 | 4 | pyrimidine deoxyribonucleotide catabolic process |
| GO:0009396 | 0.027 | 12.050 | 1 | 3 | 4 | folic acid-containing compound biosynthetic process |
| GO:0032875 | 0.027 | 12.050 | 1 | 3 | 4 | regulation of DNA endoreduplication |
| GO:0032876 | 0.027 | 12.050 | 1 | 3 | 4 | negative regulation of DNA endoreduplication |
| GO:0034501 | 0.027 | 12.050 | 1 | 3 | 4 | protein localization to kinetochore |
| GO:0034720 | 0.027 | 12.050 | 1 | 3 | 4 | histone H3-K4 demethylation |
| GO:0042023 | 0.027 | 12.050 | 1 | 3 | 4 | DNA endoreduplication |
| GO:0045008 | 0.027 | 12.050 | 1 | 3 | 4 | depyrimidination |
| GO:0051182 | 0.027 | 12.050 | 1 | 3 | 4 | coenzyme transport |
| GO:0060023 | 0.027 | 12.050 | 1 | 3 | 4 | soft palate development |
| GO:0071168 | 0.027 | 12.050 | 1 | 3 | 4 | protein localization to chromatin |
| GO:0006006 | 0.027 | 1.449 | 33 | 43 | 163 | glucose metabolic process |
| GO:0043933 | 0.028 | 1.187 | 173 | 195 | 867 | macromolecular complex subunit organization |
| GO:0046148 | 0.028 | 2.094 | 8 | 13 | 38 | pigment biosynthetic process |
| GO:1901566 | 0.028 | 1.261 | 87 | 103 | 435 | organonitrogen compound biosynthetic process |
| GO:0043467 | 0.029 | 2.014 | 8 | 14 | 42 | regulation of generation of precursor metabolites and energy |
| GO:0019320 | 0.029 | 1.728 | 14 | 21 | 70 | hexose catabolic process |
| GO:1901137 | 0.029 | 1.260 | 86 | 102 | 431 | carbohydrate derivative biosynthetic process |
| GO:0006590 | 0.030 | 3.446 | 3 | 6 | 13 | thyroid hormone generation |
| GO:0009147 | 0.030 | 3.446 | 3 | 6 | 13 | pyrimidine nucleoside triphosphate metabolic process |
| GO:0015949 | 0.030 | 3.446 | 3 | 6 | 13 | nucleobase-containing small molecule interconversion |
| GO:0090307 | 0.030 | 3.446 | 3 | 6 | 13 | spindle assembly involved in mitosis |
| GO:0006360 | 0.030 | 2.367 | 5 | 10 | 27 | transcription from RNA polymerase I promoter |
| GO:0007131 | 0.030 | 2.367 | 5 | 10 | 27 | reciprocal meiotic recombination |
| GO:0035825 | 0.030 | 2.367 | 5 | 10 | 27 | reciprocal DNA recombination |
| GO:0032069 | 0.030 | 1.814 | 12 | 18 | 58 | regulation of nuclease activity |
| GO:0000387 | 0.032 | 2.682 | 4 | 8 | 20 | spliceosomal snRNP assembly |
| GO:0007062 | 0.032 | 2.682 | 4 | 8 | 20 | sister chromatid cohesion |
| GO:0009069 | 0.032 | 2.682 | 4 | 8 | 20 | serine family amino acid metabolic process |
| GO:0010822 | 0.032 | 2.682 | 4 | 8 | 20 | positive regulation of mitochondrion organization |
| GO:0034453 | 0.032 | 2.682 | 4 | 8 | 20 | microtubule anchoring |
| GO:0009117 | 0.032 | 1.220 | 115 | 133 | 577 | nucleotide metabolic process |
| GO:0009200 | 0.032 | 4.019 | 2 | 5 | 10 | deoxyribonucleoside triphosphate metabolic process |
| GO:0043586 | 0.032 | 4.019 | 2 | 5 | 10 | tongue development |
| GO:0043923 | 0.032 | 4.019 | 2 | 5 | 10 | positive regulation by host of viral transcription |
| GO:0045815 | 0.032 | 4.019 | 2 | 5 | 10 | positive regulation of gene expression, epigenetic |
| GO:0048194 | 0.032 | 4.019 | 2 | 5 | 10 | Golgi vesicle budding |
| GO:0048486 | 0.032 | 4.019 | 2 | 5 | 10 | parasympathetic nervous system development |
| GO:0071364 | 0.032 | 4.019 | 2 | 5 | 10 | cellular response to epidermal growth factor stimulus |
| GO:0072583 | 0.032 | 4.019 | 2 | 5 | 10 | clathrin-mediated endocytosis |
| GO:0071840 | 0.033 | 1.117 | 542 | 574 | 2717 | cellular component organization or biogenesis |
| GO:0006285 | 0.033 | 5.357 | 1 | 4 | 7 | base-excision repair, AP site formation |
| GO:0022038 | 0.033 | 5.357 | 1 | 4 | 7 | corpus callosum development |
| GO:0032261 | 0.033 | 5.357 | 1 | 4 | 7 | purine nucleotide salvage |
| GO:0043968 | 0.033 | 5.357 | 1 | 4 | 7 | histone H2A acetylation |
| GO:0050687 | 0.033 | 5.357 | 1 | 4 | 7 | negative regulation of defense response to virus |
| GO:0034470 | 0.034 | 1.589 | 18 | 26 | 92 | ncRNA processing |
| GO:2000243 | 0.034 | 1.589 | 18 | 26 | 92 | positive regulation of reproductive process |
| GO:0051289 | 0.035 | 2.013 | 8 | 13 | 39 | protein homotetramerization |
| GO:0051402 | 0.035 | 1.469 | 27 | 36 | 135 | neuron apoptotic process |
| GO:0010907 | 0.036 | 2.414 | 5 | 9 | 24 | positive regulation of glucose metabolic process |
| GO:0032075 | 0.036 | 1.802 | 11 | 17 | 55 | positive regulation of nuclease activity |
| GO:0031326 | 0.036 | 1.125 | 389 | 417 | 1948 | regulation of cellular biosynthetic process |
| GO:0048706 | 0.036 | 1.613 | 17 | 24 | 84 | embryonic skeletal system development |
| GO:0006355 | 0.037 | 1.133 | 324 | 350 | 1622 | regulation of transcription, DNA-dependent |
| GO:0006890 | 0.037 | 2.815 | 3 | 7 | 17 | retrograde vesicle-mediated transport, Golgi to ER |
| GO:0021602 | 0.037 | 2.815 | 3 | 7 | 17 | cranial nerve morphogenesis |
| GO:0090329 | 0.037 | 2.815 | 3 | 7 | 17 | regulation of DNA-dependent DNA replication |
| GO:0006370 | 0.038 | 2.235 | 6 | 10 | 28 | 7-methylguanosine mRNA capping |
| GO:0007093 | 0.039 | 1.513 | 22 | 30 | 110 | mitotic cell cycle checkpoint |
| GO:0046365 | 0.039 | 1.660 | 14 | 21 | 72 | monosaccharide catabolic process |
| GO:0080090 | 0.039 | 1.112 | 527 | 557 | 2640 | regulation of primary metabolic process |
| GO:0055086 | 0.040 | 1.203 | 121 | 138 | 605 | nucleobase-containing small molecule metabolic process |
| GO:0000072 | 0.040 | Inf | 0 | 2 | 2 | M phase specific microtubule process |
| GO:0000463 | 0.040 | Inf | 0 | 2 | 2 | maturation of LSU-rRNA from tricistronic rRNA transcript (SSU-rRNA, 5.8S rRNA, LSU-rRNA) |
| GO:0000470 | 0.040 | Inf | 0 | 2 | 2 | maturation of LSU-rRNA |
| GO:0001522 | 0.040 | Inf | 0 | 2 | 2 | pseudouridine synthesis |
| GO:0006097 | 0.040 | Inf | 0 | 2 | 2 | glyoxylate cycle |
| GO:0006168 | 0.040 | Inf | 0 | 2 | 2 | adenine salvage |
| GO:0006335 | 0.040 | Inf | 0 | 2 | 2 | DNA replication-dependent nucleosome assembly |
| GO:0006426 | 0.040 | Inf | 0 | 2 | 2 | glycyl-tRNA aminoacylation |
| GO:0006428 | 0.040 | Inf | 0 | 2 | 2 | isoleucyl-tRNA aminoacylation |
| GO:0006566 | 0.040 | Inf | 0 | 2 | 2 | threonine metabolic process |
| GO:0007079 | 0.040 | Inf | 0 | 2 | 2 | mitotic chromosome movement towards spindle pole |
| GO:0008078 | 0.040 | Inf | 0 | 2 | 2 | mesodermal cell migration |
| GO:0009186 | 0.040 | Inf | 0 | 2 | 2 | deoxyribonucleoside diphosphate metabolic process |
| GO:0010826 | 0.040 | Inf | 0 | 2 | 2 | negative regulation of centrosome duplication |
| GO:0010993 | 0.040 | Inf | 0 | 2 | 2 | regulation of ubiquitin homeostasis |
| GO:0010994 | 0.040 | Inf | 0 | 2 | 2 | free ubiquitin chain polymerization |
| GO:0014908 | 0.040 | Inf | 0 | 2 | 2 | myotube differentiation involved in skeletal muscle regeneration |
| GO:0015866 | 0.040 | Inf | 0 | 2 | 2 | ADP transport |
| GO:0015880 | 0.040 | Inf | 0 | 2 | 2 | coenzyme A transport |
| GO:0021578 | 0.040 | Inf | 0 | 2 | 2 | hindbrain maturation |
| GO:0021586 | 0.040 | Inf | 0 | 2 | 2 | pons maturation |
| GO:0021626 | 0.040 | Inf | 0 | 2 | 2 | central nervous system maturation |
| GO:0021718 | 0.040 | Inf | 0 | 2 | 2 | superior olivary nucleus development |
| GO:0021722 | 0.040 | Inf | 0 | 2 | 2 | superior olivary nucleus maturation |
| GO:0031914 | 0.040 | Inf | 0 | 2 | 2 | negative regulation of synaptic plasticity |
| GO:0032416 | 0.040 | Inf | 0 | 2 | 2 | negative regulation of sodium:hydrogen antiporter activity |
| GO:0032596 | 0.040 | Inf | 0 | 2 | 2 | protein transport into membrane raft |
| GO:0033578 | 0.040 | Inf | 0 | 2 | 2 | protein glycosylation in Golgi |
| GO:0035349 | 0.040 | Inf | 0 | 2 | 2 | coenzyme A transmembrane transport |
| GO:0035519 | 0.040 | Inf | 0 | 2 | 2 | protein K29-linked ubiquitination |
| GO:0038169 | 0.040 | Inf | 0 | 2 | 2 | somatostatin receptor signaling pathway |
| GO:0038170 | 0.040 | Inf | 0 | 2 | 2 | somatostatin signaling pathway |
| GO:0042350 | 0.040 | Inf | 0 | 2 | 2 | GDP-L-fucose biosynthetic process |
| GO:0042351 | 0.040 | Inf | 0 | 2 | 2 | 'de novo' GDP-L-fucose biosynthetic process |
| GO:0042791 | 0.040 | Inf | 0 | 2 | 2 | 5S class rRNA transcription from RNA polymerase III type 1 promoter |
| GO:0042797 | 0.040 | Inf | 0 | 2 | 2 | tRNA transcription from RNA polymerase III promoter |
| GO:0043628 | 0.040 | Inf | 0 | 2 | 2 | ncRNA 3'-end processing |
| GO:0044209 | 0.040 | Inf | 0 | 2 | 2 | AMP salvage |
| GO:0046070 | 0.040 | Inf | 0 | 2 | 2 | dGTP metabolic process |
| GO:0046083 | 0.040 | Inf | 0 | 2 | 2 | adenine metabolic process |
| GO:0046084 | 0.040 | Inf | 0 | 2 | 2 | adenine biosynthetic process |
| GO:0046368 | 0.040 | Inf | 0 | 2 | 2 | GDP-L-fucose metabolic process |
| GO:0046607 | 0.040 | Inf | 0 | 2 | 2 | positive regulation of centrosome cycle |
| GO:0051305 | 0.040 | Inf | 0 | 2 | 2 | chromosome movement towards spindle pole |
| GO:0055011 | 0.040 | Inf | 0 | 2 | 2 | atrial cardiac muscle cell differentiation |
| GO:0055014 | 0.040 | Inf | 0 | 2 | 2 | atrial cardiac muscle cell development |
| GO:0055129 | 0.040 | Inf | 0 | 2 | 2 | L-proline biosynthetic process |
| GO:0060060 | 0.040 | Inf | 0 | 2 | 2 | post-embryonic retina morphogenesis in camera-type eye |
| GO:0070827 | 0.040 | Inf | 0 | 2 | 2 | chromatin maintenance |
| GO:0071106 | 0.040 | Inf | 0 | 2 | 2 | adenosine 3',5'-bisphosphate transmembrane transport |
| GO:0071281 | 0.040 | Inf | 0 | 2 | 2 | cellular response to iron ion |
| GO:0071459 | 0.040 | Inf | 0 | 2 | 2 | protein localization to chromosome, centromeric region |
| GO:0071896 | 0.040 | Inf | 0 | 2 | 2 | protein localization to adherens junction |
| GO:0071930 | 0.040 | Inf | 0 | 2 | 2 | negative regulation of transcription involved in G1/S phase of mitotic cell cycle |
| GO:0080121 | 0.040 | Inf | 0 | 2 | 2 | AMP transport |
| GO:0097411 | 0.040 | Inf | 0 | 2 | 2 | hypoxia-inducible factor-1alpha signaling pathway |
| GO:1901165 | 0.040 | Inf | 0 | 2 | 2 | positive regulation of trophoblast cell migration |
| GO:2000276 | 0.040 | Inf | 0 | 2 | 2 | negative regulation of oxidative phosphorylation uncoupler activity |
| GO:2000650 | 0.040 | Inf | 0 | 2 | 2 | negative regulation of sodium ion transmembrane transporter activity |
| GO:0042451 | 0.040 | 1.679 | 14 | 20 | 68 | purine nucleoside biosynthetic process |
| GO:0046129 | 0.040 | 1.679 | 14 | 20 | 68 | purine ribonucleoside biosynthetic process |
| GO:0006397 | 0.040 | 1.315 | 51 | 63 | 257 | mRNA processing |
| GO:0032410 | 0.042 | 2.475 | 4 | 8 | 21 | negative regulation of transporter activity |
| GO:0006303 | 0.043 | 3.015 | 3 | 6 | 14 | double-strand break repair via nonhomologous end joining |
| GO:0006337 | 0.043 | 3.015 | 3 | 6 | 14 | nucleosome disassembly |
| GO:0031498 | 0.043 | 3.015 | 3 | 6 | 14 | chromatin disassembly |
| GO:0032986 | 0.043 | 3.015 | 3 | 6 | 14 | protein-DNA complex disassembly |
| GO:0071398 | 0.043 | 3.015 | 3 | 6 | 14 | cellular response to fatty acid |
| GO:0090342 | 0.043 | 3.015 | 3 | 6 | 14 | regulation of cell aging |
| GO:0005975 | 0.043 | 1.219 | 99 | 114 | 494 | carbohydrate metabolic process |
| GO:0009260 | 0.045 | 1.378 | 35 | 44 | 173 | ribonucleotide biosynthetic process |
| GO:0016925 | 0.046 | 2.263 | 5 | 9 | 25 | protein sumoylation |
| GO:0051351 | 0.046 | 1.645 | 14 | 20 | 69 | positive regulation of ligase activity |
| GO:0006487 | 0.047 | 1.664 | 13 | 19 | 65 | protein N-linked glycosylation |
| GO:0005996 | 0.048 | 1.339 | 41 | 51 | 205 | monosaccharide metabolic process |
| GO:0018196 | 0.048 | 1.686 | 12 | 18 | 61 | peptidyl-asparagine modification |
| GO:0018279 | 0.048 | 1.686 | 12 | 18 | 61 | protein N-linked glycosylation via asparagine |
| GO:0032886 | 0.049 | 1.712 | 11 | 17 | 57 | regulation of microtubule-based process |
| GO:0006987 | 0.050 | 1.741 | 11 | 16 | 53 | activation of signaling protein activity involved in unfolded protein response |
| GO:0051438 | 0.050 | 1.583 | 16 | 22 | 78 | regulation of ubiquitin-protein ligase activity |
| GO:0034505 | 0.050 | 3.349 | 2 | 5 | 11 | tooth mineralization |
| GO:0043486 | 0.050 | 3.349 | 2 | 5 | 11 | histone exchange |
| GO:0051193 | 0.050 | 3.349 | 2 | 5 | 11 | regulation of cofactor metabolic process |
| GO:0051196 | 0.050 | 3.349 | 2 | 5 | 11 | regulation of coenzyme metabolic process |
