# Supplementary material for: Hidden Treasures in “Ancient” Microarrays: Gene-Expression Portrays Biology and Potential Resistance Pathways of Major Lung Cancer Subtypes and Normal Tissue
Source: Front Oncol. 2014 Sep 29;4:251. doi: 10.3389/fonc.2014.00251 (PMC4178426; doi:10.3389/fonc.2014.00251)
Supplement: Figure S1 — Clinically relevant differentially expressed pathways in each tissue type versus the rest. The pathway maps are arranged according to the scheme in the left-top corner. Red indicates overexpression in genes, dark green downregulation. In the Cell Cycle pathway, tumor suppressor genes were overexpressed in normal lung tissue while oncogenes and tumor driving cyclins were overexpressed in cancers. The gene thymidylate synthase (TYMS or TS, belonging to the pathway “one carbon pool by folate”) is relevant for tumor growth and is also a treatment target. Notably, TYMS was overexpressed only in the tumors that are generally refractory to the drug pemetrexed, as the squamous and the small-cell lung cancer. TYMS was not overexpressed in mesothelioma, but it is known that TYMS expression is highly variable in this cancer. In the ERBB pathway, the ERBB2/HER2 and ERBB3/HER3 were overexpressed in adenocarcinoma, while the ERBB4 was overexpressed in the carcinoids. [file Data_Sheet_1.ZIP › File S3.ZIP!/GO Analysis/Normals vs Rest Enrichment Analysis/Overrepresented GO terms of Up-regulated Genes.html]

Gene to GO BP test for over-representation

| GOBPID | Pvalue | OddsRatio | ExpCount | Count | Size | Term |
| --- | --- | --- | --- | --- | --- | --- |
| GO:0006955 | <0.0001 | 2.633 | 125 | 239 | 785 | immune response |
| GO:0002376 | <0.0001 | 2.173 | 215 | 349 | 1352 | immune system process |
| GO:0006952 | <0.0001 | 2.480 | 127 | 235 | 801 | defense response |
| GO:0050896 | <0.0001 | 1.902 | 681 | 841 | 4288 | response to stimulus |
| GO:0009611 | <0.0001 | 2.356 | 136 | 242 | 855 | response to wounding |
| GO:0030029 | <0.0001 | 3.253 | 54 | 124 | 341 | actin filament-based process |
| GO:0030036 | <0.0001 | 3.480 | 47 | 113 | 297 | actin cytoskeleton organization |
| GO:0002682 | <0.0001 | 2.477 | 103 | 193 | 648 | regulation of immune system process |
| GO:0044699 | <0.0001 | 2.259 | 1005 | 1115 | 6334 | single-organism process |
| GO:0070887 | <0.0001 | 1.990 | 209 | 324 | 1315 | cellular response to chemical stimulus |
| GO:0001775 | <0.0001 | 2.470 | 95 | 179 | 599 | cell activation |
| GO:0050793 | <0.0001 | 2.089 | 165 | 269 | 1038 | regulation of developmental process |
| GO:0048583 | <0.0001 | 1.860 | 254 | 372 | 1602 | regulation of response to stimulus |
| GO:0044763 | <0.0001 | 1.931 | 919 | 1037 | 5791 | single-organism cellular process |
| GO:0040011 | <0.0001 | 2.072 | 151 | 247 | 951 | locomotion |
| GO:0050776 | <0.0001 | 2.748 | 64 | 132 | 405 | regulation of immune response |
| GO:0016477 | <0.0001 | 2.284 | 105 | 187 | 661 | cell migration |
| GO:0002684 | <0.0001 | 2.675 | 67 | 135 | 422 | positive regulation of immune system process |
| GO:0042221 | <0.0001 | 1.752 | 330 | 453 | 2077 | response to chemical stimulus |
| GO:0048518 | <0.0001 | 1.705 | 386 | 512 | 2429 | positive regulation of biological process |
| GO:0065007 | <0.0001 | 1.768 | 834 | 956 | 5255 | biological regulation |
| GO:0006954 | <0.0001 | 2.714 | 60 | 122 | 376 | inflammatory response |
| GO:0009605 | <0.0001 | 1.988 | 154 | 245 | 969 | response to external stimulus |
| GO:0006928 | <0.0001 | 2.031 | 141 | 229 | 888 | cellular component movement |
| GO:0044767 | <0.0001 | 1.685 | 377 | 499 | 2374 | single-organism developmental process |
| GO:0048870 | <0.0001 | 2.154 | 111 | 190 | 699 | cell motility |
| GO:0051674 | <0.0001 | 2.154 | 111 | 190 | 699 | localization of cell |
| GO:0044707 | <0.0001 | 1.634 | 565 | 693 | 3559 | single-multicellular organism process |
| GO:0065008 | <0.0001 | 1.727 | 293 | 405 | 1845 | regulation of biological quality |
| GO:0051239 | <0.0001 | 1.805 | 209 | 306 | 1315 | regulation of multicellular organismal process |
| GO:0048856 | <0.0001 | 1.631 | 420 | 540 | 2648 | anatomical structure development |
| GO:0032879 | <0.0001 | 1.905 | 160 | 248 | 1010 | regulation of localization |
| GO:0048731 | <0.0001 | 1.643 | 374 | 490 | 2358 | system development |
| GO:0045087 | <0.0001 | 2.523 | 62 | 121 | 391 | innate immune response |
| GO:0051716 | <0.0001 | 1.600 | 536 | 658 | 3376 | cellular response to stimulus |
| GO:0001944 | <0.0001 | 2.463 | 65 | 125 | 411 | vasculature development |
| GO:0048584 | <0.0001 | 1.968 | 135 | 216 | 852 | positive regulation of response to stimulus |
| GO:0050789 | <0.0001 | 1.655 | 787 | 903 | 4958 | regulation of biological process |
| GO:0007165 | <0.0001 | 1.607 | 447 | 565 | 2814 | signal transduction |
| GO:0032501 | <0.0001 | 1.593 | 582 | 703 | 3664 | multicellular organismal process |
| GO:0023052 | <0.0001 | 1.595 | 499 | 619 | 3144 | signaling |
| GO:0044700 | <0.0001 | 1.595 | 499 | 619 | 3144 | single organism signaling |
| GO:0001568 | <0.0001 | 2.471 | 62 | 120 | 393 | blood vessel development |
| GO:0048869 | <0.0001 | 1.667 | 295 | 400 | 1859 | cellular developmental process |
| GO:0032502 | <0.0001 | 1.591 | 461 | 578 | 2904 | developmental process |
| GO:0007154 | <0.0001 | 1.583 | 507 | 625 | 3192 | cell communication |
| GO:0007599 | <0.0001 | 2.389 | 65 | 122 | 409 | hemostasis |
| GO:0007596 | <0.0001 | 2.393 | 64 | 121 | 405 | blood coagulation |
| GO:0030168 | <0.0001 | 3.293 | 30 | 71 | 190 | platelet activation |
| GO:0030334 | <0.0001 | 2.591 | 52 | 103 | 325 | regulation of cell migration |
| GO:0050817 | <0.0001 | 2.376 | 65 | 121 | 407 | coagulation |
| GO:0051270 | <0.0001 | 2.423 | 60 | 115 | 381 | regulation of cellular component movement |
| GO:0051128 | <0.0001 | 1.882 | 141 | 219 | 891 | regulation of cellular component organization |
| GO:0050900 | <0.0001 | 3.017 | 35 | 78 | 221 | leukocyte migration |
| GO:0042060 | <0.0001 | 2.210 | 78 | 139 | 494 | wound healing |
| GO:0048514 | <0.0001 | 2.509 | 54 | 106 | 342 | blood vessel morphogenesis |
| GO:0002252 | <0.0001 | 2.498 | 54 | 106 | 343 | immune effector process |
| GO:0071310 | <0.0001 | 1.805 | 165 | 246 | 1038 | cellular response to organic substance |
| GO:0009653 | <0.0001 | 1.694 | 228 | 319 | 1434 | anatomical structure morphogenesis |
| GO:0022610 | <0.0001 | 1.991 | 108 | 176 | 680 | biological adhesion |
| GO:0022603 | <0.0001 | 2.256 | 71 | 128 | 447 | regulation of anatomical structure morphogenesis |
| GO:0007275 | <0.0001 | 1.563 | 419 | 528 | 2641 | multicellular organismal development |
| GO:2000145 | <0.0001 | 2.472 | 54 | 105 | 342 | regulation of cell motility |
| GO:0050865 | <0.0001 | 2.660 | 45 | 91 | 281 | regulation of cell activation |
| GO:0030154 | <0.0001 | 1.627 | 283 | 380 | 1783 | cell differentiation |
| GO:0007155 | <0.0001 | 1.978 | 108 | 175 | 679 | cell adhesion |
| GO:0050878 | <0.0001 | 2.178 | 77 | 135 | 484 | regulation of body fluid levels |
| GO:0040012 | <0.0001 | 2.387 | 58 | 109 | 364 | regulation of locomotion |
| GO:2000026 | <0.0001 | 1.880 | 129 | 201 | 814 | regulation of multicellular organismal development |
| GO:0050778 | <0.0001 | 2.664 | 43 | 87 | 268 | positive regulation of immune response |
| GO:0048522 | <0.0001 | 1.569 | 353 | 454 | 2221 | positive regulation of cellular process |
| GO:0050867 | <0.0001 | 3.064 | 31 | 69 | 193 | positive regulation of cell activation |
| GO:0007015 | <0.0001 | 3.348 | 26 | 61 | 161 | actin filament organization |
| GO:0006950 | <0.0001 | 1.577 | 330 | 429 | 2079 | response to stress |
| GO:0006935 | <0.0001 | 2.156 | 76 | 132 | 476 | chemotaxis |
| GO:0042330 | <0.0001 | 2.156 | 76 | 132 | 476 | taxis |
| GO:0007010 | <0.0001 | 2.070 | 86 | 146 | 544 | cytoskeleton organization |
| GO:0072358 | <0.0001 | 2.005 | 95 | 156 | 596 | cardiovascular system development |
| GO:0072359 | <0.0001 | 2.005 | 95 | 156 | 596 | circulatory system development |
| GO:0035556 | <0.0001 | 1.676 | 207 | 291 | 1307 | intracellular signal transduction |
| GO:0001525 | <0.0001 | 2.524 | 45 | 88 | 281 | angiogenesis |
| GO:0034097 | <0.0001 | 2.209 | 64 | 114 | 402 | response to cytokine stimulus |
| GO:0032970 | <0.0001 | 3.200 | 25 | 59 | 160 | regulation of actin filament-based process |
| GO:0032956 | <0.0001 | 3.448 | 22 | 53 | 137 | regulation of actin cytoskeleton organization |
| GO:0032101 | <0.0001 | 2.402 | 49 | 94 | 311 | regulation of response to external stimulus |
| GO:0045321 | <0.0001 | 2.151 | 68 | 119 | 428 | leukocyte activation |
| GO:0010033 | <0.0001 | 1.610 | 234 | 317 | 1472 | response to organic substance |
| GO:0060326 | <0.0001 | 3.480 | 21 | 51 | 131 | cell chemotaxis |
| GO:0002576 | <0.0001 | 4.763 | 12 | 36 | 77 | platelet degranulation |
| GO:0009617 | <0.0001 | 2.603 | 38 | 77 | 240 | response to bacterium |
| GO:0006897 | <0.0001 | 2.481 | 43 | 84 | 271 | endocytosis |
| GO:0016192 | <0.0001 | 1.915 | 97 | 155 | 611 | vesicle-mediated transport |
| GO:0031347 | <0.0001 | 2.288 | 53 | 97 | 332 | regulation of defense response |
| GO:0007243 | <0.0001 | 1.861 | 104 | 162 | 653 | intracellular protein kinase cascade |
| GO:0008360 | <0.0001 | 4.626 | 12 | 35 | 76 | regulation of cell shape |
| GO:0008283 | <0.0001 | 1.644 | 185 | 258 | 1163 | cell proliferation |
| GO:0048513 | <0.0001 | 1.548 | 272 | 356 | 1712 | organ development |
| GO:0050794 | <0.0001 | 1.482 | 745 | 841 | 4695 | regulation of cellular process |
| GO:0002696 | <0.0001 | 2.806 | 29 | 62 | 183 | positive regulation of leukocyte activation |
| GO:0071345 | <0.0001 | 2.241 | 52 | 94 | 326 | cellular response to cytokine stimulus |
| GO:0001816 | <0.0001 | 2.265 | 50 | 91 | 313 | cytokine production |
| GO:0051094 | <0.0001 | 1.926 | 84 | 136 | 531 | positive regulation of developmental process |
| GO:0048646 | <0.0001 | 1.628 | 180 | 250 | 1132 | anatomical structure formation involved in morphogenesis |
| GO:0032989 | <0.0001 | 1.793 | 110 | 168 | 696 | cellular component morphogenesis |
| GO:0002253 | <0.0001 | 2.575 | 34 | 69 | 216 | activation of immune response |
| GO:0002694 | <0.0001 | 2.411 | 41 | 78 | 256 | regulation of leukocyte activation |
| GO:0072001 | <0.0001 | 2.712 | 29 | 61 | 184 | renal system development |
| GO:0045597 | <0.0001 | 2.069 | 62 | 106 | 390 | positive regulation of cell differentiation |
| GO:0000902 | <0.0001 | 1.790 | 104 | 159 | 658 | cell morphogenesis |
| GO:0050727 | <0.0001 | 2.919 | 24 | 53 | 152 | regulation of inflammatory response |
| GO:0030595 | <0.0001 | 3.456 | 17 | 42 | 108 | leukocyte chemotaxis |
| GO:0007166 | <0.0001 | 1.512 | 264 | 342 | 1664 | cell surface receptor signaling pathway |
| GO:0032103 | <0.0001 | 3.110 | 21 | 48 | 132 | positive regulation of response to external stimulus |
| GO:0009888 | <0.0001 | 1.669 | 143 | 205 | 902 | tissue development |
| GO:0009607 | <0.0001 | 1.991 | 68 | 113 | 428 | response to biotic stimulus |
| GO:0007167 | <0.0001 | 1.756 | 110 | 165 | 693 | enzyme linked receptor protein signaling pathway |
| GO:0045595 | <0.0001 | 1.730 | 117 | 173 | 736 | regulation of cell differentiation |
| GO:0048519 | <0.0001 | 1.457 | 347 | 430 | 2184 | negative regulation of biological process |
| GO:0071495 | <0.0001 | 1.828 | 91 | 141 | 572 | cellular response to endogenous stimulus |
| GO:0030335 | <0.0001 | 2.567 | 31 | 62 | 194 | positive regulation of cell migration |
| GO:0051707 | <0.0001 | 1.980 | 66 | 109 | 414 | response to other organism |
| GO:0002697 | <0.0001 | 2.703 | 27 | 56 | 169 | regulation of immune effector process |
| GO:0030097 | <0.0001 | 2.004 | 63 | 105 | 395 | hemopoiesis |
| GO:0051272 | <0.0001 | 2.500 | 32 | 64 | 204 | positive regulation of cellular component movement |
| GO:0001655 | <0.0001 | 2.429 | 35 | 67 | 218 | urogenital system development |
| GO:0080134 | <0.0001 | 1.815 | 89 | 138 | 562 | regulation of response to stress |
| GO:0051251 | <0.0001 | 2.701 | 26 | 55 | 166 | positive regulation of lymphocyte activation |
| GO:0009966 | <0.0001 | 1.550 | 194 | 261 | 1225 | regulation of signal transduction |
| GO:0040017 | <0.0001 | 2.464 | 33 | 64 | 206 | positive regulation of locomotion |
| GO:0030155 | <0.0001 | 2.423 | 34 | 66 | 215 | regulation of cell adhesion |
| GO:0048468 | <0.0001 | 1.580 | 170 | 233 | 1072 | cell development |
| GO:2000147 | <0.0001 | 2.490 | 31 | 62 | 198 | positive regulation of cell motility |
| GO:0042127 | <0.0001 | 1.634 | 141 | 199 | 887 | regulation of cell proliferation |
| GO:0023051 | <0.0001 | 1.514 | 223 | 293 | 1408 | regulation of signaling |
| GO:0046649 | <0.0001 | 2.027 | 57 | 97 | 361 | lymphocyte activation |
| GO:0071363 | <0.0001 | 2.038 | 56 | 95 | 352 | cellular response to growth factor stimulus |
| GO:0010646 | <0.0001 | 1.510 | 224 | 293 | 1410 | regulation of cell communication |
| GO:0006887 | <0.0001 | 2.472 | 32 | 62 | 199 | exocytosis |
| GO:0031589 | <0.0001 | 2.609 | 27 | 56 | 173 | cell-substrate adhesion |
| GO:0031349 | <0.0001 | 2.551 | 29 | 58 | 182 | positive regulation of defense response |
| GO:0009306 | <0.0001 | 3.023 | 20 | 44 | 123 | protein secretion |
| GO:0009719 | <0.0001 | 1.631 | 137 | 194 | 865 | response to endogenous stimulus |
| GO:0070848 | <0.0001 | 2.004 | 58 | 97 | 364 | response to growth factor stimulus |
| GO:0051249 | <0.0001 | 2.349 | 35 | 67 | 223 | regulation of lymphocyte activation |
| GO:0051493 | <0.0001 | 2.480 | 30 | 60 | 192 | regulation of cytoskeleton organization |
| GO:0002443 | <0.0001 | 2.647 | 26 | 53 | 162 | leukocyte mediated immunity |
| GO:0032496 | <0.0001 | 2.602 | 27 | 54 | 167 | response to lipopolysaccharide |
| GO:0051179 | <0.0001 | 1.400 | 431 | 513 | 2714 | localization |
| GO:0002250 | <0.0001 | 2.719 | 24 | 50 | 150 | adaptive immune response |
| GO:0048534 | <0.0001 | 1.907 | 67 | 108 | 421 | hematopoietic or lymphoid organ development |
| GO:0002237 | <0.0001 | 2.543 | 28 | 56 | 176 | response to molecule of bacterial origin |
| GO:1901342 | <0.0001 | 2.838 | 21 | 46 | 134 | regulation of vasculature development |
| GO:0002520 | <0.0001 | 1.872 | 70 | 112 | 443 | immune system development |
| GO:0019221 | <0.0001 | 2.169 | 43 | 76 | 268 | cytokine-mediated signaling pathway |
| GO:0051049 | <0.0001 | 1.666 | 116 | 168 | 733 | regulation of transport |
| GO:0002764 | <0.0001 | 2.470 | 30 | 58 | 186 | immune response-regulating signaling pathway |
| GO:0050663 | <0.0001 | 4.194 | 10 | 28 | 64 | cytokine secretion |
| GO:0046903 | <0.0001 | 1.744 | 93 | 140 | 587 | secretion |
| GO:0045785 | <0.0001 | 3.229 | 16 | 37 | 99 | positive regulation of cell adhesion |
| GO:0051017 | <0.0001 | 4.080 | 10 | 28 | 65 | actin filament bundle assembly |
| GO:0071241 | <0.0001 | 3.637 | 12 | 31 | 77 | cellular response to inorganic substance |
| GO:0045765 | <0.0001 | 2.843 | 20 | 43 | 125 | regulation of angiogenesis |
| GO:0001817 | <0.0001 | 2.098 | 44 | 77 | 278 | regulation of cytokine production |
| GO:0008154 | <0.0001 | 3.340 | 14 | 34 | 89 | actin polymerization or depolymerization |
| GO:0034341 | <0.0001 | 3.340 | 14 | 34 | 89 | response to interferon-gamma |
| GO:0022604 | <0.0001 | 2.268 | 35 | 64 | 218 | regulation of cell morphogenesis |
| GO:0000165 | <0.0001 | 1.874 | 64 | 103 | 406 | MAPK cascade |
| GO:0030593 | <0.0001 | 4.928 | 7 | 22 | 46 | neutrophil chemotaxis |
| GO:0042110 | <0.0001 | 2.083 | 43 | 75 | 272 | T cell activation |
| GO:0030030 | <0.0001 | 1.664 | 103 | 150 | 652 | cell projection organization |
| GO:0032940 | <0.0001 | 1.755 | 81 | 123 | 511 | secretion by cell |
| GO:0048523 | <0.0001 | 1.400 | 318 | 389 | 2006 | negative regulation of cellular process |
| GO:0032943 | <0.0001 | 2.576 | 23 | 47 | 146 | mononuclear cell proliferation |
| GO:0071248 | <0.0001 | 3.553 | 12 | 29 | 73 | cellular response to metal ion |
| GO:0010627 | <0.0001 | 1.731 | 82 | 123 | 516 | regulation of intracellular protein kinase cascade |
| GO:0071346 | <0.0001 | 3.442 | 12 | 30 | 77 | cellular response to interferon-gamma |
| GO:0018212 | <0.0001 | 2.430 | 26 | 51 | 165 | peptidyl-tyrosine modification |
| GO:0050708 | <0.0001 | 3.112 | 15 | 34 | 93 | regulation of protein secretion |
| GO:0034330 | <0.0001 | 2.504 | 24 | 48 | 152 | cell junction organization |
| GO:0030041 | <0.0001 | 3.473 | 12 | 29 | 74 | actin filament polymerization |
| GO:0070661 | <0.0001 | 2.480 | 24 | 48 | 153 | leukocyte proliferation |
| GO:0065009 | <0.0001 | 1.455 | 206 | 265 | 1299 | regulation of molecular function |
| GO:0033993 | <0.0001 | 1.772 | 71 | 109 | 448 | response to lipid |
| GO:0018108 | <0.0001 | 2.401 | 26 | 50 | 163 | peptidyl-tyrosine phosphorylation |
| GO:0002685 | <0.0001 | 3.397 | 12 | 29 | 75 | regulation of leukocyte migration |
| GO:0034329 | <0.0001 | 2.563 | 22 | 44 | 137 | cell junction assembly |
| GO:0050870 | <0.0001 | 2.563 | 22 | 44 | 137 | positive regulation of T cell activation |
| GO:0002757 | <0.0001 | 2.322 | 28 | 53 | 177 | immune response-activating signal transduction |
| GO:0050863 | <0.0001 | 2.322 | 28 | 53 | 177 | regulation of T cell activation |
| GO:0043149 | <0.0001 | 5.098 | 6 | 19 | 39 | stress fiber assembly |
| GO:0050707 | <0.0001 | 4.121 | 8 | 23 | 53 | regulation of cytokine secretion |
| GO:0002274 | <0.0001 | 2.960 | 15 | 34 | 96 | myeloid leukocyte activation |
| GO:0010811 | <0.0001 | 4.668 | 7 | 20 | 43 | positive regulation of cell-substrate adhesion |
| GO:0060333 | <0.0001 | 3.736 | 10 | 25 | 61 | interferon-gamma-mediated signaling pathway |
| GO:0030099 | <0.0001 | 2.221 | 31 | 56 | 193 | myeloid cell differentiation |
| GO:0044087 | <0.0001 | 2.007 | 42 | 71 | 264 | regulation of cellular component biogenesis |
| GO:0002460 | <0.0001 | 2.530 | 21 | 43 | 135 | adaptive immune response based on somatic recombination of immune receptors built from immunoglobulin superfamily domains |
| GO:0006909 | <0.0001 | 3.461 | 11 | 27 | 69 | phagocytosis |
| GO:0046651 | <0.0001 | 2.462 | 23 | 45 | 144 | lymphocyte proliferation |
| GO:0001822 | <0.0001 | 2.419 | 24 | 46 | 149 | kidney development |
| GO:0071294 | <0.0001 | 42.669 | 1 | 8 | 9 | cellular response to zinc ion |
| GO:0002573 | <0.0001 | 2.777 | 17 | 36 | 106 | myeloid leukocyte differentiation |
| GO:0002768 | <0.0001 | 2.777 | 17 | 36 | 106 | immune response-regulating cell surface receptor signaling pathway |
| GO:0019220 | <0.0001 | 1.513 | 142 | 190 | 892 | regulation of phosphate metabolic process |
| GO:0032835 | <0.0001 | 4.473 | 7 | 20 | 44 | glomerulus development |
| GO:0051223 | <0.0001 | 2.100 | 35 | 61 | 219 | regulation of protein transport |
| GO:0051050 | <0.0001 | 1.807 | 59 | 92 | 371 | positive regulation of transport |
| GO:0060627 | <0.0001 | 2.344 | 25 | 48 | 159 | regulation of vesicle-mediated transport |
| GO:0051093 | <0.0001 | 1.784 | 61 | 95 | 387 | negative regulation of developmental process |
| GO:0051271 | <0.0001 | 2.738 | 17 | 36 | 107 | negative regulation of cellular component movement |
| GO:0051174 | <0.0001 | 1.503 | 143 | 191 | 901 | regulation of phosphorus metabolic process |
| GO:0016043 | <0.0001 | 1.331 | 424 | 493 | 2670 | cellular component organization |
| GO:0050714 | <0.0001 | 3.411 | 11 | 26 | 67 | positive regulation of protein secretion |
| GO:0010543 | <0.0001 | 7.726 | 3 | 13 | 22 | regulation of platelet activation |
| GO:0070372 | <0.0001 | 2.983 | 14 | 31 | 87 | regulation of ERK1 and ERK2 cascade |
| GO:0070201 | <0.0001 | 2.022 | 38 | 65 | 240 | regulation of establishment of protein localization |
| GO:0030336 | <0.0001 | 2.779 | 16 | 34 | 100 | negative regulation of cell migration |
| GO:0032880 | <0.0001 | 1.934 | 44 | 72 | 275 | regulation of protein localization |
| GO:0030100 | <0.0001 | 2.825 | 15 | 33 | 96 | regulation of endocytosis |
| GO:0003013 | <0.0001 | 1.894 | 47 | 76 | 295 | circulatory system process |
| GO:0007159 | <0.0001 | 5.061 | 6 | 17 | 35 | leukocyte cell-cell adhesion |
| GO:0000904 | <0.0001 | 1.646 | 83 | 121 | 526 | cell morphogenesis involved in differentiation |
| GO:0002699 | <0.0001 | 2.993 | 13 | 30 | 84 | positive regulation of immune effector process |
| GO:0061061 | <0.0001 | 1.803 | 56 | 87 | 351 | muscle structure development |
| GO:0051924 | <0.0001 | 2.698 | 17 | 35 | 105 | regulation of calcium ion transport |
| GO:0033559 | <0.0001 | 3.811 | 8 | 22 | 53 | unsaturated fatty acid metabolic process |
| GO:0010959 | <0.0001 | 2.342 | 24 | 45 | 149 | regulation of metal ion transport |
| GO:0033002 | <0.0001 | 2.827 | 15 | 32 | 93 | muscle cell proliferation |
| GO:0002444 | <0.0001 | 4.595 | 6 | 18 | 39 | myeloid leukocyte mediated immunity |
| GO:0016337 | <0.0001 | 1.949 | 41 | 68 | 258 | cell-cell adhesion |
| GO:0050729 | <0.0001 | 3.486 | 10 | 24 | 61 | positive regulation of inflammatory response |
| GO:0008064 | <0.0001 | 3.252 | 11 | 26 | 69 | regulation of actin polymerization or depolymerization |
| GO:2000146 | <0.0001 | 2.696 | 16 | 34 | 102 | negative regulation of cell motility |
| GO:0006690 | <0.0001 | 4.245 | 7 | 19 | 43 | icosanoid metabolic process |
| GO:1901568 | <0.0001 | 4.245 | 7 | 19 | 43 | fatty acid derivative metabolic process |
| GO:0008015 | <0.0001 | 1.867 | 47 | 75 | 294 | blood circulation |
| GO:0019216 | <0.0001 | 2.252 | 25 | 47 | 160 | regulation of lipid metabolic process |
| GO:0002521 | <0.0001 | 1.917 | 42 | 69 | 265 | leukocyte differentiation |
| GO:0010563 | <0.0001 | 2.107 | 31 | 54 | 193 | negative regulation of phosphorus metabolic process |
| GO:0045936 | <0.0001 | 2.107 | 31 | 54 | 193 | negative regulation of phosphate metabolic process |
| GO:0042098 | <0.0001 | 2.657 | 16 | 34 | 103 | T cell proliferation |
| GO:0030832 | <0.0001 | 3.177 | 11 | 26 | 70 | regulation of actin filament length |
| GO:0043408 | <0.0001 | 1.773 | 56 | 86 | 351 | regulation of MAPK cascade |
| GO:0009612 | <0.0001 | 2.556 | 18 | 36 | 112 | response to mechanical stimulus |
| GO:0051240 | <0.0001 | 1.712 | 64 | 96 | 403 | positive regulation of multicellular organismal process |
| GO:0043304 | <0.0001 | 10.678 | 2 | 10 | 15 | regulation of mast cell degranulation |
| GO:0007178 | <0.0001 | 2.053 | 32 | 56 | 204 | transmembrane receptor protein serine/threonine kinase signaling pathway |
| GO:0009725 | <0.0001 | 1.602 | 86 | 122 | 541 | response to hormone stimulus |
| GO:0061437 | <0.0001 | 8.394 | 3 | 11 | 18 | renal system vasculature development |
| GO:0061440 | <0.0001 | 8.394 | 3 | 11 | 18 | kidney vasculature development |
| GO:0051336 | <0.0001 | 1.592 | 87 | 123 | 548 | regulation of hydrolase activity |
| GO:0045807 | <0.0001 | 3.635 | 8 | 21 | 52 | positive regulation of endocytosis |
| GO:0050715 | <0.0001 | 4.194 | 7 | 18 | 41 | positive regulation of cytokine secretion |
| GO:0006636 | <0.0001 | 4.760 | 5 | 16 | 34 | unsaturated fatty acid biosynthetic process |
| GO:0051492 | <0.0001 | 4.760 | 5 | 16 | 34 | regulation of stress fiber assembly |
| GO:0008284 | <0.0001 | 1.618 | 80 | 114 | 501 | positive regulation of cell proliferation |
| GO:0045621 | <0.0001 | 3.473 | 9 | 22 | 56 | positive regulation of lymphocyte differentiation |
| GO:0070371 | <0.0001 | 2.615 | 16 | 33 | 101 | ERK1 and ERK2 cascade |
| GO:0007160 | <0.0001 | 2.413 | 20 | 38 | 123 | cell-matrix adhesion |
| GO:0048659 | <0.0001 | 3.223 | 10 | 24 | 64 | smooth muscle cell proliferation |
| GO:0010942 | <0.0001 | 1.750 | 55 | 84 | 346 | positive regulation of cell death |
| GO:0001932 | <0.0001 | 1.551 | 97 | 134 | 610 | regulation of protein phosphorylation |
| GO:0071560 | <0.0001 | 2.182 | 25 | 46 | 160 | cellular response to transforming growth factor beta stimulus |
| GO:0060537 | <0.0001 | 1.899 | 40 | 65 | 251 | muscle tissue development |
| GO:0032231 | <0.0001 | 4.336 | 6 | 17 | 38 | regulation of actin filament bundle assembly |
| GO:0060191 | <0.0001 | 2.737 | 14 | 30 | 89 | regulation of lipase activity |
| GO:0048871 | <0.0001 | 2.457 | 18 | 36 | 115 | multicellular organismal homeostasis |
| GO:0043068 | <0.0001 | 1.760 | 53 | 81 | 332 | positive regulation of programmed cell death |
| GO:0044255 | <0.0001 | 1.595 | 83 | 117 | 520 | cellular lipid metabolic process |
| GO:0002429 | <0.0001 | 2.611 | 16 | 32 | 98 | immune response-activating cell surface receptor signaling pathway |
| GO:0051100 | <0.0001 | 3.373 | 9 | 22 | 57 | negative regulation of binding |
| GO:0071559 | <0.0001 | 2.163 | 26 | 46 | 161 | response to transforming growth factor beta stimulus |
| GO:0045638 | <0.0001 | 4.019 | 7 | 18 | 42 | negative regulation of myeloid cell differentiation |
| GO:0007169 | <0.0001 | 1.602 | 79 | 113 | 500 | transmembrane receptor protein tyrosine kinase signaling pathway |
| GO:0001933 | <0.0001 | 2.246 | 23 | 42 | 143 | negative regulation of protein phosphorylation |
| GO:0071840 | <0.0001 | 1.295 | 431 | 494 | 2717 | cellular component organization or biogenesis |
| GO:0002548 | <0.0001 | 5.792 | 4 | 13 | 25 | monocyte chemotaxis |
| GO:0002449 | <0.0001 | 2.356 | 20 | 38 | 125 | lymphocyte mediated immunity |
| GO:0033006 | <0.0001 | 8.897 | 3 | 10 | 16 | regulation of mast cell activation involved in immune response |
| GO:0050866 | <0.0001 | 2.737 | 14 | 29 | 86 | negative regulation of cell activation |
| GO:0022008 | <0.0001 | 1.464 | 131 | 172 | 823 | neurogenesis |
| GO:0006816 | <0.0001 | 2.088 | 28 | 49 | 176 | calcium ion transport |
| GO:0002886 | <0.0001 | 7.344 | 3 | 11 | 19 | regulation of myeloid leukocyte mediated immunity |
| GO:0043300 | <0.0001 | 7.344 | 3 | 11 | 19 | regulation of leukocyte degranulation |
| GO:0031175 | <0.0001 | 1.582 | 83 | 117 | 523 | neuron projection development |
| GO:1901700 | <0.0001 | 1.534 | 98 | 134 | 615 | response to oxygen-containing compound |
| GO:0010035 | <0.0001 | 1.830 | 43 | 69 | 274 | response to inorganic substance |
| GO:0043549 | <0.0001 | 1.606 | 76 | 109 | 481 | regulation of kinase activity |
| GO:0031032 | <0.0001 | 4.721 | 5 | 15 | 32 | actomyosin structure organization |
| GO:0046456 | <0.0001 | 4.721 | 5 | 15 | 32 | icosanoid biosynthetic process |
| GO:1901570 | <0.0001 | 4.721 | 5 | 15 | 32 | fatty acid derivative biosynthetic process |
| GO:0040013 | <0.0001 | 2.396 | 19 | 36 | 117 | negative regulation of locomotion |
| GO:0043065 | <0.0001 | 1.744 | 52 | 80 | 330 | positive regulation of apoptotic process |
| GO:0048660 | <0.0001 | 3.166 | 10 | 23 | 62 | regulation of smooth muscle cell proliferation |
| GO:0071216 | <0.0001 | 2.689 | 14 | 29 | 87 | cellular response to biotic stimulus |
| GO:0051338 | <0.0001 | 1.593 | 78 | 111 | 493 | regulation of transferase activity |
| GO:0050790 | <0.0001 | 1.407 | 167 | 212 | 1052 | regulation of catalytic activity |
| GO:0048661 | <0.0001 | 4.282 | 6 | 16 | 36 | positive regulation of smooth muscle cell proliferation |
| GO:0045859 | <0.0001 | 1.613 | 73 | 104 | 457 | regulation of protein kinase activity |
| GO:0070838 | <0.0001 | 2.034 | 29 | 50 | 183 | divalent metal ion transport |
| GO:0023056 | <0.0001 | 1.515 | 101 | 137 | 635 | positive regulation of signaling |
| GO:0050921 | <0.0001 | 2.919 | 11 | 25 | 71 | positive regulation of chemotaxis |
| GO:0008285 | <0.0001 | 1.636 | 67 | 97 | 421 | negative regulation of cell proliferation |
| GO:0006898 | <0.0001 | 2.410 | 17 | 34 | 110 | receptor-mediated endocytosis |
| GO:0090066 | <0.0001 | 1.905 | 35 | 58 | 223 | regulation of anatomical structure size |
| GO:0009967 | <0.0001 | 1.524 | 95 | 130 | 599 | positive regulation of signal transduction |
| GO:0003012 | <0.0001 | 1.881 | 37 | 60 | 233 | muscle system process |
| GO:0070663 | <0.0001 | 2.357 | 18 | 35 | 115 | regulation of leukocyte proliferation |
| GO:0002703 | <0.0001 | 2.794 | 12 | 26 | 76 | regulation of leukocyte mediated immunity |
| GO:0043269 | <0.0001 | 1.920 | 34 | 56 | 214 | regulation of ion transport |
| GO:0042439 | <0.0001 | 3.511 | 8 | 19 | 48 | ethanolamine-containing compound metabolic process |
| GO:0048699 | <0.0001 | 1.455 | 123 | 162 | 777 | generation of neurons |
| GO:0045637 | <0.0001 | 2.460 | 16 | 32 | 102 | regulation of myeloid cell differentiation |
| GO:0072012 | <0.0001 | 7.625 | 3 | 10 | 17 | glomerulus vasculature development |
| GO:0050920 | <0.0001 | 2.599 | 14 | 29 | 89 | regulation of chemotaxis |
| GO:0007264 | <0.0001 | 1.713 | 52 | 79 | 330 | small GTPase mediated signal transduction |
| GO:0014070 | <0.0001 | 1.605 | 71 | 101 | 445 | response to organic cyclic compound |
| GO:0032944 | <0.0001 | 2.378 | 18 | 34 | 111 | regulation of mononuclear cell proliferation |
| GO:0070374 | <0.0001 | 3.010 | 10 | 23 | 64 | positive regulation of ERK1 and ERK2 cascade |
| GO:0034134 | <0.0001 | 3.106 | 10 | 22 | 60 | toll-like receptor 2 signaling pathway |
| GO:0006915 | <0.0001 | 1.380 | 179 | 224 | 1129 | apoptotic process |
| GO:0010810 | <0.0001 | 2.640 | 13 | 28 | 85 | regulation of cell-substrate adhesion |
| GO:0002683 | <0.0001 | 2.214 | 21 | 39 | 134 | negative regulation of immune system process |
| GO:0051222 | <0.0001 | 2.214 | 21 | 39 | 134 | positive regulation of protein transport |
| GO:0072511 | <0.0001 | 1.988 | 30 | 50 | 186 | divalent inorganic cation transport |
| GO:0008219 | <0.0001 | 1.361 | 200 | 246 | 1257 | cell death |
| GO:0042325 | <0.0001 | 1.491 | 103 | 139 | 652 | regulation of phosphorylation |
| GO:0048520 | <0.0001 | 2.738 | 12 | 26 | 77 | positive regulation of behavior |
| GO:0012501 | <0.0001 | 1.375 | 180 | 225 | 1137 | programmed cell death |
| GO:0006936 | <0.0001 | 1.923 | 33 | 54 | 206 | muscle contraction |
| GO:0042742 | <0.0001 | 2.797 | 12 | 25 | 73 | defense response to bacterium |
| GO:0016265 | <0.0001 | 1.358 | 200 | 246 | 1259 | death |
| GO:0051246 | <0.0001 | 1.386 | 168 | 211 | 1058 | regulation of protein metabolic process |
| GO:0071347 | <0.0001 | 4.223 | 5 | 15 | 34 | cellular response to interleukin-1 |
| GO:0010038 | <0.0001 | 1.956 | 30 | 51 | 192 | response to metal ion |
| GO:0002480 | <0.0001 | 31.950 | 1 | 6 | 7 | antigen processing and presentation of exogenous peptide antigen via MHC class I, TAP-independent |
| GO:0072239 | <0.0001 | 31.950 | 1 | 6 | 7 | metanephric glomerulus vasculature development |
| GO:0022607 | <0.0001 | 1.390 | 160 | 202 | 1009 | cellular component assembly |
| GO:0006956 | <0.0001 | 3.892 | 6 | 16 | 38 | complement activation |
| GO:0071222 | <0.0001 | 2.685 | 12 | 26 | 78 | cellular response to lipopolysaccharide |
| GO:0050851 | <0.0001 | 2.514 | 14 | 29 | 91 | antigen receptor-mediated signaling pathway |
| GO:0006468 | <0.0001 | 1.419 | 136 | 175 | 857 | protein phosphorylation |
| GO:0045088 | <0.0001 | 1.999 | 28 | 47 | 174 | regulation of innate immune response |
| GO:0030182 | <0.0001 | 1.457 | 113 | 149 | 712 | neuron differentiation |
| GO:0044093 | <0.0001 | 1.423 | 133 | 171 | 835 | positive regulation of molecular function |
| GO:0010647 | <0.0001 | 1.483 | 101 | 135 | 635 | positive regulation of cell communication |
| GO:0002819 | <0.0001 | 2.800 | 11 | 24 | 70 | regulation of adaptive immune response |
| GO:0045123 | <0.0001 | 5.873 | 3 | 11 | 21 | cellular extravasation |
| GO:0040007 | <0.0001 | 1.514 | 89 | 121 | 559 | growth |
| GO:0051130 | <0.0001 | 1.638 | 59 | 86 | 372 | positive regulation of cellular component organization |
| GO:0030833 | <0.0001 | 2.950 | 10 | 22 | 62 | regulation of actin filament polymerization |
| GO:0032355 | <0.0001 | 2.950 | 10 | 22 | 62 | response to estradiol stimulus |
| GO:0002446 | <0.0001 | 8.001 | 2 | 9 | 15 | neutrophil mediated immunity |
| GO:0009887 | <0.0001 | 1.505 | 91 | 123 | 571 | organ morphogenesis |
| GO:0050670 | <0.0001 | 2.306 | 17 | 33 | 110 | regulation of lymphocyte proliferation |
| GO:0010562 | <0.0001 | 1.532 | 81 | 112 | 512 | positive regulation of phosphorus metabolic process |
| GO:0045937 | <0.0001 | 1.532 | 81 | 112 | 512 | positive regulation of phosphate metabolic process |
| GO:0023057 | <0.0001 | 1.557 | 74 | 104 | 469 | negative regulation of signaling |
| GO:0010740 | <0.0001 | 1.639 | 58 | 84 | 363 | positive regulation of intracellular protein kinase cascade |
| GO:0007179 | <0.0001 | 2.123 | 22 | 39 | 138 | transforming growth factor beta receptor signaling pathway |
| GO:0060429 | <0.0001 | 1.582 | 68 | 96 | 427 | epithelium development |
| GO:0009968 | <0.0001 | 1.571 | 70 | 99 | 443 | negative regulation of signal transduction |
| GO:0048666 | <0.0001 | 1.488 | 94 | 127 | 595 | neuron development |
| GO:0014068 | <0.0001 | 3.500 | 7 | 17 | 43 | positive regulation of phosphatidylinositol 3-kinase cascade |
| GO:0030316 | <0.0001 | 3.500 | 7 | 17 | 43 | osteoclast differentiation |
| GO:0032844 | <0.0001 | 1.836 | 35 | 56 | 221 | regulation of homeostatic process |
| GO:0002758 | <0.0001 | 2.348 | 16 | 31 | 102 | innate immune response-activating signal transduction |
| GO:0010648 | <0.0001 | 1.548 | 75 | 104 | 471 | negative regulation of cell communication |
| GO:0051092 | <0.0001 | 2.585 | 13 | 26 | 80 | positive regulation of NF-kappaB transcription factor activity |
| GO:0032675 | <0.0001 | 3.323 | 7 | 18 | 47 | regulation of interleukin-6 production |
| GO:0070555 | <0.0001 | 3.062 | 9 | 20 | 55 | response to interleukin-1 |
| GO:0072376 | <0.0001 | 3.062 | 9 | 20 | 55 | protein activation cascade |
| GO:0010043 | <0.0001 | 4.930 | 4 | 12 | 25 | response to zinc ion |
| GO:0042326 | <0.0001 | 2.017 | 25 | 43 | 158 | negative regulation of phosphorylation |
| GO:0061041 | <0.0001 | 2.631 | 12 | 25 | 76 | regulation of wound healing |
| GO:0009893 | <0.0001 | 1.316 | 232 | 278 | 1461 | positive regulation of metabolic process |
| GO:0034109 | <0.0001 | 3.820 | 6 | 15 | 36 | homotypic cell-cell adhesion |
| GO:0040008 | <0.0001 | 1.608 | 60 | 86 | 377 | regulation of growth |
| GO:0072006 | <0.0001 | 2.741 | 11 | 23 | 68 | nephron development |
| GO:0001954 | <0.0001 | 5.338 | 3 | 11 | 22 | positive regulation of cell-matrix adhesion |
| GO:0033003 | <0.0001 | 5.338 | 3 | 11 | 22 | regulation of mast cell activation |
| GO:0006633 | <0.0001 | 2.538 | 13 | 26 | 81 | fatty acid biosynthetic process |
| GO:0045619 | <0.0001 | 2.538 | 13 | 26 | 81 | regulation of lymphocyte differentiation |
| GO:0071219 | <0.0001 | 2.538 | 13 | 26 | 81 | cellular response to molecule of bacterial origin |
| GO:0002755 | <0.0001 | 2.808 | 10 | 22 | 64 | MyD88-dependent toll-like receptor signaling pathway |
| GO:0031532 | <0.0001 | 3.369 | 7 | 17 | 44 | actin cytoskeleton reorganization |
| GO:0002695 | <0.0001 | 2.580 | 12 | 25 | 77 | negative regulation of leukocyte activation |
| GO:0045834 | <0.0001 | 2.580 | 12 | 25 | 77 | positive regulation of lipid metabolic process |
| GO:0006629 | <0.0001 | 1.424 | 117 | 152 | 739 | lipid metabolic process |
| GO:0032635 | <0.0001 | 3.212 | 8 | 18 | 48 | interleukin-6 production |
| GO:0001763 | <0.0001 | 2.004 | 25 | 42 | 155 | morphogenesis of a branching structure |
| GO:0048812 | <0.0001 | 1.557 | 69 | 96 | 432 | neuron projection morphogenesis |
| GO:0002218 | <0.0001 | 2.283 | 17 | 31 | 104 | activation of innate immune response |
| GO:0002263 | <0.0001 | 2.283 | 17 | 31 | 104 | cell activation involved in immune response |
| GO:0002366 | <0.0001 | 2.283 | 17 | 31 | 104 | leukocyte activation involved in immune response |
| GO:0010744 | <0.0001 | 8.529 | 2 | 8 | 13 | positive regulation of macrophage derived foam cell differentiation |
| GO:0072224 | <0.0001 | 8.529 | 2 | 8 | 13 | metanephric glomerulus development |
| GO:0006650 | <0.0001 | 2.033 | 23 | 40 | 146 | glycerophospholipid metabolic process |
| GO:0019370 | <0.0001 | 6.857 | 3 | 9 | 16 | leukotriene biosynthetic process |
| GO:0061082 | <0.0001 | 6.857 | 3 | 9 | 16 | myeloid leukocyte cytokine production |
| GO:0060548 | <0.0001 | 1.520 | 77 | 105 | 482 | negative regulation of cell death |
| GO:0043627 | <0.0001 | 2.234 | 17 | 32 | 109 | response to estrogen stimulus |
| GO:0042592 | <0.0001 | 1.382 | 142 | 179 | 894 | homeostatic process |
| GO:0071822 | <0.0001 | 1.427 | 112 | 146 | 708 | protein complex subunit organization |
| GO:0002822 | <0.0001 | 2.742 | 10 | 22 | 65 | regulation of adaptive immune response based on somatic recombination of immune receptors built from immunoglobulin superfamily domains |
| GO:0048858 | <0.0001 | 1.521 | 76 | 104 | 477 | cell projection morphogenesis |
| GO:0002761 | <0.0001 | 2.813 | 10 | 21 | 61 | regulation of myeloid leukocyte differentiation |
| GO:0043067 | <0.0001 | 1.378 | 143 | 180 | 901 | regulation of programmed cell death |
| GO:0010941 | <0.0001 | 1.373 | 147 | 184 | 924 | regulation of cell death |
| GO:0061138 | <0.0001 | 2.014 | 23 | 40 | 147 | morphogenesis of a branching epithelium |
| GO:0034130 | <0.0001 | 2.895 | 9 | 20 | 57 | toll-like receptor 1 signaling pathway |
| GO:0002688 | <0.0001 | 2.992 | 8 | 19 | 53 | regulation of leukocyte chemotaxis |
| GO:0006644 | <0.0001 | 1.812 | 33 | 53 | 211 | phospholipid metabolic process |
| GO:0016525 | <0.0001 | 3.249 | 7 | 17 | 45 | negative regulation of angiogenesis |
| GO:0048144 | <0.0001 | 3.108 | 8 | 18 | 49 | fibroblast proliferation |
| GO:0050730 | <0.0001 | 2.116 | 20 | 35 | 124 | regulation of peptidyl-tyrosine phosphorylation |
| GO:0010517 | <0.0001 | 2.624 | 11 | 23 | 70 | regulation of phospholipase activity |
| GO:0007411 | <0.0001 | 1.681 | 45 | 67 | 283 | axon guidance |
| GO:0014706 | <0.0001 | 1.744 | 38 | 59 | 242 | striated muscle tissue development |
| GO:0006069 | <0.0001 | 15.973 | 1 | 6 | 8 | ethanol oxidation |
| GO:2000352 | <0.0001 | 15.973 | 1 | 6 | 8 | negative regulation of endothelial cell apoptotic process |
| GO:0032535 | <0.0001 | 2.083 | 20 | 36 | 129 | regulation of cellular component size |
| GO:0001934 | <0.0001 | 1.532 | 70 | 97 | 442 | positive regulation of protein phosphorylation |
| GO:0006469 | <0.0001 | 2.221 | 17 | 31 | 106 | negative regulation of protein kinase activity |
| GO:0043069 | <0.0001 | 1.517 | 74 | 101 | 464 | negative regulation of programmed cell death |
| GO:0007517 | <0.0001 | 1.712 | 41 | 62 | 258 | muscle organ development |
| GO:0043299 | <0.0001 | 3.741 | 5 | 14 | 34 | leukocyte degranulation |
| GO:0045428 | <0.0001 | 3.741 | 5 | 14 | 34 | regulation of nitric oxide biosynthetic process |
| GO:0050764 | <0.0001 | 3.741 | 5 | 14 | 34 | regulation of phagocytosis |
| GO:0043066 | <0.0001 | 1.518 | 73 | 100 | 459 | negative regulation of apoptotic process |
| GO:0051090 | <0.0001 | 1.742 | 38 | 58 | 238 | regulation of sequence-specific DNA binding transcription factor activity |
| GO:0045089 | <0.0001 | 2.092 | 20 | 35 | 125 | positive regulation of innate immune response |
| GO:0044765 | <0.0001 | 1.274 | 288 | 335 | 1814 | single-organism transport |
| GO:0002687 | <0.0001 | 2.819 | 9 | 20 | 58 | positive regulation of leukocyte migration |
| GO:0008610 | <0.0001 | 1.585 | 58 | 82 | 363 | lipid biosynthetic process |
| GO:0042981 | <0.0001 | 1.368 | 142 | 178 | 895 | regulation of apoptotic process |
| GO:0001894 | <0.0001 | 2.405 | 13 | 26 | 84 | tissue homeostasis |
| GO:0034394 | <0.0001 | 5.335 | 3 | 10 | 20 | protein localization to cell surface |
| GO:0006461 | <0.0001 | 1.471 | 86 | 115 | 542 | protein complex assembly |
| GO:0033673 | <0.0001 | 2.137 | 18 | 33 | 116 | negative regulation of kinase activity |
| GO:0032990 | <0.0001 | 1.500 | 77 | 104 | 482 | cell part morphogenesis |
| GO:0002673 | <0.0001 | 3.136 | 7 | 17 | 46 | regulation of acute inflammatory response |
| GO:0032091 | <0.0001 | 4.272 | 4 | 12 | 27 | negative regulation of protein binding |
| GO:0060396 | <0.0001 | 4.272 | 4 | 12 | 27 | growth hormone receptor signaling pathway |
| GO:1901698 | <0.0001 | 1.483 | 81 | 109 | 510 | response to nitrogen compound |
| GO:0048878 | <0.0001 | 1.431 | 101 | 132 | 637 | chemical homeostasis |
| GO:0002526 | <0.0001 | 2.337 | 14 | 27 | 89 | acute inflammatory response |
| GO:0048585 | <0.0001 | 1.463 | 87 | 116 | 549 | negative regulation of response to stimulus |
| GO:0033674 | <0.0001 | 1.604 | 53 | 76 | 333 | positive regulation of kinase activity |
| GO:0032870 | <0.0001 | 1.626 | 50 | 72 | 312 | cellular response to hormone stimulus |
| GO:0036296 | <0.0001 | 5.999 | 3 | 9 | 17 | response to increased oxygen levels |
| GO:0055093 | <0.0001 | 5.999 | 3 | 9 | 17 | response to hyperoxia |
| GO:2000516 | <0.0001 | 5.999 | 3 | 9 | 17 | positive regulation of CD4-positive, alpha-beta T cell activation |
| GO:0006067 | <0.0001 | 9.322 | 2 | 7 | 11 | ethanol metabolic process |
| GO:0070271 | <0.0001 | 1.464 | 86 | 115 | 544 | protein complex biogenesis |
| GO:0048771 | <0.0001 | 2.207 | 16 | 30 | 103 | tissue remodeling |
| GO:0030003 | <0.0001 | 1.668 | 44 | 65 | 276 | cellular cation homeostasis |
| GO:0006929 | <0.0001 | 7.106 | 2 | 8 | 14 | substrate-dependent cell migration |
| GO:0042327 | <0.0001 | 1.507 | 72 | 98 | 452 | positive regulation of phosphorylation |
| GO:0051348 | <0.0001 | 2.077 | 19 | 34 | 122 | negative regulation of transferase activity |
| GO:0034142 | 0.001 | 2.516 | 11 | 23 | 72 | toll-like receptor 4 signaling pathway |
| GO:0002221 | 0.001 | 2.224 | 16 | 29 | 99 | pattern recognition receptor signaling pathway |
| GO:0001780 | 0.001 | 26.604 | 1 | 5 | 6 | neutrophil homeostasis |
| GO:0042482 | 0.001 | 26.604 | 1 | 5 | 6 | positive regulation of odontogenesis |
| GO:0050795 | 0.001 | 2.123 | 18 | 32 | 113 | regulation of behavior |
| GO:0002275 | 0.001 | 3.341 | 6 | 15 | 39 | myeloid cell activation involved in immune response |
| GO:0045055 | 0.001 | 3.341 | 6 | 15 | 39 | regulated secretory pathway |
| GO:0046486 | 0.001 | 1.787 | 32 | 50 | 201 | glycerolipid metabolic process |
| GO:0051345 | 0.001 | 1.582 | 54 | 77 | 341 | positive regulation of hydrolase activity |
| GO:0051347 | 0.001 | 1.582 | 54 | 77 | 341 | positive regulation of transferase activity |
| GO:0010243 | 0.001 | 1.479 | 77 | 104 | 487 | response to organic nitrogen |
| GO:0055080 | 0.001 | 1.605 | 50 | 72 | 315 | cation homeostasis |
| GO:0045216 | 0.001 | 2.023 | 20 | 35 | 128 | cell-cell junction organization |
| GO:0048545 | 0.001 | 1.727 | 36 | 55 | 227 | response to steroid hormone stimulus |
| GO:0010742 | 0.001 | 4.004 | 4 | 12 | 28 | macrophage derived foam cell differentiation |
| GO:0030239 | 0.001 | 4.004 | 4 | 12 | 28 | myofibril assembly |
| GO:0045646 | 0.001 | 4.004 | 4 | 12 | 28 | regulation of erythrocyte differentiation |
| GO:0071378 | 0.001 | 4.004 | 4 | 12 | 28 | cellular response to growth hormone stimulus |
| GO:0090077 | 0.001 | 4.004 | 4 | 12 | 28 | foam cell differentiation |
| GO:0050818 | 0.001 | 2.677 | 10 | 20 | 60 | regulation of coagulation |
| GO:0071071 | 0.001 | Inf | 1 | 4 | 4 | regulation of phospholipid biosynthetic process |
| GO:0050853 | 0.001 | 4.849 | 3 | 10 | 21 | B cell receptor signaling pathway |
| GO:0030278 | 0.001 | 2.062 | 19 | 33 | 119 | regulation of ossification |
| GO:0045860 | 0.001 | 1.599 | 50 | 72 | 316 | positive regulation of protein kinase activity |
| GO:0030193 | 0.001 | 2.748 | 9 | 19 | 56 | regulation of blood coagulation |
| GO:0050864 | 0.001 | 2.748 | 9 | 19 | 56 | regulation of B cell activation |
| GO:1900046 | 0.001 | 2.748 | 9 | 19 | 56 | regulation of hemostasis |
| GO:1901888 | 0.001 | 3.654 | 5 | 13 | 32 | regulation of cell junction assembly |
| GO:0001819 | 0.001 | 1.905 | 24 | 40 | 153 | positive regulation of cytokine production |
| GO:0030198 | 0.001 | 1.905 | 24 | 40 | 153 | extracellular matrix organization |
| GO:0043062 | 0.001 | 1.905 | 24 | 40 | 153 | extracellular structure organization |
| GO:0014066 | 0.001 | 2.832 | 8 | 18 | 52 | regulation of phosphatidylinositol 3-kinase cascade |
| GO:0016311 | 0.001 | 1.869 | 26 | 42 | 163 | dephosphorylation |
| GO:0007409 | 0.001 | 1.524 | 63 | 87 | 397 | axonogenesis |
| GO:0045582 | 0.001 | 2.933 | 8 | 17 | 48 | positive regulation of T cell differentiation |
| GO:0048145 | 0.001 | 2.933 | 8 | 17 | 48 | regulation of fibroblast proliferation |
| GO:0046470 | 0.001 | 3.400 | 6 | 14 | 36 | phosphatidylcholine metabolic process |
| GO:0042493 | 0.001 | 1.614 | 47 | 68 | 296 | response to drug |
| GO:0007585 | 0.001 | 3.207 | 6 | 15 | 40 | respiratory gaseous exchange |
| GO:0007249 | 0.001 | 1.951 | 22 | 37 | 139 | I-kappaB kinase/NF-kappaB cascade |
| GO:0035295 | 0.001 | 1.582 | 51 | 73 | 323 | tube development |
| GO:0001945 | 0.001 | 5.331 | 3 | 9 | 18 | lymph vessel development |
| GO:0006691 | 0.001 | 5.331 | 3 | 9 | 18 | leukotriene metabolic process |
| GO:0009620 | 0.001 | 5.331 | 3 | 9 | 18 | response to fungus |
| GO:0006959 | 0.001 | 2.311 | 13 | 25 | 83 | humoral immune response |
| GO:0010741 | 0.001 | 2.119 | 17 | 30 | 106 | negative regulation of intracellular protein kinase cascade |
| GO:0010883 | 0.001 | 4.193 | 4 | 11 | 25 | regulation of lipid storage |
| GO:0060761 | 0.001 | 4.193 | 4 | 11 | 25 | negative regulation of response to cytokine stimulus |
| GO:0043122 | 0.001 | 1.980 | 21 | 35 | 130 | regulation of I-kappaB kinase/NF-kappaB cascade |
| GO:0051056 | 0.001 | 1.689 | 37 | 56 | 235 | regulation of small GTPase mediated signal transduction |
| GO:0030001 | 0.001 | 1.546 | 57 | 79 | 356 | metal ion transport |
| GO:0007229 | 0.001 | 2.676 | 9 | 19 | 57 | integrin-mediated signaling pathway |
| GO:0030213 | 0.001 | 10.647 | 1 | 6 | 9 | hyaluronan biosynthetic process |
| GO:0033008 | 0.001 | 10.647 | 1 | 6 | 9 | positive regulation of mast cell activation involved in immune response |
| GO:0043306 | 0.001 | 10.647 | 1 | 6 | 9 | positive regulation of mast cell degranulation |
| GO:0045628 | 0.001 | 10.647 | 1 | 6 | 9 | regulation of T-helper 2 cell differentiation |
| GO:0042129 | 0.001 | 2.339 | 13 | 24 | 79 | regulation of T cell proliferation |
| GO:0042119 | 0.001 | 6.090 | 2 | 8 | 15 | neutrophil activation |
| GO:0043372 | 0.001 | 6.090 | 2 | 8 | 15 | positive regulation of CD4-positive, alpha-beta T cell differentiation |
| GO:0001952 | 0.001 | 2.751 | 8 | 18 | 53 | regulation of cell-matrix adhesion |
| GO:0043550 | 0.001 | 3.768 | 5 | 12 | 29 | regulation of lipid kinase activity |
| GO:0008063 | 0.001 | 2.499 | 10 | 21 | 66 | Toll signaling pathway |
| GO:0051250 | 0.001 | 2.499 | 10 | 21 | 66 | negative regulation of lymphocyte activation |
| GO:0007399 | 0.001 | 1.296 | 191 | 228 | 1201 | nervous system development |
| GO:0060314 | 0.001 | 7.456 | 2 | 7 | 12 | regulation of ryanodine-sensitive calcium-release channel activity |
| GO:0072577 | 0.001 | 7.456 | 2 | 7 | 12 | endothelial cell apoptotic process |
| GO:0090025 | 0.001 | 7.456 | 2 | 7 | 12 | regulation of monocyte chemotaxis |
| GO:2000351 | 0.001 | 7.456 | 2 | 7 | 12 | regulation of endothelial cell apoptotic process |
| GO:0046677 | 0.001 | 3.471 | 5 | 13 | 33 | response to antibiotic |
| GO:0043407 | 0.001 | 2.949 | 7 | 16 | 45 | negative regulation of MAP kinase activity |
| GO:0006066 | 0.001 | 1.710 | 34 | 52 | 216 | alcohol metabolic process |
| GO:0019915 | 0.001 | 3.251 | 6 | 14 | 37 | lipid storage |
| GO:0002009 | 0.001 | 1.621 | 43 | 63 | 273 | morphogenesis of an epithelium |
| GO:0032602 | 0.001 | 3.083 | 7 | 15 | 41 | chemokine production |
| GO:0044092 | 0.001 | 1.451 | 78 | 104 | 494 | negative regulation of molecular function |
| GO:0043303 | 0.001 | 4.444 | 3 | 10 | 22 | mast cell degranulation |
| GO:0060397 | 0.001 | 4.444 | 3 | 10 | 22 | JAK-STAT cascade involved in growth hormone signaling pathway |
| GO:0031325 | 0.001 | 1.276 | 221 | 260 | 1391 | positive regulation of cellular metabolic process |
| GO:0007568 | 0.001 | 1.855 | 25 | 40 | 156 | aging |
| GO:2000021 | 0.001 | 1.991 | 19 | 33 | 122 | regulation of ion homeostasis |
| GO:0048667 | 0.001 | 1.483 | 68 | 92 | 429 | cell morphogenesis involved in neuron differentiation |
| GO:0022407 | 0.001 | 2.607 | 9 | 19 | 58 | regulation of cell-cell adhesion |
| GO:0050679 | 0.001 | 2.161 | 15 | 27 | 94 | positive regulation of epithelial cell proliferation |
| GO:0045596 | 0.001 | 1.575 | 49 | 69 | 306 | negative regulation of cell differentiation |
| GO:1901699 | 0.001 | 1.575 | 49 | 69 | 306 | cellular response to nitrogen compound |
| GO:0006576 | 0.001 | 2.325 | 12 | 23 | 76 | cellular biogenic amine metabolic process |
| GO:0001569 | 0.001 | 3.912 | 4 | 11 | 26 | patterning of blood vessels |
| GO:0010743 | 0.001 | 3.912 | 4 | 11 | 26 | regulation of macrophage derived foam cell differentiation |
| GO:0022409 | 0.001 | 3.912 | 4 | 11 | 26 | positive regulation of cell-cell adhesion |
| GO:0032233 | 0.001 | 3.912 | 4 | 11 | 26 | positive regulation of actin filament bundle assembly |
| GO:0070098 | 0.001 | 3.912 | 4 | 11 | 26 | chemokine-mediated signaling pathway |
| GO:0008217 | 0.001 | 1.998 | 19 | 32 | 118 | regulation of blood pressure |
| GO:0006873 | 0.001 | 1.460 | 73 | 97 | 458 | cellular ion homeostasis |
| GO:0002224 | 0.001 | 2.178 | 14 | 26 | 90 | toll-like receptor signaling pathway |
| GO:0007163 | 0.001 | 2.178 | 14 | 26 | 90 | establishment or maintenance of cell polarity |
| GO:0030851 | 0.001 | 4.798 | 3 | 9 | 19 | granulocyte differentiation |
| GO:0032321 | 0.001 | 2.851 | 7 | 16 | 46 | positive regulation of Rho GTPase activity |
| GO:0051145 | 0.001 | 3.558 | 5 | 12 | 30 | smooth muscle cell differentiation |
| GO:0048738 | 0.001 | 2.038 | 17 | 30 | 109 | cardiac muscle tissue development |
| GO:0051258 | 0.001 | 2.038 | 17 | 30 | 109 | protein polymerization |
| GO:0097305 | 0.001 | 1.734 | 31 | 47 | 193 | response to alcohol |
| GO:0045576 | 0.001 | 3.305 | 5 | 13 | 34 | mast cell activation |
| GO:0050871 | 0.001 | 3.116 | 6 | 14 | 38 | positive regulation of B cell activation |
| GO:0050801 | 0.001 | 1.431 | 80 | 105 | 504 | ion homeostasis |
| GO:0007259 | 0.001 | 2.196 | 14 | 25 | 86 | JAK-STAT cascade |
| GO:0019932 | 0.001 | 1.975 | 19 | 32 | 119 | second-messenger-mediated signaling |
| GO:0072330 | 0.001 | 1.975 | 19 | 32 | 119 | monocarboxylic acid biosynthetic process |
| GO:0043623 | 0.001 | 1.686 | 34 | 51 | 214 | cellular protein complex assembly |
| GO:0046890 | 0.001 | 2.281 | 12 | 23 | 77 | regulation of lipid biosynthetic process |
| GO:0070665 | 0.001 | 2.281 | 12 | 23 | 77 | positive regulation of leukocyte proliferation |
| GO:0043085 | 0.001 | 1.363 | 110 | 139 | 696 | positive regulation of catalytic activity |
| GO:0006875 | 0.002 | 1.622 | 40 | 58 | 251 | cellular metal ion homeostasis |
| GO:0061448 | 0.002 | 1.858 | 23 | 37 | 144 | connective tissue development |
| GO:0030449 | 0.002 | 5.328 | 3 | 8 | 16 | regulation of complement activation |
| GO:2000257 | 0.002 | 5.328 | 3 | 8 | 16 | regulation of protein activation cascade |
| GO:0002279 | 0.002 | 4.102 | 4 | 10 | 23 | mast cell activation involved in immune response |
| GO:0002448 | 0.002 | 4.102 | 4 | 10 | 23 | mast cell mediated immunity |
| GO:0032722 | 0.002 | 4.102 | 4 | 10 | 23 | positive regulation of chemokine production |
| GO:0010518 | 0.002 | 2.432 | 10 | 20 | 64 | positive regulation of phospholipase activity |
| GO:0043123 | 0.002 | 2.098 | 15 | 27 | 96 | positive regulation of I-kappaB kinase/NF-kappaB cascade |
| GO:0002700 | 0.002 | 2.673 | 8 | 17 | 51 | regulation of production of molecular mediator of immune response |
| GO:0090330 | 0.002 | 13.300 | 1 | 5 | 7 | regulation of platelet aggregation |
| GO:0032386 | 0.002 | 1.692 | 33 | 49 | 205 | regulation of intracellular transport |
| GO:0055065 | 0.002 | 1.587 | 43 | 62 | 273 | metal ion homeostasis |
| GO:0044057 | 0.002 | 1.501 | 58 | 79 | 364 | regulation of system process |
| GO:0002690 | 0.002 | 2.758 | 7 | 16 | 47 | positive regulation of leukocyte chemotaxis |
| GO:0014015 | 0.002 | 3.667 | 4 | 11 | 27 | positive regulation of gliogenesis |
| GO:0046638 | 0.002 | 3.667 | 4 | 11 | 27 | positive regulation of alpha-beta T cell differentiation |
| GO:0071277 | 0.002 | 3.667 | 4 | 11 | 27 | cellular response to calcium ion |
| GO:0007507 | 0.002 | 1.554 | 48 | 67 | 300 | heart development |
| GO:0009118 | 0.002 | 1.778 | 26 | 41 | 165 | regulation of nucleoside metabolic process |
| GO:0033121 | 0.002 | 1.778 | 26 | 41 | 165 | regulation of purine nucleotide catabolic process |
| GO:0060193 | 0.002 | 2.341 | 11 | 21 | 69 | positive regulation of lipase activity |
| GO:0016049 | 0.002 | 1.595 | 42 | 60 | 263 | cell growth |
| GO:0050678 | 0.002 | 1.792 | 25 | 40 | 160 | regulation of epithelial cell proliferation |
| GO:0045598 | 0.002 | 2.862 | 7 | 15 | 43 | regulation of fat cell differentiation |
| GO:0034110 | 0.002 | 6.213 | 2 | 7 | 13 | regulation of homotypic cell-cell adhesion |
| GO:0071276 | 0.002 | 6.213 | 2 | 7 | 13 | cellular response to cadmium ion |
| GO:0060389 | 0.002 | 3.370 | 5 | 12 | 31 | pathway-restricted SMAD protein phosphorylation |
| GO:0019725 | 0.002 | 1.401 | 87 | 112 | 547 | cellular homeostasis |
| GO:0070482 | 0.002 | 1.698 | 31 | 47 | 196 | response to oxygen levels |
| GO:0002283 | 0.002 | 7.984 | 2 | 6 | 10 | neutrophil activation involved in immune response |
| GO:0010934 | 0.002 | 7.984 | 2 | 6 | 10 | macrophage cytokine production |
| GO:0043302 | 0.002 | 7.984 | 2 | 6 | 10 | positive regulation of leukocyte degranulation |
| GO:0097067 | 0.002 | 7.984 | 2 | 6 | 10 | cellular response to thyroid hormone stimulus |
| GO:2000178 | 0.002 | 7.984 | 2 | 6 | 10 | negative regulation of neural precursor cell proliferation |
| GO:0016310 | 0.002 | 1.303 | 152 | 184 | 959 | phosphorylation |
| GO:0060759 | 0.002 | 2.265 | 12 | 22 | 74 | regulation of response to cytokine stimulus |
| GO:0055082 | 0.002 | 1.416 | 79 | 103 | 498 | cellular chemical homeostasis |
| GO:0030811 | 0.002 | 1.764 | 26 | 41 | 166 | regulation of nucleotide catabolic process |
| GO:0002828 | 0.002 | 4.361 | 3 | 9 | 20 | regulation of type 2 immune response |
| GO:0046627 | 0.002 | 4.361 | 3 | 9 | 20 | negative regulation of insulin receptor signaling pathway |
| GO:0032640 | 0.002 | 2.596 | 8 | 17 | 52 | tumor necrosis factor production |
| GO:0032680 | 0.002 | 2.596 | 8 | 17 | 52 | regulation of tumor necrosis factor production |
| GO:0035023 | 0.002 | 1.994 | 17 | 29 | 107 | regulation of Rho protein signal transduction |
| GO:0033043 | 0.002 | 1.493 | 57 | 77 | 356 | regulation of organelle organization |
| GO:0034138 | 0.002 | 2.419 | 10 | 19 | 61 | toll-like receptor 3 signaling pathway |
| GO:0034340 | 0.002 | 2.419 | 10 | 19 | 61 | response to type I interferon |
| GO:0060337 | 0.002 | 2.419 | 10 | 19 | 61 | type I interferon-mediated signaling pathway |
| GO:0071357 | 0.002 | 2.419 | 10 | 19 | 61 | cellular response to type I interferon |
| GO:0071901 | 0.002 | 2.419 | 10 | 19 | 61 | negative regulation of protein serine/threonine kinase activity |
| GO:0031399 | 0.002 | 1.333 | 121 | 150 | 765 | regulation of protein modification process |
| GO:0050673 | 0.002 | 1.705 | 30 | 45 | 187 | epithelial cell proliferation |
| GO:0072507 | 0.002 | 1.645 | 35 | 51 | 218 | divalent inorganic cation homeostasis |
| GO:0051098 | 0.002 | 1.842 | 22 | 35 | 137 | regulation of binding |
| GO:0048729 | 0.002 | 1.497 | 55 | 75 | 346 | tissue morphogenesis |
| GO:0006809 | 0.002 | 2.763 | 7 | 15 | 44 | nitric oxide biosynthetic process |
| GO:0050766 | 0.002 | 3.808 | 4 | 10 | 24 | positive regulation of phagocytosis |
| GO:0032946 | 0.002 | 2.222 | 12 | 22 | 75 | positive regulation of mononuclear cell proliferation |
| GO:0002367 | 0.002 | 2.875 | 6 | 14 | 40 | cytokine production involved in immune response |
| GO:0003158 | 0.002 | 2.875 | 6 | 14 | 40 | endothelium development |
| GO:0071417 | 0.002 | 1.542 | 46 | 64 | 288 | cellular response to organic nitrogen |
| GO:0032663 | 0.002 | 3.201 | 5 | 12 | 32 | regulation of interleukin-2 production |
| GO:0045670 | 0.002 | 3.201 | 5 | 12 | 32 | regulation of osteoclast differentiation |
| GO:0060416 | 0.002 | 3.201 | 5 | 12 | 32 | response to growth hormone stimulus |
| GO:0032271 | 0.002 | 2.160 | 13 | 23 | 80 | regulation of protein polymerization |
| GO:0002262 | 0.002 | 4.735 | 3 | 8 | 17 | myeloid cell homeostasis |
| GO:0003016 | 0.002 | 4.735 | 3 | 8 | 17 | respiratory system process |
| GO:0048872 | 0.003 | 1.807 | 23 | 36 | 143 | homeostasis of number of cells |
| GO:0044085 | 0.003 | 1.277 | 172 | 204 | 1082 | cellular component biogenesis |
| GO:1901654 | 0.003 | 2.363 | 10 | 19 | 62 | response to ketone |
| GO:0001570 | 0.003 | 2.590 | 8 | 16 | 49 | vasculogenesis |
| GO:0071260 | 0.003 | 2.590 | 8 | 16 | 49 | cellular response to mechanical stimulus |
| GO:0031643 | 0.003 | 21.266 | 1 | 4 | 5 | positive regulation of myelination |
| GO:0035791 | 0.003 | 21.266 | 1 | 4 | 5 | platelet-derived growth factor receptor-beta signaling pathway |
| GO:0050855 | 0.003 | 21.266 | 1 | 4 | 5 | regulation of B cell receptor signaling pathway |
| GO:0071493 | 0.003 | 21.266 | 1 | 4 | 5 | cellular response to UV-B |
| GO:0071670 | 0.003 | 21.266 | 1 | 4 | 5 | smooth muscle cell chemotaxis |
| GO:0072223 | 0.003 | 21.266 | 1 | 4 | 5 | metanephric glomerular mesangium development |
| GO:0090197 | 0.003 | 21.266 | 1 | 4 | 5 | positive regulation of chemokine secretion |
| GO:0043409 | 0.003 | 2.180 | 12 | 22 | 76 | negative regulation of MAPK cascade |
| GO:0051495 | 0.003 | 2.180 | 12 | 22 | 76 | positive regulation of cytoskeleton organization |
| GO:0030323 | 0.003 | 1.866 | 20 | 32 | 124 | respiratory tube development |
| GO:0043254 | 0.003 | 1.774 | 24 | 37 | 149 | regulation of protein complex assembly |
| GO:0033124 | 0.003 | 1.790 | 23 | 36 | 144 | regulation of GTP catabolic process |
| GO:0009890 | 0.003 | 1.334 | 111 | 138 | 702 | negative regulation of biosynthetic process |
| GO:0007265 | 0.003 | 1.669 | 30 | 45 | 190 | Ras protein signal transduction |
| GO:0001960 | 0.003 | 3.997 | 3 | 9 | 21 | negative regulation of cytokine-mediated signaling pathway |
| GO:1900077 | 0.003 | 3.997 | 3 | 9 | 21 | negative regulation of cellular response to insulin stimulus |
| GO:1901701 | 0.003 | 1.516 | 48 | 66 | 301 | cellular response to oxygen-containing compound |
| GO:0002507 | 0.003 | 5.324 | 2 | 7 | 14 | tolerance induction |
| GO:0045577 | 0.003 | 5.324 | 2 | 7 | 14 | regulation of B cell differentiation |
| GO:0055003 | 0.003 | 5.324 | 2 | 7 | 14 | cardiac myofibril assembly |
| GO:0060216 | 0.003 | 5.324 | 2 | 7 | 14 | definitive hemopoiesis |
| GO:0045444 | 0.003 | 2.072 | 14 | 24 | 86 | fat cell differentiation |
| GO:0032319 | 0.003 | 2.309 | 10 | 19 | 63 | regulation of Rho GTPase activity |
| GO:0090092 | 0.003 | 1.892 | 18 | 30 | 115 | regulation of transmembrane receptor protein serine/threonine kinase signaling pathway |
| GO:1901652 | 0.003 | 1.521 | 46 | 64 | 291 | response to peptide |
| GO:0032623 | 0.003 | 2.891 | 6 | 13 | 37 | interleukin-2 production |
| GO:0045446 | 0.003 | 2.891 | 6 | 13 | 37 | endothelial cell differentiation |
| GO:2000106 | 0.003 | 2.891 | 6 | 13 | 37 | regulation of leukocyte apoptotic process |
| GO:0032570 | 0.003 | 3.554 | 4 | 10 | 25 | response to progesterone stimulus |
| GO:0045429 | 0.003 | 3.554 | 4 | 10 | 25 | positive regulation of nitric oxide biosynthetic process |
| GO:2000379 | 0.003 | 3.554 | 4 | 10 | 25 | positive regulation of reactive oxygen species metabolic process |
| GO:2000514 | 0.003 | 3.554 | 4 | 10 | 25 | regulation of CD4-positive, alpha-beta T cell activation |
| GO:0030031 | 0.003 | 1.825 | 21 | 33 | 130 | cell projection assembly |
| GO:0060341 | 0.003 | 1.387 | 80 | 103 | 506 | regulation of cellular localization |
| GO:0007266 | 0.003 | 2.140 | 12 | 22 | 77 | Rho protein signal transduction |
| GO:0000122 | 0.003 | 1.460 | 57 | 77 | 362 | negative regulation of transcription from RNA polymerase II promoter |
| GO:0010717 | 0.003 | 3.259 | 5 | 11 | 29 | regulation of epithelial to mesenchymal transition |
| GO:0030098 | 0.003 | 1.666 | 30 | 44 | 186 | lymphocyte differentiation |
| GO:1900542 | 0.003 | 1.563 | 40 | 56 | 249 | regulation of purine nucleotide metabolic process |
| GO:0046209 | 0.003 | 2.514 | 8 | 16 | 50 | nitric oxide metabolic process |
| GO:0046849 | 0.003 | 2.514 | 8 | 16 | 50 | bone remodeling |
| GO:0072503 | 0.003 | 1.624 | 33 | 48 | 207 | cellular divalent inorganic cation homeostasis |
| GO:0035666 | 0.004 | 2.346 | 9 | 18 | 59 | TRIF-dependent toll-like receptor signaling pathway |
| GO:0006837 | 0.004 | 6.386 | 2 | 6 | 11 | serotonin transport |
| GO:0007567 | 0.004 | 6.386 | 2 | 6 | 11 | parturition |
| GO:0033005 | 0.004 | 6.386 | 2 | 6 | 11 | positive regulation of mast cell activation |
| GO:0036151 | 0.004 | 6.386 | 2 | 6 | 11 | phosphatidylcholine acyl-chain remodeling |
| GO:0071470 | 0.004 | 6.386 | 2 | 6 | 11 | cellular response to osmotic stress |
| GO:0043410 | 0.004 | 1.572 | 38 | 54 | 239 | positive regulation of MAPK cascade |
| GO:0090287 | 0.004 | 1.895 | 18 | 29 | 111 | regulation of cellular response to growth factor stimulus |
| GO:0055001 | 0.004 | 1.870 | 18 | 30 | 116 | muscle cell development |
| GO:0031579 | 0.004 | 8.865 | 1 | 5 | 8 | membrane raft organization |
| GO:0043129 | 0.004 | 8.865 | 1 | 5 | 8 | surfactant homeostasis |
| GO:0045059 | 0.004 | 8.865 | 1 | 5 | 8 | positive thymic T cell selection |
| GO:0085029 | 0.004 | 8.865 | 1 | 5 | 8 | extracellular matrix assembly |
| GO:0034333 | 0.004 | 2.584 | 7 | 15 | 46 | adherens junction assembly |
| GO:2000377 | 0.004 | 2.584 | 7 | 15 | 46 | regulation of reactive oxygen species metabolic process |
| GO:0045926 | 0.004 | 1.756 | 23 | 36 | 146 | negative regulation of growth |
| GO:0051607 | 0.004 | 1.806 | 21 | 33 | 131 | defense response to virus |
| GO:0051091 | 0.004 | 1.788 | 22 | 34 | 136 | positive regulation of sequence-specific DNA binding transcription factor activity |
| GO:0031327 | 0.004 | 1.324 | 110 | 135 | 690 | negative regulation of cellular biosynthetic process |
| GO:0071706 | 0.004 | 2.390 | 9 | 17 | 55 | tumor necrosis factor superfamily cytokine production |
| GO:0001885 | 0.004 | 4.261 | 3 | 8 | 18 | endothelial cell development |
| GO:0032633 | 0.004 | 4.261 | 3 | 8 | 18 | interleukin-4 production |
| GO:0070527 | 0.004 | 4.261 | 3 | 8 | 18 | platelet aggregation |
| GO:0002456 | 0.004 | 2.669 | 7 | 14 | 42 | T cell mediated immunity |
| GO:0002513 | 0.004 | Inf | 0 | 3 | 3 | tolerance induction to self antigen |
| GO:0014722 | 0.004 | Inf | 0 | 3 | 3 | regulation of skeletal muscle contraction by calcium ion signaling |
| GO:0032632 | 0.004 | Inf | 0 | 3 | 3 | interleukin-3 production |
| GO:0035793 | 0.004 | Inf | 0 | 3 | 3 | positive regulation of metanephric mesenchymal cell migration by platelet-derived growth factor receptor-beta signaling pathway |
| GO:0036015 | 0.004 | Inf | 0 | 3 | 3 | response to interleukin-3 |
| GO:0036016 | 0.004 | Inf | 0 | 3 | 3 | cellular response to interleukin-3 |
| GO:0046813 | 0.004 | Inf | 0 | 3 | 3 | virion attachment, binding of host cell surface receptor |
| GO:0071072 | 0.004 | Inf | 0 | 3 | 3 | negative regulation of phospholipid biosynthetic process |
| GO:0072275 | 0.004 | Inf | 0 | 3 | 3 | metanephric glomerulus morphogenesis |
| GO:0072276 | 0.004 | Inf | 0 | 3 | 3 | metanephric glomerulus vasculature morphogenesis |
| GO:0072277 | 0.004 | Inf | 0 | 3 | 3 | metanephric glomerular capillary formation |
| GO:0097242 | 0.004 | Inf | 0 | 3 | 3 | beta-amyloid clearance |
| GO:1900238 | 0.004 | Inf | 0 | 3 | 3 | regulation of metanephric mesenchymal cell migration by platelet-derived growth factor receptor-beta signaling pathway |
| GO:2000591 | 0.004 | Inf | 0 | 3 | 3 | positive regulation of metanephric mesenchymal cell migration |
| GO:0006140 | 0.004 | 1.547 | 40 | 56 | 251 | regulation of nucleotide metabolic process |
| GO:0003008 | 0.004 | 1.249 | 190 | 222 | 1199 | system process |
| GO:0001959 | 0.004 | 2.182 | 11 | 20 | 69 | regulation of cytokine-mediated signaling pathway |
| GO:0019722 | 0.004 | 2.182 | 11 | 20 | 69 | calcium-mediated signaling |
| GO:0043087 | 0.004 | 1.755 | 23 | 35 | 142 | regulation of GTPase activity |
| GO:0030324 | 0.004 | 1.826 | 19 | 31 | 122 | lung development |
| GO:0060485 | 0.004 | 1.826 | 19 | 31 | 122 | mesenchyme development |
| GO:0002040 | 0.004 | 2.775 | 6 | 13 | 38 | sprouting angiogenesis |
| GO:0032642 | 0.004 | 2.775 | 6 | 13 | 38 | regulation of chemokine production |
| GO:0035690 | 0.004 | 2.775 | 6 | 13 | 38 | cellular response to drug |
| GO:0046635 | 0.004 | 2.775 | 6 | 13 | 38 | positive regulation of alpha-beta T cell activation |
| GO:0010604 | 0.004 | 1.235 | 215 | 248 | 1354 | positive regulation of macromolecule metabolic process |
| GO:0002756 | 0.004 | 2.290 | 10 | 18 | 60 | MyD88-independent toll-like receptor signaling pathway |
| GO:0051496 | 0.004 | 3.689 | 3 | 9 | 22 | positive regulation of stress fiber assembly |
| GO:0002718 | 0.004 | 2.909 | 5 | 12 | 34 | regulation of cytokine production involved in immune response |
| GO:0045453 | 0.004 | 2.909 | 5 | 12 | 34 | bone resorption |
| GO:0048844 | 0.004 | 2.909 | 5 | 12 | 34 | artery morphogenesis |
| GO:0050671 | 0.004 | 2.119 | 12 | 21 | 74 | positive regulation of lymphocyte proliferation |
| GO:0006937 | 0.004 | 1.968 | 15 | 25 | 93 | regulation of muscle contraction |
| GO:0051055 | 0.005 | 3.331 | 4 | 10 | 26 | negative regulation of lipid biosynthetic process |
| GO:0048146 | 0.005 | 3.087 | 5 | 11 | 30 | positive regulation of fibroblast proliferation |
| GO:0051279 | 0.005 | 3.087 | 5 | 11 | 30 | regulation of release of sequestered calcium ion into cytosol |
| GO:0072210 | 0.005 | 3.087 | 5 | 11 | 30 | metanephric nephron development |
| GO:0015918 | 0.005 | 2.503 | 7 | 15 | 47 | sterol transport |
| GO:0030301 | 0.005 | 2.503 | 7 | 15 | 47 | cholesterol transport |
| GO:0042035 | 0.005 | 2.208 | 10 | 19 | 65 | regulation of cytokine biosynthetic process |
| GO:0034101 | 0.005 | 2.064 | 13 | 22 | 79 | erythrocyte homeostasis |
| GO:0042063 | 0.005 | 1.787 | 20 | 32 | 128 | gliogenesis |
| GO:0006357 | 0.005 | 1.278 | 141 | 169 | 891 | regulation of transcription from RNA polymerase II promoter |
| GO:0006810 | 0.005 | 1.195 | 341 | 379 | 2148 | transport |
| GO:0006654 | 0.005 | 4.658 | 2 | 7 | 15 | phosphatidic acid biosynthetic process |
| GO:0032673 | 0.005 | 4.658 | 2 | 7 | 15 | regulation of interleukin-4 production |
| GO:0046473 | 0.005 | 4.658 | 2 | 7 | 15 | phosphatidic acid metabolic process |
| GO:0048745 | 0.005 | 4.658 | 2 | 7 | 15 | smooth muscle tissue development |
| GO:0050777 | 0.005 | 2.576 | 7 | 14 | 43 | negative regulation of immune response |
| GO:0001558 | 0.005 | 1.583 | 33 | 48 | 211 | regulation of cell growth |
| GO:0042692 | 0.005 | 1.559 | 36 | 51 | 227 | muscle cell differentiation |
| GO:0051247 | 0.005 | 1.316 | 106 | 130 | 667 | positive regulation of protein metabolic process |
| GO:0090100 | 0.005 | 2.373 | 8 | 16 | 52 | positive regulation of transmembrane receptor protein serine/threonine kinase signaling pathway |
| GO:0051234 | 0.005 | 1.192 | 346 | 384 | 2181 | establishment of localization |
| GO:0043547 | 0.005 | 1.878 | 17 | 27 | 104 | positive regulation of GTPase activity |
| GO:0001909 | 0.005 | 2.667 | 6 | 13 | 39 | leukocyte mediated cytotoxicity |
| GO:0045833 | 0.005 | 2.667 | 6 | 13 | 39 | negative regulation of lipid metabolic process |
| GO:1901615 | 0.006 | 1.496 | 44 | 60 | 276 | organic hydroxy compound metabolic process |
| GO:0045580 | 0.006 | 2.160 | 10 | 19 | 66 | regulation of T cell differentiation |
| GO:0046637 | 0.006 | 2.782 | 6 | 12 | 35 | regulation of alpha-beta T cell differentiation |
| GO:0021772 | 0.006 | 3.873 | 3 | 8 | 19 | olfactory bulb development |
| GO:0046632 | 0.006 | 2.270 | 9 | 17 | 57 | alpha-beta T cell differentiation |
| GO:0002762 | 0.006 | 3.425 | 4 | 9 | 23 | negative regulation of myeloid leukocyte differentiation |
| GO:0043370 | 0.006 | 3.425 | 4 | 9 | 23 | regulation of CD4-positive, alpha-beta T cell differentiation |
| GO:0090218 | 0.006 | 3.425 | 4 | 9 | 23 | positive regulation of lipid kinase activity |
| GO:1901019 | 0.006 | 3.425 | 4 | 9 | 23 | regulation of calcium ion transmembrane transporter activity |
| GO:0002686 | 0.006 | 5.321 | 2 | 6 | 12 | negative regulation of leukocyte migration |
| GO:0006957 | 0.006 | 5.321 | 2 | 6 | 12 | complement activation, alternative pathway |
| GO:0010922 | 0.006 | 5.321 | 2 | 6 | 12 | positive regulation of phosphatase activity |
| GO:0033137 | 0.006 | 5.321 | 2 | 6 | 12 | negative regulation of peptidyl-serine phosphorylation |
| GO:0050832 | 0.006 | 5.321 | 2 | 6 | 12 | defense response to fungus |
| GO:1901020 | 0.006 | 5.321 | 2 | 6 | 12 | negative regulation of calcium ion transmembrane transporter activity |
| GO:2000278 | 0.006 | 5.321 | 2 | 6 | 12 | regulation of DNA biosynthetic process |
| GO:0034446 | 0.006 | 3.135 | 4 | 10 | 27 | substrate adhesion-dependent cell spreading |
| GO:0060393 | 0.006 | 3.135 | 4 | 10 | 27 | regulation of pathway-restricted SMAD protein phosphorylation |
| GO:0030509 | 0.006 | 2.041 | 12 | 21 | 76 | BMP signaling pathway |
| GO:0055074 | 0.006 | 1.569 | 33 | 47 | 208 | calcium ion homeostasis |
| GO:0002698 | 0.006 | 2.490 | 7 | 14 | 44 | negative regulation of immune effector process |
| GO:0002440 | 0.006 | 1.994 | 13 | 22 | 81 | production of molecular mediator of immune response |
| GO:0045639 | 0.006 | 2.309 | 8 | 16 | 53 | positive regulation of myeloid cell differentiation |
| GO:0010876 | 0.006 | 1.668 | 25 | 37 | 156 | lipid localization |
| GO:0014074 | 0.007 | 1.953 | 14 | 23 | 86 | response to purine-containing compound |
| GO:0009615 | 0.007 | 1.580 | 31 | 45 | 198 | response to virus |
| GO:0014065 | 0.007 | 2.115 | 11 | 19 | 67 | phosphatidylinositol 3-kinase cascade |
| GO:0035303 | 0.007 | 2.115 | 11 | 19 | 67 | regulation of dephosphorylation |
| GO:0045017 | 0.007 | 1.703 | 22 | 34 | 141 | glycerolipid biosynthetic process |
| GO:0009892 | 0.007 | 1.249 | 161 | 189 | 1016 | negative regulation of metabolic process |
| GO:0055002 | 0.007 | 1.884 | 15 | 25 | 96 | striated muscle cell development |
| GO:0050731 | 0.007 | 1.855 | 16 | 26 | 101 | positive regulation of peptidyl-tyrosine phosphorylation |
| GO:0043534 | 0.007 | 2.355 | 8 | 15 | 49 | blood vessel endothelial cell migration |
| GO:0006907 | 0.007 | 6.648 | 1 | 5 | 9 | pinocytosis |
| GO:0010884 | 0.007 | 6.648 | 1 | 5 | 9 | positive regulation of lipid storage |
| GO:0010935 | 0.007 | 6.648 | 1 | 5 | 9 | regulation of macrophage cytokine production |
| GO:0032753 | 0.007 | 6.648 | 1 | 5 | 9 | positive regulation of interleukin-4 production |
| GO:0043368 | 0.007 | 6.648 | 1 | 5 | 9 | positive T cell selection |
| GO:0045579 | 0.007 | 6.648 | 1 | 5 | 9 | positive regulation of B cell differentiation |
| GO:0048268 | 0.007 | 6.648 | 1 | 5 | 9 | clathrin coat assembly |
| GO:0072109 | 0.007 | 6.648 | 1 | 5 | 9 | glomerular mesangium development |
| GO:0090026 | 0.007 | 6.648 | 1 | 5 | 9 | positive regulation of monocyte chemotaxis |
| GO:0032825 | 0.007 | 10.631 | 1 | 4 | 6 | positive regulation of natural killer cell differentiation |
| GO:0033622 | 0.007 | 10.631 | 1 | 4 | 6 | integrin activation |
| GO:0033623 | 0.007 | 10.631 | 1 | 4 | 6 | regulation of integrin activation |
| GO:0045630 | 0.007 | 10.631 | 1 | 4 | 6 | positive regulation of T-helper 2 cell differentiation |
| GO:0045647 | 0.007 | 10.631 | 1 | 4 | 6 | negative regulation of erythrocyte differentiation |
| GO:0051497 | 0.007 | 10.631 | 1 | 4 | 6 | negative regulation of stress fiber assembly |
| GO:0060426 | 0.007 | 10.631 | 1 | 4 | 6 | lung vasculature development |
| GO:0061438 | 0.007 | 10.631 | 1 | 4 | 6 | renal system vasculature morphogenesis |
| GO:0061439 | 0.007 | 10.631 | 1 | 4 | 6 | kidney vasculature morphogenesis |
| GO:0071732 | 0.007 | 10.631 | 1 | 4 | 6 | cellular response to nitric oxide |
| GO:0090195 | 0.007 | 10.631 | 1 | 4 | 6 | chemokine secretion |
| GO:0090196 | 0.007 | 10.631 | 1 | 4 | 6 | regulation of chemokine secretion |
| GO:0043434 | 0.007 | 1.466 | 45 | 61 | 285 | response to peptide hormone stimulus |
| GO:0009891 | 0.008 | 1.254 | 150 | 176 | 942 | positive regulation of biosynthetic process |
| GO:0051480 | 0.008 | 1.687 | 23 | 34 | 142 | cytosolic calcium ion homeostasis |
| GO:0001501 | 0.008 | 1.468 | 44 | 60 | 280 | skeletal system development |
| GO:0006998 | 0.008 | 4.140 | 3 | 7 | 16 | nuclear envelope organization |
| GO:0034308 | 0.008 | 4.140 | 3 | 7 | 16 | primary alcohol metabolic process |
| GO:0052646 | 0.008 | 4.140 | 3 | 7 | 16 | alditol phosphate metabolic process |
| GO:0043491 | 0.008 | 1.922 | 14 | 23 | 87 | protein kinase B signaling cascade |
| GO:0007044 | 0.008 | 2.248 | 9 | 16 | 54 | cell-substrate junction assembly |
| GO:0031295 | 0.008 | 2.248 | 9 | 16 | 54 | T cell costimulation |
| GO:0042102 | 0.008 | 2.248 | 9 | 16 | 54 | positive regulation of T cell proliferation |
| GO:0006811 | 0.008 | 1.295 | 107 | 130 | 675 | ion transport |
| GO:0071774 | 0.008 | 1.730 | 20 | 31 | 127 | response to fibroblast growth factor stimulus |
| GO:0007566 | 0.008 | 2.792 | 5 | 11 | 32 | embryo implantation |
| GO:0048008 | 0.008 | 2.792 | 5 | 11 | 32 | platelet-derived growth factor receptor signaling pathway |
| GO:0072593 | 0.008 | 1.858 | 15 | 25 | 97 | reactive oxygen species metabolic process |
| GO:0006470 | 0.008 | 1.831 | 16 | 26 | 102 | protein dephosphorylation |
| GO:0046474 | 0.008 | 1.764 | 19 | 29 | 117 | glycerophospholipid biosynthetic process |
| GO:0090257 | 0.008 | 1.806 | 17 | 27 | 107 | regulation of muscle system process |
| GO:0000302 | 0.008 | 1.784 | 18 | 28 | 112 | response to reactive oxygen species |
| GO:0001503 | 0.008 | 1.535 | 34 | 48 | 216 | ossification |
| GO:0051641 | 0.008 | 1.211 | 221 | 252 | 1395 | cellular localization |
| GO:0030218 | 0.008 | 2.016 | 12 | 20 | 73 | erythrocyte differentiation |
| GO:0043393 | 0.008 | 2.016 | 12 | 20 | 73 | regulation of protein binding |
| GO:0051592 | 0.008 | 2.016 | 12 | 20 | 73 | response to calcium ion |
| GO:0030837 | 0.008 | 2.960 | 4 | 10 | 28 | negative regulation of actin filament polymerization |
| GO:0002702 | 0.008 | 3.550 | 3 | 8 | 20 | positive regulation of production of molecular mediator of immune response |
| GO:0003081 | 0.008 | 3.550 | 3 | 8 | 20 | regulation of systemic arterial blood pressure by renin-angiotensin |
| GO:0006692 | 0.008 | 3.550 | 3 | 8 | 20 | prostanoid metabolic process |
| GO:0006693 | 0.008 | 3.550 | 3 | 8 | 20 | prostaglandin metabolic process |
| GO:0010596 | 0.008 | 3.550 | 3 | 8 | 20 | negative regulation of endothelial cell migration |
| GO:0021988 | 0.008 | 3.550 | 3 | 8 | 20 | olfactory lobe development |
| GO:0032720 | 0.008 | 3.550 | 3 | 8 | 20 | negative regulation of tumor necrosis factor production |
| GO:0002706 | 0.008 | 2.161 | 9 | 17 | 59 | regulation of lymphocyte mediated immunity |
| GO:0002920 | 0.008 | 3.196 | 4 | 9 | 24 | regulation of humoral immune response |
| GO:0032755 | 0.008 | 3.196 | 4 | 9 | 24 | positive regulation of interleukin-6 production |
| GO:0043551 | 0.008 | 3.196 | 4 | 9 | 24 | regulation of phosphatidylinositol 3-kinase activity |
| GO:0019222 | 0.009 | 1.165 | 469 | 507 | 2955 | regulation of metabolic process |
| GO:0060840 | 0.009 | 2.476 | 7 | 13 | 41 | artery development |
| GO:0006631 | 0.009 | 1.576 | 29 | 42 | 185 | fatty acid metabolic process |
| GO:0036293 | 0.009 | 1.576 | 29 | 42 | 185 | response to decreased oxygen levels |
| GO:0046634 | 0.009 | 2.287 | 8 | 15 | 50 | regulation of alpha-beta T cell activation |
| GO:0008654 | 0.009 | 1.697 | 21 | 32 | 133 | phospholipid biosynthetic process |
| GO:0048469 | 0.009 | 1.892 | 14 | 23 | 88 | cell maturation |
| GO:0001656 | 0.009 | 2.090 | 10 | 18 | 64 | metanephros development |
| GO:0006023 | 0.009 | 2.090 | 10 | 18 | 64 | aminoglycan biosynthetic process |
| GO:0006024 | 0.009 | 2.090 | 10 | 18 | 64 | glycosaminoglycan biosynthetic process |
| GO:0061024 | 0.009 | 1.446 | 46 | 61 | 288 | membrane organization |
| GO:0045766 | 0.009 | 2.030 | 11 | 19 | 69 | positive regulation of angiogenesis |
| GO:0035710 | 0.009 | 2.559 | 6 | 12 | 37 | CD4-positive, alpha-beta T cell activation |
| GO:0031294 | 0.009 | 2.190 | 9 | 16 | 55 | lymphocyte costimulation |
| GO:0055007 | 0.009 | 2.190 | 9 | 16 | 55 | cardiac muscle cell differentiation |
| GO:0032268 | 0.010 | 1.246 | 147 | 172 | 924 | regulation of cellular protein metabolic process |
| GO:0016044 | 0.010 | 1.448 | 45 | 60 | 283 | cellular membrane organization |
| GO:0042089 | 0.010 | 1.979 | 12 | 20 | 74 | cytokine biosynthetic process |
| GO:0046631 | 0.010 | 1.979 | 12 | 20 | 74 | alpha-beta T cell activation |
| GO:0045666 | 0.010 | 2.334 | 7 | 14 | 46 | positive regulation of neuron differentiation |
| GO:0045669 | 0.010 | 2.334 | 7 | 14 | 46 | positive regulation of osteoblast differentiation |
| GO:0007204 | 0.010 | 1.680 | 21 | 32 | 134 | elevation of cytosolic calcium ion concentration |
| GO:0071900 | 0.010 | 1.460 | 42 | 57 | 267 | regulation of protein serine/threonine kinase activity |
| GO:0001991 | 0.010 | 4.560 | 2 | 6 | 13 | regulation of systemic arterial blood pressure by circulatory renin-angiotensin |
| GO:0010878 | 0.010 | 4.560 | 2 | 6 | 13 | cholesterol storage |
| GO:0010885 | 0.010 | 4.560 | 2 | 6 | 13 | regulation of cholesterol storage |
| GO:0045064 | 0.010 | 4.560 | 2 | 6 | 13 | T-helper 2 cell differentiation |
| GO:0045737 | 0.010 | 4.560 | 2 | 6 | 13 | positive regulation of cyclin-dependent protein kinase activity |
| GO:0071354 | 0.010 | 4.560 | 2 | 6 | 13 | cellular response to interleukin-6 |
| GO:0097066 | 0.010 | 4.560 | 2 | 6 | 13 | response to thyroid hormone stimulus |
| GO:0048754 | 0.010 | 1.709 | 20 | 30 | 124 | branching morphogenesis of an epithelial tube |
| GO:0009790 | 0.010 | 1.291 | 101 | 122 | 634 | embryo development |
| GO:0031663 | 0.010 | 2.665 | 5 | 11 | 33 | lipopolysaccharide-mediated signaling pathway |
| GO:0060425 | 0.010 | 2.665 | 5 | 11 | 33 | lung morphogenesis |
| GO:0043933 | 0.010 | 1.250 | 138 | 162 | 867 | macromolecular complex subunit organization |
| GO:0003018 | 0.010 | 1.742 | 18 | 28 | 114 | vascular process in circulatory system |
| GO:0051051 | 0.010 | 1.507 | 35 | 48 | 219 | negative regulation of transport |
| GO:0006796 | 0.011 | 1.187 | 272 | 304 | 1716 | phosphate-containing compound metabolic process |
| GO:0051209 | 0.011 | 2.223 | 8 | 15 | 51 | release of sequestered calcium ion into cytosol |
| GO:0051282 | 0.011 | 2.223 | 8 | 15 | 51 | regulation of sequestering of calcium ion |
| GO:0051283 | 0.011 | 2.223 | 8 | 15 | 51 | negative regulation of sequestering of calcium ion |
| GO:1901616 | 0.011 | 2.390 | 7 | 13 | 42 | organic hydroxy compound catabolic process |
| GO:0031328 | 0.011 | 1.241 | 147 | 172 | 927 | positive regulation of cellular biosynthetic process |
| GO:0006874 | 0.011 | 1.533 | 31 | 44 | 198 | cellular calcium ion homeostasis |
| GO:0014909 | 0.011 | 2.804 | 5 | 10 | 29 | smooth muscle cell migration |
| GO:0002720 | 0.011 | 3.725 | 3 | 7 | 17 | positive regulation of cytokine production involved in immune response |
| GO:0043277 | 0.011 | 3.725 | 3 | 7 | 17 | apoptotic cell clearance |
| GO:0045600 | 0.011 | 3.725 | 3 | 7 | 17 | positive regulation of fat cell differentiation |
| GO:0070741 | 0.011 | 3.725 | 3 | 7 | 17 | response to interleukin-6 |
| GO:2000249 | 0.011 | 3.725 | 3 | 7 | 17 | regulation of actin cytoskeleton reorganization |
| GO:0019724 | 0.011 | 1.942 | 12 | 20 | 75 | B cell mediated immunity |
| GO:0034332 | 0.011 | 1.942 | 12 | 20 | 75 | adherens junction organization |
| GO:0001837 | 0.011 | 2.135 | 9 | 16 | 56 | epithelial to mesenchymal transition |
| GO:0014910 | 0.011 | 2.996 | 4 | 9 | 25 | regulation of smooth muscle cell migration |
| GO:0030834 | 0.011 | 2.996 | 4 | 9 | 25 | regulation of actin filament depolymerization |
| GO:0051893 | 0.011 | 2.996 | 4 | 9 | 25 | regulation of focal adhesion assembly |
| GO:0090109 | 0.011 | 2.996 | 4 | 9 | 25 | regulation of cell-substrate junction assembly |
| GO:2000107 | 0.011 | 2.996 | 4 | 9 | 25 | negative regulation of leukocyte apoptotic process |
| GO:0034765 | 0.011 | 1.902 | 13 | 21 | 80 | regulation of ion transmembrane transport |
| GO:0043405 | 0.012 | 1.543 | 30 | 42 | 188 | regulation of MAP kinase activity |
| GO:0034405 | 0.012 | 3.276 | 3 | 8 | 21 | response to fluid shear stress |
| GO:0051058 | 0.012 | 3.276 | 3 | 8 | 21 | negative regulation of small GTPase mediated signal transduction |
| GO:0060445 | 0.012 | 3.276 | 3 | 8 | 21 | branching involved in salivary gland morphogenesis |
| GO:0046488 | 0.012 | 1.835 | 14 | 23 | 90 | phosphatidylinositol metabolic process |
| GO:0001818 | 0.012 | 1.807 | 15 | 24 | 95 | negative regulation of cytokine production |
| GO:0045787 | 0.012 | 1.807 | 15 | 24 | 95 | positive regulation of cell cycle |
| GO:0045944 | 0.012 | 1.313 | 83 | 102 | 522 | positive regulation of transcription from RNA polymerase II promoter |
| GO:0006812 | 0.012 | 1.338 | 71 | 89 | 448 | cation transport |
| GO:0046164 | 0.012 | 2.460 | 6 | 12 | 38 | alcohol catabolic process |
| GO:0061035 | 0.012 | 2.460 | 6 | 12 | 38 | regulation of cartilage development |
| GO:0097006 | 0.012 | 2.460 | 6 | 12 | 38 | regulation of plasma lipoprotein particle levels |
| GO:0051928 | 0.012 | 2.263 | 7 | 14 | 47 | positive regulation of calcium ion transport |
| GO:0051253 | 0.012 | 1.295 | 92 | 112 | 580 | negative regulation of RNA metabolic process |
| GO:0010629 | 0.012 | 1.287 | 97 | 117 | 609 | negative regulation of gene expression |
| GO:0031098 | 0.012 | 1.594 | 25 | 36 | 157 | stress-activated protein kinase signaling cascade |
| GO:0010719 | 0.013 | 5.317 | 2 | 5 | 10 | negative regulation of epithelial to mesenchymal transition |
| GO:0010881 | 0.013 | 5.317 | 2 | 5 | 10 | regulation of cardiac muscle contraction by regulation of the release of sequestered calcium ion |
| GO:0035307 | 0.013 | 5.317 | 2 | 5 | 10 | positive regulation of protein dephosphorylation |
| GO:0036152 | 0.013 | 5.317 | 2 | 5 | 10 | phosphatidylethanolamine acyl-chain remodeling |
| GO:0048875 | 0.013 | 5.317 | 2 | 5 | 10 | chemical homeostasis within a tissue |
| GO:0051894 | 0.013 | 5.317 | 2 | 5 | 10 | positive regulation of focal adhesion assembly |
| GO:0060347 | 0.013 | 5.317 | 2 | 5 | 10 | heart trabecula formation |
| GO:0071675 | 0.013 | 5.317 | 2 | 5 | 10 | regulation of mononuclear cell migration |
| GO:1901890 | 0.013 | 5.317 | 2 | 5 | 10 | positive regulation of cell junction assembly |
| GO:0044344 | 0.013 | 1.672 | 20 | 30 | 126 | cellular response to fibroblast growth factor stimulus |
| GO:0001935 | 0.013 | 1.951 | 11 | 19 | 71 | endothelial cell proliferation |
| GO:0031324 | 0.013 | 1.231 | 151 | 175 | 949 | negative regulation of cellular metabolic process |
| GO:0051047 | 0.013 | 1.567 | 27 | 38 | 168 | positive regulation of secretion |
| GO:0052547 | 0.013 | 1.513 | 32 | 44 | 200 | regulation of peptidase activity |
| GO:0030888 | 0.013 | 2.549 | 5 | 11 | 34 | regulation of B cell proliferation |
| GO:0001666 | 0.013 | 1.537 | 29 | 41 | 184 | response to hypoxia |
| GO:0042107 | 0.013 | 1.907 | 12 | 20 | 76 | cytokine metabolic process |
| GO:0050852 | 0.013 | 1.907 | 12 | 20 | 76 | T cell receptor signaling pathway |
| GO:0042100 | 0.013 | 2.310 | 7 | 13 | 43 | B cell proliferation |
| GO:0042306 | 0.013 | 1.759 | 16 | 25 | 101 | regulation of protein import into nucleus |
| GO:0045892 | 0.014 | 1.295 | 89 | 108 | 559 | negative regulation of transcription, DNA-dependent |
| GO:0001701 | 0.014 | 1.454 | 39 | 52 | 244 | in utero embryonic development |
| GO:0051346 | 0.014 | 1.548 | 28 | 39 | 174 | negative regulation of hydrolase activity |
| GO:0002763 | 0.014 | 2.663 | 5 | 10 | 30 | positive regulation of myeloid leukocyte differentiation |
| GO:0055013 | 0.014 | 2.663 | 5 | 10 | 30 | cardiac muscle cell development |
| GO:0002765 | 0.014 | 15.936 | 1 | 3 | 4 | immune response-inhibiting signal transduction |
| GO:0002767 | 0.014 | 15.936 | 1 | 3 | 4 | immune response-inhibiting cell surface receptor signaling pathway |
| GO:0002866 | 0.014 | 15.936 | 1 | 3 | 4 | positive regulation of acute inflammatory response to antigenic stimulus |
| GO:0006883 | 0.014 | 15.936 | 1 | 3 | 4 | cellular sodium ion homeostasis |
| GO:0010572 | 0.014 | 15.936 | 1 | 3 | 4 | positive regulation of platelet activation |
| GO:0010713 | 0.014 | 15.936 | 1 | 3 | 4 | negative regulation of collagen metabolic process |
| GO:0032754 | 0.014 | 15.936 | 1 | 3 | 4 | positive regulation of interleukin-5 production |
| GO:0032763 | 0.014 | 15.936 | 1 | 3 | 4 | regulation of mast cell cytokine production |
| GO:0035701 | 0.014 | 15.936 | 1 | 3 | 4 | hematopoietic stem cell migration |
| GO:0035787 | 0.014 | 15.936 | 1 | 3 | 4 | cell migration involved in kidney development |
| GO:0035788 | 0.014 | 15.936 | 1 | 3 | 4 | cell migration involved in metanephros development |
| GO:0035789 | 0.014 | 15.936 | 1 | 3 | 4 | metanephric mesenchymal cell migration |
| GO:0036120 | 0.014 | 15.936 | 1 | 3 | 4 | cellular response to platelet-derived growth factor stimulus |
| GO:0044252 | 0.014 | 15.936 | 1 | 3 | 4 | negative regulation of multicellular organismal metabolic process |
| GO:0044539 | 0.014 | 15.936 | 1 | 3 | 4 | long-chain fatty acid import |
| GO:0046449 | 0.014 | 15.936 | 1 | 3 | 4 | creatinine metabolic process |
| GO:0060044 | 0.014 | 15.936 | 1 | 3 | 4 | negative regulation of cardiac muscle cell proliferation |
| GO:0060137 | 0.014 | 15.936 | 1 | 3 | 4 | maternal process involved in parturition |
| GO:0060696 | 0.014 | 15.936 | 1 | 3 | 4 | regulation of phospholipid catabolic process |
| GO:0060916 | 0.014 | 15.936 | 1 | 3 | 4 | mesenchymal cell proliferation involved in lung development |
| GO:0061014 | 0.014 | 15.936 | 1 | 3 | 4 | positive regulation of mRNA catabolic process |
| GO:0070100 | 0.014 | 15.936 | 1 | 3 | 4 | negative regulation of chemokine-mediated signaling pathway |
| GO:0070431 | 0.014 | 15.936 | 1 | 3 | 4 | nucleotide-binding oligomerization domain containing 2 signaling pathway |
| GO:0072007 | 0.014 | 15.936 | 1 | 3 | 4 | mesangial cell differentiation |
| GO:0072008 | 0.014 | 15.936 | 1 | 3 | 4 | glomerular mesangial cell differentiation |
| GO:0072011 | 0.014 | 15.936 | 1 | 3 | 4 | glomerular endothelium development |
| GO:0072262 | 0.014 | 15.936 | 1 | 3 | 4 | metanephric glomerular mesangial cell proliferation involved in metanephros development |
| GO:0097048 | 0.014 | 15.936 | 1 | 3 | 4 | dendritic cell apoptotic process |
| GO:1900125 | 0.014 | 15.936 | 1 | 3 | 4 | regulation of hyaluronan biosynthetic process |
| GO:2000589 | 0.014 | 15.936 | 1 | 3 | 4 | regulation of metanephric mesenchymal cell migration |
| GO:2000668 | 0.014 | 15.936 | 1 | 3 | 4 | regulation of dendritic cell apoptotic process |
| GO:2000794 | 0.014 | 15.936 | 1 | 3 | 4 | regulation of epithelial cell proliferation involved in lung morphogenesis |
| GO:0001936 | 0.014 | 2.016 | 10 | 17 | 62 | regulation of endothelial cell proliferation |
| GO:0046777 | 0.014 | 1.597 | 23 | 34 | 148 | protein autophosphorylation |
| GO:0006892 | 0.014 | 2.196 | 8 | 14 | 48 | post-Golgi vesicle-mediated transport |
| GO:0071496 | 0.015 | 1.606 | 23 | 33 | 143 | cellular response to external stimulus |
| GO:0001886 | 0.015 | 7.086 | 1 | 4 | 7 | endothelial cell morphogenesis |
| GO:0002523 | 0.015 | 7.086 | 1 | 4 | 7 | leukocyte migration involved in inflammatory response |
| GO:0003097 | 0.015 | 7.086 | 1 | 4 | 7 | renal water transport |
| GO:0010886 | 0.015 | 7.086 | 1 | 4 | 7 | positive regulation of cholesterol storage |
| GO:0030220 | 0.015 | 7.086 | 1 | 4 | 7 | platelet formation |
| GO:0032232 | 0.015 | 7.086 | 1 | 4 | 7 | negative regulation of actin filament bundle assembly |
| GO:0032429 | 0.015 | 7.086 | 1 | 4 | 7 | regulation of phospholipase A2 activity |
| GO:0032495 | 0.015 | 7.086 | 1 | 4 | 7 | response to muramyl dipeptide |
| GO:0032823 | 0.015 | 7.086 | 1 | 4 | 7 | regulation of natural killer cell differentiation |
| GO:0035024 | 0.015 | 7.086 | 1 | 4 | 7 | negative regulation of Rho protein signal transduction |
| GO:0036149 | 0.015 | 7.086 | 1 | 4 | 7 | phosphatidylinositol acyl-chain remodeling |
| GO:0036150 | 0.015 | 7.086 | 1 | 4 | 7 | phosphatidylserine acyl-chain remodeling |
| GO:0036344 | 0.015 | 7.086 | 1 | 4 | 7 | platelet morphogenesis |
| GO:0046475 | 0.015 | 7.086 | 1 | 4 | 7 | glycerophospholipid catabolic process |
| GO:0051770 | 0.015 | 7.086 | 1 | 4 | 7 | positive regulation of nitric-oxide synthase biosynthetic process |
| GO:0060315 | 0.015 | 7.086 | 1 | 4 | 7 | negative regulation of ryanodine-sensitive calcium-release channel activity |
| GO:0072102 | 0.015 | 7.086 | 1 | 4 | 7 | glomerulus morphogenesis |
| GO:2000810 | 0.015 | 7.086 | 1 | 4 | 7 | regulation of tight junction assembly |
| GO:0035051 | 0.015 | 1.914 | 11 | 19 | 72 | cardiocyte differentiation |
| GO:0002637 | 0.015 | 2.819 | 4 | 9 | 26 | regulation of immunoglobulin production |
| GO:0042092 | 0.015 | 2.819 | 4 | 9 | 26 | type 2 immune response |
| GO:0046626 | 0.015 | 2.819 | 4 | 9 | 26 | regulation of insulin receptor signaling pathway |
| GO:2001257 | 0.015 | 2.819 | 4 | 9 | 26 | regulation of cation channel activity |
| GO:0010224 | 0.015 | 3.990 | 2 | 6 | 14 | response to UV-B |
| GO:0030866 | 0.015 | 3.990 | 2 | 6 | 14 | cortical actin cytoskeleton organization |
| GO:0032689 | 0.015 | 3.990 | 2 | 6 | 14 | negative regulation of interferon-gamma production |
| GO:0032897 | 0.015 | 3.990 | 2 | 6 | 14 | negative regulation of viral transcription |
| GO:0045671 | 0.015 | 3.990 | 2 | 6 | 14 | negative regulation of osteoclast differentiation |
| GO:0051647 | 0.015 | 3.990 | 2 | 6 | 14 | nucleus localization |
| GO:0010927 | 0.015 | 1.839 | 13 | 21 | 82 | cellular component assembly involved in morphogenesis |
| GO:0001906 | 0.015 | 2.105 | 8 | 15 | 53 | cell killing |
| GO:0010862 | 0.016 | 3.042 | 3 | 8 | 22 | positive regulation of pathway-restricted SMAD protein phosphorylation |
| GO:0030104 | 0.016 | 3.042 | 3 | 8 | 22 | water homeostasis |
| GO:0043552 | 0.016 | 3.042 | 3 | 8 | 22 | positive regulation of phosphatidylinositol 3-kinase activity |
| GO:0050690 | 0.016 | 3.042 | 3 | 8 | 22 | regulation of defense response to virus by virus |
| GO:0006793 | 0.016 | 1.173 | 276 | 306 | 1741 | phosphorus metabolic process |
| GO:0043086 | 0.016 | 1.346 | 61 | 77 | 385 | negative regulation of catalytic activity |
| GO:0010628 | 0.016 | 1.241 | 126 | 148 | 795 | positive regulation of gene expression |
| GO:0017145 | 0.016 | 3.386 | 3 | 7 | 18 | stem cell division |
| GO:0032855 | 0.016 | 3.386 | 3 | 7 | 18 | positive regulation of Rac GTPase activity |
| GO:0045687 | 0.016 | 3.386 | 3 | 7 | 18 | positive regulation of glial cell differentiation |
| GO:0051004 | 0.016 | 3.386 | 3 | 7 | 18 | regulation of lipoprotein lipase activity |
| GO:0031333 | 0.016 | 2.032 | 9 | 16 | 58 | negative regulation of protein complex assembly |
| GO:0046578 | 0.016 | 1.520 | 29 | 40 | 181 | regulation of Ras protein signal transduction |
| GO:0010522 | 0.016 | 2.236 | 7 | 13 | 44 | regulation of calcium ion transport into cytosol |
| GO:0032615 | 0.016 | 2.442 | 6 | 11 | 35 | interleukin-12 production |
| GO:0043367 | 0.016 | 2.442 | 6 | 11 | 35 | CD4-positive, alpha-beta T cell differentiation |
| GO:0048015 | 0.016 | 1.664 | 19 | 28 | 118 | phosphatidylinositol-mediated signaling |
| GO:0048017 | 0.016 | 1.664 | 19 | 28 | 118 | inositol lipid-mediated signaling |
| GO:0051173 | 0.017 | 1.227 | 140 | 162 | 879 | positive regulation of nitrogen compound metabolic process |
| GO:0007162 | 0.017 | 1.921 | 11 | 18 | 68 | negative regulation of cell adhesion |
| GO:0060401 | 0.017 | 1.921 | 11 | 18 | 68 | cytosolic calcium ion transport |
| GO:0048762 | 0.017 | 1.713 | 16 | 25 | 103 | mesenchymal cell differentiation |
| GO:1900180 | 0.017 | 1.713 | 16 | 25 | 103 | regulation of protein localization to nucleus |
| GO:0016064 | 0.017 | 1.878 | 12 | 19 | 73 | immunoglobulin mediated immune response |
| GO:0045667 | 0.017 | 1.878 | 12 | 19 | 73 | regulation of osteoblast differentiation |
| GO:0051384 | 0.017 | 1.733 | 16 | 24 | 98 | response to glucocorticoid stimulus |
| GO:0032270 | 0.017 | 1.268 | 98 | 117 | 616 | positive regulation of cellular protein metabolic process |
| GO:0007043 | 0.017 | 2.133 | 8 | 14 | 49 | cell-cell junction assembly |
| GO:0014013 | 0.017 | 2.133 | 8 | 14 | 49 | regulation of gliogenesis |
| GO:0031623 | 0.017 | 2.133 | 8 | 14 | 49 | receptor internalization |
| GO:0071887 | 0.017 | 2.133 | 8 | 14 | 49 | leukocyte apoptotic process |
| GO:0003206 | 0.017 | 1.841 | 12 | 20 | 78 | cardiac chamber morphogenesis |
| GO:0051235 | 0.017 | 1.621 | 20 | 30 | 129 | maintenance of location |
| GO:0010605 | 0.017 | 1.218 | 150 | 173 | 945 | negative regulation of macromolecule metabolic process |
| GO:0071375 | 0.018 | 1.457 | 35 | 47 | 220 | cellular response to peptide hormone stimulus |
| GO:1901653 | 0.018 | 1.457 | 35 | 47 | 220 | cellular response to peptide |
| GO:0042116 | 0.018 | 2.536 | 5 | 10 | 31 | macrophage activation |
| GO:0043297 | 0.018 | 2.536 | 5 | 10 | 31 | apical junction assembly |
| GO:0046686 | 0.018 | 2.536 | 5 | 10 | 31 | response to cadmium ion |
| GO:0070613 | 0.018 | 2.536 | 5 | 10 | 31 | regulation of protein processing |
| GO:0031401 | 0.018 | 1.279 | 89 | 107 | 559 | positive regulation of protein modification process |
| GO:0002286 | 0.018 | 2.284 | 6 | 12 | 40 | T cell activation involved in immune response |
| GO:0009628 | 0.018 | 1.270 | 94 | 113 | 594 | response to abiotic stimulus |
| GO:0048011 | 0.018 | 1.495 | 30 | 41 | 188 | nerve growth factor receptor signaling pathway |
| GO:0065003 | 0.019 | 1.252 | 107 | 127 | 676 | macromolecular complex assembly |
| GO:0032412 | 0.019 | 1.985 | 9 | 16 | 59 | regulation of ion transmembrane transporter activity |
| GO:0030217 | 0.019 | 1.595 | 21 | 31 | 135 | T cell differentiation |
| GO:0010558 | 0.019 | 1.254 | 105 | 124 | 659 | negative regulation of macromolecule biosynthetic process |
| GO:0031960 | 0.019 | 1.691 | 17 | 25 | 104 | response to corticosteroid stimulus |
| GO:0003014 | 0.019 | 1.930 | 10 | 17 | 64 | renal system process |
| GO:0052548 | 0.019 | 1.481 | 31 | 42 | 194 | regulation of endopeptidase activity |
| GO:0002244 | 0.019 | 2.662 | 4 | 9 | 27 | hematopoietic progenitor cell differentiation |
| GO:0006958 | 0.019 | 2.662 | 4 | 9 | 27 | complement activation, classical pathway |
| GO:0022406 | 0.019 | 2.662 | 4 | 9 | 27 | membrane docking |
| GO:0046503 | 0.019 | 2.662 | 4 | 9 | 27 | glycerolipid catabolic process |
| GO:0043244 | 0.019 | 2.165 | 7 | 13 | 45 | regulation of protein complex disassembly |
| GO:0050868 | 0.019 | 2.165 | 7 | 13 | 45 | negative regulation of T cell activation |
| GO:0009894 | 0.020 | 1.340 | 58 | 73 | 366 | regulation of catabolic process |
| GO:0046683 | 0.020 | 1.844 | 12 | 19 | 74 | response to organophosphorus |
| GO:0006658 | 0.020 | 4.431 | 2 | 5 | 11 | phosphatidylserine metabolic process |
| GO:0006925 | 0.020 | 4.431 | 2 | 5 | 11 | inflammatory cell apoptotic process |
| GO:0010882 | 0.020 | 4.431 | 2 | 5 | 11 | regulation of cardiac muscle contraction by calcium ion signaling |
| GO:0021591 | 0.020 | 4.431 | 2 | 5 | 11 | ventricular system development |
| GO:0032634 | 0.020 | 4.431 | 2 | 5 | 11 | interleukin-5 production |
| GO:0032674 | 0.020 | 4.431 | 2 | 5 | 11 | regulation of interleukin-5 production |
| GO:0032715 | 0.020 | 4.431 | 2 | 5 | 11 | negative regulation of interleukin-6 production |
| GO:0035306 | 0.020 | 4.431 | 2 | 5 | 11 | positive regulation of dephosphorylation |
| GO:0045922 | 0.020 | 4.431 | 2 | 5 | 11 | negative regulation of fatty acid metabolic process |
| GO:0051150 | 0.020 | 4.431 | 2 | 5 | 11 | regulation of smooth muscle cell differentiation |
| GO:0070498 | 0.020 | 4.431 | 2 | 5 | 11 | interleukin-1-mediated signaling pathway |
| GO:0071674 | 0.020 | 4.431 | 2 | 5 | 11 | mononuclear cell migration |
| GO:0002821 | 0.020 | 2.344 | 6 | 11 | 36 | positive regulation of adaptive immune response |
| GO:0071396 | 0.020 | 1.507 | 27 | 38 | 173 | cellular response to lipid |
| GO:0032609 | 0.021 | 2.073 | 8 | 14 | 50 | interferon-gamma production |
| GO:0042991 | 0.021 | 2.073 | 8 | 14 | 50 | transcription factor import into nucleus |
| GO:0030890 | 0.021 | 2.839 | 4 | 8 | 23 | positive regulation of B cell proliferation |
| GO:0044246 | 0.021 | 2.839 | 4 | 8 | 23 | regulation of multicellular organismal metabolic process |
| GO:0045599 | 0.021 | 2.839 | 4 | 8 | 23 | negative regulation of fat cell differentiation |
| GO:0060330 | 0.021 | 2.839 | 4 | 8 | 23 | regulation of response to interferon-gamma |
| GO:0060334 | 0.021 | 2.839 | 4 | 8 | 23 | regulation of interferon-gamma-mediated signaling pathway |
| GO:2000116 | 0.021 | 1.579 | 22 | 31 | 136 | regulation of cysteine-type endopeptidase activity |
| GO:0010951 | 0.021 | 1.654 | 17 | 26 | 110 | negative regulation of endopeptidase activity |
| GO:0043281 | 0.021 | 1.588 | 21 | 30 | 131 | regulation of cysteine-type endopeptidase activity involved in apoptotic process |
| GO:0030855 | 0.021 | 1.442 | 34 | 46 | 217 | epithelial cell differentiation |
| GO:0002675 | 0.022 | 3.104 | 3 | 7 | 19 | positive regulation of acute inflammatory response |
| GO:0014009 | 0.022 | 3.104 | 3 | 7 | 19 | glial cell proliferation |
| GO:0032414 | 0.022 | 3.104 | 3 | 7 | 19 | positive regulation of ion transmembrane transporter activity |
| GO:0045730 | 0.022 | 3.104 | 3 | 7 | 19 | respiratory burst |
| GO:0048260 | 0.022 | 3.104 | 3 | 7 | 19 | positive regulation of receptor-mediated endocytosis |
| GO:0050869 | 0.022 | 3.104 | 3 | 7 | 19 | negative regulation of B cell activation |
| GO:2000401 | 0.022 | 3.104 | 3 | 7 | 19 | regulation of lymphocyte migration |
| GO:0001516 | 0.022 | 3.546 | 2 | 6 | 15 | prostaglandin biosynthetic process |
| GO:0016601 | 0.022 | 3.546 | 2 | 6 | 15 | Rac protein signal transduction |
| GO:0030865 | 0.022 | 3.546 | 2 | 6 | 15 | cortical cytoskeleton organization |
| GO:0033630 | 0.022 | 3.546 | 2 | 6 | 15 | positive regulation of cell adhesion mediated by integrin |
| GO:0035455 | 0.022 | 3.546 | 2 | 6 | 15 | response to interferon-alpha |
| GO:0043001 | 0.022 | 3.546 | 2 | 6 | 15 | Golgi to plasma membrane protein transport |
| GO:0046457 | 0.022 | 3.546 | 2 | 6 | 15 | prostanoid biosynthetic process |
| GO:0060251 | 0.022 | 3.546 | 2 | 6 | 15 | regulation of glial cell proliferation |
| GO:0090279 | 0.022 | 3.546 | 2 | 6 | 15 | regulation of calcium ion import |
| GO:0030510 | 0.022 | 2.205 | 7 | 12 | 41 | regulation of BMP signaling pathway |
| GO:0055117 | 0.022 | 2.205 | 7 | 12 | 41 | regulation of cardiac muscle contraction |
| GO:0070664 | 0.022 | 2.205 | 7 | 12 | 41 | negative regulation of leukocyte proliferation |
| GO:0042113 | 0.022 | 1.599 | 20 | 29 | 126 | B cell activation |
| GO:0061351 | 0.022 | 1.889 | 10 | 17 | 65 | neural precursor cell proliferation |
| GO:0000060 | 0.022 | 2.421 | 5 | 10 | 32 | protein import into nucleus, translocation |
| GO:0014812 | 0.022 | 2.421 | 5 | 10 | 32 | muscle cell migration |
| GO:0023021 | 0.022 | 2.421 | 5 | 10 | 32 | termination of signal transduction |
| GO:0048566 | 0.022 | 2.421 | 5 | 10 | 32 | embryonic digestive tract development |
| GO:0055006 | 0.022 | 2.421 | 5 | 10 | 32 | cardiac cell development |
| GO:0051172 | 0.023 | 1.246 | 103 | 122 | 651 | negative regulation of nitrogen compound metabolic process |
| GO:0032320 | 0.023 | 1.847 | 11 | 18 | 70 | positive regulation of Ras GTPase activity |
| GO:0032526 | 0.023 | 1.847 | 11 | 18 | 70 | response to retinoic acid |
| GO:2000027 | 0.024 | 1.634 | 18 | 26 | 111 | regulation of organ morphogenesis |
| GO:0033157 | 0.024 | 1.573 | 21 | 30 | 132 | regulation of intracellular protein transport |
| GO:0071214 | 0.024 | 1.582 | 20 | 29 | 127 | cellular response to abiotic stimulus |
| GO:0002062 | 0.024 | 2.017 | 8 | 14 | 51 | chondrocyte differentiation |
| GO:0031329 | 0.024 | 1.348 | 51 | 64 | 319 | regulation of cellular catabolic process |
| GO:1901879 | 0.025 | 2.253 | 6 | 11 | 37 | regulation of protein depolymerization |
| GO:0030042 | 0.025 | 2.522 | 4 | 9 | 28 | actin filament depolymerization |
| GO:0048286 | 0.025 | 2.522 | 4 | 9 | 28 | lung alveolus development |
| GO:0060038 | 0.025 | 2.522 | 4 | 9 | 28 | cardiac muscle cell proliferation |
| GO:0034504 | 0.025 | 1.451 | 31 | 42 | 197 | protein localization to nucleus |
| GO:0050769 | 0.025 | 1.683 | 15 | 23 | 96 | positive regulation of neurogenesis |
| GO:0007569 | 0.025 | 1.951 | 9 | 15 | 56 | cell aging |
| GO:0001810 | 0.025 | Inf | 0 | 2 | 2 | regulation of type I hypersensitivity |
| GO:0001812 | 0.025 | Inf | 0 | 2 | 2 | positive regulation of type I hypersensitivity |
| GO:0002005 | 0.025 | Inf | 0 | 2 | 2 | angiotensin catabolic process in blood |
| GO:0002032 | 0.025 | Inf | 0 | 2 | 2 | desensitization of G-protein coupled receptor protein signaling pathway by arrestin |
| GO:0002220 | 0.025 | Inf | 0 | 2 | 2 | innate immune response activating cell surface receptor signaling pathway |
| GO:0002238 | 0.025 | Inf | 0 | 2 | 2 | response to molecule of fungal origin |
| GO:0002384 | 0.025 | Inf | 0 | 2 | 2 | hepatic immune response |
| GO:0002434 | 0.025 | Inf | 0 | 2 | 2 | immune complex clearance |
| GO:0002436 | 0.025 | Inf | 0 | 2 | 2 | immune complex clearance by monocytes and macrophages |
| GO:0006971 | 0.025 | Inf | 0 | 2 | 2 | hypotonic response |
| GO:0007181 | 0.025 | Inf | 0 | 2 | 2 | transforming growth factor beta receptor complex assembly |
| GO:0007521 | 0.025 | Inf | 0 | 2 | 2 | muscle cell fate determination |
| GO:0009726 | 0.025 | Inf | 0 | 2 | 2 | detection of endogenous stimulus |
| GO:0010511 | 0.025 | Inf | 0 | 2 | 2 | regulation of phosphatidylinositol biosynthetic process |
| GO:0010512 | 0.025 | Inf | 0 | 2 | 2 | negative regulation of phosphatidylinositol biosynthetic process |
| GO:0010621 | 0.025 | Inf | 0 | 2 | 2 | negative regulation of transcription by transcription factor localization |
| GO:0014040 | 0.025 | Inf | 0 | 2 | 2 | positive regulation of Schwann cell differentiation |
| GO:0014809 | 0.025 | Inf | 0 | 2 | 2 | regulation of skeletal muscle contraction by regulation of release of sequestered calcium ion |
| GO:0014841 | 0.025 | Inf | 0 | 2 | 2 | satellite cell proliferation |
| GO:0014842 | 0.025 | Inf | 0 | 2 | 2 | regulation of satellite cell proliferation |
| GO:0016068 | 0.025 | Inf | 0 | 2 | 2 | type I hypersensitivity |
| GO:0021769 | 0.025 | Inf | 0 | 2 | 2 | orbitofrontal cortex development |
| GO:0030185 | 0.025 | Inf | 0 | 2 | 2 | nitric oxide transport |
| GO:0030397 | 0.025 | Inf | 0 | 2 | 2 | membrane disassembly |
| GO:0032672 | 0.025 | Inf | 0 | 2 | 2 | regulation of interleukin-3 production |
| GO:0032764 | 0.025 | Inf | 0 | 2 | 2 | negative regulation of mast cell cytokine production |
| GO:0033159 | 0.025 | Inf | 0 | 2 | 2 | negative regulation of protein import into nucleus, translocation |
| GO:0033364 | 0.025 | Inf | 0 | 2 | 2 | mast cell secretory granule organization |
| GO:0034036 | 0.025 | Inf | 0 | 2 | 2 | purine ribonucleoside bisphosphate biosynthetic process |
| GO:0034136 | 0.025 | Inf | 0 | 2 | 2 | negative regulation of toll-like receptor 2 signaling pathway |
| GO:0034144 | 0.025 | Inf | 0 | 2 | 2 | negative regulation of toll-like receptor 4 signaling pathway |
| GO:0035360 | 0.025 | Inf | 0 | 2 | 2 | positive regulation of peroxisome proliferator activated receptor signaling pathway |
| GO:0035607 | 0.025 | Inf | 0 | 2 | 2 | fibroblast growth factor receptor signaling pathway involved in orbitofrontal cortex development |
| GO:0035759 | 0.025 | Inf | 0 | 2 | 2 | mesangial cell-matrix adhesion |
| GO:0038001 | 0.025 | Inf | 0 | 2 | 2 | paracrine signaling |
| GO:0038156 | 0.025 | Inf | 0 | 2 | 2 | interleukin-3-mediated signaling pathway |
| GO:0042223 | 0.025 | Inf | 0 | 2 | 2 | interleukin-3 biosynthetic process |
| GO:0043305 | 0.025 | Inf | 0 | 2 | 2 | negative regulation of mast cell degranulation |
| GO:0045399 | 0.025 | Inf | 0 | 2 | 2 | regulation of interleukin-3 biosynthetic process |
| GO:0045401 | 0.025 | Inf | 0 | 2 | 2 | positive regulation of interleukin-3 biosynthetic process |
| GO:0046168 | 0.025 | Inf | 0 | 2 | 2 | glycerol-3-phosphate catabolic process |
| GO:0046294 | 0.025 | Inf | 0 | 2 | 2 | formaldehyde catabolic process |
| GO:0048549 | 0.025 | Inf | 0 | 2 | 2 | positive regulation of pinocytosis |
| GO:0050428 | 0.025 | Inf | 0 | 2 | 2 | 3'-phosphoadenosine 5'-phosphosulfate biosynthetic process |
| GO:0050779 | 0.025 | Inf | 0 | 2 | 2 | RNA destabilization |
| GO:0050861 | 0.025 | Inf | 0 | 2 | 2 | positive regulation of B cell receptor signaling pathway |
| GO:0050902 | 0.025 | Inf | 0 | 2 | 2 | leukocyte adhesive activation |
| GO:0051081 | 0.025 | Inf | 0 | 2 | 2 | nuclear envelope disassembly |
| GO:0052551 | 0.025 | Inf | 0 | 2 | 2 | response to defense-related nitric oxide production by other organism involved in symbiotic interaction |
| GO:0052565 | 0.025 | Inf | 0 | 2 | 2 | response to defense-related host nitric oxide production |
| GO:0055073 | 0.025 | Inf | 0 | 2 | 2 | cadmium ion homeostasis |
| GO:0055095 | 0.025 | Inf | 0 | 2 | 2 | lipoprotein particle mediated signaling |
| GO:0055096 | 0.025 | Inf | 0 | 2 | 2 | low-density lipoprotein particle mediated signaling |
| GO:0060211 | 0.025 | Inf | 0 | 2 | 2 | regulation of nuclear-transcribed mRNA poly(A) tail shortening |
| GO:0060213 | 0.025 | Inf | 0 | 2 | 2 | positive regulation of nuclear-transcribed mRNA poly(A) tail shortening |
| GO:0060375 | 0.025 | Inf | 0 | 2 | 2 | regulation of mast cell differentiation |
| GO:0060435 | 0.025 | Inf | 0 | 2 | 2 | bronchiole development |
| GO:0061364 | 0.025 | Inf | 0 | 2 | 2 | apoptotic process involved in luteolysis |
| GO:0070341 | 0.025 | Inf | 0 | 2 | 2 | fat cell proliferation |
| GO:0070344 | 0.025 | Inf | 0 | 2 | 2 | regulation of fat cell proliferation |
| GO:0070417 | 0.025 | Inf | 0 | 2 | 2 | cellular response to cold |
| GO:0070488 | 0.025 | Inf | 0 | 2 | 2 | neutrophil aggregation |
| GO:0071472 | 0.025 | Inf | 0 | 2 | 2 | cellular response to salt stress |
| GO:0071476 | 0.025 | Inf | 0 | 2 | 2 | cellular hypotonic response |
| GO:0071505 | 0.025 | Inf | 0 | 2 | 2 | response to mycophenolic acid |
| GO:0071506 | 0.025 | Inf | 0 | 2 | 2 | cellular response to mycophenolic acid |
| GO:0071672 | 0.025 | Inf | 0 | 2 | 2 | negative regulation of smooth muscle cell chemotaxis |
| GO:0071676 | 0.025 | Inf | 0 | 2 | 2 | negative regulation of mononuclear cell migration |
| GO:0072126 | 0.025 | Inf | 0 | 2 | 2 | positive regulation of glomerular mesangial cell proliferation |
| GO:0072209 | 0.025 | Inf | 0 | 2 | 2 | metanephric mesangial cell differentiation |
| GO:0072254 | 0.025 | Inf | 0 | 2 | 2 | metanephric glomerular mesangial cell differentiation |
| GO:0072683 | 0.025 | Inf | 0 | 2 | 2 | T cell extravasation |
| GO:0090264 | 0.025 | Inf | 0 | 2 | 2 | regulation of immune complex clearance by monocytes and macrophages |
| GO:0090265 | 0.025 | Inf | 0 | 2 | 2 | positive regulation of immune complex clearance by monocytes and macrophages |
| GO:1900087 | 0.025 | Inf | 0 | 2 | 2 | positive regulation of G1/S transition of mitotic cell cycle |
| GO:1900151 | 0.025 | Inf | 0 | 2 | 2 | regulation of nuclear-transcribed mRNA catabolic process, deadenylation-dependent decay |
| GO:1900221 | 0.025 | Inf | 0 | 2 | 2 | regulation of beta-amyloid clearance |
| GO:1900222 | 0.025 | Inf | 0 | 2 | 2 | negative regulation of beta-amyloid clearance |
| GO:1901490 | 0.025 | Inf | 0 | 2 | 2 | regulation of lymphangiogenesis |
| GO:1901876 | 0.025 | Inf | 0 | 2 | 2 | regulation of calcium ion binding |
| GO:1901877 | 0.025 | Inf | 0 | 2 | 2 | negative regulation of calcium ion binding |
| GO:2000269 | 0.025 | Inf | 0 | 2 | 2 | regulation of fibroblast apoptotic process |
| GO:2000270 | 0.025 | Inf | 0 | 2 | 2 | negative regulation of fibroblast apoptotic process |
| GO:2000471 | 0.025 | Inf | 0 | 2 | 2 | regulation of hematopoietic stem cell migration |
| GO:2000473 | 0.025 | Inf | 0 | 2 | 2 | positive regulation of hematopoietic stem cell migration |
| GO:2001245 | 0.025 | Inf | 0 | 2 | 2 | regulation of phosphatidylcholine biosynthetic process |
| GO:0006869 | 0.025 | 1.549 | 22 | 31 | 138 | lipid transport |
| GO:0060541 | 0.025 | 1.549 | 22 | 31 | 138 | respiratory system development |
| GO:0034762 | 0.026 | 1.725 | 14 | 21 | 86 | regulation of transmembrane transport |
| GO:0006939 | 0.026 | 1.850 | 10 | 17 | 66 | smooth muscle contraction |
| GO:0001820 | 0.026 | 5.314 | 1 | 4 | 8 | serotonin secretion |
| GO:0002577 | 0.026 | 5.314 | 1 | 4 | 8 | regulation of antigen processing and presentation |
| GO:0007494 | 0.026 | 5.314 | 1 | 4 | 8 | midgut development |
| GO:0015669 | 0.026 | 5.314 | 1 | 4 | 8 | gas transport |
| GO:0019852 | 0.026 | 5.314 | 1 | 4 | 8 | L-ascorbic acid metabolic process |
| GO:0035357 | 0.026 | 5.314 | 1 | 4 | 8 | peroxisome proliferator activated receptor signaling pathway |
| GO:0035457 | 0.026 | 5.314 | 1 | 4 | 8 | cellular response to interferon-alpha |
| GO:0042090 | 0.026 | 5.314 | 1 | 4 | 8 | interleukin-12 biosynthetic process |
| GO:0043171 | 0.026 | 5.314 | 1 | 4 | 8 | peptide catabolic process |
| GO:0043312 | 0.026 | 5.314 | 1 | 4 | 8 | neutrophil degranulation |
| GO:0045019 | 0.026 | 5.314 | 1 | 4 | 8 | negative regulation of nitric oxide biosynthetic process |
| GO:0045066 | 0.026 | 5.314 | 1 | 4 | 8 | regulatory T cell differentiation |
| GO:0045075 | 0.026 | 5.314 | 1 | 4 | 8 | regulation of interleukin-12 biosynthetic process |
| GO:0045717 | 0.026 | 5.314 | 1 | 4 | 8 | negative regulation of fatty acid biosynthetic process |
| GO:0060218 | 0.026 | 5.314 | 1 | 4 | 8 | hematopoietic stem cell differentiation |
| GO:0060252 | 0.026 | 5.314 | 1 | 4 | 8 | positive regulation of glial cell proliferation |
| GO:0060484 | 0.026 | 5.314 | 1 | 4 | 8 | lung-associated mesenchyme development |
| GO:0061081 | 0.026 | 5.314 | 1 | 4 | 8 | positive regulation of myeloid leukocyte cytokine production involved in immune response |
| GO:0071731 | 0.026 | 5.314 | 1 | 4 | 8 | response to nitric oxide |
| GO:2000008 | 0.026 | 5.314 | 1 | 4 | 8 | regulation of protein localization to cell surface |
| GO:0051224 | 0.026 | 1.779 | 12 | 19 | 76 | negative regulation of protein transport |
| GO:0032409 | 0.026 | 1.812 | 11 | 18 | 71 | regulation of transporter activity |
| GO:0010466 | 0.026 | 1.615 | 18 | 26 | 112 | negative regulation of peptidase activity |
| GO:0045893 | 0.026 | 1.226 | 115 | 134 | 725 | positive regulation of transcription, DNA-dependent |
| GO:0051129 | 0.026 | 1.387 | 40 | 52 | 253 | negative regulation of cellular component organization |
| GO:0010463 | 0.026 | 2.131 | 7 | 12 | 42 | mesenchymal cell proliferation |
| GO:0071902 | 0.027 | 1.478 | 27 | 37 | 171 | positive regulation of protein serine/threonine kinase activity |
| GO:0030308 | 0.027 | 1.643 | 16 | 24 | 102 | negative regulation of cell growth |
| GO:0051403 | 0.028 | 1.503 | 25 | 34 | 155 | stress-activated MAPK cascade |
| GO:0007431 | 0.028 | 2.315 | 5 | 10 | 33 | salivary gland development |
| GO:0055017 | 0.028 | 2.315 | 5 | 10 | 33 | cardiac muscle tissue growth |
| GO:0001776 | 0.028 | 2.037 | 7 | 13 | 47 | leukocyte homeostasis |
| GO:0035150 | 0.028 | 1.660 | 15 | 23 | 97 | regulation of tube size |
| GO:0050880 | 0.028 | 1.660 | 15 | 23 | 97 | regulation of blood vessel size |
| GO:0051649 | 0.028 | 1.173 | 200 | 223 | 1258 | establishment of localization in cell |
| GO:0021782 | 0.029 | 1.964 | 8 | 14 | 52 | glial cell development |
| GO:0033135 | 0.029 | 1.964 | 8 | 14 | 52 | regulation of peptidyl-serine phosphorylation |
| GO:0001937 | 0.029 | 2.864 | 3 | 7 | 20 | negative regulation of endothelial cell proliferation |
| GO:0015695 | 0.029 | 2.864 | 3 | 7 | 20 | organic cation transport |
| GO:0030224 | 0.029 | 2.864 | 3 | 7 | 20 | monocyte differentiation |
| GO:0030514 | 0.029 | 2.864 | 3 | 7 | 20 | negative regulation of BMP signaling pathway |
| GO:0034381 | 0.029 | 2.864 | 3 | 7 | 20 | plasma lipoprotein particle clearance |
| GO:0042481 | 0.029 | 2.864 | 3 | 7 | 20 | regulation of odontogenesis |
| GO:0043666 | 0.029 | 2.864 | 3 | 7 | 20 | regulation of phosphoprotein phosphatase activity |
| GO:0046580 | 0.029 | 2.864 | 3 | 7 | 20 | negative regulation of Ras protein signal transduction |
| GO:0050873 | 0.029 | 2.864 | 3 | 7 | 20 | brown fat cell differentiation |
| GO:0051693 | 0.029 | 2.864 | 3 | 7 | 20 | actin filament capping |
| GO:0060043 | 0.029 | 2.864 | 3 | 7 | 20 | regulation of cardiac muscle cell proliferation |
| GO:0051494 | 0.029 | 1.904 | 9 | 15 | 57 | negative regulation of cytoskeleton organization |
| GO:2000117 | 0.029 | 1.904 | 9 | 15 | 57 | negative regulation of cysteine-type endopeptidase activity |
| GO:0008104 | 0.029 | 1.187 | 163 | 184 | 1025 | protein localization |
| GO:0006917 | 0.030 | 1.514 | 23 | 32 | 145 | induction of apoptosis |
| GO:0012502 | 0.030 | 1.514 | 23 | 32 | 145 | induction of programmed cell death |
| GO:0044236 | 0.030 | 1.855 | 10 | 16 | 62 | multicellular organismal metabolic process |
| GO:0060402 | 0.030 | 1.813 | 11 | 17 | 67 | calcium ion transport into cytosol |
| GO:0003091 | 0.030 | 3.797 | 2 | 5 | 12 | renal water homeostasis |
| GO:0010880 | 0.030 | 3.797 | 2 | 5 | 12 | regulation of release of sequestered calcium ion into cytosol by sarcoplasmic reticulum |
| GO:0032816 | 0.030 | 3.797 | 2 | 5 | 12 | positive regulation of natural killer cell activation |
| GO:0042953 | 0.030 | 3.797 | 2 | 5 | 12 | lipoprotein transport |
| GO:0045624 | 0.030 | 3.797 | 2 | 5 | 12 | positive regulation of T-helper cell differentiation |
| GO:0045672 | 0.030 | 3.797 | 2 | 5 | 12 | positive regulation of osteoclast differentiation |
| GO:0051023 | 0.030 | 3.797 | 2 | 5 | 12 | regulation of immunoglobulin secretion |
| GO:0060742 | 0.030 | 3.797 | 2 | 5 | 12 | epithelial cell differentiation involved in prostate gland development |
| GO:0090322 | 0.030 | 3.797 | 2 | 5 | 12 | regulation of superoxide metabolic process |
| GO:0032945 | 0.030 | 2.170 | 6 | 11 | 38 | negative regulation of mononuclear cell proliferation |
| GO:0050672 | 0.030 | 2.170 | 6 | 11 | 38 | negative regulation of lymphocyte proliferation |
| GO:0060419 | 0.030 | 2.170 | 6 | 11 | 38 | heart growth |
| GO:0035239 | 0.030 | 1.410 | 34 | 45 | 216 | tube morphogenesis |
| GO:0007184 | 0.030 | 3.191 | 3 | 6 | 16 | SMAD protein import into nucleus |
| GO:0009395 | 0.030 | 3.191 | 3 | 6 | 16 | phospholipid catabolic process |
| GO:0010718 | 0.030 | 3.191 | 3 | 6 | 16 | positive regulation of epithelial to mesenchymal transition |
| GO:0033687 | 0.030 | 3.191 | 3 | 6 | 16 | osteoblast proliferation |
| GO:0048246 | 0.030 | 3.191 | 3 | 6 | 16 | macrophage chemotaxis |
| GO:0050710 | 0.030 | 3.191 | 3 | 6 | 16 | negative regulation of cytokine secretion |
| GO:0050891 | 0.030 | 3.191 | 3 | 6 | 16 | multicellular organismal water homeostasis |
| GO:0090022 | 0.030 | 3.191 | 3 | 6 | 16 | regulation of neutrophil chemotaxis |
| GO:0051146 | 0.031 | 1.470 | 27 | 36 | 167 | striated muscle cell differentiation |
| GO:0001767 | 0.031 | 7.967 | 1 | 3 | 5 | establishment of lymphocyte polarity |
| GO:0001768 | 0.031 | 7.967 | 1 | 3 | 5 | establishment of T cell polarity |
| GO:0001911 | 0.031 | 7.967 | 1 | 3 | 5 | negative regulation of leukocyte mediated cytotoxicity |
| GO:0002606 | 0.031 | 7.967 | 1 | 3 | 5 | positive regulation of dendritic cell antigen processing and presentation |
| GO:0002634 | 0.031 | 7.967 | 1 | 3 | 5 | regulation of germinal center formation |
| GO:0002691 | 0.031 | 7.967 | 1 | 3 | 5 | regulation of cellular extravasation |
| GO:0003181 | 0.031 | 7.967 | 1 | 3 | 5 | atrioventricular valve morphogenesis |
| GO:0014819 | 0.031 | 7.967 | 1 | 3 | 5 | regulation of skeletal muscle contraction |
| GO:0015670 | 0.031 | 7.967 | 1 | 3 | 5 | carbon dioxide transport |
| GO:0015884 | 0.031 | 7.967 | 1 | 3 | 5 | folic acid transport |
| GO:0019062 | 0.031 | 7.967 | 1 | 3 | 5 | viral attachment to host cell |
| GO:0021670 | 0.031 | 7.967 | 1 | 3 | 5 | lateral ventricle development |
| GO:0022028 | 0.031 | 7.967 | 1 | 3 | 5 | tangential migration from the subventricular zone to the olfactory bulb |
| GO:0022614 | 0.031 | 7.967 | 1 | 3 | 5 | membrane to membrane docking |
| GO:0031342 | 0.031 | 7.967 | 1 | 3 | 5 | negative regulation of cell killing |
| GO:0032516 | 0.031 | 7.967 | 1 | 3 | 5 | positive regulation of phosphoprotein phosphatase activity |
| GO:0032762 | 0.031 | 7.967 | 1 | 3 | 5 | mast cell cytokine production |
| GO:0033004 | 0.031 | 7.967 | 1 | 3 | 5 | negative regulation of mast cell activation |
| GO:0034350 | 0.031 | 7.967 | 1 | 3 | 5 | regulation of glial cell apoptotic process |
| GO:0034351 | 0.031 | 7.967 | 1 | 3 | 5 | negative regulation of glial cell apoptotic process |
| GO:0034638 | 0.031 | 7.967 | 1 | 3 | 5 | phosphatidylcholine catabolic process |
| GO:0035855 | 0.031 | 7.967 | 1 | 3 | 5 | megakaryocyte development |
| GO:0036119 | 0.031 | 7.967 | 1 | 3 | 5 | response to platelet-derived growth factor stimulus |
| GO:0043301 | 0.031 | 7.967 | 1 | 3 | 5 | negative regulation of leukocyte degranulation |
| GO:0043619 | 0.031 | 7.967 | 1 | 3 | 5 | regulation of transcription from RNA polymerase II promoter in response to oxidative stress |
| GO:0044406 | 0.031 | 7.967 | 1 | 3 | 5 | adhesion to host |
| GO:0044650 | 0.031 | 7.967 | 1 | 3 | 5 | adhesion of symbiont to host cell |
| GO:0045586 | 0.031 | 7.967 | 1 | 3 | 5 | regulation of gamma-delta T cell differentiation |
| GO:0045588 | 0.031 | 7.967 | 1 | 3 | 5 | positive regulation of gamma-delta T cell differentiation |
| GO:0045589 | 0.031 | 7.967 | 1 | 3 | 5 | regulation of regulatory T cell differentiation |
| GO:0051014 | 0.031 | 7.967 | 1 | 3 | 5 | actin filament severing |
| GO:0060373 | 0.031 | 7.967 | 1 | 3 | 5 | regulation of ventricular cardiac muscle cell membrane depolarization |
| GO:0060394 | 0.031 | 7.967 | 1 | 3 | 5 | negative regulation of pathway-restricted SMAD protein phosphorylation |
| GO:0060502 | 0.031 | 7.967 | 1 | 3 | 5 | epithelial cell proliferation involved in lung morphogenesis |
| GO:0060856 | 0.031 | 7.967 | 1 | 3 | 5 | establishment of blood-brain barrier |
| GO:0060907 | 0.031 | 7.967 | 1 | 3 | 5 | positive regulation of macrophage cytokine production |
| GO:0061013 | 0.031 | 7.967 | 1 | 3 | 5 | regulation of mRNA catabolic process |
| GO:0070099 | 0.031 | 7.967 | 1 | 3 | 5 | regulation of chemokine-mediated signaling pathway |
| GO:0070120 | 0.031 | 7.967 | 1 | 3 | 5 | ciliary neurotrophic factor-mediated signaling pathway |
| GO:0070391 | 0.031 | 7.967 | 1 | 3 | 5 | response to lipoteichoic acid |
| GO:0071223 | 0.031 | 7.967 | 1 | 3 | 5 | cellular response to lipoteichoic acid |
| GO:0071503 | 0.031 | 7.967 | 1 | 3 | 5 | response to heparin |
| GO:0071504 | 0.031 | 7.967 | 1 | 3 | 5 | cellular response to heparin |
| GO:0072103 | 0.031 | 7.967 | 1 | 3 | 5 | glomerulus vasculature morphogenesis |
| GO:0072104 | 0.031 | 7.967 | 1 | 3 | 5 | glomerular capillary formation |
| GO:0072338 | 0.031 | 7.967 | 1 | 3 | 5 | cellular lactam metabolic process |
| GO:2000134 | 0.031 | 7.967 | 1 | 3 | 5 | negative regulation of G1/S transition of mitotic cell cycle |
| GO:0003007 | 0.031 | 1.527 | 21 | 30 | 135 | heart morphogenesis |
| GO:0042509 | 0.032 | 2.062 | 7 | 12 | 43 | regulation of tyrosine phosphorylation of STAT protein |
| GO:0006968 | 0.033 | 1.979 | 8 | 13 | 48 | cellular defense response |
| GO:0060284 | 0.033 | 1.302 | 58 | 71 | 364 | regulation of cell development |
| GO:0042476 | 0.033 | 1.718 | 12 | 19 | 78 | odontogenesis |
| GO:0051897 | 0.033 | 1.913 | 8 | 14 | 53 | positive regulation of protein kinase B signaling cascade |
| GO:0010720 | 0.033 | 1.542 | 20 | 28 | 125 | positive regulation of cell development |
| GO:0050767 | 0.034 | 1.335 | 47 | 59 | 296 | regulation of neurogenesis |
| GO:0001974 | 0.034 | 2.218 | 5 | 10 | 34 | blood vessel remodeling |
| GO:0032655 | 0.034 | 2.218 | 5 | 10 | 34 | regulation of interleukin-12 production |
| GO:0042044 | 0.034 | 2.218 | 5 | 10 | 34 | fluid transport |
| GO:0043242 | 0.034 | 2.218 | 5 | 10 | 34 | negative regulation of protein complex disassembly |
| GO:0046889 | 0.034 | 2.218 | 5 | 10 | 34 | positive regulation of lipid biosynthetic process |
| GO:0090382 | 0.034 | 2.218 | 5 | 10 | 34 | phagosome maturation |
| GO:0030048 | 0.034 | 1.815 | 10 | 16 | 63 | actin filament-based movement |
| GO:0032411 | 0.034 | 2.504 | 4 | 8 | 25 | positive regulation of transporter activity |
| GO:0032481 | 0.034 | 2.504 | 4 | 8 | 25 | positive regulation of type I interferon production |
| GO:0048662 | 0.034 | 2.504 | 4 | 8 | 25 | negative regulation of smooth muscle cell proliferation |
| GO:0032869 | 0.034 | 1.462 | 26 | 35 | 163 | cellular response to insulin stimulus |
| GO:0071407 | 0.034 | 1.512 | 22 | 30 | 136 | cellular response to organic cyclic compound |
| GO:0051241 | 0.035 | 1.346 | 43 | 55 | 274 | negative regulation of multicellular organismal process |
| GO:0008286 | 0.036 | 1.519 | 21 | 29 | 131 | insulin receptor signaling pathway |
| GO:0042531 | 0.036 | 2.092 | 6 | 11 | 39 | positive regulation of tyrosine phosphorylation of STAT protein |
| GO:0051248 | 0.036 | 1.300 | 56 | 69 | 354 | negative regulation of protein metabolic process |
| GO:0043542 | 0.036 | 1.648 | 14 | 21 | 89 | endothelial cell migration |
| GO:0043112 | 0.037 | 1.667 | 13 | 20 | 84 | receptor metabolic process |
| GO:0042108 | 0.037 | 1.997 | 7 | 12 | 44 | positive regulation of cytokine biosynthetic process |
| GO:0042310 | 0.037 | 1.997 | 7 | 12 | 44 | vasoconstriction |
| GO:0051046 | 0.038 | 1.306 | 53 | 65 | 332 | regulation of secretion |
| GO:0048565 | 0.038 | 1.689 | 13 | 19 | 79 | digestive tract development |
| GO:0021700 | 0.038 | 1.498 | 22 | 30 | 137 | developmental maturation |
| GO:0030835 | 0.038 | 2.659 | 3 | 7 | 21 | negative regulation of actin filament depolymerization |
| GO:0035924 | 0.038 | 2.659 | 3 | 7 | 21 | cellular response to vascular endothelial growth factor stimulus |
| GO:0042517 | 0.038 | 2.659 | 3 | 7 | 21 | positive regulation of tyrosine phosphorylation of Stat3 protein |
| GO:0042993 | 0.038 | 2.659 | 3 | 7 | 21 | positive regulation of transcription factor import into nucleus |
| GO:0043124 | 0.038 | 2.659 | 3 | 7 | 21 | negative regulation of I-kappaB kinase/NF-kappaB cascade |
| GO:0050830 | 0.038 | 2.659 | 3 | 7 | 21 | defense response to Gram-positive bacterium |
| GO:0051216 | 0.038 | 1.534 | 19 | 27 | 121 | cartilage development |
| GO:0046039 | 0.038 | 1.376 | 36 | 46 | 225 | GTP metabolic process |
| GO:0007435 | 0.038 | 2.281 | 5 | 9 | 30 | salivary gland morphogenesis |
| GO:0014855 | 0.038 | 2.281 | 5 | 9 | 30 | striated muscle cell proliferation |
| GO:0030032 | 0.038 | 2.281 | 5 | 9 | 30 | lamellipodium assembly |
| GO:0072676 | 0.038 | 2.281 | 5 | 9 | 30 | lymphocyte migration |
| GO:0042311 | 0.038 | 1.924 | 8 | 13 | 49 | vasodilation |
| GO:0042990 | 0.038 | 1.924 | 8 | 13 | 49 | regulation of transcription factor import into nucleus |
| GO:0000910 | 0.039 | 1.743 | 11 | 17 | 69 | cytokinesis |
| GO:0022898 | 0.039 | 1.777 | 10 | 16 | 64 | regulation of transmembrane transporter activity |
| GO:0032868 | 0.039 | 1.408 | 30 | 40 | 192 | response to insulin stimulus |
| GO:0051960 | 0.039 | 1.305 | 52 | 64 | 327 | regulation of nervous system development |
| GO:0006184 | 0.040 | 1.376 | 35 | 45 | 220 | GTP catabolic process |
| GO:0031400 | 0.040 | 1.360 | 38 | 48 | 237 | negative regulation of protein modification process |
| GO:0008543 | 0.040 | 1.552 | 18 | 25 | 111 | fibroblast growth factor receptor signaling pathway |
| GO:0015850 | 0.041 | 1.624 | 14 | 21 | 90 | organic hydroxy compound transport |
| GO:0000188 | 0.041 | 2.900 | 3 | 6 | 17 | inactivation of MAPK activity |
| GO:0002407 | 0.041 | 2.900 | 3 | 6 | 17 | dendritic cell chemotaxis |
| GO:0002825 | 0.041 | 2.900 | 3 | 6 | 17 | regulation of T-helper 1 type immune response |
| GO:0006706 | 0.041 | 2.900 | 3 | 6 | 17 | steroid catabolic process |
| GO:0006893 | 0.041 | 2.900 | 3 | 6 | 17 | Golgi to plasma membrane transport |
| GO:0021846 | 0.041 | 2.900 | 3 | 6 | 17 | cell proliferation in forebrain |
| GO:0032413 | 0.041 | 2.900 | 3 | 6 | 17 | negative regulation of ion transmembrane transporter activity |
| GO:0036336 | 0.041 | 2.900 | 3 | 6 | 17 | dendritic cell migration |
| GO:0048713 | 0.041 | 2.900 | 3 | 6 | 17 | regulation of oligodendrocyte differentiation |
| GO:0050732 | 0.041 | 2.900 | 3 | 6 | 17 | negative regulation of peptidyl-tyrosine phosphorylation |
| GO:0055012 | 0.041 | 2.900 | 3 | 6 | 17 | ventricular cardiac muscle cell differentiation |
| GO:2000113 | 0.041 | 1.216 | 100 | 116 | 630 | negative regulation of cellular macromolecule biosynthetic process |
| GO:0002016 | 0.041 | 4.251 | 1 | 4 | 9 | regulation of blood volume by renin-angiotensin |
| GO:0002467 | 0.041 | 4.251 | 1 | 4 | 9 | germinal center formation |
| GO:0002643 | 0.041 | 4.251 | 1 | 4 | 9 | regulation of tolerance induction |
| GO:0002645 | 0.041 | 4.251 | 1 | 4 | 9 | positive regulation of tolerance induction |
| GO:0007016 | 0.041 | 4.251 | 1 | 4 | 9 | cytoskeletal anchoring at plasma membrane |
| GO:0010544 | 0.041 | 4.251 | 1 | 4 | 9 | negative regulation of platelet activation |
| GO:0010762 | 0.041 | 4.251 | 1 | 4 | 9 | regulation of fibroblast migration |
| GO:0030730 | 0.041 | 4.251 | 1 | 4 | 9 | sequestering of triglyceride |
| GO:0032836 | 0.041 | 4.251 | 1 | 4 | 9 | glomerular basement membrane development |
| GO:0034433 | 0.041 | 4.251 | 1 | 4 | 9 | steroid esterification |
| GO:0034434 | 0.041 | 4.251 | 1 | 4 | 9 | sterol esterification |
| GO:0034435 | 0.041 | 4.251 | 1 | 4 | 9 | cholesterol esterification |
| GO:0042572 | 0.041 | 4.251 | 1 | 4 | 9 | retinol metabolic process |
| GO:0042987 | 0.041 | 4.251 | 1 | 4 | 9 | amyloid precursor protein catabolic process |
| GO:0044319 | 0.041 | 4.251 | 1 | 4 | 9 | wound healing, spreading of cells |
| GO:0045408 | 0.041 | 4.251 | 1 | 4 | 9 | regulation of interleukin-6 biosynthetic process |
| GO:0045601 | 0.041 | 4.251 | 1 | 4 | 9 | regulation of endothelial cell differentiation |
| GO:0046629 | 0.041 | 4.251 | 1 | 4 | 9 | gamma-delta T cell activation |
| GO:0051642 | 0.041 | 4.251 | 1 | 4 | 9 | centrosome localization |
| GO:0051767 | 0.041 | 4.251 | 1 | 4 | 9 | nitric-oxide synthase biosynthetic process |
| GO:0051769 | 0.041 | 4.251 | 1 | 4 | 9 | regulation of nitric-oxide synthase biosynthetic process |
| GO:0055094 | 0.041 | 4.251 | 1 | 4 | 9 | response to lipoprotein particle stimulus |
| GO:0070102 | 0.041 | 4.251 | 1 | 4 | 9 | interleukin-6-mediated signaling pathway |
| GO:0070486 | 0.041 | 4.251 | 1 | 4 | 9 | leukocyte aggregation |
| GO:0090504 | 0.041 | 4.251 | 1 | 4 | 9 | epiboly |
| GO:0090505 | 0.041 | 4.251 | 1 | 4 | 9 | epiboly involved in wound healing |
| GO:0051259 | 0.041 | 1.368 | 36 | 46 | 226 | protein oligomerization |
| GO:0002824 | 0.041 | 2.129 | 6 | 10 | 35 | positive regulation of adaptive immune response based on somatic recombination of immune receptors built from immunoglobulin superfamily domains |
| GO:0006953 | 0.041 | 2.129 | 6 | 10 | 35 | acute-phase response |
| GO:0010001 | 0.042 | 1.562 | 17 | 24 | 106 | glial cell differentiation |
| GO:0001657 | 0.042 | 1.661 | 13 | 19 | 80 | ureteric bud development |
| GO:0033158 | 0.042 | 3.322 | 2 | 5 | 13 | regulation of protein import into nucleus, translocation |
| GO:0035304 | 0.042 | 3.322 | 2 | 5 | 13 | regulation of protein dephosphorylation |
| GO:0043537 | 0.042 | 3.322 | 2 | 5 | 13 | negative regulation of blood vessel endothelial cell migration |
| GO:1901069 | 0.042 | 1.368 | 35 | 45 | 221 | guanosine-containing compound catabolic process |
| GO:0019835 | 0.043 | 2.364 | 4 | 8 | 26 | cytolysis |
| GO:0070830 | 0.043 | 2.364 | 4 | 8 | 26 | tight junction assembly |
| GO:0006942 | 0.044 | 1.871 | 8 | 13 | 50 | regulation of striated muscle contraction |
| GO:0071356 | 0.044 | 1.871 | 8 | 13 | 50 | cellular response to tumor necrosis factor |
| GO:0010594 | 0.045 | 1.776 | 10 | 15 | 60 | regulation of endothelial cell migration |
| GO:0060562 | 0.045 | 1.378 | 33 | 42 | 205 | epithelial tube morphogenesis |
| GO:0010863 | 0.045 | 1.819 | 9 | 14 | 55 | positive regulation of phospholipase C activity |
| GO:0030010 | 0.045 | 1.819 | 9 | 14 | 55 | establishment of cell polarity |
| GO:0043154 | 0.045 | 1.819 | 9 | 14 | 55 | negative regulation of cysteine-type endopeptidase activity involved in apoptotic process |
| GO:0010557 | 0.045 | 1.182 | 136 | 154 | 858 | positive regulation of macromolecule biosynthetic process |
| GO:0060560 | 0.045 | 1.600 | 14 | 21 | 91 | developmental growth involved in morphogenesis |
| GO:0010638 | 0.045 | 1.431 | 26 | 34 | 161 | positive regulation of organelle organization |
| GO:0003205 | 0.046 | 1.616 | 14 | 20 | 86 | cardiac chamber development |
| GO:0015909 | 0.047 | 2.177 | 5 | 9 | 31 | long-chain fatty acid transport |
| GO:0055024 | 0.047 | 2.177 | 5 | 9 | 31 | regulation of cardiac muscle tissue development |
| GO:0032318 | 0.047 | 1.553 | 16 | 23 | 102 | regulation of Ras GTPase activity |
| GO:0045058 | 0.048 | 2.482 | 3 | 7 | 22 | T cell selection |
| GO:0045620 | 0.048 | 2.482 | 3 | 7 | 22 | negative regulation of lymphocyte differentiation |
| GO:0016197 | 0.048 | 1.654 | 12 | 18 | 76 | endosomal transport |
| GO:0031647 | 0.048 | 1.654 | 12 | 18 | 76 | regulation of protein stability |
| GO:0007519 | 0.049 | 1.456 | 22 | 30 | 140 | skeletal muscle tissue development |
| GO:0002705 | 0.049 | 2.047 | 6 | 10 | 36 | positive regulation of leukocyte mediated immunity |
| GO:0002708 | 0.049 | 2.047 | 6 | 10 | 36 | positive regulation of lymphocyte mediated immunity |
| GO:0051896 | 0.049 | 1.678 | 11 | 17 | 71 | regulation of protein kinase B signaling cascade |
| GO:0045935 | 0.050 | 1.176 | 137 | 154 | 861 | positive regulation of nucleobase-containing compound metabolic process |
| GO:0045934 | 0.050 | 1.202 | 102 | 117 | 641 | negative regulation of nucleobase-containing compound metabolic process |
