# Supplementary material for: Hidden Treasures in “Ancient” Microarrays: Gene-Expression Portrays Biology and Potential Resistance Pathways of Major Lung Cancer Subtypes and Normal Tissue
Source: Front Oncol. 2014 Sep 29;4:251. doi: 10.3389/fonc.2014.00251 (PMC4178426; doi:10.3389/fonc.2014.00251)
Supplement: Figure S1 — Clinically relevant differentially expressed pathways in each tissue type versus the rest. The pathway maps are arranged according to the scheme in the left-top corner. Red indicates overexpression in genes, dark green downregulation. In the Cell Cycle pathway, tumor suppressor genes were overexpressed in normal lung tissue while oncogenes and tumor driving cyclins were overexpressed in cancers. The gene thymidylate synthase (TYMS or TS, belonging to the pathway “one carbon pool by folate”) is relevant for tumor growth and is also a treatment target. Notably, TYMS was overexpressed only in the tumors that are generally refractory to the drug pemetrexed, as the squamous and the small-cell lung cancer. TYMS was not overexpressed in mesothelioma, but it is known that TYMS expression is highly variable in this cancer. In the ERBB pathway, the ERBB2/HER2 and ERBB3/HER3 were overexpressed in adenocarcinoma, while the ERBB4 was overexpressed in the carcinoids. [file Data_Sheet_1.ZIP › File S3.ZIP!/GO Analysis/Normals vs Rest Enrichment Analysis/Underrepresented GO terms of Differentially Expressed Genes.html]

Gene to GO BP test for under-representation

| GOBPID | Pvalue | OddsRatio | ExpCount | Count | Size | Term |
| --- | --- | --- | --- | --- | --- | --- |
| GO:0016071 | <0.0001 | 0.630 | 146 | 108 | 408 | mRNA metabolic process |
| GO:0009914 | <0.0001 | 0.491 | 61 | 37 | 170 | hormone transport |
| GO:0046887 | <0.0001 | 0.227 | 19 | 6 | 53 | positive regulation of hormone secretion |
| GO:0046879 | <0.0001 | 0.511 | 59 | 37 | 165 | hormone secretion |
| GO:0046883 | <0.0001 | 0.486 | 52 | 31 | 144 | regulation of hormone secretion |
| GO:0043632 | <0.0001 | 0.600 | 90 | 64 | 252 | modification-dependent macromolecule catabolic process |
| GO:0051603 | <0.0001 | 0.611 | 96 | 69 | 268 | proteolysis involved in cellular protein catabolic process |
| GO:0019941 | <0.0001 | 0.607 | 90 | 64 | 250 | modification-dependent protein catabolic process |
| GO:0030073 | <0.0001 | 0.483 | 45 | 27 | 126 | insulin secretion |
| GO:0006511 | <0.0001 | 0.608 | 88 | 63 | 246 | ubiquitin-dependent protein catabolic process |
| GO:0030072 | <0.0001 | 0.509 | 50 | 31 | 139 | peptide hormone secretion |
| GO:0042886 | <0.0001 | 0.536 | 56 | 36 | 155 | amide transport |
| GO:0003001 | <0.0001 | 0.625 | 93 | 68 | 260 | generation of a signal involved in cell-cell signaling |
| GO:0023061 | <0.0001 | 0.625 | 93 | 68 | 260 | signal release |
| GO:0015833 | <0.0001 | 0.528 | 53 | 34 | 148 | peptide transport |
| GO:0044265 | 0.001 | 0.708 | 166 | 133 | 463 | cellular macromolecule catabolic process |
| GO:0000184 | 0.001 | 0.418 | 30 | 16 | 84 | nuclear-transcribed mRNA catabolic process, nonsense-mediated decay |
| GO:0002790 | 0.001 | 0.532 | 51 | 33 | 143 | peptide secretion |
| GO:0044257 | 0.001 | 0.647 | 99 | 74 | 276 | cellular protein catabolic process |
| GO:0010817 | 0.001 | 0.653 | 98 | 74 | 274 | regulation of hormone levels |
| GO:0090276 | 0.001 | 0.523 | 44 | 28 | 123 | regulation of peptide hormone secretion |
| GO:0002793 | 0.001 | 0.262 | 14 | 5 | 39 | positive regulation of peptide secretion |
| GO:0072329 | 0.001 | 0.395 | 24 | 12 | 66 | monocarboxylic acid catabolic process |
| GO:0002791 | 0.002 | 0.536 | 45 | 29 | 125 | regulation of peptide secretion |
| GO:0090087 | 0.002 | 0.536 | 45 | 29 | 125 | regulation of peptide transport |
| GO:0007601 | 0.002 | 0.551 | 48 | 32 | 135 | visual perception |
| GO:0050953 | 0.002 | 0.551 | 48 | 32 | 135 | sensory perception of light stimulus |
| GO:0090277 | 0.002 | 0.270 | 14 | 5 | 38 | positive regulation of peptide hormone secretion |
| GO:0000956 | 0.002 | 0.525 | 41 | 26 | 114 | nuclear-transcribed mRNA catabolic process |
| GO:0050796 | 0.002 | 0.525 | 41 | 26 | 114 | regulation of insulin secretion |
| GO:0070972 | 0.003 | 0.490 | 32 | 19 | 88 | protein localization to endoplasmic reticulum |
| GO:0050877 | 0.003 | 0.805 | 298 | 262 | 832 | neurological system process |
| GO:0006613 | 0.003 | 0.467 | 28 | 16 | 77 | cotranslational protein targeting to membrane |
| GO:0007268 | 0.003 | 0.757 | 172 | 144 | 479 | synaptic transmission |
| GO:0045861 | 0.004 | 0.223 | 10 | 3 | 27 | negative regulation of proteolysis |
| GO:0071705 | 0.004 | 0.741 | 143 | 118 | 399 | nitrogen compound transport |
| GO:0032024 | 0.004 | 0.264 | 11 | 4 | 31 | positive regulation of insulin secretion |
| GO:0044248 | 0.005 | 0.837 | 411 | 372 | 1148 | cellular catabolic process |
| GO:0006614 | 0.005 | 0.483 | 27 | 16 | 75 | SRP-dependent cotranslational protein targeting to membrane |
| GO:0009062 | 0.005 | 0.426 | 20 | 11 | 57 | fatty acid catabolic process |
| GO:0072599 | 0.005 | 0.496 | 28 | 17 | 78 | establishment of protein localization to endoplasmic reticulum |
| GO:0006415 | 0.005 | 0.454 | 23 | 13 | 64 | translational termination |
| GO:0006414 | 0.005 | 0.477 | 25 | 15 | 71 | translational elongation |
| GO:0007267 | 0.005 | 0.815 | 290 | 257 | 810 | cell-cell signaling |
| GO:0006508 | 0.006 | 0.788 | 209 | 180 | 582 | proteolysis |
| GO:0019226 | 0.006 | 0.784 | 198 | 170 | 552 | transmission of nerve impulse |
| GO:0046888 | 0.006 | 0.307 | 12 | 5 | 34 | negative regulation of hormone secretion |
| GO:0006402 | 0.006 | 0.586 | 43 | 30 | 121 | mRNA catabolic process |
| GO:0009057 | 0.006 | 0.795 | 215 | 187 | 601 | macromolecule catabolic process |
| GO:0045047 | 0.007 | 0.504 | 28 | 17 | 77 | protein targeting to ER |
| GO:0090278 | 0.007 | 0.188 | 8 | 2 | 21 | negative regulation of peptide hormone secretion |
| GO:0007032 | 0.008 | <0.0001 | 4 | 0 | 11 | endosome organization |
| GO:0030238 | 0.008 | <0.0001 | 4 | 0 | 11 | male sex determination |
| GO:0032204 | 0.008 | <0.0001 | 4 | 0 | 11 | regulation of telomere maintenance |
| GO:0016236 | 0.008 | 0.243 | 9 | 3 | 25 | macroautophagy |
| GO:0006413 | 0.008 | 0.586 | 40 | 28 | 113 | translational initiation |
| GO:0006476 | 0.008 | 0.319 | 12 | 5 | 33 | protein deacetylation |
| GO:0007031 | 0.010 | 0.198 | 7 | 2 | 20 | peroxisome organization |
| GO:0006112 | 0.010 | 0.628 | 48 | 35 | 134 | energy reserve metabolic process |
| GO:0072404 | 0.010 | 0.483 | 22 | 13 | 61 | signal transduction involved in G1/S transition checkpoint |
| GO:0007602 | 0.011 | 0.297 | 10 | 4 | 28 | phototransduction |
| GO:0016575 | 0.011 | 0.297 | 10 | 4 | 28 | histone deacetylation |
| GO:0035637 | 0.011 | 0.805 | 205 | 179 | 571 | multicellular organismal signaling |
| GO:0071158 | 0.011 | 0.518 | 25 | 16 | 71 | positive regulation of cell cycle arrest |
| GO:0016054 | 0.012 | 0.648 | 52 | 39 | 146 | organic acid catabolic process |
| GO:0046395 | 0.012 | 0.648 | 52 | 39 | 146 | carboxylic acid catabolic process |
| GO:0071779 | 0.012 | 0.531 | 27 | 17 | 74 | G1/S transition checkpoint |
| GO:0006635 | 0.013 | 0.434 | 16 | 9 | 46 | fatty acid beta-oxidation |
| GO:0006977 | 0.013 | 0.493 | 22 | 13 | 60 | DNA damage response, signal transduction by p53 class mediator resulting in cell cycle arrest |
| GO:0072413 | 0.013 | 0.493 | 22 | 13 | 60 | signal transduction involved in mitotic cell cycle checkpoint |
| GO:0072431 | 0.013 | 0.493 | 22 | 13 | 60 | signal transduction involved in mitotic cell cycle G1/S transition DNA damage checkpoint |
| GO:0072474 | 0.013 | 0.493 | 22 | 13 | 60 | signal transduction involved in mitotic cell cycle G1/S checkpoint |
| GO:0070647 | 0.013 | 0.771 | 132 | 112 | 369 | protein modification by small protein conjugation or removal |
| GO:0006396 | 0.013 | 0.773 | 134 | 114 | 375 | RNA processing |
| GO:0031575 | 0.014 | 0.528 | 25 | 16 | 70 | mitotic cell cycle G1/S transition checkpoint |
| GO:0046173 | 0.014 | 0.210 | 7 | 2 | 19 | polyol biosynthetic process |
| GO:0009056 | 0.014 | 0.869 | 478 | 443 | 1335 | catabolic process |
| GO:0031571 | 0.014 | 0.509 | 23 | 14 | 63 | mitotic cell cycle G1/S transition DNA damage checkpoint |
| GO:0034284 | 0.014 | 0.585 | 33 | 23 | 93 | response to monosaccharide stimulus |
| GO:0030163 | 0.015 | 0.769 | 125 | 106 | 350 | protein catabolic process |
| GO:0006775 | 0.015 | 0.268 | 8 | 3 | 23 | fat-soluble vitamin metabolic process |
| GO:0035567 | 0.015 | 0.268 | 8 | 3 | 23 | non-canonical Wnt receptor signaling pathway |
| GO:0055114 | 0.015 | 0.781 | 140 | 120 | 392 | oxidation-reduction process |
| GO:0006401 | 0.015 | 0.651 | 49 | 37 | 138 | RNA catabolic process |
| GO:0008202 | 0.015 | 0.712 | 76 | 61 | 213 | steroid metabolic process |
| GO:1901575 | 0.016 | 0.868 | 448 | 414 | 1249 | organic substance catabolic process |
| GO:0035601 | 0.017 | 0.382 | 12 | 6 | 34 | protein deacylation |
| GO:0007608 | 0.018 | 0.137 | 5 | 1 | 14 | sensory perception of smell |
| GO:0032891 | 0.018 | 0.137 | 5 | 1 | 14 | negative regulation of organic acid transport |
| GO:0042359 | 0.018 | 0.137 | 5 | 1 | 14 | vitamin D metabolic process |
| GO:0072395 | 0.018 | 0.520 | 22 | 14 | 62 | signal transduction involved in cell cycle checkpoint |
| GO:0010498 | 0.018 | 0.687 | 59 | 46 | 165 | proteasomal protein catabolic process |
| GO:0006605 | 0.018 | 0.775 | 124 | 105 | 345 | protein targeting |
| GO:0001573 | 0.018 | <0.0001 | 3 | 0 | 9 | ganglioside metabolic process |
| GO:0016241 | 0.018 | <0.0001 | 3 | 0 | 9 | regulation of macroautophagy |
| GO:0032106 | 0.018 | <0.0001 | 3 | 0 | 9 | positive regulation of response to extracellular stimulus |
| GO:0032109 | 0.018 | <0.0001 | 3 | 0 | 9 | positive regulation of response to nutrient levels |
| GO:0032274 | 0.018 | <0.0001 | 3 | 0 | 9 | gonadotropin secretion |
| GO:0060123 | 0.018 | <0.0001 | 3 | 0 | 9 | regulation of growth hormone secretion |
| GO:0007626 | 0.019 | 0.629 | 40 | 29 | 111 | locomotory behavior |
| GO:0006397 | 0.019 | 0.745 | 92 | 76 | 257 | mRNA processing |
| GO:0042461 | 0.020 | 0.325 | 9 | 4 | 26 | photoreceptor cell development |
| GO:0030433 | 0.020 | 0.223 | 6 | 2 | 18 | ER-associated protein catabolic process |
| GO:0051955 | 0.020 | 0.223 | 6 | 2 | 18 | regulation of amino acid transport |
| GO:0002792 | 0.020 | 0.282 | 8 | 3 | 22 | negative regulation of peptide secretion |
| GO:0032845 | 0.020 | 0.282 | 8 | 3 | 22 | negative regulation of homeostatic process |
| GO:0042462 | 0.020 | 0.282 | 8 | 3 | 22 | eye photoreceptor cell development |
| GO:0072401 | 0.022 | 0.531 | 22 | 14 | 61 | signal transduction involved in DNA integrity checkpoint |
| GO:0072422 | 0.022 | 0.531 | 22 | 14 | 61 | signal transduction involved in DNA damage checkpoint |
| GO:0009583 | 0.022 | 0.397 | 12 | 6 | 33 | detection of light stimulus |
| GO:0043161 | 0.023 | 0.692 | 56 | 44 | 157 | proteasomal ubiquitin-dependent protein catabolic process |
| GO:0015980 | 0.023 | 0.749 | 88 | 73 | 246 | energy derivation by oxidation of organic compounds |
| GO:0016042 | 0.023 | 0.699 | 58 | 46 | 163 | lipid catabolic process |
| GO:0007600 | 0.024 | 0.768 | 103 | 87 | 288 | sensory perception |
| GO:0009746 | 0.024 | 0.612 | 32 | 23 | 90 | response to hexose stimulus |
| GO:0071702 | 0.025 | 0.879 | 454 | 423 | 1267 | organic substance transport |
| GO:0006612 | 0.025 | 0.645 | 39 | 29 | 109 | protein targeting to membrane |
| GO:0033365 | 0.026 | 0.795 | 131 | 113 | 365 | protein localization to organelle |
| GO:0002360 | 0.026 | 0.149 | 5 | 1 | 13 | T cell lineage commitment |
| GO:0031063 | 0.026 | 0.149 | 5 | 1 | 13 | regulation of histone deacetylation |
| GO:0051000 | 0.026 | 0.149 | 5 | 1 | 13 | positive regulation of nitric-oxide synthase activity |
| GO:0038127 | 0.026 | 0.674 | 47 | 36 | 131 | ERBB signaling pathway |
| GO:0030534 | 0.028 | 0.612 | 31 | 22 | 86 | adult behavior |
| GO:0046676 | 0.028 | 0.238 | 6 | 2 | 17 | negative regulation of insulin secretion |
| GO:0051954 | 0.028 | 0.238 | 6 | 2 | 17 | positive regulation of amine transport |
| GO:0060071 | 0.028 | 0.238 | 6 | 2 | 17 | Wnt receptor signaling pathway, planar cell polarity pathway |
| GO:0090175 | 0.028 | 0.238 | 6 | 2 | 17 | regulation of establishment of planar polarity |
| GO:0000209 | 0.028 | 0.668 | 43 | 33 | 121 | protein polyubiquitination |
| GO:0006521 | 0.028 | 0.522 | 19 | 12 | 53 | regulation of cellular amino acid metabolic process |
| GO:0018298 | 0.029 | <0.0001 | 3 | 0 | 8 | protein-chromophore linkage |
| GO:0032205 | 0.029 | <0.0001 | 3 | 0 | 8 | negative regulation of telomere maintenance |
| GO:0032276 | 0.029 | <0.0001 | 3 | 0 | 8 | regulation of gonadotropin secretion |
| GO:0032959 | 0.029 | <0.0001 | 3 | 0 | 8 | inositol trisphosphate biosynthetic process |
| GO:0044068 | 0.029 | <0.0001 | 3 | 0 | 8 | modulation by symbiont of host cellular process |
| GO:0051956 | 0.029 | <0.0001 | 3 | 0 | 8 | negative regulation of amino acid transport |
| GO:0070841 | 0.029 | <0.0001 | 3 | 0 | 8 | inclusion body assembly |
| GO:0008206 | 0.029 | 0.412 | 11 | 6 | 32 | bile acid metabolic process |
| GO:0042490 | 0.029 | 0.412 | 11 | 6 | 32 | mechanoreceptor differentiation |
| GO:0048608 | 0.029 | 0.736 | 71 | 58 | 198 | reproductive structure development |
| GO:0061458 | 0.029 | 0.736 | 71 | 58 | 198 | reproductive system development |
| GO:0016567 | 0.032 | 0.788 | 111 | 95 | 309 | protein ubiquitination |
| GO:0009749 | 0.034 | 0.631 | 32 | 23 | 88 | response to glucose stimulus |
| GO:0007173 | 0.034 | 0.689 | 46 | 36 | 129 | epidermal growth factor receptor signaling pathway |
| GO:0044242 | 0.034 | 0.662 | 38 | 29 | 107 | cellular lipid catabolic process |
| GO:0019098 | 0.034 | 0.535 | 19 | 12 | 52 | reproductive behavior |
| GO:0032104 | 0.035 | 0.357 | 9 | 4 | 24 | regulation of response to extracellular stimulus |
| GO:0032107 | 0.035 | 0.357 | 9 | 4 | 24 | regulation of response to nutrient levels |
| GO:0017038 | 0.036 | 0.739 | 66 | 54 | 184 | protein import |
| GO:0070646 | 0.036 | 0.510 | 16 | 10 | 45 | protein modification by small protein removal |
| GO:0006278 | 0.037 | 0.162 | 4 | 1 | 12 | RNA-dependent DNA replication |
| GO:0010460 | 0.037 | 0.162 | 4 | 1 | 12 | positive regulation of heart rate |
| GO:0014048 | 0.037 | 0.162 | 4 | 1 | 12 | regulation of glutamate secretion |
| GO:0032105 | 0.037 | 0.162 | 4 | 1 | 12 | negative regulation of response to extracellular stimulus |
| GO:0032108 | 0.037 | 0.162 | 4 | 1 | 12 | negative regulation of response to nutrient levels |
| GO:0045494 | 0.037 | 0.162 | 4 | 1 | 12 | photoreceptor cell maintenance |
| GO:0046135 | 0.037 | 0.162 | 4 | 1 | 12 | pyrimidine nucleoside catabolic process |
| GO:0044704 | 0.038 | 0.476 | 14 | 8 | 38 | single-organism reproductive behavior |
| GO:0035773 | 0.038 | 0.315 | 7 | 3 | 20 | insulin secretion involved in cellular response to glucose stimulus |
| GO:0051865 | 0.038 | 0.315 | 7 | 3 | 20 | protein autoubiquitination |
| GO:0061178 | 0.038 | 0.315 | 7 | 3 | 20 | regulation of insulin secretion involved in cellular response to glucose stimulus |
| GO:0044764 | 0.038 | 0.841 | 194 | 174 | 540 | multi-organism cellular process |
| GO:0030162 | 0.038 | 0.688 | 44 | 34 | 122 | regulation of proteolysis |
| GO:1901617 | 0.038 | 0.693 | 45 | 35 | 125 | organic hydroxy compound biosynthetic process |
| GO:0008406 | 0.041 | 0.712 | 50 | 40 | 140 | gonad development |
| GO:0060113 | 0.042 | 0.406 | 10 | 5 | 27 | inner ear receptor cell differentiation |
| GO:0008380 | 0.042 | 0.762 | 75 | 63 | 210 | RNA splicing |
| GO:0031645 | 0.043 | 0.463 | 12 | 7 | 34 | negative regulation of neurological system process |
| GO:0016482 | 0.043 | 0.842 | 181 | 163 | 506 | cytoplasmic transport |
| GO:0016032 | 0.043 | 0.846 | 193 | 174 | 538 | viral reproduction |
| GO:0051170 | 0.045 | 0.741 | 61 | 50 | 170 | nuclear import |
| GO:0001787 | 0.045 | <0.0001 | 3 | 0 | 7 | natural killer cell proliferation |
| GO:0007442 | 0.045 | <0.0001 | 3 | 0 | 7 | hindgut morphogenesis |
| GO:0008343 | 0.045 | <0.0001 | 3 | 0 | 7 | adult feeding behavior |
| GO:0014051 | 0.045 | <0.0001 | 3 | 0 | 7 | gamma-aminobutyric acid secretion |
| GO:0014887 | 0.045 | <0.0001 | 3 | 0 | 7 | cardiac muscle adaptation |
| GO:0015812 | 0.045 | <0.0001 | 3 | 0 | 7 | gamma-aminobutyric acid transport |
| GO:0032906 | 0.045 | <0.0001 | 3 | 0 | 7 | transforming growth factor beta2 production |
| GO:0032909 | 0.045 | <0.0001 | 3 | 0 | 7 | regulation of transforming growth factor beta2 production |
| GO:0033079 | 0.045 | <0.0001 | 3 | 0 | 7 | immature T cell proliferation |
| GO:0035774 | 0.045 | <0.0001 | 3 | 0 | 7 | positive regulation of insulin secretion involved in cellular response to glucose stimulus |
| GO:0042095 | 0.045 | <0.0001 | 3 | 0 | 7 | interferon-gamma biosynthetic process |
| GO:0046133 | 0.045 | <0.0001 | 3 | 0 | 7 | pyrimidine ribonucleoside catabolic process |
| GO:0046548 | 0.045 | <0.0001 | 3 | 0 | 7 | retinal rod cell development |
| GO:0048505 | 0.045 | <0.0001 | 3 | 0 | 7 | regulation of timing of cell differentiation |
| GO:0060124 | 0.045 | <0.0001 | 3 | 0 | 7 | positive regulation of growth hormone secretion |
| GO:0071108 | 0.045 | <0.0001 | 3 | 0 | 7 | protein K48-linked deubiquitination |
| GO:0009566 | 0.045 | 0.566 | 19 | 13 | 54 | fertilization |
| GO:0071326 | 0.045 | 0.525 | 16 | 10 | 44 | cellular response to monosaccharide stimulus |
| GO:0007628 | 0.046 | 0.376 | 8 | 4 | 23 | adult walking behavior |
| GO:0043647 | 0.046 | 0.376 | 8 | 4 | 23 | inositol phosphate metabolic process |
| GO:0044708 | 0.047 | 0.791 | 94 | 81 | 263 | single-organism behavior |
| GO:0034440 | 0.047 | 0.617 | 25 | 18 | 70 | lipid oxidation |
| GO:0051650 | 0.047 | 0.493 | 13 | 8 | 37 | establishment of vesicle localization |
| GO:0051952 | 0.048 | 0.545 | 17 | 11 | 47 | regulation of amine transport |
