# Supplementary material for: Hidden Treasures in “Ancient” Microarrays: Gene-Expression Portrays Biology and Potential Resistance Pathways of Major Lung Cancer Subtypes and Normal Tissue
Source: Front Oncol. 2014 Sep 29;4:251. doi: 10.3389/fonc.2014.00251 (PMC4178426; doi:10.3389/fonc.2014.00251)
Supplement: Figure S1 — Clinically relevant differentially expressed pathways in each tissue type versus the rest. The pathway maps are arranged according to the scheme in the left-top corner. Red indicates overexpression in genes, dark green downregulation. In the Cell Cycle pathway, tumor suppressor genes were overexpressed in normal lung tissue while oncogenes and tumor driving cyclins were overexpressed in cancers. The gene thymidylate synthase (TYMS or TS, belonging to the pathway “one carbon pool by folate”) is relevant for tumor growth and is also a treatment target. Notably, TYMS was overexpressed only in the tumors that are generally refractory to the drug pemetrexed, as the squamous and the small-cell lung cancer. TYMS was not overexpressed in mesothelioma, but it is known that TYMS expression is highly variable in this cancer. In the ERBB pathway, the ERBB2/HER2 and ERBB3/HER3 were overexpressed in adenocarcinoma, while the ERBB4 was overexpressed in the carcinoids. [file Data_Sheet_1.ZIP › File S3.ZIP!/GO Analysis/Normals vs Rest Enrichment Analysis/Underrepresented GO terms of Down-regulated Genes.html]

Gene to GO BP test for under-representation

| GOBPID | Pvalue | OddsRatio | ExpCount | Count | Size | Term |
| --- | --- | --- | --- | --- | --- | --- |
| GO:0006955 | <0.0001 | 0.457 | 157 | 85 | 785 | immune response |
| GO:0006952 | <0.0001 | 0.478 | 160 | 90 | 801 | defense response |
| GO:0007154 | <0.0001 | 0.674 | 637 | 521 | 3192 | cell communication |
| GO:0023052 | <0.0001 | 0.673 | 628 | 512 | 3144 | signaling |
| GO:0044700 | <0.0001 | 0.673 | 628 | 512 | 3144 | single organism signaling |
| GO:0048583 | <0.0001 | 0.608 | 320 | 228 | 1602 | regulation of response to stimulus |
| GO:0007165 | <0.0001 | 0.678 | 562 | 454 | 2814 | signal transduction |
| GO:0050896 | <0.0001 | 0.699 | 856 | 744 | 4288 | response to stimulus |
| GO:0009611 | <0.0001 | 0.542 | 171 | 107 | 855 | response to wounding |
| GO:0009605 | <0.0001 | 0.576 | 193 | 128 | 969 | response to external stimulus |
| GO:0006954 | <0.0001 | 0.398 | 75 | 35 | 376 | inflammatory response |
| GO:0002376 | <0.0001 | 0.634 | 270 | 196 | 1352 | immune system process |
| GO:0001775 | <0.0001 | 0.500 | 120 | 69 | 599 | cell activation |
| GO:0010646 | <0.0001 | 0.643 | 281 | 207 | 1410 | regulation of cell communication |
| GO:0007166 | <0.0001 | 0.669 | 332 | 254 | 1664 | cell surface receptor signaling pathway |
| GO:0023051 | <0.0001 | 0.653 | 281 | 209 | 1408 | regulation of signaling |
| GO:0002682 | <0.0001 | 0.541 | 129 | 80 | 648 | regulation of immune system process |
| GO:0009966 | <0.0001 | 0.646 | 245 | 179 | 1225 | regulation of signal transduction |
| GO:0035556 | <0.0001 | 0.661 | 261 | 195 | 1307 | intracellular signal transduction |
| GO:0045087 | <0.0001 | 0.455 | 78 | 41 | 391 | innate immune response |
| GO:0048584 | <0.0001 | 0.603 | 170 | 116 | 852 | positive regulation of response to stimulus |
| GO:0006629 | <0.0001 | 0.580 | 148 | 97 | 739 | lipid metabolic process |
| GO:0032879 | <0.0001 | 0.633 | 202 | 144 | 1010 | regulation of localization |
| GO:0016192 | <0.0001 | 0.556 | 122 | 77 | 611 | vesicle-mediated transport |
| GO:0032940 | <0.0001 | 0.525 | 102 | 61 | 511 | secretion by cell |
| GO:0002684 | <0.0001 | 0.487 | 84 | 47 | 422 | positive regulation of immune system process |
| GO:0002252 | <0.0001 | 0.443 | 68 | 35 | 343 | immune effector process |
| GO:0051239 | <0.0001 | 0.678 | 262 | 200 | 1315 | regulation of multicellular organismal process |
| GO:0046903 | <0.0001 | 0.557 | 117 | 74 | 587 | secretion |
| GO:0051716 | <0.0001 | 0.763 | 674 | 592 | 3376 | cellular response to stimulus |
| GO:0042221 | <0.0001 | 0.735 | 415 | 343 | 2077 | response to chemical stimulus |
| GO:0030334 | <0.0001 | 0.457 | 65 | 34 | 325 | regulation of cell migration |
| GO:0040012 | <0.0001 | 0.482 | 73 | 40 | 364 | regulation of locomotion |
| GO:0032101 | <0.0001 | 0.449 | 62 | 32 | 311 | regulation of response to external stimulus |
| GO:2000145 | <0.0001 | 0.474 | 68 | 37 | 342 | regulation of cell motility |
| GO:0019932 | <0.0001 | 0.210 | 24 | 6 | 119 | second-messenger-mediated signaling |
| GO:0050865 | <0.0001 | 0.434 | 56 | 28 | 281 | regulation of cell activation |
| GO:0045321 | <0.0001 | 0.527 | 85 | 51 | 428 | leukocyte activation |
| GO:0010627 | <0.0001 | 0.570 | 103 | 66 | 516 | regulation of intracellular protein kinase cascade |
| GO:0002576 | <0.0001 | 0.106 | 15 | 2 | 77 | platelet degranulation |
| GO:0007243 | <0.0001 | 0.611 | 130 | 89 | 653 | intracellular protein kinase cascade |
| GO:0044707 | <0.0001 | 0.780 | 710 | 634 | 3559 | single-multicellular organism process |
| GO:0040011 | <0.0001 | 0.668 | 190 | 141 | 951 | locomotion |
| GO:0022610 | <0.0001 | 0.620 | 136 | 94 | 680 | biological adhesion |
| GO:0031347 | <0.0001 | 0.491 | 66 | 37 | 332 | regulation of defense response |
| GO:0007155 | <0.0001 | 0.622 | 136 | 94 | 679 | cell adhesion |
| GO:0051270 | <0.0001 | 0.523 | 76 | 45 | 381 | regulation of cellular component movement |
| GO:0050776 | <0.0001 | 0.537 | 81 | 49 | 405 | regulation of immune response |
| GO:0050900 | <0.0001 | 0.413 | 44 | 21 | 221 | leukocyte migration |
| GO:0051179 | <0.0001 | 0.777 | 542 | 472 | 2714 | localization |
| GO:0001816 | <0.0001 | 0.493 | 62 | 35 | 313 | cytokine production |
| GO:0006810 | <0.0001 | 0.763 | 429 | 364 | 2148 | transport |
| GO:0050817 | <0.0001 | 0.547 | 81 | 50 | 407 | coagulation |
| GO:0007596 | <0.0001 | 0.550 | 81 | 50 | 405 | blood coagulation |
| GO:0044255 | <0.0001 | 0.596 | 104 | 69 | 520 | cellular lipid metabolic process |
| GO:0006897 | <0.0001 | 0.471 | 54 | 29 | 271 | endocytosis |
| GO:0044765 | <0.0001 | 0.753 | 362 | 302 | 1814 | single-organism transport |
| GO:0048585 | <0.0001 | 0.606 | 110 | 74 | 549 | negative regulation of response to stimulus |
| GO:0042060 | <0.0001 | 0.591 | 99 | 65 | 494 | wound healing |
| GO:0002694 | <0.0001 | 0.464 | 51 | 27 | 256 | regulation of leukocyte activation |
| GO:0010647 | <0.0001 | 0.633 | 127 | 89 | 635 | positive regulation of cell communication |
| GO:0051049 | <0.0001 | 0.654 | 146 | 106 | 733 | regulation of transport |
| GO:0065008 | <0.0001 | 0.759 | 368 | 309 | 1845 | regulation of biological quality |
| GO:0032501 | <0.0001 | 0.797 | 731 | 661 | 3664 | multicellular organismal process |
| GO:0080134 | <0.0001 | 0.618 | 112 | 77 | 562 | regulation of response to stress |
| GO:0051234 | <0.0001 | 0.773 | 435 | 373 | 2181 | establishment of localization |
| GO:0070887 | <0.0001 | 0.730 | 262 | 211 | 1315 | cellular response to chemical stimulus |
| GO:0008202 | <0.0001 | 0.431 | 43 | 21 | 213 | steroid metabolic process |
| GO:0072358 | <0.0001 | 0.629 | 119 | 83 | 596 | cardiovascular system development |
| GO:0072359 | <0.0001 | 0.629 | 119 | 83 | 596 | circulatory system development |
| GO:0010648 | <0.0001 | 0.592 | 94 | 62 | 471 | negative regulation of cell communication |
| GO:0001817 | <0.0001 | 0.493 | 55 | 31 | 278 | regulation of cytokine production |
| GO:0051170 | <0.0001 | 0.382 | 34 | 15 | 170 | nuclear import |
| GO:0007599 | <0.0001 | 0.570 | 82 | 52 | 409 | hemostasis |
| GO:0023057 | <0.0001 | 0.595 | 94 | 62 | 469 | negative regulation of signaling |
| GO:0032989 | <0.0001 | 0.659 | 139 | 101 | 696 | cellular component morphogenesis |
| GO:0071345 | <0.0001 | 0.533 | 65 | 39 | 326 | cellular response to cytokine stimulus |
| GO:0006606 | <0.0001 | 0.387 | 34 | 15 | 168 | protein import into nucleus |
| GO:0044744 | <0.0001 | 0.387 | 34 | 15 | 168 | protein targeting to nucleus |
| GO:0050778 | <0.0001 | 0.496 | 53 | 30 | 268 | positive regulation of immune response |
| GO:0065007 | <0.0001 | 0.798 | 1049 | 985 | 5255 | biological regulation |
| GO:0023056 | <0.0001 | 0.650 | 127 | 91 | 635 | positive regulation of signaling |
| GO:0006935 | <0.0001 | 0.607 | 95 | 64 | 476 | chemotaxis |
| GO:0042330 | <0.0001 | 0.607 | 95 | 64 | 476 | taxis |
| GO:0050727 | <0.0001 | 0.370 | 30 | 13 | 152 | regulation of inflammatory response |
| GO:0051240 | <0.0001 | 0.580 | 80 | 52 | 403 | positive regulation of multicellular organismal process |
| GO:0050789 | <0.0001 | 0.804 | 990 | 925 | 4958 | regulation of biological process |
| GO:0071310 | <0.0001 | 0.718 | 207 | 163 | 1038 | cellular response to organic substance |
| GO:0034097 | <0.0001 | 0.582 | 80 | 52 | 402 | response to cytokine stimulus |
| GO:0051707 | <0.0001 | 0.587 | 83 | 54 | 414 | response to other organism |
| GO:0007167 | <0.0001 | 0.671 | 138 | 102 | 693 | enzyme linked receptor protein signaling pathway |
| GO:0001944 | <0.0001 | 0.592 | 82 | 54 | 411 | vasculature development |
| GO:0009967 | <0.0001 | 0.653 | 120 | 86 | 599 | positive regulation of signal transduction |
| GO:0030168 | <0.0001 | 0.439 | 38 | 19 | 190 | platelet activation |
| GO:1901342 | <0.0001 | 0.354 | 27 | 11 | 134 | regulation of vasculature development |
| GO:0001525 | <0.0001 | 0.523 | 56 | 33 | 281 | angiogenesis |
| GO:0002274 | <0.0001 | 0.264 | 19 | 6 | 96 | myeloid leukocyte activation |
| GO:0051249 | <0.0001 | 0.476 | 45 | 24 | 223 | regulation of lymphocyte activation |
| GO:0009607 | <0.0001 | 0.602 | 85 | 57 | 428 | response to biotic stimulus |
| GO:0019221 | <0.0001 | 0.515 | 53 | 31 | 268 | cytokine-mediated signaling pathway |
| GO:0002443 | <0.0001 | 0.403 | 32 | 15 | 162 | leukocyte mediated immunity |
| GO:0003008 | <0.0001 | 0.742 | 239 | 194 | 1199 | system process |
| GO:0045765 | <0.0001 | 0.344 | 25 | 10 | 125 | regulation of angiogenesis |
| GO:0051050 | <0.0001 | 0.583 | 74 | 48 | 371 | positive regulation of transport |
| GO:0000902 | <0.0001 | 0.673 | 131 | 97 | 658 | cell morphogenesis |
| GO:0050793 | <0.0001 | 0.729 | 207 | 165 | 1038 | regulation of developmental process |
| GO:0042110 | <0.0001 | 0.525 | 54 | 32 | 272 | T cell activation |
| GO:0032103 | <0.0001 | 0.360 | 26 | 11 | 132 | positive regulation of response to external stimulus |
| GO:0019722 | <0.0001 | 0.181 | 14 | 3 | 69 | calcium-mediated signaling |
| GO:0045766 | <0.0001 | 0.181 | 14 | 3 | 69 | positive regulation of angiogenesis |
| GO:0000165 | <0.0001 | 0.601 | 81 | 54 | 406 | MAPK cascade |
| GO:0060341 | <0.0001 | 0.638 | 101 | 71 | 506 | regulation of cellular localization |
| GO:0018212 | <0.0001 | 0.425 | 33 | 16 | 165 | peptidyl-tyrosine modification |
| GO:0043122 | <0.0001 | 0.366 | 26 | 11 | 130 | regulation of I-kappaB kinase/NF-kappaB cascade |
| GO:0048870 | <0.0001 | 0.688 | 140 | 105 | 699 | cell motility |
| GO:0051674 | <0.0001 | 0.688 | 140 | 105 | 699 | localization of cell |
| GO:0009653 | <0.0001 | 0.766 | 286 | 239 | 1434 | anatomical structure morphogenesis |
| GO:0046649 | <0.0001 | 0.588 | 72 | 47 | 361 | lymphocyte activation |
| GO:0017038 | <0.0001 | 0.455 | 37 | 19 | 184 | protein import |
| GO:0034504 | <0.0001 | 0.471 | 39 | 21 | 197 | protein localization to nucleus |
| GO:0009968 | <0.0001 | 0.626 | 88 | 61 | 443 | negative regulation of signal transduction |
| GO:0018108 | <0.0001 | 0.431 | 33 | 16 | 163 | peptidyl-tyrosine phosphorylation |
| GO:0016477 | <0.0001 | 0.687 | 132 | 99 | 661 | cell migration |
| GO:0022603 | <0.0001 | 0.631 | 89 | 62 | 447 | regulation of anatomical structure morphogenesis |
| GO:0046486 | <0.0001 | 0.486 | 40 | 22 | 201 | glycerolipid metabolic process |
| GO:0072330 | <0.0001 | 0.364 | 24 | 10 | 119 | monocarboxylic acid biosynthetic process |
| GO:0060191 | <0.0001 | 0.287 | 18 | 6 | 89 | regulation of lipase activity |
| GO:0048646 | <0.0001 | 0.754 | 226 | 185 | 1132 | anatomical structure formation involved in morphogenesis |
| GO:0050867 | <0.0001 | 0.483 | 39 | 21 | 193 | positive regulation of cell activation |
| GO:0007249 | 0.001 | 0.409 | 28 | 13 | 139 | I-kappaB kinase/NF-kappaB cascade |
| GO:0002449 | 0.001 | 0.383 | 25 | 11 | 125 | lymphocyte mediated immunity |
| GO:1901617 | 0.001 | 0.383 | 25 | 11 | 125 | organic hydroxy compound biosynthetic process |
| GO:0030029 | 0.001 | 0.598 | 68 | 45 | 341 | actin filament-based process |
| GO:0060326 | 0.001 | 0.400 | 26 | 12 | 131 | cell chemotaxis |
| GO:0030282 | 0.001 | 0.199 | 13 | 3 | 63 | bone mineralization |
| GO:2000026 | 0.001 | 0.726 | 162 | 128 | 814 | regulation of multicellular organismal development |
| GO:0001568 | 0.001 | 0.626 | 78 | 54 | 393 | blood vessel development |
| GO:0042108 | 0.001 | 0.093 | 9 | 1 | 44 | positive regulation of cytokine biosynthetic process |
| GO:0006066 | 0.001 | 0.517 | 43 | 25 | 216 | alcohol metabolic process |
| GO:0008610 | 0.001 | 0.614 | 72 | 49 | 363 | lipid biosynthetic process |
| GO:0010740 | 0.001 | 0.614 | 72 | 49 | 363 | positive regulation of intracellular protein kinase cascade |
| GO:0009914 | 0.001 | 0.469 | 34 | 18 | 170 | hormone transport |
| GO:0002250 | 0.001 | 0.440 | 30 | 15 | 150 | adaptive immune response |
| GO:0046165 | 0.001 | 0.342 | 20 | 8 | 101 | alcohol biosynthetic process |
| GO:0002696 | 0.001 | 0.485 | 37 | 20 | 183 | positive regulation of leukocyte activation |
| GO:0010033 | 0.001 | 0.786 | 294 | 250 | 1472 | response to organic substance |
| GO:0030595 | 0.001 | 0.361 | 22 | 9 | 108 | leukocyte chemotaxis |
| GO:0009617 | 0.001 | 0.543 | 48 | 29 | 240 | response to bacterium |
| GO:0016042 | 0.001 | 0.461 | 33 | 17 | 163 | lipid catabolic process |
| GO:0044699 | 0.001 | 0.800 | 1264 | 1219 | 6334 | single-organism process |
| GO:0050878 | 0.001 | 0.663 | 97 | 70 | 484 | regulation of body fluid levels |
| GO:0003013 | 0.001 | 0.583 | 59 | 38 | 295 | circulatory system process |
| GO:0002697 | 0.001 | 0.472 | 34 | 18 | 169 | regulation of immune effector process |
| GO:0050794 | 0.001 | 0.834 | 937 | 882 | 4695 | regulation of cellular process |
| GO:0008015 | 0.001 | 0.585 | 59 | 38 | 294 | blood circulation |
| GO:0048514 | 0.001 | 0.612 | 68 | 46 | 342 | blood vessel morphogenesis |
| GO:0045834 | 0.001 | 0.276 | 15 | 5 | 77 | positive regulation of lipid metabolic process |
| GO:0030335 | 0.001 | 0.506 | 39 | 22 | 194 | positive regulation of cell migration |
| GO:0008203 | 0.001 | 0.327 | 18 | 7 | 92 | cholesterol metabolic process |
| GO:0071495 | 0.001 | 0.693 | 114 | 86 | 572 | cellular response to endogenous stimulus |
| GO:0051241 | 0.001 | 0.578 | 55 | 35 | 274 | negative regulation of multicellular organismal process |
| GO:0006887 | 0.001 | 0.517 | 40 | 23 | 199 | exocytosis |
| GO:0030098 | 0.001 | 0.504 | 37 | 21 | 186 | lymphocyte differentiation |
| GO:0043408 | 0.001 | 0.624 | 70 | 48 | 351 | regulation of MAPK cascade |
| GO:0001819 | 0.001 | 0.463 | 31 | 16 | 153 | positive regulation of cytokine production |
| GO:0030036 | 0.001 | 0.596 | 59 | 39 | 297 | actin cytoskeleton organization |
| GO:0007267 | 0.001 | 0.738 | 162 | 129 | 810 | cell-cell signaling |
| GO:0051251 | 0.001 | 0.482 | 33 | 18 | 166 | positive regulation of lymphocyte activation |
| GO:0003001 | 0.001 | 0.574 | 52 | 33 | 260 | generation of a signal involved in cell-cell signaling |
| GO:0023061 | 0.001 | 0.574 | 52 | 33 | 260 | signal release |
| GO:0060627 | 0.001 | 0.474 | 32 | 17 | 159 | regulation of vesicle-mediated transport |
| GO:0051046 | 0.001 | 0.618 | 66 | 45 | 332 | regulation of secretion |
| GO:0051649 | 0.001 | 0.783 | 251 | 212 | 1258 | establishment of localization in cell |
| GO:0002253 | 0.001 | 0.541 | 43 | 26 | 216 | activation of immune response |
| GO:0046879 | 0.001 | 0.485 | 33 | 18 | 165 | hormone secretion |
| GO:0007169 | 0.001 | 0.681 | 100 | 74 | 500 | transmembrane receptor protein tyrosine kinase signaling pathway |
| GO:0032502 | 0.001 | 0.836 | 580 | 528 | 2904 | developmental process |
| GO:0002521 | 0.001 | 0.581 | 53 | 34 | 265 | leukocyte differentiation |
| GO:0006950 | 0.001 | 0.820 | 415 | 368 | 2079 | response to stress |
| GO:0051641 | 0.001 | 0.793 | 278 | 238 | 1395 | cellular localization |
| GO:0071702 | 0.001 | 0.786 | 253 | 214 | 1267 | organic substance transport |
| GO:0048869 | 0.001 | 0.814 | 371 | 326 | 1859 | cellular developmental process |
| GO:0002526 | 0.001 | 0.339 | 18 | 7 | 89 | acute inflammatory response |
| GO:0050920 | 0.001 | 0.339 | 18 | 7 | 89 | regulation of chemotaxis |
| GO:0050730 | 0.001 | 0.425 | 25 | 12 | 124 | regulation of peptidyl-tyrosine phosphorylation |
| GO:0030100 | 0.001 | 0.361 | 19 | 8 | 96 | regulation of endocytosis |
| GO:0040013 | 0.002 | 0.412 | 23 | 11 | 117 | negative regulation of locomotion |
| GO:0002237 | 0.002 | 0.508 | 35 | 20 | 176 | response to molecule of bacterial origin |
| GO:0044057 | 0.002 | 0.642 | 73 | 51 | 364 | regulation of system process |
| GO:0070848 | 0.002 | 0.642 | 73 | 51 | 364 | response to growth factor stimulus |
| GO:0031349 | 0.002 | 0.517 | 36 | 21 | 182 | positive regulation of defense response |
| GO:0016125 | 0.002 | 0.365 | 19 | 8 | 95 | sterol metabolic process |
| GO:0042035 | 0.002 | 0.261 | 13 | 4 | 65 | regulation of cytokine biosynthetic process |
| GO:0051051 | 0.002 | 0.556 | 44 | 27 | 219 | negative regulation of transport |
| GO:0006968 | 0.002 | 0.173 | 10 | 2 | 48 | cellular defense response |
| GO:0040017 | 0.002 | 0.547 | 41 | 25 | 206 | positive regulation of locomotion |
| GO:0051047 | 0.002 | 0.505 | 34 | 19 | 168 | positive regulation of secretion |
| GO:0070372 | 0.002 | 0.348 | 17 | 7 | 87 | regulation of ERK1 and ERK2 cascade |
| GO:0006928 | 0.002 | 0.760 | 177 | 145 | 888 | cellular component movement |
| GO:0050679 | 0.002 | 0.370 | 19 | 8 | 94 | positive regulation of epithelial cell proliferation |
| GO:0070371 | 0.002 | 0.389 | 20 | 9 | 101 | ERK1 and ERK2 cascade |
| GO:0002460 | 0.002 | 0.459 | 27 | 14 | 135 | adaptive immune response based on somatic recombination of immune receptors built from immunoglobulin superfamily domains |
| GO:1900274 | 0.002 | 0.225 | 11 | 3 | 56 | regulation of phospholipase C activity |
| GO:0042127 | 0.002 | 0.761 | 177 | 145 | 887 | regulation of cell proliferation |
| GO:0032496 | 0.002 | 0.509 | 33 | 19 | 167 | response to lipopolysaccharide |
| GO:0030097 | 0.002 | 0.664 | 79 | 57 | 395 | hemopoiesis |
| GO:0000904 | 0.002 | 0.704 | 105 | 80 | 526 | cell morphogenesis involved in differentiation |
| GO:2000147 | 0.002 | 0.546 | 40 | 24 | 198 | positive regulation of cell motility |
| GO:0051271 | 0.002 | 0.410 | 21 | 10 | 107 | negative regulation of cellular component movement |
| GO:0051272 | 0.002 | 0.553 | 41 | 25 | 204 | positive regulation of cellular component movement |
| GO:0010863 | 0.002 | 0.230 | 11 | 3 | 55 | positive regulation of phospholipase C activity |
| GO:0070201 | 0.002 | 0.587 | 48 | 31 | 240 | regulation of establishment of protein localization |
| GO:0050795 | 0.003 | 0.428 | 23 | 11 | 113 | regulation of behavior |
| GO:0030155 | 0.003 | 0.568 | 43 | 27 | 215 | regulation of cell adhesion |
| GO:0071363 | 0.003 | 0.653 | 70 | 50 | 352 | cellular response to growth factor stimulus |
| GO:0019915 | 0.003 | 0.111 | 7 | 1 | 37 | lipid storage |
| GO:0030073 | 0.003 | 0.457 | 25 | 13 | 126 | insulin secretion |
| GO:0010517 | 0.003 | 0.306 | 14 | 5 | 70 | regulation of phospholipase activity |
| GO:0030593 | 0.003 | 0.181 | 9 | 2 | 46 | neutrophil chemotaxis |
| GO:0030072 | 0.003 | 0.480 | 28 | 15 | 139 | peptide hormone secretion |
| GO:0030278 | 0.003 | 0.445 | 24 | 12 | 119 | regulation of ossification |
| GO:0001936 | 0.003 | 0.275 | 12 | 4 | 62 | regulation of endothelial cell proliferation |
| GO:0050863 | 0.003 | 0.533 | 35 | 21 | 177 | regulation of T cell activation |
| GO:0070098 | 0.003 | <0.0001 | 5 | 0 | 26 | chemokine-mediated signaling pathway |
| GO:0060193 | 0.003 | 0.311 | 14 | 5 | 69 | positive regulation of lipase activity |
| GO:0001938 | 0.003 | 0.185 | 9 | 2 | 45 | positive regulation of endothelial cell proliferation |
| GO:0051223 | 0.003 | 0.580 | 44 | 28 | 219 | regulation of protein transport |
| GO:0046470 | 0.003 | 0.114 | 7 | 1 | 36 | phosphatidylcholine metabolic process |
| GO:0006638 | 0.003 | 0.341 | 15 | 6 | 76 | neutral lipid metabolic process |
| GO:0008360 | 0.003 | 0.341 | 15 | 6 | 76 | regulation of cell shape |
| GO:0042107 | 0.003 | 0.341 | 15 | 6 | 76 | cytokine metabolic process |
| GO:0060333 | 0.003 | 0.280 | 12 | 4 | 61 | interferon-gamma-mediated signaling pathway |
| GO:0046887 | 0.003 | 0.239 | 11 | 3 | 53 | positive regulation of hormone secretion |
| GO:0032956 | 0.003 | 0.488 | 27 | 15 | 137 | regulation of actin cytoskeleton organization |
| GO:0050870 | 0.003 | 0.488 | 27 | 15 | 137 | positive regulation of T cell activation |
| GO:0002790 | 0.004 | 0.500 | 29 | 16 | 143 | peptide secretion |
| GO:0050673 | 0.004 | 0.556 | 37 | 23 | 187 | epithelial cell proliferation |
| GO:0010883 | 0.004 | <0.0001 | 5 | 0 | 25 | regulation of lipid storage |
| GO:1900180 | 0.004 | 0.427 | 21 | 10 | 103 | regulation of protein localization to nucleus |
| GO:0006639 | 0.004 | 0.346 | 15 | 6 | 75 | acylglycerol metabolic process |
| GO:0045088 | 0.004 | 0.544 | 35 | 21 | 174 | regulation of innate immune response |
| GO:0046824 | 0.004 | 0.285 | 12 | 4 | 60 | positive regulation of nucleocytoplasmic transport |
| GO:0090066 | 0.004 | 0.592 | 45 | 29 | 223 | regulation of anatomical structure size |
| GO:0042886 | 0.004 | 0.521 | 31 | 18 | 155 | amide transport |
| GO:0034341 | 0.004 | 0.393 | 18 | 8 | 89 | response to interferon-gamma |
| GO:0043542 | 0.004 | 0.393 | 18 | 8 | 89 | endothelial cell migration |
| GO:0006953 | 0.004 | 0.117 | 7 | 1 | 35 | acute-phase response |
| GO:0007159 | 0.004 | 0.117 | 7 | 1 | 35 | leukocyte cell-cell adhesion |
| GO:0045807 | 0.004 | 0.244 | 10 | 3 | 52 | positive regulation of endocytosis |
| GO:0008285 | 0.004 | 0.693 | 84 | 63 | 421 | negative regulation of cell proliferation |
| GO:0030003 | 0.004 | 0.631 | 55 | 38 | 276 | cellular cation homeostasis |
| GO:1901615 | 0.004 | 0.631 | 55 | 38 | 276 | organic hydroxy compound metabolic process |
| GO:0015833 | 0.004 | 0.515 | 30 | 17 | 148 | peptide transport |
| GO:2000146 | 0.004 | 0.432 | 20 | 10 | 102 | negative regulation of cell motility |
| GO:0007275 | 0.004 | 0.852 | 527 | 483 | 2641 | multicellular organismal development |
| GO:0042089 | 0.004 | 0.351 | 15 | 6 | 74 | cytokine biosynthetic process |
| GO:0030154 | 0.005 | 0.833 | 356 | 317 | 1783 | cell differentiation |
| GO:0019216 | 0.005 | 0.535 | 32 | 19 | 160 | regulation of lipid metabolic process |
| GO:0032970 | 0.005 | 0.535 | 32 | 19 | 160 | regulation of actin filament-based process |
| GO:0045619 | 0.005 | 0.376 | 16 | 7 | 81 | regulation of lymphocyte differentiation |
| GO:0090092 | 0.005 | 0.463 | 23 | 12 | 115 | regulation of transmembrane receptor protein serine/threonine kinase signaling pathway |
| GO:0032880 | 0.005 | 0.634 | 55 | 38 | 275 | regulation of protein localization |
| GO:0002706 | 0.005 | 0.290 | 12 | 4 | 59 | regulation of lymphocyte mediated immunity |
| GO:0003012 | 0.005 | 0.608 | 47 | 31 | 233 | muscle system process |
| GO:0043551 | 0.005 | <0.0001 | 5 | 0 | 24 | regulation of phosphatidylinositol 3-kinase activity |
| GO:0045428 | 0.005 | 0.121 | 7 | 1 | 34 | regulation of nitric oxide biosynthetic process |
| GO:0071347 | 0.005 | 0.121 | 7 | 1 | 34 | cellular response to interleukin-1 |
| GO:0072001 | 0.005 | 0.566 | 37 | 23 | 184 | renal system development |
| GO:0042306 | 0.005 | 0.437 | 20 | 10 | 101 | regulation of protein import into nucleus |
| GO:0050731 | 0.005 | 0.437 | 20 | 10 | 101 | positive regulation of peptidyl-tyrosine phosphorylation |
| GO:0051128 | 0.005 | 0.785 | 178 | 149 | 891 | regulation of cellular component organization |
| GO:0071216 | 0.005 | 0.403 | 17 | 8 | 87 | cellular response to biotic stimulus |
| GO:0030336 | 0.006 | 0.442 | 20 | 10 | 100 | negative regulation of cell migration |
| GO:0042991 | 0.006 | 0.255 | 10 | 3 | 50 | transcription factor import into nucleus |
| GO:0002822 | 0.006 | 0.332 | 13 | 5 | 65 | regulation of adaptive immune response based on somatic recombination of immune receptors built from immunoglobulin superfamily domains |
| GO:0090218 | 0.006 | <0.0001 | 5 | 0 | 23 | positive regulation of lipid kinase activity |
| GO:0006641 | 0.006 | 0.362 | 14 | 6 | 72 | triglyceride metabolic process |
| GO:0002768 | 0.006 | 0.460 | 21 | 11 | 106 | immune response-regulating cell surface receptor signaling pathway |
| GO:0048858 | 0.006 | 0.723 | 95 | 74 | 477 | cell projection morphogenesis |
| GO:0045595 | 0.006 | 0.771 | 147 | 121 | 736 | regulation of cell differentiation |
| GO:0032990 | 0.006 | 0.726 | 96 | 75 | 482 | cell part morphogenesis |
| GO:0032787 | 0.006 | 0.642 | 53 | 37 | 265 | monocarboxylic acid metabolic process |
| GO:0007610 | 0.006 | 0.704 | 80 | 61 | 403 | behavior |
| GO:0046883 | 0.006 | 0.532 | 29 | 17 | 144 | regulation of hormone secretion |
| GO:0051336 | 0.007 | 0.742 | 109 | 87 | 548 | regulation of hydrolase activity |
| GO:0007202 | 0.007 | 0.260 | 10 | 3 | 49 | activation of phospholipase C activity |
| GO:0042990 | 0.007 | 0.260 | 10 | 3 | 49 | regulation of transcription factor import into nucleus |
| GO:0043534 | 0.007 | 0.260 | 10 | 3 | 49 | blood vessel endothelial cell migration |
| GO:0030099 | 0.007 | 0.590 | 39 | 25 | 193 | myeloid cell differentiation |
| GO:0010518 | 0.007 | 0.338 | 13 | 5 | 64 | positive regulation of phospholipase activity |
| GO:0070374 | 0.007 | 0.338 | 13 | 5 | 64 | positive regulation of ERK1 and ERK2 cascade |
| GO:0090101 | 0.007 | 0.338 | 13 | 5 | 64 | negative regulation of transmembrane receptor protein serine/threonine kinase signaling pathway |
| GO:0006644 | 0.007 | 0.606 | 42 | 28 | 211 | phospholipid metabolic process |
| GO:0001935 | 0.007 | 0.368 | 14 | 6 | 71 | endothelial cell proliferation |
| GO:0050921 | 0.007 | 0.368 | 14 | 6 | 71 | positive regulation of chemotaxis |
| GO:0003015 | 0.007 | 0.492 | 24 | 13 | 118 | heart process |
| GO:0060047 | 0.007 | 0.492 | 24 | 13 | 118 | heart contraction |
| GO:0044087 | 0.007 | 0.645 | 53 | 37 | 264 | regulation of cellular component biogenesis |
| GO:0019229 | 0.007 | 0.129 | 6 | 1 | 32 | regulation of vasoconstriction |
| GO:0002429 | 0.007 | 0.452 | 20 | 10 | 98 | immune response-activating cell surface receptor signaling pathway |
| GO:0019226 | 0.007 | 0.746 | 110 | 88 | 552 | transmission of nerve impulse |
| GO:0002764 | 0.007 | 0.588 | 37 | 24 | 186 | immune response-regulating signaling pathway |
| GO:0009615 | 0.007 | 0.599 | 40 | 26 | 198 | response to virus |
| GO:0042157 | 0.007 | 0.307 | 11 | 4 | 56 | lipoprotein metabolic process |
| GO:0001954 | 0.007 | <0.0001 | 4 | 0 | 22 | positive regulation of cell-matrix adhesion |
| GO:0010543 | 0.007 | <0.0001 | 4 | 0 | 22 | regulation of platelet activation |
| GO:0032845 | 0.007 | <0.0001 | 4 | 0 | 22 | negative regulation of homeostatic process |
| GO:0043552 | 0.007 | <0.0001 | 4 | 0 | 22 | positive regulation of phosphatidylinositol 3-kinase activity |
| GO:0045058 | 0.007 | <0.0001 | 4 | 0 | 22 | T cell selection |
| GO:0001822 | 0.007 | 0.546 | 30 | 18 | 149 | kidney development |
| GO:0002699 | 0.008 | 0.419 | 17 | 8 | 84 | positive regulation of immune effector process |
| GO:0042439 | 0.008 | 0.266 | 10 | 3 | 48 | ethanolamine-containing compound metabolic process |
| GO:0046890 | 0.008 | 0.398 | 15 | 7 | 77 | regulation of lipid biosynthetic process |
| GO:0010817 | 0.008 | 0.657 | 55 | 39 | 274 | regulation of hormone levels |
| GO:0002819 | 0.008 | 0.373 | 14 | 6 | 70 | regulation of adaptive immune response |
| GO:0051480 | 0.008 | 0.540 | 28 | 17 | 142 | cytosolic calcium ion homeostasis |
| GO:0040008 | 0.008 | 0.703 | 75 | 57 | 377 | regulation of growth |
| GO:0009306 | 0.008 | 0.511 | 25 | 14 | 123 | protein secretion |
| GO:0050678 | 0.009 | 0.567 | 32 | 20 | 160 | regulation of epithelial cell proliferation |
| GO:0035637 | 0.009 | 0.756 | 114 | 92 | 571 | multicellular organismal signaling |
| GO:0070555 | 0.009 | 0.313 | 11 | 4 | 55 | response to interleukin-1 |
| GO:0048534 | 0.009 | 0.720 | 84 | 65 | 421 | hematopoietic or lymphoid organ development |
| GO:0030217 | 0.009 | 0.534 | 27 | 16 | 135 | T cell differentiation |
| GO:0019935 | 0.009 | 0.133 | 6 | 1 | 31 | cyclic-nucleotide-mediated signaling |
| GO:0032024 | 0.009 | 0.133 | 6 | 1 | 31 | positive regulation of insulin secretion |
| GO:0042098 | 0.009 | 0.476 | 21 | 11 | 103 | T cell proliferation |
| GO:0043269 | 0.009 | 0.622 | 43 | 29 | 214 | regulation of ion transport |
| GO:0002703 | 0.009 | 0.404 | 15 | 7 | 76 | regulation of leukocyte mediated immunity |
| GO:0006909 | 0.009 | 0.379 | 14 | 6 | 69 | phagocytosis |
| GO:0034405 | 0.009 | <0.0001 | 4 | 0 | 21 | response to fluid shear stress |
| GO:0043124 | 0.009 | <0.0001 | 4 | 0 | 21 | negative regulation of I-kappaB kinase/NF-kappaB cascade |
| GO:0051058 | 0.009 | <0.0001 | 4 | 0 | 21 | negative regulation of small GTPase mediated signal transduction |
| GO:0002793 | 0.009 | 0.216 | 8 | 2 | 39 | positive regulation of peptide secretion |
| GO:0042308 | 0.009 | 0.216 | 8 | 2 | 39 | negative regulation of protein import into nucleus |
| GO:0043149 | 0.009 | 0.216 | 8 | 2 | 39 | stress fiber assembly |
| GO:0002757 | 0.010 | 0.593 | 35 | 23 | 177 | immune response-activating signal transduction |
| GO:1901699 | 0.010 | 0.682 | 61 | 45 | 306 | cellular response to nitrogen compound |
| GO:0006650 | 0.010 | 0.559 | 29 | 18 | 146 | glycerophospholipid metabolic process |
| GO:0045926 | 0.010 | 0.559 | 29 | 18 | 146 | negative regulation of growth |
| GO:0001655 | 0.010 | 0.633 | 44 | 30 | 218 | urogenital system development |
| GO:0022604 | 0.010 | 0.633 | 44 | 30 | 218 | regulation of cell morphogenesis |
| GO:0002685 | 0.010 | 0.410 | 15 | 7 | 75 | regulation of leukocyte migration |
| GO:0048519 | 0.010 | 0.861 | 436 | 399 | 2184 | negative regulation of biological process |
| GO:0050729 | 0.010 | 0.356 | 12 | 5 | 61 | positive regulation of inflammatory response |
| GO:0006936 | 0.010 | 0.624 | 41 | 28 | 206 | muscle contraction |
| GO:0048011 | 0.011 | 0.609 | 38 | 25 | 188 | nerve growth factor receptor signaling pathway |
| GO:0045184 | 0.011 | 0.799 | 166 | 141 | 833 | establishment of protein localization |
| GO:0048731 | 0.011 | 0.864 | 471 | 433 | 2358 | system development |
| GO:0050796 | 0.011 | 0.512 | 23 | 13 | 114 | regulation of insulin secretion |
| GO:0048518 | 0.011 | 0.866 | 485 | 447 | 2429 | positive regulation of biological process |
| GO:0030500 | 0.011 | 0.278 | 9 | 3 | 46 | regulation of bone mineralization |
| GO:0046427 | 0.011 | 0.278 | 9 | 3 | 46 | positive regulation of JAK-STAT cascade |
| GO:0008016 | 0.011 | 0.486 | 20 | 11 | 101 | regulation of heart contraction |
| GO:0048856 | 0.011 | 0.870 | 529 | 490 | 2648 | anatomical structure development |
| GO:0046635 | 0.011 | 0.222 | 8 | 2 | 38 | positive regulation of alpha-beta T cell activation |
| GO:0090277 | 0.011 | 0.222 | 8 | 2 | 38 | positive regulation of peptide hormone secretion |
| GO:0048878 | 0.011 | 0.776 | 127 | 105 | 637 | chemical homeostasis |
| GO:0071219 | 0.011 | 0.437 | 16 | 8 | 81 | cellular response to molecule of bacterial origin |
| GO:0016311 | 0.012 | 0.587 | 33 | 21 | 163 | dephosphorylation |
| GO:0055080 | 0.012 | 0.694 | 63 | 47 | 315 | cation homeostasis |
| GO:0021988 | 0.012 | <0.0001 | 4 | 0 | 20 | olfactory lobe development |
| GO:0043666 | 0.012 | <0.0001 | 4 | 0 | 20 | regulation of phosphoprotein phosphatase activity |
| GO:0046580 | 0.012 | <0.0001 | 4 | 0 | 20 | negative regulation of Ras protein signal transduction |
| GO:0051865 | 0.012 | <0.0001 | 4 | 0 | 20 | protein autoubiquitination |
| GO:0071804 | 0.012 | <0.0001 | 4 | 0 | 20 | cellular potassium ion transport |
| GO:0071805 | 0.012 | <0.0001 | 4 | 0 | 20 | potassium ion transmembrane transport |
| GO:0044242 | 0.012 | 0.503 | 21 | 12 | 107 | cellular lipid catabolic process |
| GO:0051048 | 0.012 | 0.474 | 19 | 10 | 94 | negative regulation of secretion |
| GO:0001906 | 0.012 | 0.326 | 11 | 4 | 53 | cell killing |
| GO:0019218 | 0.012 | 0.326 | 11 | 4 | 53 | regulation of steroid metabolic process |
| GO:0033559 | 0.012 | 0.326 | 11 | 4 | 53 | unsaturated fatty acid metabolic process |
| GO:0046631 | 0.012 | 0.416 | 15 | 7 | 74 | alpha-beta T cell activation |
| GO:0044283 | 0.012 | 0.690 | 60 | 45 | 303 | small molecule biosynthetic process |
| GO:0010594 | 0.012 | 0.362 | 12 | 5 | 60 | regulation of endothelial cell migration |
| GO:0006875 | 0.012 | 0.664 | 50 | 36 | 251 | cellular metal ion homeostasis |
| GO:0044767 | 0.012 | 0.868 | 474 | 437 | 2374 | single-organism developmental process |
| GO:0040007 | 0.012 | 0.766 | 112 | 91 | 559 | growth |
| GO:0048812 | 0.012 | 0.738 | 86 | 68 | 432 | neuron projection morphogenesis |
| GO:0043550 | 0.013 | 0.143 | 6 | 1 | 29 | regulation of lipid kinase activity |
| GO:0002791 | 0.013 | 0.542 | 25 | 15 | 125 | regulation of peptide secretion |
| GO:0046822 | 0.013 | 0.542 | 25 | 15 | 125 | regulation of nucleocytoplasmic transport |
| GO:0090087 | 0.013 | 0.542 | 25 | 15 | 125 | regulation of peptide transport |
| GO:0060491 | 0.013 | 0.285 | 9 | 3 | 45 | regulation of cell projection assembly |
| GO:0002573 | 0.013 | 0.508 | 21 | 12 | 106 | myeloid leukocyte differentiation |
| GO:0034284 | 0.013 | 0.480 | 19 | 10 | 93 | response to monosaccharide stimulus |
| GO:0045446 | 0.013 | 0.228 | 7 | 2 | 37 | endothelial cell differentiation |
| GO:0043271 | 0.014 | 0.332 | 10 | 4 | 52 | negative regulation of ion transport |
| GO:0010959 | 0.014 | 0.581 | 30 | 19 | 149 | regulation of metal ion transport |
| GO:0045580 | 0.014 | 0.399 | 13 | 6 | 66 | regulation of T cell differentiation |
| GO:0045785 | 0.014 | 0.498 | 20 | 11 | 99 | positive regulation of cell adhesion |
| GO:0007259 | 0.014 | 0.466 | 17 | 9 | 86 | JAK-STAT cascade |
| GO:0045444 | 0.014 | 0.466 | 17 | 9 | 86 | fat cell differentiation |
| GO:0050866 | 0.014 | 0.466 | 17 | 9 | 86 | negative regulation of cell activation |
| GO:0006699 | 0.014 | <0.0001 | 4 | 0 | 19 | bile acid biosynthetic process |
| GO:0021772 | 0.014 | <0.0001 | 4 | 0 | 19 | olfactory bulb development |
| GO:0046173 | 0.014 | <0.0001 | 4 | 0 | 19 | polyol biosynthetic process |
| GO:0048260 | 0.014 | <0.0001 | 4 | 0 | 19 | positive regulation of receptor-mediated endocytosis |
| GO:0008284 | 0.015 | 0.761 | 100 | 81 | 501 | positive regulation of cell proliferation |
| GO:0033036 | 0.015 | 0.836 | 237 | 209 | 1186 | macromolecule localization |
| GO:0090287 | 0.015 | 0.528 | 22 | 13 | 111 | regulation of cellular response to growth factor stimulus |
| GO:0032835 | 0.015 | 0.292 | 9 | 3 | 44 | glomerulus development |
| GO:0042310 | 0.015 | 0.292 | 9 | 3 | 44 | vasoconstriction |
| GO:0010742 | 0.016 | 0.148 | 6 | 1 | 28 | macrophage derived foam cell differentiation |
| GO:0030101 | 0.016 | 0.148 | 6 | 1 | 28 | natural killer cell activation |
| GO:0032652 | 0.016 | 0.148 | 6 | 1 | 28 | regulation of interleukin-1 production |
| GO:0090077 | 0.016 | 0.148 | 6 | 1 | 28 | foam cell differentiation |
| GO:0009266 | 0.016 | 0.472 | 17 | 9 | 85 | response to temperature stimulus |
| GO:0090276 | 0.016 | 0.553 | 25 | 15 | 123 | regulation of peptide hormone secretion |
| GO:0002700 | 0.016 | 0.340 | 10 | 4 | 51 | regulation of production of molecular mediator of immune response |
| GO:0070167 | 0.016 | 0.340 | 10 | 4 | 51 | regulation of biomineral tissue development |
| GO:0048523 | 0.016 | 0.867 | 400 | 367 | 2006 | negative regulation of cellular process |
| GO:0051238 | 0.016 | 0.235 | 7 | 2 | 36 | sequestering of metal ion |
| GO:0015837 | 0.016 | 0.376 | 12 | 5 | 58 | amine transport |
| GO:0045017 | 0.017 | 0.582 | 28 | 18 | 141 | glycerolipid biosynthetic process |
| GO:0070661 | 0.017 | 0.598 | 31 | 20 | 153 | leukocyte proliferation |
| GO:0071222 | 0.017 | 0.455 | 16 | 8 | 78 | cellular response to lipopolysaccharide |
| GO:0050851 | 0.017 | 0.492 | 18 | 10 | 91 | antigen receptor-mediated signaling pathway |
| GO:0006898 | 0.017 | 0.533 | 22 | 13 | 110 | receptor-mediated endocytosis |
| GO:0051094 | 0.017 | 0.773 | 106 | 87 | 531 | positive regulation of developmental process |
| GO:0051056 | 0.017 | 0.671 | 47 | 34 | 235 | regulation of small GTPase mediated signal transduction |
| GO:0009719 | 0.017 | 0.817 | 173 | 149 | 865 | response to endogenous stimulus |
| GO:0030324 | 0.018 | 0.558 | 24 | 15 | 122 | lung development |
| GO:0010811 | 0.018 | 0.299 | 9 | 3 | 43 | positive regulation of cell-substrate adhesion |
| GO:0030316 | 0.018 | 0.299 | 9 | 3 | 43 | osteoclast differentiation |
| GO:0042509 | 0.018 | 0.299 | 9 | 3 | 43 | regulation of tyrosine phosphorylation of STAT protein |
| GO:0050877 | 0.018 | 0.815 | 166 | 143 | 832 | neurological system process |
| GO:0002683 | 0.018 | 0.578 | 27 | 17 | 134 | negative regulation of immune system process |
| GO:0006112 | 0.018 | 0.578 | 27 | 17 | 134 | energy reserve metabolic process |
| GO:0007204 | 0.018 | 0.578 | 27 | 17 | 134 | elevation of cytosolic calcium ion concentration |
| GO:0030855 | 0.018 | 0.662 | 43 | 31 | 217 | epithelial cell differentiation |
| GO:0051220 | 0.018 | <0.0001 | 4 | 0 | 18 | cytoplasmic sequestering of protein |
| GO:0051955 | 0.018 | <0.0001 | 4 | 0 | 18 | regulation of amino acid transport |
| GO:0061437 | 0.018 | <0.0001 | 4 | 0 | 18 | renal system vasculature development |
| GO:0061440 | 0.018 | <0.0001 | 4 | 0 | 18 | kidney vasculature development |
| GO:0071312 | 0.018 | <0.0001 | 4 | 0 | 18 | cellular response to alkaloid |
| GO:0048659 | 0.018 | 0.413 | 13 | 6 | 64 | smooth muscle cell proliferation |
| GO:0032943 | 0.018 | 0.595 | 29 | 19 | 146 | mononuclear cell proliferation |
| GO:0034330 | 0.018 | 0.602 | 30 | 20 | 152 | cell junction organization |
| GO:0001934 | 0.018 | 0.756 | 88 | 71 | 442 | positive regulation of protein phosphorylation |
| GO:0007229 | 0.019 | 0.384 | 11 | 5 | 57 | integrin-mediated signaling pathway |
| GO:0032386 | 0.019 | 0.654 | 41 | 29 | 205 | regulation of intracellular transport |
| GO:1901652 | 0.019 | 0.706 | 58 | 44 | 291 | response to peptide |
| GO:0022406 | 0.019 | 0.154 | 5 | 1 | 27 | membrane docking |
| GO:0042992 | 0.019 | 0.154 | 5 | 1 | 27 | negative regulation of transcription factor import into nucleus |
| GO:0046503 | 0.019 | 0.154 | 5 | 1 | 27 | glycerolipid catabolic process |
| GO:0046638 | 0.019 | 0.154 | 5 | 1 | 27 | positive regulation of alpha-beta T cell differentiation |
| GO:0002224 | 0.019 | 0.498 | 18 | 10 | 90 | toll-like receptor signaling pathway |
| GO:0009746 | 0.019 | 0.498 | 18 | 10 | 90 | response to hexose stimulus |
| GO:0015850 | 0.019 | 0.498 | 18 | 10 | 90 | organic hydroxy compound transport |
| GO:0006873 | 0.019 | 0.761 | 91 | 74 | 458 | cellular ion homeostasis |
| GO:0048520 | 0.019 | 0.462 | 15 | 8 | 77 | positive regulation of behavior |
| GO:0071346 | 0.019 | 0.462 | 15 | 8 | 77 | cellular response to interferon-gamma |
| GO:0015031 | 0.019 | 0.814 | 160 | 138 | 804 | protein transport |
| GO:0070663 | 0.019 | 0.552 | 23 | 14 | 115 | regulation of leukocyte proliferation |
| GO:0007409 | 0.019 | 0.746 | 79 | 63 | 397 | axonogenesis |
| GO:0001503 | 0.020 | 0.665 | 43 | 31 | 216 | ossification |
| GO:0043123 | 0.020 | 0.515 | 19 | 11 | 96 | positive regulation of I-kappaB kinase/NF-kappaB cascade |
| GO:0043410 | 0.020 | 0.681 | 48 | 35 | 239 | positive regulation of MAPK cascade |
| GO:0071774 | 0.020 | 0.574 | 25 | 16 | 127 | response to fibroblast growth factor stimulus |
| GO:0010562 | 0.020 | 0.775 | 102 | 84 | 512 | positive regulation of phosphorus metabolic process |
| GO:0045937 | 0.020 | 0.775 | 102 | 84 | 512 | positive regulation of phosphate metabolic process |
| GO:0042698 | 0.020 | 0.443 | 14 | 7 | 70 | ovulation cycle |
| GO:0007268 | 0.020 | 0.768 | 96 | 78 | 479 | synaptic transmission |
| GO:0048608 | 0.020 | 0.654 | 40 | 28 | 198 | reproductive structure development |
| GO:0061458 | 0.020 | 0.654 | 40 | 28 | 198 | reproductive system development |
| GO:0048667 | 0.021 | 0.758 | 86 | 69 | 429 | cell morphogenesis involved in neuron differentiation |
| GO:0008104 | 0.021 | 0.835 | 205 | 180 | 1025 | protein localization |
| GO:0032868 | 0.021 | 0.650 | 38 | 27 | 192 | response to insulin stimulus |
| GO:0009743 | 0.021 | 0.545 | 22 | 13 | 108 | response to carbohydrate stimulus |
| GO:0045638 | 0.021 | 0.307 | 8 | 3 | 42 | negative regulation of myeloid cell differentiation |
| GO:0072511 | 0.021 | 0.646 | 37 | 26 | 186 | divalent inorganic cation transport |
| GO:0045597 | 0.021 | 0.748 | 78 | 62 | 390 | positive regulation of cell differentiation |
| GO:0003018 | 0.021 | 0.557 | 23 | 14 | 114 | vascular process in circulatory system |
| GO:0006694 | 0.021 | 0.557 | 23 | 14 | 114 | steroid biosynthetic process |
| GO:0045596 | 0.021 | 0.719 | 61 | 47 | 306 | negative regulation of cell differentiation |
| GO:0045621 | 0.021 | 0.391 | 11 | 5 | 56 | positive regulation of lymphocyte differentiation |
| GO:0006576 | 0.021 | 0.469 | 15 | 8 | 76 | cellular biogenic amine metabolic process |
| GO:0016197 | 0.021 | 0.469 | 15 | 8 | 76 | endosomal transport |
| GO:0030509 | 0.021 | 0.469 | 15 | 8 | 76 | BMP signaling pathway |
| GO:0050852 | 0.021 | 0.469 | 15 | 8 | 76 | T cell receptor signaling pathway |
| GO:0031623 | 0.021 | 0.355 | 10 | 4 | 49 | receptor internalization |
| GO:0044344 | 0.022 | 0.579 | 25 | 16 | 126 | cellular response to fibroblast growth factor stimulus |
| GO:0007507 | 0.022 | 0.718 | 60 | 46 | 300 | heart development |
| GO:0033157 | 0.022 | 0.588 | 26 | 17 | 132 | regulation of intracellular protein transport |
| GO:0046651 | 0.022 | 0.605 | 29 | 19 | 144 | lymphocyte proliferation |
| GO:0044763 | 0.022 | 0.879 | 1156 | 1124 | 5791 | single-organism cellular process |
| GO:0000188 | 0.023 | <0.0001 | 3 | 0 | 17 | inactivation of MAPK activity |
| GO:0003016 | 0.023 | <0.0001 | 3 | 0 | 17 | respiratory system process |
| GO:0006893 | 0.023 | <0.0001 | 3 | 0 | 17 | Golgi to plasma membrane transport |
| GO:0032770 | 0.023 | <0.0001 | 3 | 0 | 17 | positive regulation of monooxygenase activity |
| GO:0043277 | 0.023 | <0.0001 | 3 | 0 | 17 | apoptotic cell clearance |
| GO:0045061 | 0.023 | <0.0001 | 3 | 0 | 17 | thymic T cell selection |
| GO:0060071 | 0.023 | <0.0001 | 3 | 0 | 17 | Wnt receptor signaling pathway, planar cell polarity pathway |
| GO:0070741 | 0.023 | <0.0001 | 3 | 0 | 17 | response to interleukin-6 |
| GO:0072012 | 0.023 | <0.0001 | 3 | 0 | 17 | glomerulus vasculature development |
| GO:0090175 | 0.023 | <0.0001 | 3 | 0 | 17 | regulation of establishment of planar polarity |
| GO:0045576 | 0.023 | 0.250 | 7 | 2 | 34 | mast cell activation |
| GO:0046888 | 0.023 | 0.250 | 7 | 2 | 34 | negative regulation of hormone secretion |
| GO:0051492 | 0.023 | 0.250 | 7 | 2 | 34 | regulation of stress fiber assembly |
| GO:0000910 | 0.023 | 0.450 | 14 | 7 | 69 | cytokinesis |
| GO:0002064 | 0.023 | 0.450 | 14 | 7 | 69 | epithelial cell development |
| GO:0010927 | 0.023 | 0.491 | 16 | 9 | 82 | cellular component assembly involved in morphogenesis |
| GO:0002637 | 0.023 | 0.160 | 5 | 1 | 26 | regulation of immunoglobulin production |
| GO:0010743 | 0.023 | 0.160 | 5 | 1 | 26 | regulation of macrophage derived foam cell differentiation |
| GO:0019933 | 0.023 | 0.160 | 5 | 1 | 26 | cAMP-mediated signaling |
| GO:0042092 | 0.023 | 0.160 | 5 | 1 | 26 | type 2 immune response |
| GO:0042345 | 0.023 | 0.160 | 5 | 1 | 26 | regulation of NF-kappaB import into nucleus |
| GO:0042348 | 0.023 | 0.160 | 5 | 1 | 26 | NF-kappaB import into nucleus |
| GO:0048525 | 0.023 | 0.160 | 5 | 1 | 26 | negative regulation of viral reproduction |
| GO:0006631 | 0.023 | 0.650 | 37 | 26 | 185 | fatty acid metabolic process |
| GO:0071417 | 0.023 | 0.715 | 57 | 44 | 288 | cellular response to organic nitrogen |
| GO:0071705 | 0.024 | 0.756 | 80 | 64 | 399 | nitrogen compound transport |
| GO:0002520 | 0.024 | 0.767 | 88 | 72 | 443 | immune system development |
| GO:0009749 | 0.024 | 0.511 | 18 | 10 | 88 | response to glucose stimulus |
| GO:0048660 | 0.024 | 0.427 | 12 | 6 | 62 | regulation of smooth muscle cell proliferation |
| GO:0007015 | 0.024 | 0.629 | 32 | 22 | 161 | actin filament organization |
| GO:0071559 | 0.024 | 0.629 | 32 | 22 | 161 | response to transforming growth factor beta stimulus |
| GO:0045089 | 0.024 | 0.584 | 25 | 16 | 125 | positive regulation of innate immune response |
| GO:0038127 | 0.024 | 0.594 | 26 | 17 | 131 | ERBB signaling pathway |
| GO:0006027 | 0.024 | 0.315 | 8 | 3 | 41 | glycosaminoglycan catabolic process |
| GO:0030510 | 0.024 | 0.315 | 8 | 3 | 41 | regulation of BMP signaling pathway |
| GO:0033077 | 0.025 | 0.399 | 11 | 5 | 55 | T cell differentiation in thymus |
| GO:0006811 | 0.025 | 0.809 | 135 | 115 | 675 | ion transport |
| GO:0045582 | 0.025 | 0.363 | 10 | 4 | 48 | positive regulation of T cell differentiation |
| GO:0051971 | 0.025 | 0.363 | 10 | 4 | 48 | positive regulation of transmission of nerve impulse |
| GO:0051093 | 0.025 | 0.755 | 77 | 62 | 387 | negative regulation of developmental process |
| GO:0031214 | 0.026 | 0.498 | 16 | 9 | 81 | biomineral tissue development |
| GO:0010741 | 0.026 | 0.557 | 21 | 13 | 106 | negative regulation of intracellular protein kinase cascade |
| GO:0017015 | 0.026 | 0.458 | 14 | 7 | 68 | regulation of transforming growth factor beta receptor signaling pathway |
| GO:0071560 | 0.026 | 0.634 | 32 | 22 | 160 | cellular response to transforming growth factor beta stimulus |
| GO:0009612 | 0.026 | 0.569 | 22 | 14 | 112 | response to mechanical stimulus |
| GO:0010466 | 0.026 | 0.569 | 22 | 14 | 112 | negative regulation of peptidase activity |
| GO:0042327 | 0.026 | 0.774 | 90 | 74 | 452 | positive regulation of phosphorylation |
| GO:0030323 | 0.026 | 0.590 | 25 | 16 | 124 | respiratory tube development |
| GO:0070838 | 0.027 | 0.658 | 37 | 26 | 183 | divalent metal ion transport |
| GO:0032612 | 0.027 | 0.258 | 7 | 2 | 33 | interleukin-1 production |
| GO:0016049 | 0.027 | 0.712 | 52 | 40 | 263 | cell growth |
| GO:0034340 | 0.027 | 0.435 | 12 | 6 | 61 | response to type I interferon |
| GO:0060337 | 0.027 | 0.435 | 12 | 6 | 61 | type I interferon-mediated signaling pathway |
| GO:0071357 | 0.027 | 0.435 | 12 | 6 | 61 | cellular response to type I interferon |
| GO:0060759 | 0.027 | 0.483 | 15 | 8 | 74 | regulation of response to cytokine stimulus |
| GO:0033002 | 0.027 | 0.535 | 19 | 11 | 93 | muscle cell proliferation |
| GO:0050708 | 0.027 | 0.535 | 19 | 11 | 93 | regulation of protein secretion |
| GO:0002548 | 0.027 | 0.167 | 5 | 1 | 25 | monocyte chemotaxis |
| GO:0010043 | 0.027 | 0.167 | 5 | 1 | 25 | response to zinc ion |
| GO:0030004 | 0.027 | 0.167 | 5 | 1 | 25 | cellular monovalent inorganic cation homeostasis |
| GO:0032651 | 0.027 | 0.167 | 5 | 1 | 25 | regulation of interleukin-1 beta production |
| GO:0045071 | 0.027 | 0.167 | 5 | 1 | 25 | negative regulation of viral genome replication |
| GO:0045429 | 0.027 | 0.167 | 5 | 1 | 25 | positive regulation of nitric oxide biosynthetic process |
| GO:0060761 | 0.027 | 0.167 | 5 | 1 | 25 | negative regulation of response to cytokine stimulus |
| GO:0051345 | 0.028 | 0.746 | 68 | 54 | 341 | positive regulation of hydrolase activity |
| GO:0002221 | 0.028 | 0.550 | 20 | 12 | 99 | pattern recognition receptor signaling pathway |
| GO:0044711 | 0.028 | 0.736 | 62 | 49 | 313 | single-organism biosynthetic process |
| GO:0043434 | 0.028 | 0.724 | 57 | 44 | 285 | response to peptide hormone stimulus |
| GO:0007184 | 0.028 | <0.0001 | 3 | 0 | 16 | SMAD protein import into nucleus |
| GO:0033687 | 0.028 | <0.0001 | 3 | 0 | 16 | osteoblast proliferation |
| GO:0042994 | 0.028 | <0.0001 | 3 | 0 | 16 | cytoplasmic sequestering of transcription factor |
| GO:0048246 | 0.028 | <0.0001 | 3 | 0 | 16 | macrophage chemotaxis |
| GO:0042493 | 0.028 | 0.730 | 59 | 46 | 296 | response to drug |
| GO:0003158 | 0.029 | 0.324 | 8 | 3 | 40 | endothelium development |
| GO:0008543 | 0.029 | 0.575 | 22 | 14 | 111 | fibroblast growth factor receptor signaling pathway |
| GO:0032944 | 0.029 | 0.575 | 22 | 14 | 111 | regulation of mononuclear cell proliferation |
| GO:0015918 | 0.029 | 0.371 | 9 | 4 | 47 | sterol transport |
| GO:0030301 | 0.029 | 0.371 | 9 | 4 | 47 | cholesterol transport |
| GO:0042307 | 0.029 | 0.371 | 9 | 4 | 47 | positive regulation of protein import into nucleus |
| GO:0048489 | 0.029 | 0.371 | 9 | 4 | 47 | synaptic vesicle transport |
| GO:0007173 | 0.029 | 0.604 | 26 | 17 | 129 | epidermal growth factor receptor signaling pathway |
| GO:0006816 | 0.029 | 0.658 | 35 | 25 | 176 | calcium ion transport |
| GO:0032870 | 0.030 | 0.739 | 62 | 49 | 312 | cellular response to hormone stimulus |
| GO:0050801 | 0.030 | 0.791 | 101 | 84 | 504 | ion homeostasis |
| GO:0055082 | 0.031 | 0.791 | 99 | 83 | 498 | cellular chemical homeostasis |
| GO:0045667 | 0.031 | 0.491 | 15 | 8 | 73 | regulation of osteoblast differentiation |
| GO:0050818 | 0.031 | 0.443 | 12 | 6 | 60 | regulation of coagulation |
| GO:0009725 | 0.031 | 0.799 | 108 | 91 | 541 | response to hormone stimulus |
| GO:0002263 | 0.031 | 0.569 | 21 | 13 | 104 | cell activation involved in immune response |
| GO:0002366 | 0.031 | 0.569 | 21 | 13 | 104 | leukocyte activation involved in immune response |
| GO:0010951 | 0.032 | 0.581 | 22 | 14 | 110 | negative regulation of endopeptidase activity |
| GO:0031346 | 0.032 | 0.581 | 22 | 14 | 110 | positive regulation of cell projection organization |
| GO:0050670 | 0.032 | 0.581 | 22 | 14 | 110 | regulation of lymphocyte proliferation |
| GO:0051222 | 0.032 | 0.618 | 27 | 18 | 134 | positive regulation of protein transport |
| GO:0008206 | 0.032 | 0.266 | 6 | 2 | 32 | bile acid metabolic process |
| GO:0031032 | 0.032 | 0.266 | 6 | 2 | 32 | actomyosin structure organization |
| GO:0042490 | 0.032 | 0.266 | 6 | 2 | 32 | mechanoreceptor differentiation |
| GO:0045670 | 0.032 | 0.266 | 6 | 2 | 32 | regulation of osteoclast differentiation |
| GO:0048566 | 0.032 | 0.266 | 6 | 2 | 32 | embryonic digestive tract development |
| GO:0043085 | 0.032 | 0.822 | 139 | 120 | 696 | positive regulation of catalytic activity |
| GO:0002688 | 0.032 | 0.416 | 11 | 5 | 53 | regulation of leukocyte chemotaxis |
| GO:0031646 | 0.032 | 0.416 | 11 | 5 | 53 | positive regulation of neurological system process |
| GO:0051704 | 0.033 | 0.850 | 206 | 184 | 1034 | multi-organism process |
| GO:0006939 | 0.033 | 0.473 | 13 | 7 | 66 | smooth muscle contraction |
| GO:0002712 | 0.033 | 0.174 | 5 | 1 | 24 | regulation of B cell mediated immunity |
| GO:0009593 | 0.033 | 0.174 | 5 | 1 | 24 | detection of chemical stimulus |
| GO:0051489 | 0.033 | 0.174 | 5 | 1 | 24 | regulation of filopodium assembly |
| GO:0002673 | 0.033 | 0.380 | 9 | 4 | 46 | regulation of acute inflammatory response |
| GO:0045669 | 0.033 | 0.380 | 9 | 4 | 46 | positive regulation of osteoblast differentiation |
| GO:0042531 | 0.033 | 0.333 | 8 | 3 | 39 | positive regulation of tyrosine phosphorylation of STAT protein |
| GO:0045833 | 0.033 | 0.333 | 8 | 3 | 39 | negative regulation of lipid metabolic process |
| GO:0030030 | 0.034 | 0.819 | 130 | 112 | 652 | cell projection organization |
| GO:0032147 | 0.034 | 0.671 | 36 | 26 | 180 | activation of protein kinase activity |
| GO:0035150 | 0.034 | 0.563 | 19 | 12 | 97 | regulation of tube size |
| GO:0050880 | 0.034 | 0.563 | 19 | 12 | 97 | regulation of blood vessel size |
| GO:0051346 | 0.035 | 0.667 | 35 | 25 | 174 | negative regulation of hydrolase activity |
| GO:0001505 | 0.035 | 0.576 | 21 | 13 | 103 | regulation of neurotransmitter levels |
| GO:0071214 | 0.035 | 0.615 | 25 | 17 | 127 | cellular response to abiotic stimulus |
| GO:0046425 | 0.035 | 0.452 | 12 | 6 | 59 | regulation of JAK-STAT cascade |
| GO:2000242 | 0.035 | 0.452 | 12 | 6 | 59 | negative regulation of reproductive process |
| GO:0009713 | 0.035 | <0.0001 | 3 | 0 | 15 | catechol-containing compound biosynthetic process |
| GO:0010888 | 0.035 | <0.0001 | 3 | 0 | 15 | negative regulation of lipid storage |
| GO:0034312 | 0.035 | <0.0001 | 3 | 0 | 15 | diol biosynthetic process |
| GO:0035455 | 0.035 | <0.0001 | 3 | 0 | 15 | response to interferon-alpha |
| GO:0042423 | 0.035 | <0.0001 | 3 | 0 | 15 | catecholamine biosynthetic process |
| GO:0043001 | 0.035 | <0.0001 | 3 | 0 | 15 | Golgi to plasma membrane protein transport |
| GO:0045907 | 0.035 | <0.0001 | 3 | 0 | 15 | positive regulation of vasoconstriction |
| GO:0046189 | 0.035 | <0.0001 | 3 | 0 | 15 | phenol-containing compound biosynthetic process |
| GO:0032640 | 0.037 | 0.425 | 10 | 5 | 52 | tumor necrosis factor production |
| GO:0032680 | 0.037 | 0.425 | 10 | 5 | 52 | regulation of tumor necrosis factor production |
| GO:0051017 | 0.037 | 0.482 | 13 | 7 | 65 | actin filament bundle assembly |
| GO:0048468 | 0.037 | 0.857 | 214 | 192 | 1072 | cell development |
| GO:0007618 | 0.038 | 0.276 | 6 | 2 | 31 | mating |
| GO:0042116 | 0.038 | 0.276 | 6 | 2 | 31 | macrophage activation |
| GO:0043297 | 0.038 | 0.276 | 6 | 2 | 31 | apical junction assembly |
| GO:0060541 | 0.038 | 0.636 | 28 | 19 | 138 | respiratory system development |
| GO:0006913 | 0.038 | 0.729 | 52 | 40 | 258 | nucleocytoplasmic transport |
| GO:1901698 | 0.038 | 0.802 | 102 | 86 | 510 | response to nitrogen compound |
| GO:0032102 | 0.038 | 0.569 | 19 | 12 | 96 | negative regulation of response to external stimulus |
| GO:0055002 | 0.038 | 0.569 | 19 | 12 | 96 | striated muscle cell development |
| GO:0043407 | 0.038 | 0.390 | 9 | 4 | 45 | negative regulation of MAP kinase activity |
| GO:0050806 | 0.038 | 0.390 | 9 | 4 | 45 | positive regulation of synaptic transmission |
| GO:0072503 | 0.038 | 0.700 | 41 | 31 | 207 | cellular divalent inorganic cation homeostasis |
| GO:0002758 | 0.038 | 0.582 | 20 | 13 | 102 | innate immune response-activating signal transduction |
| GO:0006470 | 0.038 | 0.582 | 20 | 13 | 102 | protein dephosphorylation |
| GO:0006956 | 0.039 | 0.342 | 8 | 3 | 38 | complement activation |
| GO:0002762 | 0.040 | 0.182 | 5 | 1 | 23 | negative regulation of myeloid leukocyte differentiation |
| GO:0002889 | 0.040 | 0.182 | 5 | 1 | 23 | regulation of immunoglobulin mediated immune response |
| GO:0006775 | 0.040 | 0.182 | 5 | 1 | 23 | fat-soluble vitamin metabolic process |
| GO:0016101 | 0.040 | 0.182 | 5 | 1 | 23 | diterpenoid metabolic process |
| GO:0030279 | 0.040 | 0.182 | 5 | 1 | 23 | negative regulation of ossification |
| GO:0035567 | 0.040 | 0.182 | 5 | 1 | 23 | non-canonical Wnt receptor signaling pathway |
| GO:0043647 | 0.040 | 0.182 | 5 | 1 | 23 | inositol phosphate metabolic process |
| GO:0002695 | 0.040 | 0.528 | 15 | 9 | 77 | negative regulation of leukocyte activation |
| GO:0070665 | 0.040 | 0.528 | 15 | 9 | 77 | positive regulation of leukocyte proliferation |
| GO:0034329 | 0.041 | 0.641 | 27 | 19 | 137 | cell junction assembly |
| GO:0008283 | 0.042 | 0.866 | 232 | 210 | 1163 | cell proliferation |
| GO:0030001 | 0.042 | 0.772 | 71 | 58 | 356 | metal ion transport |
| GO:0008154 | 0.042 | 0.562 | 18 | 11 | 89 | actin polymerization or depolymerization |
| GO:0010721 | 0.042 | 0.562 | 18 | 11 | 89 | negative regulation of cell development |
| GO:0001508 | 0.042 | 0.627 | 25 | 17 | 125 | regulation of action potential |
| GO:0002755 | 0.042 | 0.490 | 13 | 7 | 64 | MyD88-dependent toll-like receptor signaling pathway |
| GO:0022602 | 0.042 | 0.490 | 13 | 7 | 64 | ovulation cycle process |
| GO:0006835 | 0.042 | 0.434 | 10 | 5 | 51 | dicarboxylic acid transport |
| GO:0050728 | 0.042 | 0.434 | 10 | 5 | 51 | negative regulation of inflammatory response |
| GO:0051209 | 0.042 | 0.434 | 10 | 5 | 51 | release of sequestered calcium ion into cytosol |
| GO:0051282 | 0.042 | 0.434 | 10 | 5 | 51 | regulation of sequestering of calcium ion |
| GO:0051283 | 0.042 | 0.434 | 10 | 5 | 51 | negative regulation of sequestering of calcium ion |
| GO:0055065 | 0.042 | 0.743 | 54 | 43 | 273 | metal ion homeostasis |
| GO:0031532 | 0.044 | 0.399 | 9 | 4 | 44 | actin cytoskeleton reorganization |
| GO:0001773 | 0.044 | <0.0001 | 3 | 0 | 14 | myeloid dendritic cell activation |
| GO:0002090 | 0.044 | <0.0001 | 3 | 0 | 14 | regulation of receptor internalization |
| GO:0006656 | 0.044 | <0.0001 | 3 | 0 | 14 | phosphatidylcholine biosynthetic process |
| GO:0010894 | 0.044 | <0.0001 | 3 | 0 | 14 | negative regulation of steroid biosynthetic process |
| GO:0032814 | 0.044 | <0.0001 | 3 | 0 | 14 | regulation of natural killer cell activation |
| GO:0032891 | 0.044 | <0.0001 | 3 | 0 | 14 | negative regulation of organic acid transport |
| GO:0032958 | 0.044 | <0.0001 | 3 | 0 | 14 | inositol phosphate biosynthetic process |
| GO:0033688 | 0.044 | <0.0001 | 3 | 0 | 14 | regulation of osteoblast proliferation |
| GO:0042359 | 0.044 | <0.0001 | 3 | 0 | 14 | vitamin D metabolic process |
| GO:0043330 | 0.044 | <0.0001 | 3 | 0 | 14 | response to exogenous dsRNA |
| GO:0044110 | 0.044 | <0.0001 | 3 | 0 | 14 | growth involved in symbiotic interaction |
| GO:0044116 | 0.044 | <0.0001 | 3 | 0 | 14 | growth of symbiont involved in interaction with host |
| GO:0044117 | 0.044 | <0.0001 | 3 | 0 | 14 | growth of symbiont in host |
| GO:0045671 | 0.044 | <0.0001 | 3 | 0 | 14 | negative regulation of osteoclast differentiation |
| GO:0045822 | 0.044 | <0.0001 | 3 | 0 | 14 | negative regulation of heart contraction |
| GO:0045939 | 0.044 | <0.0001 | 3 | 0 | 14 | negative regulation of steroid metabolic process |
| GO:0050755 | 0.044 | <0.0001 | 3 | 0 | 14 | chemokine metabolic process |
| GO:0051452 | 0.044 | <0.0001 | 3 | 0 | 14 | intracellular pH reduction |
| GO:0042158 | 0.044 | 0.285 | 6 | 2 | 30 | lipoprotein biosynthetic process |
| GO:0050848 | 0.044 | 0.285 | 6 | 2 | 30 | regulation of calcium-mediated signaling |
| GO:0051145 | 0.044 | 0.285 | 6 | 2 | 30 | smooth muscle cell differentiation |
| GO:0051169 | 0.045 | 0.740 | 52 | 41 | 261 | nuclear transport |
| GO:0007033 | 0.045 | 0.353 | 7 | 3 | 37 | vacuole organization |
| GO:0010595 | 0.045 | 0.353 | 7 | 3 | 37 | positive regulation of endothelial cell migration |
| GO:0033627 | 0.045 | 0.353 | 7 | 3 | 37 | cell adhesion mediated by integrin |
| GO:0048010 | 0.045 | 0.353 | 7 | 3 | 37 | vascular endothelial growth factor receptor signaling pathway |
| GO:0034130 | 0.045 | 0.470 | 11 | 6 | 57 | toll-like receptor 1 signaling pathway |
| GO:0046632 | 0.045 | 0.470 | 11 | 6 | 57 | alpha-beta T cell differentiation |
| GO:0030258 | 0.047 | 0.607 | 21 | 14 | 106 | lipid modification |
| GO:0001738 | 0.048 | 0.190 | 4 | 1 | 22 | morphogenesis of a polarized epithelium |
| GO:0002792 | 0.048 | 0.190 | 4 | 1 | 22 | negative regulation of peptide secretion |
| GO:0033003 | 0.048 | 0.190 | 4 | 1 | 22 | regulation of mast cell activation |
| GO:0034121 | 0.048 | 0.190 | 4 | 1 | 22 | regulation of toll-like receptor signaling pathway |
| GO:0045103 | 0.048 | 0.190 | 4 | 1 | 22 | intermediate filament-based process |
| GO:0045104 | 0.048 | 0.190 | 4 | 1 | 22 | intermediate filament cytoskeleton organization |
| GO:0010921 | 0.048 | 0.444 | 10 | 5 | 50 | regulation of phosphatase activity |
| GO:0046634 | 0.048 | 0.444 | 10 | 5 | 50 | regulation of alpha-beta T cell activation |
| GO:0046578 | 0.049 | 0.698 | 36 | 27 | 181 | regulation of Ras protein signal transduction |
| GO:0006874 | 0.049 | 0.710 | 40 | 30 | 198 | cellular calcium ion homeostasis |
| GO:0001959 | 0.049 | 0.523 | 14 | 8 | 69 | regulation of cytokine-mediated signaling pathway |
| GO:0032535 | 0.049 | 0.646 | 26 | 18 | 129 | regulation of cellular component size |
