# Supplementary material for: Hidden Treasures in “Ancient” Microarrays: Gene-Expression Portrays Biology and Potential Resistance Pathways of Major Lung Cancer Subtypes and Normal Tissue
Source: Front Oncol. 2014 Sep 29;4:251. doi: 10.3389/fonc.2014.00251 (PMC4178426; doi:10.3389/fonc.2014.00251)
Supplement: Figure S1 — Clinically relevant differentially expressed pathways in each tissue type versus the rest. The pathway maps are arranged according to the scheme in the left-top corner. Red indicates overexpression in genes, dark green downregulation. In the Cell Cycle pathway, tumor suppressor genes were overexpressed in normal lung tissue while oncogenes and tumor driving cyclins were overexpressed in cancers. The gene thymidylate synthase (TYMS or TS, belonging to the pathway “one carbon pool by folate”) is relevant for tumor growth and is also a treatment target. Notably, TYMS was overexpressed only in the tumors that are generally refractory to the drug pemetrexed, as the squamous and the small-cell lung cancer. TYMS was not overexpressed in mesothelioma, but it is known that TYMS expression is highly variable in this cancer. In the ERBB pathway, the ERBB2/HER2 and ERBB3/HER3 were overexpressed in adenocarcinoma, while the ERBB4 was overexpressed in the carcinoids. [file Data_Sheet_1.ZIP › File S3.ZIP!/GO Analysis/Normals vs Rest Enrichment Analysis/Underrepresented GO terms of Up-regulated Genes.html]

Gene to GO BP test for under-representation

| GOBPID | Pvalue | OddsRatio | ExpCount | Count | Size | Term |
| --- | --- | --- | --- | --- | --- | --- |
| GO:0016071 | <0.0001 | 0.247 | 65 | 19 | 408 | mRNA metabolic process |
| GO:0006396 | <0.0001 | 0.241 | 60 | 17 | 375 | RNA processing |
| GO:0044237 | <0.0001 | 0.673 | 790 | 691 | 4977 | cellular metabolic process |
| GO:0006725 | <0.0001 | 0.678 | 498 | 403 | 3135 | cellular aromatic compound metabolic process |
| GO:0044238 | <0.0001 | 0.687 | 800 | 707 | 5040 | primary metabolic process |
| GO:0046483 | <0.0001 | 0.686 | 496 | 404 | 3122 | heterocycle metabolic process |
| GO:0008380 | <0.0001 | 0.178 | 33 | 7 | 210 | RNA splicing |
| GO:1901360 | <0.0001 | 0.692 | 520 | 429 | 3278 | organic cyclic compound metabolic process |
| GO:0006139 | <0.0001 | 0.695 | 480 | 393 | 3026 | nucleobase-containing compound metabolic process |
| GO:0044260 | <0.0001 | 0.706 | 595 | 505 | 3749 | cellular macromolecule metabolic process |
| GO:0071704 | <0.0001 | 0.701 | 822 | 736 | 5180 | organic substance metabolic process |
| GO:0006281 | <0.0001 | 0.262 | 39 | 12 | 248 | DNA repair |
| GO:0034660 | <0.0001 | 0.081 | 21 | 2 | 131 | ncRNA metabolic process |
| GO:0090304 | <0.0001 | 0.690 | 395 | 315 | 2488 | nucleic acid metabolic process |
| GO:0006397 | <0.0001 | 0.275 | 41 | 13 | 257 | mRNA processing |
| GO:0008152 | <0.0001 | 0.707 | 864 | 783 | 5441 | metabolic process |
| GO:0044265 | <0.0001 | 0.444 | 73 | 37 | 463 | cellular macromolecule catabolic process |
| GO:0000075 | <0.0001 | 0.206 | 29 | 7 | 183 | cell cycle checkpoint |
| GO:0006807 | <0.0001 | 0.729 | 550 | 470 | 3468 | nitrogen compound metabolic process |
| GO:0000375 | <0.0001 | 0.175 | 24 | 5 | 154 | RNA splicing, via transesterification reactions |
| GO:0034641 | <0.0001 | 0.731 | 520 | 442 | 3277 | cellular nitrogen compound metabolic process |
| GO:0000377 | <0.0001 | 0.181 | 24 | 5 | 149 | RNA splicing, via transesterification reactions with bulged adenosine as nucleophile |
| GO:0000398 | <0.0001 | 0.181 | 24 | 5 | 149 | mRNA splicing, via spliceosome |
| GO:0006613 | <0.0001 | <0.0001 | 12 | 0 | 77 | cotranslational protein targeting to membrane |
| GO:0006412 | <0.0001 | 0.362 | 44 | 18 | 275 | translation |
| GO:0022403 | <0.0001 | 0.523 | 89 | 52 | 560 | cell cycle phase |
| GO:0051276 | <0.0001 | 0.473 | 70 | 37 | 438 | chromosome organization |
| GO:0034470 | <0.0001 | 0.057 | 15 | 1 | 92 | ncRNA processing |
| GO:0006614 | <0.0001 | <0.0001 | 12 | 0 | 75 | SRP-dependent cotranslational protein targeting to membrane |
| GO:0070972 | <0.0001 | 0.060 | 14 | 1 | 88 | protein localization to endoplasmic reticulum |
| GO:0006414 | <0.0001 | <0.0001 | 11 | 0 | 71 | translational elongation |
| GO:0000216 | <0.0001 | <0.0001 | 11 | 0 | 70 | M/G1 transition of mitotic cell cycle |
| GO:0031145 | <0.0001 | <0.0001 | 11 | 0 | 70 | anaphase-promoting complex-dependent proteasomal ubiquitin-dependent protein catabolic process |
| GO:0071156 | <0.0001 | 0.304 | 32 | 11 | 199 | regulation of cell cycle arrest |
| GO:0043170 | <0.0001 | 0.761 | 662 | 590 | 4168 | macromolecule metabolic process |
| GO:0051351 | <0.0001 | <0.0001 | 11 | 0 | 69 | positive regulation of ligase activity |
| GO:0000184 | <0.0001 | 0.063 | 13 | 1 | 84 | nuclear-transcribed mRNA catabolic process, nonsense-mediated decay |
| GO:0022613 | <0.0001 | 0.204 | 21 | 5 | 133 | ribonucleoprotein complex biogenesis |
| GO:0006974 | <0.0001 | 0.491 | 66 | 36 | 413 | response to DNA damage stimulus |
| GO:0051443 | <0.0001 | <0.0001 | 11 | 0 | 67 | positive regulation of ubiquitin-protein ligase activity |
| GO:0051603 | <0.0001 | 0.395 | 43 | 19 | 268 | proteolysis involved in cellular protein catabolic process |
| GO:0051321 | <0.0001 | 0.113 | 15 | 2 | 95 | meiotic cell cycle |
| GO:0051439 | <0.0001 | <0.0001 | 10 | 0 | 65 | regulation of ubiquitin-protein ligase activity involved in mitotic cell cycle |
| GO:0006511 | <0.0001 | 0.385 | 39 | 17 | 246 | ubiquitin-dependent protein catabolic process |
| GO:0051340 | <0.0001 | 0.066 | 13 | 1 | 80 | regulation of ligase activity |
| GO:0000278 | <0.0001 | 0.556 | 84 | 52 | 532 | mitotic cell cycle |
| GO:0042254 | <0.0001 | 0.067 | 13 | 1 | 79 | ribosome biogenesis |
| GO:0043632 | <0.0001 | 0.399 | 40 | 18 | 252 | modification-dependent macromolecule catabolic process |
| GO:0051438 | <0.0001 | 0.068 | 12 | 1 | 78 | regulation of ubiquitin-protein ligase activity |
| GO:0072599 | <0.0001 | 0.068 | 12 | 1 | 78 | establishment of protein localization to endoplasmic reticulum |
| GO:0006259 | <0.0001 | 0.556 | 83 | 51 | 522 | DNA metabolic process |
| GO:0007126 | <0.0001 | 0.118 | 14 | 2 | 91 | meiosis |
| GO:0051327 | <0.0001 | 0.118 | 14 | 2 | 91 | M phase of meiotic cell cycle |
| GO:0045047 | <0.0001 | 0.069 | 12 | 1 | 77 | protein targeting to ER |
| GO:0019941 | <0.0001 | 0.403 | 40 | 18 | 250 | modification-dependent protein catabolic process |
| GO:0051437 | <0.0001 | <0.0001 | 10 | 0 | 61 | positive regulation of ubiquitin-protein ligase activity involved in mitotic cell cycle |
| GO:0072594 | <0.0001 | 0.243 | 21 | 6 | 135 | establishment of protein localization to organelle |
| GO:0051329 | <0.0001 | 0.457 | 49 | 25 | 308 | interphase of mitotic cell cycle |
| GO:0000209 | <0.0001 | 0.225 | 19 | 5 | 121 | protein polyubiquitination |
| GO:0007093 | <0.0001 | 0.197 | 17 | 4 | 110 | mitotic cell cycle checkpoint |
| GO:0051325 | <0.0001 | 0.471 | 50 | 26 | 312 | interphase |
| GO:0016070 | <0.0001 | 0.759 | 350 | 294 | 2204 | RNA metabolic process |
| GO:0044257 | <0.0001 | 0.449 | 44 | 22 | 276 | cellular protein catabolic process |
| GO:0051436 | <0.0001 | <0.0001 | 9 | 0 | 56 | negative regulation of ubiquitin-protein ligase activity involved in mitotic cell cycle |
| GO:0006354 | <0.0001 | 0.128 | 13 | 2 | 84 | DNA-dependent transcription, elongation |
| GO:0006364 | <0.0001 | <0.0001 | 9 | 0 | 55 | rRNA processing |
| GO:0016072 | <0.0001 | <0.0001 | 9 | 0 | 55 | rRNA metabolic process |
| GO:0022402 | <0.0001 | 0.632 | 111 | 77 | 700 | cell cycle process |
| GO:0006310 | <0.0001 | 0.281 | 22 | 7 | 137 | DNA recombination |
| GO:0010498 | <0.0001 | 0.337 | 26 | 10 | 165 | proteasomal protein catabolic process |
| GO:0034645 | <0.0001 | 0.772 | 373 | 318 | 2348 | cellular macromolecule biosynthetic process |
| GO:0000956 | <0.0001 | 0.240 | 18 | 5 | 114 | nuclear-transcribed mRNA catabolic process |
| GO:0000084 | <0.0001 | 0.268 | 20 | 6 | 123 | S phase of mitotic cell cycle |
| GO:0009057 | <0.0001 | 0.623 | 95 | 65 | 601 | macromolecule catabolic process |
| GO:0010467 | <0.0001 | 0.781 | 396 | 342 | 2497 | gene expression |
| GO:0000279 | <0.0001 | 0.506 | 50 | 28 | 315 | M phase |
| GO:0045333 | <0.0001 | 0.249 | 17 | 5 | 110 | cellular respiration |
| GO:0006415 | <0.0001 | 0.083 | 10 | 1 | 64 | translational termination |
| GO:0006289 | <0.0001 | 0.085 | 10 | 1 | 63 | nucleotide-excision repair |
| GO:0043161 | <0.0001 | 0.355 | 25 | 10 | 157 | proteasomal ubiquitin-dependent protein catabolic process |
| GO:0051320 | <0.0001 | 0.303 | 20 | 7 | 128 | S phase |
| GO:0072395 | <0.0001 | 0.086 | 10 | 1 | 62 | signal transduction involved in cell cycle checkpoint |
| GO:0022904 | <0.0001 | 0.146 | 12 | 2 | 74 | respiratory electron transport chain |
| GO:0051352 | <0.0001 | 0.088 | 10 | 1 | 61 | negative regulation of ligase activity |
| GO:0051444 | <0.0001 | 0.088 | 10 | 1 | 61 | negative regulation of ubiquitin-protein ligase activity |
| GO:0072401 | <0.0001 | 0.088 | 10 | 1 | 61 | signal transduction involved in DNA integrity checkpoint |
| GO:0072404 | <0.0001 | 0.088 | 10 | 1 | 61 | signal transduction involved in G1/S transition checkpoint |
| GO:0072422 | <0.0001 | 0.088 | 10 | 1 | 61 | signal transduction involved in DNA damage checkpoint |
| GO:0006091 | <0.0001 | 0.538 | 52 | 31 | 330 | generation of precursor metabolites and energy |
| GO:0006977 | <0.0001 | 0.089 | 10 | 1 | 60 | DNA damage response, signal transduction by p53 class mediator resulting in cell cycle arrest |
| GO:0072413 | <0.0001 | 0.089 | 10 | 1 | 60 | signal transduction involved in mitotic cell cycle checkpoint |
| GO:0072431 | <0.0001 | 0.089 | 10 | 1 | 60 | signal transduction involved in mitotic cell cycle G1/S transition DNA damage checkpoint |
| GO:0072474 | <0.0001 | 0.089 | 10 | 1 | 60 | signal transduction involved in mitotic cell cycle G1/S checkpoint |
| GO:0031570 | <0.0001 | 0.262 | 17 | 5 | 105 | DNA integrity checkpoint |
| GO:0006283 | 0.001 | <0.0001 | 7 | 0 | 43 | transcription-coupled nucleotide-excision repair |
| GO:0019080 | 0.001 | 0.322 | 19 | 7 | 121 | viral genome expression |
| GO:0019083 | 0.001 | 0.322 | 19 | 7 | 121 | viral transcription |
| GO:0070647 | 0.001 | 0.578 | 59 | 37 | 369 | protein modification by small protein conjugation or removal |
| GO:0000077 | 0.001 | 0.276 | 16 | 5 | 100 | DNA damage checkpoint |
| GO:0006520 | 0.001 | 0.543 | 47 | 28 | 296 | cellular amino acid metabolic process |
| GO:0006368 | 0.001 | 0.162 | 11 | 2 | 67 | transcription elongation from RNA polymerase II promoter |
| GO:1901576 | 0.001 | 0.816 | 478 | 429 | 3014 | organic substance biosynthetic process |
| GO:0006325 | 0.001 | 0.568 | 52 | 32 | 325 | chromatin organization |
| GO:0006839 | 0.001 | 0.216 | 12 | 3 | 76 | mitochondrial transport |
| GO:0006521 | 0.001 | 0.101 | 8 | 1 | 53 | regulation of cellular amino acid metabolic process |
| GO:0009058 | 0.001 | 0.822 | 485 | 437 | 3056 | biosynthetic process |
| GO:0006399 | 0.001 | 0.103 | 8 | 1 | 52 | tRNA metabolic process |
| GO:0009059 | 0.001 | 0.813 | 387 | 342 | 2439 | macromolecule biosynthetic process |
| GO:0048285 | 0.001 | 0.517 | 37 | 21 | 233 | organelle fission |
| GO:0016052 | 0.001 | 0.321 | 17 | 6 | 104 | carbohydrate catabolic process |
| GO:0031571 | 0.001 | 0.172 | 10 | 2 | 63 | mitotic cell cycle G1/S transition DNA damage checkpoint |
| GO:0006413 | 0.002 | 0.346 | 18 | 7 | 113 | translational initiation |
| GO:0030330 | 0.002 | 0.295 | 15 | 5 | 94 | DNA damage response, signal transduction by p53 class mediator |
| GO:0042770 | 0.002 | 0.325 | 16 | 6 | 103 | signal transduction in response to DNA damage |
| GO:0044249 | 0.002 | 0.826 | 468 | 422 | 2950 | cellular biosynthetic process |
| GO:0000086 | 0.002 | 0.350 | 18 | 7 | 112 | G2/M transition of mitotic cell cycle |
| GO:0006402 | 0.002 | 0.371 | 19 | 8 | 121 | mRNA catabolic process |
| GO:0010564 | 0.002 | 0.581 | 49 | 31 | 309 | regulation of cell cycle process |
| GO:0015980 | 0.002 | 0.538 | 39 | 23 | 246 | energy derivation by oxidation of organic compounds |
| GO:0070085 | 0.002 | 0.424 | 23 | 11 | 147 | glycosylation |
| GO:0044724 | 0.002 | 0.328 | 16 | 6 | 102 | single-organism carbohydrate catabolic process |
| GO:0007050 | 0.002 | 0.563 | 44 | 27 | 277 | cell cycle arrest |
| GO:0006401 | 0.002 | 0.409 | 22 | 10 | 138 | RNA catabolic process |
| GO:0006486 | 0.002 | 0.427 | 23 | 11 | 146 | protein glycosylation |
| GO:0016054 | 0.002 | 0.427 | 23 | 11 | 146 | organic acid catabolic process |
| GO:0043413 | 0.002 | 0.427 | 23 | 11 | 146 | macromolecule glycosylation |
| GO:0046395 | 0.002 | 0.427 | 23 | 11 | 146 | carboxylic acid catabolic process |
| GO:0000723 | 0.002 | 0.110 | 8 | 1 | 49 | telomere maintenance |
| GO:0032200 | 0.002 | 0.110 | 8 | 1 | 49 | telomere organization |
| GO:0006383 | 0.002 | <0.0001 | 6 | 0 | 35 | transcription from RNA polymerase III promoter |
| GO:0006612 | 0.002 | 0.360 | 17 | 7 | 109 | protein targeting to membrane |
| GO:0006403 | 0.002 | 0.309 | 14 | 5 | 90 | RNA localization |
| GO:0031575 | 0.003 | 0.235 | 11 | 3 | 70 | mitotic cell cycle G1/S transition checkpoint |
| GO:0048610 | 0.003 | 0.637 | 60 | 41 | 376 | cellular process involved in reproduction |
| GO:0071824 | 0.004 | 0.320 | 14 | 5 | 87 | protein-DNA complex subunit organization |
| GO:0006284 | 0.004 | <0.0001 | 5 | 0 | 32 | base-excision repair |
| GO:0010833 | 0.004 | <0.0001 | 5 | 0 | 32 | telomere maintenance via telomere lengthening |
| GO:0022616 | 0.004 | <0.0001 | 5 | 0 | 32 | DNA strand elongation |
| GO:0072329 | 0.004 | 0.251 | 10 | 3 | 66 | monocarboxylic acid catabolic process |
| GO:0000280 | 0.004 | 0.555 | 35 | 21 | 219 | nuclear division |
| GO:0007067 | 0.004 | 0.555 | 35 | 21 | 219 | mitosis |
| GO:0050657 | 0.005 | 0.329 | 13 | 5 | 85 | nucleic acid transport |
| GO:0050658 | 0.005 | 0.329 | 13 | 5 | 85 | RNA transport |
| GO:0051236 | 0.005 | 0.329 | 13 | 5 | 85 | establishment of RNA localization |
| GO:0022900 | 0.005 | 0.358 | 15 | 6 | 94 | electron transport chain |
| GO:0006487 | 0.005 | 0.255 | 10 | 3 | 65 | protein N-linked glycosylation |
| GO:0000087 | 0.005 | 0.567 | 36 | 22 | 225 | M phase of mitotic cell cycle |
| GO:0071779 | 0.005 | 0.301 | 12 | 4 | 74 | G1/S transition checkpoint |
| GO:0006271 | 0.006 | <0.0001 | 5 | 0 | 30 | DNA strand elongation involved in DNA replication |
| GO:0006302 | 0.006 | 0.305 | 12 | 4 | 73 | double-strand break repair |
| GO:0043687 | 0.006 | 0.416 | 17 | 8 | 109 | post-translational protein modification |
| GO:0000082 | 0.006 | 0.506 | 25 | 14 | 159 | G1/S transition of mitotic cell cycle |
| GO:0030163 | 0.006 | 0.654 | 56 | 39 | 350 | protein catabolic process |
| GO:0015931 | 0.006 | 0.396 | 16 | 7 | 100 | nucleobase-containing compound transport |
| GO:0009452 | 0.007 | <0.0001 | 5 | 0 | 29 | 7-methylguanosine RNA capping |
| GO:0036260 | 0.007 | <0.0001 | 5 | 0 | 29 | RNA capping |
| GO:0072331 | 0.007 | 0.420 | 17 | 8 | 108 | signal transduction by p53 class mediator |
| GO:0046365 | 0.007 | 0.310 | 11 | 4 | 72 | monosaccharide catabolic process |
| GO:0044248 | 0.007 | 0.796 | 182 | 154 | 1148 | cellular catabolic process |
| GO:0019098 | 0.007 | 0.211 | 8 | 2 | 52 | reproductive behavior |
| GO:0016568 | 0.007 | 0.624 | 45 | 30 | 281 | chromatin modification |
| GO:0032446 | 0.007 | 0.652 | 53 | 37 | 333 | protein modification by small protein conjugation |
| GO:0007600 | 0.008 | 0.630 | 46 | 31 | 288 | sensory perception |
| GO:0071158 | 0.008 | 0.314 | 11 | 4 | 71 | positive regulation of cell cycle arrest |
| GO:0006370 | 0.008 | <0.0001 | 4 | 0 | 28 | 7-methylguanosine mRNA capping |
| GO:0008033 | 0.008 | <0.0001 | 4 | 0 | 28 | tRNA processing |
| GO:0031397 | 0.008 | 0.351 | 13 | 5 | 80 | negative regulation of protein ubiquitination |
| GO:0018196 | 0.008 | 0.272 | 10 | 3 | 61 | peptidyl-asparagine modification |
| GO:0018279 | 0.008 | 0.272 | 10 | 3 | 61 | protein N-linked glycosylation via asparagine |
| GO:0016567 | 0.008 | 0.646 | 49 | 34 | 309 | protein ubiquitination |
| GO:1901362 | 0.008 | 0.844 | 350 | 315 | 2205 | organic cyclic compound biosynthetic process |
| GO:1901575 | 0.009 | 0.809 | 198 | 170 | 1249 | organic substance catabolic process |
| GO:0019320 | 0.009 | 0.319 | 11 | 4 | 70 | hexose catabolic process |
| GO:0006903 | 0.009 | <0.0001 | 4 | 0 | 27 | vesicle targeting |
| GO:0007131 | 0.009 | <0.0001 | 4 | 0 | 27 | reciprocal meiotic recombination |
| GO:0035825 | 0.009 | <0.0001 | 4 | 0 | 27 | reciprocal DNA recombination |
| GO:0031577 | 0.011 | <0.0001 | 4 | 0 | 26 | spindle checkpoint |
| GO:0044704 | 0.011 | 0.143 | 6 | 1 | 38 | single-organism reproductive behavior |
| GO:0018130 | 0.011 | 0.849 | 337 | 304 | 2123 | heterocycle biosynthetic process |
| GO:0019438 | 0.011 | 0.849 | 337 | 304 | 2123 | aromatic compound biosynthetic process |
| GO:0007127 | 0.012 | 0.229 | 8 | 2 | 48 | meiosis I |
| GO:0050434 | 0.012 | 0.229 | 8 | 2 | 48 | positive regulation of viral transcription |
| GO:0007601 | 0.013 | 0.512 | 21 | 12 | 135 | visual perception |
| GO:0050953 | 0.013 | 0.512 | 21 | 12 | 135 | sensory perception of light stimulus |
| GO:0051028 | 0.013 | 0.371 | 12 | 5 | 76 | mRNA transport |
| GO:0051650 | 0.013 | 0.147 | 6 | 1 | 37 | establishment of vesicle localization |
| GO:0009062 | 0.014 | 0.293 | 9 | 3 | 57 | fatty acid catabolic process |
| GO:0006261 | 0.014 | 0.376 | 12 | 5 | 75 | DNA-dependent DNA replication |
| GO:2000243 | 0.015 | 0.433 | 15 | 7 | 92 | positive regulation of reproductive process |
| GO:0032201 | 0.016 | <0.0001 | 4 | 0 | 24 | telomere maintenance via semi-conservative replication |
| GO:0006508 | 0.016 | 0.758 | 92 | 74 | 582 | proteolysis |
| GO:0000236 | 0.016 | 0.240 | 7 | 2 | 46 | mitotic prometaphase |
| GO:0009123 | 0.018 | 0.155 | 6 | 1 | 35 | nucleoside monophosphate metabolic process |
| GO:0034728 | 0.018 | 0.351 | 10 | 4 | 64 | nucleosome organization |
| GO:0044723 | 0.018 | 0.710 | 58 | 44 | 368 | single-organism carbohydrate metabolic process |
| GO:0071174 | 0.019 | <0.0001 | 4 | 0 | 23 | mitotic cell cycle spindle checkpoint |
| GO:0071103 | 0.019 | 0.488 | 17 | 9 | 106 | DNA conformation change |
| GO:0034654 | 0.020 | 0.862 | 331 | 301 | 2083 | nucleobase-containing compound biosynthetic process |
| GO:0046034 | 0.021 | 0.473 | 15 | 8 | 97 | ATP metabolic process |
| GO:0045786 | 0.021 | 0.701 | 52 | 39 | 330 | negative regulation of cell cycle |
| GO:0006119 | 0.021 | 0.251 | 7 | 2 | 44 | oxidative phosphorylation |
| GO:0009101 | 0.021 | 0.608 | 29 | 19 | 183 | glycoprotein biosynthetic process |
| GO:0009056 | 0.021 | 0.839 | 212 | 187 | 1335 | catabolic process |
| GO:0044764 | 0.022 | 0.763 | 86 | 69 | 540 | multi-organism cellular process |
| GO:0009126 | 0.022 | <0.0001 | 3 | 0 | 22 | purine nucleoside monophosphate metabolic process |
| GO:0009167 | 0.022 | <0.0001 | 3 | 0 | 22 | purine ribonucleoside monophosphate metabolic process |
| GO:0046883 | 0.022 | 0.566 | 23 | 14 | 144 | regulation of hormone secretion |
| GO:0046887 | 0.023 | 0.316 | 8 | 3 | 53 | positive regulation of hormone secretion |
| GO:0007059 | 0.024 | 0.433 | 13 | 6 | 79 | chromosome segregation |
| GO:0016032 | 0.024 | 0.767 | 85 | 69 | 538 | viral reproduction |
| GO:0006312 | 0.024 | 0.165 | 5 | 1 | 33 | mitotic recombination |
| GO:0007632 | 0.024 | 0.165 | 5 | 1 | 33 | visual behavior |
| GO:0007338 | 0.024 | 0.257 | 7 | 2 | 43 | single fertilization |
| GO:0019058 | 0.024 | 0.616 | 29 | 19 | 181 | viral infectious cycle |
| GO:0007346 | 0.025 | 0.659 | 37 | 26 | 233 | regulation of mitotic cell cycle |
| GO:0031396 | 0.025 | 0.560 | 21 | 13 | 135 | regulation of protein ubiquitination |
| GO:0006333 | 0.025 | 0.405 | 11 | 5 | 70 | chromatin assembly or disassembly |
| GO:0065004 | 0.025 | 0.405 | 11 | 5 | 70 | protein-DNA complex assembly |
| GO:0051188 | 0.026 | 0.439 | 12 | 6 | 78 | cofactor biosynthetic process |
| GO:0044271 | 0.026 | 0.871 | 344 | 316 | 2170 | cellular nitrogen compound biosynthetic process |
| GO:0007094 | 0.026 | <0.0001 | 3 | 0 | 21 | mitotic cell cycle spindle assembly checkpoint |
| GO:0009072 | 0.026 | <0.0001 | 3 | 0 | 21 | aromatic amino acid family metabolic process |
| GO:0045841 | 0.026 | <0.0001 | 3 | 0 | 21 | negative regulation of mitotic metaphase/anaphase transition |
| GO:0071173 | 0.026 | <0.0001 | 3 | 0 | 21 | spindle assembly checkpoint |
| GO:0043467 | 0.028 | 0.264 | 7 | 2 | 42 | regulation of generation of precursor metabolites and energy |
| GO:0006353 | 0.028 | 0.377 | 10 | 4 | 60 | DNA-dependent transcription, termination |
| GO:0019318 | 0.030 | 0.641 | 31 | 21 | 193 | hexose metabolic process |
| GO:0022618 | 0.031 | 0.418 | 11 | 5 | 68 | ribonucleoprotein complex assembly |
| GO:0000387 | 0.031 | <0.0001 | 3 | 0 | 20 | spliceosomal snRNP assembly |
| GO:0007031 | 0.031 | <0.0001 | 3 | 0 | 20 | peroxisome organization |
| GO:0009262 | 0.031 | <0.0001 | 3 | 0 | 20 | deoxyribonucleotide metabolic process |
| GO:0035773 | 0.031 | <0.0001 | 3 | 0 | 20 | insulin secretion involved in cellular response to glucose stimulus |
| GO:0043094 | 0.031 | <0.0001 | 3 | 0 | 20 | cellular metabolic compound salvage |
| GO:0051568 | 0.031 | <0.0001 | 3 | 0 | 20 | histone H3-K4 methylation |
| GO:0061178 | 0.031 | <0.0001 | 3 | 0 | 20 | regulation of insulin secretion involved in cellular response to glucose stimulus |
| GO:0006200 | 0.032 | 0.383 | 9 | 4 | 59 | ATP catabolic process |
| GO:0006282 | 0.032 | 0.176 | 5 | 1 | 31 | regulation of DNA repair |
| GO:0007091 | 0.032 | 0.176 | 5 | 1 | 31 | mitotic metaphase/anaphase transition |
| GO:0009161 | 0.032 | 0.176 | 5 | 1 | 31 | ribonucleoside monophosphate metabolic process |
| GO:0055114 | 0.033 | 0.747 | 62 | 49 | 392 | oxidation-reduction process |
| GO:0006323 | 0.035 | 0.458 | 12 | 6 | 75 | DNA packaging |
| GO:0005975 | 0.036 | 0.777 | 78 | 64 | 494 | carbohydrate metabolic process |
| GO:0001708 | 0.036 | 0.344 | 8 | 3 | 49 | cell fate specification |
| GO:0048199 | 0.037 | <0.0001 | 3 | 0 | 19 | vesicle targeting, to, from or within Golgi |
| GO:0065002 | 0.037 | <0.0001 | 3 | 0 | 19 | intracellular protein transmembrane transport |
| GO:0071806 | 0.037 | <0.0001 | 3 | 0 | 19 | protein transmembrane transport |
| GO:0006007 | 0.040 | 0.398 | 9 | 4 | 57 | glucose catabolic process |
| GO:0009108 | 0.040 | 0.398 | 9 | 4 | 57 | coenzyme biosynthetic process |
| GO:0048663 | 0.041 | 0.285 | 6 | 2 | 39 | neuron fate commitment |
| GO:0009112 | 0.041 | 0.352 | 8 | 3 | 48 | nucleobase metabolic process |
| GO:0008542 | 0.043 | 0.189 | 5 | 1 | 29 | visual learning |
| GO:0009124 | 0.043 | 0.189 | 5 | 1 | 29 | nucleoside monophosphate biosynthetic process |
| GO:0034968 | 0.043 | 0.189 | 5 | 1 | 29 | histone lysine methylation |
| GO:0007005 | 0.043 | 0.616 | 23 | 15 | 143 | mitochondrion organization |
| GO:0009063 | 0.043 | 0.499 | 13 | 7 | 81 | cellular amino acid catabolic process |
| GO:0000003 | 0.044 | 0.852 | 176 | 156 | 1106 | reproduction |
| GO:0006260 | 0.044 | 0.665 | 30 | 21 | 187 | DNA replication |
| GO:0009127 | 0.044 | <0.0001 | 3 | 0 | 18 | purine nucleoside monophosphate biosynthetic process |
| GO:0009168 | 0.044 | <0.0001 | 3 | 0 | 18 | purine ribonucleoside monophosphate biosynthetic process |
| GO:0006308 | 0.044 | 0.406 | 9 | 4 | 56 | DNA catabolic process |
| GO:0009060 | 0.046 | 0.293 | 6 | 2 | 38 | aerobic respiration |
| GO:0006338 | 0.046 | 0.447 | 10 | 5 | 64 | chromatin remodeling |
| GO:0007602 | 0.049 | 0.196 | 4 | 1 | 28 | phototransduction |
| GO:0043038 | 0.049 | 0.196 | 4 | 1 | 28 | amino acid activation |
| GO:0043039 | 0.049 | 0.196 | 4 | 1 | 28 | tRNA aminoacylation |
