# Supplementary material for: Hidden Treasures in “Ancient” Microarrays: Gene-Expression Portrays Biology and Potential Resistance Pathways of Major Lung Cancer Subtypes and Normal Tissue
Source: Front Oncol. 2014 Sep 29;4:251. doi: 10.3389/fonc.2014.00251 (PMC4178426; doi:10.3389/fonc.2014.00251)
Supplement: Figure S1 — Clinically relevant differentially expressed pathways in each tissue type versus the rest. The pathway maps are arranged according to the scheme in the left-top corner. Red indicates overexpression in genes, dark green downregulation. In the Cell Cycle pathway, tumor suppressor genes were overexpressed in normal lung tissue while oncogenes and tumor driving cyclins were overexpressed in cancers. The gene thymidylate synthase (TYMS or TS, belonging to the pathway “one carbon pool by folate”) is relevant for tumor growth and is also a treatment target. Notably, TYMS was overexpressed only in the tumors that are generally refractory to the drug pemetrexed, as the squamous and the small-cell lung cancer. TYMS was not overexpressed in mesothelioma, but it is known that TYMS expression is highly variable in this cancer. In the ERBB pathway, the ERBB2/HER2 and ERBB3/HER3 were overexpressed in adenocarcinoma, while the ERBB4 was overexpressed in the carcinoids. [file Data_Sheet_1.ZIP › File S3.ZIP!/GO Analysis/Small Cell vs Rest Enrichment Analysis/Overrepresented GO terms of Differentially Expressed Genes.html]

Gene to GO BP Conditional test for over-representation

| GOBPID | Pvalue | OddsRatio | ExpCount | Count | Size | Term |
| --- | --- | --- | --- | --- | --- | --- |
| GO:0000278 | <0.0001 | 3.557 | 49 | 128 | 499 | mitotic cell cycle |
| GO:0051301 | <0.0001 | 3.465 | 30 | 79 | 301 | cell division |
| GO:0048285 | <0.0001 | 3.809 | 23 | 66 | 233 | organelle fission |
| GO:0051325 | <0.0001 | 3.176 | 31 | 77 | 312 | interphase |
| GO:0000236 | <0.0001 | 10.141 | 5 | 24 | 46 | mitotic prometaphase |
| GO:0000279 | <0.0001 | 2.827 | 24 | 56 | 250 | M phase |
| GO:0006260 | <0.0001 | 3.248 | 16 | 41 | 160 | DNA replication |
| GO:0000075 | <0.0001 | 4.906 | 7 | 24 | 70 | cell cycle checkpoint |
| GO:0000086 | <0.0001 | 4.070 | 9 | 27 | 89 | G2/M transition of mitotic cell cycle |
| GO:0006281 | <0.0001 | 3.323 | 13 | 34 | 133 | DNA repair |
| GO:0010564 | <0.0001 | 2.352 | 30 | 60 | 302 | regulation of cell cycle process |
| GO:0000083 | <0.0001 | 12.591 | 2 | 11 | 19 | regulation of transcription involved in G1/S phase of mitotic cell cycle |
| GO:0007051 | <0.0001 | 8.631 | 3 | 13 | 27 | spindle organization |
| GO:0006271 | <0.0001 | 10.032 | 2 | 12 | 23 | DNA strand elongation involved in DNA replication |
| GO:0006310 | <0.0001 | 3.552 | 10 | 27 | 98 | DNA recombination |
| GO:0051276 | <0.0001 | 5.317 | 5 | 17 | 48 | chromosome organization |
| GO:0033554 | <0.0001 | 1.734 | 75 | 115 | 763 | cellular response to stress |
| GO:0000070 | <0.0001 | 10.156 | 2 | 11 | 21 | mitotic sister chromatid segregation |
| GO:0007018 | <0.0001 | 3.481 | 8 | 23 | 84 | microtubule-based movement |
| GO:0007017 | <0.0001 | 3.068 | 11 | 27 | 111 | microtubule-based process |
| GO:0006284 | <0.0001 | 6.272 | 3 | 13 | 32 | base-excision repair |
| GO:0000722 | <0.0001 | 7.743 | 2 | 11 | 24 | telomere maintenance via recombination |
| GO:0032201 | <0.0001 | 7.743 | 2 | 11 | 24 | telomere maintenance via semi-conservative replication |
| GO:0006323 | <0.0001 | 3.584 | 7 | 21 | 75 | DNA packaging |
| GO:0006139 | <0.0001 | 1.392 | 301 | 358 | 3026 | nucleobase-containing compound metabolic process |
| GO:0006297 | <0.0001 | 10.275 | 2 | 9 | 17 | nucleotide-excision repair, DNA gap filling |
| GO:0006270 | <0.0001 | 7.189 | 2 | 11 | 25 | DNA replication initiation |
| GO:0016043 | <0.0001 | 1.460 | 144 | 185 | 1514 | cellular component organization |
| GO:0000084 | <0.0001 | 2.597 | 12 | 27 | 123 | S phase of mitotic cell cycle |
| GO:0007062 | <0.0001 | 10.666 | 1 | 7 | 13 | sister chromatid cohesion |
| GO:0031577 | <0.0001 | 5.709 | 3 | 10 | 26 | spindle checkpoint |
| GO:0007059 | <0.0001 | 4.196 | 4 | 13 | 42 | chromosome segregation |
| GO:0000079 | <0.0001 | 3.334 | 6 | 16 | 60 | regulation of cyclin-dependent protein kinase activity |
| GO:0051783 | <0.0001 | 3.183 | 7 | 17 | 66 | regulation of nuclear division |
| GO:0048199 | <0.0001 | 6.631 | 2 | 8 | 19 | vesicle targeting, to, from or within Golgi |
| GO:0048208 | <0.0001 | 10.922 | 1 | 6 | 11 | COPII vesicle coating |
| GO:0007067 | <0.0001 | 2.440 | 11 | 24 | 119 | mitosis |
| GO:0022402 | <0.0001 | 2.039 | 19 | 35 | 213 | cell cycle process |
| GO:0045786 | <0.0001 | 1.808 | 30 | 49 | 303 | negative regulation of cell cycle |
| GO:0019985 | <0.0001 | 36.334 | 0 | 4 | 5 | translesion synthesis |
| GO:0007010 | <0.0001 | 1.570 | 54 | 78 | 544 | cytoskeleton organization |
| GO:0006298 | <0.0001 | 7.084 | 2 | 7 | 16 | mismatch repair |
| GO:0030071 | 0.001 | 5.629 | 2 | 8 | 21 | regulation of mitotic metaphase/anaphase transition |
| GO:0051321 | 0.001 | 2.557 | 9 | 20 | 92 | meiotic cell cycle |
| GO:0007094 | 0.001 | 5.609 | 2 | 8 | 21 | mitotic cell cycle spindle assembly checkpoint |
| GO:0051172 | 0.001 | 1.509 | 65 | 90 | 651 | negative regulation of nitrogen compound metabolic process |
| GO:0006283 | 0.001 | 3.538 | 4 | 12 | 43 | transcription-coupled nucleotide-excision repair |
| GO:0032465 | 0.001 | 6.375 | 2 | 7 | 17 | regulation of cytokinesis |
| GO:0032200 | 0.001 | 3.302 | 5 | 13 | 49 | telomere organization |
| GO:0006807 | 0.001 | 1.295 | 271 | 310 | 2817 | nitrogen compound metabolic process |
| GO:0007076 | 0.001 | 11.364 | 1 | 5 | 9 | mitotic chromosome condensation |
| GO:0044260 | 0.001 | 1.294 | 294 | 333 | 3227 | cellular macromolecule metabolic process |
| GO:0051983 | 0.001 | Inf | 0 | 3 | 3 | regulation of chromosome segregation |
| GO:0030951 | 0.001 | Inf | 0 | 3 | 3 | establishment or maintenance of microtubule cytoskeleton polarity |
| GO:0009987 | 0.001 | 1.655 | 722 | 743 | 7246 | cellular process |
| GO:0031324 | 0.001 | 1.395 | 95 | 122 | 949 | negative regulation of cellular metabolic process |
| GO:0016446 | 0.001 | 18.165 | 1 | 4 | 6 | somatic hypermutation of immunoglobulin genes |
| GO:0071704 | 0.001 | 1.283 | 516 | 554 | 5180 | organic substance metabolic process |
| GO:0045132 | 0.002 | 9.090 | 1 | 5 | 10 | meiotic chromosome segregation |
| GO:0051322 | 0.002 | 9.090 | 1 | 5 | 10 | anaphase |
| GO:0006334 | 0.002 | 3.132 | 5 | 12 | 47 | nucleosome assembly |
| GO:0071824 | 0.002 | 2.390 | 9 | 18 | 87 | protein-DNA complex subunit organization |
| GO:0045665 | 0.002 | 3.508 | 4 | 10 | 36 | negative regulation of neuron differentiation |
| GO:0009059 | 0.002 | 1.603 | 36 | 53 | 377 | macromolecule biosynthetic process |
| GO:0034622 | 0.002 | 1.607 | 34 | 51 | 345 | cellular macromolecular complex assembly |
| GO:0009263 | 0.003 | 12.108 | 1 | 4 | 7 | deoxyribonucleotide biosynthetic process |
| GO:0048715 | 0.003 | 12.108 | 1 | 4 | 7 | negative regulation of oligodendrocyte differentiation |
| GO:0051488 | 0.003 | 12.108 | 1 | 4 | 7 | activation of anaphase-promoting complex activity |
| GO:0051303 | 0.003 | 7.574 | 1 | 5 | 11 | establishment of chromosome localization |
| GO:0051640 | 0.003 | 2.140 | 11 | 21 | 111 | organelle localization |
| GO:0014014 | 0.003 | 4.551 | 2 | 7 | 21 | negative regulation of gliogenesis |
| GO:0010629 | 0.003 | 1.431 | 61 | 81 | 609 | negative regulation of gene expression |
| GO:0003183 | 0.004 | 27.216 | 0 | 3 | 4 | mitral valve morphogenesis |
| GO:0003289 | 0.004 | 27.216 | 0 | 3 | 4 | atrial septum primum morphogenesis |
| GO:0009396 | 0.004 | 27.216 | 0 | 3 | 4 | folic acid-containing compound biosynthetic process |
| GO:0019614 | 0.004 | 27.216 | 0 | 3 | 4 | catechol-containing compound catabolic process |
| GO:0034501 | 0.004 | 27.216 | 0 | 3 | 4 | protein localization to kinetochore |
| GO:0042420 | 0.004 | 27.216 | 0 | 3 | 4 | dopamine catabolic process |
| GO:0045839 | 0.004 | 3.835 | 3 | 8 | 27 | negative regulation of mitosis |
| GO:0006302 | 0.004 | 3.170 | 4 | 10 | 39 | double-strand break repair |
| GO:0042136 | 0.004 | 6.491 | 1 | 5 | 12 | neurotransmitter biosynthetic process |
| GO:0042149 | 0.004 | 6.491 | 1 | 5 | 12 | cellular response to glucose starvation |
| GO:0034976 | 0.004 | 2.254 | 9 | 17 | 86 | response to endoplasmic reticulum stress |
| GO:0019886 | 0.005 | 2.460 | 7 | 14 | 66 | antigen processing and presentation of exogenous peptide antigen via MHC class II |
| GO:0000082 | 0.005 | 1.923 | 14 | 24 | 140 | G1/S transition of mitotic cell cycle |
| GO:0000724 | 0.005 | 3.280 | 3 | 9 | 34 | double-strand break repair via homologous recombination |
| GO:0045685 | 0.005 | 3.280 | 3 | 9 | 34 | regulation of glial cell differentiation |
| GO:0051383 | 0.005 | 9.080 | 1 | 4 | 8 | kinetochore organization |
| GO:0006987 | 0.005 | 2.671 | 5 | 12 | 53 | activation of signaling protein activity involved in unfolded protein response |
| GO:2000113 | 0.005 | 1.411 | 60 | 79 | 603 | negative regulation of cellular macromolecule biosynthetic process |
| GO:0007126 | 0.005 | 2.264 | 8 | 16 | 81 | meiosis |
| GO:0022607 | 0.005 | 1.341 | 87 | 109 | 879 | cellular component assembly |
| GO:0002504 | 0.005 | 2.413 | 7 | 14 | 67 | antigen processing and presentation of peptide or polysaccharide antigen via MHC class II |
| GO:0007093 | 0.006 | 2.035 | 11 | 20 | 110 | mitotic cell cycle checkpoint |
| GO:0010389 | 0.006 | 3.981 | 2 | 7 | 23 | regulation of G2/M transition of mitotic cell cycle |
| GO:0051253 | 0.006 | 1.415 | 57 | 75 | 570 | negative regulation of RNA metabolic process |
| GO:0015949 | 0.006 | 5.679 | 1 | 5 | 13 | nucleobase-containing small molecule interconversion |
| GO:0045787 | 0.007 | 2.435 | 6 | 13 | 62 | positive regulation of cell cycle |
| GO:0006403 | 0.007 | 2.129 | 9 | 17 | 90 | RNA localization |
| GO:0006921 | 0.007 | 2.422 | 6 | 13 | 62 | cellular component disassembly involved in execution phase of apoptosis |
| GO:0050658 | 0.008 | 7.320 | 1 | 4 | 9 | RNA transport |
| GO:0007098 | 0.008 | 7.316 | 1 | 4 | 9 | centrosome cycle |
| GO:0006396 | 0.008 | 1.488 | 37 | 52 | 375 | RNA processing |
| GO:0033599 | 0.008 | 7.281 | 1 | 4 | 9 | regulation of mammary gland epithelial cell proliferation |
| GO:0033240 | 0.008 | 7.263 | 1 | 4 | 9 | positive regulation of cellular amine metabolic process |
| GO:0046653 | 0.008 | 7.263 | 1 | 4 | 9 | tetrahydrofolate metabolic process |
| GO:0007091 | 0.008 | 13.771 | 0 | 3 | 5 | mitotic metaphase/anaphase transition |
| GO:0031572 | 0.008 | 4.196 | 2 | 6 | 19 | G2/M transition DNA damage checkpoint |
| GO:0008608 | 0.008 | 13.655 | 0 | 3 | 5 | attachment of spindle microtubules to kinetochore |
| GO:0051988 | 0.008 | 13.606 | 0 | 3 | 5 | regulation of attachment of spindle microtubules to kinetochore |
| GO:1900027 | 0.008 | 13.606 | 0 | 3 | 5 | regulation of ruffle assembly |
| GO:0009890 | 0.008 | 1.352 | 70 | 89 | 702 | negative regulation of biosynthetic process |
| GO:0051650 | 0.009 | 2.927 | 4 | 9 | 37 | establishment of vesicle localization |
| GO:0043044 | 0.009 | 5.047 | 1 | 5 | 14 | ATP-dependent chromatin remodeling |
| GO:0080090 | 0.010 | 1.205 | 263 | 293 | 2640 | regulation of primary metabolic process |
| GO:0032075 | 0.010 | Inf | 0 | 2 | 2 | positive regulation of nuclease activity |
| GO:0006097 | 0.010 | Inf | 0 | 2 | 2 | glyoxylate cycle |
| GO:0007079 | 0.010 | Inf | 0 | 2 | 2 | mitotic chromosome movement towards spindle pole |
| GO:0009186 | 0.010 | Inf | 0 | 2 | 2 | deoxyribonucleoside diphosphate metabolic process |
| GO:0010032 | 0.010 | Inf | 0 | 2 | 2 | meiotic chromosome condensation |
| GO:0010994 | 0.010 | Inf | 0 | 2 | 2 | free ubiquitin chain polymerization |
| GO:0030997 | 0.010 | Inf | 0 | 2 | 2 | regulation of centriole-centriole cohesion |
| GO:0033601 | 0.010 | Inf | 0 | 2 | 2 | positive regulation of mammary gland epithelial cell proliferation |
| GO:0036089 | 0.010 | Inf | 0 | 2 | 2 | cleavage furrow formation |
| GO:0048478 | 0.010 | Inf | 0 | 2 | 2 | replication fork protection |
| GO:0051984 | 0.010 | Inf | 0 | 2 | 2 | positive regulation of chromosome segregation |
| GO:0090007 | 0.010 | Inf | 0 | 2 | 2 | regulation of mitotic anaphase |
| GO:0010639 | 0.010 | 1.866 | 12 | 21 | 124 | negative regulation of organelle organization |
| GO:0006900 | 0.011 | 3.034 | 3 | 8 | 32 | membrane budding |
| GO:0090307 | 0.012 | 6.066 | 1 | 4 | 10 | spindle assembly involved in mitosis |
| GO:0006461 | 0.013 | 1.374 | 54 | 70 | 542 | protein complex assembly |
| GO:0006266 | 0.013 | 6.051 | 1 | 4 | 10 | DNA ligation |
| GO:0050686 | 0.013 | 6.051 | 1 | 4 | 10 | negative regulation of mRNA processing |
| GO:0070935 | 0.013 | 6.051 | 1 | 4 | 10 | 3'-UTR-mediated mRNA stabilization |
| GO:0071777 | 0.013 | 6.051 | 1 | 4 | 10 | positive regulation of cell cycle cytokinesis |
| GO:0006687 | 0.013 | 2.731 | 4 | 9 | 39 | glycosphingolipid metabolic process |
| GO:0051289 | 0.013 | 2.731 | 4 | 9 | 39 | protein homotetramerization |
| GO:0051052 | 0.013 | 1.770 | 14 | 23 | 142 | regulation of DNA metabolic process |
| GO:0034620 | 0.014 | 2.196 | 7 | 13 | 67 | cellular response to unfolded protein |
| GO:0043488 | 0.014 | 3.183 | 3 | 7 | 27 | regulation of mRNA stability |
| GO:0070727 | 0.015 | 1.328 | 66 | 83 | 663 | cellular macromolecule localization |
| GO:0042552 | 0.015 | 2.094 | 7 | 14 | 75 | myelination |
| GO:0000122 | 0.015 | 1.443 | 36 | 49 | 362 | negative regulation of transcription from RNA polymerase II promoter |
| GO:0000731 | 0.016 | 9.069 | 1 | 3 | 6 | DNA synthesis involved in DNA repair |
| GO:0045842 | 0.016 | 9.069 | 1 | 3 | 6 | positive regulation of mitotic metaphase/anaphase transition |
| GO:0042982 | 0.017 | 4.128 | 2 | 5 | 16 | amyloid precursor protein metabolic process |
| GO:0048665 | 0.017 | 4.128 | 2 | 5 | 16 | neuron fate specification |
| GO:0006984 | 0.017 | 2.060 | 8 | 14 | 76 | ER-nucleus signaling pathway |
| GO:0007272 | 0.017 | 2.060 | 8 | 14 | 76 | ensheathment of neurons |
| GO:0032268 | 0.017 | 1.273 | 92 | 111 | 924 | regulation of cellular protein metabolic process |
| GO:0000080 | 0.017 | 2.560 | 4 | 9 | 41 | G1 phase of mitotic cell cycle |
| GO:0000375 | 0.018 | 1.690 | 15 | 24 | 154 | RNA splicing, via transesterification reactions |
| GO:0051258 | 0.018 | 1.852 | 11 | 18 | 107 | protein polymerization |
| GO:0000578 | 0.018 | 3.408 | 2 | 6 | 22 | embryonic axis specification |
| GO:2001020 | 0.018 | 2.187 | 6 | 12 | 62 | regulation of response to DNA damage stimulus |
| GO:0050767 | 0.018 | 1.478 | 29 | 41 | 296 | regulation of neurogenesis |
| GO:0033598 | 0.018 | 5.223 | 1 | 4 | 11 | mammary gland epithelial cell proliferation |
| GO:0030879 | 0.018 | 1.847 | 11 | 18 | 107 | mammary gland development |
| GO:0000085 | 0.018 | 5.186 | 1 | 4 | 11 | G2 phase of mitotic cell cycle |
| GO:0003283 | 0.018 | 5.186 | 1 | 4 | 11 | atrial septum development |
| GO:0021781 | 0.018 | 5.186 | 1 | 4 | 11 | glial cell fate commitment |
| GO:0055015 | 0.018 | 5.186 | 1 | 4 | 11 | ventricular cardiac muscle cell development |
| GO:0015931 | 0.019 | 1.870 | 10 | 17 | 100 | nucleobase-containing compound transport |
| GO:0051084 | 0.019 | 2.695 | 3 | 8 | 35 | 'de novo' posttranslational protein folding |
| GO:0031145 | 0.019 | 2.079 | 7 | 13 | 70 | anaphase-promoting complex-dependent proteasomal ubiquitin-dependent protein catabolic process |
| GO:0032507 | 0.019 | 2.079 | 7 | 13 | 70 | maintenance of protein location in cell |
| GO:0006355 | 0.020 | 1.208 | 160 | 183 | 1614 | regulation of transcription, DNA-dependent |
| GO:0016447 | 0.021 | 2.893 | 3 | 7 | 29 | somatic recombination of immunoglobulin gene segments |
| GO:0070647 | 0.021 | 1.410 | 37 | 49 | 369 | protein modification by small protein conjugation or removal |
| GO:0019692 | 0.022 | 3.784 | 2 | 5 | 17 | deoxyribose phosphate metabolic process |
| GO:0042558 | 0.022 | 3.784 | 2 | 5 | 17 | pteridine-containing compound metabolic process |
| GO:0045739 | 0.022 | 3.784 | 2 | 5 | 17 | positive regulation of DNA repair |
| GO:0051298 | 0.022 | 3.784 | 2 | 5 | 17 | centrosome duplication |
| GO:0000398 | 0.022 | 1.670 | 15 | 23 | 149 | mRNA splicing, via spliceosome |
| GO:0006888 | 0.023 | 2.599 | 4 | 8 | 36 | ER to Golgi vesicle-mediated transport |
| GO:0016444 | 0.023 | 2.599 | 4 | 8 | 36 | somatic cell DNA recombination |
| GO:0032886 | 0.023 | 2.178 | 6 | 11 | 57 | regulation of microtubule-based process |
| GO:0030705 | 0.023 | 2.409 | 4 | 9 | 43 | cytoskeleton-dependent intracellular transport |
| GO:0030900 | 0.024 | 1.544 | 21 | 30 | 208 | forebrain development |
| GO:0001101 | 0.024 | 2.008 | 7 | 13 | 72 | response to acid |
| GO:0010468 | 0.025 | 1.472 | 27 | 37 | 279 | regulation of gene expression |
| GO:0006325 | 0.025 | 2.376 | 4 | 9 | 44 | chromatin organization |
| GO:0006376 | 0.025 | 4.537 | 1 | 4 | 12 | mRNA splice site selection |
| GO:0051569 | 0.025 | 4.537 | 1 | 4 | 12 | regulation of histone H3-K4 methylation |
| GO:0071697 | 0.025 | 4.537 | 1 | 4 | 12 | ectodermal placode morphogenesis |
| GO:0000320 | 0.025 | 6.801 | 1 | 3 | 7 | re-entry into mitotic cell cycle |
| GO:0006268 | 0.025 | 6.801 | 1 | 3 | 7 | DNA unwinding involved in replication |
| GO:0006273 | 0.025 | 6.801 | 1 | 3 | 7 | lagging strand elongation |
| GO:0006538 | 0.025 | 6.801 | 1 | 3 | 7 | glutamate catabolic process |
| GO:0007063 | 0.025 | 6.801 | 1 | 3 | 7 | regulation of sister chromatid cohesion |
| GO:0031396 | 0.026 | 1.683 | 13 | 21 | 135 | regulation of protein ubiquitination |
| GO:0061351 | 0.026 | 3.050 | 2 | 6 | 24 | neural precursor cell proliferation |
| GO:0031099 | 0.026 | 1.874 | 9 | 15 | 88 | regeneration |
| GO:0002200 | 0.027 | 2.509 | 4 | 8 | 37 | somatic diversification of immune receptors |
| GO:0032088 | 0.027 | 2.509 | 4 | 8 | 37 | negative regulation of NF-kappaB transcription factor activity |
| GO:0010556 | 0.027 | 1.188 | 181 | 203 | 1822 | regulation of macromolecule biosynthetic process |
| GO:0021543 | 0.027 | 1.975 | 7 | 13 | 73 | pallium development |
| GO:0051489 | 0.027 | 3.028 | 2 | 6 | 24 | regulation of filopodium assembly |
| GO:0006259 | 0.027 | 1.964 | 7 | 13 | 79 | DNA metabolic process |
| GO:0000212 | 0.028 | 18.121 | 0 | 2 | 3 | meiotic spindle organization |
| GO:0006065 | 0.028 | 18.121 | 0 | 2 | 3 | UDP-glucuronate biosynthetic process |
| GO:0006177 | 0.028 | 18.121 | 0 | 2 | 3 | GMP biosynthetic process |
| GO:0006235 | 0.028 | 18.121 | 0 | 2 | 3 | dTTP biosynthetic process |
| GO:0008054 | 0.028 | 18.121 | 0 | 2 | 3 | cyclin catabolic process |
| GO:0010956 | 0.028 | 18.121 | 0 | 2 | 3 | negative regulation of calcidiol 1-monooxygenase activity |
| GO:0010957 | 0.028 | 18.121 | 0 | 2 | 3 | negative regulation of vitamin D biosynthetic process |
| GO:0014722 | 0.028 | 18.121 | 0 | 2 | 3 | regulation of skeletal muscle contraction by calcium ion signaling |
| GO:0016199 | 0.028 | 18.121 | 0 | 2 | 3 | axon midline choice point recognition |
| GO:0021530 | 0.028 | 18.121 | 0 | 2 | 3 | spinal cord oligodendrocyte cell fate specification |
| GO:0030910 | 0.028 | 18.121 | 0 | 2 | 3 | olfactory placode formation |
| GO:0031536 | 0.028 | 18.121 | 0 | 2 | 3 | positive regulation of exit from mitosis |
| GO:0034723 | 0.028 | 18.121 | 0 | 2 | 3 | DNA replication-dependent nucleosome organization |
| GO:0043153 | 0.028 | 18.121 | 0 | 2 | 3 | entrainment of circadian clock by photoperiod |
| GO:0045007 | 0.028 | 18.121 | 0 | 2 | 3 | depurination |
| GO:0051176 | 0.028 | 18.121 | 0 | 2 | 3 | positive regulation of sulfur metabolic process |
| GO:0051256 | 0.028 | 18.121 | 0 | 2 | 3 | spindle midzone assembly involved in mitosis |
| GO:0060166 | 0.028 | 18.121 | 0 | 2 | 3 | olfactory pit development |
| GO:0060236 | 0.028 | 18.121 | 0 | 2 | 3 | regulation of mitotic spindle organization |
| GO:0060440 | 0.028 | 18.121 | 0 | 2 | 3 | trachea formation |
| GO:0061198 | 0.028 | 18.121 | 0 | 2 | 3 | fungiform papilla formation |
| GO:0071698 | 0.028 | 18.121 | 0 | 2 | 3 | olfactory placode development |
| GO:2000017 | 0.028 | 18.121 | 0 | 2 | 3 | positive regulation of determination of dorsal identity |
| GO:2001240 | 0.028 | 18.121 | 0 | 2 | 3 | negative regulation of extrinsic apoptotic signaling pathway in absence of ligand |
| GO:0007420 | 0.029 | 1.615 | 15 | 23 | 155 | brain development |
| GO:0016568 | 0.031 | 1.435 | 28 | 38 | 281 | chromatin modification |
| GO:0018130 | 0.032 | 1.171 | 211 | 234 | 2123 | heterocycle biosynthetic process |
| GO:0019438 | 0.032 | 1.171 | 211 | 234 | 2123 | aromatic compound biosynthetic process |
| GO:0030031 | 0.032 | 1.660 | 13 | 20 | 130 | cell projection assembly |
| GO:0032774 | 0.032 | 1.177 | 185 | 207 | 1862 | RNA biosynthetic process |
| GO:0051297 | 0.032 | 4.081 | 1 | 4 | 13 | centrosome organization |
| GO:0016925 | 0.033 | 2.869 | 2 | 6 | 25 | protein sumoylation |
| GO:0043933 | 0.033 | 1.256 | 77 | 92 | 780 | macromolecular complex subunit organization |
| GO:0042063 | 0.033 | 1.735 | 11 | 17 | 107 | gliogenesis |
| GO:0006471 | 0.034 | 4.033 | 1 | 4 | 13 | protein ADP-ribosylation |
| GO:0021522 | 0.034 | 4.033 | 1 | 4 | 13 | spinal cord motor neuron differentiation |
| GO:0003209 | 0.035 | 3.242 | 2 | 5 | 19 | cardiac atrium morphogenesis |
| GO:0006672 | 0.036 | 2.346 | 4 | 8 | 39 | ceramide metabolic process |
| GO:0031647 | 0.036 | 1.880 | 8 | 13 | 76 | regulation of protein stability |
| GO:0051028 | 0.036 | 1.880 | 8 | 13 | 76 | mRNA transport |
| GO:0007422 | 0.037 | 2.003 | 6 | 11 | 61 | peripheral nervous system development |
| GO:0044271 | 0.037 | 1.163 | 216 | 238 | 2170 | cellular nitrogen compound biosynthetic process |
| GO:0016570 | 0.037 | 1.524 | 18 | 26 | 182 | histone modification |
| GO:0048754 | 0.037 | 1.651 | 12 | 19 | 124 | branching morphogenesis of an epithelial tube |
| GO:0001967 | 0.038 | 5.440 | 1 | 3 | 8 | suckling behavior |
| GO:0006356 | 0.038 | 5.440 | 1 | 3 | 8 | regulation of transcription from RNA polymerase I promoter |
| GO:0009219 | 0.038 | 5.440 | 1 | 3 | 8 | pyrimidine deoxyribonucleotide metabolic process |
| GO:0009650 | 0.038 | 5.440 | 1 | 3 | 8 | UV protection |
| GO:0033160 | 0.038 | 5.440 | 1 | 3 | 8 | positive regulation of protein import into nucleus, translocation |
| GO:0034080 | 0.038 | 5.440 | 1 | 3 | 8 | CENP-A containing nucleosome assembly at centromere |
| GO:0034724 | 0.038 | 5.440 | 1 | 3 | 8 | DNA replication-independent nucleosome organization |
| GO:0043569 | 0.038 | 5.440 | 1 | 3 | 8 | negative regulation of insulin-like growth factor receptor signaling pathway |
| GO:0045910 | 0.038 | 5.440 | 1 | 3 | 8 | negative regulation of DNA recombination |
| GO:0050884 | 0.038 | 5.440 | 1 | 3 | 8 | neuromuscular process controlling posture |
| GO:0051593 | 0.038 | 5.440 | 1 | 3 | 8 | response to folic acid |
| GO:0060911 | 0.038 | 5.440 | 1 | 3 | 8 | cardiac cell fate commitment |
| GO:0046661 | 0.038 | 1.737 | 10 | 16 | 100 | male sex differentiation |
| GO:0032885 | 0.039 | 2.725 | 3 | 6 | 26 | regulation of polysaccharide biosynthetic process |
| GO:0009064 | 0.040 | 2.154 | 5 | 9 | 47 | glutamine family amino acid metabolic process |
| GO:0007405 | 0.041 | 2.446 | 3 | 7 | 33 | neuroblast proliferation |
| GO:0045931 | 0.041 | 2.446 | 3 | 7 | 33 | positive regulation of mitotic cell cycle |
| GO:0000216 | 0.042 | 1.884 | 7 | 12 | 70 | M/G1 transition of mitotic cell cycle |
| GO:0043489 | 0.042 | 3.026 | 2 | 5 | 20 | RNA stabilization |
| GO:0051225 | 0.043 | 3.653 | 1 | 4 | 14 | spindle assembly |
| GO:0008104 | 0.044 | 1.207 | 102 | 118 | 1025 | protein localization |
| GO:0009112 | 0.045 | 2.098 | 5 | 9 | 48 | nucleobase metabolic process |
| GO:0007131 | 0.046 | 2.595 | 3 | 6 | 27 | reciprocal meiotic recombination |
| GO:0008156 | 0.046 | 2.595 | 3 | 6 | 27 | negative regulation of DNA replication |
| GO:0032091 | 0.046 | 2.595 | 3 | 6 | 27 | negative regulation of protein binding |
| GO:0043392 | 0.046 | 2.595 | 3 | 6 | 27 | negative regulation of DNA binding |
| GO:0045682 | 0.046 | 2.595 | 3 | 6 | 27 | regulation of epidermis development |
| GO:2000179 | 0.046 | 2.595 | 3 | 6 | 27 | positive regulation of neural precursor cell proliferation |
| GO:0005978 | 0.047 | 2.355 | 3 | 7 | 34 | glycogen biosynthetic process |
| GO:1901607 | 0.047 | 2.355 | 3 | 7 | 34 | alpha-amino acid biosynthetic process |
| GO:0048519 | 0.048 | 1.152 | 218 | 238 | 2184 | negative regulation of biological process |
| GO:0006308 | 0.048 | 1.977 | 6 | 10 | 56 | DNA catabolic process |
| GO:0060255 | 0.050 | 1.310 | 42 | 53 | 454 | regulation of macromolecule metabolic process |
