# Supplementary material for: Hidden Treasures in “Ancient” Microarrays: Gene-Expression Portrays Biology and Potential Resistance Pathways of Major Lung Cancer Subtypes and Normal Tissue
Source: Front Oncol. 2014 Sep 29;4:251. doi: 10.3389/fonc.2014.00251 (PMC4178426; doi:10.3389/fonc.2014.00251)
Supplement: Figure S1 — Clinically relevant differentially expressed pathways in each tissue type versus the rest. The pathway maps are arranged according to the scheme in the left-top corner. Red indicates overexpression in genes, dark green downregulation. In the Cell Cycle pathway, tumor suppressor genes were overexpressed in normal lung tissue while oncogenes and tumor driving cyclins were overexpressed in cancers. The gene thymidylate synthase (TYMS or TS, belonging to the pathway “one carbon pool by folate”) is relevant for tumor growth and is also a treatment target. Notably, TYMS was overexpressed only in the tumors that are generally refractory to the drug pemetrexed, as the squamous and the small-cell lung cancer. TYMS was not overexpressed in mesothelioma, but it is known that TYMS expression is highly variable in this cancer. In the ERBB pathway, the ERBB2/HER2 and ERBB3/HER3 were overexpressed in adenocarcinoma, while the ERBB4 was overexpressed in the carcinoids. [file Data_Sheet_1.ZIP › File S3.ZIP!/GO Analysis/Small Cell vs Rest Enrichment Analysis/Overrepresented GO terms of Down-regulated Genes.html]

Gene to GO BP Conditional test for over-representation

| GOBPID | Pvalue | OddsRatio | ExpCount | Count | Size | Term |
| --- | --- | --- | --- | --- | --- | --- |
| GO:0048199 | <0.0001 | 16.851 | 1 | 8 | 19 | vesicle targeting, to, from or within Golgi |
| GO:0048208 | <0.0001 | 27.655 | 0 | 6 | 11 | COPII vesicle coating |
| GO:0006888 | <0.0001 | 6.605 | 2 | 8 | 36 | ER to Golgi vesicle-mediated transport |
| GO:0051650 | <0.0001 | 6.376 | 2 | 8 | 37 | establishment of vesicle localization |
| GO:0018279 | <0.0001 | 4.547 | 3 | 10 | 61 | protein N-linked glycosylation via asparagine |
| GO:0016192 | <0.0001 | 1.930 | 24 | 42 | 579 | vesicle-mediated transport |
| GO:0034976 | <0.0001 | 3.772 | 4 | 12 | 86 | response to endoplasmic reticulum stress |
| GO:0051289 | 0.001 | 5.039 | 2 | 7 | 39 | protein homotetramerization |
| GO:0044712 | 0.001 | 2.448 | 8 | 18 | 190 | single-organism catabolic process |
| GO:0061024 | 0.001 | 2.141 | 12 | 24 | 288 | membrane organization |
| GO:0009100 | 0.001 | 2.590 | 7 | 16 | 162 | glycoprotein metabolic process |
| GO:0006984 | 0.001 | 3.506 | 3 | 10 | 76 | ER-nucleus signaling pathway |
| GO:0006027 | 0.001 | 4.741 | 2 | 7 | 41 | glycosaminoglycan catabolic process |
| GO:0035966 | 0.002 | 2.994 | 4 | 12 | 105 | response to topologically incorrect protein |
| GO:0006987 | 0.002 | 4.100 | 2 | 8 | 53 | activation of signaling protein activity involved in unfolded protein response |
| GO:0005975 | 0.002 | 1.812 | 21 | 35 | 494 | carbohydrate metabolic process |
| GO:0072329 | 0.002 | 3.647 | 3 | 9 | 66 | monocarboxylic acid catabolic process |
| GO:0044242 | 0.002 | 2.930 | 5 | 12 | 107 | cellular lipid catabolic process |
| GO:0006900 | 0.002 | 6.803 | 1 | 5 | 22 | membrane budding |
| GO:0034620 | 0.002 | 3.584 | 3 | 9 | 67 | cellular response to unfolded protein |
| GO:0033598 | 0.002 | 6.747 | 1 | 5 | 22 | mammary gland epithelial cell proliferation |
| GO:0051641 | 0.003 | 2.804 | 5 | 12 | 120 | cellular localization |
| GO:0034440 | 0.003 | 3.406 | 3 | 9 | 70 | lipid oxidation |
| GO:0032069 | 0.003 | 3.688 | 2 | 8 | 58 | regulation of nuclease activity |
| GO:0006635 | 0.003 | 4.130 | 2 | 7 | 46 | fatty acid beta-oxidation |
| GO:0016054 | 0.003 | 2.463 | 6 | 14 | 146 | organic acid catabolic process |
| GO:0006886 | 0.004 | 2.409 | 6 | 14 | 153 | intracellular protein transport |
| GO:0006687 | 0.005 | 4.319 | 2 | 6 | 38 | glycosphingolipid metabolic process |
| GO:0002576 | 0.005 | 3.053 | 3 | 9 | 77 | platelet degranulation |
| GO:0006065 | 0.005 | 45.558 | 0 | 2 | 3 | UDP-glucuronate biosynthetic process |
| GO:0006177 | 0.005 | 45.558 | 0 | 2 | 3 | GMP biosynthetic process |
| GO:0051176 | 0.005 | 45.558 | 0 | 2 | 3 | positive regulation of sulfur metabolic process |
| GO:0060440 | 0.005 | 45.558 | 0 | 2 | 3 | trachea formation |
| GO:2000017 | 0.005 | 45.558 | 0 | 2 | 3 | positive regulation of determination of dorsal identity |
| GO:0006684 | 0.005 | 11.416 | 0 | 3 | 9 | sphingomyelin metabolic process |
| GO:0007016 | 0.005 | 11.416 | 0 | 3 | 9 | cytoskeletal anchoring at plasma membrane |
| GO:0033197 | 0.005 | 11.416 | 0 | 3 | 9 | response to vitamin E |
| GO:0042987 | 0.005 | 11.416 | 0 | 3 | 9 | amyloid precursor protein catabolic process |
| GO:0048205 | 0.005 | 11.416 | 0 | 3 | 9 | COPI coating of Golgi vesicle |
| GO:0048754 | 0.006 | 2.480 | 5 | 12 | 124 | branching morphogenesis of an epithelial tube |
| GO:0043687 | 0.006 | 2.595 | 5 | 11 | 109 | post-translational protein modification |
| GO:0048194 | 0.007 | 9.784 | 0 | 3 | 10 | Golgi vesicle budding |
| GO:0007040 | 0.007 | 6.100 | 1 | 4 | 19 | lysosome organization |
| GO:0006629 | 0.007 | 1.542 | 31 | 45 | 739 | lipid metabolic process |
| GO:0044281 | 0.008 | 1.378 | 72 | 91 | 1708 | small molecule metabolic process |
| GO:0006022 | 0.008 | 2.653 | 4 | 10 | 97 | aminoglycan metabolic process |
| GO:0006144 | 0.008 | 4.583 | 1 | 5 | 30 | purine nucleobase metabolic process |
| GO:1901137 | 0.008 | 1.704 | 18 | 29 | 430 | carbohydrate derivative biosynthetic process |
| GO:0006364 | 0.008 | 3.352 | 2 | 7 | 55 | rRNA processing |
| GO:0006486 | 0.009 | 2.263 | 6 | 13 | 146 | protein glycosylation |
| GO:0070085 | 0.009 | 2.246 | 6 | 13 | 147 | glycosylation |
| GO:0031581 | 0.010 | 8.560 | 0 | 3 | 11 | hemidesmosome assembly |
| GO:0050435 | 0.010 | 8.560 | 0 | 3 | 11 | beta-amyloid metabolic process |
| GO:0003183 | 0.010 | 22.776 | 0 | 2 | 4 | mitral valve morphogenesis |
| GO:0006011 | 0.010 | 22.776 | 0 | 2 | 4 | UDP-glucose metabolic process |
| GO:0019614 | 0.010 | 22.776 | 0 | 2 | 4 | catechol-containing compound catabolic process |
| GO:0032506 | 0.010 | 22.776 | 0 | 2 | 4 | cytokinetic process |
| GO:0042420 | 0.010 | 22.776 | 0 | 2 | 4 | dopamine catabolic process |
| GO:0044539 | 0.010 | 22.776 | 0 | 2 | 4 | long-chain fatty acid import |
| GO:0008104 | 0.012 | 1.760 | 14 | 23 | 357 | protein localization |
| GO:0007169 | 0.012 | 1.607 | 21 | 32 | 500 | transmembrane receptor protein tyrosine kinase signaling pathway |
| GO:0051649 | 0.012 | 1.403 | 51 | 66 | 1221 | establishment of localization in cell |
| GO:0034613 | 0.012 | 2.160 | 6 | 13 | 161 | cellular protein localization |
| GO:0042136 | 0.012 | 7.608 | 1 | 3 | 12 | neurotransmitter biosynthetic process |
| GO:0042149 | 0.012 | 7.608 | 1 | 3 | 12 | cellular response to glucose starvation |
| GO:0001763 | 0.014 | 2.117 | 7 | 13 | 155 | morphogenesis of a branching structure |
| GO:0051246 | 0.014 | 1.410 | 45 | 59 | 1058 | regulation of protein metabolic process |
| GO:0051234 | 0.014 | 1.465 | 36 | 49 | 923 | establishment of localization |
| GO:1901565 | 0.015 | 1.578 | 21 | 32 | 508 | organonitrogen compound catabolic process |
| GO:0010876 | 0.015 | 2.102 | 7 | 13 | 156 | lipid localization |
| GO:0006892 | 0.015 | 3.276 | 2 | 6 | 48 | post-Golgi vesicle-mediated transport |
| GO:0006921 | 0.015 | 2.923 | 3 | 7 | 62 | cellular component disassembly involved in execution phase of apoptosis |
| GO:0006307 | 0.016 | 15.182 | 0 | 2 | 5 | DNA dealkylation involved in DNA repair |
| GO:0006678 | 0.016 | 15.182 | 0 | 2 | 5 | glucosylceramide metabolic process |
| GO:0009812 | 0.016 | 15.182 | 0 | 2 | 5 | flavonoid metabolic process |
| GO:0019388 | 0.016 | 15.182 | 0 | 2 | 5 | galactose catabolic process |
| GO:0030952 | 0.016 | 15.182 | 0 | 2 | 5 | establishment or maintenance of cytoskeleton polarity |
| GO:0035965 | 0.016 | 15.182 | 0 | 2 | 5 | cardiolipin acyl-chain remodeling |
| GO:0071681 | 0.016 | 15.182 | 0 | 2 | 5 | cellular response to indole-3-methanol |
| GO:1900027 | 0.016 | 15.182 | 0 | 2 | 5 | regulation of ruffle assembly |
| GO:0002755 | 0.018 | 2.819 | 3 | 7 | 64 | MyD88-dependent toll-like receptor signaling pathway |
| GO:0051640 | 0.019 | 2.281 | 5 | 10 | 111 | organelle localization |
| GO:0031667 | 0.019 | 1.872 | 9 | 16 | 214 | response to nutrient levels |
| GO:0008210 | 0.019 | 6.223 | 1 | 3 | 14 | estrogen metabolic process |
| GO:0007034 | 0.020 | 4.354 | 1 | 4 | 25 | vacuolar transport |
| GO:0006029 | 0.020 | 3.056 | 2 | 6 | 51 | proteoglycan metabolic process |
| GO:0043123 | 0.020 | 2.380 | 4 | 9 | 96 | positive regulation of I-kappaB kinase/NF-kappaB cascade |
| GO:0006605 | 0.020 | 1.663 | 15 | 23 | 345 | protein targeting |
| GO:0009060 | 0.021 | 3.468 | 2 | 5 | 38 | aerobic respiration |
| GO:0015031 | 0.021 | 1.722 | 12 | 20 | 306 | protein transport |
| GO:0042119 | 0.023 | 5.704 | 1 | 3 | 15 | neutrophil activation |
| GO:0001942 | 0.024 | 2.926 | 2 | 6 | 53 | hair follicle development |
| GO:0022404 | 0.024 | 2.926 | 2 | 6 | 53 | molting cycle process |
| GO:0006412 | 0.024 | 1.722 | 12 | 19 | 275 | translation |
| GO:0016239 | 0.024 | 11.385 | 0 | 2 | 6 | positive regulation of macroautophagy |
| GO:0019471 | 0.024 | 11.385 | 0 | 2 | 6 | 4-hydroxyproline metabolic process |
| GO:0042178 | 0.024 | 11.385 | 0 | 2 | 6 | xenobiotic catabolic process |
| GO:0042273 | 0.024 | 11.385 | 0 | 2 | 6 | ribosomal large subunit biogenesis |
| GO:0045176 | 0.024 | 11.385 | 0 | 2 | 6 | apical protein localization |
| GO:0048262 | 0.024 | 11.385 | 0 | 2 | 6 | determination of dorsal/ventral asymmetry |
| GO:0048570 | 0.024 | 11.385 | 0 | 2 | 6 | notochord morphogenesis |
| GO:0070141 | 0.024 | 11.385 | 0 | 2 | 6 | response to UV-A |
| GO:2000258 | 0.024 | 11.385 | 0 | 2 | 6 | negative regulation of protein activation cascade |
| GO:0002474 | 0.025 | 2.418 | 4 | 8 | 84 | antigen processing and presentation of peptide antigen via MHC class I |
| GO:0009410 | 0.025 | 2.274 | 4 | 9 | 100 | response to xenobiotic stimulus |
| GO:0009156 | 0.026 | 3.974 | 1 | 4 | 27 | ribonucleoside monophosphate biosynthetic process |
| GO:0042633 | 0.026 | 2.864 | 2 | 6 | 54 | hair cycle |
| GO:0005996 | 0.027 | 1.825 | 9 | 15 | 205 | monosaccharide metabolic process |
| GO:0042744 | 0.028 | 5.264 | 1 | 3 | 16 | hydrogen peroxide catabolic process |
| GO:0070373 | 0.028 | 5.264 | 1 | 3 | 16 | negative regulation of ERK1 and ERK2 cascade |
| GO:0019752 | 0.029 | 1.476 | 24 | 33 | 556 | carboxylic acid metabolic process |
| GO:0051259 | 0.030 | 1.762 | 10 | 16 | 226 | protein oligomerization |
| GO:0034329 | 0.030 | 2.010 | 6 | 11 | 137 | cell junction assembly |
| GO:0030168 | 0.030 | 1.837 | 8 | 14 | 190 | platelet activation |
| GO:0009967 | 0.031 | 1.453 | 25 | 35 | 599 | positive regulation of signal transduction |
| GO:0002526 | 0.032 | 2.303 | 4 | 8 | 88 | acute inflammatory response |
| GO:0009108 | 0.032 | 2.695 | 2 | 6 | 57 | coenzyme biosynthetic process |
| GO:0034130 | 0.032 | 2.695 | 2 | 6 | 57 | toll-like receptor 1 signaling pathway |
| GO:0002921 | 0.033 | 9.107 | 0 | 2 | 7 | negative regulation of humoral immune response |
| GO:0006538 | 0.033 | 9.107 | 0 | 2 | 7 | glutamate catabolic process |
| GO:0042135 | 0.033 | 9.107 | 0 | 2 | 7 | neurotransmitter catabolic process |
| GO:0071501 | 0.033 | 9.107 | 0 | 2 | 7 | cellular response to sterol depletion |
| GO:1900024 | 0.033 | 9.107 | 0 | 2 | 7 | regulation of substrate adhesion-dependent cell spreading |
| GO:0006890 | 0.033 | 4.888 | 1 | 3 | 17 | retrograde vesicle-mediated transport, Golgi to ER |
| GO:0022415 | 0.033 | 1.519 | 19 | 27 | 441 | viral reproductive process |
| GO:0090407 | 0.034 | 1.572 | 15 | 23 | 364 | organophosphate biosynthetic process |
| GO:0001934 | 0.034 | 1.515 | 19 | 27 | 442 | positive regulation of protein phosphorylation |
| GO:0043200 | 0.035 | 2.643 | 2 | 6 | 58 | response to amino acid stimulus |
| GO:0010741 | 0.035 | 2.132 | 4 | 9 | 106 | negative regulation of intracellular protein kinase cascade |
| GO:0052548 | 0.035 | 1.795 | 8 | 14 | 194 | regulation of endopeptidase activity |
| GO:0006643 | 0.036 | 2.239 | 4 | 8 | 90 | membrane lipid metabolic process |
| GO:0000271 | 0.037 | 2.932 | 2 | 5 | 44 | polysaccharide biosynthetic process |
| GO:0009127 | 0.038 | 4.561 | 1 | 3 | 18 | purine nucleoside monophosphate biosynthetic process |
| GO:0006614 | 0.039 | 2.360 | 3 | 7 | 75 | SRP-dependent cotranslational protein targeting to membrane |
| GO:0042455 | 0.039 | 2.360 | 3 | 7 | 75 | ribonucleoside biosynthetic process |
| GO:0009267 | 0.039 | 2.882 | 2 | 5 | 45 | cellular response to starvation |
| GO:0043297 | 0.040 | 3.383 | 1 | 4 | 31 | apical junction assembly |
| GO:0060443 | 0.040 | 2.859 | 2 | 5 | 45 | mammary gland morphogenesis |
| GO:0034134 | 0.040 | 2.544 | 3 | 6 | 60 | toll-like receptor 2 signaling pathway |
| GO:0046546 | 0.040 | 2.185 | 4 | 8 | 92 | development of primary male sexual characteristics |
| GO:0043627 | 0.041 | 2.067 | 5 | 9 | 109 | response to estrogen stimulus |
| GO:0016197 | 0.041 | 2.325 | 3 | 7 | 76 | endosomal transport |
| GO:0001967 | 0.042 | 7.588 | 0 | 2 | 8 | suckling behavior |
| GO:0006013 | 0.042 | 7.588 | 0 | 2 | 8 | mannose metabolic process |
| GO:0006356 | 0.042 | 7.588 | 0 | 2 | 8 | regulation of transcription from RNA polymerase I promoter |
| GO:0006740 | 0.042 | 7.588 | 0 | 2 | 8 | NADPH regeneration |
| GO:0006878 | 0.042 | 7.588 | 0 | 2 | 8 | cellular copper ion homeostasis |
| GO:0009650 | 0.042 | 7.588 | 0 | 2 | 8 | UV protection |
| GO:0043312 | 0.042 | 7.588 | 0 | 2 | 8 | neutrophil degranulation |
| GO:0048617 | 0.042 | 7.588 | 0 | 2 | 8 | embryonic foregut morphogenesis |
| GO:0060252 | 0.042 | 7.588 | 0 | 2 | 8 | positive regulation of glial cell proliferation |
| GO:0060911 | 0.042 | 7.588 | 0 | 2 | 8 | cardiac cell fate commitment |
| GO:2000008 | 0.042 | 7.588 | 0 | 2 | 8 | regulation of protein localization to cell surface |
| GO:0000046 | 0.042 | Inf | 0 | 1 | 1 | autophagic vacuole fusion |
| GO:0000160 | 0.042 | Inf | 0 | 1 | 1 | phosphorelay signal transduction system |
| GO:0001575 | 0.042 | Inf | 0 | 1 | 1 | globoside metabolic process |
| GO:0001971 | 0.042 | Inf | 0 | 1 | 1 | negative regulation of activation of membrane attack complex |
| GO:0002543 | 0.042 | Inf | 0 | 1 | 1 | activation of blood coagulation via clotting cascade |
| GO:0002651 | 0.042 | Inf | 0 | 1 | 1 | positive regulation of tolerance induction to self antigen |
| GO:0006042 | 0.042 | Inf | 0 | 1 | 1 | glucosamine biosynthetic process |
| GO:0006430 | 0.042 | Inf | 0 | 1 | 1 | lysyl-tRNA aminoacylation |
| GO:0006747 | 0.042 | Inf | 0 | 1 | 1 | FAD biosynthetic process |
| GO:0007207 | 0.042 | Inf | 0 | 1 | 1 | phospholipase C-activating G-protein coupled acetylcholine receptor signaling pathway |
| GO:0009443 | 0.042 | Inf | 0 | 1 | 1 | pyridoxal 5'-phosphate salvage |
| GO:0014810 | 0.042 | Inf | 0 | 1 | 1 | positive regulation of skeletal muscle contraction by regulation of release of sequestered calcium ion |
| GO:0015904 | 0.042 | Inf | 0 | 1 | 1 | tetracycline transport |
| GO:0019464 | 0.042 | Inf | 0 | 1 | 1 | glycine decarboxylation via glycine cleavage system |
| GO:0021589 | 0.042 | Inf | 0 | 1 | 1 | cerebellum structural organization |
| GO:0021682 | 0.042 | Inf | 0 | 1 | 1 | nerve maturation |
| GO:0032618 | 0.042 | Inf | 0 | 1 | 1 | interleukin-15 production |
| GO:0033306 | 0.042 | Inf | 0 | 1 | 1 | phytol metabolic process |
| GO:0033615 | 0.042 | Inf | 0 | 1 | 1 | mitochondrial proton-transporting ATP synthase complex assembly |
| GO:0033986 | 0.042 | Inf | 0 | 1 | 1 | response to methanol |
| GO:0035105 | 0.042 | Inf | 0 | 1 | 1 | sterol regulatory element binding protein import into nucleus |
| GO:0035752 | 0.042 | Inf | 0 | 1 | 1 | lysosomal lumen pH elevation |
| GO:0035928 | 0.042 | Inf | 0 | 1 | 1 | rRNA import into mitochondrion |
| GO:0036111 | 0.042 | Inf | 0 | 1 | 1 | very long-chain fatty-acyl-CoA metabolic process |
| GO:0036112 | 0.042 | Inf | 0 | 1 | 1 | medium-chain fatty-acyl-CoA metabolic process |
| GO:0042697 | 0.042 | Inf | 0 | 1 | 1 | menopause |
| GO:0042816 | 0.042 | Inf | 0 | 1 | 1 | vitamin B6 metabolic process |
| GO:0042822 | 0.042 | Inf | 0 | 1 | 1 | pyridoxal phosphate metabolic process |
| GO:0042998 | 0.042 | Inf | 0 | 1 | 1 | positive regulation of Golgi to plasma membrane protein transport |
| GO:0043000 | 0.042 | Inf | 0 | 1 | 1 | Golgi to plasma membrane CFTR protein transport |
| GO:0043006 | 0.042 | Inf | 0 | 1 | 1 | activation of phospholipase A2 activity by calcium-mediated signaling |
| GO:0043128 | 0.042 | Inf | 0 | 1 | 1 | positive regulation of 1-phosphatidylinositol 4-kinase activity |
| GO:0043538 | 0.042 | Inf | 0 | 1 | 1 | regulation of actin phosphorylation |
| GO:0045083 | 0.042 | Inf | 0 | 1 | 1 | negative regulation of interleukin-12 biosynthetic process |
| GO:0045175 | 0.042 | Inf | 0 | 1 | 1 | basal protein localization |
| GO:0045887 | 0.042 | Inf | 0 | 1 | 1 | positive regulation of synaptic growth at neuromuscular junction |
| GO:0046108 | 0.042 | Inf | 0 | 1 | 1 | uridine metabolic process |
| GO:0046166 | 0.042 | Inf | 0 | 1 | 1 | glyceraldehyde-3-phosphate biosynthetic process |
| GO:0046836 | 0.042 | Inf | 0 | 1 | 1 | glycolipid transport |
| GO:0048105 | 0.042 | Inf | 0 | 1 | 1 | establishment of body hair planar orientation |
| GO:0050668 | 0.042 | Inf | 0 | 1 | 1 | positive regulation of homocysteine metabolic process |
| GO:0051490 | 0.042 | Inf | 0 | 1 | 1 | negative regulation of filopodium assembly |
| GO:0052250 | 0.042 | Inf | 0 | 1 | 1 | modulation of signal transduction in other organism involved in symbiotic interaction |
| GO:0060400 | 0.042 | Inf | 0 | 1 | 1 | negative regulation of growth hormone receptor signaling pathway |
| GO:0060434 | 0.042 | Inf | 0 | 1 | 1 | bronchus morphogenesis |
| GO:0060488 | 0.042 | Inf | 0 | 1 | 1 | orthogonal dichotomous subdivision of terminal units involved in lung branching morphogenesis |
| GO:0060489 | 0.042 | Inf | 0 | 1 | 1 | planar dichotomous subdivision of terminal units involved in lung branching morphogenesis |
| GO:0060490 | 0.042 | Inf | 0 | 1 | 1 | lateral sprouting involved in lung morphogenesis |
| GO:0060598 | 0.042 | Inf | 0 | 1 | 1 | dichotomous subdivision of terminal units involved in mammary gland duct morphogenesis |
| GO:0060904 | 0.042 | Inf | 0 | 1 | 1 | regulation of protein folding in endoplasmic reticulum |
| GO:0061445 | 0.042 | Inf | 0 | 1 | 1 | endocardial cushion cell fate commitment |
| GO:0071275 | 0.042 | Inf | 0 | 1 | 1 | cellular response to aluminum ion |
| GO:0071569 | 0.042 | Inf | 0 | 1 | 1 | protein ufmylation |
| GO:0071580 | 0.042 | Inf | 0 | 1 | 1 | regulation of zinc ion transmembrane transport |
| GO:0071582 | 0.042 | Inf | 0 | 1 | 1 | negative regulation of zinc ion transport |
| GO:0071584 | 0.042 | Inf | 0 | 1 | 1 | negative regulation of zinc ion transmembrane import |
| GO:0072053 | 0.042 | Inf | 0 | 1 | 1 | renal inner medulla development |
| GO:0072054 | 0.042 | Inf | 0 | 1 | 1 | renal outer medulla development |
| GO:0085032 | 0.042 | Inf | 0 | 1 | 1 | modulation by symbiont of host I-kappaB kinase/NF-kappaB cascade |
| GO:0090251 | 0.042 | Inf | 0 | 1 | 1 | protein localization involved in establishment of planar polarity |
| GO:1900163 | 0.042 | Inf | 0 | 1 | 1 | positive regulation of phospholipid scramblase activity |
| GO:2000680 | 0.042 | Inf | 0 | 1 | 1 | regulation of rubidium ion transport |
| GO:2000753 | 0.042 | Inf | 0 | 1 | 1 | positive regulation of glucosylceramide catabolic process |
| GO:2000755 | 0.042 | Inf | 0 | 1 | 1 | positive regulation of sphingomyelin catabolic process |
| GO:2000767 | 0.042 | Inf | 0 | 1 | 1 | positive regulation of cytoplasmic translation |
| GO:2001138 | 0.042 | Inf | 0 | 1 | 1 | regulation of phospholipid transport |
| GO:2001247 | 0.042 | Inf | 0 | 1 | 1 | positive regulation of phosphatidylcholine biosynthetic process |
| GO:0033674 | 0.043 | 1.560 | 14 | 21 | 333 | positive regulation of kinase activity |
| GO:0006793 | 0.043 | 1.259 | 74 | 87 | 1741 | phosphorus metabolic process |
| GO:0034637 | 0.044 | 2.788 | 2 | 5 | 46 | cellular carbohydrate biosynthetic process |
| GO:0030879 | 0.044 | 2.477 | 3 | 6 | 62 | mammary gland development |
| GO:0006644 | 0.045 | 1.731 | 8 | 14 | 202 | phospholipid metabolic process |
| GO:0072599 | 0.046 | 2.259 | 3 | 7 | 78 | establishment of protein localization to endoplasmic reticulum |
| GO:0009612 | 0.047 | 2.006 | 5 | 9 | 112 | response to mechanical stimulus |
| GO:0009064 | 0.047 | 2.722 | 2 | 5 | 47 | glutamine family amino acid metabolic process |
| GO:0030301 | 0.047 | 2.722 | 2 | 5 | 47 | cholesterol transport |
| GO:0031668 | 0.047 | 2.109 | 4 | 8 | 95 | cellular response to extracellular stimulus |
| GO:0045859 | 0.048 | 1.459 | 19 | 27 | 457 | regulation of protein kinase activity |
| GO:0071496 | 0.049 | 2.693 | 2 | 5 | 48 | cellular response to external stimulus |
| GO:0045185 | 0.049 | 2.227 | 3 | 7 | 79 | maintenance of protein location |
| GO:0030204 | 0.049 | 3.149 | 1 | 4 | 33 | chondroitin sulfate metabolic process |
| GO:0060425 | 0.049 | 3.149 | 1 | 4 | 33 | lung morphogenesis |
| GO:0006631 | 0.050 | 1.741 | 8 | 13 | 185 | fatty acid metabolic process |
| GO:0043603 | 0.050 | 2.215 | 3 | 7 | 80 | cellular amide metabolic process |
