# Supplementary material for: Hidden Treasures in “Ancient” Microarrays: Gene-Expression Portrays Biology and Potential Resistance Pathways of Major Lung Cancer Subtypes and Normal Tissue
Source: Front Oncol. 2014 Sep 29;4:251. doi: 10.3389/fonc.2014.00251 (PMC4178426; doi:10.3389/fonc.2014.00251)
Supplement: Figure S1 — Clinically relevant differentially expressed pathways in each tissue type versus the rest. The pathway maps are arranged according to the scheme in the left-top corner. Red indicates overexpression in genes, dark green downregulation. In the Cell Cycle pathway, tumor suppressor genes were overexpressed in normal lung tissue while oncogenes and tumor driving cyclins were overexpressed in cancers. The gene thymidylate synthase (TYMS or TS, belonging to the pathway “one carbon pool by folate”) is relevant for tumor growth and is also a treatment target. Notably, TYMS was overexpressed only in the tumors that are generally refractory to the drug pemetrexed, as the squamous and the small-cell lung cancer. TYMS was not overexpressed in mesothelioma, but it is known that TYMS expression is highly variable in this cancer. In the ERBB pathway, the ERBB2/HER2 and ERBB3/HER3 were overexpressed in adenocarcinoma, while the ERBB4 was overexpressed in the carcinoids. [file Data_Sheet_1.ZIP › File S3.ZIP!/GO Analysis/Small Cell vs Rest Enrichment Analysis/Overrepresented GO terms of Up-regulated Genes.html]

Gene to GO BP Conditional test for over-representation

| GOBPID | Pvalue | OddsRatio | ExpCount | Count | Size | Term |
| --- | --- | --- | --- | --- | --- | --- |
| GO:0048285 | <0.0001 | 6.590 | 13 | 61 | 233 | organelle fission |
| GO:0051301 | <0.0001 | 5.240 | 17 | 66 | 301 | cell division |
| GO:0000278 | <0.0001 | 6.237 | 12 | 52 | 228 | mitotic cell cycle |
| GO:0000236 | <0.0001 | 15.798 | 3 | 22 | 46 | mitotic prometaphase |
| GO:0051325 | <0.0001 | 5.513 | 9 | 36 | 158 | interphase |
| GO:0006260 | <0.0001 | 5.349 | 9 | 37 | 160 | DNA replication |
| GO:0010564 | <0.0001 | 6.250 | 6 | 27 | 106 | regulation of cell cycle process |
| GO:0051276 | <0.0001 | 3.632 | 16 | 47 | 294 | chromosome organization |
| GO:0006310 | <0.0001 | 6.334 | 6 | 26 | 98 | DNA recombination |
| GO:0000075 | <0.0001 | 12.162 | 2 | 15 | 37 | cell cycle checkpoint |
| GO:0000086 | <0.0001 | 6.072 | 5 | 23 | 89 | G2/M transition of mitotic cell cycle |
| GO:0006281 | <0.0001 | 4.821 | 7 | 27 | 130 | DNA repair |
| GO:0000083 | <0.0001 | 23.153 | 1 | 11 | 19 | regulation of transcription involved in G1/S phase of mitotic cell cycle |
| GO:0000070 | <0.0001 | 28.495 | 1 | 10 | 16 | mitotic sister chromatid segregation |
| GO:0006807 | <0.0001 | 1.754 | 199 | 258 | 3468 | nitrogen compound metabolic process |
| GO:0007018 | <0.0001 | 5.701 | 5 | 21 | 84 | microtubule-based movement |
| GO:0006139 | <0.0001 | 2.483 | 33 | 66 | 671 | nucleobase-containing compound metabolic process |
| GO:0006271 | <0.0001 | 15.524 | 1 | 11 | 23 | DNA strand elongation involved in DNA replication |
| GO:0006270 | <0.0001 | 13.219 | 1 | 11 | 25 | DNA replication initiation |
| GO:0016043 | <0.0001 | 1.863 | 88 | 132 | 1643 | cellular component organization |
| GO:2000112 | <0.0001 | 1.760 | 102 | 150 | 1787 | regulation of cellular macromolecule biosynthetic process |
| GO:0000084 | <0.0001 | 4.155 | 7 | 24 | 123 | S phase of mitotic cell cycle |
| GO:0033554 | <0.0001 | 2.023 | 49 | 85 | 849 | cellular response to stress |
| GO:0051321 | <0.0001 | 4.755 | 5 | 20 | 92 | meiotic cell cycle |
| GO:2001141 | <0.0001 | 1.755 | 94 | 139 | 1641 | regulation of RNA biosynthetic process |
| GO:0022402 | <0.0001 | 4.057 | 7 | 23 | 146 | cell cycle process |
| GO:0000722 | <0.0001 | 11.990 | 1 | 10 | 24 | telomere maintenance via recombination |
| GO:0032201 | <0.0001 | 11.990 | 1 | 10 | 24 | telomere maintenance via semi-conservative replication |
| GO:0010468 | <0.0001 | 1.742 | 92 | 135 | 1659 | regulation of gene expression |
| GO:0007062 | <0.0001 | 13.709 | 1 | 9 | 20 | sister chromatid cohesion |
| GO:0031577 | <0.0001 | 10.489 | 1 | 10 | 26 | spindle checkpoint |
| GO:0006284 | <0.0001 | 8.805 | 2 | 11 | 32 | base-excision repair |
| GO:0007051 | <0.0001 | 24.151 | 1 | 7 | 12 | spindle organization |
| GO:0071824 | <0.0001 | 4.427 | 5 | 18 | 87 | protein-DNA complex subunit organization |
| GO:0006297 | <0.0001 | 14.864 | 1 | 8 | 17 | nucleotide-excision repair, DNA gap filling |
| GO:0007059 | <0.0001 | 7.001 | 2 | 12 | 42 | chromosome segregation |
| GO:0007067 | <0.0001 | 4.521 | 5 | 17 | 87 | mitosis |
| GO:0034654 | <0.0001 | 1.588 | 119 | 161 | 2083 | nucleobase-containing compound biosynthetic process |
| GO:0007399 | <0.0001 | 1.704 | 69 | 103 | 1201 | nervous system development |
| GO:0006334 | <0.0001 | 5.765 | 3 | 12 | 47 | nucleosome assembly |
| GO:0007126 | <0.0001 | 4.206 | 5 | 16 | 81 | meiosis |
| GO:0006323 | <0.0001 | 5.177 | 3 | 13 | 56 | DNA packaging |
| GO:0030071 | <0.0001 | 10.351 | 1 | 8 | 21 | regulation of mitotic metaphase/anaphase transition |
| GO:0007094 | <0.0001 | 10.285 | 1 | 8 | 21 | mitotic cell cycle spindle assembly checkpoint |
| GO:0007093 | <0.0001 | 3.541 | 6 | 19 | 110 | mitotic cell cycle checkpoint |
| GO:0032200 | <0.0001 | 5.452 | 3 | 12 | 49 | telomere organization |
| GO:0010558 | <0.0001 | 1.896 | 38 | 64 | 659 | negative regulation of macromolecule biosynthetic process |
| GO:0009892 | <0.0001 | 1.722 | 58 | 89 | 1016 | negative regulation of metabolic process |
| GO:0000375 | <0.0001 | 2.989 | 9 | 23 | 154 | RNA splicing, via transesterification reactions |
| GO:0009889 | <0.0001 | 1.547 | 113 | 151 | 1972 | regulation of biosynthetic process |
| GO:0000079 | <0.0001 | 4.654 | 3 | 13 | 60 | regulation of cyclin-dependent protein kinase activity |
| GO:0034622 | <0.0001 | 2.200 | 20 | 39 | 345 | cellular macromolecular complex assembly |
| GO:0031327 | <0.0001 | 1.830 | 40 | 65 | 690 | negative regulation of cellular biosynthetic process |
| GO:0019985 | <0.0001 | 66.359 | 0 | 4 | 5 | translesion synthesis |
| GO:0007076 | <0.0001 | 20.775 | 1 | 5 | 9 | mitotic chromosome condensation |
| GO:0000724 | <0.0001 | 6.020 | 2 | 9 | 34 | double-strand break repair via homologous recombination |
| GO:0045839 | <0.0001 | 7.031 | 2 | 8 | 27 | negative regulation of mitosis |
| GO:0051983 | <0.0001 | 16.764 | 1 | 5 | 10 | regulation of chromosome segregation |
| GO:0045132 | <0.0001 | 16.618 | 1 | 5 | 10 | meiotic chromosome segregation |
| GO:0009059 | <0.0001 | 1.527 | 96 | 127 | 1781 | macromolecule biosynthetic process |
| GO:0016568 | <0.0001 | 2.199 | 16 | 32 | 281 | chromatin modification |
| GO:0031145 | <0.0001 | 3.832 | 4 | 13 | 70 | anaphase-promoting complex-dependent proteasomal ubiquitin-dependent protein catabolic process |
| GO:0045665 | <0.0001 | 5.573 | 2 | 9 | 36 | negative regulation of neuron differentiation |
| GO:0016446 | <0.0001 | 33.175 | 0 | 4 | 6 | somatic hypermutation of immunoglobulin genes |
| GO:0006396 | <0.0001 | 1.995 | 21 | 39 | 375 | RNA processing |
| GO:0051303 | <0.0001 | 13.846 | 1 | 5 | 11 | establishment of chromosome localization |
| GO:0010389 | <0.0001 | 7.292 | 1 | 7 | 23 | regulation of G2/M transition of mitotic cell cycle |
| GO:0032465 | <0.0001 | 9.077 | 1 | 6 | 17 | regulation of cytokinesis |
| GO:0000082 | <0.0001 | 2.718 | 8 | 19 | 140 | G1/S transition of mitotic cell cycle |
| GO:0070647 | <0.0001 | 1.970 | 21 | 38 | 369 | protein modification by small protein conjugation or removal |
| GO:0051488 | <0.0001 | 22.114 | 0 | 4 | 7 | activation of anaphase-promoting complex activity |
| GO:0006298 | <0.0001 | 11.917 | 1 | 5 | 12 | mismatch repair |
| GO:0071704 | <0.0001 | 1.446 | 297 | 330 | 5180 | organic substance metabolic process |
| GO:0006259 | <0.0001 | 3.593 | 4 | 12 | 78 | DNA metabolic process |
| GO:0048523 | <0.0001 | 1.442 | 109 | 139 | 1939 | negative regulation of cellular process |
| GO:0045685 | 0.001 | 5.133 | 2 | 8 | 34 | regulation of glial cell differentiation |
| GO:0006403 | 0.001 | 3.094 | 5 | 14 | 90 | RNA localization |
| GO:0000216 | 0.001 | 3.468 | 4 | 12 | 70 | M/G1 transition of mitotic cell cycle |
| GO:0006283 | 0.001 | 4.421 | 2 | 9 | 43 | transcription-coupled nucleotide-excision repair |
| GO:0045787 | 0.001 | 3.647 | 4 | 11 | 62 | positive regulation of cell cycle |
| GO:0010639 | 0.001 | 2.676 | 7 | 17 | 124 | negative regulation of organelle organization |
| GO:0009396 | 0.001 | 49.658 | 0 | 3 | 4 | folic acid-containing compound biosynthetic process |
| GO:0034501 | 0.001 | 49.658 | 0 | 3 | 4 | protein localization to kinetochore |
| GO:0006351 | 0.001 | 1.641 | 41 | 61 | 776 | transcription, DNA-dependent |
| GO:0000279 | 0.001 | 3.530 | 4 | 11 | 71 | M phase |
| GO:0043044 | 0.001 | 9.227 | 1 | 5 | 14 | ATP-dependent chromatin remodeling |
| GO:0000122 | 0.001 | 1.887 | 21 | 36 | 362 | negative regulation of transcription from RNA polymerase II promoter |
| GO:0046653 | 0.001 | 13.265 | 1 | 4 | 9 | tetrahydrofolate metabolic process |
| GO:0016070 | 0.001 | 1.513 | 62 | 84 | 1168 | RNA metabolic process |
| GO:0022607 | 0.001 | 1.506 | 58 | 80 | 1009 | cellular component assembly |
| GO:0016567 | 0.002 | 1.895 | 18 | 31 | 309 | protein ubiquitination |
| GO:0015931 | 0.002 | 2.731 | 6 | 14 | 100 | nucleobase-containing compound transport |
| GO:0006333 | 0.002 | 3.446 | 3 | 10 | 59 | chromatin assembly or disassembly |
| GO:0007091 | 0.002 | 25.400 | 0 | 3 | 5 | mitotic metaphase/anaphase transition |
| GO:0008608 | 0.002 | 24.986 | 0 | 3 | 5 | attachment of spindle microtubules to kinetochore |
| GO:0051322 | 0.002 | 11.052 | 1 | 4 | 10 | anaphase |
| GO:0051988 | 0.002 | 24.826 | 0 | 3 | 5 | regulation of attachment of spindle microtubules to kinetochore |
| GO:0061351 | 0.002 | 5.605 | 1 | 6 | 24 | neural precursor cell proliferation |
| GO:0050789 | 0.002 | 1.355 | 284 | 313 | 4958 | regulation of biological process |
| GO:0050767 | 0.002 | 5.432 | 1 | 6 | 25 | regulation of neurogenesis |
| GO:0051298 | 0.002 | 6.918 | 1 | 5 | 17 | centrosome duplication |
| GO:0000085 | 0.003 | 9.472 | 1 | 4 | 11 | G2 phase of mitotic cell cycle |
| GO:0031570 | 0.003 | 2.579 | 6 | 14 | 105 | DNA integrity checkpoint |
| GO:0006302 | 0.003 | 5.088 | 1 | 6 | 26 | double-strand break repair |
| GO:0050684 | 0.003 | 4.315 | 2 | 7 | 34 | regulation of mRNA processing |
| GO:0051084 | 0.003 | 4.160 | 2 | 7 | 35 | 'de novo' posttranslational protein folding |
| GO:0009263 | 0.003 | Inf | 0 | 2 | 2 | deoxyribonucleotide biosynthetic process |
| GO:0051383 | 0.003 | Inf | 0 | 2 | 2 | kinetochore organization |
| GO:0007079 | 0.003 | Inf | 0 | 2 | 2 | mitotic chromosome movement towards spindle pole |
| GO:0009186 | 0.003 | Inf | 0 | 2 | 2 | deoxyribonucleoside diphosphate metabolic process |
| GO:0010032 | 0.003 | Inf | 0 | 2 | 2 | meiotic chromosome condensation |
| GO:0010994 | 0.003 | Inf | 0 | 2 | 2 | free ubiquitin chain polymerization |
| GO:0048478 | 0.003 | Inf | 0 | 2 | 2 | replication fork protection |
| GO:0051984 | 0.003 | Inf | 0 | 2 | 2 | positive regulation of chromosome segregation |
| GO:0090007 | 0.003 | Inf | 0 | 2 | 2 | regulation of mitotic anaphase |
| GO:0000731 | 0.003 | 16.548 | 0 | 3 | 6 | DNA synthesis involved in DNA repair |
| GO:0045842 | 0.003 | 16.548 | 0 | 3 | 6 | positive regulation of mitotic metaphase/anaphase transition |
| GO:0007131 | 0.004 | 4.748 | 2 | 6 | 27 | reciprocal meiotic recombination |
| GO:0016444 | 0.004 | 4.016 | 2 | 7 | 36 | somatic cell DNA recombination |
| GO:0021953 | 0.004 | 3.001 | 4 | 10 | 66 | central nervous system neuron differentiation |
| GO:0002200 | 0.004 | 3.882 | 2 | 7 | 37 | somatic diversification of immune receptors |
| GO:0051297 | 0.005 | 7.516 | 1 | 4 | 13 | centrosome organization |
| GO:0015949 | 0.005 | 7.365 | 1 | 4 | 13 | nucleobase-containing small molecule interconversion |
| GO:0016447 | 0.005 | 4.334 | 2 | 6 | 29 | somatic recombination of immunoglobulin gene segments |
| GO:0034968 | 0.005 | 4.334 | 2 | 6 | 29 | histone lysine methylation |
| GO:0007098 | 0.005 | 12.583 | 0 | 3 | 7 | centrosome cycle |
| GO:0007420 | 0.005 | 2.137 | 9 | 17 | 155 | brain development |
| GO:0006268 | 0.006 | 12.409 | 0 | 3 | 7 | DNA unwinding involved in replication |
| GO:0006273 | 0.006 | 12.409 | 0 | 3 | 7 | lagging strand elongation |
| GO:0048715 | 0.006 | 12.409 | 0 | 3 | 7 | negative regulation of oligodendrocyte differentiation |
| GO:0014014 | 0.006 | 5.185 | 1 | 5 | 21 | negative regulation of gliogenesis |
| GO:0030900 | 0.006 | 2.123 | 9 | 17 | 153 | forebrain development |
| GO:0045892 | 0.006 | 1.978 | 11 | 20 | 197 | negative regulation of transcription, DNA-dependent |
| GO:0016071 | 0.006 | 1.643 | 23 | 36 | 408 | mRNA metabolic process |
| GO:0010498 | 0.006 | 2.056 | 9 | 18 | 165 | proteasomal protein catabolic process |
| GO:0044260 | 0.006 | 1.481 | 52 | 68 | 1261 | cellular macromolecule metabolic process |
| GO:0021515 | 0.007 | 4.880 | 1 | 5 | 22 | cell differentiation in spinal cord |
| GO:0043484 | 0.008 | 3.423 | 2 | 7 | 41 | regulation of RNA splicing |
| GO:0021543 | 0.008 | 2.647 | 4 | 10 | 73 | pallium development |
| GO:0007052 | 0.008 | 6.114 | 1 | 4 | 15 | mitotic spindle organization |
| GO:0009219 | 0.008 | 9.926 | 0 | 3 | 8 | pyrimidine deoxyribonucleotide metabolic process |
| GO:0034080 | 0.008 | 9.926 | 0 | 3 | 8 | CENP-A containing nucleosome assembly at centromere |
| GO:0034724 | 0.008 | 9.926 | 0 | 3 | 8 | DNA replication-independent nucleosome organization |
| GO:0045910 | 0.008 | 9.926 | 0 | 3 | 8 | negative regulation of DNA recombination |
| GO:0051318 | 0.009 | 3.325 | 2 | 7 | 42 | G1 phase |
| GO:0000212 | 0.009 | 33.031 | 0 | 2 | 3 | meiotic spindle organization |
| GO:0006235 | 0.009 | 33.031 | 0 | 2 | 3 | dTTP biosynthetic process |
| GO:0008054 | 0.009 | 33.031 | 0 | 2 | 3 | cyclin catabolic process |
| GO:0016199 | 0.009 | 33.031 | 0 | 2 | 3 | axon midline choice point recognition |
| GO:0021530 | 0.009 | 33.031 | 0 | 2 | 3 | spinal cord oligodendrocyte cell fate specification |
| GO:0030910 | 0.009 | 33.031 | 0 | 2 | 3 | olfactory placode formation |
| GO:0030951 | 0.009 | 33.031 | 0 | 2 | 3 | establishment or maintenance of microtubule cytoskeleton polarity |
| GO:0031536 | 0.009 | 33.031 | 0 | 2 | 3 | positive regulation of exit from mitosis |
| GO:0034723 | 0.009 | 33.031 | 0 | 2 | 3 | DNA replication-dependent nucleosome organization |
| GO:0051256 | 0.009 | 33.031 | 0 | 2 | 3 | spindle midzone assembly involved in mitosis |
| GO:0060166 | 0.009 | 33.031 | 0 | 2 | 3 | olfactory pit development |
| GO:0060236 | 0.009 | 33.031 | 0 | 2 | 3 | regulation of mitotic spindle organization |
| GO:0071698 | 0.009 | 33.031 | 0 | 2 | 3 | olfactory placode development |
| GO:2001240 | 0.009 | 33.031 | 0 | 2 | 3 | negative regulation of extrinsic apoptotic signaling pathway in absence of ligand |
| GO:0019219 | 0.009 | 1.469 | 40 | 54 | 759 | regulation of nucleobase-containing compound metabolic process |
| GO:0045893 | 0.010 | 1.449 | 41 | 56 | 720 | positive regulation of transcription, DNA-dependent |
| GO:0090068 | 0.010 | 2.170 | 7 | 14 | 123 | positive regulation of cell cycle process |
| GO:0090090 | 0.010 | 2.958 | 3 | 8 | 53 | negative regulation of canonical Wnt receptor signaling pathway |
| GO:0045931 | 0.010 | 3.690 | 2 | 6 | 33 | positive regulation of mitotic cell cycle |
| GO:0030705 | 0.010 | 3.232 | 2 | 7 | 43 | cytoskeleton-dependent intracellular transport |
| GO:0009987 | 0.011 | 1.725 | 266 | 278 | 5240 | cellular process |
| GO:0016572 | 0.011 | 5.522 | 1 | 4 | 16 | histone phosphorylation |
| GO:0051439 | 0.011 | 2.676 | 4 | 9 | 65 | regulation of ubiquitin-protein ligase activity involved in mitotic cell cycle |
| GO:0048025 | 0.012 | 8.271 | 1 | 3 | 9 | negative regulation of mRNA splicing, via spliceosome |
| GO:0048712 | 0.012 | 8.271 | 1 | 3 | 9 | negative regulation of astrocyte differentiation |
| GO:0048935 | 0.012 | 8.271 | 1 | 3 | 9 | peripheral nervous system neuron development |
| GO:0051571 | 0.012 | 8.271 | 1 | 3 | 9 | positive regulation of histone H3-K4 methylation |
| GO:0021536 | 0.012 | 2.832 | 3 | 8 | 55 | diencephalon development |
| GO:0016570 | 0.013 | 1.925 | 9 | 17 | 166 | histone modification |
| GO:0072527 | 0.013 | 3.061 | 3 | 7 | 45 | pyrimidine-containing compound metabolic process |
| GO:0006308 | 0.014 | 2.772 | 3 | 8 | 56 | DNA catabolic process |
| GO:0019692 | 0.014 | 5.096 | 1 | 4 | 17 | deoxyribose phosphate metabolic process |
| GO:0042558 | 0.014 | 5.096 | 1 | 4 | 17 | pteridine-containing compound metabolic process |
| GO:0090329 | 0.014 | 5.096 | 1 | 4 | 17 | regulation of DNA-dependent DNA replication |
| GO:0008283 | 0.014 | 1.348 | 62 | 78 | 1098 | cell proliferation |
| GO:0090305 | 0.015 | 2.982 | 3 | 7 | 46 | nucleic acid phosphodiester bond hydrolysis |
| GO:0031175 | 0.015 | 1.499 | 28 | 40 | 496 | neuron projection development |
| GO:0032886 | 0.015 | 2.715 | 3 | 8 | 57 | regulation of microtubule-based process |
| GO:0045595 | 0.016 | 1.405 | 42 | 56 | 736 | regulation of cell differentiation |
| GO:0033043 | 0.016 | 1.842 | 10 | 18 | 186 | regulation of organelle organization |
| GO:0030261 | 0.016 | 7.165 | 1 | 3 | 10 | chromosome condensation |
| GO:0090307 | 0.016 | 7.119 | 1 | 3 | 10 | spindle assembly involved in mitosis |
| GO:0048665 | 0.016 | 7.116 | 1 | 3 | 10 | neuron fate specification |
| GO:0071777 | 0.017 | 7.088 | 1 | 3 | 10 | positive regulation of cell cycle cytokinesis |
| GO:0048489 | 0.017 | 2.907 | 3 | 7 | 47 | synaptic vesicle transport |
| GO:0000245 | 0.017 | 3.768 | 2 | 5 | 27 | spliceosomal complex assembly |
| GO:0008156 | 0.017 | 3.768 | 2 | 5 | 27 | negative regulation of DNA replication |
| GO:0010977 | 0.017 | 3.768 | 2 | 5 | 27 | negative regulation of neuron projection development |
| GO:0043392 | 0.017 | 3.768 | 2 | 5 | 27 | negative regulation of DNA binding |
| GO:0051785 | 0.017 | 3.768 | 2 | 5 | 27 | positive regulation of nuclear division |
| GO:2000179 | 0.017 | 3.768 | 2 | 5 | 27 | positive regulation of neural precursor cell proliferation |
| GO:0031060 | 0.017 | 4.732 | 1 | 4 | 18 | regulation of histone methylation |
| GO:0042771 | 0.017 | 4.732 | 1 | 4 | 18 | intrinsic apoptotic signaling pathway in response to DNA damage by p53 class mediator |
| GO:0044249 | 0.017 | 1.269 | 122 | 141 | 2261 | cellular biosynthetic process |
| GO:0048015 | 0.017 | 2.067 | 7 | 13 | 118 | phosphatidylinositol-mediated signaling |
| GO:0042063 | 0.018 | 2.126 | 6 | 12 | 107 | gliogenesis |
| GO:0000389 | 0.018 | 16.513 | 0 | 2 | 4 | mRNA 3'-splice site recognition |
| GO:0000710 | 0.018 | 16.513 | 0 | 2 | 4 | meiotic mismatch repair |
| GO:0003289 | 0.018 | 16.513 | 0 | 2 | 4 | atrial septum primum morphogenesis |
| GO:0003357 | 0.018 | 16.513 | 0 | 2 | 4 | noradrenergic neuron differentiation |
| GO:0007089 | 0.018 | 16.513 | 0 | 2 | 4 | traversing start control point of mitotic cell cycle |
| GO:0007549 | 0.018 | 16.513 | 0 | 2 | 4 | dosage compensation |
| GO:0009202 | 0.018 | 16.513 | 0 | 2 | 4 | deoxyribonucleoside triphosphate biosynthetic process |
| GO:0009211 | 0.018 | 16.513 | 0 | 2 | 4 | pyrimidine deoxyribonucleoside triphosphate metabolic process |
| GO:0021559 | 0.018 | 16.513 | 0 | 2 | 4 | trigeminal nerve development |
| GO:0021780 | 0.018 | 16.513 | 0 | 2 | 4 | glial cell fate specification |
| GO:0031573 | 0.018 | 16.513 | 0 | 2 | 4 | intra-S DNA damage checkpoint |
| GO:0042769 | 0.018 | 16.513 | 0 | 2 | 4 | DNA damage response, detection of DNA damage |
| GO:0045654 | 0.018 | 16.513 | 0 | 2 | 4 | positive regulation of megakaryocyte differentiation |
| GO:0050957 | 0.018 | 16.513 | 0 | 2 | 4 | equilibrioception |
| GO:0051096 | 0.018 | 16.513 | 0 | 2 | 4 | positive regulation of helicase activity |
| GO:0051290 | 0.018 | 16.513 | 0 | 2 | 4 | protein heterotetramerization |
| GO:0071168 | 0.018 | 16.513 | 0 | 2 | 4 | protein localization to chromatin |
| GO:0072574 | 0.018 | 16.513 | 0 | 2 | 4 | hepatocyte proliferation |
| GO:0072576 | 0.018 | 16.513 | 0 | 2 | 4 | liver morphogenesis |
| GO:0007281 | 0.020 | 2.281 | 5 | 10 | 83 | germ cell development |
| GO:0045664 | 0.020 | 1.868 | 9 | 16 | 162 | regulation of neuron differentiation |
| GO:0050905 | 0.020 | 2.558 | 3 | 8 | 60 | neuromuscular process |
| GO:0016925 | 0.021 | 4.433 | 1 | 4 | 19 | protein sumoylation |
| GO:0003209 | 0.021 | 4.416 | 1 | 4 | 19 | cardiac atrium morphogenesis |
| GO:0021517 | 0.021 | 4.416 | 1 | 4 | 19 | ventral spinal cord development |
| GO:0021772 | 0.021 | 4.416 | 1 | 4 | 19 | olfactory bulb development |
| GO:0043567 | 0.021 | 4.416 | 1 | 4 | 19 | regulation of insulin-like growth factor receptor signaling pathway |
| GO:0003283 | 0.022 | 6.201 | 1 | 3 | 11 | atrial septum development |
| GO:0006978 | 0.022 | 6.201 | 1 | 3 | 11 | DNA damage response, signal transduction by p53 class mediator resulting in transcription of p21 class mediator |
| GO:0055015 | 0.022 | 6.201 | 1 | 3 | 11 | ventricular cardiac muscle cell development |
| GO:0048858 | 0.023 | 1.462 | 27 | 38 | 477 | cell projection morphogenesis |
| GO:0051052 | 0.024 | 2.037 | 6 | 12 | 111 | regulation of DNA metabolic process |
| GO:0010948 | 0.024 | 2.974 | 2 | 6 | 40 | negative regulation of cell cycle process |
| GO:0051253 | 0.024 | 4.151 | 1 | 4 | 21 | negative regulation of RNA metabolic process |
| GO:0009790 | 0.027 | 1.388 | 36 | 48 | 634 | embryo development |
| GO:0051320 | 0.027 | 11.475 | 0 | 2 | 5 | S phase |
| GO:0021782 | 0.028 | 2.583 | 3 | 7 | 52 | glial cell development |
| GO:0009148 | 0.028 | 5.512 | 1 | 3 | 12 | pyrimidine nucleoside triphosphate biosynthetic process |
| GO:0046605 | 0.028 | 5.512 | 1 | 3 | 12 | regulation of centrosome cycle |
| GO:0071697 | 0.028 | 5.512 | 1 | 3 | 12 | ectodermal placode morphogenesis |
| GO:0048869 | 0.028 | 1.242 | 107 | 124 | 1859 | cellular developmental process |
| GO:0050658 | 0.028 | 11.206 | 0 | 2 | 5 | RNA transport |
| GO:0000729 | 0.029 | 11.007 | 0 | 2 | 5 | DNA double-strand break processing |
| GO:0001880 | 0.029 | 11.007 | 0 | 2 | 5 | Mullerian duct regression |
| GO:0002326 | 0.029 | 11.007 | 0 | 2 | 5 | B cell lineage commitment |
| GO:0007100 | 0.029 | 11.007 | 0 | 2 | 5 | mitotic centrosome separation |
| GO:0009265 | 0.029 | 11.007 | 0 | 2 | 5 | 2'-deoxyribonucleotide biosynthetic process |
| GO:0009448 | 0.029 | 11.007 | 0 | 2 | 5 | gamma-aminobutyric acid metabolic process |
| GO:0021779 | 0.029 | 11.007 | 0 | 2 | 5 | oligodendrocyte cell fate commitment |
| GO:0033600 | 0.029 | 11.007 | 0 | 2 | 5 | negative regulation of mammary gland epithelial cell proliferation |
| GO:0034661 | 0.029 | 11.007 | 0 | 2 | 5 | ncRNA catabolic process |
| GO:0046826 | 0.029 | 11.007 | 0 | 2 | 5 | negative regulation of protein export from nucleus |
| GO:0048026 | 0.029 | 11.007 | 0 | 2 | 5 | positive regulation of mRNA splicing, via spliceosome |
| GO:0048096 | 0.029 | 11.007 | 0 | 2 | 5 | chromatin-mediated maintenance of transcription |
| GO:0051573 | 0.029 | 11.007 | 0 | 2 | 5 | negative regulation of histone H3-K9 methylation |
| GO:0070562 | 0.029 | 11.007 | 0 | 2 | 5 | regulation of vitamin D receptor signaling pathway |
| GO:0085020 | 0.029 | 11.007 | 0 | 2 | 5 | protein K6-linked ubiquitination |
| GO:0007157 | 0.029 | 3.895 | 1 | 4 | 21 | heterophilic cell-cell adhesion |
| GO:0008038 | 0.029 | 3.895 | 1 | 4 | 21 | neuron recognition |
| GO:0021879 | 0.029 | 3.895 | 1 | 4 | 21 | forebrain neuron differentiation |
| GO:0000398 | 0.030 | 2.032 | 6 | 11 | 103 | mRNA splicing, via spliceosome |
| GO:0006282 | 0.030 | 3.187 | 2 | 5 | 31 | regulation of DNA repair |
| GO:0032024 | 0.030 | 3.187 | 2 | 5 | 31 | positive regulation of insulin secretion |
| GO:0006357 | 0.030 | 1.431 | 28 | 38 | 515 | regulation of transcription from RNA polymerase II promoter |
| GO:0007409 | 0.031 | 1.476 | 23 | 32 | 397 | axonogenesis |
| GO:0007088 | 0.031 | 3.144 | 2 | 5 | 32 | regulation of mitosis |
| GO:0045935 | 0.032 | 1.320 | 49 | 62 | 861 | positive regulation of nucleobase-containing compound metabolic process |
| GO:0010608 | 0.033 | 1.613 | 14 | 21 | 239 | posttranscriptional regulation of gene expression |
| GO:0051438 | 0.034 | 5.023 | 1 | 3 | 13 | regulation of ubiquitin-protein ligase activity |
| GO:0043967 | 0.034 | 3.068 | 2 | 5 | 32 | histone H4 acetylation |
| GO:0000087 | 0.034 | 9.351 | 0 | 2 | 6 | M phase of mitotic cell cycle |
| GO:0019886 | 0.034 | 2.291 | 4 | 8 | 66 | antigen processing and presentation of exogenous peptide antigen via MHC class II |
| GO:0009066 | 0.034 | 3.678 | 1 | 4 | 22 | aspartate family amino acid metabolic process |
| GO:0031572 | 0.034 | 4.979 | 1 | 3 | 13 | G2/M transition DNA damage checkpoint |
| GO:0006406 | 0.035 | 2.689 | 2 | 6 | 43 | mRNA export from nucleus |
| GO:0007019 | 0.035 | 4.960 | 1 | 3 | 13 | microtubule depolymerization |
| GO:0048385 | 0.035 | 4.960 | 1 | 3 | 13 | regulation of retinoic acid receptor signaling pathway |
| GO:0048857 | 0.035 | 4.960 | 1 | 3 | 13 | neural nucleus development |
| GO:0033044 | 0.036 | 2.137 | 5 | 9 | 79 | regulation of chromosome organization |
| GO:0002504 | 0.037 | 2.252 | 4 | 8 | 67 | antigen processing and presentation of peptide or polysaccharide antigen via MHC class II |
| GO:0051028 | 0.037 | 2.984 | 2 | 5 | 33 | mRNA transport |
| GO:0035108 | 0.037 | 2.028 | 5 | 10 | 92 | limb morphogenesis |
| GO:0007405 | 0.038 | 2.958 | 2 | 5 | 33 | neuroblast proliferation |
| GO:0042384 | 0.038 | 2.958 | 2 | 5 | 33 | cilium assembly |
| GO:0006913 | 0.040 | 1.560 | 15 | 22 | 258 | nucleocytoplasmic transport |
| GO:0010226 | 0.040 | 3.484 | 1 | 4 | 23 | response to lithium ion |
| GO:0051258 | 0.041 | 1.915 | 6 | 11 | 107 | protein polymerization |
| GO:0018394 | 0.041 | 2.077 | 5 | 9 | 81 | peptidyl-lysine acetylation |
| GO:0051225 | 0.041 | 4.554 | 1 | 3 | 14 | spindle assembly |
| GO:0006405 | 0.042 | 8.325 | 0 | 2 | 6 | RNA export from nucleus |
| GO:0033599 | 0.042 | 8.288 | 0 | 2 | 6 | regulation of mammary gland epithelial cell proliferation |
| GO:0006479 | 0.042 | 2.551 | 3 | 6 | 45 | protein methylation |
| GO:0060249 | 0.042 | 1.754 | 8 | 14 | 147 | anatomical structure homeostasis |
| GO:0000733 | 0.042 | 8.254 | 0 | 2 | 6 | DNA strand renaturation |
| GO:0006208 | 0.042 | 8.254 | 0 | 2 | 6 | pyrimidine nucleobase catabolic process |
| GO:0007080 | 0.042 | 8.254 | 0 | 2 | 6 | mitotic metaphase plate congression |
| GO:0007095 | 0.042 | 8.254 | 0 | 2 | 6 | mitotic cell cycle G2/M transition DNA damage checkpoint |
| GO:0009649 | 0.042 | 8.254 | 0 | 2 | 6 | entrainment of circadian clock |
| GO:0021520 | 0.042 | 8.254 | 0 | 2 | 6 | spinal cord motor neuron cell fate specification |
| GO:0021892 | 0.042 | 8.254 | 0 | 2 | 6 | cerebral cortex GABAergic interneuron differentiation |
| GO:0033235 | 0.042 | 8.254 | 0 | 2 | 6 | positive regulation of protein sumoylation |
| GO:0035970 | 0.042 | 8.254 | 0 | 2 | 6 | peptidyl-threonine dephosphorylation |
| GO:0038034 | 0.042 | 8.254 | 0 | 2 | 6 | signal transduction in absence of ligand |
| GO:0048880 | 0.042 | 8.254 | 0 | 2 | 6 | sensory system development |
| GO:0051103 | 0.042 | 8.254 | 0 | 2 | 6 | DNA ligation involved in DNA repair |
| GO:0051382 | 0.042 | 8.254 | 0 | 2 | 6 | kinetochore assembly |
| GO:0060235 | 0.042 | 8.254 | 0 | 2 | 6 | lens induction in camera-type eye |
| GO:0060896 | 0.042 | 8.254 | 0 | 2 | 6 | neural plate pattern specification |
| GO:0090129 | 0.042 | 8.254 | 0 | 2 | 6 | positive regulation of synapse maturation |
| GO:0097150 | 0.042 | 8.254 | 0 | 2 | 6 | neuronal stem cell maintenance |
| GO:0000380 | 0.042 | 4.508 | 1 | 3 | 14 | alternative mRNA splicing, via spliceosome |
| GO:0006303 | 0.042 | 4.508 | 1 | 3 | 14 | double-strand break repair via nonhomologous end joining |
| GO:0006337 | 0.042 | 4.508 | 1 | 3 | 14 | nucleosome disassembly |
| GO:0031290 | 0.042 | 4.508 | 1 | 3 | 14 | retinal ganglion cell axon guidance |
| GO:0006511 | 0.043 | 1.561 | 14 | 21 | 246 | ubiquitin-dependent protein catabolic process |
| GO:0045934 | 0.044 | 2.310 | 3 | 7 | 61 | negative regulation of nucleobase-containing compound metabolic process |
| GO:0007050 | 0.044 | 1.960 | 5 | 10 | 102 | cell cycle arrest |
| GO:0030262 | 0.045 | 3.309 | 1 | 4 | 24 | apoptotic nuclear changes |
| GO:0045666 | 0.046 | 2.486 | 3 | 6 | 46 | positive regulation of neuron differentiation |
| GO:0050807 | 0.047 | 2.760 | 2 | 5 | 35 | regulation of synapse organization |
| GO:0048736 | 0.048 | 1.933 | 6 | 10 | 96 | appendage development |
| GO:0050769 | 0.048 | 1.933 | 6 | 10 | 96 | positive regulation of neurogenesis |
| GO:0000226 | 0.049 | 1.851 | 6 | 11 | 115 | microtubule cytoskeleton organization |
