# Supplementary material for: Hidden Treasures in “Ancient” Microarrays: Gene-Expression Portrays Biology and Potential Resistance Pathways of Major Lung Cancer Subtypes and Normal Tissue
Source: Front Oncol. 2014 Sep 29;4:251. doi: 10.3389/fonc.2014.00251 (PMC4178426; doi:10.3389/fonc.2014.00251)
Supplement: Figure S1 — Clinically relevant differentially expressed pathways in each tissue type versus the rest. The pathway maps are arranged according to the scheme in the left-top corner. Red indicates overexpression in genes, dark green downregulation. In the Cell Cycle pathway, tumor suppressor genes were overexpressed in normal lung tissue while oncogenes and tumor driving cyclins were overexpressed in cancers. The gene thymidylate synthase (TYMS or TS, belonging to the pathway “one carbon pool by folate”) is relevant for tumor growth and is also a treatment target. Notably, TYMS was overexpressed only in the tumors that are generally refractory to the drug pemetrexed, as the squamous and the small-cell lung cancer. TYMS was not overexpressed in mesothelioma, but it is known that TYMS expression is highly variable in this cancer. In the ERBB pathway, the ERBB2/HER2 and ERBB3/HER3 were overexpressed in adenocarcinoma, while the ERBB4 was overexpressed in the carcinoids. [file Data_Sheet_1.ZIP › File S3.ZIP!/GO Analysis/Small Cell vs Rest Enrichment Analysis/Underrepresented GO terms of Differentially Expressed Genes.html]

Gene to GO BP Conditional test for under-representation

| GOBPID | Pvalue | OddsRatio | ExpCount | Count | Size | Term |
| --- | --- | --- | --- | --- | --- | --- |
| GO:1900542 | <0.0001 | 0.293 | 25 | 8 | 249 | regulation of purine nucleotide metabolic process |
| GO:0030799 | <0.0001 | <0.0001 | 9 | 0 | 90 | regulation of cyclic nucleotide metabolic process |
| GO:0007267 | <0.0001 | 0.593 | 81 | 52 | 810 | cell-cell signaling |
| GO:0030808 | <0.0001 | <0.0001 | 8 | 0 | 84 | regulation of nucleotide biosynthetic process |
| GO:0030817 | <0.0001 | <0.0001 | 8 | 0 | 76 | regulation of cAMP biosynthetic process |
| GO:0007186 | 0.001 | 0.466 | 34 | 17 | 337 | G-protein coupled receptor signaling pathway |
| GO:0050804 | 0.001 | 0.253 | 14 | 4 | 145 | regulation of synaptic transmission |
| GO:0051241 | 0.001 | 0.424 | 24 | 11 | 240 | negative regulation of multicellular organismal process |
| GO:0045981 | 0.002 | <0.0001 | 6 | 0 | 61 | positive regulation of nucleotide metabolic process |
| GO:0008217 | 0.002 | 0.233 | 12 | 3 | 118 | regulation of blood pressure |
| GO:0030804 | 0.002 | <0.0001 | 6 | 0 | 58 | positive regulation of cyclic nucleotide biosynthetic process |
| GO:1900373 | 0.002 | <0.0001 | 6 | 0 | 58 | positive regulation of purine nucleotide biosynthetic process |
| GO:0007165 | 0.003 | 0.799 | 280 | 245 | 2814 | signal transduction |
| GO:0052652 | 0.003 | 0.192 | 9 | 2 | 95 | cyclic purine nucleotide metabolic process |
| GO:0051339 | 0.003 | <0.0001 | 5 | 0 | 55 | regulation of lyase activity |
| GO:0034220 | 0.003 | 0.434 | 21 | 10 | 215 | ion transmembrane transport |
| GO:0030816 | 0.003 | <0.0001 | 5 | 0 | 54 | positive regulation of cAMP metabolic process |
| GO:0031279 | 0.003 | <0.0001 | 5 | 0 | 54 | regulation of cyclase activity |
| GO:0002685 | 0.003 | 0.121 | 7 | 1 | 75 | regulation of leukocyte migration |
| GO:0050778 | 0.004 | <0.0001 | 5 | 0 | 52 | positive regulation of immune response |
| GO:0006955 | 0.004 | 0.633 | 53 | 36 | 527 | immune response |
| GO:0006954 | 0.005 | 0.576 | 37 | 23 | 376 | inflammatory response |
| GO:0030099 | 0.005 | 0.436 | 19 | 9 | 193 | myeloid cell differentiation |
| GO:0032318 | 0.007 | 0.271 | 10 | 3 | 102 | regulation of Ras GTPase activity |
| GO:0009259 | 0.007 | 0.631 | 46 | 31 | 465 | ribonucleotide metabolic process |
| GO:0002690 | 0.007 | <0.0001 | 5 | 0 | 47 | positive regulation of leukocyte chemotaxis |
| GO:0045926 | 0.007 | 0.383 | 15 | 6 | 146 | negative regulation of growth |
| GO:0035303 | 0.007 | 0.136 | 7 | 1 | 67 | regulation of dephosphorylation |
| GO:0055085 | 0.008 | 0.498 | 23 | 12 | 223 | transmembrane transport |
| GO:0031644 | 0.008 | 0.386 | 14 | 6 | 144 | regulation of neurological system process |
| GO:0055080 | 0.008 | 0.570 | 31 | 19 | 315 | cation homeostasis |
| GO:0050886 | 0.009 | <0.0001 | 4 | 0 | 45 | endocrine process |
| GO:0044057 | 0.009 | 0.282 | 10 | 3 | 96 | regulation of system process |
| GO:0090066 | 0.010 | 0.506 | 22 | 12 | 223 | regulation of anatomical structure size |
| GO:0048534 | 0.010 | 0.631 | 42 | 28 | 421 | hematopoietic or lymphoid organ development |
| GO:0007188 | 0.010 | 0.227 | 8 | 2 | 81 | adenylate cyclase-modulating G-protein coupled receptor signaling pathway |
| GO:0072522 | 0.011 | 0.467 | 18 | 9 | 181 | purine-containing compound biosynthetic process |
| GO:0023052 | 0.011 | 0.834 | 313 | 283 | 3144 | signaling |
| GO:0006936 | 0.012 | 0.503 | 21 | 11 | 206 | muscle contraction |
| GO:0040017 | 0.012 | 0.503 | 21 | 11 | 206 | positive regulation of locomotion |
| GO:0030811 | 0.012 | 0.452 | 17 | 8 | 166 | regulation of nucleotide catabolic process |
| GO:0030198 | 0.013 | 0.377 | 12 | 5 | 123 | extracellular matrix organization |
| GO:0009152 | 0.013 | 0.455 | 16 | 8 | 165 | purine ribonucleotide biosynthetic process |
| GO:0006812 | 0.013 | 0.555 | 25 | 15 | 253 | cation transport |
| GO:0007586 | 0.014 | 0.239 | 8 | 2 | 77 | digestion |
| GO:0090257 | 0.014 | 0.348 | 11 | 4 | 107 | regulation of muscle system process |
| GO:0006875 | 0.016 | 0.566 | 25 | 15 | 251 | cellular metal ion homeostasis |
| GO:0000165 | 0.018 | 0.657 | 40 | 28 | 406 | MAPK cascade |
| GO:0008361 | 0.018 | 0.160 | 6 | 1 | 57 | regulation of cell size |
| GO:0044281 | 0.018 | 0.817 | 170 | 147 | 1708 | small molecule metabolic process |
| GO:0007270 | 0.019 | 0.253 | 7 | 2 | 73 | neuron-neuron synaptic transmission |
| GO:0051056 | 0.019 | 0.565 | 23 | 14 | 235 | regulation of small GTPase mediated signal transduction |
| GO:0009306 | 0.019 | 0.162 | 6 | 1 | 56 | protein secretion |
| GO:0051607 | 0.019 | 0.429 | 13 | 6 | 131 | defense response to virus |
| GO:0033124 | 0.020 | 0.457 | 14 | 7 | 144 | regulation of GTP catabolic process |
| GO:0071453 | 0.021 | 0.256 | 7 | 2 | 72 | cellular response to oxygen levels |
| GO:0030335 | 0.023 | 0.537 | 19 | 11 | 194 | positive regulation of cell migration |
| GO:0002708 | 0.023 | <0.0001 | 4 | 0 | 36 | positive regulation of lymphocyte mediated immunity |
| GO:0006940 | 0.023 | <0.0001 | 4 | 0 | 36 | regulation of smooth muscle contraction |
| GO:0030195 | 0.023 | <0.0001 | 4 | 0 | 36 | negative regulation of blood coagulation |
| GO:0051238 | 0.023 | <0.0001 | 4 | 0 | 36 | sequestering of metal ion |
| GO:0008015 | 0.023 | 0.440 | 13 | 6 | 126 | blood circulation |
| GO:0007588 | 0.024 | 0.169 | 5 | 1 | 54 | excretion |
| GO:0060349 | 0.024 | 0.169 | 5 | 1 | 54 | bone morphogenesis |
| GO:0007254 | 0.024 | 0.411 | 11 | 5 | 114 | JNK cascade |
| GO:0002824 | 0.025 | <0.0001 | 3 | 0 | 35 | positive regulation of adaptive immune response based on somatic recombination of immune receptors built from immunoglobulin superfamily domains |
| GO:0003044 | 0.025 | <0.0001 | 3 | 0 | 35 | regulation of systemic arterial blood pressure mediated by a chemical signal |
| GO:0032890 | 0.025 | <0.0001 | 3 | 0 | 35 | regulation of organic acid transport |
| GO:0001819 | 0.026 | 0.493 | 15 | 8 | 153 | positive regulation of cytokine production |
| GO:0051272 | 0.026 | 0.558 | 20 | 12 | 204 | positive regulation of cellular component movement |
| GO:0060048 | 0.026 | 0.173 | 5 | 1 | 53 | cardiac muscle contraction |
| GO:0001894 | 0.026 | 0.332 | 8 | 3 | 84 | tissue homeostasis |
| GO:0002682 | 0.027 | 0.730 | 58 | 44 | 573 | regulation of immune system process |
| GO:0001974 | 0.028 | <0.0001 | 3 | 0 | 34 | blood vessel remodeling |
| GO:0002718 | 0.028 | <0.0001 | 3 | 0 | 34 | regulation of cytokine production involved in immune response |
| GO:0031645 | 0.028 | <0.0001 | 3 | 0 | 34 | negative regulation of neurological system process |
| GO:0048168 | 0.028 | <0.0001 | 3 | 0 | 34 | regulation of neuronal synaptic plasticity |
| GO:0050764 | 0.028 | <0.0001 | 3 | 0 | 34 | regulation of phagocytosis |
| GO:0001817 | 0.028 | 0.421 | 11 | 5 | 110 | regulation of cytokine production |
| GO:0045807 | 0.028 | 0.176 | 5 | 1 | 52 | positive regulation of endocytosis |
| GO:0050880 | 0.029 | 0.386 | 10 | 4 | 97 | regulation of blood vessel size |
| GO:0046390 | 0.030 | 0.539 | 18 | 10 | 176 | ribose phosphate biosynthetic process |
| GO:0050714 | 0.031 | 0.276 | 7 | 2 | 67 | positive regulation of protein secretion |
| GO:0006898 | 0.031 | 0.427 | 11 | 5 | 110 | receptor-mediated endocytosis |
| GO:0051707 | 0.031 | 0.694 | 41 | 30 | 414 | response to other organism |
| GO:0043270 | 0.031 | 0.390 | 10 | 4 | 96 | positive regulation of ion transport |
| GO:0071456 | 0.033 | 0.281 | 7 | 2 | 66 | cellular response to hypoxia |
| GO:0030595 | 0.034 | <0.0001 | 3 | 0 | 32 | leukocyte chemotaxis |
| GO:0009205 | 0.034 | 0.660 | 32 | 22 | 319 | purine ribonucleoside triphosphate metabolic process |
| GO:0032609 | 0.034 | 0.183 | 5 | 1 | 50 | interferon-gamma production |
| GO:0046849 | 0.034 | 0.183 | 5 | 1 | 50 | bone remodeling |
| GO:0007601 | 0.034 | 0.490 | 13 | 7 | 135 | visual perception |
| GO:0045762 | 0.035 | <0.0001 | 3 | 0 | 32 | positive regulation of adenylate cyclase activity |
| GO:0048583 | 0.036 | 0.836 | 160 | 140 | 1602 | regulation of response to stimulus |
| GO:0036293 | 0.036 | 0.565 | 18 | 11 | 185 | response to decreased oxygen levels |
| GO:0007204 | 0.036 | 0.494 | 13 | 7 | 134 | elevation of cytosolic calcium ion concentration |
| GO:0035023 | 0.037 | 0.439 | 11 | 5 | 107 | regulation of Rho protein signal transduction |
| GO:0006820 | 0.037 | 0.604 | 22 | 14 | 221 | anion transport |
| GO:0032844 | 0.037 | 0.604 | 22 | 14 | 221 | regulation of homeostatic process |
| GO:0043620 | 0.038 | <0.0001 | 3 | 0 | 31 | regulation of DNA-dependent transcription in response to stress |
| GO:0009154 | 0.039 | 0.656 | 29 | 20 | 292 | purine ribonucleotide catabolic process |
| GO:0006935 | 0.039 | 0.727 | 47 | 36 | 476 | chemotaxis |
| GO:0002479 | 0.039 | 0.290 | 6 | 2 | 64 | antigen processing and presentation of exogenous peptide antigen via MHC class I, TAP-dependent |
| GO:0070374 | 0.039 | 0.290 | 6 | 2 | 64 | positive regulation of ERK1 and ERK2 cascade |
| GO:0046942 | 0.040 | 0.541 | 16 | 9 | 158 | carboxylic acid transport |
| GO:0070838 | 0.040 | 0.572 | 18 | 11 | 183 | divalent metal ion transport |
| GO:0002768 | 0.040 | 0.444 | 11 | 5 | 106 | immune response-regulating cell surface receptor signaling pathway |
| GO:0015672 | 0.040 | 0.586 | 19 | 12 | 195 | monovalent inorganic cation transport |
| GO:0051971 | 0.041 | 0.191 | 5 | 1 | 48 | positive regulation of transmission of nerve impulse |
| GO:0072503 | 0.041 | 0.599 | 21 | 13 | 207 | cellular divalent inorganic cation homeostasis |
| GO:0031349 | 0.042 | 0.575 | 18 | 11 | 182 | positive regulation of defense response |
| GO:0030282 | 0.042 | 0.295 | 6 | 2 | 63 | bone mineralization |
| GO:0006493 | 0.043 | <0.0001 | 3 | 0 | 30 | protein O-linked glycosylation |
| GO:0022617 | 0.043 | <0.0001 | 3 | 0 | 30 | extracellular matrix disassembly |
| GO:0072676 | 0.043 | <0.0001 | 3 | 0 | 30 | lymphocyte migration |
| GO:0003015 | 0.043 | 0.480 | 12 | 6 | 118 | heart process |
| GO:0008277 | 0.044 | 0.364 | 8 | 3 | 77 | regulation of G-protein coupled receptor protein signaling pathway |
| GO:0048520 | 0.044 | 0.364 | 8 | 3 | 77 | positive regulation of behavior |
| GO:0051952 | 0.044 | 0.195 | 5 | 1 | 47 | regulation of amine transport |
| GO:0048872 | 0.045 | 0.531 | 14 | 8 | 143 | homeostasis of number of cells |
| GO:0007600 | 0.045 | 0.666 | 29 | 20 | 288 | sensory perception |
| GO:0042454 | 0.045 | 0.666 | 29 | 20 | 288 | ribonucleoside catabolic process |
| GO:0048588 | 0.046 | 0.299 | 6 | 2 | 62 | developmental cell growth |
| GO:0071478 | 0.046 | 0.299 | 6 | 2 | 62 | cellular response to radiation |
| GO:0002253 | 0.047 | 0.620 | 22 | 14 | 216 | activation of immune response |
| GO:0006839 | 0.047 | 0.369 | 8 | 3 | 76 | mitochondrial transport |
| GO:0061041 | 0.047 | 0.369 | 8 | 3 | 76 | regulation of wound healing |
| GO:0001910 | 0.047 | <0.0001 | 3 | 0 | 29 | regulation of leukocyte mediated cytotoxicity |
| GO:0002709 | 0.047 | <0.0001 | 3 | 0 | 29 | regulation of T cell mediated immunity |
| GO:0010923 | 0.047 | <0.0001 | 3 | 0 | 29 | negative regulation of phosphatase activity |
| GO:0032637 | 0.047 | <0.0001 | 3 | 0 | 29 | interleukin-8 production |
| GO:0030593 | 0.048 | 0.200 | 5 | 1 | 46 | neutrophil chemotaxis |
| GO:0034340 | 0.049 | 0.305 | 6 | 2 | 61 | response to type I interferon |
| GO:0060337 | 0.049 | 0.305 | 6 | 2 | 61 | type I interferon-mediated signaling pathway |
| GO:0060191 | 0.050 | 0.422 | 9 | 4 | 89 | regulation of lipase activity |
