# Supplementary material for: Hidden Treasures in “Ancient” Microarrays: Gene-Expression Portrays Biology and Potential Resistance Pathways of Major Lung Cancer Subtypes and Normal Tissue
Source: Front Oncol. 2014 Sep 29;4:251. doi: 10.3389/fonc.2014.00251 (PMC4178426; doi:10.3389/fonc.2014.00251)
Supplement: Figure S1 — Clinically relevant differentially expressed pathways in each tissue type versus the rest. The pathway maps are arranged according to the scheme in the left-top corner. Red indicates overexpression in genes, dark green downregulation. In the Cell Cycle pathway, tumor suppressor genes were overexpressed in normal lung tissue while oncogenes and tumor driving cyclins were overexpressed in cancers. The gene thymidylate synthase (TYMS or TS, belonging to the pathway “one carbon pool by folate”) is relevant for tumor growth and is also a treatment target. Notably, TYMS was overexpressed only in the tumors that are generally refractory to the drug pemetrexed, as the squamous and the small-cell lung cancer. TYMS was not overexpressed in mesothelioma, but it is known that TYMS expression is highly variable in this cancer. In the ERBB pathway, the ERBB2/HER2 and ERBB3/HER3 were overexpressed in adenocarcinoma, while the ERBB4 was overexpressed in the carcinoids. [file Data_Sheet_1.ZIP › File S3.ZIP!/GO Analysis/Small Cell vs Rest Enrichment Analysis/Underrepresented GO terms of Down-regulated Genes.html]

Gene to GO BP Conditional test for under-representation

| GOBPID | Pvalue | OddsRatio | ExpCount | Count | Size | Term |
| --- | --- | --- | --- | --- | --- | --- |
| GO:0051252 | 0.001 | 0.610 | 71 | 48 | 1680 | regulation of RNA metabolic process |
| GO:0090304 | 0.001 | 0.674 | 105 | 80 | 2488 | nucleic acid metabolic process |
| GO:0032774 | 0.001 | 0.642 | 79 | 56 | 1862 | RNA biosynthetic process |
| GO:0051171 | 0.002 | 0.668 | 88 | 65 | 2072 | regulation of nitrogen compound metabolic process |
| GO:0002520 | 0.003 | 0.402 | 19 | 8 | 443 | immune system development |
| GO:0007267 | 0.004 | 0.546 | 34 | 20 | 810 | cell-cell signaling |
| GO:0051276 | 0.004 | 0.407 | 19 | 8 | 438 | chromosome organization |
| GO:0010556 | 0.004 | 0.677 | 78 | 58 | 1848 | regulation of macromolecule biosynthetic process |
| GO:0008217 | 0.006 | <0.0001 | 5 | 0 | 118 | regulation of blood pressure |
| GO:1901362 | 0.006 | 0.712 | 93 | 73 | 2205 | organic cyclic compound biosynthetic process |
| GO:0019438 | 0.006 | 0.711 | 90 | 70 | 2123 | aromatic compound biosynthetic process |
| GO:0044271 | 0.007 | 0.715 | 92 | 72 | 2170 | cellular nitrogen compound biosynthetic process |
| GO:0031326 | 0.007 | 0.703 | 80 | 61 | 1864 | regulation of cellular biosynthetic process |
| GO:0006935 | 0.007 | 0.470 | 20 | 10 | 476 | chemotaxis |
| GO:0048666 | 0.008 | 0.526 | 25 | 14 | 595 | neuron development |
| GO:0030595 | 0.009 | <0.0001 | 5 | 0 | 108 | leukocyte chemotaxis |
| GO:0018130 | 0.009 | 0.725 | 90 | 71 | 2123 | heterocycle biosynthetic process |
| GO:0000375 | 0.010 | 0.145 | 7 | 1 | 154 | RNA splicing, via transesterification reactions |
| GO:0030154 | 0.010 | 0.711 | 75 | 58 | 1783 | cell differentiation |
| GO:0007283 | 0.011 | 0.236 | 8 | 2 | 190 | spermatogenesis |
| GO:0000398 | 0.012 | 0.150 | 6 | 1 | 149 | mRNA splicing, via spliceosome |
| GO:0030097 | 0.012 | 0.455 | 17 | 8 | 395 | hemopoiesis |
| GO:0030308 | 0.012 | <0.0001 | 4 | 0 | 102 | negative regulation of cell growth |
| GO:0032844 | 0.014 | 0.305 | 9 | 3 | 221 | regulation of homeostatic process |
| GO:0048872 | 0.015 | 0.157 | 6 | 1 | 143 | homeostasis of number of cells |
| GO:0051321 | 0.016 | <0.0001 | 4 | 0 | 95 | meiotic cell cycle |
| GO:0052652 | 0.016 | <0.0001 | 4 | 0 | 95 | cyclic purine nucleotide metabolic process |
| GO:0006355 | 0.017 | 0.617 | 32 | 21 | 731 | regulation of transcription, DNA-dependent |
| GO:1900542 | 0.017 | 0.362 | 11 | 4 | 249 | regulation of purine nucleotide metabolic process |
| GO:0046058 | 0.018 | <0.0001 | 4 | 0 | 93 | cAMP metabolic process |
| GO:0000279 | 0.018 | 0.429 | 13 | 6 | 315 | M phase |
| GO:0010467 | 0.019 | 0.765 | 106 | 88 | 2497 | gene expression |
| GO:0007126 | 0.019 | <0.0001 | 4 | 0 | 91 | meiosis |
| GO:0030799 | 0.020 | <0.0001 | 4 | 0 | 90 | regulation of cyclic nucleotide metabolic process |
| GO:0006357 | 0.020 | 0.654 | 38 | 26 | 891 | regulation of transcription from RNA polymerase II promoter |
| GO:0007186 | 0.021 | 0.464 | 14 | 7 | 337 | G-protein coupled receptor signaling pathway |
| GO:0007050 | 0.021 | 0.325 | 9 | 3 | 206 | cell cycle arrest |
| GO:0007423 | 0.023 | 0.443 | 13 | 6 | 306 | sensory organ development |
| GO:0032501 | 0.025 | 0.795 | 155 | 137 | 3664 | multicellular organismal process |
| GO:0007399 | 0.025 | 0.706 | 51 | 38 | 1201 | nervous system development |
| GO:0044057 | 0.025 | 0.497 | 15 | 8 | 364 | regulation of system process |
| GO:0030808 | 0.026 | <0.0001 | 4 | 0 | 84 | regulation of nucleotide biosynthetic process |
| GO:0010975 | 0.028 | 0.275 | 7 | 2 | 164 | regulation of neuron projection development |
| GO:0050767 | 0.029 | 0.459 | 13 | 6 | 296 | regulation of neurogenesis |
| GO:0051726 | 0.030 | 0.392 | 10 | 4 | 224 | regulation of cell cycle |
| GO:0003013 | 0.030 | 0.460 | 12 | 6 | 295 | circulatory system process |
| GO:2000602 | 0.031 | 0.183 | 5 | 1 | 123 | regulation of interphase of mitotic cell cycle |
| GO:0000209 | 0.033 | 0.186 | 5 | 1 | 121 | protein polyubiquitination |
| GO:0051438 | 0.034 | <0.0001 | 3 | 0 | 78 | regulation of ubiquitin-protein ligase activity |
| GO:0008277 | 0.035 | <0.0001 | 3 | 0 | 77 | regulation of G-protein coupled receptor protein signaling pathway |
| GO:0055085 | 0.036 | 0.569 | 19 | 11 | 438 | transmembrane transport |
| GO:0030817 | 0.037 | <0.0001 | 3 | 0 | 76 | regulation of cAMP biosynthetic process |
| GO:0002685 | 0.039 | <0.0001 | 3 | 0 | 75 | regulation of leukocyte migration |
| GO:0006323 | 0.039 | <0.0001 | 3 | 0 | 75 | DNA packaging |
| GO:0010564 | 0.039 | 0.361 | 8 | 3 | 186 | regulation of cell cycle process |
| GO:0048812 | 0.041 | 0.578 | 18 | 11 | 432 | neuron projection morphogenesis |
| GO:0030218 | 0.042 | <0.0001 | 3 | 0 | 73 | erythrocyte differentiation |
| GO:0016568 | 0.043 | 0.485 | 12 | 6 | 281 | chromatin modification |
| GO:0034645 | 0.044 | 0.799 | 99 | 85 | 2348 | cellular macromolecule biosynthetic process |
| GO:0030030 | 0.045 | 0.660 | 28 | 19 | 652 | cell projection organization |
| GO:0071158 | 0.046 | <0.0001 | 3 | 0 | 71 | positive regulation of cell cycle arrest |
| GO:0032990 | 0.047 | 0.613 | 20 | 13 | 482 | cell part morphogenesis |
| GO:0000216 | 0.048 | <0.0001 | 3 | 0 | 70 | M/G1 transition of mitotic cell cycle |
| GO:0006333 | 0.048 | <0.0001 | 3 | 0 | 70 | chromatin assembly or disassembly |
| GO:0031145 | 0.048 | <0.0001 | 3 | 0 | 70 | anaphase-promoting complex-dependent proteasomal ubiquitin-dependent protein catabolic process |
| GO:0065004 | 0.048 | <0.0001 | 3 | 0 | 70 | protein-DNA complex assembly |
| GO:0071826 | 0.048 | <0.0001 | 3 | 0 | 70 | ribonucleoprotein complex subunit organization |
| GO:0007093 | 0.049 | 0.205 | 5 | 1 | 110 | mitotic cell cycle checkpoint |
