# Supplementary material for: Hidden Treasures in “Ancient” Microarrays: Gene-Expression Portrays Biology and Potential Resistance Pathways of Major Lung Cancer Subtypes and Normal Tissue
Source: Front Oncol. 2014 Sep 29;4:251. doi: 10.3389/fonc.2014.00251 (PMC4178426; doi:10.3389/fonc.2014.00251)
Supplement: Figure S1 — Clinically relevant differentially expressed pathways in each tissue type versus the rest. The pathway maps are arranged according to the scheme in the left-top corner. Red indicates overexpression in genes, dark green downregulation. In the Cell Cycle pathway, tumor suppressor genes were overexpressed in normal lung tissue while oncogenes and tumor driving cyclins were overexpressed in cancers. The gene thymidylate synthase (TYMS or TS, belonging to the pathway “one carbon pool by folate”) is relevant for tumor growth and is also a treatment target. Notably, TYMS was overexpressed only in the tumors that are generally refractory to the drug pemetrexed, as the squamous and the small-cell lung cancer. TYMS was not overexpressed in mesothelioma, but it is known that TYMS expression is highly variable in this cancer. In the ERBB pathway, the ERBB2/HER2 and ERBB3/HER3 were overexpressed in adenocarcinoma, while the ERBB4 was overexpressed in the carcinoids. [file Data_Sheet_1.ZIP › File S3.ZIP!/GO Analysis/Small Cell vs Rest Enrichment Analysis/Underrepresented GO terms of Up-regulated Genes.html]

Gene to GO BP Conditional test for under-representation

| GOBPID | Pvalue | OddsRatio | ExpCount | Count | Size | Term |
| --- | --- | --- | --- | --- | --- | --- |
| GO:0016192 | <0.0001 | 0.284 | 35 | 11 | 611 | vesicle-mediated transport |
| GO:0044281 | <0.0001 | 0.480 | 47 | 25 | 785 | small molecule metabolic process |
| GO:0051234 | <0.0001 | 0.644 | 125 | 91 | 2181 | establishment of localization |
| GO:0030198 | <0.0001 | <0.0001 | 9 | 0 | 153 | extracellular matrix organization |
| GO:0008015 | <0.0001 | 0.276 | 17 | 5 | 294 | blood circulation |
| GO:1901564 | 0.001 | 0.579 | 59 | 37 | 1015 | organonitrogen compound metabolic process |
| GO:0043436 | 0.001 | 0.494 | 36 | 19 | 625 | oxoacid metabolic process |
| GO:0015849 | 0.001 | 0.103 | 9 | 1 | 158 | organic acid transport |
| GO:0006140 | 0.001 | 0.260 | 14 | 4 | 251 | regulation of nucleotide metabolic process |
| GO:0044057 | 0.001 | 0.172 | 11 | 2 | 186 | regulation of system process |
| GO:0006812 | 0.001 | 0.438 | 26 | 12 | 448 | cation transport |
| GO:0032787 | 0.002 | 0.309 | 15 | 5 | 265 | monocarboxylic acid metabolic process |
| GO:0010740 | 0.002 | 0.406 | 21 | 9 | 363 | positive regulation of intracellular protein kinase cascade |
| GO:0051346 | 0.002 | 0.188 | 10 | 2 | 174 | negative regulation of hydrolase activity |
| GO:0010647 | 0.002 | 0.541 | 36 | 21 | 635 | positive regulation of cell communication |
| GO:0023056 | 0.002 | 0.541 | 36 | 21 | 635 | positive regulation of signaling |
| GO:0009410 | 0.003 | <0.0001 | 6 | 0 | 100 | response to xenobiotic stimulus |
| GO:0009607 | 0.003 | 0.460 | 25 | 12 | 428 | response to biotic stimulus |
| GO:0023052 | 0.003 | 0.751 | 180 | 152 | 3144 | signaling |
| GO:0006805 | 0.003 | <0.0001 | 6 | 0 | 98 | xenobiotic metabolic process |
| GO:0005975 | 0.003 | 0.498 | 28 | 15 | 494 | carbohydrate metabolic process |
| GO:0072594 | 0.003 | 0.121 | 8 | 1 | 135 | establishment of protein localization to organelle |
| GO:0070482 | 0.003 | 0.251 | 11 | 3 | 196 | response to oxygen levels |
| GO:0006022 | 0.003 | <0.0001 | 6 | 0 | 97 | aminoglycan metabolic process |
| GO:0009152 | 0.003 | 0.198 | 9 | 2 | 165 | purine ribonucleotide biosynthetic process |
| GO:0018212 | 0.003 | 0.198 | 9 | 2 | 165 | peptidyl-tyrosine modification |
| GO:0006954 | 0.004 | 0.256 | 11 | 3 | 188 | inflammatory response |
| GO:0015711 | 0.004 | 0.257 | 11 | 3 | 191 | organic anion transport |
| GO:0034220 | 0.005 | 0.306 | 12 | 4 | 215 | ion transmembrane transport |
| GO:0002526 | 0.005 | <0.0001 | 5 | 0 | 89 | acute inflammatory response |
| GO:0008202 | 0.005 | 0.309 | 12 | 4 | 213 | steroid metabolic process |
| GO:0006955 | 0.005 | 0.547 | 31 | 18 | 527 | immune response |
| GO:0001666 | 0.005 | 0.268 | 11 | 3 | 184 | response to hypoxia |
| GO:0015031 | 0.006 | 0.378 | 15 | 6 | 253 | protein transport |
| GO:0001819 | 0.006 | 0.214 | 9 | 2 | 153 | positive regulation of cytokine production |
| GO:0051056 | 0.006 | 0.350 | 13 | 5 | 235 | regulation of small GTPase mediated signal transduction |
| GO:0072522 | 0.006 | 0.272 | 10 | 3 | 181 | purine-containing compound biosynthetic process |
| GO:0051241 | 0.006 | 0.272 | 10 | 3 | 179 | negative regulation of multicellular organismal process |
| GO:0042278 | 0.007 | 0.465 | 20 | 10 | 355 | purine nucleoside metabolic process |
| GO:0030802 | 0.007 | <0.0001 | 5 | 0 | 84 | regulation of cyclic nucleotide biosynthetic process |
| GO:1900371 | 0.007 | <0.0001 | 5 | 0 | 84 | regulation of purine nucleotide biosynthetic process |
| GO:0031644 | 0.007 | 0.277 | 10 | 3 | 178 | regulation of neurological system process |
| GO:0043408 | 0.008 | 0.470 | 20 | 10 | 351 | regulation of MAPK cascade |
| GO:0046390 | 0.008 | 0.280 | 10 | 3 | 176 | ribose phosphate biosynthetic process |
| GO:0030334 | 0.008 | 0.458 | 19 | 9 | 325 | regulation of cell migration |
| GO:0055114 | 0.008 | 0.439 | 17 | 8 | 298 | oxidation-reduction process |
| GO:0016054 | 0.008 | 0.225 | 8 | 2 | 146 | organic acid catabolic process |
| GO:0070887 | 0.008 | 0.708 | 75 | 57 | 1315 | cellular response to chemical stimulus |
| GO:0050804 | 0.009 | 0.227 | 8 | 2 | 145 | regulation of synaptic transmission |
| GO:0050778 | 0.009 | 0.227 | 8 | 2 | 143 | positive regulation of immune response |
| GO:0009119 | 0.009 | 0.494 | 21 | 11 | 368 | ribonucleoside metabolic process |
| GO:0007169 | 0.010 | 0.562 | 29 | 17 | 500 | transmembrane receptor protein tyrosine kinase signaling pathway |
| GO:2000147 | 0.010 | 0.333 | 11 | 4 | 198 | positive regulation of cell motility |
| GO:0051704 | 0.010 | 0.683 | 59 | 43 | 1034 | multi-organism process |
| GO:0030814 | 0.010 | <0.0001 | 4 | 0 | 78 | regulation of cAMP metabolic process |
| GO:0090066 | 0.010 | 0.370 | 13 | 5 | 223 | regulation of anatomical structure size |
| GO:0044255 | 0.010 | 0.499 | 21 | 11 | 359 | cellular lipid metabolic process |
| GO:0007186 | 0.010 | 0.525 | 24 | 13 | 410 | G-protein coupled receptor signaling pathway |
| GO:1901657 | 0.010 | 0.525 | 24 | 13 | 410 | glycosyl compound metabolic process |
| GO:0002576 | 0.010 | <0.0001 | 4 | 0 | 77 | platelet degranulation |
| GO:0045834 | 0.010 | <0.0001 | 4 | 0 | 77 | positive regulation of lipid metabolic process |
| GO:0007267 | 0.010 | 0.652 | 46 | 32 | 810 | cell-cell signaling |
| GO:0002697 | 0.011 | 0.292 | 10 | 3 | 169 | regulation of immune effector process |
| GO:0043406 | 0.011 | 0.235 | 8 | 2 | 140 | positive regulation of MAP kinase activity |
| GO:0006898 | 0.011 | 0.149 | 6 | 1 | 110 | receptor-mediated endocytosis |
| GO:0008104 | 0.012 | 0.690 | 59 | 43 | 1025 | protein localization |
| GO:0006869 | 0.012 | 0.238 | 8 | 2 | 138 | lipid transport |
| GO:0030595 | 0.012 | 0.152 | 6 | 1 | 108 | leukocyte chemotaxis |
| GO:0032940 | 0.013 | 0.584 | 29 | 18 | 511 | secretion by cell |
| GO:0044242 | 0.013 | 0.153 | 6 | 1 | 107 | cellular lipid catabolic process |
| GO:0042742 | 0.013 | <0.0001 | 4 | 0 | 73 | defense response to bacterium |
| GO:0030168 | 0.013 | 0.348 | 11 | 4 | 190 | platelet activation |
| GO:0044712 | 0.013 | 0.348 | 11 | 4 | 190 | single-organism catabolic process |
| GO:0030258 | 0.014 | 0.155 | 6 | 1 | 106 | lipid modification |
| GO:0015718 | 0.014 | <0.0001 | 4 | 0 | 72 | monocarboxylic acid transport |
| GO:0048771 | 0.016 | 0.159 | 6 | 1 | 103 | tissue remodeling |
| GO:0007155 | 0.016 | 0.623 | 34 | 22 | 580 | cell adhesion |
| GO:0006811 | 0.016 | 0.311 | 9 | 3 | 153 | ion transport |
| GO:0006909 | 0.017 | <0.0001 | 4 | 0 | 69 | phagocytosis |
| GO:0032318 | 0.017 | 0.161 | 6 | 1 | 102 | regulation of Ras GTPase activity |
| GO:0003012 | 0.017 | 0.427 | 13 | 6 | 233 | muscle system process |
| GO:0046129 | 0.018 | <0.0001 | 4 | 0 | 68 | purine ribonucleoside biosynthetic process |
| GO:0043122 | 0.018 | 0.254 | 7 | 2 | 130 | regulation of I-kappaB kinase/NF-kappaB cascade |
| GO:0046165 | 0.018 | 0.163 | 6 | 1 | 101 | alcohol biosynthetic process |
| GO:0050731 | 0.018 | 0.163 | 6 | 1 | 101 | positive regulation of peptidyl-tyrosine phosphorylation |
| GO:0006886 | 0.018 | 0.601 | 29 | 18 | 498 | intracellular protein transport |
| GO:1901068 | 0.018 | 0.431 | 13 | 6 | 231 | guanosine-containing compound metabolic process |
| GO:0019395 | 0.019 | <0.0001 | 4 | 0 | 67 | fatty acid oxidation |
| GO:0035303 | 0.019 | <0.0001 | 4 | 0 | 67 | regulation of dephosphorylation |
| GO:0048584 | 0.019 | 0.637 | 34 | 23 | 584 | positive regulation of response to stimulus |
| GO:0045785 | 0.020 | 0.166 | 6 | 1 | 99 | positive regulation of cell adhesion |
| GO:0071214 | 0.021 | 0.260 | 7 | 2 | 127 | cellular response to abiotic stimulus |
| GO:0007165 | 0.021 | 0.735 | 70 | 55 | 1150 | signal transduction |
| GO:0006875 | 0.021 | 0.463 | 14 | 7 | 251 | cellular metal ion homeostasis |
| GO:0009205 | 0.021 | 0.522 | 18 | 10 | 319 | purine ribonucleoside triphosphate metabolic process |
| GO:0002757 | 0.022 | 0.375 | 10 | 4 | 177 | immune response-activating signal transduction |
| GO:0045089 | 0.022 | 0.264 | 7 | 2 | 125 | positive regulation of innate immune response |
| GO:0002479 | 0.023 | <0.0001 | 4 | 0 | 64 | antigen processing and presentation of exogenous peptide antigen via MHC class I, TAP-dependent |
| GO:0006024 | 0.023 | <0.0001 | 4 | 0 | 64 | glycosaminoglycan biosynthetic process |
| GO:0006415 | 0.023 | <0.0001 | 4 | 0 | 64 | translational termination |
| GO:0070374 | 0.023 | <0.0001 | 4 | 0 | 64 | positive regulation of ERK1 and ERK2 cascade |
| GO:0002274 | 0.023 | 0.171 | 6 | 1 | 96 | myeloid leukocyte activation |
| GO:0044765 | 0.024 | 0.717 | 56 | 42 | 900 | single-organism transport |
| GO:0051270 | 0.024 | 0.568 | 22 | 13 | 381 | regulation of cellular component movement |
| GO:0052547 | 0.024 | 0.415 | 11 | 5 | 200 | regulation of peptidase activity |
| GO:0001818 | 0.024 | 0.173 | 5 | 1 | 95 | negative regulation of cytokine production |
| GO:0022900 | 0.026 | 0.175 | 5 | 1 | 94 | electron transport chain |
| GO:0009615 | 0.026 | 0.420 | 11 | 5 | 198 | response to virus |
| GO:0006937 | 0.027 | 0.177 | 5 | 1 | 93 | regulation of muscle contraction |
| GO:0050708 | 0.027 | 0.177 | 5 | 1 | 93 | regulation of protein secretion |
| GO:0018279 | 0.027 | <0.0001 | 3 | 0 | 61 | protein N-linked glycosylation via asparagine |
| GO:0034340 | 0.027 | <0.0001 | 3 | 0 | 61 | response to type I interferon |
| GO:0050729 | 0.027 | <0.0001 | 3 | 0 | 61 | positive regulation of inflammatory response |
| GO:0060337 | 0.027 | <0.0001 | 3 | 0 | 61 | type I interferon-mediated signaling pathway |
| GO:1900544 | 0.027 | <0.0001 | 3 | 0 | 61 | positive regulation of purine nucleotide metabolic process |
| GO:0019083 | 0.027 | 0.273 | 7 | 2 | 121 | viral transcription |
| GO:0033674 | 0.027 | 0.551 | 19 | 11 | 333 | positive regulation of kinase activity |
| GO:0070085 | 0.027 | 0.338 | 8 | 3 | 147 | glycosylation |
| GO:0006184 | 0.027 | 0.454 | 13 | 6 | 220 | GTP catabolic process |
| GO:0008203 | 0.028 | 0.179 | 5 | 1 | 92 | cholesterol metabolic process |
| GO:0006486 | 0.028 | 0.341 | 8 | 3 | 146 | protein glycosylation |
| GO:0006650 | 0.028 | 0.341 | 8 | 3 | 146 | glycerophospholipid metabolic process |
| GO:0030801 | 0.029 | <0.0001 | 3 | 0 | 60 | positive regulation of cyclic nucleotide metabolic process |
| GO:0050727 | 0.029 | 0.179 | 5 | 1 | 91 | regulation of inflammatory response |
| GO:0009725 | 0.029 | 0.648 | 31 | 21 | 541 | response to hormone stimulus |
| GO:0006917 | 0.030 | 0.343 | 8 | 3 | 145 | induction of apoptosis |
| GO:0003015 | 0.031 | 0.280 | 7 | 2 | 118 | heart process |
| GO:0006865 | 0.031 | 0.183 | 5 | 1 | 90 | amino acid transport |
| GO:0002687 | 0.032 | <0.0001 | 3 | 0 | 58 | positive regulation of leukocyte migration |
| GO:0003073 | 0.032 | <0.0001 | 3 | 0 | 58 | regulation of systemic arterial blood pressure |
| GO:0030810 | 0.032 | <0.0001 | 3 | 0 | 58 | positive regulation of nucleotide biosynthetic process |
| GO:0055080 | 0.032 | 0.511 | 15 | 8 | 259 | cation homeostasis |
| GO:0016044 | 0.032 | 0.531 | 16 | 9 | 283 | cellular membrane organization |
| GO:0060191 | 0.033 | 0.185 | 5 | 1 | 89 | regulation of lipase activity |
| GO:0048878 | 0.033 | 0.681 | 37 | 26 | 637 | chemical homeostasis |
| GO:0006066 | 0.033 | 0.285 | 7 | 2 | 115 | alcohol metabolic process |
| GO:0043269 | 0.034 | 0.467 | 12 | 6 | 214 | regulation of ion transport |
| GO:0000041 | 0.034 | <0.0001 | 3 | 0 | 57 | transition metal ion transport |
| GO:0009056 | 0.034 | 0.186 | 5 | 1 | 85 | catabolic process |
| GO:0070972 | 0.035 | 0.187 | 5 | 1 | 88 | protein localization to endoplasmic reticulum |
| GO:0009100 | 0.035 | 0.470 | 12 | 6 | 213 | glycoprotein metabolic process |
| GO:0009118 | 0.036 | 0.403 | 9 | 4 | 165 | regulation of nucleoside metabolic process |
| GO:0033121 | 0.036 | 0.403 | 9 | 4 | 165 | regulation of purine nucleotide catabolic process |
| GO:0042180 | 0.036 | 0.403 | 9 | 4 | 165 | cellular ketone metabolic process |
| GO:0030193 | 0.036 | <0.0001 | 3 | 0 | 56 | regulation of blood coagulation |
| GO:0045621 | 0.036 | <0.0001 | 3 | 0 | 56 | positive regulation of lymphocyte differentiation |
| GO:0055072 | 0.036 | <0.0001 | 3 | 0 | 56 | iron ion homeostasis |
| GO:0003018 | 0.037 | 0.290 | 7 | 2 | 114 | vascular process in circulatory system |
| GO:0007254 | 0.037 | 0.290 | 7 | 2 | 114 | JNK cascade |
| GO:0040012 | 0.038 | 0.597 | 21 | 13 | 364 | regulation of locomotion |
| GO:0072523 | 0.038 | 0.573 | 18 | 11 | 321 | purine-containing compound catabolic process |
| GO:0072376 | 0.038 | <0.0001 | 3 | 0 | 55 | protein activation cascade |
| GO:1901575 | 0.038 | 0.771 | 72 | 58 | 1249 | organic substance catabolic process |
| GO:0006413 | 0.039 | 0.293 | 6 | 2 | 113 | translational initiation |
| GO:0007166 | 0.039 | 0.774 | 74 | 60 | 1254 | cell surface receptor signaling pathway |
| GO:0019693 | 0.040 | 0.561 | 17 | 10 | 293 | ribose phosphate metabolic process |
| GO:0007588 | 0.041 | <0.0001 | 3 | 0 | 54 | excretion |
| GO:0019217 | 0.041 | <0.0001 | 3 | 0 | 54 | regulation of fatty acid metabolic process |
| GO:0030819 | 0.041 | <0.0001 | 3 | 0 | 54 | positive regulation of cAMP biosynthetic process |
| GO:0051174 | 0.041 | 0.741 | 52 | 40 | 901 | regulation of phosphorus metabolic process |
| GO:0000184 | 0.042 | 0.196 | 5 | 1 | 84 | nuclear-transcribed mRNA catabolic process, nonsense-mediated decay |
| GO:0002474 | 0.042 | 0.196 | 5 | 1 | 84 | antigen processing and presentation of peptide antigen via MHC class I |
| GO:0009150 | 0.042 | 0.565 | 17 | 10 | 291 | purine ribonucleotide metabolic process |
| GO:0055086 | 0.043 | 0.692 | 35 | 25 | 605 | nucleobase-containing small molecule metabolic process |
| GO:0015992 | 0.043 | <0.0001 | 3 | 0 | 53 | proton transport |
| GO:0060048 | 0.043 | <0.0001 | 3 | 0 | 53 | cardiac muscle contraction |
| GO:0031347 | 0.044 | 0.455 | 10 | 5 | 180 | regulation of defense response |
| GO:0010951 | 0.044 | 0.301 | 6 | 2 | 110 | negative regulation of endopeptidase activity |
| GO:0045333 | 0.044 | 0.301 | 6 | 2 | 110 | cellular respiration |
| GO:0070838 | 0.044 | 0.456 | 10 | 5 | 183 | divalent metal ion transport |
| GO:0014066 | 0.046 | <0.0001 | 3 | 0 | 52 | regulation of phosphatidylinositol 3-kinase cascade |
| GO:0055085 | 0.046 | 0.515 | 13 | 7 | 223 | transmembrane transport |
| GO:0006612 | 0.046 | 0.304 | 6 | 2 | 109 | protein targeting to membrane |
| GO:0043627 | 0.046 | 0.304 | 6 | 2 | 109 | response to estrogen stimulus |
| GO:0009261 | 0.046 | 0.572 | 17 | 10 | 293 | ribonucleotide catabolic process |
| GO:0051222 | 0.046 | 0.372 | 8 | 3 | 134 | positive regulation of protein transport |
| GO:1901292 | 0.047 | 0.600 | 19 | 12 | 335 | nucleoside phosphate catabolic process |
| GO:0009164 | 0.047 | 0.574 | 17 | 10 | 292 | nucleoside catabolic process |
| GO:0008654 | 0.048 | 0.375 | 8 | 3 | 133 | phospholipid biosynthetic process |
| GO:0031098 | 0.049 | 0.425 | 9 | 4 | 157 | stress-activated protein kinase signaling cascade |
| GO:0045761 | 0.049 | <0.0001 | 3 | 0 | 51 | regulation of adenylate cyclase activity |
| GO:0006633 | 0.049 | 0.204 | 5 | 1 | 81 | fatty acid biosynthetic process |
| GO:0007188 | 0.049 | 0.204 | 5 | 1 | 81 | adenylate cyclase-modulating G-protein coupled receptor signaling pathway |
| GO:0009063 | 0.049 | 0.204 | 5 | 1 | 81 | cellular amino acid catabolic process |
| GO:0031214 | 0.049 | 0.204 | 5 | 1 | 81 | biomineral tissue development |
| GO:0032147 | 0.049 | 0.464 | 10 | 5 | 180 | activation of protein kinase activity |
