# Supplementary material for: Hidden Treasures in “Ancient” Microarrays: Gene-Expression Portrays Biology and Potential Resistance Pathways of Major Lung Cancer Subtypes and Normal Tissue
Source: Front Oncol. 2014 Sep 29;4:251. doi: 10.3389/fonc.2014.00251 (PMC4178426; doi:10.3389/fonc.2014.00251)
Supplement: Figure S1 — Clinically relevant differentially expressed pathways in each tissue type versus the rest. The pathway maps are arranged according to the scheme in the left-top corner. Red indicates overexpression in genes, dark green downregulation. In the Cell Cycle pathway, tumor suppressor genes were overexpressed in normal lung tissue while oncogenes and tumor driving cyclins were overexpressed in cancers. The gene thymidylate synthase (TYMS or TS, belonging to the pathway “one carbon pool by folate”) is relevant for tumor growth and is also a treatment target. Notably, TYMS was overexpressed only in the tumors that are generally refractory to the drug pemetrexed, as the squamous and the small-cell lung cancer. TYMS was not overexpressed in mesothelioma, but it is known that TYMS expression is highly variable in this cancer. In the ERBB pathway, the ERBB2/HER2 and ERBB3/HER3 were overexpressed in adenocarcinoma, while the ERBB4 was overexpressed in the carcinoids. [file Data_Sheet_1.ZIP › File S3.ZIP!/GO Analysis/Squamous vs Rest Enrichment Analysis/Overrepresented GO terms of Differentially Expressed Genes.html]

Gene to GO BP Conditional test for over-representation

| GOBPID | Pvalue | OddsRatio | ExpCount | Count | Size | Term |
| --- | --- | --- | --- | --- | --- | --- |
| GO:0006271 | <0.0001 | 6.745 | 13 | 25 | 30 | DNA strand elongation involved in DNA replication |
| GO:0000216 | <0.0001 | 2.764 | 30 | 47 | 70 | M/G1 transition of mitotic cell cycle |
| GO:0000084 | <0.0001 | 2.120 | 53 | 75 | 123 | S phase of mitotic cell cycle |
| GO:0000722 | <0.0001 | 5.117 | 10 | 19 | 24 | telomere maintenance via recombination |
| GO:0051325 | <0.0001 | 1.490 | 133 | 163 | 312 | interphase |
| GO:0006281 | <0.0001 | 1.612 | 92 | 117 | 216 | DNA repair |
| GO:0000075 | 0.001 | 1.634 | 78 | 100 | 183 | cell cycle checkpoint |
| GO:0006270 | 0.001 | 4.263 | 11 | 19 | 25 | DNA replication initiation |
| GO:0007006 | 0.001 | 3.847 | 12 | 20 | 27 | mitochondrial membrane organization |
| GO:0000226 | 0.001 | 1.630 | 74 | 95 | 174 | microtubule cytoskeleton organization |
| GO:0031581 | 0.001 | 13.442 | 5 | 10 | 11 | hemidesmosome assembly |
| GO:0045786 | 0.001 | 1.411 | 141 | 168 | 330 | negative regulation of cell cycle |
| GO:0032201 | 0.001 | 4.038 | 10 | 18 | 24 | telomere maintenance via semi-conservative replication |
| GO:0000236 | 0.002 | 2.527 | 20 | 30 | 46 | mitotic prometaphase |
| GO:0033554 | 0.002 | 1.234 | 363 | 402 | 849 | cellular response to stress |
| GO:0032200 | 0.003 | 2.321 | 21 | 31 | 49 | telomere organization |
| GO:0050657 | 0.004 | 1.837 | 36 | 49 | 85 | nucleic acid transport |
| GO:0051236 | 0.004 | 1.837 | 36 | 49 | 85 | establishment of RNA localization |
| GO:0000082 | 0.004 | 1.554 | 68 | 85 | 159 | G1/S transition of mitotic cell cycle |
| GO:0005980 | 0.004 | 3.587 | 9 | 16 | 22 | glycogen catabolic process |
| GO:0044275 | 0.004 | 2.298 | 20 | 29 | 46 | cellular carbohydrate catabolic process |
| GO:0007569 | 0.005 | 2.083 | 24 | 34 | 56 | cell aging |
| GO:0006297 | 0.005 | 4.370 | 7 | 13 | 17 | nucleotide-excision repair, DNA gap filling |
| GO:0000375 | 0.005 | 1.540 | 66 | 82 | 154 | RNA splicing, via transesterification reactions |
| GO:0005996 | 0.005 | 1.449 | 88 | 106 | 205 | monosaccharide metabolic process |
| GO:0007155 | 0.006 | 1.231 | 290 | 322 | 679 | cell adhesion |
| GO:0010564 | 0.006 | 1.348 | 132 | 154 | 309 | regulation of cell cycle process |
| GO:0006068 | 0.006 | Inf | 3 | 6 | 6 | ethanol catabolic process |
| GO:0010954 | 0.006 | Inf | 3 | 6 | 6 | positive regulation of protein processing |
| GO:0008053 | 0.006 | 10.747 | 4 | 8 | 9 | mitochondrial fusion |
| GO:0006820 | 0.007 | 1.417 | 94 | 113 | 221 | anion transport |
| GO:0006521 | 0.007 | 2.053 | 23 | 32 | 53 | regulation of cellular amino acid metabolic process |
| GO:0097035 | 0.007 | 4.928 | 6 | 11 | 14 | regulation of membrane lipid distribution |
| GO:0045806 | 0.007 | 3.362 | 9 | 15 | 21 | negative regulation of endocytosis |
| GO:0006284 | 0.007 | 2.569 | 14 | 21 | 32 | base-excision repair |
| GO:0042490 | 0.007 | 2.569 | 14 | 21 | 32 | mechanoreceptor differentiation |
| GO:0034329 | 0.008 | 1.541 | 59 | 73 | 137 | cell junction assembly |
| GO:0022402 | 0.009 | 1.313 | 143 | 165 | 338 | cell cycle process |
| GO:0000280 | 0.010 | 1.391 | 94 | 111 | 219 | nuclear division |
| GO:0060008 | 0.010 | 6.046 | 5 | 9 | 11 | Sertoli cell differentiation |
| GO:0051028 | 0.010 | 1.756 | 32 | 43 | 76 | mRNA transport |
| GO:0019320 | 0.010 | 1.797 | 30 | 40 | 70 | hexose catabolic process |
| GO:0014015 | 0.010 | 2.690 | 12 | 18 | 27 | positive regulation of gliogenesis |
| GO:0010822 | 0.013 | 3.137 | 9 | 14 | 20 | positive regulation of mitochondrion organization |
| GO:0000087 | 0.013 | 1.365 | 96 | 113 | 225 | M phase of mitotic cell cycle |
| GO:0045332 | 0.013 | 4.479 | 6 | 10 | 13 | phospholipid translocation |
| GO:0010001 | 0.014 | 1.569 | 45 | 57 | 106 | glial cell differentiation |
| GO:0005975 | 0.014 | 1.233 | 211 | 235 | 494 | carbohydrate metabolic process |
| GO:0015884 | 0.014 | Inf | 2 | 5 | 5 | folic acid transport |
| GO:0016188 | 0.014 | Inf | 2 | 5 | 5 | synaptic vesicle maturation |
| GO:0048026 | 0.014 | Inf | 2 | 5 | 5 | positive regulation of mRNA splicing, via spliceosome |
| GO:0048341 | 0.014 | Inf | 2 | 5 | 5 | paraxial mesoderm formation |
| GO:1901028 | 0.014 | Inf | 2 | 5 | 5 | regulation of mitochondrial outer membrane permeabilization |
| GO:0006283 | 0.014 | 2.058 | 18 | 26 | 43 | transcription-coupled nucleotide-excision repair |
| GO:0006405 | 0.015 | 1.952 | 21 | 29 | 49 | RNA export from nucleus |
| GO:0000272 | 0.015 | 2.689 | 10 | 16 | 24 | polysaccharide catabolic process |
| GO:0030330 | 0.015 | 1.599 | 40 | 51 | 94 | DNA damage response, signal transduction by p53 class mediator |
| GO:0045216 | 0.017 | 1.482 | 55 | 67 | 128 | cell-cell junction organization |
| GO:0007052 | 0.018 | 2.323 | 13 | 19 | 30 | mitotic spindle organization |
| GO:0031334 | 0.019 | 1.684 | 31 | 40 | 72 | positive regulation of protein complex assembly |
| GO:0000070 | 0.019 | 2.242 | 14 | 20 | 32 | mitotic sister chromatid segregation |
| GO:0006310 | 0.019 | 1.452 | 59 | 71 | 137 | DNA recombination |
| GO:0031145 | 0.019 | 1.694 | 30 | 39 | 70 | anaphase-promoting complex-dependent proteasomal ubiquitin-dependent protein catabolic process |
| GO:0042487 | 0.019 | 5.372 | 4 | 8 | 10 | regulation of odontogenesis of dentin-containing tooth |
| GO:0060601 | 0.019 | 5.372 | 4 | 8 | 10 | lateral sprouting from an epithelium |
| GO:0044724 | 0.020 | 1.804 | 24 | 32 | 56 | single-organism carbohydrate catabolic process |
| GO:0006006 | 0.020 | 1.401 | 70 | 83 | 163 | glucose metabolic process |
| GO:0061035 | 0.020 | 2.063 | 16 | 23 | 38 | regulation of cartilage development |
| GO:0006260 | 0.021 | 1.365 | 80 | 94 | 187 | DNA replication |
| GO:0031400 | 0.021 | 1.318 | 101 | 117 | 237 | negative regulation of protein modification process |
| GO:0000278 | 0.024 | 1.298 | 108 | 124 | 255 | mitotic cell cycle |
| GO:0001829 | 0.025 | 4.030 | 5 | 9 | 12 | trophectodermal cell differentiation |
| GO:0032011 | 0.025 | 4.030 | 5 | 9 | 12 | ARF protein signal transduction |
| GO:0032781 | 0.025 | 4.030 | 5 | 9 | 12 | positive regulation of ATPase activity |
| GO:0006730 | 0.025 | 2.520 | 10 | 15 | 23 | one-carbon metabolic process |
| GO:0007059 | 0.026 | 1.686 | 27 | 35 | 63 | chromosome segregation |
| GO:0051439 | 0.026 | 1.671 | 28 | 36 | 65 | regulation of ubiquitin-protein ligase activity involved in mitotic cell cycle |
| GO:0002066 | 0.027 | 8.056 | 3 | 6 | 7 | columnar/cuboidal epithelial cell development |
| GO:0008356 | 0.027 | 8.056 | 3 | 6 | 7 | asymmetric cell division |
| GO:0009263 | 0.027 | 8.056 | 3 | 6 | 7 | deoxyribonucleotide biosynthetic process |
| GO:0035794 | 0.027 | 8.056 | 3 | 6 | 7 | positive regulation of mitochondrial membrane permeability |
| GO:0046548 | 0.027 | 8.056 | 3 | 6 | 7 | retinal rod cell development |
| GO:0048711 | 0.027 | 8.056 | 3 | 6 | 7 | positive regulation of astrocyte differentiation |
| GO:0051570 | 0.027 | 8.056 | 3 | 6 | 7 | regulation of histone H3-K9 methylation |
| GO:0034446 | 0.027 | 2.285 | 12 | 17 | 27 | substrate adhesion-dependent cell spreading |
| GO:0001824 | 0.028 | 2.133 | 13 | 19 | 31 | blastocyst development |
| GO:0001704 | 0.028 | 1.738 | 23 | 31 | 55 | formation of primary germ layer |
| GO:0016071 | 0.028 | 1.226 | 167 | 186 | 392 | mRNA metabolic process |
| GO:0033043 | 0.029 | 1.237 | 152 | 170 | 356 | regulation of organelle organization |
| GO:0040014 | 0.029 | 1.755 | 23 | 30 | 53 | regulation of multicellular organism growth |
| GO:0006303 | 0.029 | 3.358 | 6 | 10 | 14 | double-strand break repair via nonhomologous end joining |
| GO:0032891 | 0.029 | 3.358 | 6 | 10 | 14 | negative regulation of organic acid transport |
| GO:0072655 | 0.029 | 2.069 | 14 | 20 | 33 | establishment of protein localization to mitochondrion |
| GO:0006096 | 0.029 | 1.794 | 21 | 28 | 49 | glycolysis |
| GO:0051494 | 0.029 | 1.938 | 17 | 23 | 39 | negative regulation of cytoskeleton organization |
| GO:0032272 | 0.030 | 1.972 | 16 | 22 | 37 | negative regulation of protein polymerization |
| GO:0043588 | 0.030 | 1.933 | 17 | 23 | 39 | skin development |
| GO:0048546 | 0.030 | 1.933 | 17 | 23 | 39 | digestive tract morphogenesis |
| GO:0001657 | 0.030 | 1.565 | 34 | 43 | 80 | ureteric bud development |
| GO:0000398 | 0.031 | 1.389 | 61 | 73 | 144 | mRNA splicing, via spliceosome |
| GO:0008334 | 0.032 | 2.955 | 7 | 11 | 16 | histone mRNA metabolic process |
| GO:0051898 | 0.032 | 2.955 | 7 | 11 | 16 | negative regulation of protein kinase B signaling cascade |
| GO:0051983 | 0.032 | 2.955 | 7 | 11 | 16 | regulation of chromosome segregation |
| GO:0001661 | 0.033 | Inf | 2 | 4 | 4 | conditioned taste aversion |
| GO:0001844 | 0.033 | Inf | 2 | 4 | 4 | protein insertion into mitochondrial membrane involved in apoptotic signaling pathway |
| GO:0006868 | 0.033 | Inf | 2 | 4 | 4 | glutamine transport |
| GO:0009211 | 0.033 | Inf | 2 | 4 | 4 | pyrimidine deoxyribonucleoside triphosphate metabolic process |
| GO:0009221 | 0.033 | Inf | 2 | 4 | 4 | pyrimidine deoxyribonucleotide biosynthetic process |
| GO:0010898 | 0.033 | Inf | 2 | 4 | 4 | positive regulation of triglyceride catabolic process |
| GO:0015886 | 0.033 | Inf | 2 | 4 | 4 | heme transport |
| GO:0021843 | 0.033 | Inf | 2 | 4 | 4 | substrate-independent telencephalic tangential interneuron migration |
| GO:0033088 | 0.033 | Inf | 2 | 4 | 4 | negative regulation of immature T cell proliferation in thymus |
| GO:0035372 | 0.033 | Inf | 2 | 4 | 4 | protein localization to microtubule |
| GO:0044030 | 0.033 | Inf | 2 | 4 | 4 | regulation of DNA methylation |
| GO:0051782 | 0.033 | Inf | 2 | 4 | 4 | negative regulation of cell division |
| GO:0060312 | 0.033 | Inf | 2 | 4 | 4 | regulation of blood vessel remodeling |
| GO:0060430 | 0.033 | Inf | 2 | 4 | 4 | lung saccule development |
| GO:0060686 | 0.033 | Inf | 2 | 4 | 4 | negative regulation of prostatic bud formation |
| GO:0010720 | 0.033 | 1.414 | 53 | 64 | 125 | positive regulation of cell development |
| GO:0031111 | 0.035 | 2.687 | 8 | 12 | 18 | negative regulation of microtubule polymerization or depolymerization |
| GO:2001020 | 0.036 | 1.634 | 26 | 34 | 62 | regulation of response to DNA damage stimulus |
| GO:0007168 | 0.037 | 4.699 | 4 | 7 | 9 | receptor guanylyl cyclase signaling pathway |
| GO:0055094 | 0.037 | 4.699 | 4 | 7 | 9 | response to lipoprotein particle stimulus |
| GO:0060575 | 0.037 | 4.699 | 4 | 7 | 9 | intestinal epithelial cell differentiation |
| GO:0032459 | 0.037 | 2.495 | 9 | 13 | 20 | regulation of protein oligomerization |
| GO:0086065 | 0.037 | 2.495 | 9 | 13 | 20 | cell communication involved in cardiac conduction |
| GO:0046942 | 0.038 | 1.349 | 68 | 79 | 158 | carboxylic acid transport |
| GO:0000077 | 0.038 | 1.459 | 43 | 52 | 100 | DNA damage checkpoint |
| GO:0006308 | 0.038 | 1.668 | 24 | 31 | 56 | DNA catabolic process |
| GO:0006073 | 0.039 | 1.682 | 23 | 30 | 54 | cellular glucan metabolic process |
| GO:0016266 | 0.039 | 2.351 | 9 | 14 | 22 | O-glycan processing |
| GO:0006942 | 0.040 | 1.712 | 21 | 28 | 50 | regulation of striated muscle contraction |
| GO:0001975 | 0.040 | 2.240 | 10 | 15 | 24 | response to amphetamine |
| GO:0010498 | 0.041 | 1.332 | 70 | 82 | 165 | proteasomal protein catabolic process |
| GO:0006396 | 0.041 | 1.209 | 160 | 177 | 375 | RNA processing |
| GO:0070830 | 0.041 | 2.150 | 11 | 16 | 26 | tight junction assembly |
| GO:0032434 | 0.042 | 1.793 | 18 | 24 | 42 | regulation of proteasomal ubiquitin-dependent protein catabolic process |
| GO:0014047 | 0.042 | 2.077 | 12 | 17 | 28 | glutamate secretion |
| GO:0060512 | 0.042 | 2.077 | 12 | 17 | 28 | prostate gland morphogenesis |
| GO:1901880 | 0.043 | 1.965 | 14 | 19 | 32 | negative regulation of protein depolymerization |
| GO:0008630 | 0.043 | 1.920 | 15 | 20 | 34 | intrinsic apoptotic signaling pathway in response to DNA damage |
| GO:0050684 | 0.043 | 1.920 | 15 | 20 | 34 | regulation of mRNA processing |
| GO:0048710 | 0.044 | 3.586 | 5 | 8 | 11 | regulation of astrocyte differentiation |
| GO:0001832 | 0.044 | 3.581 | 5 | 8 | 11 | blastocyst growth |
| GO:0001946 | 0.044 | 3.581 | 5 | 8 | 11 | lymphangiogenesis |
| GO:0031639 | 0.044 | 3.581 | 5 | 8 | 11 | plasminogen activation |
| GO:0007422 | 0.048 | 1.586 | 26 | 33 | 61 | peripheral nervous system development |
| GO:0051444 | 0.048 | 1.586 | 26 | 33 | 61 | negative regulation of ubiquitin-protein ligase activity |
| GO:0051301 | 0.048 | 1.230 | 124 | 138 | 290 | cell division |
| GO:0051340 | 0.049 | 1.488 | 34 | 42 | 80 | regulation of ligase activity |
| GO:0006200 | 0.049 | 1.594 | 25 | 32 | 59 | ATP catabolic process |
| GO:0030216 | 0.049 | 1.594 | 25 | 32 | 59 | keratinocyte differentiation |
| GO:0007019 | 0.050 | 3.022 | 6 | 9 | 13 | microtubule depolymerization |
| GO:0010869 | 0.050 | 3.022 | 6 | 9 | 13 | regulation of receptor biosynthetic process |
| GO:0015949 | 0.050 | 3.022 | 6 | 9 | 13 | nucleobase-containing small molecule interconversion |
| GO:0032332 | 0.050 | 3.022 | 6 | 9 | 13 | positive regulation of chondrocyte differentiation |
| GO:0042026 | 0.050 | 3.022 | 6 | 9 | 13 | protein refolding |
| GO:0048339 | 0.050 | 3.022 | 6 | 9 | 13 | paraxial mesoderm development |
| GO:0051917 | 0.050 | 3.022 | 6 | 9 | 13 | regulation of fibrinolysis |
| GO:0060442 | 0.050 | 3.022 | 6 | 9 | 13 | branching involved in prostate gland morphogenesis |
