# Supplementary material for: Hidden Treasures in “Ancient” Microarrays: Gene-Expression Portrays Biology and Potential Resistance Pathways of Major Lung Cancer Subtypes and Normal Tissue
Source: Front Oncol. 2014 Sep 29;4:251. doi: 10.3389/fonc.2014.00251 (PMC4178426; doi:10.3389/fonc.2014.00251)
Supplement: Figure S1 — Clinically relevant differentially expressed pathways in each tissue type versus the rest. The pathway maps are arranged according to the scheme in the left-top corner. Red indicates overexpression in genes, dark green downregulation. In the Cell Cycle pathway, tumor suppressor genes were overexpressed in normal lung tissue while oncogenes and tumor driving cyclins were overexpressed in cancers. The gene thymidylate synthase (TYMS or TS, belonging to the pathway “one carbon pool by folate”) is relevant for tumor growth and is also a treatment target. Notably, TYMS was overexpressed only in the tumors that are generally refractory to the drug pemetrexed, as the squamous and the small-cell lung cancer. TYMS was not overexpressed in mesothelioma, but it is known that TYMS expression is highly variable in this cancer. In the ERBB pathway, the ERBB2/HER2 and ERBB3/HER3 were overexpressed in adenocarcinoma, while the ERBB4 was overexpressed in the carcinoids. [file Data_Sheet_1.ZIP › File S3.ZIP!/GO Analysis/Squamous vs Rest Enrichment Analysis/Overrepresented GO terms of Down-regulated Genes.html]

Gene to GO BP Conditional test for over-representation

| GOBPID | Pvalue | OddsRatio | ExpCount | Count | Size | Term |
| --- | --- | --- | --- | --- | --- | --- |
| GO:0006811 | <0.0001 | 1.760 | 78 | 115 | 338 | ion transport |
| GO:0007601 | <0.0001 | 2.143 | 32 | 53 | 135 | visual perception |
| GO:0007626 | <0.0001 | 2.256 | 26 | 45 | 111 | locomotory behavior |
| GO:0032501 | <0.0001 | 1.226 | 859 | 931 | 3664 | multicellular organismal process |
| GO:0015711 | <0.0001 | 1.835 | 45 | 68 | 191 | organic anion transport |
| GO:0007600 | <0.0001 | 1.640 | 68 | 95 | 288 | sensory perception |
| GO:0006835 | <0.0001 | 3.024 | 11 | 22 | 46 | dicarboxylic acid transport |
| GO:0030534 | 0.001 | 2.155 | 20 | 34 | 86 | adult behavior |
| GO:0015884 | 0.001 | Inf | 1 | 5 | 5 | folic acid transport |
| GO:0016188 | 0.001 | Inf | 1 | 5 | 5 | synaptic vesicle maturation |
| GO:0007155 | 0.001 | 1.342 | 159 | 194 | 679 | cell adhesion |
| GO:0007168 | 0.001 | 11.464 | 2 | 7 | 9 | receptor guanylyl cyclase signaling pathway |
| GO:0051056 | 0.001 | 1.584 | 55 | 76 | 235 | regulation of small GTPase mediated signal transduction |
| GO:0001975 | 0.001 | 3.878 | 6 | 13 | 24 | response to amphetamine |
| GO:0015872 | 0.001 | 4.509 | 4 | 11 | 19 | dopamine transport |
| GO:0006865 | 0.002 | 2.000 | 21 | 34 | 90 | amino acid transport |
| GO:0015850 | 0.002 | 2.000 | 21 | 34 | 90 | organic hydroxy compound transport |
| GO:0003014 | 0.002 | 2.251 | 15 | 26 | 64 | renal system process |
| GO:0006936 | 0.002 | 1.595 | 48 | 67 | 206 | muscle contraction |
| GO:0003008 | 0.002 | 1.296 | 176 | 209 | 769 | system process |
| GO:0006004 | 0.002 | 6.552 | 3 | 8 | 12 | fucose metabolic process |
| GO:0007602 | 0.002 | 3.281 | 7 | 14 | 28 | phototransduction |
| GO:0015849 | 0.002 | 1.707 | 34 | 49 | 144 | organic acid transport |
| GO:0009187 | 0.002 | 2.304 | 13 | 23 | 56 | cyclic nucleotide metabolic process |
| GO:0036065 | 0.002 | 7.641 | 2 | 7 | 10 | fucosylation |
| GO:0006654 | 0.003 | 4.915 | 4 | 9 | 15 | phosphatidic acid biosynthetic process |
| GO:0032099 | 0.003 | 9.821 | 2 | 6 | 8 | negative regulation of appetite |
| GO:0045956 | 0.003 | 9.821 | 2 | 6 | 8 | positive regulation of calcium ion-dependent exocytosis |
| GO:0008542 | 0.003 | 3.062 | 7 | 14 | 29 | visual learning |
| GO:0001661 | 0.003 | Inf | 1 | 4 | 4 | conditioned taste aversion |
| GO:0008206 | 0.003 | 2.895 | 8 | 15 | 32 | bile acid metabolic process |
| GO:0008015 | 0.003 | 1.729 | 29 | 43 | 126 | blood circulation |
| GO:0010954 | 0.003 | 16.362 | 1 | 5 | 6 | positive regulation of protein processing |
| GO:0030802 | 0.004 | 1.925 | 20 | 31 | 84 | regulation of cyclic nucleotide biosynthetic process |
| GO:1900371 | 0.004 | 1.925 | 20 | 31 | 84 | regulation of purine nucleotide biosynthetic process |
| GO:0007267 | 0.004 | 1.255 | 190 | 221 | 810 | cell-cell signaling |
| GO:0030814 | 0.004 | 1.947 | 18 | 29 | 78 | regulation of cAMP metabolic process |
| GO:0030449 | 0.005 | 4.212 | 4 | 9 | 16 | regulation of complement activation |
| GO:0051898 | 0.005 | 4.212 | 4 | 9 | 16 | negative regulation of protein kinase B signaling cascade |
| GO:0032095 | 0.005 | 5.730 | 3 | 7 | 11 | regulation of response to food |
| GO:0035810 | 0.005 | 5.730 | 3 | 7 | 11 | positive regulation of urine volume |
| GO:0052652 | 0.005 | 1.913 | 18 | 29 | 79 | cyclic purine nucleotide metabolic process |
| GO:0044062 | 0.005 | 3.641 | 4 | 10 | 19 | regulation of excretion |
| GO:0016266 | 0.006 | 3.278 | 5 | 11 | 22 | O-glycan processing |
| GO:0003015 | 0.006 | 1.689 | 28 | 40 | 118 | heart process |
| GO:0060048 | 0.006 | 2.155 | 12 | 21 | 53 | cardiac muscle contraction |
| GO:0050433 | 0.006 | 3.026 | 6 | 12 | 25 | regulation of catecholamine secretion |
| GO:0007190 | 0.006 | 2.842 | 7 | 13 | 28 | activation of adenylate cyclase activity |
| GO:0006816 | 0.006 | 1.540 | 41 | 56 | 176 | calcium ion transport |
| GO:0007610 | 0.006 | 1.341 | 95 | 116 | 403 | behavior |
| GO:0007608 | 0.007 | 4.367 | 3 | 8 | 14 | sensory perception of smell |
| GO:0044704 | 0.008 | 2.386 | 9 | 16 | 38 | single-organism reproductive behavior |
| GO:0023052 | 0.008 | 1.140 | 737 | 782 | 3144 | signaling |
| GO:0007588 | 0.008 | 2.470 | 8 | 15 | 35 | excretion |
| GO:0010563 | 0.008 | 1.488 | 45 | 60 | 193 | negative regulation of phosphorus metabolic process |
| GO:0045761 | 0.008 | 2.118 | 12 | 20 | 51 | regulation of adenylate cyclase activity |
| GO:0031281 | 0.009 | 2.460 | 8 | 15 | 35 | positive regulation of cyclase activity |
| GO:1900544 | 0.009 | 1.988 | 14 | 23 | 61 | positive regulation of purine nucleotide metabolic process |
| GO:0044723 | 0.009 | 1.340 | 86 | 106 | 368 | single-organism carbohydrate metabolic process |
| GO:0072511 | 0.009 | 1.495 | 44 | 58 | 186 | divalent inorganic cation transport |
| GO:0051969 | 0.009 | 1.530 | 38 | 52 | 164 | regulation of transmission of nerve impulse |
| GO:0030810 | 0.009 | 2.007 | 14 | 22 | 58 | positive regulation of nucleotide biosynthetic process |
| GO:0001505 | 0.010 | 1.693 | 24 | 35 | 103 | regulation of neurotransmitter levels |
| GO:0051604 | 0.010 | 1.723 | 23 | 33 | 96 | protein maturation |
| GO:0021700 | 0.010 | 1.595 | 31 | 43 | 132 | developmental maturation |
| GO:0072376 | 0.010 | 2.028 | 13 | 21 | 55 | protein activation cascade |
| GO:0030336 | 0.010 | 1.694 | 23 | 34 | 100 | negative regulation of cell migration |
| GO:0048167 | 0.010 | 1.804 | 19 | 28 | 79 | regulation of synaptic plasticity |
| GO:0042326 | 0.011 | 1.525 | 37 | 50 | 158 | negative regulation of phosphorylation |
| GO:0051349 | 0.012 | 2.342 | 8 | 15 | 36 | positive regulation of lyase activity |
| GO:0010891 | 0.012 | 13.082 | 1 | 4 | 5 | negative regulation of sequestering of triglyceride |
| GO:0016081 | 0.012 | 13.082 | 1 | 4 | 5 | synaptic vesicle docking involved in exocytosis |
| GO:0019388 | 0.012 | 13.082 | 1 | 4 | 5 | galactose catabolic process |
| GO:0031953 | 0.012 | 13.082 | 1 | 4 | 5 | negative regulation of protein autophosphorylation |
| GO:0046339 | 0.012 | 13.082 | 1 | 4 | 5 | diacylglycerol metabolic process |
| GO:0060259 | 0.012 | 13.082 | 1 | 4 | 5 | regulation of feeding behavior |
| GO:0060394 | 0.012 | 13.082 | 1 | 4 | 5 | negative regulation of pathway-restricted SMAD protein phosphorylation |
| GO:0060433 | 0.012 | 13.082 | 1 | 4 | 5 | bronchus development |
| GO:0008016 | 0.012 | 1.669 | 24 | 34 | 101 | regulation of heart contraction |
| GO:0006814 | 0.012 | 1.817 | 17 | 26 | 73 | sodium ion transport |
| GO:0034333 | 0.013 | 2.109 | 11 | 18 | 46 | adherens junction assembly |
| GO:0006524 | 0.013 | Inf | 1 | 3 | 3 | alanine catabolic process |
| GO:0009078 | 0.013 | Inf | 1 | 3 | 3 | pyruvate family amino acid metabolic process |
| GO:0019064 | 0.013 | Inf | 1 | 3 | 3 | viral entry into host cell via membrane fusion with the plasma membrane |
| GO:0021853 | 0.013 | Inf | 1 | 3 | 3 | cerebral cortex GABAergic interneuron migration |
| GO:0033085 | 0.013 | Inf | 1 | 3 | 3 | negative regulation of T cell differentiation in thymus |
| GO:0033603 | 0.013 | Inf | 1 | 3 | 3 | positive regulation of dopamine secretion |
| GO:0034287 | 0.013 | Inf | 1 | 3 | 3 | detection of monosaccharide stimulus |
| GO:0036155 | 0.013 | Inf | 1 | 3 | 3 | acylglycerol acyl-chain remodeling |
| GO:0042271 | 0.013 | Inf | 1 | 3 | 3 | susceptibility to natural killer cell mediated cytotoxicity |
| GO:0045759 | 0.013 | Inf | 1 | 3 | 3 | negative regulation of action potential |
| GO:0046271 | 0.013 | Inf | 1 | 3 | 3 | phenylpropanoid catabolic process |
| GO:0051594 | 0.013 | Inf | 1 | 3 | 3 | detection of glucose |
| GO:0071420 | 0.013 | Inf | 1 | 3 | 3 | cellular response to histamine |
| GO:0097011 | 0.013 | Inf | 1 | 3 | 3 | cellular response to granulocyte macrophage colony-stimulating factor stimulus |
| GO:0045806 | 0.013 | 2.978 | 5 | 10 | 21 | negative regulation of endocytosis |
| GO:0051953 | 0.013 | 2.978 | 5 | 10 | 21 | negative regulation of amine transport |
| GO:1901135 | 0.013 | 1.206 | 202 | 229 | 862 | carbohydrate derivative metabolic process |
| GO:0032374 | 0.013 | 2.772 | 6 | 11 | 24 | regulation of cholesterol transport |
| GO:0055078 | 0.013 | 2.772 | 6 | 11 | 24 | sodium ion homeostasis |
| GO:0044699 | 0.014 | 1.167 | 1466 | 1499 | 6279 | single-organism process |
| GO:0010875 | 0.014 | 4.909 | 2 | 6 | 10 | positive regulation of cholesterol efflux |
| GO:0042487 | 0.014 | 4.909 | 2 | 6 | 10 | regulation of odontogenesis of dentin-containing tooth |
| GO:0042737 | 0.014 | 4.909 | 2 | 6 | 10 | drug catabolic process |
| GO:0045745 | 0.014 | 4.909 | 2 | 6 | 10 | positive regulation of G-protein coupled receptor protein signaling pathway |
| GO:0060134 | 0.014 | 4.909 | 2 | 6 | 10 | prepulse inhibition |
| GO:0007585 | 0.014 | 2.186 | 9 | 16 | 40 | respiratory gaseous exchange |
| GO:0030801 | 0.014 | 1.900 | 14 | 22 | 60 | positive regulation of cyclic nucleotide metabolic process |
| GO:0006942 | 0.015 | 2.011 | 12 | 19 | 50 | regulation of striated muscle contraction |
| GO:0055088 | 0.016 | 1.914 | 13 | 21 | 57 | lipid homeostasis |
| GO:0045216 | 0.016 | 1.550 | 30 | 41 | 128 | cell-cell junction organization |
| GO:0007193 | 0.016 | 2.295 | 8 | 14 | 34 | adenylate cyclase-inhibiting G-protein coupled receptor signaling pathway |
| GO:0046463 | 0.016 | 2.295 | 8 | 14 | 34 | acylglycerol biosynthetic process |
| GO:0007265 | 0.016 | 1.444 | 43 | 56 | 184 | Ras protein signal transduction |
| GO:0043269 | 0.016 | 1.407 | 50 | 64 | 214 | regulation of ion transport |
| GO:0048878 | 0.017 | 1.239 | 135 | 157 | 580 | chemical homeostasis |
| GO:0030819 | 0.017 | 1.930 | 13 | 20 | 54 | positive regulation of cAMP biosynthetic process |
| GO:0007274 | 0.017 | 3.819 | 3 | 7 | 13 | neuromuscular synaptic transmission |
| GO:0032148 | 0.017 | 3.819 | 3 | 7 | 13 | activation of protein kinase B activity |
| GO:0035815 | 0.017 | 3.819 | 3 | 7 | 13 | positive regulation of renal sodium excretion |
| GO:0045332 | 0.017 | 3.819 | 3 | 7 | 13 | phospholipid translocation |
| GO:0061097 | 0.017 | 2.367 | 7 | 13 | 31 | regulation of protein tyrosine kinase activity |
| GO:0051047 | 0.018 | 1.481 | 36 | 48 | 155 | positive regulation of secretion |
| GO:0006813 | 0.018 | 1.703 | 19 | 28 | 82 | potassium ion transport |
| GO:0042312 | 0.018 | 2.457 | 7 | 12 | 28 | regulation of vasodilation |
| GO:0045777 | 0.018 | 2.457 | 7 | 12 | 28 | positive regulation of blood pressure |
| GO:0034220 | 0.018 | 1.397 | 50 | 64 | 215 | ion transmembrane transport |
| GO:0055080 | 0.018 | 1.321 | 74 | 90 | 315 | cation homeostasis |
| GO:0006182 | 0.019 | 3.274 | 4 | 8 | 16 | cGMP biosynthetic process |
| GO:0050708 | 0.019 | 1.643 | 22 | 31 | 93 | regulation of protein secretion |
| GO:0030317 | 0.019 | 2.947 | 4 | 9 | 19 | sperm motility |
| GO:0003073 | 0.019 | 1.862 | 14 | 21 | 58 | regulation of systemic arterial blood pressure |
| GO:0044057 | 0.019 | 1.376 | 54 | 68 | 233 | regulation of system process |
| GO:0016052 | 0.019 | 1.596 | 24 | 34 | 104 | carbohydrate catabolic process |
| GO:0015844 | 0.020 | 1.968 | 11 | 18 | 48 | monoamine transport |
| GO:0042355 | 0.021 | 5.452 | 2 | 5 | 8 | L-fucose catabolic process |
| GO:0010876 | 0.021 | 1.463 | 37 | 48 | 156 | lipid localization |
| GO:0006909 | 0.022 | 1.751 | 16 | 24 | 69 | phagocytosis |
| GO:0030029 | 0.023 | 1.294 | 80 | 96 | 341 | actin filament-based process |
| GO:0032940 | 0.023 | 1.238 | 120 | 139 | 511 | secretion by cell |
| GO:0045776 | 0.024 | 2.313 | 7 | 12 | 29 | negative regulation of blood pressure |
| GO:0006198 | 0.025 | 3.926 | 3 | 6 | 11 | cAMP catabolic process |
| GO:0016338 | 0.025 | 3.926 | 3 | 6 | 11 | calcium-independent cell-cell adhesion |
| GO:0032373 | 0.025 | 3.926 | 3 | 6 | 11 | positive regulation of sterol transport |
| GO:0060008 | 0.025 | 3.926 | 3 | 6 | 11 | Sertoli cell differentiation |
| GO:0086012 | 0.025 | 3.926 | 3 | 6 | 11 | membrane depolarization involved in regulation of cardiac muscle cell action potential |
| GO:0015748 | 0.026 | 2.049 | 9 | 15 | 39 | organophosphate ester transport |
| GO:0042461 | 0.026 | 2.402 | 6 | 11 | 26 | photoreceptor cell development |
| GO:0046626 | 0.026 | 2.402 | 6 | 11 | 26 | regulation of insulin receptor signaling pathway |
| GO:0040013 | 0.026 | 1.520 | 27 | 37 | 117 | negative regulation of locomotion |
| GO:0007628 | 0.027 | 2.519 | 5 | 10 | 23 | adult walking behavior |
| GO:0032891 | 0.027 | 3.273 | 3 | 7 | 14 | negative regulation of organic acid transport |
| GO:0050909 | 0.027 | 3.273 | 3 | 7 | 14 | sensory perception of taste |
| GO:0097035 | 0.027 | 3.273 | 3 | 7 | 14 | regulation of membrane lipid distribution |
| GO:0006873 | 0.027 | 1.242 | 107 | 125 | 458 | cellular ion homeostasis |
| GO:0015698 | 0.028 | 2.085 | 8 | 14 | 36 | inorganic anion transport |
| GO:0048708 | 0.028 | 2.085 | 8 | 14 | 36 | astrocyte differentiation |
| GO:0007612 | 0.028 | 1.674 | 17 | 25 | 74 | learning |
| GO:0032413 | 0.028 | 2.910 | 4 | 8 | 17 | negative regulation of ion transmembrane transporter activity |
| GO:0051271 | 0.030 | 1.530 | 25 | 34 | 107 | negative regulation of cellular component movement |
| GO:0051588 | 0.030 | 6.559 | 1 | 4 | 6 | regulation of neurotransmitter transport |
| GO:0000052 | 0.030 | 6.540 | 1 | 4 | 6 | citrulline metabolic process |
| GO:0002923 | 0.030 | 6.540 | 1 | 4 | 6 | regulation of humoral immune response mediated by circulating immunoglobulin |
| GO:0006734 | 0.030 | 6.540 | 1 | 4 | 6 | NADH metabolic process |
| GO:0010715 | 0.030 | 6.540 | 1 | 4 | 6 | regulation of extracellular matrix disassembly |
| GO:0010887 | 0.030 | 6.540 | 1 | 4 | 6 | negative regulation of cholesterol storage |
| GO:0014050 | 0.030 | 6.540 | 1 | 4 | 6 | negative regulation of glutamate secretion |
| GO:0021892 | 0.030 | 6.540 | 1 | 4 | 6 | cerebral cortex GABAergic interneuron differentiation |
| GO:0030432 | 0.030 | 6.540 | 1 | 4 | 6 | peristalsis |
| GO:0032486 | 0.030 | 6.540 | 1 | 4 | 6 | Rap protein signal transduction |
| GO:0033083 | 0.030 | 6.540 | 1 | 4 | 6 | regulation of immature T cell proliferation |
| GO:0043951 | 0.030 | 6.540 | 1 | 4 | 6 | negative regulation of cAMP-mediated signaling |
| GO:0046655 | 0.030 | 6.540 | 1 | 4 | 6 | folic acid metabolic process |
| GO:0051481 | 0.030 | 6.540 | 1 | 4 | 6 | reduction of cytosolic calcium ion concentration |
| GO:0051497 | 0.030 | 6.540 | 1 | 4 | 6 | negative regulation of stress fiber assembly |
| GO:0061037 | 0.030 | 6.540 | 1 | 4 | 6 | negative regulation of cartilage development |
| GO:0070254 | 0.030 | 6.540 | 1 | 4 | 6 | mucus secretion |
| GO:2000021 | 0.031 | 1.487 | 29 | 38 | 122 | regulation of ion homeostasis |
| GO:0008217 | 0.031 | 1.982 | 9 | 15 | 40 | regulation of blood pressure |
| GO:0045937 | 0.031 | 1.221 | 120 | 138 | 512 | positive regulation of phosphate metabolic process |
| GO:0001974 | 0.032 | 2.187 | 7 | 12 | 30 | blood vessel remodeling |
| GO:0022600 | 0.032 | 1.966 | 9 | 15 | 40 | digestive system process |
| GO:0043547 | 0.032 | 1.527 | 24 | 33 | 104 | positive regulation of GTPase activity |
| GO:0007188 | 0.033 | 1.866 | 11 | 17 | 47 | adenylate cyclase-modulating G-protein coupled receptor signaling pathway |
| GO:0007187 | 0.033 | 2.271 | 6 | 11 | 27 | G-protein coupled receptor signaling pathway, coupled to cyclic nucleotide second messenger |
| GO:0019932 | 0.033 | 1.482 | 28 | 37 | 119 | second-messenger-mediated signaling |
| GO:0007204 | 0.034 | 1.449 | 31 | 41 | 134 | elevation of cytosolic calcium ion concentration |
| GO:0009743 | 0.034 | 1.509 | 25 | 34 | 108 | response to carbohydrate stimulus |
| GO:0050880 | 0.034 | 1.542 | 23 | 31 | 97 | regulation of blood vessel size |
| GO:0007165 | 0.034 | 1.108 | 651 | 684 | 2786 | signal transduction |
| GO:0006958 | 0.034 | 2.251 | 6 | 11 | 27 | complement activation, classical pathway |
| GO:0035094 | 0.034 | 2.251 | 6 | 11 | 27 | response to nicotine |
| GO:0048646 | 0.035 | 1.147 | 265 | 290 | 1132 | anatomical structure formation involved in morphogenesis |
| GO:0046365 | 0.036 | 1.640 | 17 | 24 | 72 | monosaccharide catabolic process |
| GO:0010524 | 0.036 | 2.339 | 6 | 10 | 24 | positive regulation of calcium ion transport into cytosol |
| GO:0060428 | 0.036 | 2.339 | 6 | 10 | 24 | lung epithelium development |
| GO:0002016 | 0.038 | 4.088 | 2 | 5 | 9 | regulation of blood volume by renin-angiotensin |
| GO:0043249 | 0.038 | 4.088 | 2 | 5 | 9 | erythrocyte maturation |
| GO:0070874 | 0.038 | 4.088 | 2 | 5 | 9 | negative regulation of glycogen metabolic process |
| GO:0072310 | 0.038 | 4.088 | 2 | 5 | 9 | glomerular epithelial cell development |
| GO:0045576 | 0.038 | 2.028 | 8 | 13 | 34 | mast cell activation |
| GO:1901215 | 0.039 | 1.557 | 20 | 28 | 87 | negative regulation of neuron death |
| GO:0019637 | 0.039 | 1.167 | 190 | 211 | 811 | organophosphate metabolic process |
| GO:0045766 | 0.039 | 1.640 | 16 | 23 | 69 | positive regulation of angiogenesis |
| GO:0048588 | 0.040 | 1.680 | 15 | 21 | 62 | developmental cell growth |
| GO:0002347 | 0.041 | 3.271 | 3 | 6 | 12 | response to tumor cell |
| GO:0006957 | 0.041 | 3.271 | 3 | 6 | 12 | complement activation, alternative pathway |
| GO:0032108 | 0.041 | 3.271 | 3 | 6 | 12 | negative regulation of response to nutrient levels |
| GO:0032781 | 0.041 | 3.271 | 3 | 6 | 12 | positive regulation of ATPase activity |
| GO:0050802 | 0.041 | 3.271 | 3 | 6 | 12 | circadian sleep/wake cycle, sleep |
| GO:0017157 | 0.041 | 1.730 | 13 | 19 | 55 | regulation of exocytosis |
| GO:0001961 | 0.041 | 2.863 | 4 | 7 | 15 | positive regulation of cytokine-mediated signaling pathway |
| GO:0006111 | 0.041 | 2.863 | 4 | 7 | 15 | regulation of gluconeogenesis |
| GO:0007270 | 0.042 | 1.607 | 17 | 24 | 73 | neuron-neuron synaptic transmission |
| GO:0006469 | 0.042 | 1.484 | 25 | 33 | 106 | negative regulation of protein kinase activity |
| GO:0001844 | 0.042 | 9.806 | 1 | 3 | 4 | protein insertion into mitochondrial membrane involved in apoptotic signaling pathway |
| GO:0006562 | 0.042 | 9.806 | 1 | 3 | 4 | proline catabolic process |
| GO:0006685 | 0.042 | 9.806 | 1 | 3 | 4 | sphingomyelin catabolic process |
| GO:0009804 | 0.042 | 9.806 | 1 | 3 | 4 | coumarin metabolic process |
| GO:0010898 | 0.042 | 9.806 | 1 | 3 | 4 | positive regulation of triglyceride catabolic process |
| GO:0010966 | 0.042 | 9.806 | 1 | 3 | 4 | regulation of phosphate transport |
| GO:0016082 | 0.042 | 9.806 | 1 | 3 | 4 | synaptic vesicle priming |
| GO:0021843 | 0.042 | 9.806 | 1 | 3 | 4 | substrate-independent telencephalic tangential interneuron migration |
| GO:0030321 | 0.042 | 9.806 | 1 | 3 | 4 | transepithelial chloride transport |
| GO:0033088 | 0.042 | 9.806 | 1 | 3 | 4 | negative regulation of immature T cell proliferation in thymus |
| GO:0034442 | 0.042 | 9.806 | 1 | 3 | 4 | regulation of lipoprotein oxidation |
| GO:0035630 | 0.042 | 9.806 | 1 | 3 | 4 | bone mineralization involved in bone maturation |
| GO:0060312 | 0.042 | 9.806 | 1 | 3 | 4 | regulation of blood vessel remodeling |
| GO:0060430 | 0.042 | 9.806 | 1 | 3 | 4 | lung saccule development |
| GO:0060632 | 0.042 | 9.806 | 1 | 3 | 4 | regulation of microtubule-based movement |
| GO:0072592 | 0.042 | 9.806 | 1 | 3 | 4 | oxygen metabolic process |
| GO:1900542 | 0.043 | 1.379 | 37 | 47 | 160 | regulation of purine nucleotide metabolic process |
| GO:0032231 | 0.044 | 1.911 | 9 | 14 | 38 | regulation of actin filament bundle assembly |
| GO:0001990 | 0.045 | 2.119 | 7 | 11 | 28 | regulation of systemic arterial blood pressure by hormone |
| GO:0010742 | 0.045 | 2.119 | 7 | 11 | 28 | macrophage derived foam cell differentiation |
| GO:0035637 | 0.046 | 1.189 | 134 | 151 | 571 | multicellular organismal signaling |
| GO:1901615 | 0.046 | 1.274 | 65 | 77 | 276 | organic hydroxy compound metabolic process |
| GO:0051348 | 0.048 | 1.429 | 29 | 37 | 122 | negative regulation of transferase activity |
| GO:0032012 | 0.048 | 2.182 | 6 | 10 | 25 | regulation of ARF protein signal transduction |
| GO:0042417 | 0.048 | 2.182 | 6 | 10 | 25 | dopamine metabolic process |
| GO:0046928 | 0.048 | 2.182 | 6 | 10 | 25 | regulation of neurotransmitter secretion |
| GO:0051893 | 0.048 | 2.182 | 6 | 10 | 25 | regulation of focal adhesion assembly |
| GO:0030001 | 0.049 | 1.312 | 49 | 60 | 212 | metal ion transport |
