# Supplementary material for: Hidden Treasures in “Ancient” Microarrays: Gene-Expression Portrays Biology and Potential Resistance Pathways of Major Lung Cancer Subtypes and Normal Tissue
Source: Front Oncol. 2014 Sep 29;4:251. doi: 10.3389/fonc.2014.00251 (PMC4178426; doi:10.3389/fonc.2014.00251)
Supplement: Figure S1 — Clinically relevant differentially expressed pathways in each tissue type versus the rest. The pathway maps are arranged according to the scheme in the left-top corner. Red indicates overexpression in genes, dark green downregulation. In the Cell Cycle pathway, tumor suppressor genes were overexpressed in normal lung tissue while oncogenes and tumor driving cyclins were overexpressed in cancers. The gene thymidylate synthase (TYMS or TS, belonging to the pathway “one carbon pool by folate”) is relevant for tumor growth and is also a treatment target. Notably, TYMS was overexpressed only in the tumors that are generally refractory to the drug pemetrexed, as the squamous and the small-cell lung cancer. TYMS was not overexpressed in mesothelioma, but it is known that TYMS expression is highly variable in this cancer. In the ERBB pathway, the ERBB2/HER2 and ERBB3/HER3 were overexpressed in adenocarcinoma, while the ERBB4 was overexpressed in the carcinoids. [file Data_Sheet_1.ZIP › File S3.ZIP!/GO Analysis/Squamous vs Rest Enrichment Analysis/Overrepresented GO terms of Up-regulated Genes.html]

Gene to GO BP Conditional test for over-representation

| GOBPID | Pvalue | OddsRatio | ExpCount | Count | Size | Term |
| --- | --- | --- | --- | --- | --- | --- |
| GO:0090304 | <0.0001 | 1.818 | 480 | 646 | 2488 | nucleic acid metabolic process |
| GO:0000216 | <0.0001 | 8.801 | 13 | 47 | 70 | M/G1 transition of mitotic cell cycle |
| GO:0000084 | <0.0001 | 5.025 | 24 | 66 | 123 | S phase of mitotic cell cycle |
| GO:0000278 | <0.0001 | 3.045 | 53 | 113 | 284 | mitotic cell cycle |
| GO:0044238 | <0.0001 | 1.803 | 432 | 544 | 2405 | primary metabolic process |
| GO:0006725 | <0.0001 | 1.597 | 604 | 744 | 3135 | cellular aromatic compound metabolic process |
| GO:0046483 | <0.0001 | 1.598 | 591 | 729 | 3077 | heterocycle metabolic process |
| GO:0000375 | <0.0001 | 3.811 | 30 | 72 | 154 | RNA splicing, via transesterification reactions |
| GO:0034641 | <0.0001 | 1.559 | 590 | 719 | 3097 | cellular nitrogen compound metabolic process |
| GO:0006281 | <0.0001 | 3.722 | 28 | 68 | 149 | DNA repair |
| GO:1901360 | <0.0001 | 1.524 | 621 | 746 | 3233 | organic cyclic compound metabolic process |
| GO:0045786 | <0.0001 | 2.550 | 58 | 111 | 303 | negative regulation of cell cycle |
| GO:0044267 | <0.0001 | 2.438 | 58 | 108 | 315 | cellular protein metabolic process |
| GO:0000236 | <0.0001 | 7.992 | 9 | 30 | 46 | mitotic prometaphase |
| GO:0031145 | <0.0001 | 5.382 | 13 | 39 | 70 | anaphase-promoting complex-dependent proteasomal ubiquitin-dependent protein catabolic process |
| GO:0016032 | <0.0001 | 2.097 | 84 | 141 | 441 | viral reproduction |
| GO:0016071 | <0.0001 | 5.885 | 11 | 34 | 60 | mRNA metabolic process |
| GO:0048285 | <0.0001 | 2.587 | 45 | 87 | 233 | organelle fission |
| GO:0010564 | <0.0001 | 3.557 | 22 | 53 | 119 | regulation of cell cycle process |
| GO:0006271 | <0.0001 | 11.673 | 6 | 22 | 30 | DNA strand elongation involved in DNA replication |
| GO:0051438 | <0.0001 | 4.501 | 15 | 40 | 78 | regulation of ubiquitin-protein ligase activity |
| GO:0007093 | <0.0001 | 3.575 | 21 | 50 | 110 | mitotic cell cycle checkpoint |
| GO:0050657 | <0.0001 | 4.179 | 16 | 42 | 85 | nucleic acid transport |
| GO:0051236 | <0.0001 | 4.179 | 16 | 42 | 85 | establishment of RNA localization |
| GO:0051301 | <0.0001 | 2.265 | 58 | 103 | 301 | cell division |
| GO:0000398 | <0.0001 | 3.646 | 20 | 48 | 105 | mRNA splicing, via spliceosome |
| GO:0010498 | <0.0001 | 2.874 | 32 | 66 | 165 | proteasomal protein catabolic process |
| GO:0000075 | <0.0001 | 9.183 | 6 | 23 | 34 | cell cycle checkpoint |
| GO:0044260 | <0.0001 | 2.183 | 55 | 95 | 347 | cellular macromolecule metabolic process |
| GO:0051352 | <0.0001 | 4.700 | 12 | 32 | 61 | negative regulation of ligase activity |
| GO:0051437 | <0.0001 | 4.700 | 12 | 32 | 61 | positive regulation of ubiquitin-protein ligase activity involved in mitotic cell cycle |
| GO:0000082 | <0.0001 | 4.123 | 14 | 36 | 74 | G1/S transition of mitotic cell cycle |
| GO:0006310 | <0.0001 | 2.997 | 25 | 53 | 129 | DNA recombination |
| GO:0006521 | <0.0001 | 5.140 | 10 | 29 | 53 | regulation of cellular amino acid metabolic process |
| GO:0051436 | <0.0001 | 4.910 | 11 | 30 | 56 | negative regulation of ubiquitin-protein ligase activity involved in mitotic cell cycle |
| GO:0006270 | <0.0001 | 10.888 | 5 | 18 | 25 | DNA replication initiation |
| GO:0006511 | <0.0001 | 2.241 | 47 | 84 | 246 | ubiquitin-dependent protein catabolic process |
| GO:0051351 | <0.0001 | 4.139 | 13 | 34 | 69 | positive regulation of ligase activity |
| GO:0072422 | <0.0001 | 4.398 | 12 | 31 | 61 | signal transduction involved in DNA damage checkpoint |
| GO:0031397 | <0.0001 | 3.669 | 15 | 37 | 80 | negative regulation of protein ubiquitination |
| GO:0072395 | <0.0001 | 4.255 | 12 | 31 | 62 | signal transduction involved in cell cycle checkpoint |
| GO:0043632 | <0.0001 | 2.159 | 49 | 84 | 252 | modification-dependent macromolecule catabolic process |
| GO:0000722 | <0.0001 | 10.276 | 5 | 17 | 24 | telomere maintenance via recombination |
| GO:0034660 | <0.0001 | 3.589 | 15 | 36 | 79 | ncRNA metabolic process |
| GO:0031571 | <0.0001 | 4.121 | 12 | 31 | 63 | mitotic cell cycle G1/S transition DNA damage checkpoint |
| GO:0006977 | <0.0001 | 4.253 | 12 | 30 | 60 | DNA damage response, signal transduction by p53 class mediator resulting in cell cycle arrest |
| GO:0072474 | <0.0001 | 4.253 | 12 | 30 | 60 | signal transduction involved in mitotic cell cycle G1/S checkpoint |
| GO:0051325 | <0.0001 | 2.380 | 34 | 64 | 184 | interphase |
| GO:0034622 | <0.0001 | 1.922 | 66 | 106 | 345 | cellular macromolecular complex assembly |
| GO:0044703 | <0.0001 | 1.677 | 115 | 165 | 596 | multi-organism reproductive process |
| GO:0071779 | <0.0001 | 3.619 | 14 | 34 | 74 | G1/S transition checkpoint |
| GO:0000279 | <0.0001 | 2.100 | 48 | 81 | 250 | M phase |
| GO:0044257 | <0.0001 | 2.020 | 53 | 88 | 276 | cellular protein catabolic process |
| GO:0000003 | <0.0001 | 1.480 | 213 | 276 | 1106 | reproduction |
| GO:0044265 | <0.0001 | 2.284 | 35 | 63 | 185 | cellular macromolecule catabolic process |
| GO:0000209 | <0.0001 | 2.932 | 19 | 41 | 101 | protein polyubiquitination |
| GO:0032201 | <0.0001 | 8.456 | 5 | 16 | 24 | telomere maintenance via semi-conservative replication |
| GO:0006283 | <0.0001 | 4.876 | 8 | 23 | 43 | transcription-coupled nucleotide-excision repair |
| GO:0009058 | <0.0001 | 1.362 | 442 | 519 | 2355 | biosynthetic process |
| GO:0071704 | <0.0001 | 1.516 | 232 | 286 | 1448 | organic substance metabolic process |
| GO:0090068 | <0.0001 | 2.484 | 26 | 50 | 136 | positive regulation of cell cycle process |
| GO:0032200 | <0.0001 | 4.070 | 9 | 24 | 49 | telomere organization |
| GO:2000045 | <0.0001 | 3.012 | 16 | 34 | 82 | regulation of G1/S transition of mitotic cell cycle |
| GO:0031398 | <0.0001 | 2.722 | 19 | 39 | 100 | positive regulation of protein ubiquitination |
| GO:0031323 | <0.0001 | 1.309 | 511 | 586 | 2650 | regulation of cellular metabolic process |
| GO:0006369 | <0.0001 | 5.024 | 7 | 19 | 35 | termination of RNA polymerase II transcription |
| GO:0032446 | <0.0001 | 1.815 | 59 | 91 | 308 | protein modification by small protein conjugation |
| GO:0051028 | <0.0001 | 5.120 | 6 | 18 | 33 | mRNA transport |
| GO:0002479 | <0.0001 | 3.300 | 12 | 28 | 64 | antigen processing and presentation of exogenous peptide antigen via MHC class I, TAP-dependent |
| GO:0010467 | <0.0001 | 1.796 | 59 | 90 | 340 | gene expression |
| GO:0019048 | <0.0001 | 1.797 | 58 | 89 | 302 | virus-host interaction |
| GO:0019058 | <0.0001 | 2.067 | 35 | 59 | 181 | viral infectious cycle |
| GO:0034654 | <0.0001 | 1.309 | 401 | 468 | 2083 | nucleobase-containing compound biosynthetic process |
| GO:0051276 | <0.0001 | 1.798 | 56 | 86 | 297 | chromosome organization |
| GO:0031581 | <0.0001 | 18.954 | 2 | 9 | 11 | hemidesmosome assembly |
| GO:0010639 | <0.0001 | 2.320 | 23 | 43 | 122 | negative regulation of organelle organization |
| GO:0007059 | <0.0001 | 3.615 | 9 | 22 | 48 | chromosome segregation |
| GO:0000070 | <0.0001 | 4.834 | 6 | 16 | 30 | mitotic sister chromatid segregation |
| GO:0006396 | <0.0001 | 2.414 | 20 | 37 | 105 | RNA processing |
| GO:0071840 | <0.0001 | 3.560 | 8 | 20 | 47 | cellular component organization or biogenesis |
| GO:0007052 | <0.0001 | 7.750 | 3 | 11 | 17 | mitotic spindle organization |
| GO:0006297 | <0.0001 | 7.728 | 3 | 11 | 17 | nucleotide-excision repair, DNA gap filling |
| GO:0006370 | <0.0001 | 4.871 | 5 | 15 | 28 | 7-methylguanosine mRNA capping |
| GO:0045934 | <0.0001 | 1.466 | 124 | 162 | 641 | negative regulation of nucleobase-containing compound metabolic process |
| GO:0044403 | <0.0001 | 1.628 | 70 | 100 | 364 | symbiosis, encompassing mutualism through parasitism |
| GO:0006913 | <0.0001 | 1.754 | 50 | 75 | 258 | nucleocytoplasmic transport |
| GO:0036260 | <0.0001 | 4.522 | 6 | 15 | 29 | RNA capping |
| GO:0006364 | <0.0001 | 3.041 | 11 | 23 | 55 | rRNA processing |
| GO:0012501 | <0.0001 | 1.339 | 219 | 266 | 1137 | programmed cell death |
| GO:0002474 | <0.0001 | 2.480 | 16 | 31 | 84 | antigen processing and presentation of peptide antigen via MHC class I |
| GO:0030216 | <0.0001 | 2.902 | 11 | 24 | 59 | keratinocyte differentiation |
| GO:0032269 | <0.0001 | 1.655 | 59 | 85 | 305 | negative regulation of cellular protein metabolic process |
| GO:0016043 | <0.0001 | 1.375 | 178 | 220 | 977 | cellular component organization |
| GO:0051171 | <0.0001 | 1.260 | 399 | 456 | 2072 | regulation of nitrogen compound metabolic process |
| GO:0006368 | <0.0001 | 2.685 | 13 | 26 | 67 | transcription elongation from RNA polymerase II promoter |
| GO:0006406 | <0.0001 | 3.345 | 8 | 19 | 43 | mRNA export from nucleus |
| GO:0008334 | <0.0001 | 7.020 | 3 | 10 | 16 | histone mRNA metabolic process |
| GO:2001141 | <0.0001 | 1.279 | 316 | 368 | 1641 | regulation of RNA biosynthetic process |
| GO:0034645 | <0.0001 | 1.294 | 288 | 337 | 1580 | cellular macromolecule biosynthetic process |
| GO:0006402 | <0.0001 | 2.097 | 23 | 40 | 121 | mRNA catabolic process |
| GO:0034446 | <0.0001 | 4.574 | 5 | 13 | 25 | substrate adhesion-dependent cell spreading |
| GO:0050434 | <0.0001 | 3.018 | 9 | 20 | 48 | positive regulation of viral transcription |
| GO:0051704 | <0.0001 | 1.339 | 183 | 223 | 955 | multi-organism process |
| GO:0002478 | <0.0001 | 2.004 | 25 | 42 | 131 | antigen processing and presentation of exogenous peptide antigen |
| GO:0071826 | <0.0001 | 2.501 | 13 | 26 | 70 | ribonucleoprotein complex subunit organization |
| GO:0000086 | <0.0001 | 2.275 | 17 | 31 | 89 | G2/M transition of mitotic cell cycle |
| GO:0009124 | <0.0001 | 3.936 | 6 | 14 | 29 | nucleoside monophosphate biosynthetic process |
| GO:0080090 | <0.0001 | 1.227 | 492 | 548 | 2587 | regulation of primary metabolic process |
| GO:0050792 | <0.0001 | 2.185 | 19 | 33 | 97 | regulation of viral reproduction |
| GO:0009890 | <0.0001 | 1.381 | 135 | 170 | 702 | negative regulation of biosynthetic process |
| GO:0022402 | <0.0001 | 1.723 | 41 | 61 | 226 | cell cycle process |
| GO:0006446 | <0.0001 | 3.262 | 8 | 17 | 39 | regulation of translational initiation |
| GO:0006413 | <0.0001 | 2.400 | 14 | 27 | 75 | translational initiation |
| GO:0034655 | <0.0001 | 1.438 | 101 | 131 | 523 | nucleobase-containing compound catabolic process |
| GO:1901575 | <0.0001 | 1.286 | 241 | 284 | 1249 | organic substance catabolic process |
| GO:0022613 | 0.001 | 2.727 | 10 | 21 | 54 | ribonucleoprotein complex biogenesis |
| GO:0010605 | 0.001 | 1.555 | 63 | 87 | 336 | negative regulation of macromolecule metabolic process |
| GO:0010821 | 0.001 | 3.515 | 6 | 15 | 33 | regulation of mitochondrion organization |
| GO:0071824 | 0.001 | 2.229 | 17 | 30 | 87 | protein-DNA complex subunit organization |
| GO:0006284 | 0.001 | 3.695 | 6 | 14 | 30 | base-excision repair |
| GO:0000184 | 0.001 | 2.232 | 16 | 29 | 84 | nuclear-transcribed mRNA catabolic process, nonsense-mediated decay |
| GO:1901796 | 0.001 | 4.634 | 4 | 11 | 21 | regulation of signal transduction by p53 class mediator |
| GO:0008637 | 0.001 | 2.725 | 10 | 20 | 51 | apoptotic mitochondrial changes |
| GO:0010941 | 0.001 | 1.315 | 178 | 215 | 924 | regulation of cell death |
| GO:0016265 | 0.001 | 1.271 | 243 | 284 | 1259 | death |
| GO:0006333 | 0.001 | 2.349 | 13 | 25 | 70 | chromatin assembly or disassembly |
| GO:0022607 | 0.001 | 1.296 | 194 | 232 | 1009 | cellular component assembly |
| GO:0000083 | 0.001 | 4.678 | 4 | 10 | 19 | regulation of transcription involved in G1/S phase of mitotic cell cycle |
| GO:0000718 | 0.001 | 4.678 | 4 | 10 | 19 | nucleotide-excision repair, DNA damage removal |
| GO:0007049 | 0.001 | 1.796 | 28 | 44 | 160 | cell cycle |
| GO:0001824 | 0.001 | 2.869 | 8 | 17 | 42 | blastocyst development |
| GO:0043902 | 0.001 | 2.196 | 15 | 27 | 79 | positive regulation of multi-organism process |
| GO:0006302 | 0.001 | 3.309 | 6 | 14 | 32 | double-strand break repair |
| GO:0008544 | 0.001 | 2.084 | 17 | 30 | 92 | epidermis development |
| GO:0006338 | 0.001 | 2.370 | 12 | 23 | 64 | chromatin remodeling |
| GO:0022404 | 0.001 | 2.559 | 10 | 20 | 53 | molting cycle process |
| GO:0009211 | 0.001 | Inf | 1 | 4 | 4 | pyrimidine deoxyribonucleoside triphosphate metabolic process |
| GO:2001020 | 0.001 | 3.443 | 6 | 13 | 29 | regulation of response to DNA damage stimulus |
| GO:0000085 | 0.002 | 7.359 | 2 | 7 | 11 | G2 phase of mitotic cell cycle |
| GO:0016441 | 0.002 | 7.359 | 2 | 7 | 11 | posttranscriptional gene silencing |
| GO:0042633 | 0.002 | 2.483 | 10 | 20 | 54 | hair cycle |
| GO:0051983 | 0.002 | 5.616 | 3 | 8 | 14 | regulation of chromosome segregation |
| GO:0019692 | 0.002 | 4.734 | 3 | 9 | 17 | deoxyribose phosphate metabolic process |
| GO:0043412 | 0.002 | 1.219 | 322 | 364 | 1671 | macromolecule modification |
| GO:0022904 | 0.002 | 2.156 | 14 | 25 | 74 | respiratory electron transport chain |
| GO:0044237 | 0.002 | 1.464 | 70 | 91 | 482 | cellular metabolic process |
| GO:0097193 | 0.002 | 2.011 | 17 | 29 | 90 | intrinsic apoptotic signaling pathway |
| GO:0007051 | 0.002 | 3.401 | 5 | 12 | 27 | spindle organization |
| GO:0006200 | 0.002 | 2.333 | 11 | 21 | 59 | ATP catabolic process |
| GO:0006508 | 0.002 | 1.346 | 112 | 139 | 582 | proteolysis |
| GO:0007006 | 0.002 | 3.369 | 5 | 12 | 27 | mitochondrial membrane organization |
| GO:0045839 | 0.002 | 3.369 | 5 | 12 | 27 | negative regulation of mitosis |
| GO:0006974 | 0.002 | 2.196 | 13 | 23 | 71 | response to DNA damage stimulus |
| GO:0008053 | 0.002 | 8.406 | 2 | 6 | 9 | mitochondrial fusion |
| GO:0009113 | 0.002 | 8.406 | 2 | 6 | 9 | purine nucleobase biosynthetic process |
| GO:0006997 | 0.003 | 2.702 | 8 | 16 | 41 | nucleus organization |
| GO:0000724 | 0.003 | 2.950 | 7 | 14 | 34 | double-strand break repair via homologous recombination |
| GO:0042981 | 0.003 | 1.307 | 141 | 170 | 736 | regulation of apoptotic process |
| GO:0006323 | 0.003 | 2.355 | 11 | 20 | 56 | DNA packaging |
| GO:2000241 | 0.003 | 1.682 | 31 | 46 | 162 | regulation of reproductive process |
| GO:0006221 | 0.003 | 4.207 | 3 | 9 | 18 | pyrimidine nucleotide biosynthetic process |
| GO:0009168 | 0.003 | 4.207 | 3 | 9 | 18 | purine ribonucleoside monophosphate biosynthetic process |
| GO:0042771 | 0.003 | 4.207 | 3 | 9 | 18 | intrinsic apoptotic signaling pathway in response to DNA damage by p53 class mediator |
| GO:0000726 | 0.003 | 4.807 | 3 | 8 | 15 | non-recombinational repair |
| GO:0032434 | 0.003 | 2.594 | 8 | 16 | 42 | regulation of proteasomal ubiquitin-dependent protein catabolic process |
| GO:0051084 | 0.004 | 2.809 | 7 | 14 | 35 | 'de novo' posttranslational protein folding |
| GO:0045892 | 0.004 | 1.589 | 37 | 53 | 197 | negative regulation of transcription, DNA-dependent |
| GO:0006268 | 0.004 | 10.502 | 1 | 5 | 7 | DNA unwinding involved in replication |
| GO:0016925 | 0.004 | 3.308 | 5 | 11 | 25 | protein sumoylation |
| GO:0009987 | 0.004 | 1.488 | 251 | 270 | 1915 | cellular process |
| GO:0006260 | 0.004 | 1.805 | 21 | 33 | 112 | DNA replication |
| GO:0046825 | 0.005 | 3.507 | 4 | 10 | 22 | regulation of protein export from nucleus |
| GO:0051783 | 0.005 | 2.110 | 13 | 22 | 66 | regulation of nuclear division |
| GO:0010468 | 0.005 | 1.223 | 243 | 277 | 1304 | regulation of gene expression |
| GO:0006334 | 0.005 | 2.389 | 9 | 17 | 47 | nucleosome assembly |
| GO:0030261 | 0.005 | 3.786 | 4 | 9 | 19 | chromosome condensation |
| GO:0031572 | 0.005 | 3.786 | 4 | 9 | 19 | G2/M transition DNA damage checkpoint |
| GO:0006950 | 0.005 | 1.180 | 401 | 441 | 2079 | response to stress |
| GO:0008608 | 0.005 | 6.304 | 2 | 6 | 10 | attachment of spindle microtubules to kinetochore |
| GO:0043586 | 0.005 | 6.304 | 2 | 6 | 10 | tongue development |
| GO:0051900 | 0.005 | 6.304 | 2 | 6 | 10 | regulation of mitochondrial depolarization |
| GO:0060601 | 0.005 | 6.304 | 2 | 6 | 10 | lateral sprouting from an epithelium |
| GO:0051321 | 0.006 | 1.857 | 18 | 29 | 95 | meiotic cell cycle |
| GO:0015949 | 0.006 | 4.904 | 3 | 7 | 13 | nucleobase-containing small molecule interconversion |
| GO:0006405 | 0.006 | 16.956 | 1 | 4 | 5 | RNA export from nucleus |
| GO:0007091 | 0.006 | 16.892 | 1 | 4 | 5 | mitotic metaphase/anaphase transition |
| GO:0043588 | 0.006 | 2.744 | 6 | 13 | 33 | skin development |
| GO:0033044 | 0.006 | 1.955 | 15 | 25 | 79 | regulation of chromosome organization |
| GO:0009265 | 0.006 | 16.795 | 1 | 4 | 5 | 2'-deoxyribonucleotide biosynthetic process |
| GO:0016246 | 0.006 | 16.795 | 1 | 4 | 5 | RNA interference |
| GO:0019985 | 0.006 | 16.795 | 1 | 4 | 5 | translesion synthesis |
| GO:0048026 | 0.006 | 16.795 | 1 | 4 | 5 | positive regulation of mRNA splicing, via spliceosome |
| GO:0051292 | 0.006 | 16.795 | 1 | 4 | 5 | nuclear pore complex assembly |
| GO:0051299 | 0.006 | 16.795 | 1 | 4 | 5 | centrosome separation |
| GO:0051573 | 0.006 | 16.795 | 1 | 4 | 5 | negative regulation of histone H3-K9 methylation |
| GO:0070389 | 0.006 | 16.795 | 1 | 4 | 5 | chaperone cofactor-dependent protein refolding |
| GO:0085020 | 0.006 | 16.795 | 1 | 4 | 5 | protein K6-linked ubiquitination |
| GO:0070585 | 0.006 | 2.737 | 6 | 13 | 33 | protein localization to mitochondrion |
| GO:0060740 | 0.006 | 3.087 | 5 | 11 | 26 | prostate gland epithelium morphogenesis |
| GO:0009112 | 0.006 | 2.312 | 9 | 17 | 48 | nucleobase metabolic process |
| GO:0000122 | 0.006 | 1.388 | 70 | 89 | 362 | negative regulation of transcription from RNA polymerase II promoter |
| GO:0042176 | 0.006 | 1.721 | 23 | 35 | 121 | regulation of protein catabolic process |
| GO:0061025 | 0.007 | 2.165 | 11 | 19 | 56 | membrane fusion |
| GO:0010389 | 0.007 | 3.237 | 4 | 10 | 23 | regulation of G2/M transition of mitotic cell cycle |
| GO:0051881 | 0.007 | 3.237 | 4 | 10 | 23 | regulation of mitochondrial membrane potential |
| GO:0022411 | 0.007 | 1.602 | 31 | 44 | 161 | cellular component disassembly |
| GO:0006235 | 0.007 | Inf | 1 | 3 | 3 | dTTP biosynthetic process |
| GO:0006269 | 0.007 | Inf | 1 | 3 | 3 | DNA replication, synthesis of RNA primer |
| GO:0032808 | 0.007 | Inf | 1 | 3 | 3 | lacrimal gland development |
| GO:0035986 | 0.007 | Inf | 1 | 3 | 3 | senescence-associated heterochromatin focus assembly |
| GO:0038031 | 0.007 | Inf | 1 | 3 | 3 | non-canonical Wnt receptor signaling pathway via JNK cascade |
| GO:0045040 | 0.007 | Inf | 1 | 3 | 3 | protein import into mitochondrial outer membrane |
| GO:0046100 | 0.007 | Inf | 1 | 3 | 3 | hypoxanthine metabolic process |
| GO:0046602 | 0.007 | Inf | 1 | 3 | 3 | regulation of mitotic centrosome separation |
| GO:2000774 | 0.007 | Inf | 1 | 3 | 3 | positive regulation of cellular senescence |
| GO:0032508 | 0.008 | 3.452 | 4 | 9 | 20 | DNA duplex unwinding |
| GO:0009411 | 0.008 | 2.248 | 9 | 17 | 49 | response to UV |
| GO:0010629 | 0.008 | 2.240 | 9 | 17 | 50 | negative regulation of gene expression |
| GO:0000387 | 0.008 | 3.441 | 4 | 9 | 20 | spliceosomal snRNP assembly |
| GO:0007062 | 0.008 | 3.441 | 4 | 9 | 20 | sister chromatid cohesion |
| GO:0014013 | 0.008 | 2.239 | 9 | 17 | 49 | regulation of gliogenesis |
| GO:0000245 | 0.008 | 2.893 | 5 | 11 | 27 | spliceosomal complex assembly |
| GO:0060113 | 0.008 | 2.893 | 5 | 11 | 27 | inner ear receptor cell differentiation |
| GO:0071103 | 0.009 | 2.691 | 6 | 12 | 31 | DNA conformation change |
| GO:0031069 | 0.009 | 3.737 | 3 | 8 | 17 | hair follicle morphogenesis |
| GO:0090200 | 0.009 | 3.737 | 3 | 8 | 17 | positive regulation of release of cytochrome c from mitochondria |
| GO:0031047 | 0.009 | 7.019 | 2 | 5 | 8 | gene silencing by RNA |
| GO:0001833 | 0.009 | 7.000 | 2 | 5 | 8 | inner cell mass cell proliferation |
| GO:0030850 | 0.009 | 2.340 | 8 | 15 | 42 | prostate gland development |
| GO:0009161 | 0.009 | 2.658 | 6 | 12 | 31 | ribonucleoside monophosphate metabolic process |
| GO:0043933 | 0.009 | 1.260 | 135 | 159 | 711 | macromolecular complex subunit organization |
| GO:0019882 | 0.010 | 1.565 | 31 | 44 | 163 | antigen processing and presentation |
| GO:1900117 | 0.010 | 1.569 | 31 | 43 | 159 | regulation of execution phase of apoptosis |
| GO:0007126 | 0.011 | 1.781 | 18 | 27 | 91 | meiosis |
| GO:0007346 | 0.011 | 1.654 | 23 | 34 | 123 | regulation of mitotic cell cycle |
| GO:0007067 | 0.011 | 1.667 | 22 | 33 | 119 | mitosis |
| GO:0043038 | 0.011 | 2.723 | 5 | 11 | 28 | amino acid activation |
| GO:0043966 | 0.012 | 2.525 | 6 | 12 | 32 | histone H3 acetylation |
| GO:0016482 | 0.013 | 1.299 | 92 | 111 | 477 | cytoplasmic transport |
| GO:0010165 | 0.013 | 3.363 | 3 | 8 | 18 | response to X-ray |
| GO:0031057 | 0.013 | 3.363 | 3 | 8 | 18 | negative regulation of histone modification |
| GO:0031060 | 0.013 | 3.363 | 3 | 8 | 18 | regulation of histone methylation |
| GO:0051297 | 0.013 | 2.385 | 7 | 13 | 36 | centrosome organization |
| GO:0033205 | 0.013 | 2.804 | 5 | 10 | 25 | cell cycle cytokinesis |
| GO:0007018 | 0.013 | 1.788 | 16 | 25 | 84 | microtubule-based movement |
| GO:0043241 | 0.014 | 1.648 | 22 | 32 | 114 | protein complex disassembly |
| GO:0009219 | 0.015 | 8.407 | 1 | 4 | 6 | pyrimidine deoxyribonucleotide metabolic process |
| GO:0050769 | 0.015 | 1.745 | 17 | 26 | 89 | positive regulation of neurogenesis |
| GO:0006189 | 0.015 | 8.396 | 1 | 4 | 6 | 'de novo' IMP biosynthetic process |
| GO:0010388 | 0.015 | 8.396 | 1 | 4 | 6 | cullin deneddylation |
| GO:0016446 | 0.015 | 8.396 | 1 | 4 | 6 | somatic hypermutation of immunoglobulin genes |
| GO:0043248 | 0.015 | 8.396 | 1 | 4 | 6 | proteasome assembly |
| GO:0043589 | 0.015 | 8.396 | 1 | 4 | 6 | skin morphogenesis |
| GO:0045842 | 0.015 | 8.396 | 1 | 4 | 6 | positive regulation of mitotic metaphase/anaphase transition |
| GO:0006081 | 0.015 | 2.574 | 6 | 11 | 29 | cellular aldehyde metabolic process |
| GO:0000079 | 0.015 | 1.953 | 12 | 19 | 60 | regulation of cyclin-dependent protein kinase activity |
| GO:0006399 | 0.015 | 2.046 | 10 | 17 | 52 | tRNA metabolic process |
| GO:0006006 | 0.015 | 1.515 | 31 | 43 | 163 | glucose metabolic process |
| GO:0019083 | 0.016 | 1.835 | 14 | 22 | 73 | viral transcription |
| GO:0009126 | 0.016 | 2.911 | 4 | 9 | 22 | purine nucleoside monophosphate metabolic process |
| GO:0009148 | 0.016 | 4.201 | 2 | 6 | 12 | pyrimidine nucleoside triphosphate biosynthetic process |
| GO:0030517 | 0.016 | 4.201 | 2 | 6 | 12 | negative regulation of axon extension |
| GO:0071359 | 0.016 | 4.201 | 2 | 6 | 12 | cellular response to dsRNA |
| GO:0007183 | 0.017 | 5.250 | 2 | 5 | 9 | SMAD protein complex assembly |
| GO:0043518 | 0.017 | 5.250 | 2 | 5 | 9 | negative regulation of DNA damage response, signal transduction by p53 class mediator |
| GO:0048340 | 0.017 | 5.250 | 2 | 5 | 9 | paraxial mesoderm morphogenesis |
| GO:0051571 | 0.017 | 5.250 | 2 | 5 | 9 | positive regulation of histone H3-K4 methylation |
| GO:0055094 | 0.017 | 5.250 | 2 | 5 | 9 | response to lipoprotein particle stimulus |
| GO:0070918 | 0.017 | 5.250 | 2 | 5 | 9 | production of small RNA involved in gene silencing by RNA |
| GO:0090399 | 0.017 | 5.250 | 2 | 5 | 9 | replicative senescence |
| GO:0006461 | 0.017 | 1.264 | 104 | 124 | 542 | protein complex assembly |
| GO:0051052 | 0.017 | 1.544 | 27 | 38 | 142 | regulation of DNA metabolic process |
| GO:0072527 | 0.018 | 2.105 | 9 | 15 | 45 | pyrimidine-containing compound metabolic process |
| GO:0031577 | 0.018 | 2.628 | 5 | 10 | 26 | spindle checkpoint |
| GO:0031124 | 0.018 | 1.944 | 11 | 18 | 57 | mRNA 3'-end processing |
| GO:0070507 | 0.018 | 2.042 | 9 | 16 | 49 | regulation of microtubule cytoskeleton organization |
| GO:0006366 | 0.018 | 1.256 | 108 | 128 | 580 | transcription from RNA polymerase II promoter |
| GO:0043616 | 0.019 | 3.057 | 4 | 8 | 19 | keratinocyte proliferation |
| GO:0048523 | 0.019 | 1.159 | 307 | 337 | 1666 | negative regulation of cellular process |
| GO:0030032 | 0.020 | 2.435 | 6 | 11 | 30 | lamellipodium assembly |
| GO:0010638 | 0.020 | 1.492 | 31 | 42 | 161 | positive regulation of organelle organization |
| GO:0032259 | 0.020 | 1.806 | 13 | 21 | 70 | methylation |
| GO:0006464 | 0.021 | 1.163 | 278 | 306 | 1455 | cellular protein modification process |
| GO:0007569 | 0.021 | 2.722 | 4 | 9 | 23 | cell aging |
| GO:0006921 | 0.021 | 1.861 | 12 | 19 | 62 | cellular component disassembly involved in execution phase of apoptosis |
| GO:0048729 | 0.021 | 1.318 | 67 | 82 | 346 | tissue morphogenesis |
| GO:0044106 | 0.021 | 1.536 | 26 | 36 | 135 | cellular amine metabolic process |
| GO:0072594 | 0.021 | 1.536 | 26 | 36 | 135 | establishment of protein localization to organelle |
| GO:0007219 | 0.022 | 1.715 | 16 | 24 | 83 | Notch signaling pathway |
| GO:0010827 | 0.022 | 1.895 | 11 | 18 | 58 | regulation of glucose transport |
| GO:0071230 | 0.022 | 2.703 | 4 | 9 | 23 | cellular response to amino acid stimulus |
| GO:0006261 | 0.022 | 2.517 | 5 | 10 | 27 | DNA-dependent DNA replication |
| GO:0070979 | 0.022 | 3.268 | 3 | 7 | 16 | protein K11-linked ubiquitination |
| GO:0010556 | 0.022 | 1.427 | 38 | 50 | 207 | regulation of macromolecule biosynthetic process |
| GO:0009790 | 0.022 | 1.244 | 110 | 129 | 573 | embryo development |
| GO:0000226 | 0.022 | 1.731 | 15 | 23 | 80 | microtubule cytoskeleton organization |
| GO:0045682 | 0.024 | 2.473 | 5 | 10 | 27 | regulation of epidermis development |
| GO:0006015 | 0.024 | 12.588 | 1 | 3 | 4 | 5-phosphoribose 1-diphosphate biosynthetic process |
| GO:0009202 | 0.024 | 12.588 | 1 | 3 | 4 | deoxyribonucleoside triphosphate biosynthetic process |
| GO:0021861 | 0.024 | 12.588 | 1 | 3 | 4 | forebrain radial glial cell differentiation |
| GO:0031053 | 0.024 | 12.588 | 1 | 3 | 4 | primary miRNA processing |
| GO:0032287 | 0.024 | 12.588 | 1 | 3 | 4 | peripheral nervous system myelin maintenance |
| GO:0034501 | 0.024 | 12.588 | 1 | 3 | 4 | protein localization to kinetochore |
| GO:0034720 | 0.024 | 12.588 | 1 | 3 | 4 | histone H3-K4 demethylation |
| GO:0035308 | 0.024 | 12.588 | 1 | 3 | 4 | negative regulation of protein dephosphorylation |
| GO:0045654 | 0.024 | 12.588 | 1 | 3 | 4 | positive regulation of megakaryocyte differentiation |
| GO:0060686 | 0.024 | 12.588 | 1 | 3 | 4 | negative regulation of prostatic bud formation |
| GO:0070934 | 0.024 | 12.588 | 1 | 3 | 4 | CRD-mediated mRNA stabilization |
| GO:0021799 | 0.025 | 3.600 | 3 | 6 | 13 | cerebral cortex radially oriented cell migration |
| GO:0048385 | 0.025 | 3.600 | 3 | 6 | 13 | regulation of retinoic acid receptor signaling pathway |
| GO:0060713 | 0.025 | 3.600 | 3 | 6 | 13 | labyrinthine layer morphogenesis |
| GO:0090307 | 0.025 | 3.600 | 3 | 6 | 13 | spindle assembly involved in mitosis |
| GO:2000736 | 0.025 | 3.600 | 3 | 6 | 13 | regulation of stem cell differentiation |
| GO:0006906 | 0.026 | 2.802 | 4 | 8 | 20 | vesicle fusion |
| GO:0010001 | 0.026 | 1.589 | 20 | 29 | 106 | glial cell differentiation |
| GO:0007369 | 0.026 | 1.636 | 18 | 26 | 93 | gastrulation |
| GO:0048546 | 0.027 | 2.104 | 8 | 13 | 39 | digestive tract morphogenesis |
| GO:0010604 | 0.027 | 1.156 | 261 | 287 | 1354 | positive regulation of macromolecule metabolic process |
| GO:0042254 | 0.027 | 2.548 | 5 | 9 | 24 | ribosome biogenesis |
| GO:2000779 | 0.028 | 4.205 | 2 | 5 | 10 | regulation of double-strand break repair |
| GO:0050686 | 0.028 | 4.199 | 2 | 5 | 10 | negative regulation of mRNA processing |
| GO:0051322 | 0.028 | 4.199 | 2 | 5 | 10 | anaphase |
| GO:0071364 | 0.028 | 4.199 | 2 | 5 | 10 | cellular response to epidermal growth factor stimulus |
| GO:0090201 | 0.028 | 4.199 | 2 | 5 | 10 | negative regulation of release of cytochrome c from mitochondria |
| GO:0006418 | 0.029 | 2.525 | 5 | 9 | 24 | tRNA aminoacylation for protein translation |
| GO:0030330 | 0.029 | 3.001 | 3 | 7 | 17 | DNA damage response, signal transduction by p53 class mediator |
| GO:0034613 | 0.029 | 1.451 | 31 | 41 | 162 | cellular protein localization |
| GO:0006098 | 0.029 | 5.597 | 1 | 4 | 7 | pentose-phosphate shunt |
| GO:0050775 | 0.029 | 5.597 | 1 | 4 | 7 | positive regulation of dendrite morphogenesis |
| GO:0060968 | 0.029 | 5.597 | 1 | 4 | 7 | regulation of gene silencing |
| GO:0070828 | 0.029 | 5.597 | 1 | 4 | 7 | heterochromatin organization |
| GO:0086069 | 0.029 | 5.597 | 1 | 4 | 7 | bundle of His cell to Purkinje myocyte communication |
| GO:0006415 | 0.029 | 1.778 | 12 | 19 | 64 | translational termination |
| GO:0042180 | 0.029 | 1.442 | 32 | 42 | 165 | cellular ketone metabolic process |
| GO:0060249 | 0.030 | 1.472 | 28 | 38 | 147 | anatomical structure homeostasis |
| GO:0016568 | 0.030 | 1.379 | 42 | 53 | 217 | chromatin modification |
| GO:0040029 | 0.030 | 1.843 | 11 | 17 | 56 | regulation of gene expression, epigenetic |
| GO:0034644 | 0.031 | 2.336 | 5 | 10 | 28 | cellular response to UV |
| GO:0048538 | 0.031 | 2.336 | 5 | 10 | 28 | thymus development |
| GO:0001736 | 0.031 | 2.941 | 3 | 7 | 17 | establishment of planar polarity |
| GO:0001783 | 0.031 | 2.941 | 3 | 7 | 17 | B cell apoptotic process |
| GO:0006541 | 0.031 | 2.941 | 3 | 7 | 17 | glutamine metabolic process |
| GO:0001942 | 0.032 | 2.111 | 7 | 12 | 36 | hair follicle development |
| GO:0061458 | 0.032 | 1.390 | 38 | 49 | 198 | reproductive system development |
| GO:0008283 | 0.033 | 1.161 | 218 | 241 | 1136 | cell proliferation |
| GO:0008380 | 0.035 | 1.838 | 10 | 16 | 54 | RNA splicing |
| GO:0030071 | 0.035 | 2.592 | 4 | 8 | 21 | regulation of mitotic metaphase/anaphase transition |
| GO:2000113 | 0.035 | 1.704 | 13 | 20 | 71 | negative regulation of cellular macromolecule biosynthetic process |
| GO:0007094 | 0.035 | 2.586 | 4 | 8 | 21 | mitotic cell cycle spindle assembly checkpoint |
| GO:0046134 | 0.035 | 2.586 | 4 | 8 | 21 | pyrimidine nucleoside biosynthetic process |
| GO:0042476 | 0.035 | 1.655 | 15 | 22 | 78 | odontogenesis |
| GO:0045995 | 0.035 | 1.762 | 12 | 18 | 61 | regulation of embryonic development |
| GO:0043009 | 0.035 | 1.264 | 76 | 90 | 392 | chordate embryonic development |
| GO:0043583 | 0.036 | 1.500 | 24 | 32 | 122 | ear development |
| GO:0051656 | 0.037 | 1.669 | 14 | 21 | 74 | establishment of organelle localization |
| GO:0009263 | 0.037 | Inf | 0 | 2 | 2 | deoxyribonucleotide biosynthetic process |
| GO:0035412 | 0.037 | 3.150 | 3 | 6 | 14 | regulation of catenin import into nucleus |
| GO:0090342 | 0.037 | 3.150 | 3 | 6 | 14 | regulation of cell aging |
| GO:0000059 | 0.037 | Inf | 0 | 2 | 2 | protein import into nucleus, docking |
| GO:0000463 | 0.037 | Inf | 0 | 2 | 2 | maturation of LSU-rRNA from tricistronic rRNA transcript (SSU-rRNA, 5.8S rRNA, LSU-rRNA) |
| GO:0006288 | 0.037 | Inf | 0 | 2 | 2 | base-excision repair, DNA ligation |
| GO:0006388 | 0.037 | Inf | 0 | 2 | 2 | tRNA splicing, via endonucleolytic cleavage and ligation |
| GO:0006428 | 0.037 | Inf | 0 | 2 | 2 | isoleucyl-tRNA aminoacylation |
| GO:0006597 | 0.037 | Inf | 0 | 2 | 2 | spermine biosynthetic process |
| GO:0006659 | 0.037 | Inf | 0 | 2 | 2 | phosphatidylserine biosynthetic process |
| GO:0007000 | 0.037 | Inf | 0 | 2 | 2 | nucleolus organization |
| GO:0007079 | 0.037 | Inf | 0 | 2 | 2 | mitotic chromosome movement towards spindle pole |
| GO:0009177 | 0.037 | Inf | 0 | 2 | 2 | pyrimidine deoxyribonucleoside monophosphate biosynthetic process |
| GO:0009186 | 0.037 | Inf | 0 | 2 | 2 | deoxyribonucleoside diphosphate metabolic process |
| GO:0009217 | 0.037 | Inf | 0 | 2 | 2 | purine deoxyribonucleoside triphosphate catabolic process |
| GO:0009438 | 0.037 | Inf | 0 | 2 | 2 | methylglyoxal metabolic process |
| GO:0009912 | 0.037 | Inf | 0 | 2 | 2 | auditory receptor cell fate commitment |
| GO:0010668 | 0.037 | Inf | 0 | 2 | 2 | ectodermal cell differentiation |
| GO:0010826 | 0.037 | Inf | 0 | 2 | 2 | negative regulation of centrosome duplication |
| GO:0010994 | 0.037 | Inf | 0 | 2 | 2 | free ubiquitin chain polymerization |
| GO:0014040 | 0.037 | Inf | 0 | 2 | 2 | positive regulation of Schwann cell differentiation |
| GO:0015959 | 0.037 | Inf | 0 | 2 | 2 | diadenosine polyphosphate metabolic process |
| GO:0015966 | 0.037 | Inf | 0 | 2 | 2 | diadenosine tetraphosphate biosynthetic process |
| GO:0016488 | 0.037 | Inf | 0 | 2 | 2 | farnesol catabolic process |
| GO:0019100 | 0.037 | Inf | 0 | 2 | 2 | male germ-line sex determination |
| GO:0021557 | 0.037 | Inf | 0 | 2 | 2 | oculomotor nerve development |
| GO:0021747 | 0.037 | Inf | 0 | 2 | 2 | cochlear nucleus development |
| GO:0031022 | 0.037 | Inf | 0 | 2 | 2 | nuclear migration along microfilament |
| GO:0035519 | 0.037 | Inf | 0 | 2 | 2 | protein K29-linked ubiquitination |
| GO:0043103 | 0.037 | Inf | 0 | 2 | 2 | hypoxanthine salvage |
| GO:0044314 | 0.037 | Inf | 0 | 2 | 2 | protein K27-linked ubiquitination |
| GO:0046070 | 0.037 | Inf | 0 | 2 | 2 | dGTP metabolic process |
| GO:0046078 | 0.037 | Inf | 0 | 2 | 2 | dUMP metabolic process |
| GO:0046122 | 0.037 | Inf | 0 | 2 | 2 | purine deoxyribonucleoside metabolic process |
| GO:0046511 | 0.037 | Inf | 0 | 2 | 2 | sphinganine biosynthetic process |
| GO:0046833 | 0.037 | Inf | 0 | 2 | 2 | positive regulation of RNA export from nucleus |
| GO:0048478 | 0.037 | Inf | 0 | 2 | 2 | replication fork protection |
| GO:0048627 | 0.037 | Inf | 0 | 2 | 2 | myoblast development |
| GO:0048743 | 0.037 | Inf | 0 | 2 | 2 | positive regulation of skeletal muscle fiber development |
| GO:0051574 | 0.037 | Inf | 0 | 2 | 2 | positive regulation of histone H3-K9 methylation |
| GO:0051984 | 0.037 | Inf | 0 | 2 | 2 | positive regulation of chromosome segregation |
| GO:0060529 | 0.037 | Inf | 0 | 2 | 2 | squamous basal epithelial stem cell differentiation involved in prostate gland acinus development |
| GO:0060594 | 0.037 | Inf | 0 | 2 | 2 | mammary gland specification |
| GO:0060648 | 0.037 | Inf | 0 | 2 | 2 | mammary gland bud morphogenesis |
| GO:0060956 | 0.037 | Inf | 0 | 2 | 2 | endocardial cell differentiation |
| GO:0061443 | 0.037 | Inf | 0 | 2 | 2 | endocardial cushion cell differentiation |
| GO:0070488 | 0.037 | Inf | 0 | 2 | 2 | neutrophil aggregation |
| GO:0070827 | 0.037 | Inf | 0 | 2 | 2 | chromatin maintenance |
| GO:0071109 | 0.037 | Inf | 0 | 2 | 2 | superior temporal gyrus development |
| GO:0071281 | 0.037 | Inf | 0 | 2 | 2 | cellular response to iron ion |
| GO:0071864 | 0.037 | Inf | 0 | 2 | 2 | positive regulation of cell proliferation in bone marrow |
| GO:0071866 | 0.037 | Inf | 0 | 2 | 2 | negative regulation of apoptotic process in bone marrow |
| GO:0090007 | 0.037 | Inf | 0 | 2 | 2 | regulation of mitotic anaphase |
| GO:0090402 | 0.037 | Inf | 0 | 2 | 2 | oncogene-induced senescence |
| GO:1900025 | 0.037 | Inf | 0 | 2 | 2 | negative regulation of substrate adhesion-dependent cell spreading |
| GO:1900028 | 0.037 | Inf | 0 | 2 | 2 | negative regulation of ruffle assembly |
| GO:2000172 | 0.037 | Inf | 0 | 2 | 2 | regulation of branching morphogenesis of a nerve |
| GO:2000615 | 0.037 | Inf | 0 | 2 | 2 | regulation of histone H3-K9 acetylation |
| GO:2000630 | 0.037 | Inf | 0 | 2 | 2 | positive regulation of miRNA metabolic process |
| GO:2000659 | 0.037 | Inf | 0 | 2 | 2 | regulation of interleukin-1-mediated signaling pathway |
| GO:2001032 | 0.037 | Inf | 0 | 2 | 2 | regulation of double-strand break repair via nonhomologous end joining |
| GO:0043388 | 0.038 | 2.364 | 5 | 9 | 25 | positive regulation of DNA binding |
| GO:0009059 | 0.038 | 1.945 | 8 | 13 | 45 | macromolecule biosynthetic process |
| GO:0035265 | 0.038 | 1.684 | 13 | 20 | 70 | organ growth |
| GO:0006096 | 0.038 | 1.856 | 9 | 15 | 49 | glycolysis |
| GO:0060429 | 0.038 | 1.247 | 82 | 97 | 427 | epithelium development |
| GO:0016570 | 0.039 | 1.413 | 32 | 41 | 164 | histone modification |
| GO:0034968 | 0.039 | 2.212 | 6 | 10 | 29 | histone lysine methylation |
| GO:0006479 | 0.039 | 1.900 | 9 | 14 | 45 | protein methylation |
| GO:0042440 | 0.039 | 1.900 | 9 | 14 | 45 | pigment metabolic process |
| GO:0070646 | 0.039 | 1.900 | 9 | 14 | 45 | protein modification by small protein removal |
| GO:0000077 | 0.039 | 2.730 | 3 | 7 | 18 | DNA damage checkpoint |
| GO:0002200 | 0.040 | 2.018 | 7 | 12 | 37 | somatic diversification of immune receptors |
| GO:0045185 | 0.040 | 1.626 | 15 | 22 | 79 | maintenance of protein location |
| GO:0090398 | 0.042 | 2.678 | 3 | 7 | 18 | cellular senescence |
| GO:0040036 | 0.043 | 2.673 | 3 | 7 | 18 | regulation of fibroblast growth factor receptor signaling pathway |
| GO:0048710 | 0.043 | 2.673 | 3 | 7 | 18 | regulation of astrocyte differentiation |
| GO:0051252 | 0.043 | 1.976 | 7 | 12 | 39 | regulation of RNA metabolic process |
| GO:0010800 | 0.044 | 3.499 | 2 | 5 | 11 | positive regulation of peptidyl-threonine phosphorylation |
| GO:0032467 | 0.044 | 3.499 | 2 | 5 | 11 | positive regulation of cytokinesis |
| GO:0033119 | 0.044 | 3.499 | 2 | 5 | 11 | negative regulation of RNA splicing |
| GO:0033151 | 0.044 | 3.499 | 2 | 5 | 11 | V(D)J recombination |
| GO:0043517 | 0.044 | 3.499 | 2 | 5 | 11 | positive regulation of DNA damage response, signal transduction by p53 class mediator |
| GO:0046040 | 0.044 | 3.499 | 2 | 5 | 11 | IMP metabolic process |
| GO:0060068 | 0.044 | 3.499 | 2 | 5 | 11 | vagina development |
| GO:0055123 | 0.044 | 1.588 | 16 | 23 | 84 | digestive system development |
| GO:0006417 | 0.045 | 1.534 | 19 | 26 | 98 | regulation of translation |
| GO:2001022 | 0.046 | 2.406 | 4 | 8 | 22 | positive regulation of response to DNA damage stimulus |
| GO:0009314 | 0.046 | 1.322 | 45 | 56 | 235 | response to radiation |
| GO:0032271 | 0.046 | 1.597 | 15 | 22 | 80 | regulation of protein polymerization |
| GO:0006886 | 0.046 | 1.217 | 96 | 111 | 498 | intracellular protein transport |
| GO:0051128 | 0.046 | 1.163 | 172 | 191 | 891 | regulation of cellular component organization |
| GO:0044275 | 0.047 | 1.840 | 9 | 14 | 46 | cellular carbohydrate catabolic process |
| GO:0010948 | 0.047 | 1.954 | 7 | 12 | 38 | negative regulation of cell cycle process |
| GO:0006626 | 0.048 | 2.229 | 5 | 9 | 26 | protein targeting to mitochondrion |
| GO:0009060 | 0.048 | 1.941 | 7 | 12 | 38 | aerobic respiration |
| GO:0051320 | 0.049 | 6.521 | 1 | 3 | 5 | S phase |
| GO:0014855 | 0.049 | 2.101 | 6 | 10 | 30 | striated muscle cell proliferation |
| GO:0042026 | 0.049 | 4.207 | 2 | 4 | 8 | protein refolding |
| GO:0006397 | 0.050 | 1.669 | 12 | 18 | 65 | mRNA processing |
| GO:0006044 | 0.050 | 4.197 | 2 | 4 | 8 | N-acetylglucosamine metabolic process |
| GO:0007350 | 0.050 | 4.197 | 2 | 4 | 8 | blastoderm segmentation |
| GO:0008595 | 0.050 | 4.197 | 2 | 4 | 8 | anterior/posterior axis specification, embryo |
| GO:0008635 | 0.050 | 4.197 | 2 | 4 | 8 | activation of cysteine-type endopeptidase activity involved in apoptotic process by cytochrome c |
| GO:0009129 | 0.050 | 4.197 | 2 | 4 | 8 | pyrimidine nucleoside monophosphate metabolic process |
| GO:0010820 | 0.050 | 4.197 | 2 | 4 | 8 | positive regulation of T cell chemotaxis |
| GO:0035195 | 0.050 | 4.197 | 2 | 4 | 8 | gene silencing by miRNA |
| GO:0035886 | 0.050 | 4.197 | 2 | 4 | 8 | vascular smooth muscle cell differentiation |
| GO:0045910 | 0.050 | 4.197 | 2 | 4 | 8 | negative regulation of DNA recombination |
| GO:0051131 | 0.050 | 4.197 | 2 | 4 | 8 | chaperone-mediated protein complex assembly |
| GO:0060252 | 0.050 | 4.197 | 2 | 4 | 8 | positive regulation of glial cell proliferation |
| GO:0061029 | 0.050 | 4.197 | 2 | 4 | 8 | eyelid development in camera-type eye |
| GO:0031570 | 0.050 | 6.446 | 1 | 3 | 5 | DNA integrity checkpoint |
