# Supplementary material for: Hidden Treasures in “Ancient” Microarrays: Gene-Expression Portrays Biology and Potential Resistance Pathways of Major Lung Cancer Subtypes and Normal Tissue
Source: Front Oncol. 2014 Sep 29;4:251. doi: 10.3389/fonc.2014.00251 (PMC4178426; doi:10.3389/fonc.2014.00251)
Supplement: Figure S1 — Clinically relevant differentially expressed pathways in each tissue type versus the rest. The pathway maps are arranged according to the scheme in the left-top corner. Red indicates overexpression in genes, dark green downregulation. In the Cell Cycle pathway, tumor suppressor genes were overexpressed in normal lung tissue while oncogenes and tumor driving cyclins were overexpressed in cancers. The gene thymidylate synthase (TYMS or TS, belonging to the pathway “one carbon pool by folate”) is relevant for tumor growth and is also a treatment target. Notably, TYMS was overexpressed only in the tumors that are generally refractory to the drug pemetrexed, as the squamous and the small-cell lung cancer. TYMS was not overexpressed in mesothelioma, but it is known that TYMS expression is highly variable in this cancer. In the ERBB pathway, the ERBB2/HER2 and ERBB3/HER3 were overexpressed in adenocarcinoma, while the ERBB4 was overexpressed in the carcinoids. [file Data_Sheet_1.ZIP › File S3.ZIP!/GO Analysis/Squamous vs Rest Enrichment Analysis/Underrepresented GO terms of Differentially Expressed Genes.html]

Gene to GO BP Conditional test for under-representation

| GOBPID | Pvalue | OddsRatio | ExpCount | Count | Size | Term |
| --- | --- | --- | --- | --- | --- | --- |
| GO:0034340 | <0.0001 | 0.293 | 26 | 11 | 61 | response to type I interferon |
| GO:0071345 | <0.0001 | 0.625 | 139 | 105 | 326 | cellular response to cytokine stimulus |
| GO:0043550 | <0.0001 | 0.154 | 12 | 3 | 29 | regulation of lipid kinase activity |
| GO:0007166 | <0.0001 | 0.823 | 711 | 649 | 1664 | cell surface receptor signaling pathway |
| GO:0032760 | <0.0001 | 0.127 | 10 | 2 | 23 | positive regulation of tumor necrosis factor production |
| GO:0060337 | <0.0001 | 0.229 | 15 | 5 | 34 | type I interferon-mediated signaling pathway |
| GO:0006586 | 0.001 | <0.0001 | 6 | 0 | 13 | indolalkylamine metabolic process |
| GO:0032755 | 0.002 | 0.191 | 10 | 3 | 24 | positive regulation of interleukin-6 production |
| GO:0050829 | 0.002 | 0.089 | 7 | 1 | 16 | defense response to Gram-negative bacterium |
| GO:0002822 | 0.002 | 0.435 | 28 | 16 | 65 | regulation of adaptive immune response based on somatic recombination of immune receptors built from immunoglobulin superfamily domains |
| GO:0070887 | 0.002 | 0.838 | 562 | 515 | 1315 | cellular response to chemical stimulus |
| GO:0002763 | 0.002 | 0.267 | 13 | 5 | 30 | positive regulation of myeloid leukocyte differentiation |
| GO:0051607 | 0.003 | 0.584 | 56 | 40 | 131 | defense response to virus |
| GO:0035455 | 0.003 | 0.095 | 6 | 1 | 15 | response to interferon-alpha |
| GO:0050691 | 0.003 | 0.095 | 6 | 1 | 15 | regulation of defense response to virus by host |
| GO:0032845 | 0.004 | 0.211 | 9 | 3 | 22 | negative regulation of homeostatic process |
| GO:0043552 | 0.004 | 0.211 | 9 | 3 | 22 | positive regulation of phosphatidylinositol 3-kinase activity |
| GO:0042402 | 0.004 | <0.0001 | 4 | 0 | 10 | cellular biogenic amine catabolic process |
| GO:0072182 | 0.004 | <0.0001 | 4 | 0 | 10 | regulation of nephron tubule epithelial cell differentiation |
| GO:0043123 | 0.004 | 0.548 | 41 | 28 | 96 | positive regulation of I-kappaB kinase/NF-kappaB cascade |
| GO:0035637 | 0.005 | 0.790 | 244 | 214 | 571 | multicellular organismal signaling |
| GO:0045671 | 0.005 | 0.103 | 6 | 1 | 14 | negative regulation of osteoclast differentiation |
| GO:0055003 | 0.005 | 0.103 | 6 | 1 | 14 | cardiac myofibril assembly |
| GO:0050776 | 0.005 | 0.753 | 165 | 140 | 385 | regulation of immune response |
| GO:0006661 | 0.005 | 0.497 | 30 | 19 | 70 | phosphatidylinositol biosynthetic process |
| GO:0045619 | 0.005 | 0.528 | 35 | 23 | 81 | regulation of lymphocyte differentiation |
| GO:0002696 | 0.005 | 0.664 | 78 | 61 | 183 | positive regulation of leukocyte activation |
| GO:0009072 | 0.006 | 0.223 | 9 | 3 | 21 | aromatic amino acid family metabolic process |
| GO:0007243 | 0.006 | 0.807 | 279 | 248 | 653 | intracellular protein kinase cascade |
| GO:0050877 | 0.006 | 0.825 | 355 | 321 | 832 | neurological system process |
| GO:0034341 | 0.006 | 0.550 | 38 | 26 | 89 | response to interferon-gamma |
| GO:0002685 | 0.006 | 0.518 | 32 | 21 | 75 | regulation of leukocyte migration |
| GO:0051707 | 0.006 | 0.767 | 177 | 152 | 414 | response to other organism |
| GO:0035914 | 0.006 | 0.404 | 18 | 10 | 43 | skeletal muscle cell differentiation |
| GO:0006952 | 0.007 | 0.807 | 270 | 240 | 627 | defense response |
| GO:0006955 | 0.008 | 0.768 | 168 | 145 | 390 | immune response |
| GO:0002828 | 0.009 | 0.236 | 9 | 3 | 20 | regulation of type 2 immune response |
| GO:0030947 | 0.009 | 0.236 | 9 | 3 | 20 | regulation of vascular endothelial growth factor receptor signaling pathway |
| GO:0006612 | 0.009 | 0.604 | 47 | 34 | 109 | protein targeting to membrane |
| GO:0045184 | 0.010 | 0.838 | 356 | 324 | 833 | establishment of protein localization |
| GO:0072599 | 0.011 | 0.558 | 33 | 23 | 78 | establishment of protein localization to endoplasmic reticulum |
| GO:0002690 | 0.011 | 0.458 | 20 | 12 | 47 | positive regulation of leukocyte chemotaxis |
| GO:0006614 | 0.012 | 0.554 | 32 | 22 | 75 | SRP-dependent cotranslational protein targeting to membrane |
| GO:0048584 | 0.012 | 0.837 | 332 | 302 | 775 | positive regulation of response to stimulus |
| GO:0002676 | 0.012 | <0.0001 | 3 | 0 | 8 | regulation of chronic inflammatory response |
| GO:0007158 | 0.012 | <0.0001 | 3 | 0 | 8 | neuron cell-cell adhesion |
| GO:0045625 | 0.012 | <0.0001 | 3 | 0 | 8 | regulation of T-helper 1 cell differentiation |
| GO:0023056 | 0.012 | 0.824 | 271 | 244 | 635 | positive regulation of signaling |
| GO:0010830 | 0.012 | 0.122 | 5 | 1 | 12 | regulation of myotube differentiation |
| GO:0022037 | 0.012 | 0.508 | 25 | 16 | 58 | metencephalon development |
| GO:0050793 | 0.013 | 0.858 | 443 | 410 | 1038 | regulation of developmental process |
| GO:0007268 | 0.013 | 0.796 | 189 | 166 | 441 | synaptic transmission |
| GO:0048520 | 0.014 | 0.568 | 33 | 23 | 77 | positive regulation of behavior |
| GO:0010033 | 0.014 | 0.878 | 629 | 591 | 1472 | response to organic substance |
| GO:0002792 | 0.014 | 0.297 | 9 | 4 | 22 | negative regulation of peptide secretion |
| GO:0006886 | 0.014 | 0.809 | 213 | 189 | 498 | intracellular protein transport |
| GO:0044255 | 0.015 | 0.813 | 222 | 198 | 520 | cellular lipid metabolic process |
| GO:0002548 | 0.015 | 0.334 | 11 | 5 | 25 | monocyte chemotaxis |
| GO:0006414 | 0.015 | 0.560 | 30 | 21 | 71 | translational elongation |
| GO:0060135 | 0.015 | 0.445 | 17 | 10 | 40 | maternal process involved in female pregnancy |
| GO:0035710 | 0.016 | 0.429 | 16 | 9 | 37 | CD4-positive, alpha-beta T cell activation |
| GO:0010647 | 0.016 | 0.830 | 269 | 243 | 629 | positive regulation of cell communication |
| GO:0002700 | 0.018 | 0.505 | 22 | 14 | 51 | regulation of production of molecular mediator of immune response |
| GO:0006968 | 0.018 | 0.496 | 21 | 13 | 48 | cellular defense response |
| GO:0045582 | 0.018 | 0.496 | 21 | 13 | 48 | positive regulation of T cell differentiation |
| GO:0042088 | 0.019 | 0.267 | 8 | 3 | 18 | T-helper 1 type immune response |
| GO:0002532 | 0.019 | 0.267 | 8 | 3 | 18 | production of molecular mediator involved in inflammatory response |
| GO:0061180 | 0.019 | 0.540 | 25 | 17 | 59 | mammary gland epithelium development |
| GO:0006355 | 0.020 | 0.889 | 693 | 656 | 1622 | regulation of transcription, DNA-dependent |
| GO:0044706 | 0.020 | 0.703 | 69 | 56 | 162 | multi-multicellular organism process |
| GO:0002719 | 0.020 | 0.134 | 5 | 1 | 11 | negative regulation of cytokine production involved in immune response |
| GO:0006837 | 0.020 | 0.134 | 5 | 1 | 11 | serotonin transport |
| GO:0007004 | 0.020 | 0.134 | 5 | 1 | 11 | telomere maintenance via telomerase |
| GO:0046621 | 0.020 | 0.134 | 5 | 1 | 11 | negative regulation of organ growth |
| GO:0051044 | 0.020 | 0.134 | 5 | 1 | 11 | positive regulation of membrane protein ectodomain proteolysis |
| GO:2000058 | 0.020 | 0.134 | 5 | 1 | 11 | regulation of protein ubiquitination involved in ubiquitin-dependent protein catabolic process |
| GO:0033762 | 0.020 | <0.0001 | 3 | 0 | 7 | response to glucagon stimulus |
| GO:0001886 | 0.020 | <0.0001 | 3 | 0 | 7 | endothelial cell morphogenesis |
| GO:0002480 | 0.020 | <0.0001 | 3 | 0 | 7 | antigen processing and presentation of exogenous peptide antigen via MHC class I, TAP-independent |
| GO:0002827 | 0.020 | <0.0001 | 3 | 0 | 7 | positive regulation of T-helper 1 type immune response |
| GO:0006569 | 0.020 | <0.0001 | 3 | 0 | 7 | tryptophan catabolic process |
| GO:0014866 | 0.020 | <0.0001 | 3 | 0 | 7 | skeletal myofibril assembly |
| GO:0031065 | 0.020 | <0.0001 | 3 | 0 | 7 | positive regulation of histone deacetylation |
| GO:0042375 | 0.020 | <0.0001 | 3 | 0 | 7 | quinone cofactor metabolic process |
| GO:0042436 | 0.020 | <0.0001 | 3 | 0 | 7 | indole-containing compound catabolic process |
| GO:0051187 | 0.020 | <0.0001 | 3 | 0 | 7 | cofactor catabolic process |
| GO:0070472 | 0.020 | <0.0001 | 3 | 0 | 7 | regulation of uterine smooth muscle contraction |
| GO:1901661 | 0.020 | <0.0001 | 3 | 0 | 7 | quinone metabolic process |
| GO:1901881 | 0.020 | <0.0001 | 3 | 0 | 7 | positive regulation of protein depolymerization |
| GO:0051347 | 0.020 | 0.787 | 146 | 127 | 341 | positive regulation of transferase activity |
| GO:0050830 | 0.021 | 0.315 | 9 | 4 | 21 | defense response to Gram-positive bacterium |
| GO:0050863 | 0.021 | 0.717 | 76 | 62 | 177 | regulation of T cell activation |
| GO:0043500 | 0.021 | 0.445 | 15 | 9 | 36 | muscle adaptation |
| GO:0050673 | 0.021 | 0.380 | 12 | 6 | 27 | epithelial cell proliferation |
| GO:0046425 | 0.021 | 0.526 | 23 | 15 | 53 | regulation of JAK-STAT cascade |
| GO:0006650 | 0.022 | 0.694 | 62 | 50 | 146 | glycerophospholipid metabolic process |
| GO:0046395 | 0.022 | 0.694 | 62 | 50 | 146 | carboxylic acid catabolic process |
| GO:0048013 | 0.022 | 0.352 | 10 | 5 | 24 | ephrin receptor signaling pathway |
| GO:0006901 | 0.022 | 0.382 | 12 | 6 | 27 | vesicle coating |
| GO:0060338 | 0.022 | 0.382 | 12 | 6 | 27 | regulation of type I interferon-mediated signaling pathway |
| GO:0002521 | 0.023 | 0.767 | 113 | 97 | 265 | leukocyte differentiation |
| GO:0050729 | 0.023 | 0.559 | 26 | 18 | 61 | positive regulation of inflammatory response |
| GO:0002576 | 0.025 | 0.604 | 33 | 24 | 77 | platelet degranulation |
| GO:0045834 | 0.025 | 0.604 | 33 | 24 | 77 | positive regulation of lipid metabolic process |
| GO:0070665 | 0.025 | 0.604 | 33 | 24 | 77 | positive regulation of leukocyte proliferation |
| GO:0048193 | 0.025 | 0.666 | 49 | 38 | 114 | Golgi vesicle transport |
| GO:0007617 | 0.025 | 0.223 | 6 | 2 | 14 | mating behavior |
| GO:0031290 | 0.025 | 0.223 | 6 | 2 | 14 | retinal ganglion cell axon guidance |
| GO:0044766 | 0.025 | 0.223 | 6 | 2 | 14 | multi-organism transport |
| GO:0034754 | 0.026 | 0.580 | 28 | 20 | 66 | cellular hormone metabolic process |
| GO:0050671 | 0.026 | 0.602 | 32 | 23 | 74 | positive regulation of lymphocyte proliferation |
| GO:0060759 | 0.026 | 0.602 | 32 | 23 | 74 | regulation of response to cytokine stimulus |
| GO:0032642 | 0.028 | 0.477 | 16 | 10 | 38 | regulation of chemokine production |
| GO:0050803 | 0.028 | 0.477 | 16 | 10 | 38 | regulation of synapse structure and activity |
| GO:0046676 | 0.028 | 0.287 | 7 | 3 | 17 | negative regulation of insulin secretion |
| GO:0051930 | 0.028 | 0.287 | 7 | 3 | 17 | regulation of sensory perception of pain |
| GO:0023052 | 0.029 | 0.914 | 1343 | 1302 | 3144 | signaling |
| GO:0043534 | 0.029 | 0.534 | 21 | 14 | 49 | blood vessel endothelial cell migration |
| GO:0031032 | 0.030 | 0.446 | 14 | 8 | 32 | actomyosin structure organization |
| GO:0032774 | 0.030 | 0.902 | 796 | 760 | 1862 | RNA biosynthetic process |
| GO:0002293 | 0.030 | 0.425 | 12 | 7 | 29 | alpha-beta T cell differentiation involved in immune response |
| GO:0043370 | 0.031 | 0.371 | 10 | 5 | 23 | regulation of CD4-positive, alpha-beta T cell differentiation |
| GO:0071377 | 0.031 | 0.401 | 11 | 6 | 26 | cellular response to glucagon stimulus |
| GO:0009108 | 0.031 | 0.568 | 24 | 17 | 57 | coenzyme biosynthetic process |
| GO:0070972 | 0.031 | 0.148 | 4 | 1 | 10 | protein localization to endoplasmic reticulum |
| GO:0032733 | 0.032 | 0.149 | 4 | 1 | 10 | positive regulation of interleukin-10 production |
| GO:0034374 | 0.032 | 0.149 | 4 | 1 | 10 | low-density lipoprotein particle remodeling |
| GO:0035112 | 0.032 | 0.149 | 4 | 1 | 10 | genitalia morphogenesis |
| GO:0042976 | 0.032 | 0.149 | 4 | 1 | 10 | activation of Janus kinase activity |
| GO:0014068 | 0.033 | 0.517 | 18 | 12 | 43 | positive regulation of phosphatidylinositol 3-kinase cascade |
| GO:0031326 | 0.033 | 0.906 | 832 | 797 | 1948 | regulation of cellular biosynthetic process |
| GO:0046649 | 0.034 | 0.812 | 154 | 137 | 361 | lymphocyte activation |
| GO:0021587 | 0.035 | <0.0001 | 3 | 0 | 6 | cerebellum morphogenesis |
| GO:0002579 | 0.035 | <0.0001 | 3 | 0 | 6 | positive regulation of antigen processing and presentation |
| GO:0006703 | 0.035 | <0.0001 | 3 | 0 | 6 | estrogen biosynthetic process |
| GO:0007217 | 0.035 | <0.0001 | 3 | 0 | 6 | tachykinin receptor signaling pathway |
| GO:0008535 | 0.035 | <0.0001 | 3 | 0 | 6 | respiratory chain complex IV assembly |
| GO:0016239 | 0.035 | <0.0001 | 3 | 0 | 6 | positive regulation of macroautophagy |
| GO:0021561 | 0.035 | <0.0001 | 3 | 0 | 6 | facial nerve development |
| GO:0021604 | 0.035 | <0.0001 | 3 | 0 | 6 | cranial nerve structural organization |
| GO:0032610 | 0.035 | <0.0001 | 3 | 0 | 6 | interleukin-1 alpha production |
| GO:0038034 | 0.035 | <0.0001 | 3 | 0 | 6 | signal transduction in absence of ligand |
| GO:0042501 | 0.035 | <0.0001 | 3 | 0 | 6 | serine phosphorylation of STAT protein |
| GO:0045924 | 0.035 | <0.0001 | 3 | 0 | 6 | regulation of female receptivity |
| GO:0048752 | 0.035 | <0.0001 | 3 | 0 | 6 | semicircular canal morphogenesis |
| GO:0070189 | 0.035 | <0.0001 | 3 | 0 | 6 | kynurenine metabolic process |
| GO:0071875 | 0.035 | <0.0001 | 3 | 0 | 6 | adrenergic receptor signaling pathway |
| GO:2001239 | 0.035 | <0.0001 | 3 | 0 | 6 | regulation of extrinsic apoptotic signaling pathway in absence of ligand |
| GO:0051252 | 0.036 | 0.903 | 718 | 685 | 1680 | regulation of RNA metabolic process |
| GO:0040017 | 0.036 | 0.762 | 88 | 75 | 206 | positive regulation of locomotion |
| GO:0002706 | 0.037 | 0.586 | 25 | 18 | 59 | regulation of lymphocyte mediated immunity |
| GO:0007260 | 0.037 | 0.550 | 21 | 14 | 48 | tyrosine phosphorylation of STAT protein |
| GO:0045453 | 0.038 | 0.481 | 15 | 9 | 34 | bone resorption |
| GO:0044130 | 0.038 | 0.243 | 6 | 2 | 13 | negative regulation of growth of symbiont in host |
| GO:0044144 | 0.038 | 0.243 | 6 | 2 | 13 | modulation of growth of symbiont involved in interaction with host |
| GO:0046641 | 0.038 | 0.243 | 6 | 2 | 13 | positive regulation of alpha-beta T cell proliferation |
| GO:0071391 | 0.038 | 0.243 | 6 | 2 | 13 | cellular response to estrogen stimulus |
| GO:0055074 | 0.039 | 0.767 | 89 | 76 | 208 | calcium ion homeostasis |
| GO:0050678 | 0.039 | 0.737 | 68 | 57 | 160 | regulation of epithelial cell proliferation |
| GO:0071363 | 0.039 | 0.817 | 150 | 134 | 352 | cellular response to growth factor stimulus |
| GO:0006415 | 0.040 | 0.607 | 27 | 20 | 64 | translational termination |
| GO:0050663 | 0.040 | 0.607 | 27 | 20 | 64 | cytokine secretion |
| GO:0043901 | 0.040 | 0.543 | 19 | 13 | 45 | negative regulation of multi-organism process |
| GO:0051649 | 0.041 | 0.895 | 537 | 509 | 1258 | establishment of localization in cell |
| GO:0042093 | 0.041 | 0.446 | 12 | 7 | 28 | T-helper cell differentiation |
| GO:0071378 | 0.041 | 0.446 | 12 | 7 | 28 | cellular response to growth hormone stimulus |
| GO:0002429 | 0.042 | 0.677 | 42 | 33 | 98 | immune response-activating cell surface receptor signaling pathway |
| GO:0007184 | 0.042 | 0.309 | 7 | 3 | 16 | SMAD protein import into nucleus |
| GO:0010556 | 0.042 | 0.910 | 790 | 757 | 1848 | regulation of macromolecule biosynthetic process |
| GO:0019438 | 0.042 | 0.914 | 907 | 873 | 2123 | aromatic compound biosynthetic process |
| GO:0002456 | 0.042 | 0.535 | 18 | 12 | 42 | T cell mediated immunity |
| GO:0045638 | 0.042 | 0.535 | 18 | 12 | 42 | negative regulation of myeloid cell differentiation |
| GO:0007257 | 0.042 | 0.422 | 11 | 6 | 25 | activation of JUN kinase activity |
| GO:0018279 | 0.043 | 0.604 | 26 | 19 | 61 | protein N-linked glycosylation via asparagine |
| GO:0060333 | 0.043 | 0.604 | 26 | 19 | 61 | interferon-gamma-mediated signaling pathway |
| GO:0006700 | 0.043 | 0.357 | 8 | 4 | 19 | C21-steroid hormone biosynthetic process |
| GO:0021696 | 0.043 | 0.357 | 8 | 4 | 19 | cerebellar cortex morphogenesis |
| GO:0032735 | 0.043 | 0.357 | 8 | 4 | 19 | positive regulation of interleukin-12 production |
| GO:0034368 | 0.043 | 0.357 | 8 | 4 | 19 | protein-lipid complex remodeling |
| GO:0048260 | 0.043 | 0.357 | 8 | 4 | 19 | positive regulation of receptor-mediated endocytosis |
| GO:0001912 | 0.043 | 0.393 | 9 | 5 | 22 | positive regulation of leukocyte mediated cytotoxicity |
| GO:0060397 | 0.043 | 0.393 | 9 | 5 | 22 | JAK-STAT cascade involved in growth hormone signaling pathway |
| GO:0014706 | 0.044 | 0.788 | 103 | 90 | 242 | striated muscle tissue development |
| GO:0072503 | 0.044 | 0.773 | 88 | 76 | 207 | cellular divalent inorganic cation homeostasis |
| GO:0045860 | 0.045 | 0.814 | 135 | 120 | 316 | positive regulation of protein kinase activity |
| GO:0019886 | 0.046 | 0.623 | 28 | 21 | 66 | antigen processing and presentation of exogenous peptide antigen via MHC class II |
| GO:0030335 | 0.046 | 0.769 | 83 | 71 | 194 | positive regulation of cell migration |
| GO:0050865 | 0.047 | 0.806 | 120 | 106 | 281 | regulation of cell activation |
| GO:0002821 | 0.047 | 0.514 | 15 | 10 | 36 | positive regulation of adaptive immune response |
| GO:0033143 | 0.047 | 0.514 | 15 | 10 | 36 | regulation of intracellular steroid hormone receptor signaling pathway |
| GO:0031294 | 0.049 | 0.598 | 23 | 17 | 55 | lymphocyte costimulation |
| GO:0055007 | 0.049 | 0.598 | 23 | 17 | 55 | cardiac muscle cell differentiation |
| GO:0071706 | 0.049 | 0.598 | 23 | 17 | 55 | tumor necrosis factor superfamily cytokine production |
| GO:1901362 | 0.049 | 0.918 | 942 | 909 | 2205 | organic cyclic compound biosynthetic process |
