# Supplementary material for: Hidden Treasures in “Ancient” Microarrays: Gene-Expression Portrays Biology and Potential Resistance Pathways of Major Lung Cancer Subtypes and Normal Tissue
Source: Front Oncol. 2014 Sep 29;4:251. doi: 10.3389/fonc.2014.00251 (PMC4178426; doi:10.3389/fonc.2014.00251)
Supplement: Figure S1 — Clinically relevant differentially expressed pathways in each tissue type versus the rest. The pathway maps are arranged according to the scheme in the left-top corner. Red indicates overexpression in genes, dark green downregulation. In the Cell Cycle pathway, tumor suppressor genes were overexpressed in normal lung tissue while oncogenes and tumor driving cyclins were overexpressed in cancers. The gene thymidylate synthase (TYMS or TS, belonging to the pathway “one carbon pool by folate”) is relevant for tumor growth and is also a treatment target. Notably, TYMS was overexpressed only in the tumors that are generally refractory to the drug pemetrexed, as the squamous and the small-cell lung cancer. TYMS was not overexpressed in mesothelioma, but it is known that TYMS expression is highly variable in this cancer. In the ERBB pathway, the ERBB2/HER2 and ERBB3/HER3 were overexpressed in adenocarcinoma, while the ERBB4 was overexpressed in the carcinoids. [file Data_Sheet_1.ZIP › File S3.ZIP!/GO Analysis/Squamous vs Rest Enrichment Analysis/Underrepresented GO terms of Down-regulated Genes.html]

Gene to GO BP Conditional test for under-representation

| GOBPID | Pvalue | OddsRatio | ExpCount | Count | Size | Term |
| --- | --- | --- | --- | --- | --- | --- |
| GO:0090304 | <0.0001 | 0.574 | 583 | 425 | 2488 | nucleic acid metabolic process |
| GO:0000278 | <0.0001 | 0.406 | 118 | 58 | 502 | mitotic cell cycle |
| GO:0009058 | <0.0001 | 0.688 | 717 | 595 | 3056 | biosynthetic process |
| GO:0016071 | <0.0001 | 0.338 | 68 | 28 | 287 | mRNA metabolic process |
| GO:0051325 | <0.0001 | 0.323 | 63 | 25 | 270 | interphase |
| GO:0019438 | <0.0001 | 0.688 | 498 | 399 | 2123 | aromatic compound biosynthetic process |
| GO:0019058 | <0.0001 | 0.227 | 42 | 12 | 181 | viral infectious cycle |
| GO:0044271 | <0.0001 | 0.694 | 509 | 411 | 2170 | cellular nitrogen compound biosynthetic process |
| GO:0051252 | <0.0001 | 0.669 | 394 | 305 | 1680 | regulation of RNA metabolic process |
| GO:0044703 | <0.0001 | 0.513 | 140 | 84 | 596 | multi-organism reproductive process |
| GO:0018130 | <0.0001 | 0.695 | 496 | 400 | 2111 | heterocycle biosynthetic process |
| GO:1901362 | <0.0001 | 0.701 | 515 | 419 | 2193 | organic cyclic compound biosynthetic process |
| GO:0000216 | <0.0001 | <0.0001 | 16 | 0 | 70 | M/G1 transition of mitotic cell cycle |
| GO:0031145 | <0.0001 | <0.0001 | 16 | 0 | 70 | anaphase-promoting complex-dependent proteasomal ubiquitin-dependent protein catabolic process |
| GO:0051351 | <0.0001 | <0.0001 | 16 | 0 | 69 | positive regulation of ligase activity |
| GO:0006281 | <0.0001 | 0.341 | 58 | 24 | 248 | DNA repair |
| GO:0000375 | <0.0001 | 0.222 | 36 | 10 | 154 | RNA splicing, via transesterification reactions |
| GO:0046483 | <0.0001 | 0.623 | 251 | 184 | 999 | heterocycle metabolic process |
| GO:0051438 | <0.0001 | 0.042 | 18 | 1 | 78 | regulation of ubiquitin-protein ligase activity |
| GO:0032774 | <0.0001 | 0.693 | 413 | 329 | 1741 | RNA biosynthetic process |
| GO:0006413 | <0.0001 | 0.149 | 27 | 5 | 113 | translational initiation |
| GO:0044238 | <0.0001 | 0.702 | 599 | 514 | 2405 | primary metabolic process |
| GO:0007093 | <0.0001 | 0.153 | 26 | 5 | 110 | mitotic cell cycle checkpoint |
| GO:0051437 | <0.0001 | <0.0001 | 14 | 0 | 61 | positive regulation of ubiquitin-protein ligase activity involved in mitotic cell cycle |
| GO:0071840 | <0.0001 | 0.745 | 637 | 546 | 2717 | cellular component organization or biogenesis |
| GO:0006725 | <0.0001 | 0.649 | 255 | 192 | 1012 | cellular aromatic compound metabolic process |
| GO:0009987 | <0.0001 | 0.617 | 1286 | 1233 | 5240 | cellular process |
| GO:0016032 | <0.0001 | 0.512 | 104 | 62 | 441 | viral reproduction |
| GO:0051436 | <0.0001 | <0.0001 | 13 | 0 | 56 | negative regulation of ubiquitin-protein ligase activity involved in mitotic cell cycle |
| GO:0034641 | <0.0001 | 0.655 | 257 | 195 | 1013 | cellular nitrogen compound metabolic process |
| GO:0043632 | <0.0001 | 0.399 | 59 | 28 | 252 | modification-dependent macromolecule catabolic process |
| GO:0034645 | <0.0001 | 0.727 | 460 | 381 | 1894 | cellular macromolecule biosynthetic process |
| GO:0000184 | <0.0001 | 0.119 | 20 | 3 | 84 | nuclear-transcribed mRNA catabolic process, nonsense-mediated decay |
| GO:0006614 | <0.0001 | 0.088 | 18 | 2 | 75 | SRP-dependent cotranslational protein targeting to membrane |
| GO:0044260 | <0.0001 | 0.499 | 93 | 56 | 347 | cellular macromolecule metabolic process |
| GO:0051171 | <0.0001 | 0.738 | 486 | 406 | 2072 | regulation of nitrogen compound metabolic process |
| GO:0006415 | <0.0001 | 0.051 | 15 | 1 | 64 | translational termination |
| GO:0000279 | <0.0001 | 0.463 | 74 | 40 | 315 | M phase |
| GO:0019083 | <0.0001 | 0.090 | 17 | 2 | 73 | viral transcription |
| GO:0000003 | <0.0001 | 0.677 | 259 | 198 | 1106 | reproduction |
| GO:0031571 | <0.0001 | 0.052 | 15 | 1 | 63 | mitotic cell cycle G1/S transition DNA damage checkpoint |
| GO:0072395 | <0.0001 | 0.053 | 15 | 1 | 62 | signal transduction involved in cell cycle checkpoint |
| GO:0031397 | <0.0001 | 0.126 | 19 | 3 | 80 | negative regulation of protein ubiquitination |
| GO:0006414 | <0.0001 | 0.094 | 17 | 2 | 71 | translational elongation |
| GO:1901360 | <0.0001 | 0.679 | 270 | 211 | 1073 | organic cyclic compound metabolic process |
| GO:0051352 | <0.0001 | 0.054 | 14 | 1 | 61 | negative regulation of ligase activity |
| GO:0072422 | <0.0001 | 0.054 | 14 | 1 | 61 | signal transduction involved in DNA damage checkpoint |
| GO:0010564 | <0.0001 | 0.345 | 41 | 17 | 173 | regulation of cell cycle process |
| GO:0000084 | <0.0001 | 0.254 | 29 | 9 | 123 | S phase of mitotic cell cycle |
| GO:0006977 | <0.0001 | 0.055 | 14 | 1 | 60 | DNA damage response, signal transduction by p53 class mediator resulting in cell cycle arrest |
| GO:0072474 | <0.0001 | 0.055 | 14 | 1 | 60 | signal transduction involved in mitotic cell cycle G1/S checkpoint |
| GO:0072599 | <0.0001 | 0.129 | 18 | 3 | 78 | establishment of protein localization to endoplasmic reticulum |
| GO:0000398 | <0.0001 | 0.269 | 30 | 10 | 129 | mRNA splicing, via spliceosome |
| GO:0072331 | <0.0001 | 0.223 | 25 | 7 | 108 | signal transduction by p53 class mediator |
| GO:0031398 | <0.0001 | 0.206 | 23 | 6 | 100 | positive regulation of protein ubiquitination |
| GO:0006402 | <0.0001 | 0.259 | 28 | 9 | 121 | mRNA catabolic process |
| GO:0009615 | <0.0001 | 0.098 | 16 | 2 | 67 | response to virus |
| GO:0002474 | <0.0001 | 0.161 | 20 | 4 | 84 | antigen processing and presentation of peptide antigen via MHC class I |
| GO:0010556 | <0.0001 | 0.703 | 286 | 226 | 1189 | regulation of macromolecule biosynthetic process |
| GO:0010498 | <0.0001 | 0.345 | 39 | 16 | 165 | proteasomal protein catabolic process |
| GO:0030163 | <0.0001 | 0.425 | 54 | 27 | 229 | protein catabolic process |
| GO:0000236 | <0.0001 | <0.0001 | 11 | 0 | 46 | mitotic prometaphase |
| GO:2000045 | <0.0001 | 0.166 | 19 | 4 | 82 | regulation of G1/S transition of mitotic cell cycle |
| GO:0071779 | <0.0001 | 0.137 | 17 | 3 | 74 | G1/S transition checkpoint |
| GO:0002478 | <0.0001 | 0.102 | 15 | 2 | 65 | antigen processing and presentation of exogenous peptide antigen |
| GO:0033554 | <0.0001 | 0.630 | 153 | 109 | 648 | cellular response to stress |
| GO:0002479 | <0.0001 | 0.104 | 15 | 2 | 64 | antigen processing and presentation of exogenous peptide antigen via MHC class I, TAP-dependent |
| GO:0016569 | <0.0001 | 0.394 | 43 | 20 | 183 | covalent chromatin modification |
| GO:0006511 | <0.0001 | 0.423 | 48 | 24 | 205 | ubiquitin-dependent protein catabolic process |
| GO:0033365 | <0.0001 | 0.537 | 83 | 51 | 353 | protein localization to organelle |
| GO:0048285 | <0.0001 | 0.455 | 55 | 29 | 233 | organelle fission |
| GO:0032446 | <0.0001 | 0.517 | 72 | 43 | 308 | protein modification by small protein conjugation |
| GO:0044265 | <0.0001 | 0.440 | 50 | 26 | 211 | cellular macromolecule catabolic process |
| GO:0000209 | <0.0001 | 0.265 | 25 | 8 | 105 | protein polyubiquitination |
| GO:0000082 | <0.0001 | 0.228 | 21 | 6 | 90 | G1/S transition of mitotic cell cycle |
| GO:0090068 | <0.0001 | 0.340 | 32 | 13 | 136 | positive regulation of cell cycle process |
| GO:0010558 | <0.0001 | 0.654 | 155 | 113 | 659 | negative regulation of macromolecule biosynthetic process |
| GO:0045786 | <0.0001 | 0.527 | 71 | 43 | 303 | negative regulation of cell cycle |
| GO:0080090 | <0.0001 | 0.795 | 610 | 540 | 2587 | regulation of primary metabolic process |
| GO:0031327 | <0.0001 | 0.673 | 162 | 121 | 690 | negative regulation of cellular biosynthetic process |
| GO:0045934 | <0.0001 | 0.663 | 150 | 111 | 641 | negative regulation of nucleobase-containing compound metabolic process |
| GO:0006325 | <0.0001 | 0.507 | 60 | 35 | 255 | chromatin organization |
| GO:0051704 | <0.0001 | 0.687 | 178 | 136 | 748 | multi-organism process |
| GO:0070727 | <0.0001 | 0.671 | 155 | 116 | 663 | cellular macromolecule localization |
| GO:0031326 | <0.0001 | 0.748 | 302 | 250 | 1258 | regulation of cellular biosynthetic process |
| GO:0050792 | <0.0001 | 0.290 | 23 | 8 | 97 | regulation of viral reproduction |
| GO:0019882 | <0.0001 | 0.292 | 23 | 8 | 96 | antigen processing and presentation |
| GO:0000075 | <0.0001 | 0.205 | 16 | 4 | 66 | cell cycle checkpoint |
| GO:0071824 | <0.0001 | 0.283 | 20 | 7 | 87 | protein-DNA complex subunit organization |
| GO:0034622 | <0.0001 | 0.594 | 81 | 54 | 345 | cellular macromolecular complex assembly |
| GO:0034660 | <0.0001 | 0.265 | 19 | 6 | 79 | ncRNA metabolic process |
| GO:0006355 | <0.0001 | 0.647 | 113 | 82 | 466 | regulation of transcription, DNA-dependent |
| GO:0050657 | <0.0001 | 0.290 | 20 | 7 | 85 | nucleic acid transport |
| GO:0051236 | <0.0001 | 0.290 | 20 | 7 | 85 | establishment of RNA localization |
| GO:0006612 | <0.0001 | 0.363 | 26 | 11 | 109 | protein targeting to membrane |
| GO:0009892 | <0.0001 | 0.749 | 238 | 195 | 1016 | negative regulation of metabolic process |
| GO:0071826 | <0.0001 | 0.249 | 16 | 5 | 70 | ribonucleoprotein complex subunit organization |
| GO:0006323 | <0.0001 | 0.281 | 18 | 6 | 75 | DNA packaging |
| GO:0043412 | <0.0001 | 0.800 | 392 | 341 | 1671 | macromolecule modification |
| GO:0006368 | 0.001 | 0.261 | 16 | 5 | 67 | transcription elongation from RNA polymerase II promoter |
| GO:0006521 | 0.001 | 0.195 | 12 | 3 | 53 | regulation of cellular amino acid metabolic process |
| GO:0044403 | 0.001 | 0.632 | 85 | 60 | 364 | symbiosis, encompassing mutualism through parasitism |
| GO:0006310 | 0.001 | 0.381 | 24 | 11 | 104 | DNA recombination |
| GO:0019048 | 0.001 | 0.607 | 71 | 48 | 302 | virus-host interaction |
| GO:0016482 | 0.001 | 0.646 | 91 | 65 | 383 | cytoplasmic transport |
| GO:0006996 | 0.001 | 0.778 | 268 | 226 | 1123 | organelle organization |
| GO:0051301 | 0.001 | 0.569 | 55 | 35 | 232 | cell division |
| GO:0006369 | 0.001 | 0.096 | 8 | 1 | 35 | termination of RNA polymerase II transcription |
| GO:0051168 | 0.001 | 0.369 | 21 | 9 | 88 | nuclear export |
| GO:0006605 | 0.002 | 0.592 | 56 | 37 | 236 | protein targeting |
| GO:0006364 | 0.002 | 0.254 | 13 | 4 | 55 | rRNA processing |
| GO:0051028 | 0.002 | 0.101 | 8 | 1 | 33 | mRNA transport |
| GO:0006417 | 0.002 | 0.489 | 32 | 18 | 137 | regulation of translation |
| GO:0034340 | 0.002 | 0.289 | 14 | 5 | 61 | response to type I interferon |
| GO:0060337 | 0.002 | 0.289 | 14 | 5 | 61 | type I interferon-mediated signaling pathway |
| GO:0040029 | 0.002 | 0.319 | 16 | 6 | 67 | regulation of gene expression, epigenetic |
| GO:0043241 | 0.002 | 0.453 | 27 | 14 | 114 | protein complex disassembly |
| GO:0071230 | 0.002 | <0.0001 | 5 | 0 | 23 | cellular response to amino acid stimulus |
| GO:0042981 | 0.002 | 0.778 | 210 | 176 | 895 | regulation of apoptotic process |
| GO:0001101 | 0.002 | 0.349 | 17 | 7 | 72 | response to acid |
| GO:0000122 | 0.003 | 0.677 | 85 | 63 | 362 | negative regulation of transcription from RNA polymerase II promoter |
| GO:0032845 | 0.003 | <0.0001 | 5 | 0 | 22 | negative regulation of homeostatic process |
| GO:0009411 | 0.003 | 0.376 | 18 | 8 | 77 | response to UV |
| GO:0012501 | 0.003 | 0.803 | 267 | 230 | 1137 | programmed cell death |
| GO:0043901 | 0.003 | 0.232 | 11 | 3 | 45 | negative regulation of multi-organism process |
| GO:0006144 | 0.003 | 0.112 | 7 | 1 | 30 | purine nucleobase metabolic process |
| GO:0070848 | 0.003 | 0.685 | 85 | 64 | 364 | response to growth factor stimulus |
| GO:0031023 | 0.004 | 0.238 | 10 | 3 | 44 | microtubule organizing center organization |
| GO:0008380 | 0.004 | 0.312 | 13 | 5 | 56 | RNA splicing |
| GO:0009124 | 0.004 | 0.116 | 7 | 1 | 29 | nucleoside monophosphate biosynthetic process |
| GO:0036260 | 0.004 | 0.116 | 7 | 1 | 29 | RNA capping |
| GO:0043550 | 0.004 | 0.116 | 7 | 1 | 29 | regulation of lipid kinase activity |
| GO:2001251 | 0.004 | 0.116 | 7 | 1 | 29 | negative regulation of chromosome organization |
| GO:2000241 | 0.004 | 0.562 | 38 | 24 | 162 | regulation of reproductive process |
| GO:0008104 | 0.004 | 0.804 | 240 | 207 | 1025 | protein localization |
| GO:0016265 | 0.004 | 0.820 | 295 | 259 | 1259 | death |
| GO:0006283 | 0.005 | 0.244 | 10 | 3 | 43 | transcription-coupled nucleotide-excision repair |
| GO:0006357 | 0.005 | 0.743 | 126 | 101 | 529 | regulation of transcription from RNA polymerase II promoter |
| GO:0000387 | 0.005 | <0.0001 | 5 | 0 | 20 | spliceosomal snRNP assembly |
| GO:0007062 | 0.005 | <0.0001 | 5 | 0 | 20 | sister chromatid cohesion |
| GO:0006260 | 0.005 | 0.496 | 26 | 15 | 112 | DNA replication |
| GO:0043902 | 0.005 | 0.417 | 19 | 9 | 79 | positive regulation of multi-organism process |
| GO:0006370 | 0.005 | 0.120 | 7 | 1 | 28 | 7-methylguanosine mRNA capping |
| GO:0000086 | 0.006 | 0.501 | 26 | 15 | 112 | G2/M transition of mitotic cell cycle |
| GO:0006396 | 0.006 | 0.566 | 36 | 23 | 151 | RNA processing |
| GO:0010941 | 0.006 | 0.803 | 217 | 186 | 924 | regulation of cell death |
| GO:0071704 | 0.006 | 0.839 | 494 | 457 | 1860 | organic substance metabolic process |
| GO:0022613 | 0.006 | 0.328 | 13 | 5 | 54 | ribonucleoprotein complex biogenesis |
| GO:0016573 | 0.006 | 0.423 | 18 | 9 | 78 | histone acetylation |
| GO:0006464 | 0.006 | 0.839 | 356 | 319 | 1507 | cellular protein modification process |
| GO:0050434 | 0.006 | 0.295 | 11 | 4 | 48 | positive regulation of viral transcription |
| GO:0015031 | 0.006 | 0.793 | 189 | 160 | 804 | protein transport |
| GO:0019886 | 0.006 | 0.385 | 15 | 7 | 66 | antigen processing and presentation of exogenous peptide antigen via MHC class II |
| GO:0030071 | 0.007 | 0.125 | 6 | 1 | 27 | regulation of mitotic metaphase/anaphase transition |
| GO:0032508 | 0.007 | 0.125 | 6 | 1 | 27 | DNA duplex unwinding |
| GO:0071479 | 0.007 | 0.125 | 6 | 1 | 27 | cellular response to ionizing radiation |
| GO:0042787 | 0.007 | 0.256 | 10 | 3 | 41 | protein ubiquitination involved in ubiquitin-dependent protein catabolic process |
| GO:0000724 | 0.007 | 0.203 | 8 | 2 | 34 | double-strand break repair via homologous recombination |
| GO:0035601 | 0.007 | 0.203 | 8 | 2 | 34 | protein deacylation |
| GO:0006334 | 0.007 | 0.302 | 11 | 4 | 47 | nucleosome assembly |
| GO:0010604 | 0.008 | 0.837 | 318 | 283 | 1354 | positive regulation of macromolecule metabolic process |
| GO:0009168 | 0.008 | <0.0001 | 4 | 0 | 18 | purine ribonucleoside monophosphate biosynthetic process |
| GO:0022904 | 0.008 | 0.263 | 9 | 3 | 40 | respiratory electron transport chain |
| GO:0031577 | 0.009 | 0.130 | 6 | 1 | 26 | spindle checkpoint |
| GO:0051649 | 0.009 | 0.835 | 295 | 262 | 1258 | establishment of localization in cell |
| GO:0006333 | 0.009 | 0.419 | 16 | 8 | 70 | chromatin assembly or disassembly |
| GO:0018394 | 0.009 | 0.457 | 19 | 10 | 81 | peptidyl-lysine acetylation |
| GO:0048523 | 0.009 | 0.854 | 398 | 362 | 1666 | negative regulation of cellular process |
| GO:0006913 | 0.010 | 0.444 | 18 | 9 | 74 | nucleocytoplasmic transport |
| GO:0002825 | 0.011 | <0.0001 | 4 | 0 | 17 | regulation of T-helper 1 type immune response |
| GO:0031047 | 0.011 | <0.0001 | 4 | 0 | 17 | gene silencing by RNA |
| GO:0035020 | 0.011 | <0.0001 | 4 | 0 | 17 | regulation of Rac protein signal transduction |
| GO:0006270 | 0.011 | 0.136 | 6 | 1 | 25 | DNA replication initiation |
| GO:0032651 | 0.011 | 0.136 | 6 | 1 | 25 | regulation of interleukin-1 beta production |
| GO:0045071 | 0.011 | 0.136 | 6 | 1 | 25 | negative regulation of viral genome replication |
| GO:0006479 | 0.011 | 0.317 | 11 | 4 | 45 | protein methylation |
| GO:0034655 | 0.011 | 0.768 | 123 | 101 | 523 | nucleobase-containing compound catabolic process |
| GO:0000070 | 0.011 | 0.217 | 8 | 2 | 32 | mitotic sister chromatid segregation |
| GO:0043123 | 0.011 | 0.508 | 23 | 13 | 96 | positive regulation of I-kappaB kinase/NF-kappaB cascade |
| GO:0032642 | 0.012 | 0.279 | 9 | 3 | 38 | regulation of chemokine production |
| GO:0051321 | 0.013 | 0.514 | 22 | 13 | 95 | meiotic cell cycle |
| GO:0006261 | 0.013 | 0.360 | 12 | 5 | 50 | DNA-dependent DNA replication |
| GO:0009792 | 0.013 | 0.745 | 94 | 75 | 399 | embryo development ending in birth or egg hatching |
| GO:0071103 | 0.013 | 0.222 | 7 | 2 | 31 | DNA conformation change |
| GO:0043543 | 0.013 | 0.528 | 23 | 14 | 100 | protein acylation |
| GO:0032755 | 0.014 | 0.141 | 6 | 1 | 24 | positive regulation of interleukin-6 production |
| GO:0048013 | 0.014 | 0.141 | 6 | 1 | 24 | ephrin receptor signaling pathway |
| GO:0002504 | 0.014 | 0.440 | 16 | 8 | 67 | antigen processing and presentation of peptide or polysaccharide antigen via MHC class II |
| GO:0050829 | 0.014 | <0.0001 | 4 | 0 | 16 | defense response to Gram-negative bacterium |
| GO:0070979 | 0.014 | <0.0001 | 4 | 0 | 16 | protein K11-linked ubiquitination |
| GO:0045893 | 0.014 | 0.808 | 170 | 146 | 725 | positive regulation of transcription, DNA-dependent |
| GO:0071345 | 0.015 | 0.713 | 69 | 53 | 292 | cellular response to cytokine stimulus |
| GO:0048732 | 0.015 | 0.680 | 54 | 40 | 230 | gland development |
| GO:0001701 | 0.015 | 0.691 | 57 | 43 | 244 | in utero embryonic development |
| GO:0051783 | 0.016 | 0.448 | 15 | 8 | 66 | regulation of nuclear division |
| GO:0045892 | 0.016 | 0.663 | 47 | 34 | 197 | negative regulation of transcription, DNA-dependent |
| GO:0007052 | 0.017 | 0.232 | 7 | 2 | 30 | mitotic spindle organization |
| GO:0060324 | 0.017 | 0.232 | 7 | 2 | 30 | face development |
| GO:0016458 | 0.017 | 0.147 | 5 | 1 | 23 | gene silencing |
| GO:0032760 | 0.017 | 0.148 | 5 | 1 | 23 | positive regulation of tumor necrosis factor production |
| GO:0000077 | 0.017 | 0.293 | 9 | 3 | 36 | DNA damage checkpoint |
| GO:0042254 | 0.018 | <0.0001 | 4 | 0 | 15 | ribosome biogenesis |
| GO:0042633 | 0.018 | 0.406 | 13 | 6 | 54 | hair cycle |
| GO:0034508 | 0.018 | <0.0001 | 4 | 0 | 15 | centromere complex assembly |
| GO:0043928 | 0.018 | <0.0001 | 4 | 0 | 15 | exonucleolytic nuclear-transcribed mRNA catabolic process involved in deadenylation-dependent decay |
| GO:2001021 | 0.018 | <0.0001 | 4 | 0 | 15 | negative regulation of response to DNA damage stimulus |
| GO:0070302 | 0.018 | 0.564 | 25 | 16 | 108 | regulation of stress-activated protein kinase signaling cascade |
| GO:0006888 | 0.018 | 0.296 | 8 | 3 | 36 | ER to Golgi vesicle-mediated transport |
| GO:0007059 | 0.019 | 0.436 | 14 | 7 | 59 | chromosome segregation |
| GO:0006606 | 0.020 | 0.647 | 39 | 28 | 168 | protein import into nucleus |
| GO:0061180 | 0.020 | 0.437 | 14 | 7 | 59 | mammary gland epithelium development |
| GO:0006661 | 0.020 | 0.479 | 16 | 9 | 70 | phosphatidylinositol biosynthetic process |
| GO:0032259 | 0.020 | 0.479 | 16 | 9 | 70 | methylation |
| GO:0006475 | 0.021 | 0.526 | 20 | 12 | 86 | internal protein amino acid acetylation |
| GO:0001942 | 0.021 | 0.415 | 12 | 6 | 53 | hair follicle development |
| GO:0022404 | 0.021 | 0.415 | 12 | 6 | 53 | molting cycle process |
| GO:0007126 | 0.021 | 0.541 | 21 | 13 | 91 | meiosis |
| GO:0007584 | 0.021 | 0.598 | 29 | 19 | 122 | response to nutrient |
| GO:0043552 | 0.022 | 0.155 | 5 | 1 | 22 | positive regulation of phosphatidylinositol 3-kinase activity |
| GO:0051607 | 0.023 | 0.531 | 20 | 12 | 85 | defense response to virus |
| GO:0008544 | 0.023 | 0.608 | 30 | 20 | 126 | epidermis development |
| GO:0043044 | 0.024 | <0.0001 | 3 | 0 | 14 | ATP-dependent chromatin remodeling |
| GO:0044766 | 0.024 | <0.0001 | 3 | 0 | 14 | multi-organism transport |
| GO:0046685 | 0.024 | <0.0001 | 3 | 0 | 14 | response to arsenic-containing substance |
| GO:0055003 | 0.024 | <0.0001 | 3 | 0 | 14 | cardiac myofibril assembly |
| GO:0042176 | 0.024 | 0.604 | 28 | 19 | 121 | regulation of protein catabolic process |
| GO:0044344 | 0.024 | 0.612 | 30 | 20 | 126 | cellular response to fibroblast growth factor stimulus |
| GO:0006399 | 0.025 | 0.424 | 12 | 6 | 52 | tRNA metabolic process |
| GO:0007098 | 0.025 | 0.250 | 7 | 2 | 28 | centrosome cycle |
| GO:0016575 | 0.025 | 0.250 | 7 | 2 | 28 | histone deacetylation |
| GO:0031058 | 0.025 | 0.250 | 7 | 2 | 28 | positive regulation of histone modification |
| GO:0043487 | 0.025 | 0.250 | 7 | 2 | 28 | regulation of RNA stability |
| GO:0050688 | 0.026 | 0.396 | 11 | 5 | 46 | regulation of defense response to virus |
| GO:0042180 | 0.026 | 0.662 | 39 | 28 | 165 | cellular ketone metabolic process |
| GO:0050678 | 0.026 | 0.658 | 38 | 27 | 160 | regulation of epithelial cell proliferation |
| GO:0022607 | 0.026 | 0.851 | 237 | 212 | 1009 | cellular component assembly |
| GO:0060135 | 0.026 | 0.361 | 9 | 4 | 40 | maternal process involved in female pregnancy |
| GO:0042773 | 0.026 | 0.315 | 8 | 3 | 34 | ATP synthesis coupled electron transport |
| GO:0071347 | 0.026 | 0.315 | 8 | 3 | 34 | cellular response to interleukin-1 |
| GO:0090263 | 0.026 | 0.315 | 8 | 3 | 34 | positive regulation of canonical Wnt receptor signaling pathway |
| GO:0022411 | 0.027 | 0.707 | 52 | 40 | 223 | cellular component disassembly |
| GO:0044106 | 0.027 | 0.631 | 32 | 22 | 135 | cellular amine metabolic process |
| GO:0002437 | 0.027 | 0.163 | 5 | 1 | 21 | inflammatory response to antigenic stimulus |
| GO:0007094 | 0.027 | 0.163 | 5 | 1 | 21 | mitotic cell cycle spindle assembly checkpoint |
| GO:0016574 | 0.027 | 0.163 | 5 | 1 | 21 | histone ubiquitination |
| GO:0043516 | 0.027 | 0.163 | 5 | 1 | 21 | regulation of DNA damage response, signal transduction by p53 class mediator |
| GO:0080135 | 0.030 | 0.571 | 22 | 14 | 93 | regulation of cellular response to stress |
| GO:0030518 | 0.030 | 0.549 | 19 | 12 | 83 | intracellular steroid hormone receptor signaling pathway |
| GO:0070646 | 0.030 | 0.406 | 11 | 5 | 45 | protein modification by small protein removal |
| GO:0031056 | 0.030 | 0.259 | 6 | 2 | 27 | regulation of histone modification |
| GO:0006901 | 0.031 | 0.260 | 6 | 2 | 27 | vesicle coating |
| GO:0007131 | 0.031 | 0.260 | 6 | 2 | 27 | reciprocal meiotic recombination |
| GO:0034446 | 0.031 | 0.260 | 6 | 2 | 27 | substrate adhesion-dependent cell spreading |
| GO:0045839 | 0.031 | 0.260 | 6 | 2 | 27 | negative regulation of mitosis |
| GO:0051225 | 0.031 | 0.260 | 6 | 2 | 27 | spindle assembly |
| GO:0006586 | 0.031 | <0.0001 | 3 | 0 | 13 | indolalkylamine metabolic process |
| GO:0042255 | 0.031 | <0.0001 | 3 | 0 | 13 | ribosome assembly |
| GO:0042401 | 0.031 | <0.0001 | 3 | 0 | 13 | cellular biogenic amine biosynthetic process |
| GO:0045684 | 0.031 | <0.0001 | 3 | 0 | 13 | positive regulation of epidermis development |
| GO:0046641 | 0.031 | <0.0001 | 3 | 0 | 13 | positive regulation of alpha-beta T cell proliferation |
| GO:0071276 | 0.031 | <0.0001 | 3 | 0 | 13 | cellular response to cadmium ion |
| GO:0001836 | 0.032 | 0.372 | 9 | 4 | 39 | release of cytochrome c from mitochondria |
| GO:0006312 | 0.032 | 0.325 | 8 | 3 | 33 | mitotic recombination |
| GO:0002009 | 0.032 | 0.743 | 64 | 51 | 273 | morphogenesis of an epithelium |
| GO:0006338 | 0.033 | 0.442 | 12 | 6 | 50 | chromatin remodeling |
| GO:0044267 | 0.033 | 0.668 | 37 | 27 | 148 | cellular protein metabolic process |
| GO:0030947 | 0.034 | 0.171 | 5 | 1 | 20 | regulation of vascular endothelial growth factor receptor signaling pathway |
| GO:0032732 | 0.034 | 0.171 | 5 | 1 | 20 | positive regulation of interleukin-1 production |
| GO:0048255 | 0.034 | 0.171 | 5 | 1 | 20 | mRNA stabilization |
| GO:0051146 | 0.034 | 0.681 | 39 | 29 | 167 | striated muscle cell differentiation |
| GO:0044706 | 0.034 | 0.677 | 38 | 28 | 162 | multi-multicellular organism process |
| GO:0009059 | 0.035 | 0.442 | 12 | 6 | 45 | macromolecule biosynthetic process |
| GO:0006120 | 0.038 | 0.271 | 6 | 2 | 26 | mitochondrial electron transport, NADH to ubiquinone |
| GO:0006418 | 0.038 | 0.271 | 6 | 2 | 26 | tRNA aminoacylation for protein translation |
| GO:0010833 | 0.038 | 0.337 | 8 | 3 | 32 | telomere maintenance via telomere lengthening |
| GO:0007067 | 0.039 | 0.653 | 31 | 22 | 130 | mitosis |
| GO:0046328 | 0.040 | 0.590 | 21 | 14 | 91 | regulation of JNK cascade |
| GO:0006595 | 0.040 | <0.0001 | 3 | 0 | 12 | polyamine metabolic process |
| GO:0009310 | 0.040 | <0.0001 | 3 | 0 | 12 | amine catabolic process |
| GO:0010830 | 0.040 | <0.0001 | 3 | 0 | 12 | regulation of myotube differentiation |
| GO:0034502 | 0.040 | <0.0001 | 3 | 0 | 12 | protein localization to chromosome |
| GO:0043101 | 0.040 | <0.0001 | 3 | 0 | 12 | purine-containing compound salvage |
| GO:0045063 | 0.040 | <0.0001 | 3 | 0 | 12 | T-helper 1 cell differentiation |
| GO:0046112 | 0.040 | <0.0001 | 3 | 0 | 12 | nucleobase biosynthetic process |
| GO:0051569 | 0.040 | <0.0001 | 3 | 0 | 12 | regulation of histone H3-K4 methylation |
| GO:0051965 | 0.040 | <0.0001 | 3 | 0 | 12 | positive regulation of synapse assembly |
| GO:0071359 | 0.040 | <0.0001 | 3 | 0 | 12 | cellular response to dsRNA |
| GO:0072160 | 0.040 | <0.0001 | 3 | 0 | 12 | nephron tubule epithelial cell differentiation |
| GO:0001890 | 0.040 | 0.611 | 24 | 16 | 101 | placenta development |
| GO:0055001 | 0.041 | 0.636 | 27 | 19 | 116 | muscle cell development |
| GO:0002768 | 0.041 | 0.620 | 25 | 17 | 106 | immune response-regulating cell surface receptor signaling pathway |
| GO:0032507 | 0.042 | 0.542 | 16 | 10 | 70 | maintenance of protein location in cell |
| GO:0006406 | 0.042 | 0.428 | 10 | 5 | 43 | mRNA export from nucleus |
| GO:0043933 | 0.042 | 0.845 | 169 | 150 | 711 | macromolecular complex subunit organization |
| GO:0000718 | 0.042 | 0.181 | 4 | 1 | 19 | nucleotide-excision repair, DNA damage removal |
| GO:0007620 | 0.042 | 0.181 | 4 | 1 | 19 | copulation |
| GO:0033280 | 0.042 | 0.181 | 4 | 1 | 19 | response to vitamin D |
| GO:0048199 | 0.042 | 0.181 | 4 | 1 | 19 | vesicle targeting, to, from or within Golgi |
| GO:0006412 | 0.043 | 0.429 | 10 | 5 | 42 | translation |
| GO:0030216 | 0.044 | 0.510 | 14 | 8 | 59 | keratinocyte differentiation |
| GO:0046822 | 0.044 | 0.655 | 29 | 21 | 125 | regulation of nucleocytoplasmic transport |
| GO:0097193 | 0.045 | 0.598 | 21 | 14 | 90 | intrinsic apoptotic signaling pathway |
| GO:0045787 | 0.045 | 0.609 | 22 | 15 | 95 | positive regulation of cell cycle |
| GO:0019827 | 0.046 | 0.532 | 15 | 9 | 64 | stem cell maintenance |
| GO:0007257 | 0.046 | 0.283 | 6 | 2 | 25 | activation of JUN kinase activity |
| GO:0010043 | 0.046 | 0.283 | 6 | 2 | 25 | response to zinc ion |
| GO:0016925 | 0.046 | 0.283 | 6 | 2 | 25 | protein sumoylation |
| GO:0043388 | 0.046 | 0.283 | 6 | 2 | 25 | positive regulation of DNA binding |
| GO:0009161 | 0.046 | 0.349 | 7 | 3 | 31 | ribonucleoside monophosphate metabolic process |
| GO:0010332 | 0.046 | 0.349 | 7 | 3 | 31 | response to gamma radiation |
| GO:0042770 | 0.047 | 0.435 | 10 | 5 | 42 | signal transduction in response to DNA damage |
| GO:0000910 | 0.047 | 0.551 | 16 | 10 | 69 | cytokinesis |
| GO:0010468 | 0.048 | 0.778 | 73 | 61 | 295 | regulation of gene expression |
| GO:0051603 | 0.048 | 0.631 | 24 | 17 | 103 | proteolysis involved in cellular protein catabolic process |
| GO:0051318 | 0.049 | 0.440 | 10 | 5 | 42 | G1 phase |
| GO:0032268 | 0.049 | 0.857 | 186 | 167 | 787 | regulation of cellular protein metabolic process |
