# Supplementary material for: Hidden Treasures in “Ancient” Microarrays: Gene-Expression Portrays Biology and Potential Resistance Pathways of Major Lung Cancer Subtypes and Normal Tissue
Source: Front Oncol. 2014 Sep 29;4:251. doi: 10.3389/fonc.2014.00251 (PMC4178426; doi:10.3389/fonc.2014.00251)
Supplement: Figure S1 — Clinically relevant differentially expressed pathways in each tissue type versus the rest. The pathway maps are arranged according to the scheme in the left-top corner. Red indicates overexpression in genes, dark green downregulation. In the Cell Cycle pathway, tumor suppressor genes were overexpressed in normal lung tissue while oncogenes and tumor driving cyclins were overexpressed in cancers. The gene thymidylate synthase (TYMS or TS, belonging to the pathway “one carbon pool by folate”) is relevant for tumor growth and is also a treatment target. Notably, TYMS was overexpressed only in the tumors that are generally refractory to the drug pemetrexed, as the squamous and the small-cell lung cancer. TYMS was not overexpressed in mesothelioma, but it is known that TYMS expression is highly variable in this cancer. In the ERBB pathway, the ERBB2/HER2 and ERBB3/HER3 were overexpressed in adenocarcinoma, while the ERBB4 was overexpressed in the carcinoids. [file Data_Sheet_1.ZIP › File S3.ZIP!/GO Analysis/Squamous vs Rest Enrichment Analysis/Underrepresented GO terms of Up-regulated Genes.html]

Gene to GO BP Conditional test for under-representation

| GOBPID | Pvalue | OddsRatio | ExpCount | Count | Size | Term |
| --- | --- | --- | --- | --- | --- | --- |
| GO:0007268 | <0.0001 | 0.385 | 84 | 38 | 434 | synaptic transmission |
| GO:0035637 | <0.0001 | 0.499 | 110 | 63 | 571 | multicellular organismal signaling |
| GO:0023052 | <0.0001 | 0.741 | 606 | 520 | 3144 | signaling |
| GO:0032501 | <0.0001 | 0.754 | 706 | 621 | 3664 | multicellular organismal process |
| GO:0050877 | <0.0001 | 0.521 | 96 | 57 | 486 | neurological system process |
| GO:0007601 | <0.0001 | 0.260 | 26 | 8 | 135 | visual perception |
| GO:0006955 | <0.0001 | 0.351 | 37 | 15 | 186 | immune response |
| GO:0006874 | <0.0001 | 0.386 | 38 | 17 | 198 | cellular calcium ion homeostasis |
| GO:0006813 | <0.0001 | 0.157 | 16 | 3 | 82 | potassium ion transport |
| GO:0044057 | <0.0001 | 0.467 | 51 | 27 | 263 | regulation of system process |
| GO:0007186 | <0.0001 | 0.451 | 47 | 24 | 239 | G-protein coupled receptor signaling pathway |
| GO:0007600 | <0.0001 | 0.438 | 44 | 22 | 227 | sensory perception |
| GO:0072507 | <0.0001 | 0.439 | 42 | 21 | 218 | divalent inorganic cation homeostasis |
| GO:0008277 | <0.0001 | 0.168 | 15 | 3 | 77 | regulation of G-protein coupled receptor protein signaling pathway |
| GO:0006812 | <0.0001 | 0.587 | 83 | 54 | 429 | cation transport |
| GO:0030802 | <0.0001 | 0.207 | 16 | 4 | 84 | regulation of cyclic nucleotide biosynthetic process |
| GO:1900371 | <0.0001 | 0.207 | 16 | 4 | 84 | regulation of purine nucleotide biosynthetic process |
| GO:0045937 | <0.0001 | 0.625 | 99 | 68 | 512 | positive regulation of phosphate metabolic process |
| GO:0052652 | <0.0001 | 0.221 | 15 | 4 | 79 | cyclic purine nucleotide metabolic process |
| GO:0007204 | <0.0001 | 0.370 | 26 | 11 | 134 | elevation of cytosolic calcium ion concentration |
| GO:0030814 | <0.0001 | 0.224 | 15 | 4 | 78 | regulation of cAMP metabolic process |
| GO:0015844 | <0.0001 | 0.089 | 9 | 1 | 48 | monoamine transport |
| GO:0003073 | <0.0001 | 0.148 | 11 | 2 | 58 | regulation of systemic arterial blood pressure |
| GO:0051174 | <0.0001 | 0.726 | 174 | 137 | 901 | regulation of phosphorus metabolic process |
| GO:0072376 | 0.001 | 0.157 | 11 | 2 | 55 | protein activation cascade |
| GO:1901342 | 0.001 | 0.407 | 26 | 12 | 134 | regulation of vasculature development |
| GO:0007165 | 0.001 | 0.825 | 539 | 486 | 2786 | signal transduction |
| GO:0003001 | 0.001 | 0.558 | 50 | 31 | 260 | generation of a signal involved in cell-cell signaling |
| GO:0032940 | 0.001 | 0.650 | 84 | 60 | 435 | secretion by cell |
| GO:0006833 | 0.001 | <0.0001 | 6 | 0 | 32 | water transport |
| GO:0007269 | 0.001 | 0.169 | 10 | 2 | 51 | neurotransmitter secretion |
| GO:0045761 | 0.002 | 0.170 | 10 | 2 | 51 | regulation of adenylate cyclase activity |
| GO:0070838 | 0.002 | 0.507 | 35 | 20 | 183 | divalent metal ion transport |
| GO:0042220 | 0.002 | <0.0001 | 6 | 0 | 30 | response to cocaine |
| GO:0002703 | 0.002 | 0.293 | 15 | 5 | 76 | regulation of leukocyte mediated immunity |
| GO:0033674 | 0.002 | 0.627 | 64 | 44 | 333 | positive regulation of kinase activity |
| GO:0038032 | 0.002 | <0.0001 | 6 | 0 | 29 | termination of G-protein coupled receptor signaling pathway |
| GO:0006140 | 0.002 | 0.582 | 48 | 31 | 251 | regulation of nucleotide metabolic process |
| GO:0030003 | 0.002 | 0.599 | 53 | 35 | 276 | cellular cation homeostasis |
| GO:0030810 | 0.002 | 0.227 | 11 | 3 | 58 | positive regulation of nucleotide biosynthetic process |
| GO:0043270 | 0.002 | 0.377 | 19 | 8 | 96 | positive regulation of ion transport |
| GO:0007602 | 0.002 | <0.0001 | 5 | 0 | 28 | phototransduction |
| GO:0018212 | 0.003 | 0.507 | 32 | 18 | 165 | peptidyl-tyrosine modification |
| GO:0006814 | 0.003 | 0.306 | 14 | 5 | 73 | sodium ion transport |
| GO:0006968 | 0.003 | 0.181 | 9 | 2 | 48 | cellular defense response |
| GO:0007267 | 0.003 | 0.563 | 42 | 26 | 208 | cell-cell signaling |
| GO:0051969 | 0.003 | 0.511 | 32 | 18 | 164 | regulation of transmission of nerve impulse |
| GO:1900274 | 0.003 | 0.236 | 11 | 3 | 56 | regulation of phospholipase C activity |
| GO:0050731 | 0.003 | 0.406 | 19 | 9 | 101 | positive regulation of peptidyl-tyrosine phosphorylation |
| GO:0007187 | 0.003 | 0.424 | 21 | 10 | 108 | G-protein coupled receptor signaling pathway, coupled to cyclic nucleotide second messenger |
| GO:0055082 | 0.003 | 0.705 | 96 | 73 | 498 | cellular chemical homeostasis |
| GO:0007610 | 0.004 | 0.595 | 46 | 30 | 236 | behavior |
| GO:0045921 | 0.004 | <0.0001 | 5 | 0 | 26 | positive regulation of exocytosis |
| GO:0055065 | 0.004 | 0.607 | 48 | 32 | 249 | metal ion homeostasis |
| GO:0002819 | 0.004 | 0.320 | 13 | 5 | 70 | regulation of adaptive immune response |
| GO:0030819 | 0.004 | 0.245 | 10 | 3 | 54 | positive regulation of cAMP biosynthetic process |
| GO:0050778 | 0.004 | 0.620 | 52 | 35 | 268 | positive regulation of immune response |
| GO:0010817 | 0.004 | 0.625 | 53 | 36 | 274 | regulation of hormone levels |
| GO:0051179 | 0.004 | 0.850 | 523 | 479 | 2714 | localization |
| GO:0030049 | 0.004 | 0.119 | 7 | 1 | 36 | muscle filament sliding |
| GO:0043500 | 0.004 | 0.119 | 7 | 1 | 36 | muscle adaptation |
| GO:0007586 | 0.004 | 0.351 | 15 | 6 | 77 | digestion |
| GO:0051046 | 0.004 | 0.475 | 24 | 13 | 125 | regulation of secretion |
| GO:0006644 | 0.004 | 0.581 | 41 | 26 | 211 | phospholipid metabolic process |
| GO:0006897 | 0.004 | 0.615 | 49 | 33 | 254 | endocytosis |
| GO:1900544 | 0.005 | 0.292 | 12 | 4 | 61 | positive regulation of purine nucleotide metabolic process |
| GO:0051924 | 0.005 | 0.437 | 20 | 10 | 105 | regulation of calcium ion transport |
| GO:0050727 | 0.005 | 0.522 | 29 | 17 | 152 | regulation of inflammatory response |
| GO:0032103 | 0.005 | 0.492 | 25 | 14 | 132 | positive regulation of response to external stimulus |
| GO:0031281 | 0.005 | 0.123 | 7 | 1 | 35 | positive regulation of cyclase activity |
| GO:0002791 | 0.005 | 0.482 | 24 | 13 | 125 | regulation of peptide secretion |
| GO:0003015 | 0.005 | 0.470 | 23 | 12 | 118 | heart process |
| GO:0030801 | 0.006 | 0.297 | 12 | 4 | 60 | positive regulation of cyclic nucleotide metabolic process |
| GO:0007611 | 0.006 | 0.497 | 25 | 14 | 131 | learning or memory |
| GO:0019724 | 0.006 | 0.362 | 14 | 6 | 75 | B cell mediated immunity |
| GO:0009593 | 0.006 | <0.0001 | 5 | 0 | 24 | detection of chemical stimulus |
| GO:0051602 | 0.006 | <0.0001 | 5 | 0 | 24 | response to electrical stimulus |
| GO:0055078 | 0.006 | <0.0001 | 5 | 0 | 24 | sodium ion homeostasis |
| GO:0048514 | 0.006 | 0.673 | 66 | 48 | 342 | blood vessel morphogenesis |
| GO:0050714 | 0.006 | 0.336 | 13 | 5 | 67 | positive regulation of protein secretion |
| GO:0006954 | 0.006 | 0.609 | 44 | 29 | 224 | inflammatory response |
| GO:0006959 | 0.006 | 0.302 | 11 | 4 | 59 | humoral immune response |
| GO:0010627 | 0.007 | 0.732 | 99 | 78 | 516 | regulation of intracellular protein kinase cascade |
| GO:0043687 | 0.007 | 0.466 | 21 | 11 | 109 | post-translational protein modification |
| GO:0042325 | 0.007 | 0.761 | 126 | 102 | 652 | regulation of phosphorylation |
| GO:0046395 | 0.007 | 0.397 | 15 | 7 | 80 | carboxylic acid catabolic process |
| GO:0042742 | 0.008 | 0.373 | 14 | 6 | 73 | defense response to bacterium |
| GO:0050796 | 0.008 | 0.489 | 22 | 12 | 114 | regulation of insulin secretion |
| GO:0015837 | 0.009 | 0.213 | 8 | 2 | 41 | amine transport |
| GO:0044242 | 0.009 | 0.476 | 21 | 11 | 107 | cellular lipid catabolic process |
| GO:0055088 | 0.009 | 0.314 | 11 | 4 | 57 | lipid homeostasis |
| GO:0001912 | 0.009 | <0.0001 | 4 | 0 | 22 | positive regulation of leukocyte mediated cytotoxicity |
| GO:0060627 | 0.009 | 0.563 | 31 | 19 | 159 | regulation of vesicle-mediated transport |
| GO:0007202 | 0.009 | 0.272 | 9 | 3 | 49 | activation of phospholipase C activity |
| GO:0031341 | 0.009 | 0.135 | 6 | 1 | 32 | regulation of cell killing |
| GO:0051338 | 0.009 | 0.739 | 95 | 75 | 493 | regulation of transferase activity |
| GO:0044282 | 0.009 | 0.599 | 37 | 24 | 190 | small molecule catabolic process |
| GO:0030258 | 0.010 | 0.481 | 20 | 11 | 106 | lipid modification |
| GO:0010647 | 0.010 | 0.768 | 122 | 100 | 635 | positive regulation of cell communication |
| GO:0023056 | 0.010 | 0.768 | 122 | 100 | 635 | positive regulation of signaling |
| GO:0045582 | 0.011 | 0.278 | 9 | 3 | 48 | positive regulation of T cell differentiation |
| GO:0008202 | 0.011 | 0.627 | 41 | 28 | 213 | steroid metabolic process |
| GO:0045123 | 0.011 | <0.0001 | 4 | 0 | 21 | cellular extravasation |
| GO:0060326 | 0.011 | 0.537 | 25 | 15 | 131 | cell chemotaxis |
| GO:0016042 | 0.012 | 0.281 | 9 | 3 | 47 | lipid catabolic process |
| GO:0002699 | 0.012 | 0.364 | 12 | 5 | 62 | positive regulation of immune effector process |
| GO:0006687 | 0.012 | 0.225 | 8 | 2 | 39 | glycosphingolipid metabolic process |
| GO:0032675 | 0.012 | 0.284 | 9 | 3 | 47 | regulation of interleukin-6 production |
| GO:0070252 | 0.012 | 0.284 | 9 | 3 | 47 | actin-mediated cell contraction |
| GO:0042330 | 0.013 | 0.746 | 92 | 73 | 476 | taxis |
| GO:0008360 | 0.013 | 0.422 | 15 | 7 | 76 | regulation of cell shape |
| GO:0051279 | 0.013 | 0.144 | 6 | 1 | 30 | regulation of release of sequestered calcium ion into cytosol |
| GO:0060079 | 0.013 | 0.144 | 6 | 1 | 30 | regulation of excitatory postsynaptic membrane potential |
| GO:0051056 | 0.013 | 0.653 | 45 | 32 | 235 | regulation of small GTPase mediated signal transduction |
| GO:0007588 | 0.014 | 0.333 | 10 | 4 | 54 | excretion |
| GO:0071805 | 0.014 | <0.0001 | 4 | 0 | 20 | potassium ion transmembrane transport |
| GO:0002274 | 0.014 | 0.484 | 19 | 10 | 96 | myeloid leukocyte activation |
| GO:0003012 | 0.014 | 0.627 | 38 | 26 | 197 | muscle system process |
| GO:0048731 | 0.014 | 0.869 | 454 | 419 | 2358 | system development |
| GO:1901615 | 0.014 | 0.681 | 53 | 39 | 276 | organic hydroxy compound metabolic process |
| GO:0060191 | 0.014 | 0.468 | 17 | 9 | 89 | regulation of lipase activity |
| GO:0002761 | 0.014 | 0.372 | 12 | 5 | 61 | regulation of myeloid leukocyte differentiation |
| GO:0008306 | 0.014 | 0.291 | 9 | 3 | 46 | associative learning |
| GO:0046427 | 0.014 | 0.291 | 9 | 3 | 46 | positive regulation of JAK-STAT cascade |
| GO:0051050 | 0.015 | 0.684 | 53 | 39 | 273 | positive regulation of transport |
| GO:0045776 | 0.016 | 0.149 | 6 | 1 | 29 | negative regulation of blood pressure |
| GO:0032147 | 0.016 | 0.604 | 32 | 21 | 165 | activation of protein kinase activity |
| GO:0045619 | 0.017 | 0.409 | 13 | 6 | 67 | regulation of lymphocyte differentiation |
| GO:0016525 | 0.017 | 0.298 | 9 | 3 | 45 | negative regulation of angiogenesis |
| GO:0050806 | 0.017 | 0.298 | 9 | 3 | 45 | positive regulation of synaptic transmission |
| GO:0043413 | 0.017 | 0.584 | 28 | 18 | 146 | macromolecule glycosylation |
| GO:0006810 | 0.017 | 0.860 | 348 | 317 | 1777 | transport |
| GO:0002675 | 0.017 | <0.0001 | 4 | 0 | 19 | positive regulation of acute inflammatory response |
| GO:0006699 | 0.017 | <0.0001 | 4 | 0 | 19 | bile acid biosynthetic process |
| GO:0007040 | 0.017 | <0.0001 | 4 | 0 | 19 | lysosome organization |
| GO:0009651 | 0.017 | <0.0001 | 4 | 0 | 19 | response to salt stress |
| GO:0015872 | 0.017 | <0.0001 | 4 | 0 | 19 | dopamine transport |
| GO:0031952 | 0.017 | <0.0001 | 4 | 0 | 19 | regulation of protein autophosphorylation |
| GO:0034367 | 0.017 | <0.0001 | 4 | 0 | 19 | macromolecular complex remodeling |
| GO:0034369 | 0.017 | <0.0001 | 4 | 0 | 19 | plasma lipoprotein particle remodeling |
| GO:0048260 | 0.017 | <0.0001 | 4 | 0 | 19 | positive regulation of receptor-mediated endocytosis |
| GO:0014902 | 0.017 | 0.238 | 7 | 2 | 37 | myotube differentiation |
| GO:0001934 | 0.018 | 0.721 | 66 | 51 | 341 | positive regulation of protein phosphorylation |
| GO:0002250 | 0.018 | 0.459 | 16 | 8 | 80 | adaptive immune response |
| GO:0009410 | 0.019 | 0.514 | 19 | 11 | 100 | response to xenobiotic stimulus |
| GO:0050795 | 0.019 | 0.541 | 22 | 13 | 113 | regulation of behavior |
| GO:0001990 | 0.019 | 0.155 | 5 | 1 | 28 | regulation of systemic arterial blood pressure by hormone |
| GO:0007190 | 0.019 | 0.155 | 5 | 1 | 28 | activation of adenylate cyclase activity |
| GO:0034754 | 0.019 | 0.417 | 13 | 6 | 66 | cellular hormone metabolic process |
| GO:0072329 | 0.019 | 0.417 | 13 | 6 | 66 | monocarboxylic acid catabolic process |
| GO:0019233 | 0.019 | 0.304 | 8 | 3 | 44 | sensory perception of pain |
| GO:0045859 | 0.019 | 0.759 | 88 | 71 | 457 | regulation of protein kinase activity |
| GO:0006809 | 0.020 | 0.305 | 8 | 3 | 44 | nitric oxide biosynthetic process |
| GO:0050776 | 0.020 | 0.542 | 22 | 13 | 111 | regulation of immune response |
| GO:0043269 | 0.020 | 0.555 | 23 | 14 | 118 | regulation of ion transport |
| GO:0048583 | 0.020 | 0.839 | 219 | 194 | 1109 | regulation of response to stimulus |
| GO:0002708 | 0.020 | 0.245 | 7 | 2 | 36 | positive regulation of lymphocyte mediated immunity |
| GO:0046470 | 0.020 | 0.245 | 7 | 2 | 36 | phosphatidylcholine metabolic process |
| GO:0051349 | 0.020 | 0.245 | 7 | 2 | 36 | positive regulation of lyase activity |
| GO:0046883 | 0.020 | 0.594 | 28 | 18 | 144 | regulation of hormone secretion |
| GO:0051283 | 0.021 | 0.355 | 10 | 4 | 51 | negative regulation of sequestering of calcium ion |
| GO:0001885 | 0.021 | <0.0001 | 3 | 0 | 18 | endothelial cell development |
| GO:0007626 | 0.021 | 0.391 | 11 | 5 | 58 | locomotory behavior |
| GO:0021700 | 0.022 | 0.589 | 26 | 17 | 137 | developmental maturation |
| GO:0002687 | 0.022 | 0.393 | 11 | 5 | 58 | positive regulation of leukocyte migration |
| GO:0015850 | 0.022 | 0.311 | 8 | 3 | 43 | organic hydroxy compound transport |
| GO:0021549 | 0.023 | 0.160 | 5 | 1 | 27 | cerebellum development |
| GO:0006958 | 0.023 | 0.161 | 5 | 1 | 27 | complement activation, classical pathway |
| GO:0060396 | 0.023 | 0.161 | 5 | 1 | 27 | growth hormone receptor signaling pathway |
| GO:0014068 | 0.023 | 0.313 | 8 | 3 | 43 | positive regulation of phosphatidylinositol 3-kinase cascade |
| GO:0042509 | 0.023 | 0.313 | 8 | 3 | 43 | regulation of tyrosine phosphorylation of STAT protein |
| GO:0006805 | 0.023 | 0.526 | 19 | 11 | 98 | xenobiotic metabolic process |
| GO:0031623 | 0.024 | 0.252 | 7 | 2 | 35 | receptor internalization |
| GO:0032496 | 0.024 | 0.630 | 32 | 22 | 167 | response to lipopolysaccharide |
| GO:0006953 | 0.024 | 0.253 | 7 | 2 | 35 | acute-phase response |
| GO:0006631 | 0.024 | 0.649 | 36 | 25 | 185 | fatty acid metabolic process |
| GO:0046849 | 0.024 | 0.363 | 10 | 4 | 50 | bone remodeling |
| GO:0002366 | 0.024 | 0.543 | 20 | 12 | 104 | leukocyte activation involved in immune response |
| GO:0055080 | 0.025 | <0.0001 | 3 | 0 | 17 | cation homeostasis |
| GO:0015833 | 0.025 | 0.612 | 29 | 19 | 148 | peptide transport |
| GO:0010518 | 0.025 | 0.431 | 12 | 6 | 64 | positive regulation of phospholipase activity |
| GO:1901606 | 0.025 | 0.431 | 12 | 6 | 64 | alpha-amino acid catabolic process |
| GO:0009187 | 0.025 | <0.0001 | 3 | 0 | 17 | cyclic nucleotide metabolic process |
| GO:0071345 | 0.025 | 0.732 | 63 | 49 | 326 | cellular response to cytokine stimulus |
| GO:0008015 | 0.026 | 0.647 | 34 | 24 | 176 | blood circulation |
| GO:0001963 | 0.026 | <0.0001 | 3 | 0 | 17 | synaptic transmission, dopaminergic |
| GO:0002825 | 0.026 | <0.0001 | 3 | 0 | 17 | regulation of T-helper 1 type immune response |
| GO:0031640 | 0.026 | <0.0001 | 3 | 0 | 17 | killing of cells of other organism |
| GO:0035813 | 0.026 | <0.0001 | 3 | 0 | 17 | regulation of renal sodium excretion |
| GO:0048488 | 0.026 | <0.0001 | 3 | 0 | 17 | synaptic vesicle endocytosis |
| GO:0051354 | 0.026 | <0.0001 | 3 | 0 | 17 | negative regulation of oxidoreductase activity |
| GO:0051930 | 0.026 | <0.0001 | 3 | 0 | 17 | regulation of sensory perception of pain |
| GO:0051954 | 0.026 | <0.0001 | 3 | 0 | 17 | positive regulation of amine transport |
| GO:0070741 | 0.026 | <0.0001 | 3 | 0 | 17 | response to interleukin-6 |
| GO:0045087 | 0.026 | 0.736 | 64 | 50 | 330 | innate immune response |
| GO:0002576 | 0.027 | 0.483 | 15 | 8 | 77 | platelet degranulation |
| GO:0002637 | 0.027 | 0.167 | 5 | 1 | 26 | regulation of immunoglobulin production |
| GO:0071825 | 0.027 | 0.167 | 5 | 1 | 26 | protein-lipid complex subunit organization |
| GO:0042311 | 0.028 | 0.371 | 9 | 4 | 49 | vasodilation |
| GO:0043534 | 0.028 | 0.371 | 9 | 4 | 49 | blood vessel endothelial cell migration |
| GO:0009712 | 0.028 | 0.261 | 7 | 2 | 34 | catechol-containing compound metabolic process |
| GO:0031645 | 0.028 | 0.261 | 7 | 2 | 34 | negative regulation of neurological system process |
| GO:0002696 | 0.028 | 0.657 | 35 | 25 | 183 | positive regulation of leukocyte activation |
| GO:0009101 | 0.028 | 0.657 | 35 | 25 | 183 | glycoprotein biosynthetic process |
| GO:0046486 | 0.028 | 0.672 | 39 | 28 | 201 | glycerolipid metabolic process |
| GO:0048871 | 0.029 | 0.577 | 22 | 14 | 115 | multicellular organismal homeostasis |
| GO:0007259 | 0.030 | 0.328 | 8 | 3 | 41 | JAK-STAT cascade |
| GO:0045766 | 0.031 | 0.470 | 13 | 7 | 69 | positive regulation of angiogenesis |
| GO:0003008 | 0.032 | 0.608 | 26 | 17 | 124 | system process |
| GO:0050663 | 0.032 | 0.378 | 9 | 4 | 48 | cytokine secretion |
| GO:0030072 | 0.032 | 0.619 | 27 | 18 | 139 | peptide hormone secretion |
| GO:0023051 | 0.032 | 0.806 | 116 | 99 | 590 | regulation of signaling |
| GO:0007631 | 0.032 | 0.447 | 12 | 6 | 62 | feeding behavior |
| GO:0032355 | 0.032 | 0.447 | 12 | 6 | 62 | response to estradiol stimulus |
| GO:0003018 | 0.032 | 0.583 | 22 | 14 | 114 | vascular process in circulatory system |
| GO:0071706 | 0.032 | 0.417 | 11 | 5 | 55 | tumor necrosis factor superfamily cytokine production |
| GO:0030534 | 0.032 | 0.268 | 6 | 2 | 33 | adult behavior |
| GO:0001964 | 0.032 | <0.0001 | 3 | 0 | 16 | startle response |
| GO:0006182 | 0.032 | <0.0001 | 3 | 0 | 16 | cGMP biosynthetic process |
| GO:0006704 | 0.032 | <0.0001 | 3 | 0 | 16 | glucocorticoid biosynthetic process |
| GO:0017158 | 0.032 | <0.0001 | 3 | 0 | 16 | regulation of calcium ion-dependent exocytosis |
| GO:0042430 | 0.032 | <0.0001 | 3 | 0 | 16 | indole-containing compound metabolic process |
| GO:0048246 | 0.032 | <0.0001 | 3 | 0 | 16 | macrophage chemotaxis |
| GO:0050710 | 0.032 | <0.0001 | 3 | 0 | 16 | negative regulation of cytokine secretion |
| GO:0060487 | 0.032 | <0.0001 | 3 | 0 | 16 | lung epithelial cell differentiation |
| GO:0002548 | 0.033 | 0.174 | 5 | 1 | 25 | monocyte chemotaxis |
| GO:0016079 | 0.033 | 0.174 | 5 | 1 | 25 | synaptic vesicle exocytosis |
| GO:0021587 | 0.033 | 0.174 | 5 | 1 | 25 | cerebellum morphogenesis |
| GO:0050433 | 0.033 | 0.174 | 5 | 1 | 25 | regulation of catecholamine secretion |
| GO:0051893 | 0.033 | 0.174 | 5 | 1 | 25 | regulation of focal adhesion assembly |
| GO:0070509 | 0.033 | 0.174 | 5 | 1 | 25 | calcium ion import |
| GO:0045936 | 0.033 | 0.675 | 37 | 27 | 193 | negative regulation of phosphate metabolic process |
| GO:0007632 | 0.033 | 0.269 | 6 | 2 | 33 | visual behavior |
| GO:0033762 | 0.033 | 0.269 | 6 | 2 | 33 | response to glucagon stimulus |
| GO:0046677 | 0.033 | 0.269 | 6 | 2 | 33 | response to antibiotic |
| GO:0008016 | 0.033 | 0.561 | 19 | 12 | 101 | regulation of heart contraction |
| GO:0050865 | 0.033 | 0.728 | 54 | 42 | 281 | regulation of cell activation |
| GO:0006811 | 0.033 | 0.676 | 37 | 27 | 188 | ion transport |
| GO:0006952 | 0.034 | 0.498 | 14 | 8 | 72 | defense response |
| GO:0048511 | 0.035 | 0.624 | 27 | 18 | 138 | rhythmic process |
| GO:0009581 | 0.035 | 0.478 | 13 | 7 | 68 | detection of external stimulus |
| GO:0060401 | 0.035 | 0.478 | 13 | 7 | 68 | cytosolic calcium ion transport |
| GO:0008217 | 0.035 | 0.384 | 9 | 4 | 47 | regulation of blood pressure |
| GO:0051048 | 0.035 | 0.552 | 18 | 11 | 94 | negative regulation of secretion |
| GO:0048878 | 0.036 | 0.552 | 18 | 11 | 92 | chemical homeostasis |
| GO:0002286 | 0.036 | 0.338 | 8 | 3 | 40 | T cell activation involved in immune response |
| GO:0042632 | 0.036 | 0.338 | 8 | 3 | 40 | cholesterol homeostasis |
| GO:0009617 | 0.036 | 0.659 | 32 | 23 | 167 | response to bacterium |
| GO:0034340 | 0.036 | 0.455 | 12 | 6 | 61 | response to type I interferon |
| GO:0060337 | 0.036 | 0.455 | 12 | 6 | 61 | type I interferon-mediated signaling pathway |
| GO:0015918 | 0.037 | 0.388 | 9 | 4 | 47 | sterol transport |
| GO:0010646 | 0.037 | 0.813 | 117 | 100 | 592 | regulation of cell communication |
| GO:0009152 | 0.037 | 0.643 | 29 | 20 | 149 | purine ribonucleotide biosynthetic process |
| GO:0031032 | 0.039 | 0.278 | 6 | 2 | 32 | actomyosin structure organization |
| GO:0046058 | 0.039 | <0.0001 | 3 | 0 | 15 | cAMP metabolic process |
| GO:0055085 | 0.039 | 0.787 | 84 | 70 | 438 | transmembrane transport |
| GO:0002920 | 0.039 | 0.182 | 5 | 1 | 24 | regulation of humoral immune response |
| GO:0003300 | 0.039 | 0.182 | 5 | 1 | 24 | cardiac muscle hypertrophy |
| GO:0009311 | 0.039 | 0.182 | 5 | 1 | 24 | oligosaccharide metabolic process |
| GO:0010524 | 0.039 | 0.182 | 5 | 1 | 24 | positive regulation of calcium ion transport into cytosol |
| GO:0030800 | 0.039 | 0.182 | 5 | 1 | 24 | negative regulation of cyclic nucleotide metabolic process |
| GO:1900543 | 0.039 | 0.182 | 5 | 1 | 24 | negative regulation of purine nucleotide metabolic process |
| GO:0001504 | 0.040 | <0.0001 | 3 | 0 | 15 | neurotransmitter uptake |
| GO:0007263 | 0.040 | <0.0001 | 3 | 0 | 15 | nitric oxide mediated signal transduction |
| GO:0009074 | 0.040 | <0.0001 | 3 | 0 | 15 | aromatic amino acid family catabolic process |
| GO:0009584 | 0.040 | <0.0001 | 3 | 0 | 15 | detection of visible light |
| GO:0015813 | 0.040 | <0.0001 | 3 | 0 | 15 | L-glutamate transport |
| GO:0034199 | 0.040 | <0.0001 | 3 | 0 | 15 | activation of protein kinase A activity |
| GO:0034312 | 0.040 | <0.0001 | 3 | 0 | 15 | diol biosynthetic process |
| GO:0035455 | 0.040 | <0.0001 | 3 | 0 | 15 | response to interferon-alpha |
| GO:0042269 | 0.040 | <0.0001 | 3 | 0 | 15 | regulation of natural killer cell mediated cytotoxicity |
| GO:0042403 | 0.040 | <0.0001 | 3 | 0 | 15 | thyroid hormone metabolic process |
| GO:0042423 | 0.040 | <0.0001 | 3 | 0 | 15 | catecholamine biosynthetic process |
| GO:0042537 | 0.040 | <0.0001 | 3 | 0 | 15 | benzene-containing compound metabolic process |
| GO:0043304 | 0.040 | <0.0001 | 3 | 0 | 15 | regulation of mast cell degranulation |
| GO:0046189 | 0.040 | <0.0001 | 3 | 0 | 15 | phenol-containing compound biosynthetic process |
| GO:0050691 | 0.040 | <0.0001 | 3 | 0 | 15 | regulation of defense response to virus by host |
| GO:0010594 | 0.041 | 0.463 | 12 | 6 | 60 | regulation of endothelial cell migration |
| GO:0030902 | 0.041 | 0.512 | 14 | 8 | 73 | hindbrain development |
| GO:0002688 | 0.042 | 0.434 | 10 | 5 | 53 | regulation of leukocyte chemotaxis |
| GO:0008344 | 0.042 | 0.434 | 10 | 5 | 53 | adult locomotory behavior |
| GO:0031646 | 0.042 | 0.434 | 10 | 5 | 53 | positive regulation of neurological system process |
| GO:0051897 | 0.042 | 0.434 | 10 | 5 | 53 | positive regulation of protein kinase B signaling cascade |
| GO:0060048 | 0.042 | 0.434 | 10 | 5 | 53 | cardiac muscle contraction |
| GO:0051592 | 0.042 | 0.513 | 14 | 8 | 73 | response to calcium ion |
| GO:0006635 | 0.042 | 0.397 | 9 | 4 | 46 | fatty acid beta-oxidation |
| GO:0030593 | 0.042 | 0.397 | 9 | 4 | 46 | neutrophil chemotaxis |
| GO:0033555 | 0.042 | 0.397 | 9 | 4 | 46 | multicellular organismal response to stress |
| GO:0070887 | 0.044 | 0.867 | 230 | 208 | 1184 | cellular response to chemical stimulus |
| GO:0007215 | 0.045 | 0.288 | 6 | 2 | 31 | glutamate receptor signaling pathway |
| GO:0019935 | 0.045 | 0.288 | 6 | 2 | 31 | cyclic-nucleotide-mediated signaling |
| GO:0050994 | 0.045 | 0.288 | 6 | 2 | 31 | regulation of lipid catabolic process |
| GO:0002279 | 0.047 | 0.190 | 4 | 1 | 23 | mast cell activation involved in immune response |
| GO:0002448 | 0.047 | 0.190 | 4 | 1 | 23 | mast cell mediated immunity |
| GO:0002889 | 0.047 | 0.190 | 4 | 1 | 23 | regulation of immunoglobulin mediated immune response |
| GO:0030809 | 0.047 | 0.190 | 4 | 1 | 23 | negative regulation of nucleotide biosynthetic process |
| GO:0030818 | 0.047 | 0.190 | 4 | 1 | 23 | negative regulation of cAMP biosynthetic process |
| GO:0032760 | 0.047 | 0.190 | 4 | 1 | 23 | positive regulation of tumor necrosis factor production |
| GO:0002040 | 0.048 | 0.358 | 7 | 3 | 38 | sprouting angiogenesis |
| GO:0007189 | 0.048 | 0.358 | 7 | 3 | 38 | adenylate cyclase-activating G-protein coupled receptor signaling pathway |
| GO:0046390 | 0.048 | 0.688 | 34 | 25 | 176 | ribose phosphate biosynthetic process |
| GO:0035150 | 0.049 | 0.588 | 19 | 12 | 97 | regulation of tube size |
| GO:0031347 | 0.049 | 0.695 | 35 | 26 | 180 | regulation of defense response |
| GO:0006487 | 0.049 | 0.503 | 13 | 7 | 65 | protein N-linked glycosylation |
| GO:0002090 | 0.050 | <0.0001 | 3 | 0 | 14 | regulation of receptor internalization |
| GO:0007608 | 0.050 | <0.0001 | 3 | 0 | 14 | sensory perception of smell |
| GO:0007617 | 0.050 | <0.0001 | 3 | 0 | 14 | mating behavior |
| GO:0014743 | 0.050 | <0.0001 | 3 | 0 | 14 | regulation of muscle hypertrophy |
| GO:0021533 | 0.050 | <0.0001 | 3 | 0 | 14 | cell differentiation in hindbrain |
| GO:0021680 | 0.050 | <0.0001 | 3 | 0 | 14 | cerebellar Purkinje cell layer development |
| GO:0035809 | 0.050 | <0.0001 | 3 | 0 | 14 | regulation of urine volume |
| GO:0042523 | 0.050 | <0.0001 | 3 | 0 | 14 | positive regulation of tyrosine phosphorylation of Stat5 protein |
| GO:0043691 | 0.050 | <0.0001 | 3 | 0 | 14 | reverse cholesterol transport |
| GO:0044110 | 0.050 | <0.0001 | 3 | 0 | 14 | growth involved in symbiotic interaction |
| GO:0044117 | 0.050 | <0.0001 | 3 | 0 | 14 | growth of symbiont in host |
| GO:0045577 | 0.050 | <0.0001 | 3 | 0 | 14 | regulation of B cell differentiation |
| GO:0045671 | 0.050 | <0.0001 | 3 | 0 | 14 | negative regulation of osteoclast differentiation |
| GO:0097028 | 0.050 | <0.0001 | 3 | 0 | 14 | dendritic cell differentiation |
