# Supplementary material for: Hidden Treasures in “Ancient” Microarrays: Gene-Expression Portrays Biology and Potential Resistance Pathways of Major Lung Cancer Subtypes and Normal Tissue
Source: Front Oncol. 2014 Sep 29;4:251. doi: 10.3389/fonc.2014.00251 (PMC4178426; doi:10.3389/fonc.2014.00251)
Supplement: Figure S1 — Clinically relevant differentially expressed pathways in each tissue type versus the rest. The pathway maps are arranged according to the scheme in the left-top corner. Red indicates overexpression in genes, dark green downregulation. In the Cell Cycle pathway, tumor suppressor genes were overexpressed in normal lung tissue while oncogenes and tumor driving cyclins were overexpressed in cancers. The gene thymidylate synthase (TYMS or TS, belonging to the pathway “one carbon pool by folate”) is relevant for tumor growth and is also a treatment target. Notably, TYMS was overexpressed only in the tumors that are generally refractory to the drug pemetrexed, as the squamous and the small-cell lung cancer. TYMS was not overexpressed in mesothelioma, but it is known that TYMS expression is highly variable in this cancer. In the ERBB pathway, the ERBB2/HER2 and ERBB3/HER3 were overexpressed in adenocarcinoma, while the ERBB4 was overexpressed in the carcinoids. [file Data_Sheet_1.ZIP › File S4.ZIP!/KEGG Analysis/Normals vs Rest Enrichment Analysis/Overrepresented KEGG terms of Up-regulated Genes.html]

Gene to KEGG test for over-representation

| KEGGID | Pvalue | OddsRatio | ExpCount | Count | Size | Term |
| --- | --- | --- | --- | --- | --- | --- |
| 04810 | <0.0001 | 2.710 | 29 | 58 | 171 | Regulation of actin cytoskeleton |
| 04510 | <0.0001 | 2.598 | 30 | 60 | 182 | Focal adhesion |
| 05416 | <0.0001 | 4.795 | 9 | 27 | 56 | Viral myocarditis |
| 04380 | <0.0001 | 3.071 | 19 | 43 | 116 | Osteoclast differentiation |
| 04270 | <0.0001 | 3.457 | 15 | 36 | 90 | Vascular smooth muscle contraction |
| 05140 | <0.0001 | 4.341 | 10 | 27 | 59 | Leishmaniasis |
| 04145 | <0.0001 | 3.047 | 19 | 41 | 111 | Phagosome |
| 05144 | <0.0001 | 5.126 | 8 | 23 | 46 | Malaria |
| 04062 | <0.0001 | 2.545 | 27 | 52 | 159 | Chemokine signaling pathway |
| 04670 | <0.0001 | 3.120 | 16 | 35 | 93 | Leukocyte transendothelial migration |
| 05150 | <0.0001 | 4.611 | 7 | 19 | 40 | Staphylococcus aureus infection |
| 04514 | <0.0001 | 2.652 | 17 | 34 | 100 | Cell adhesion molecules (CAMs) |
| 05332 | <0.0001 | 5.915 | 4 | 14 | 26 | Graft-versus-host disease |
| 05323 | <0.0001 | 2.942 | 12 | 27 | 74 | Rheumatoid arthritis |
| 04666 | <0.0001 | 2.869 | 13 | 28 | 78 | Fc gamma R-mediated phagocytosis |
| 04144 | <0.0001 | 2.166 | 25 | 44 | 149 | Endocytosis |
| 04640 | <0.0001 | 2.653 | 14 | 28 | 82 | Hematopoietic cell lineage |
| 04010 | <0.0001 | 1.837 | 36 | 57 | 218 | MAPK signaling pathway |
| 04142 | <0.0001 | 2.299 | 17 | 31 | 100 | Lysosome |
| 05145 | <0.0001 | 2.225 | 18 | 33 | 109 | Toxoplasmosis |
| 05146 | <0.0001 | 2.289 | 16 | 30 | 97 | Amoebiasis |
| 04650 | 0.001 | 2.158 | 17 | 30 | 101 | Natural killer cell mediated cytotoxicity |
| 04060 | 0.001 | 1.742 | 35 | 53 | 210 | Cytokine-cytokine receptor interaction |
| 05100 | 0.001 | 2.537 | 10 | 20 | 60 | Bacterial invasion of epithelial cells |
| 04614 | 0.001 | 5.749 | 3 | 8 | 15 | Renin-angiotensin system |
| 05142 | 0.001 | 2.149 | 15 | 27 | 91 | Chagas disease (American trypanosomiasis) |
| 05330 | 0.001 | 3.562 | 5 | 12 | 29 | Allograft rejection |
| 04530 | 0.003 | 2.020 | 16 | 27 | 95 | Tight junction |
| 04664 | 0.004 | 2.202 | 11 | 20 | 66 | Fc epsilon RI signaling pathway |
| 04610 | 0.005 | 2.236 | 10 | 19 | 62 | Complement and coagulation cascades |
| 04662 | 0.005 | 2.154 | 11 | 20 | 67 | B cell receptor signaling pathway |
| 04612 | 0.009 | 2.244 | 9 | 16 | 52 | Antigen processing and presentation |
| 04940 | 0.009 | 2.628 | 6 | 12 | 35 | Type I diabetes mellitus |
| 05322 | 0.009 | 2.115 | 10 | 18 | 61 | Systemic lupus erythematosus |
| 05310 | 0.012 | 3.015 | 4 | 9 | 24 | Asthma |
| 04621 | 0.012 | 2.276 | 8 | 14 | 45 | NOD-like receptor signaling pathway |
| 05131 | 0.015 | 2.069 | 9 | 16 | 55 | Shigellosis |
| 04972 | 0.019 | 1.836 | 13 | 20 | 75 | Pancreatic secretion |
| 04630 | 0.019 | 1.618 | 21 | 30 | 124 | Jak-STAT signaling pathway |
| 05143 | 0.020 | 2.513 | 5 | 10 | 30 | African trypanosomiasis |
| 04520 | 0.020 | 1.892 | 11 | 18 | 66 | Adherens junction |
| 05410 | 0.024 | 1.809 | 12 | 19 | 72 | Hypertrophic cardiomyopathy (HCM) |
| 05020 | 0.025 | 2.392 | 5 | 10 | 31 | Prion diseases |
| 05320 | 0.026 | 2.155 | 7 | 12 | 40 | Autoimmune thyroid disease |
| 00565 | 0.028 | 2.925 | 3 | 7 | 19 | Ether lipid metabolism |
| 00590 | 0.029 | 2.211 | 6 | 11 | 36 | Arachidonic acid metabolism |
| 04672 | 0.029 | 2.211 | 6 | 11 | 36 | Intestinal immune network for IgA production |
| 05414 | 0.032 | 1.710 | 13 | 20 | 79 | Dilated cardiomyopathy |
| 00920 | 0.036 | 7.498 | 1 | 3 | 5 | Sulfur metabolism |
| 05130 | 0.038 | 2.010 | 7 | 12 | 42 | Pathogenic Escherichia coli infection |
| 05200 | 0.046 | 1.318 | 47 | 58 | 282 | Pathways in cancer |
| 00350 | 0.046 | 2.091 | 6 | 10 | 34 | Tyrosine metabolism |
| 04512 | 0.046 | 1.650 | 13 | 19 | 77 | ECM-receptor interaction |
