# Supplementary material for: Hidden Treasures in “Ancient” Microarrays: Gene-Expression Portrays Biology and Potential Resistance Pathways of Major Lung Cancer Subtypes and Normal Tissue
Source: Front Oncol. 2014 Sep 29;4:251. doi: 10.3389/fonc.2014.00251 (PMC4178426; doi:10.3389/fonc.2014.00251)
Supplement: Figure S1 — Clinically relevant differentially expressed pathways in each tissue type versus the rest. The pathway maps are arranged according to the scheme in the left-top corner. Red indicates overexpression in genes, dark green downregulation. In the Cell Cycle pathway, tumor suppressor genes were overexpressed in normal lung tissue while oncogenes and tumor driving cyclins were overexpressed in cancers. The gene thymidylate synthase (TYMS or TS, belonging to the pathway “one carbon pool by folate”) is relevant for tumor growth and is also a treatment target. Notably, TYMS was overexpressed only in the tumors that are generally refractory to the drug pemetrexed, as the squamous and the small-cell lung cancer. TYMS was not overexpressed in mesothelioma, but it is known that TYMS expression is highly variable in this cancer. In the ERBB pathway, the ERBB2/HER2 and ERBB3/HER3 were overexpressed in adenocarcinoma, while the ERBB4 was overexpressed in the carcinoids. [file Data_Sheet_1.ZIP › File S4.ZIP!/KEGG Analysis/Squamous vs Rest Enrichment Analysis/Underrepresented KEGG terms of Up-regulated Genes.html]

Gene to KEGG test for under-representation

| KEGGID | Pvalue | OddsRatio | ExpCount | Count | Size | Term |
| --- | --- | --- | --- | --- | --- | --- |
| 04060 | <0.0001 | 0.188 | 42 | 10 | 210 | Cytokine-cytokine receptor interaction |
| 04080 | <0.0001 | 0.178 | 40 | 9 | 200 | Neuroactive ligand-receptor interaction |
| 04630 | <0.0001 | 0.267 | 25 | 8 | 124 | Jak-STAT signaling pathway |
| 05320 | <0.0001 | <0.0001 | 8 | 0 | 40 | Autoimmune thyroid disease |
| 04020 | <0.0001 | 0.402 | 30 | 14 | 149 | Calcium signaling pathway |
| 04672 | <0.0001 | <0.0001 | 7 | 0 | 36 | Intestinal immune network for IgA production |
| 04640 | <0.0001 | 0.253 | 16 | 5 | 82 | Hematopoietic cell lineage |
| 04610 | 0.001 | 0.199 | 12 | 3 | 62 | Complement and coagulation cascades |
| 04270 | 0.001 | 0.329 | 18 | 7 | 90 | Vascular smooth muscle contraction |
| 05150 | 0.001 | 0.101 | 8 | 1 | 40 | Staphylococcus aureus infection |
| 05330 | 0.001 | <0.0001 | 6 | 0 | 29 | Allograft rejection |
| 04514 | 0.002 | 0.386 | 20 | 9 | 100 | Cell adhesion molecules (CAMs) |
| 05332 | 0.003 | <0.0001 | 5 | 0 | 26 | Graft-versus-host disease |
| 04380 | 0.003 | 0.450 | 23 | 12 | 116 | Osteoclast differentiation |
| 04940 | 0.004 | 0.116 | 7 | 1 | 35 | Type I diabetes mellitus |
| 05323 | 0.004 | 0.346 | 15 | 6 | 74 | Rheumatoid arthritis |
| 05140 | 0.004 | 0.285 | 12 | 4 | 59 | Leishmaniasis |
| 05310 | 0.005 | <0.0001 | 5 | 0 | 24 | Asthma |
| 04070 | 0.006 | 0.333 | 13 | 5 | 64 | Phosphatidylinositol signaling system |
| 04970 | 0.008 | 0.374 | 14 | 6 | 69 | Salivary secretion |
| 04744 | 0.009 | <0.0001 | 4 | 0 | 21 | Phototransduction |
| 05143 | 0.010 | 0.136 | 6 | 1 | 30 | African trypanosomiasis |
| 05144 | 0.010 | 0.275 | 9 | 3 | 46 | Malaria |
| 05160 | 0.010 | 0.494 | 22 | 12 | 107 | Hepatitis C |
| 04623 | 0.011 | 0.219 | 8 | 2 | 38 | Cytosolic DNA-sensing pathway |
| 04612 | 0.013 | 0.328 | 10 | 4 | 52 | Antigen processing and presentation |
| 05414 | 0.013 | 0.442 | 16 | 8 | 79 | Dilated cardiomyopathy |
| 04740 | 0.018 | 0.239 | 7 | 2 | 35 | Olfactory transduction |
| 04930 | 0.020 | 0.303 | 8 | 3 | 42 | Type II diabetes mellitus |
| 04142 | 0.023 | 0.535 | 20 | 12 | 100 | Lysosome |
| 04976 | 0.023 | 0.394 | 11 | 5 | 55 | Bile secretion |
| 04062 | 0.024 | 0.628 | 32 | 22 | 159 | Chemokine signaling pathway |
| 00380 | 0.026 | 0.255 | 7 | 2 | 33 | Tryptophan metabolism |
| 00830 | 0.026 | 0.255 | 7 | 2 | 33 | Retinol metabolism |
| 05340 | 0.030 | 0.263 | 6 | 2 | 32 | Primary immunodeficiency |
| 05142 | 0.031 | 0.540 | 18 | 11 | 91 | Chagas disease (American trypanosomiasis) |
| 04614 | 0.034 | <0.0001 | 3 | 0 | 15 | Renin-angiotensin system |
| 00531 | 0.034 | <0.0001 | 3 | 0 | 15 | Glycosaminoglycan degradation |
| 04650 | 0.039 | 0.580 | 20 | 13 | 101 | Natural killer cell mediated cytotoxicity |
| 04971 | 0.042 | 0.464 | 11 | 6 | 57 | Gastric acid secretion |
| 04972 | 0.047 | 0.537 | 15 | 9 | 75 | Pancreatic secretion |
| 05416 | 0.048 | 0.473 | 11 | 6 | 56 | Viral myocarditis |
